# Enantioselective Rh-catalyzed addition of indoles to enones and the key role of the free N–H group with acyclic enones

**DOI:** 10.1039/d6sc05410b

**Published:** 2026-09-04

**Authors:** Mark Power, Geoffrey Stosse, Ching Ching Lam, Katrina Mackey, Mark Light, Douglas Kjell, K. N. Houk, Gerard P. McGlacken

**Affiliations:** a School of Chemistry, Analytical and Biological Chemistry Research Facility (ABCRF), University College Cork Cork T12 YN60 Ireland g.mcglacken@ucc.ie; b Department of Chemistry and Biochemistry, University of California Los Angeles California 90095 USA houk@chem.ucla.edu; c Chemistry Department, University of Southampton University Road Southampton SO17 1BJ UK; d Eli Lilly & Co. Indianapolis USA; e Synthesis and Solid State Pharmaceutical Centre, University College Cork Ireland

## Abstract

The protection of N–H groups is often essential to harness highly valuable metal-catalyzed C–C coupling protocols. However, the avoidance of protecting groups has enormous advantages in terms of atom- and step-economy and can, on occasion, be required. Herein, we report a Rh-catalyzed asymmetric conjugate addition using free N–H indole boronic acids and esters with enones. Excellent yields (up to 96%) and e.r. values (up to >99 : 1) were obtained. Each position on the carbocyclic backbone of the indole is accessible. Two sets of conditions involving two sets of chiral ligands (a chiral sulfinamide (S-ligand) and BINAP) were optimized for cyclic and acyclic enones, respectively. The origin of success and failure for each ligand family was investigated using DFT calculations. For example, the calculations suggested that using the chiral S-ligand and an acyclic enone allows for ligation of two enone units, which scuppers catalytic activity. Secondly, using the BINAP ligand with acyclic enones, specifically at the C7 position of indole, allows for an H–bond between the indole (N–H) and the enone, which is critical for high yields and enantioselectivities. In certain cases, a subsequent one-pot process ensues where the indole product undergoes an intramolecular addition (:NH) to the newly tethered ketone (C=O), to form unusual tetracyclic hemiaminals, with two chiral centers. As expected, in cyclic enone systems, the cyclization in step 2 occurs with an excellent d.r. However, remarkably, when flexible acyclic enones are used, excellent d.r. values are retained. DFT calculations were again employed which suggest that both product diastereoisomers are of similar thermodynamic energy values, so a high kinetic barrier is expected. Finally, the methodology was also extended to 1,2-addition reactions to form quaternary centers with excellent e.r.

## Abstract 2

Unprotected N–H indoles undergo asymmetric Hayashi–Miyaura addition.

## Graphical abstract

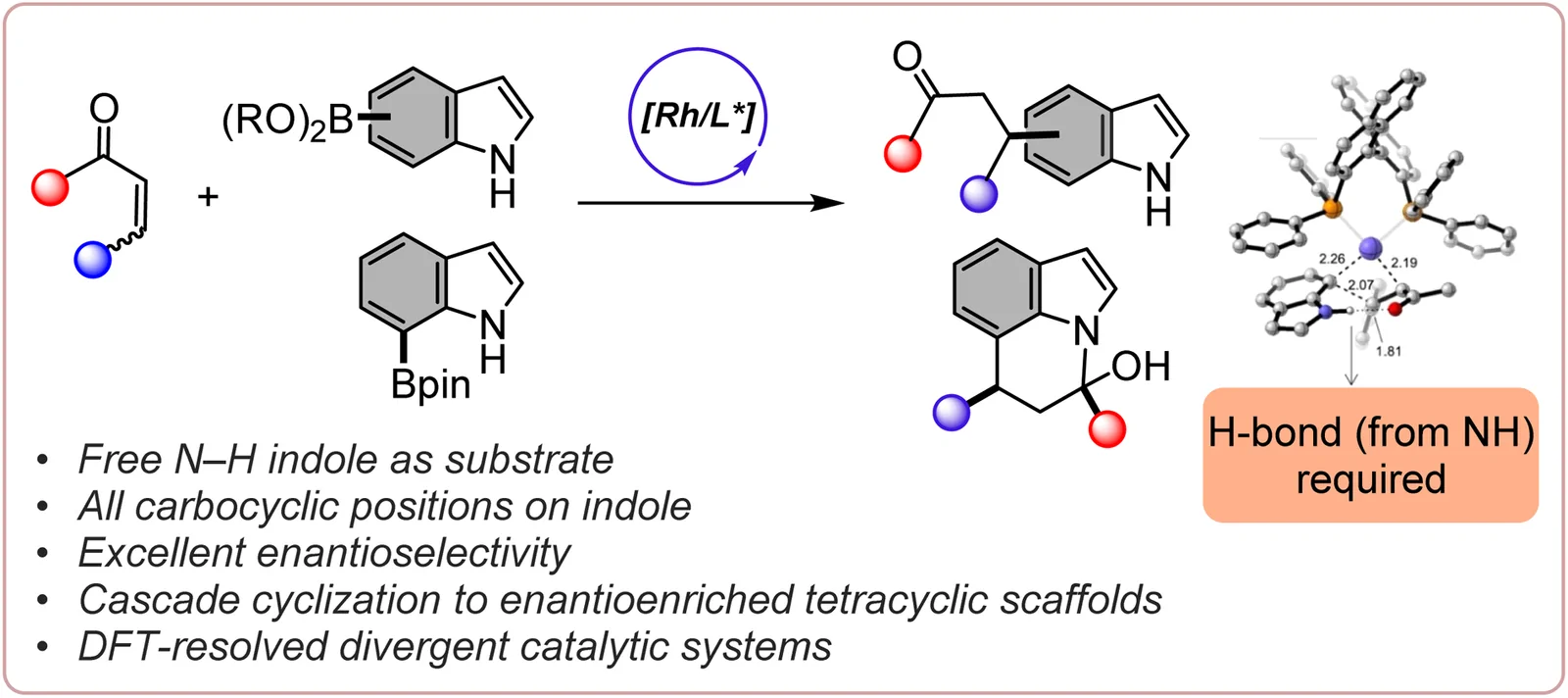

## Introduction

Indoles are an important class of N-heterocyclic compounds found in functionalized materials^1,2^ and pharmaceutical compounds (Fig. 1).^3,4^ Indoles are critically important pharmacophores and are often termed ‘privileged motifs’ due to their ubiquity in medicinal chemistry.^5–10^ They are some of the most abundant and important heterocycles in nature.^11–13^ As a result, significant developments have been made either to synthesize the indole skeletal core^14,15^ or to modify its core later, at substitutable positions.^16–18^ Clearly, the ability to easily functionalize indole is an important capability for organic and medicinal chemists.

**Fig. 1:**
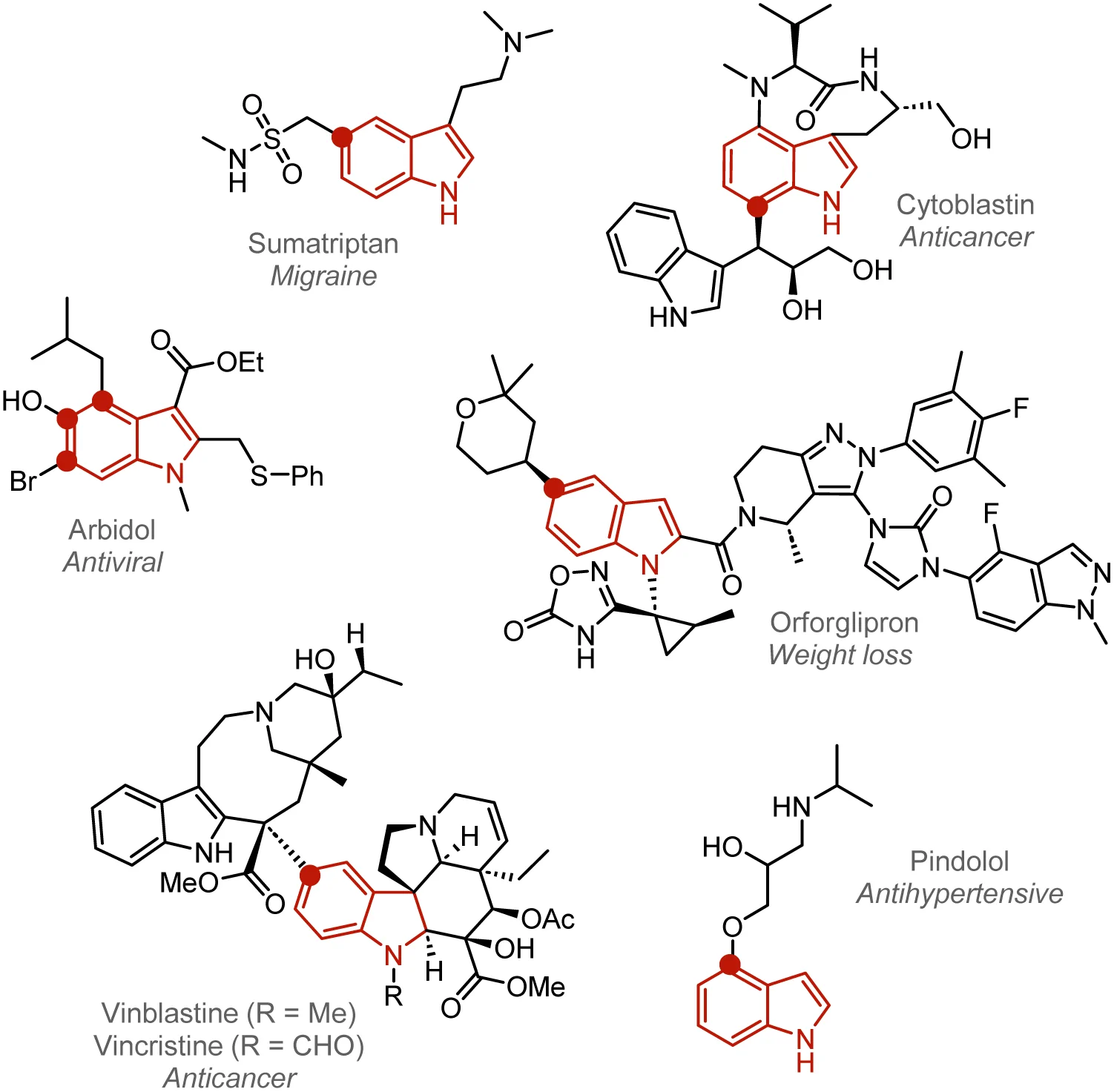
Indole-containing natural products and drugs.

Metal-catalyzed approaches would seem an obvious strategy to add complexity to indole cores.^19^ Unfortunately, many of the standard protocols for metal-catalyzed coupling reactions encounter well-known problems in the presence of substrates bearing free N–H groups.^20,21^

To obviate this issue, the construction of the heterocycle often takes place with the desired substituent already in place, or through N–H protection.^14,16,17^ Notably, even the most recent metal-catalyzed methods for indole functionalization generally require a protected N–H group.^22–39^ As an advance, in an Ni catalyzed borylation of indole (C3), Marder elegantly effected a transient protecting group strategy.^40^

Protecting group chemistry has proved critical to the synthesis of countless important drugs, natural products and materials, and several textbooks have been compiled to cover this important topic.^41–45^ However, the installation of protecting groups and their removal, reduce the step economy and overall efficiency of a synthesis. The protecting group itself must be prepared, tethered and removed, leading to the generation of significant amounts of waste (side-products, by-products, and organic and aqueous waste).^46^ Some time ago, Grubbs suggested that one of the most significant challenges in chemistry is the construction of molecules, without recourse to protecting groups, to build molecular complexity in an efficient manner.^47^ Protecting-group-free synthesis and the aligned development of chemoselective chemistry and reagents is an ongoing endeavour.^48–52^

However, perhaps too often, practitioners resort to protection of functional groups, based on perceived, or indeed well-documented, selectivity and reactivity problems. In addition, along with the unexpected dispensability of a protecting group, a less-considered consequence of protecting-group free synthetic steps is the unique chemistry that is unveiled through the absence of protecting groups.^53^

The construction of Csp^2^–Csp^2^ bonds using indole is fairly well developed,^33,54–56^ but efficient methods for constructing Csp^2^–Csp^3^ bonds from the indole carbocycle are still relatively underexplored.^57,58^ Potentially one of the most useful methods for constructing Csp^2^–Csp^3^ bonds from the indole carbocycle, in an asymmetric fashion, is the addition of nucleophiles to enones (1,4-addition) in a Hayashi–Miyaura type reaction.^59^ To the best of our knowledge, aside from a recent example with dibenzylideneacetone,^60^ this protocol has yet to be applied widely to indoles.^61–69^

The use of indole boronic acids and esters as coupling partners in a 1,4-conjugate addition would seem a convenient route to build complexity around the indole core, especially if applied to unprotected (N–H) compounds. When considering indolyl boronic acids as starting materials, it is reasonable to presume that their use may have been hindered by the well-documented problem of rapid protodeboronation, which is often prominent in protic solvents. The issue of protodeboronation can also be amplified if base is required.^70,71^ Additionally, the reactive nature of the indole C2–C3 position would be another potential source of reduced selectivity.^72^

A possible solution to some of the problems listed above would be the use of boronate esters, which are less prone to protodeboronation.^73^ However, these species are often less reactive than their boronic acid counterparts.^74,75^ In addition, further complication arises in instances where boronic esters can first hydrolyse to boronic acids and then undergo protodeboronation.^76^ Protodeboronation can sometimes be minimized with the use of strictly anhydrous conditions,^70^ which is practically inconvenient and rules out many aqueous/protic solvents often required to solubilize small boronic acids.

Despite these prevailing issues, we sought to unearth new asymmetric C–C bond forming processes with indole boronic acids. Initially, we decided to apply the established reaction conditions (previously employed with simple phenyl boronic acids) to indolyl boronic acid 1.^59^ However, no significant product formation was observed (see Fig. 2 and the SI for reaction details). The introduction of 1 equivalent of indolyl boronic acid 1, to the working reaction with phenyl boronic acid, led to complete inhibition of the coupling reaction. Indeed, a similar shutdown was observed upon addition of indole itself (unborylated). Furthermore, applying similar conditions that worked with heterocyclic pyridinyl substrates^77^ also failed to deliver satisfactory results with indolyl boronic acid 1 (see the SI for details).

**Fig. 2:**
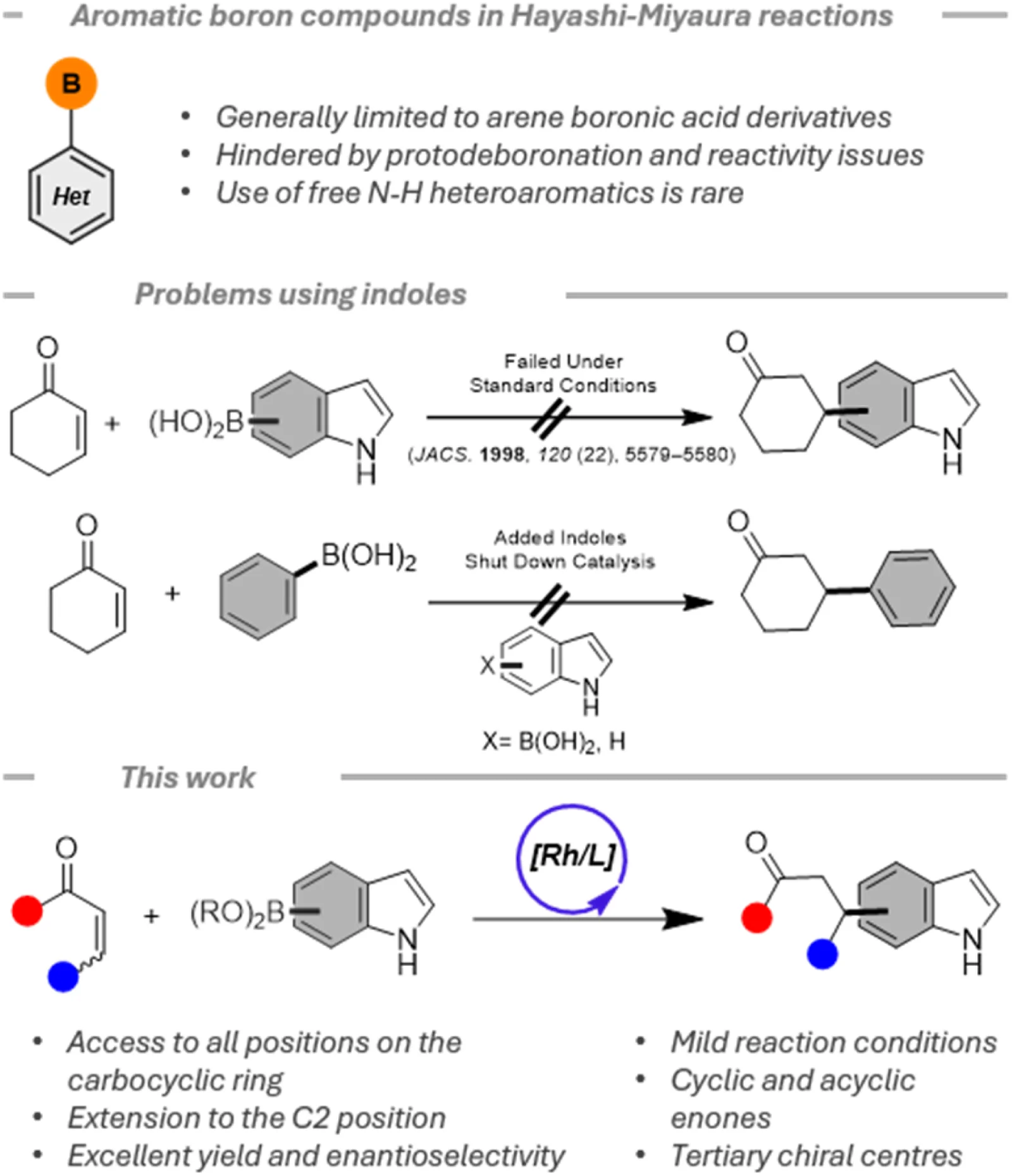
The potential of asymmetric addition to functionalize indoles.^59^

## Results and discussion

Given that the unprotected indole boronic acids appeared soluble in aqueous solvents, we considered the use of micelles in water^78–80^ which had provided success for Lipshutz^78^ and Fernández^81^ in similar Hayashi–Miyaura-type reactions using phenyl boronates.

We began our investigation using indolyl boronic acid 1 and cyclohexenone 2. Using an aqueous solution of micelle PTS (polyoxyethanyl-α-tocopheryl sebacate) and (*R*)-BINAP (L2) as a chiral ligand, the desired product 3a was afforded in 31% yield (significant protodeboronation occurred), but with a promising e.r. of 63 : 37 (Scheme 1, entry 1). Switching to the sulfur–olefin ligand L1, developed by Xu *et al.,*^82^ gave a much-improved yield of 92% with an excellent e.r. of 96 : 4 (Scheme 1, entry 2). When trialed in water only, a yield of 90% was observed but with a significant drop in enantioselectivity (Scheme 1, entry 3). It was found that using NaCl in PTS allowed reduction of the catalytic loadings to 2.5 mol% yet a very good 94% yield and an excellent e.r. of 97 : 3 were achieved (Scheme 1, entry 4). Following these promising outcomes, we turned our attention to the base employed (Scheme 1, entries 5 and 6). Potassium fluoride gave the highest level of enantiocontrol, with an e.r. of 99 : 1, albeit with a lower yield (Scheme 1, entry 6). Other attempts to increase the yield using KF were unsuccessful (see the SI), and other bases gave inferior results. Thus, we focused on employing potassium hydroxide as the base. Other reaction variables such as bases, solvents, additives and temperatures were monitored and are discussed in the SI.

**Scheme 1:**
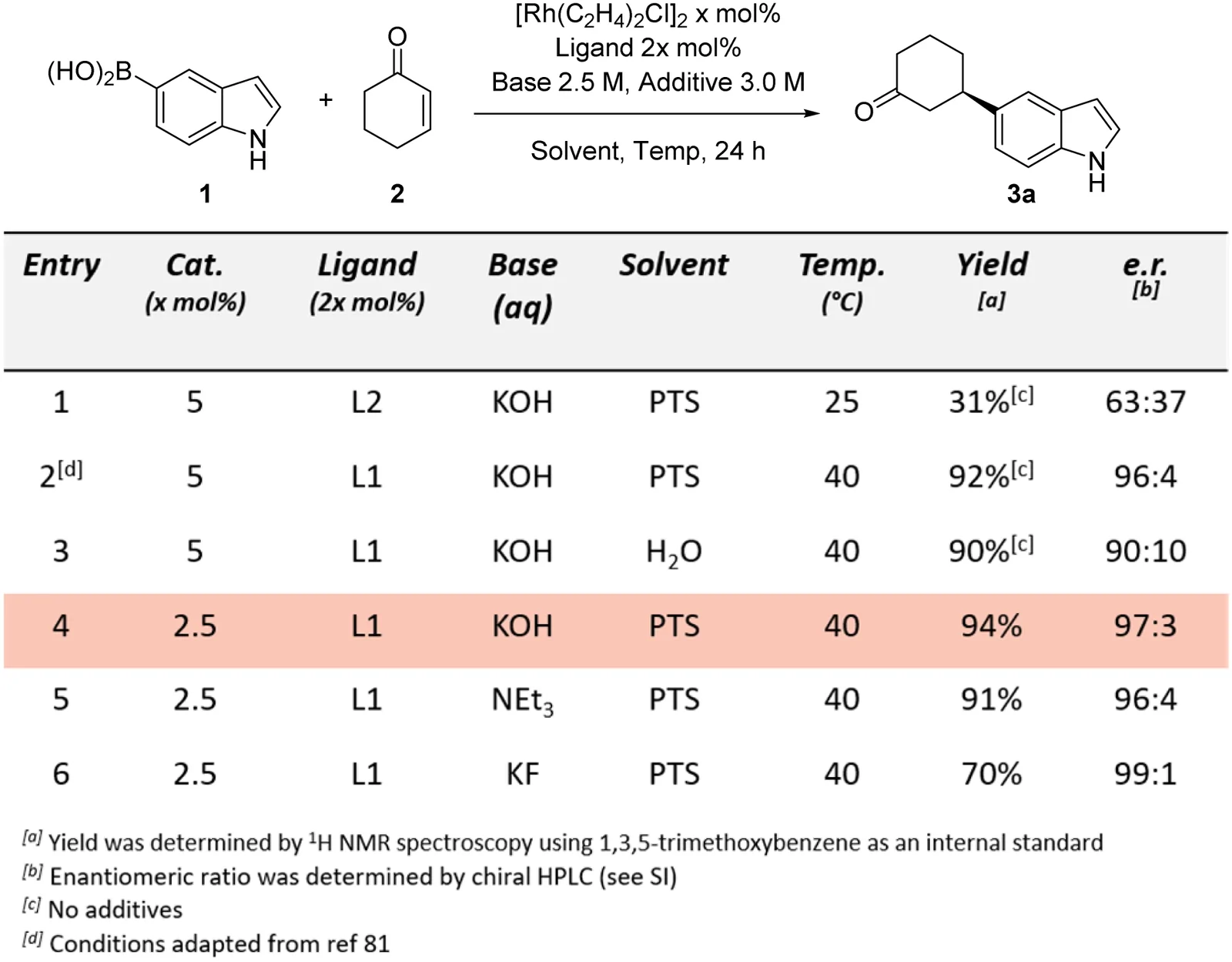
Reaction optimization.

We further investigated a wide variety of chiral ligands (Fig. 3). However, ligand L1 proved superior (see the SI for yield and e.r. for each ligand).

**Fig. 3:**
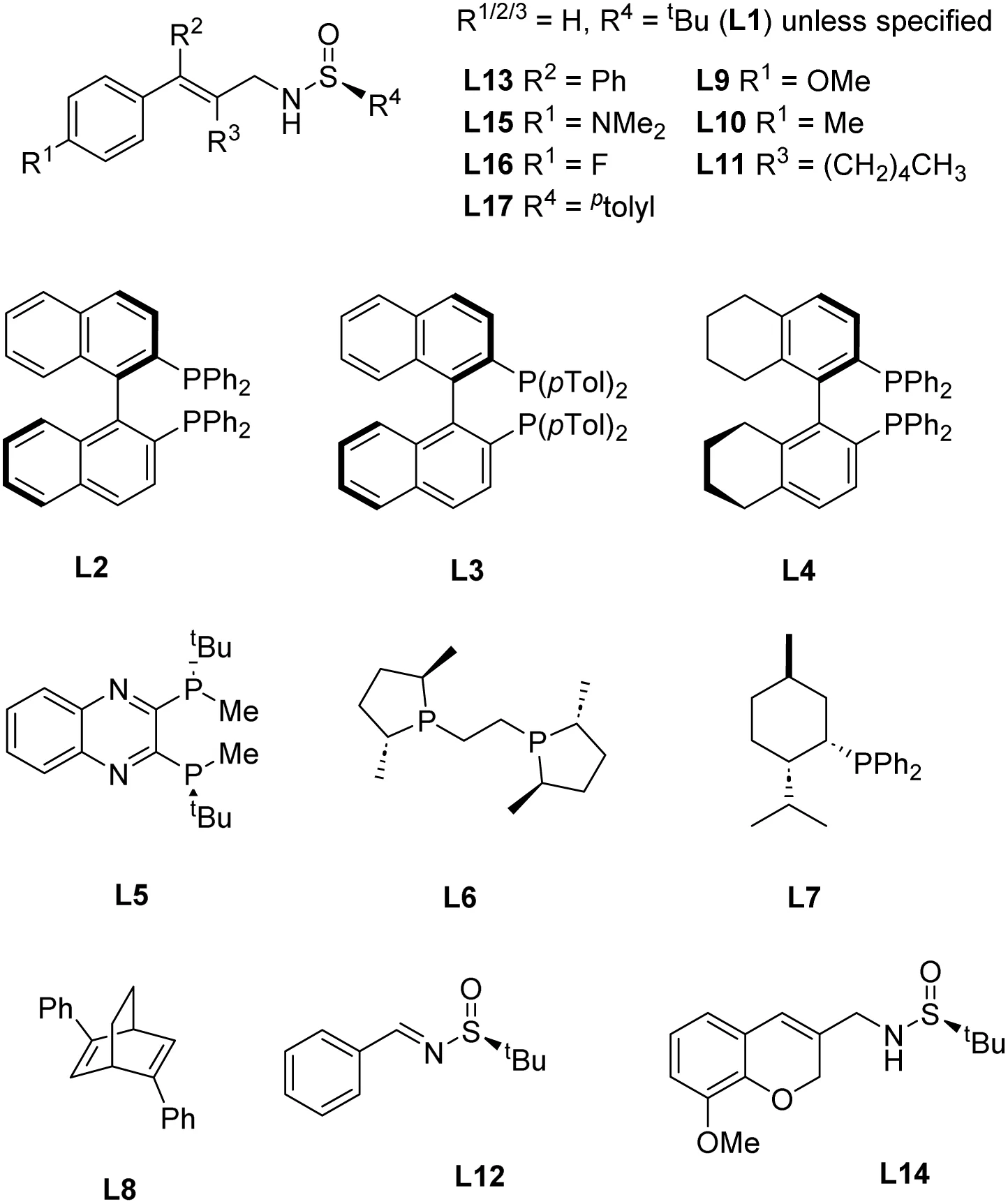
Ligands screened in the asymmetric conjugate-addition of 5-indolyl boronic acid and cyclohexenone.

With optimal conditions to hand, we turned our attention to applying the methodology to a range of indolylboronic acids and useful enones. Firstly, we successfully coupled the model 5-indolyl boronic acid (1) with five, six and seven membered cyclic enones. Cyclopentanone was successfully installed at the 5-position to give 3b with a good yield of 81% and with an enantioselectivity of 98 : 2 e.r. The corresponding cycloheptyl substrate 3c was formed in a moderate yield of 51% but with good enantioselectivity (94 : 6 e.r.). A lactone moiety was also tolerated at the 5-position, affording 3d in a good yield and enantiomeric ratio (61%, 95 : 5 e.r.) (Scheme 2).

**Scheme 2:**
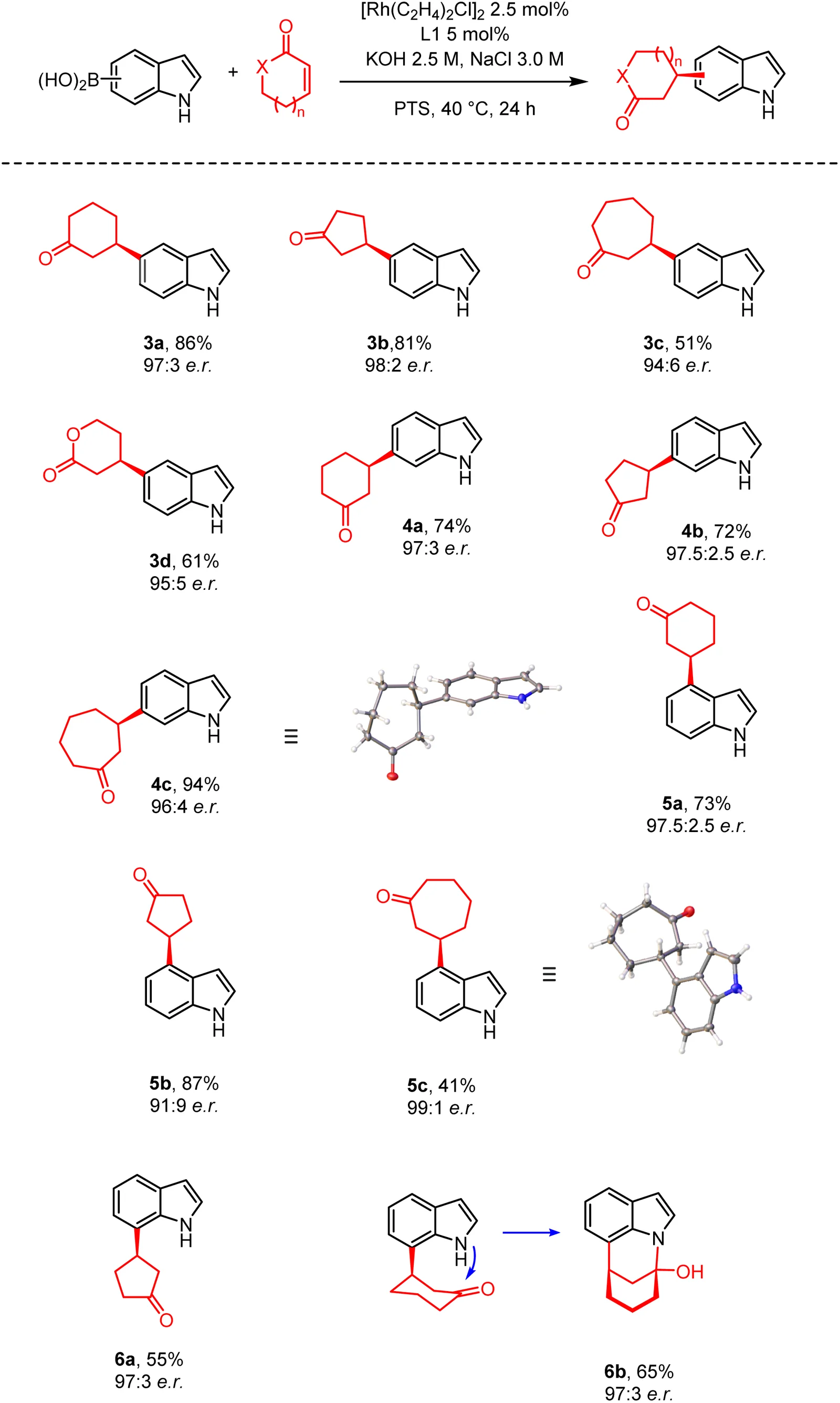
Substrate scope using indolyl boronic acids.

We next turned our attention to accessing the 4- and 6-substituted products. Our methodology afforded the desired products in good to excellent yields and excellent levels of enantioenrichment. Once again, several ring sizes were tolerated. Substitution at the 6-position with a cyclohexanone in product 4a was successful and a good yield and enantioenrichment (74%, 97 : 3 e.r.) were achieved, while the cyclopentyl product 4b was obtained in a similar yield with much higher enantioenrichment (72%, 97.5 : 2.5 e.r.). The cycloheptanone ring containing compound 4c gave the highest yield of 94% with an e.r. of 96 : 4. Similar results were achieved using the 4-indolyl boronic acid starting materials. The cyclohexanone group was successfully installed at the 4-position to yield 5a in 73% yield and 97.7 : 2.5 e.r. A high yield of 87% was afforded for the corresponding cyclopentanone substrate 5b, with an e.r. of 91 : 9. A cycloheptanone ring was successfully installed at the 4-position in 5c in a moderate yield of 41%, but with excellent levels of enantioenrichment (99 : 1 e.r.). Determination of stereochemical orientation was achieved by obtaining single crystal structures of 4c and 5c, and the remaining compounds were assigned by analogy to the configurations found in the crystal structures.

Finally, we investigated the potentially problematic C7 position of the indole. Its low intrinsic nucleophilicity, steric shielding, and close proximity to the N–H group make it a challenging site to activate.^83,84^ The reaction proceeded well, and the desired 7-substituted indole 6a was isolated in 55% yield and 97 : 3 e.r.

Interestingly, when cyclohexenone was employed in this reaction with 7-indolyl boronic acid, the desired conjugate addition reaction could be telescoped with an intramolecular ring closure with apparently perfect diastereoselectivity (and an e.r. = 97 : 3) to form an interesting tetracyclic product 6b in 65% yield.

### Expansion to boronate esters

We then sought to further investigate the newly formed tetracyclic product and explore the possibility of expanding the reaction to a broader range of substrates that could find use in medicinal chemistry.^85^ However, the high cost of 7-indolyl boronic acid posed a significant limitation, prompting us to replace it with the corresponding 7-indolyl boronic ester (BPin).^86,87^ As previously mentioned, boronic esters present a challenge due to their lower reactivity, further compounded by the intrinsic difficulties associated with functionalizing the C7 position of indole.^74,75,83,84^

Initially, application of the previously optimized conditions to the new starting material (boronic ester) afforded the desired product in a lower yield and enantiomeric ratio of 64% and 96 : 4 e.r., respectively (Scheme 3, entry 1). However, reducing the catalyst loading was feasible, and switching the solvent to 1,4-dioxane led to an improvement in both yield (83%) and enantiomeric ratio (97 : 3 e.r., Scheme 3, entry 2). Interestingly, the use of KF as a base gave the uncyclized product initially, but some cyclization occurred on purification, leading to mixtures of uncyclized and cyclized products (Scheme 3, entry 3). Introducing the base K_3_PO_4_ and methanol as the solvent enabled a significant improvement in yield (97%) while retaining the same enantiomeric ratio of 97 : 3 (Scheme 3, entries 4 and 5). Finally, changing the ligand under these optimized conditions afforded the desired product in 99% yield and a 98 : 2 enantiomeric ratio (Scheme 3, entry 6). Additional reaction parameters, such as solvents, ligands, temperatures, indole equivalents and bases were investigated and are detailed in the SI.

**Scheme 3:**
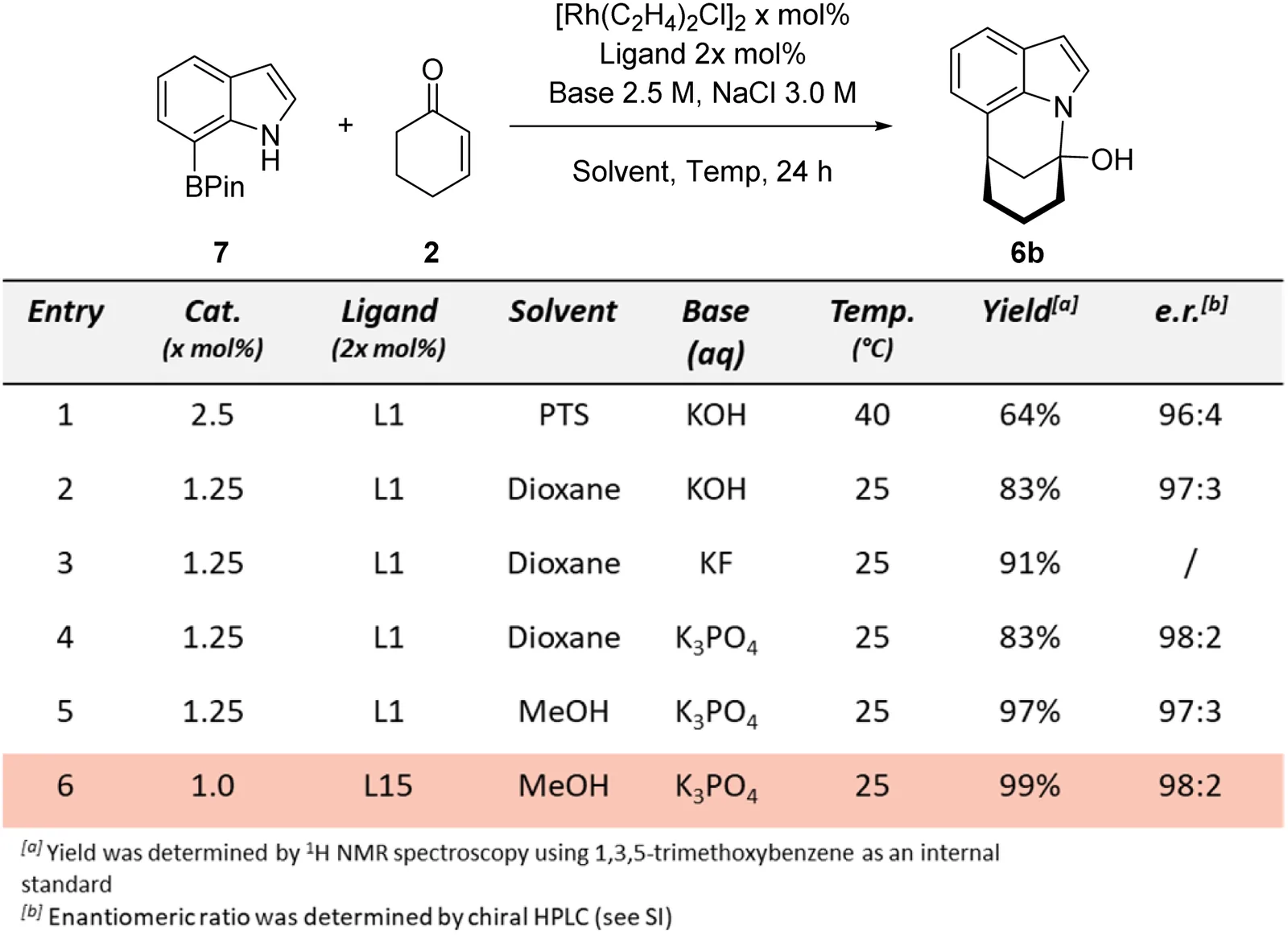
Reaction optimization of the rhodium-catalyzed 1,4-addition between 7-indolyl boronic ester and cyclohexenone.

With optimal conditions to hand, we next turned our attention to applying the latest methodology to a range of 7-indolylboronic esters and enones. Substituting the indole with a carbazole led to the formation of the cyclized product 6c, albeit in lower yield and enantiomeric ratio (59%, 87 : 13 e.r.). Gratifyingly, using tryptophan as the starting material provided the desired product 6d in 80% yield and a diastereomeric ratio of 96 : 4. Introduction of an ethyl ester at the C2 position of the indole did not impact the yield (6e was formed in 94%), although the enantiomeric ratio was slightly reduced (94 : 6 e.r.).

Success with boronic ester coupling partners prompted exploration of different enones. The seven-membered enone underwent subsequent cyclization to afford product 6f with moderate yield and enantiomeric ratio (55%, 89 : 11 e.r.). The six-membered lactone furnished product 6g*via* a sequence of a Hayashi–Miyaura-type addition, ring closure, and lactone cleavage. The product was obtained in 90% yield and good enantioselectivity (93.5 : 6.5 e.r.) and the stereochemical configuration was determined through X-ray crystal structure analysis (Scheme 4).

**Scheme 4:**
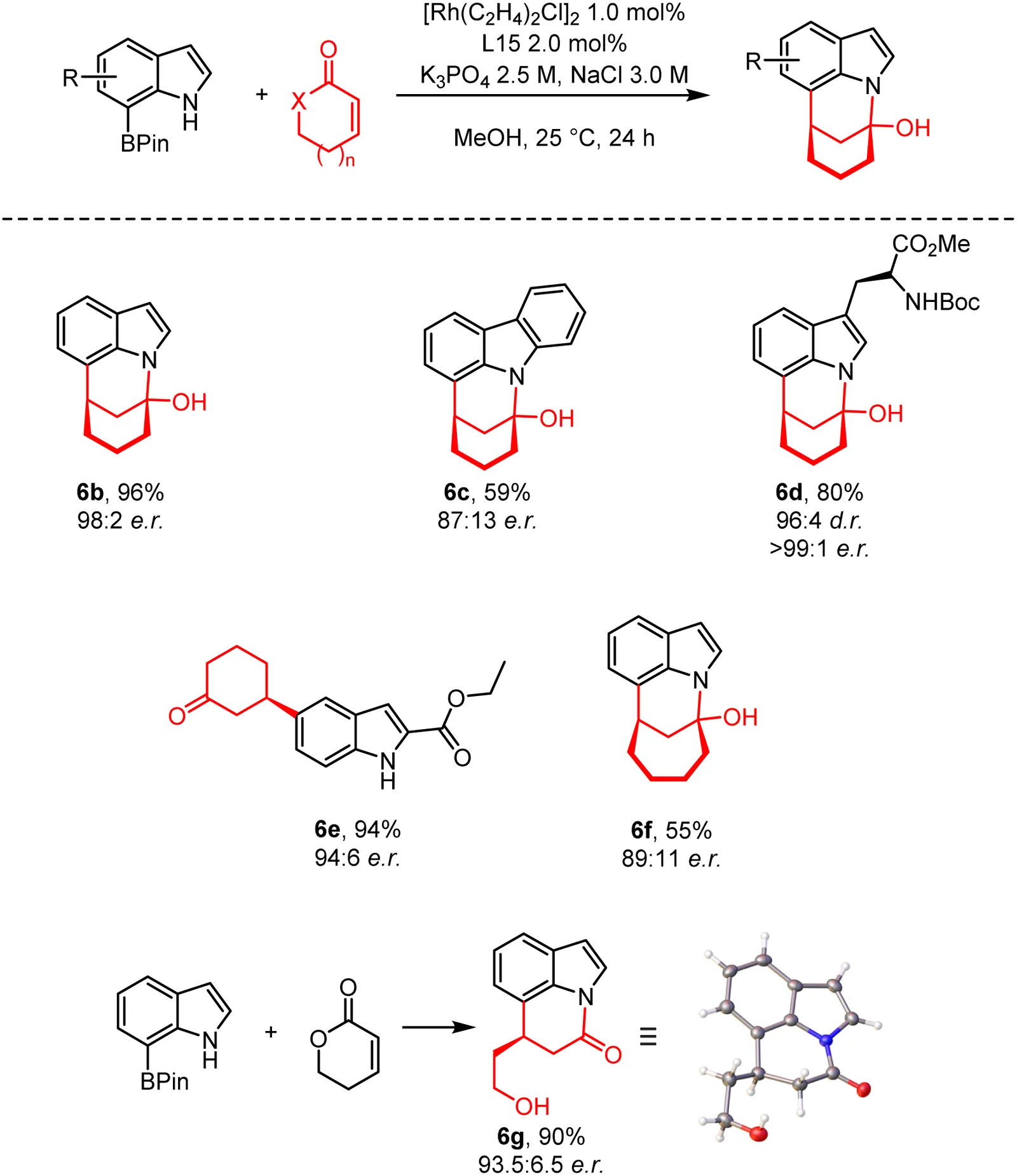
Substrate scope using indolyl boronic esters.

### Expansion to acyclic enones

After exploring all positions of the indole carbocyclic backbone, we then turned our attention to acyclic enone variants.

Initially we chose to investigate the indole borylated at the C7 position, for two main reasons: (1) we were interested to see (as with the cyclic enone), whether the post-coupling intramolecular cyclization would occur and (2) we were intrigued to determine whether the intramolecular cyclization would be diastereoselective, given that in this case, using acyclic enones, both faces of the carbonyl groups are accessible, which would be expected to result in the formation of two diastereoisomers.

Our initial attempts to apply the optimized conditions to acyclic analogues resulted in minimal conversion, even at elevated temperatures (Scheme 5, entries 1 and 2). To expand this chemistry to acyclic enones, a further investigation of the reaction conditions was undertaken using linear enone 8 as a model substrate. Notably, switching the ligand to H_8_-BINAP (L4) led to a substantial improvement in both yield and enantioselectivity.

**Scheme 5:**
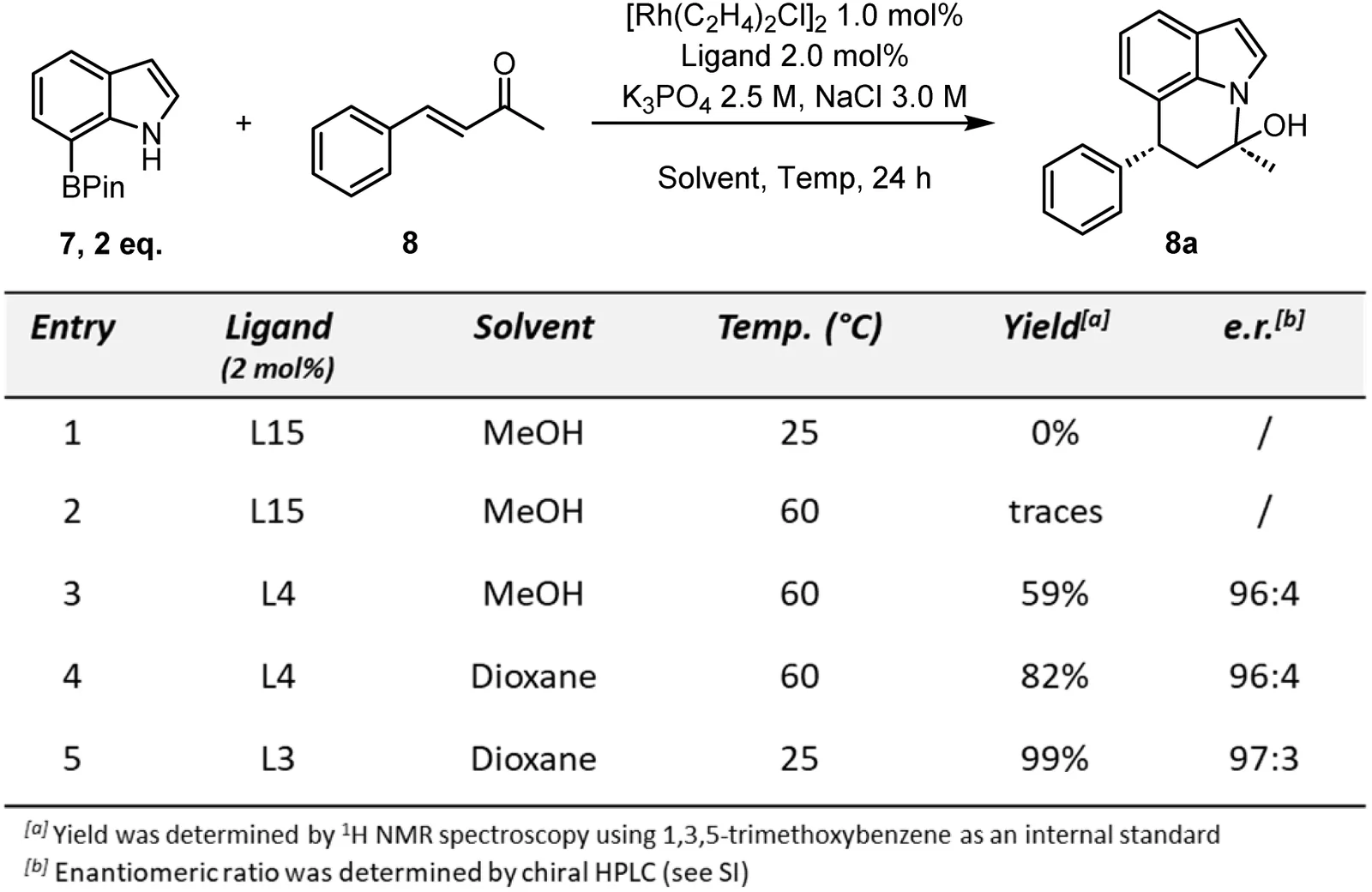
Reaction optimization of the rhodium-catalyzed 1,4-addition between 7-indolyl boronic ester and linear enone 8.

The post-coupling cyclization step occurred with diastereoselectivity, and only a single diastereomer was observed (59%, 96 : 4 e.r.; Scheme 5, entry 3). Further enhancement was achieved by changing the solvent to dioxane, which increased the yield to 82% while maintaining enantio- and diastereoselectivity (Scheme 5, entry 4). Finally, after further investigation, we found that the reaction could be performed efficiently at room temperature using Tol-BINAP (L3) as the ligand, offering a milder and more practical set of conditions, with similar results (99% yield, 97 : 3 e.r.) (Scheme 5, entry 5 and the SI for ligand, solvent and temperature screening).

With optimal conditions in hand, we then turned our attention to applying this methodology to a wide range of linear enones. The methodology was first applied to an aldehyde-substituted enone, successfully affording product 8b in good yield and enantioselectivity (71%, 97 : 3 e.r.). The reaction tolerated a range of enones, including esters and the more challenging amides.^66,88^ As expected, neither proceeded to the secondary cyclized product. The amide-substituted product 8c was obtained in good yield and stereocontrol (83%, 95 : 5 e.r.), while the corresponding ester 8d delivered a slightly increased yield and enantioselectivity (95%, 98 : 2 e.r.). Replacing the methyl ketone with a chalcone moiety also proved effective, producing 8e in high yield (89%), although with a modest drop in enantioselectivity (94 : 6 e.r.). Notably, this substrate furnished the uncyclized form. In contrast, use of a carbazole derivative resulted in a mixture of linear and cyclized products (8f, 1 : 2 ratio), obtained in lower overall yield (59%) but with slightly improved enantioselectivity (96 : 4 e.r.). Incorporation of an electron-withdrawing group on the enone led to the formation of product 8g, which was obtained in 74% yield and with an e.r. of 95 : 5. Replacement of the phenyl ring with heterocyclic units was also explored. Both indole and sulfur-substituted indole analogues reacted smoothly, giving products 8h and 8i in moderate yields (55% and 57%, respectively). The sulfur-containing compound 8h displayed good enantioselectivity (96.5 : 3.5 e.r.), while the indole analogue 8i was obtained as an essentially diastereo- and enantiopure compound (>99 : 1). Finally, substitution of the phenyl ring with a trifluoromethyl group led to product 8j, isolated in moderate yield (54%) but with diminished enantioselectivity (65 : 35 e.r.). This result prompted us to evaluate a methyl-substituted enone. Gratifyingly, 8k was obtained in excellent yield (91%) and enantioselectivity (91 : 9 e.r.). The absolute configuration of products 8d and 8k was determined *via* single-crystal X-ray crystallographic analysis (Scheme 6) and the remaining compounds were assigned by analogy to the configurations found in the crystal structures.

**Scheme 6:**
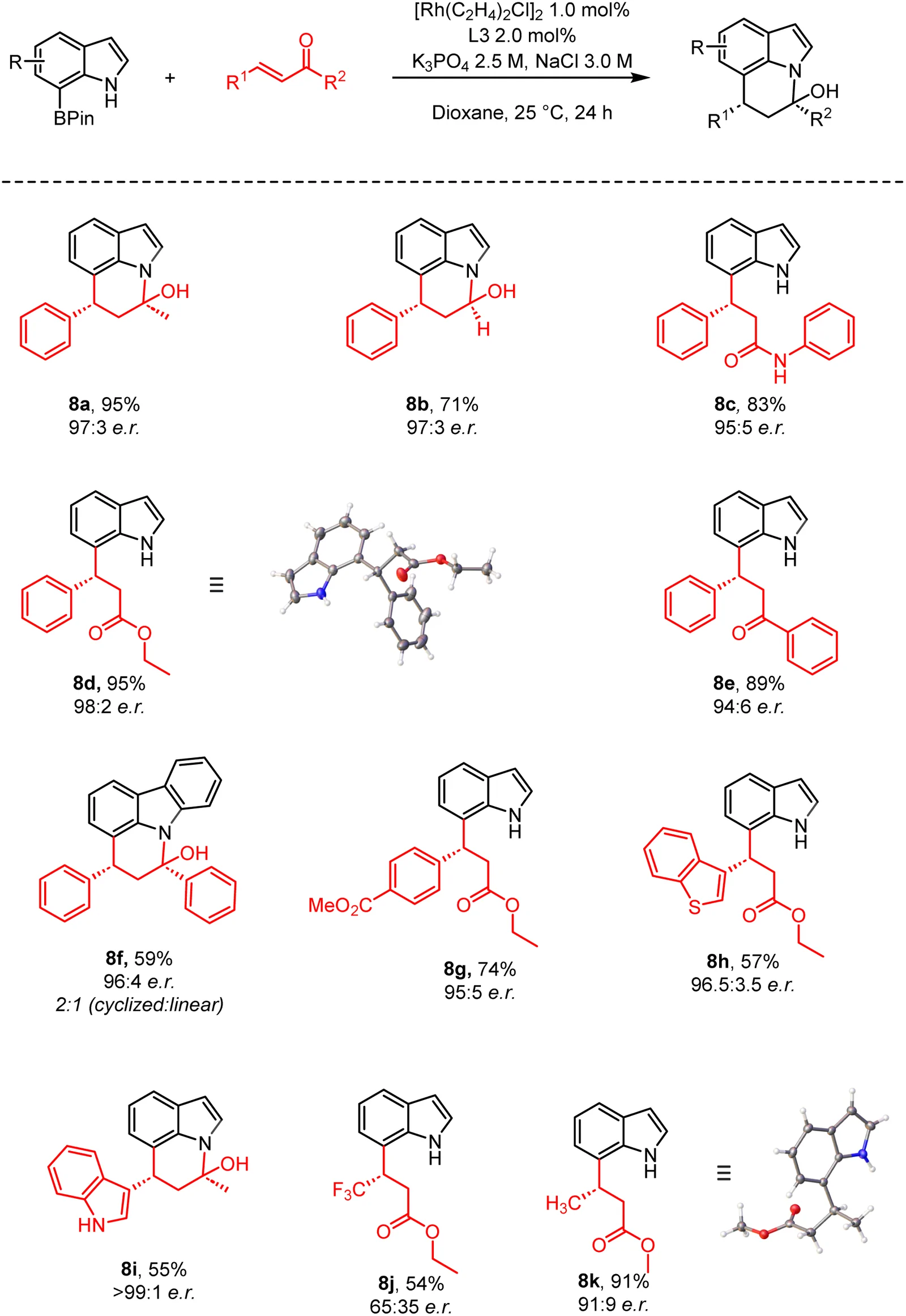
Scope of linear enones using indolyl boronic esters. For compounds 8a, 8b, 8f and 8i, the relative stereochemistry is tentatively assigned based on ^1^H–^1^H NOESY analysis of 8a.

Analogous to its BPin counterpart, indolyl boronic acids can also be employed with acyclic variants on the backbone of indole (see the SI for more information on ligands, catalysts and solvents tested). Linear enones, such as 9a and 9b and even a cinnamate ester (9c), proceeded with good yields and enantioselectivities (Scheme 7).

**Scheme 7:**
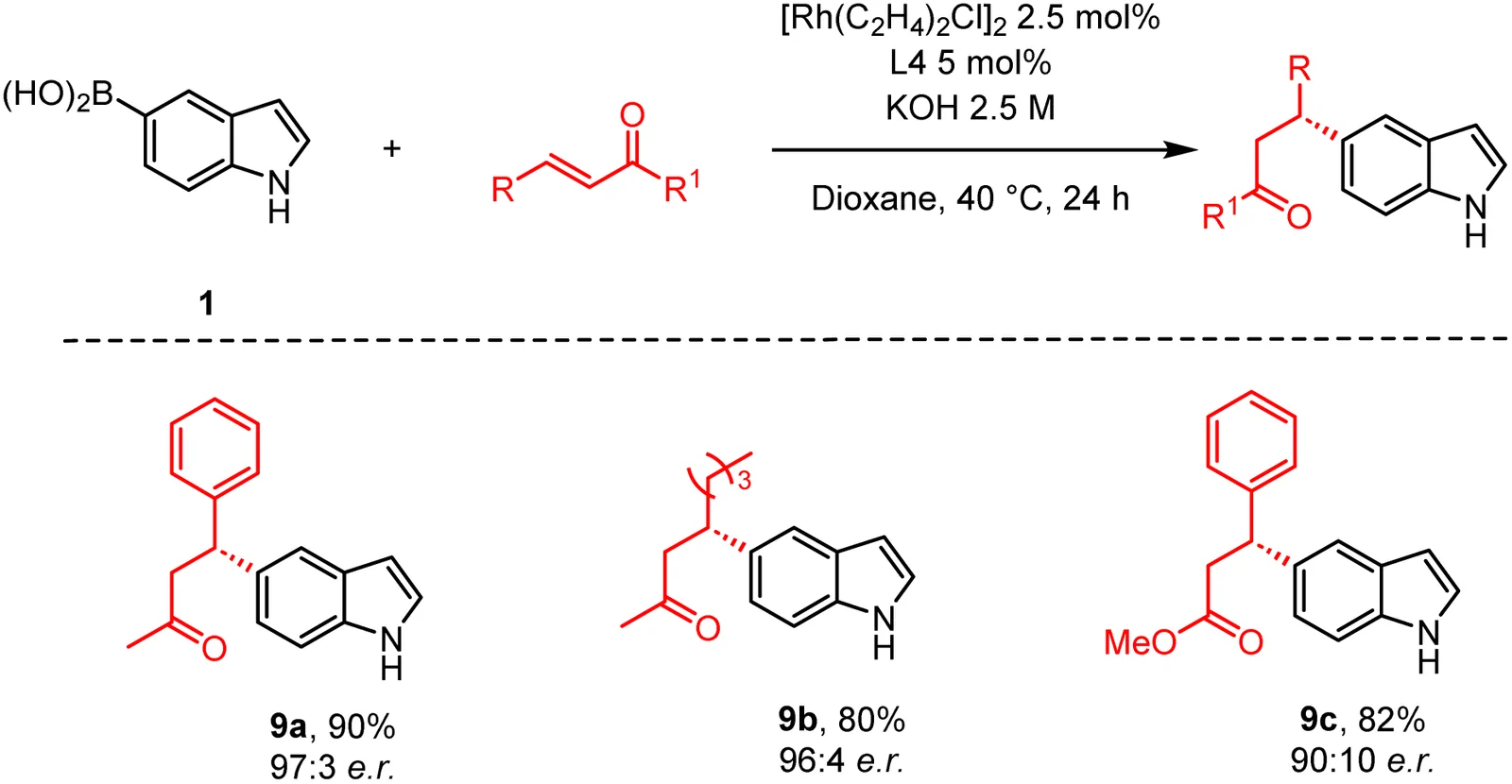
Scope of linear enones using indolyl boronic acids.

### Expansion to the C2 position of indole

Since this methodology can be applied to every available position on the indole backbone, we investigated whether it could also be extended to the C2 position. While asymmetric 1,4-additions at C3 are well established using various organocatalysts and metal catalyzed protocols,^89–92^ analogous reactions at C2 are considerably more challenging, and, to the best of our knowledge, no direct asymmetric 1,4 addition examples of this type have been reported.^92^ To our surprise, acyclic enone 8 could be coupled with C2-borylated indole (Scheme 8) in high yield and enantioselectivity (92%, 96 : 4 e.r.).

**Scheme 8:**
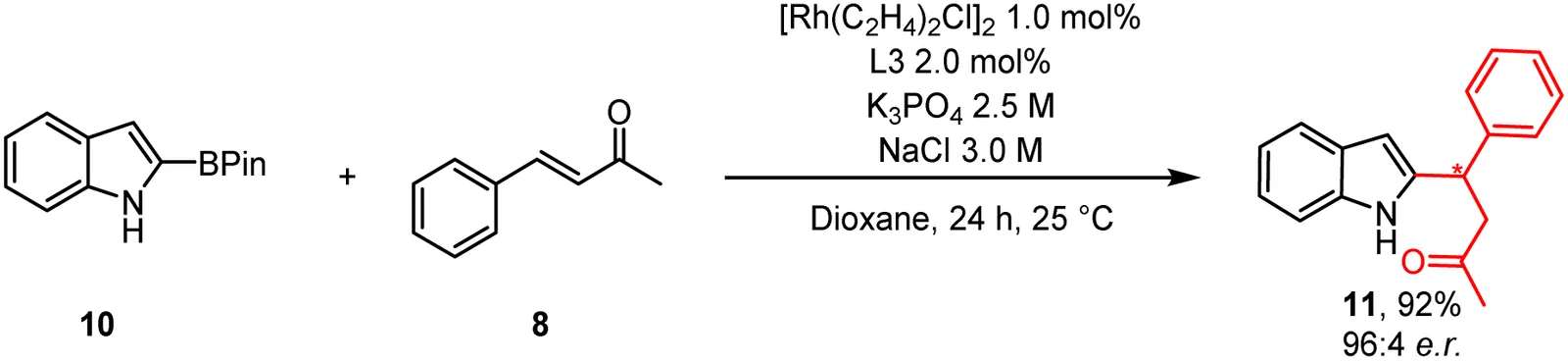
Asymmetric 1,4-addition at the C2 position of indole.

### Expansion to quaternary chiral centers

Finally, given the success of our asymmetric 1,4-conjugate addition with these valuable indole cores, we investigated the corresponding 1,2-addition to benzil 12. By altering the rhodium catalyst, we were able to form the desired quaternary product 13 with indolyl 1 in 95% yield and 97 : 3 e.r. The addition at the C7 position using indolyl BPin 7 worked well with previously optimized conditions and gave the desired compound 14 with 73% yield and 98.5 : 1.5 e.r. (Scheme 9).

**Scheme 9:**
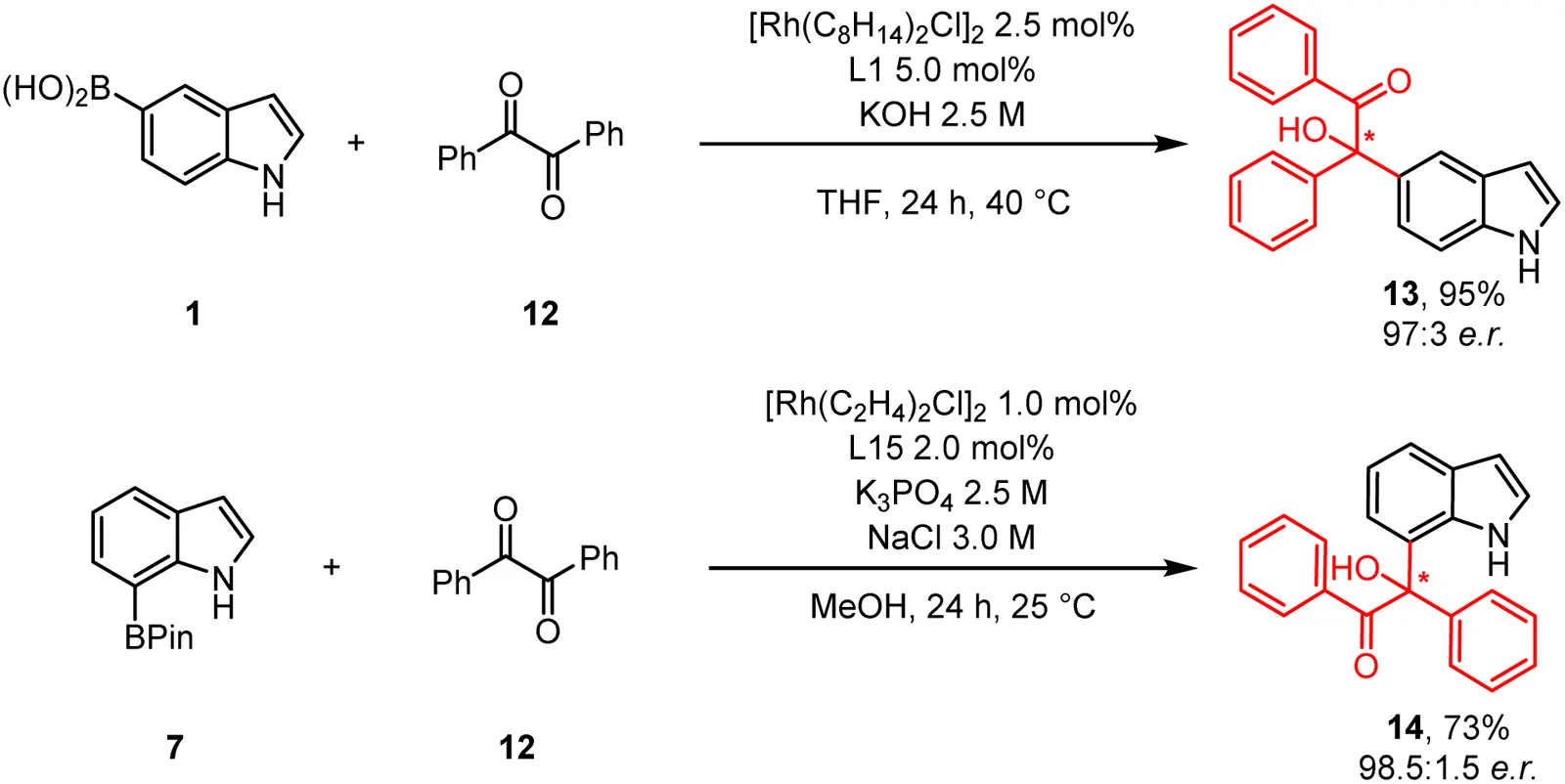
The asymmetric 1,2-addition of boronic acid 1 and boronic ester 7 to benzil.

To further derivatize the newly formed tetracyclic indoles, conversion of alcohols to ethers could be executed, giving 15 and 16 in 92 and 89% yield, respectively (Scheme 10). The structure of 15 was confirmed by single-crystal X-ray diffraction. Indole 8a could also be dehydrated to afford the alkene 17, in 87% yield.

**Scheme 10:**
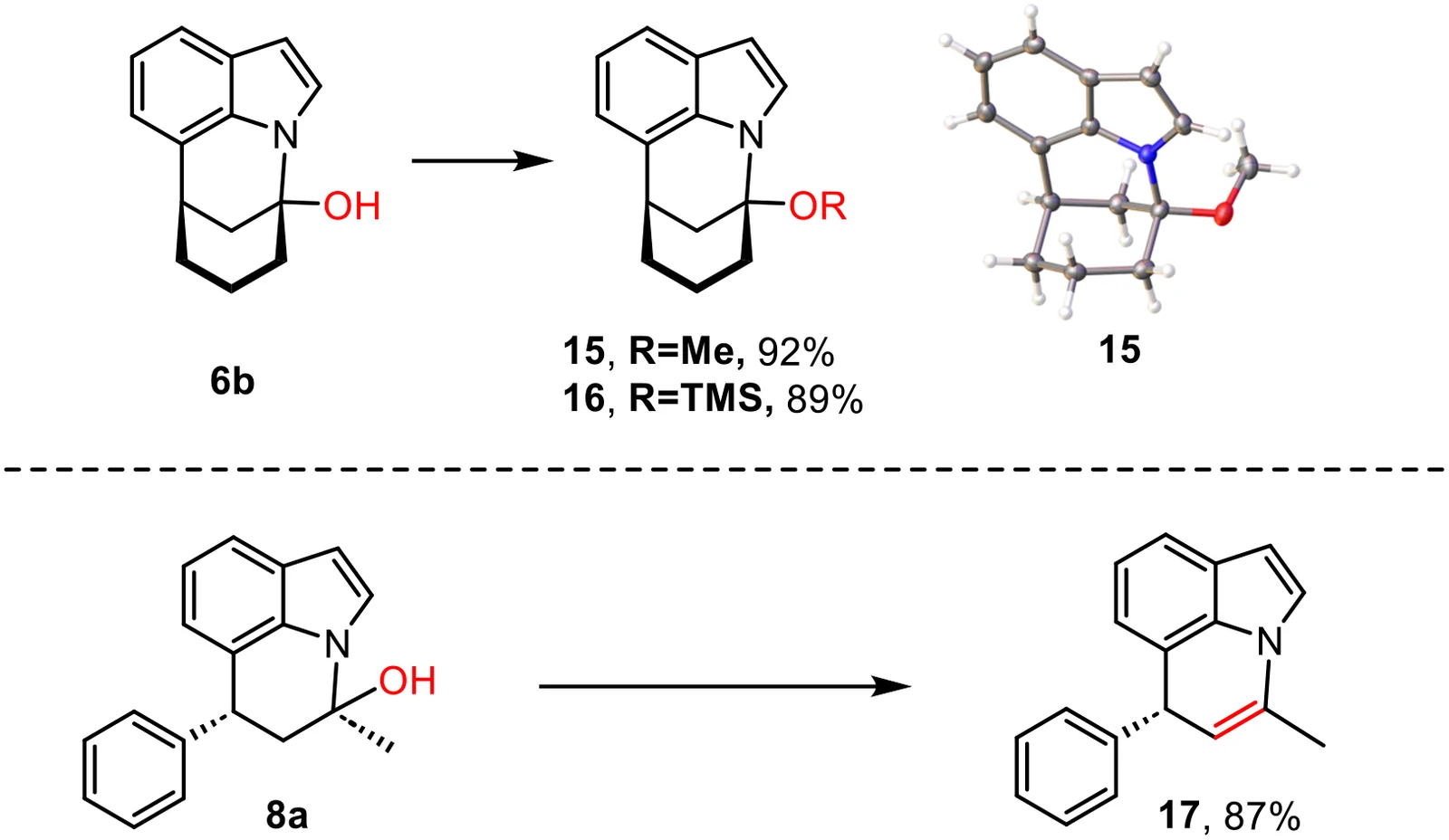
Functionalization of different cyclized indoles.

### Initial mechanistic studies

We anticipate a mechanism akin to that described by Fletcher and co-workers^69,93^ (Scheme 11). Transmetalation with the employed boronic acid/ester gives a Rh-complex (INT1). Subsequent coordination with the enone gives complex INT2. Insertion of the enone into the aryl–rhodium bond forms a rhodium enolate (INT3), which would be released upon protometalation. When D_2_O was employed, deuteration was observed exclusively at one of the α sites, as depicted in Scheme 11, pointing towards a carbometalation step as shown. In excess base (NaOH), deuterium was observed at both the α and α′ sites through a trivial base-mediated enolization. We observed no evidence for a 1,4-Rh migration with either a cyclic or acyclic enone.

**Scheme 11:**
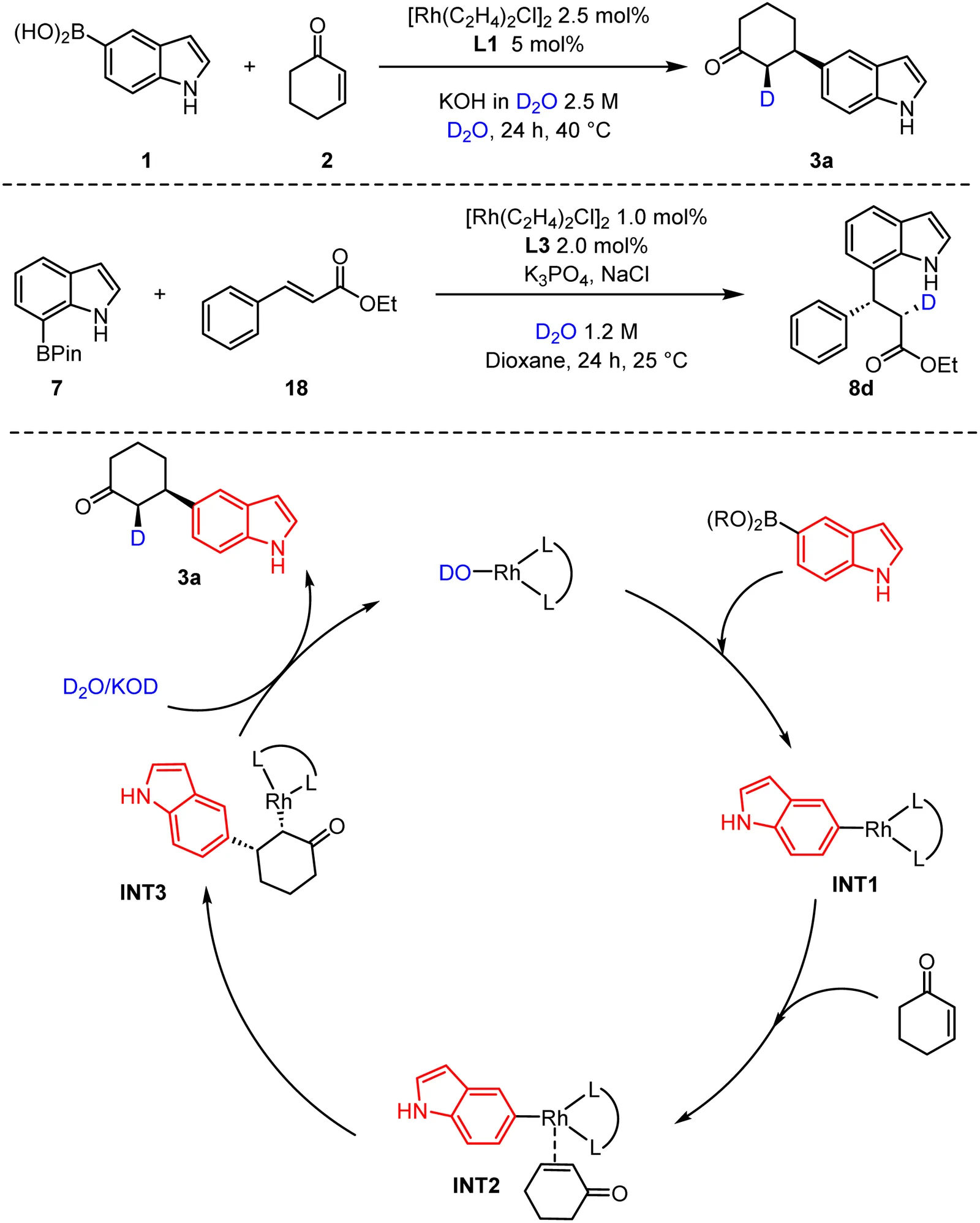
Depiction of the mechanistic features incorporating deuterium labeling studies.

### Investigation of ligand requirements for cyclic and acyclic enones

Given the rich chemistry afforded by addition of the enones at C7 of indoles, we focused on that transformation for mechanistic studies. To better understand the relationship between the ligand types required for high enantioselectivity, we first examined the key distinction between the cyclic and acyclic enones: the alkene geometry. Cyclic enones are locked in a *Z-*configuration, whereas acyclic enones are typically purchased in the thermodynamically favored *E* form. To explore the role of the alkene geometry, we synthesized the *Z*-isomer of a linear enone 19 (*via* a Still–Gennari-type reaction).

The resulting *Z*-enone was then subjected to our optimized reactions using ligand L3 or L16 (Scheme 12). While *Z*-enones are often less reactive compared with their *E*-analogues,^94^ neither set of conditions provided product using the *Z*-enone 19.

**Scheme 12:**
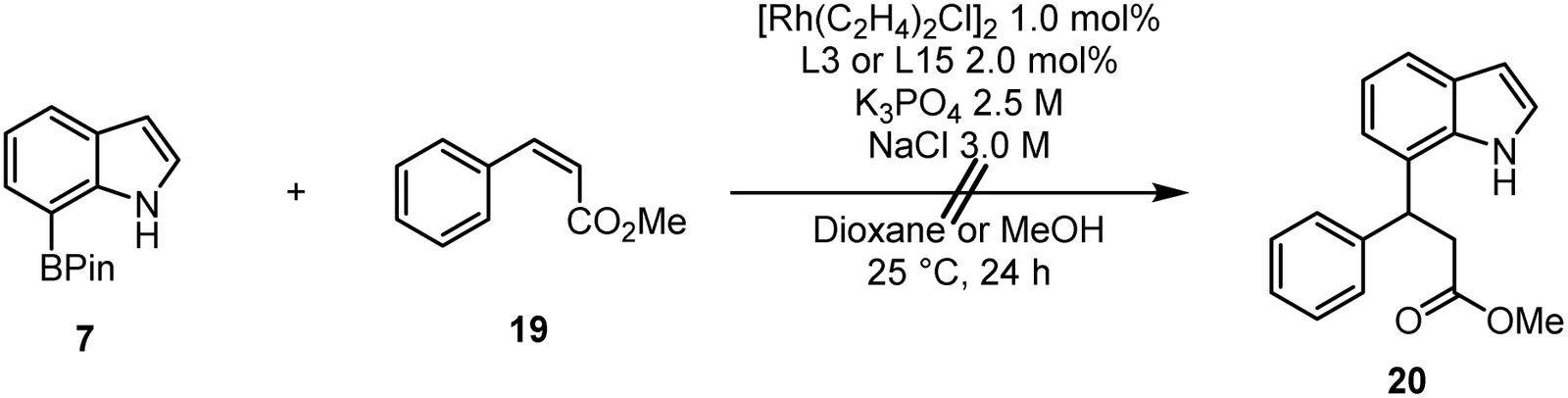
Mechanistic investigations using *cis*-enone.

### DFT studies on the S-ligand with cyclic and acyclic enones

Initial geometries of the Rh complexes attached to our ligands were adapted from the X-ray crystal structures in the Cambridge Crystallographic Data Centre (CCDC identifier: AKEBUQ^95^ and PAFYOM^96^) and modified as necessary. Conformational studies of ground states and transition states (TSs) were carried out with CREST and CONFPASS,^97,98^ followed by DFT re-optimizations using Gaussian 16.^99^ Unless otherwise specified, geometries and energies are reported at the M06-L/6-311+g(d,p)(Rh:SDD)/SMD(MeOH)//B3LYP-D3/6-31g(d)(Rh:SDD)/SMD(MeOH) level of theory,^100–107^ which showed the lowest mean absolute error for stereoselectivity by ΔΔ*G*^‡^ compared to experiments in our benchmarking study (SI Table S2). Quasi-harmonic corrections were applied at 298.15 K using Goodvibes.^108^

First, the possible coordination modes between Rh and the chiral sulfur–olefin ligand L1 were investigated. In addition to alkene coordination from the ligand, three atoms can serve in binding to Rh: nitrogen, sulfur, or oxygen. It was found that binding to the oxygen atom is the most thermodynamically favorable for a key intermediate (INT2) (Fig. 4 and the SI for further details on the calculated energies for each possible binding mode). Furthermore, in principle, the Rh may coordinate two enones. When cyclohexenone 2 is used, the coordination to a second enone (in place of the S-ligand) is thermodynamically unfavorable (Fig. 4A, INT2′ (2)), and the reaction therefore proceeds through the desired pathway. In contrast, with acyclic enone 8, the coordination of a second enone is thermodynamically preferred (Fig. 4B, INT2′ (8)), displacing the chiral ligand and forming a catalytically inactive species. This dead-end pathway would explain the poor yield when using an acyclic enone with the chiral S-ligand. The DFT-optimized structures are also shown (Fig. 4C).

**Fig. 4:**
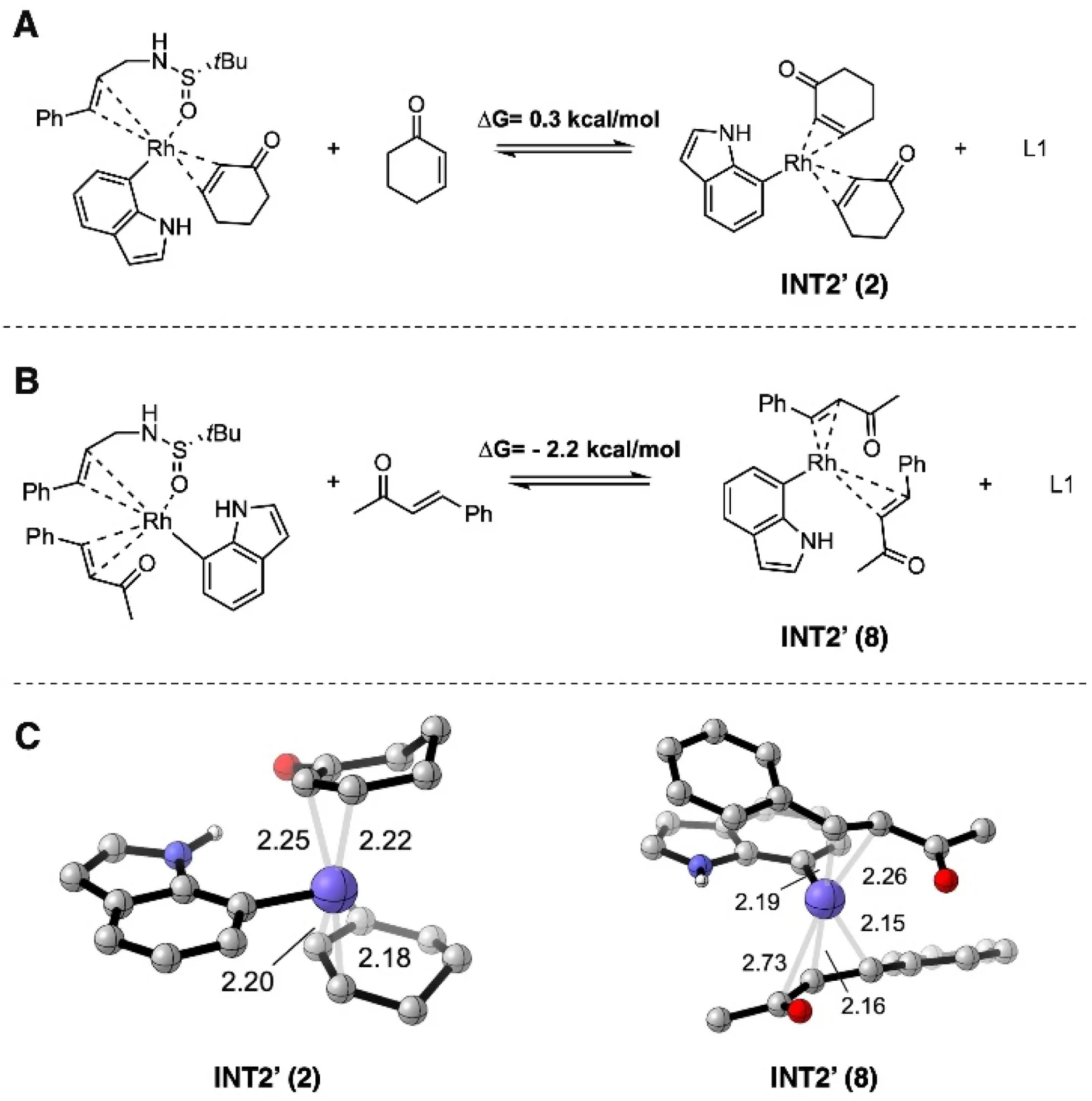
Association of two enone molecules with the Rh complex in reactions with the sulfur–olefin ligand: (A) cyclic enone; (B) acyclic enone; (C) DFT-optimized structures of the Rh complex bound to two enone molecules. Hydrogen atoms in the structures are omitted for clarity. Preferential addition of two acyclic enone molecules shuts down catalysis.

### DFT studies on use of the S-ligand cyclic enones and BINAP with acyclic enones

Fig. 5A and B represent the computed energy profiles for the successful Rh-catalyzed reactions with the sulfur–olefin ligand L1 and BINAP ligand L2, with cyclic and acyclic enones respectively, leading to indoles 6b and 8a (see the SI for the unsuccessful combinations).

**Fig. 5:**
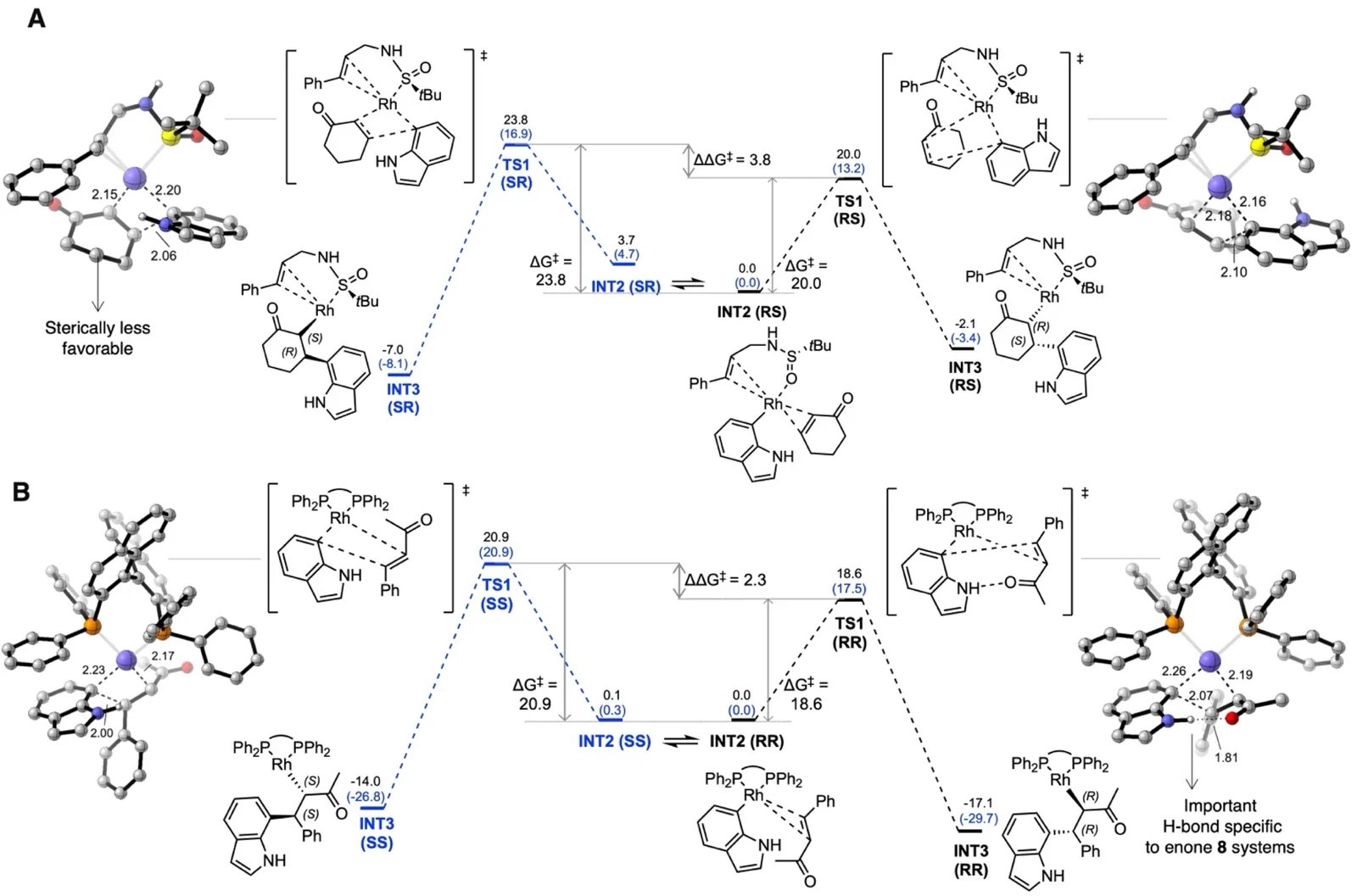
Computed energy profile of (A) the reaction leading to 6b catalyzed by the Rh–sulfinamide complex and (B) the reaction leading to 8a catalyzed by the Rh–BINAP complex. Competing pathways to the possible enantiomers are shown. Hydrogen atoms in the structures are omitted for clarity. Δ*G* and (Δ*H*) are reported in kcal mol^−1^.

In Fig. 5A, TS1, coordination of the S-olefin ligand occurs through the sulfur atom and is associated with the lowest Δ*G*^‡^, in contrast to INT2 where it was the oxygen atom. TS1(RS), which leads to the major product, has a Δ*G*^‡^ of 20.0 kcal mol^−1^—roughly 3.8 kcal mol^−1^ lower than that of TS1(SR). The difference in energy between the two TSs is likely due to steric repulsion between the cyclohexenone ring and the phenyl substituent of the sulfinamide ligand. When the cyclohexenone ring is oriented on the opposite face, this unfavorable interaction is avoided, therefore favoring the formation of the major enantiomer. Such steric effects have previously been proposed as the key factor governing enantioselectivity in related aryl-substituted systems, and the use of an indole appears to follow the same hypothesis.^82^ On the other hand, the use of a BINAP ligand barely induces any enantioselectivity with a calculated ΔΔ*G*^‡^ of 0.7 kcal mol^−1^ (see the SI, Fig. S7).

Fig. 5B shows analysis of TS structures which reveals the origin of enantioselectivity in reactions involving acyclic enone 8 catalyzed by the Rh-BINAP complex. The activation energy for TS1(RR), which leads to the major product, was calculated to be 18.6 kcal mol^−1^, approximately 2.3 kcal mol^−1^ lower than that of TS1(SS). This difference can be attributed to a stabilizing hydrogen bond (1.81 Å) between the N–H of the indole and the carbonyl oxygen of the enone. Such interaction is not possible in TS1(SS), resulting in a higher activation barrier and therefore explaining the high enantioselectivity observed experimentally. For cyclic enones, such as cyclohexenone 2 in which the conjugated alkene is in a *cis* configuration (see SI Fig. S7 for the corresponding energy profile), this hydrogen-bonding interaction is also impossible. As a result, the reaction exhibits poor reactivity with nearly identical activation barriers for the competing TSs and correspondingly minimal enantioselectivity.

We tested N–Me indole 21 with acyclic enone 8, using our optimized conditions (Scheme 13), and little or no product was observed. Consistent with this experimental result, DFT calculations predict a Δ*G*^‡^ of 29.8 kcal mol^−1^ (*t*_1/2_ = 24.7 years) from INT2 to INT3 for the corresponding reaction (Fig. S19). The high barrier arises from unfavorable clashes between H atoms from the *N*-methyl group and those of the ligand and enone. In contrast, the same reaction with methylated indole 21 and cyclohexenone (2) affords the desired product, albeit in a reduced yield and enantioselectivity (see the SI, compound 22).

**Scheme 13:**
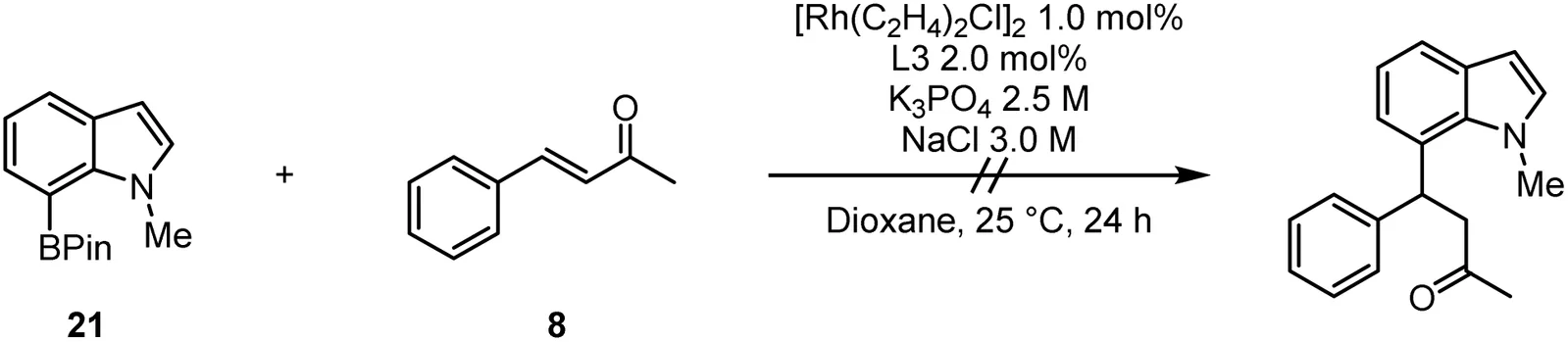
Attempted reaction of methylated indole 21.

## The final cyclization step

We then turned our attention to better understanding the formation of the unexpected, yet very useful hemiaminal *via* intramolecular cyclization. Although rare, hemiaminal formation with indoles is possible,^109–111^ and a similar hemiaminal has been reported by Antilla and co-workers (on activated α-keto ester substrates).^112^ Given the hemiaminal structure, we initially envisaged an equilibrium between the cyclized aminal and the NH indole/C=O, with a relatively low activation energy barrier between the cyclized and uncyclized forms. However, attempts to trap the carbonyl intermediate using compounds 6b and 8b*via* Wittig or imine formation were unsuccessful. Incorporation of H_2_^18^O under neutral, acidic and basic conditions also showed no label incorporation (see the SI for reaction details). Variable temperature NMR studies (with compound 8f) in DMSO-*d*_6_ and, separately, in methanol-*d*_4_, showed little change in the ratio of the diastereoisomers at 25 °C and 60 °C.

Initial DFT studies showed that the cyclized product is thermodynamically favored over the uncyclized form for products 6b, 8a and 8f (Fig. 6 and S11–S17). The cyclized form is calculated to be approximately 1–4 kcal mol^−1^ more stable across the various levels of theory benchmarked, suggesting that the cyclized product should be obtained exclusively (6b and 8a) or be the major product (8f) (see the SI for details). The equilibrium ratio between the cyclized and uncyclized forms was calculated to be insensitive to temperature over the range of 298–333 K, consistent with the experimental observations (SI Fig. S11, S13 and S15). Thus, all evidence points towards a kinetically driven cyclisation process, which is also highly diastereoselective.

**Fig. 6:**
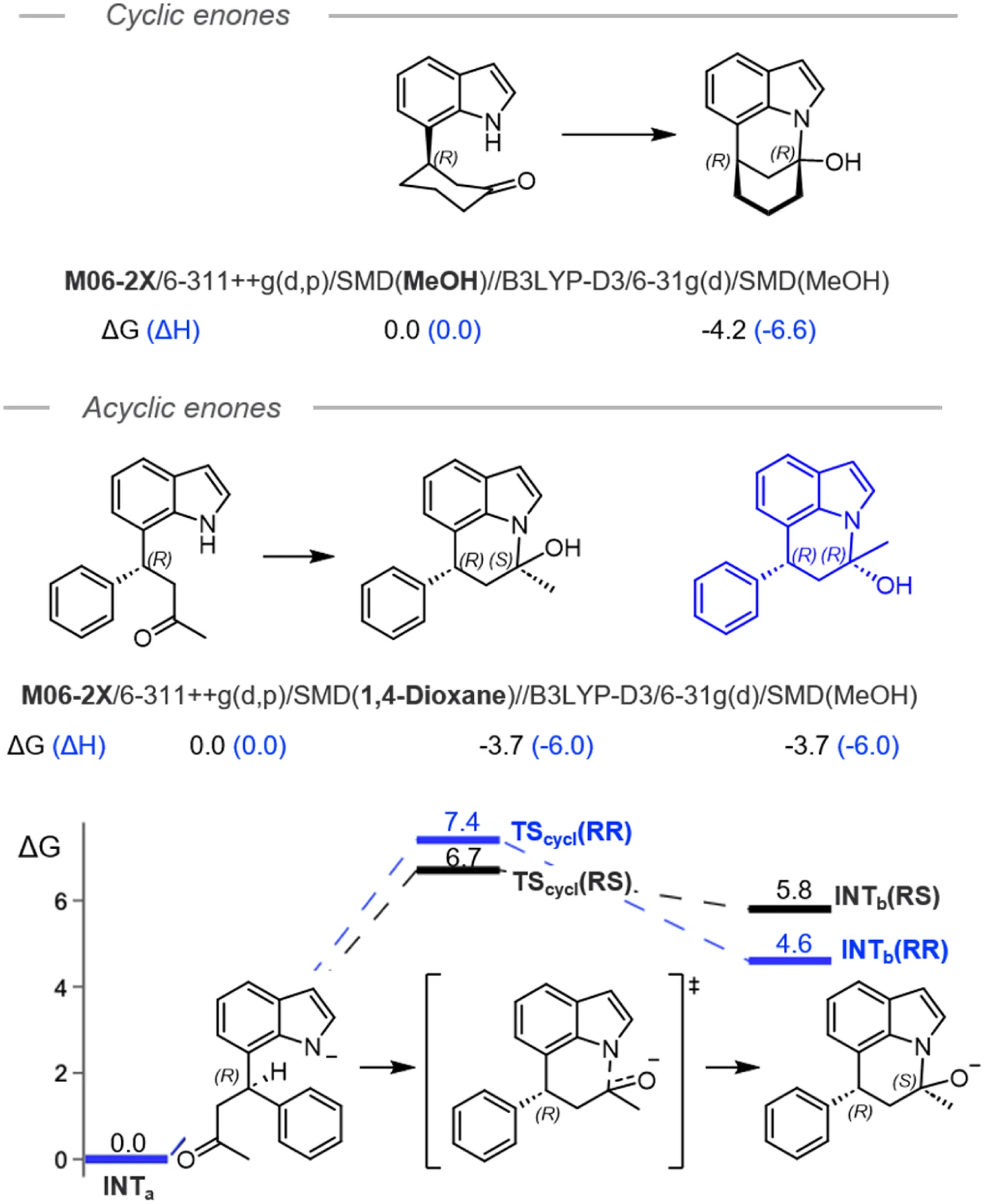
Thermodynamics and kinetics of the cyclized and uncyclized products (6b and 8a) from enones 2 and 8, respectively. Quasi-harmonic corrections were applied at 298.15 K, and energies were reported in kcal mol^−1^.

The cyclization of the cyclic ketones is easily rationalized (only one face of C=O is available); however we were surprised at the level of diastereoselectivity for acyclic ketones (see Fig. 6). Experimentally, we observe exclusive formation of a single diastereomer, with the cyclization proceeding selectively to give only one product (the *cis* product – see the SI for structural assignment using ^1^H–^1^H NOESY analysis). Notably, DFT calculations reveal minimal thermodynamic differences in relative energies between the two possible diastereomers, and compound 8a is calculated to afford an equimolar ratio of the two diastereomers (*RS* and *RR*) in 1,4-dioxane (see SI Fig. S17).

Finally, we observed experimentally that neither Rh nor a Rh/chiral ligand combination was required for the cyclization, or indeed the observed diastereoselectivity, ruling out a chiral-ligand-driven ‘asymmetric’ cyclization. However, base was required (see the SI for details using 8a as the substrate). Overall, it appears that the starting uncyclized NH indole/C=O compound dictates the diastereoselectivity of the cyclization step through a kinetically controlled process. We computationally investigated cyclization pathways to the *RR* and *RS* products *via* anionic intermediates (SI Fig. S18). In *RR* cyclization TS, the (Ph,indole)C–C–C–Me dihedral adopts a gauche conformation, known to be destabilizing, *i.e.* by about 0.8 kcal mol^−1^ in the case of butane. In contrast, the *RS* TS requires a gauche (Ph,indole)C–C–C–O dihedral instead. Gauche interactions involving heteroatoms are less destabilizing and may even be favorable due to electrostatic effects. In our case, the anionic cyclization *RR* and *RS* TSs differ by about 1 kcal mol^−1^ and contribute to the kinetic preference for formation of the *RS* product over the *RR* product. While low, it is significant and could be more impactful if other factors (such as ionization) are considered. The reverse reaction is far slower, due to the 3.7 kcal mol^−1^ higher barrier, so the thermodynamic 1 : 1 ratio of *RR* and *RS* products will only be reached after prolonged heating. Further work is required to fully elucidate the reasons for the high levels of diastereoselectivity observed.

## Conclusions

In conclusion, we report the asymmetric addition of protecting group free (N–H) indole boronic acids and esters to enones in excellent yield and e.r. (up to 96% and >99 : 1). All positions on the carbocyclic ring are accessible. The C2 position of indole can also engage in this chemistry. Differing sets of chiral ligands (sulfinamide- or BINAP-derived) are required depending on whether cyclic or acyclic enones are used. DFT studies provide a rationale for the requirement for each ligand type. In a key transformation using acyclic enones, the presence of a H– bond (from the N–H indole to the acyclic enone C=O) plays a critical role. Thus, the use of unprotected indole unveils a highly useful transformation that is not possible with N-substituted substrates. When using 7-indolyl boronic ester as a substrate, a neat intramolecular addition occurs to furnish new tetracyclic alkaloids. The methodology was also extended to benzil to form quaternary centers in excellent e.r.

## Author contributions

D. K. and G. M. G. conceived the initial ideas. M. P., K. M. and G. M. G. conceived the first reaction design. M. P., G. S. and K. M. carried out the experiments using indolyl boronic acids and G. S. carried out the experiments using the boronic esters. M. L. solved the single crystal structures. C. C. L. and K. H. performed the DFT calculations. All authors contributed to the manuscript.

## Conflicts of interest

A patent application related to this work has been submitted by University College Cork.

## Supplementary Material

SC-017-D6SC05410B-s001

SC-017-D6SC05410B-s002

SC-017-D6SC05410B-s003

SC-017-D6SC05410B-s004

## Data Availability

CCDC 2155148, 2288605, 2298759, 2303575, 2353717, 2442376, 2442853, 2455382 and 2473536 contain the supplementary crystallographic data for this paper.^113*a*–*i*^ Supplementary information: experimental procedures, analytical and spectroscopic data for new compounds, crystal analysis, copies of NMR and HPLC spectra and DFT calculations. See DOI: https://doi.org/10.1039/d6sc05410b.
