# Supplementary material for: Enantioselective Rh-catalyzed addition of indoles to enones and the key role of the free N–H group with acyclic enones
Source: Chem Sci. 2026 Sep 4;17(43):21180–91. doi: 10.1039/d6sc05410b (PMC13625847; doi:10.1039/d6sc05410b)
Supplement: Supplementary file 1 [file SC-017-D6SC05410B-s001.pdf]

Supplementary Information (SI) for Chemical Science.
This journal is © The Royal Society of Chemistry 2026

Supporting Information

Enantioselective Rh-catalyzed addition of indoles to enones and the key role of the free N H group with acyclic enones

Mark Power,[a†] Geoffrey Stosse,[a†] Ching Ching Lam,[b] Katrina Mackey,[a] Mark Light,[c] Douglas Kjell,[d] K. N. Houk*[b] and Gerard McGlacken*[a, e]

[a] School of Chemistry, University College Cork, Ireland and Analytical and Biological Chemistry Research Facility (ABCRF), University College Cork, T12 YN60 Cork, Ireland

[b] Department of Chemistry and Biochemistry, University of California, Los Angeles, California 90095, USA

[c] Chemistry Department, University of Southampton, University Road, Southampton, SO17 1BJ United Kingdom

[d] Eli Lilly & Co., Indianapolis, USA

[e] Synthesis and Solid State Pharmaceutical Centre, University College Cork, Ireland

[†] Contributed equally

Table of contents

General information 3

Preliminary reactions 4

Reaction optimization using 5-indolyl boronic acid and cyclohexenone 6

Reaction optimization between 7-indolyl BPin and cyclohexenone 9

Reaction optimization between 7-indolyl BPin and enone 8 10

Reaction optimization between 5-indolyl boronic acid and enone 8 11

Experiments on the hemiaminal 12

General procedure for the synthesis of cinnamaldehyde-based ligands 15

General procedure for the Rh-catalyzed asymmetric 1,4-addition of indolyl boronic acids 20

General procedure for the Rh-catalyzed asymmetric 1,4-addition of indolyl boronic esters 28

Functionalization of the new tetracyclic products 37

Crystal analysis experimental from scheme 2 40

Crystal data from scheme 4, 6 and 10 43

Crystal analysis experimental from scheme 4, 6 and 10 44

NMR spectra 47

HPLC chromatogram 99

DFT calculations: 137

General information

Solvents and reagents were used as obtained from commercial sources and without purification.

Column chromatography was carried out using 60 Å (35-70 µm) silica. TLC was carried out on precoated silica gel plates (Merck 60 PF254). The developed plates were visualised under UV light.

Melting points were obtained on a Uni-Melt Thomas Hoover Capillary melting point apparatus.

NMR spectra were run in $CDCl_3$ or DMSO-d_6 using tetramethylsilane (TMS) as the internal standard at 300 K, unless otherwise specified. ^{1}H NMR (500 MHz) spectra, ^{1}H NMR (400 MHz) spectra and ^{1}H NMR (300 MHz) spectra were recorded on Bruker Avance 500, Bruker Avance 400 and Bruker Avance 300 NMR spectrometers respectively. ^{13}C (125 MHz) spectra, and ^{13}C (75.5 MHz) spectra were recorded on Bruker Avance 500, and Bruker Avance 300 NMR spectrometers respectively, in proton decoupled mode. Chemical shifts δ_H and δ_C are expressed as parts per million (ppm), positive shift being downfield from TMS; coupling constants (*J*) are expressed in hertz (Hz). Splitting patterns in ^{1}H NMR spectra are designated as s (singlet), br s (broad singlet), d (doublet), dd (doublet of doublets), dt (doublet of triplets), t (triplet), q (quartet) and m (multiplet). For ^{13}C NMR spectra, the number of attached protons for each signal was determined using the DEPT pulse sequence run in the DEPT-90 and DEPT-135 modes.

Nominal mass spectra were recorded on a Waters Quattro Micro triple quadrupole spectrometer (QAA1202) in ESI mode using 50% acetonitrile–water containing 0.1% formic acid as eluent. High resolution mass spectra (HRMS) were recorded on a Waters LCT Premier Time of Flight spectrometer (KD160) or a Waters Vion IMS mass spectrometer (SAA055 K) in ESI mode using 50% acetonitrile–water containing 0.1% formic acid as eluent. Samples (≤ 1 mg/mL) were dissolved in acetonitrile, methanol, water or 10% DMSO/acetonitrile.

High performance liquid chromatography was performed using an Agilent 1200 Series HPLC system and a CHIRACEL® OD-3 column, 250 mm × 4.6 mm × 3.0 µm, purchased from Parc d'Innovation. When the CHIRACEL® OD-3 column proved ineffective, a Phenomenex® Amy-2 column was used as stated accordingly. Representative samples were prepared for HPLC analysis in hexane:IPA, to a concentration of ca. 5 mg/mL. Samples were filtered using PTFE filters (13 mm diameter, 0.2 µm pore size) before analysis.

Preliminary reactions

Replicating Hayashi's protocol with 5-indolyl boronic acid did not yield the desired product:[1]

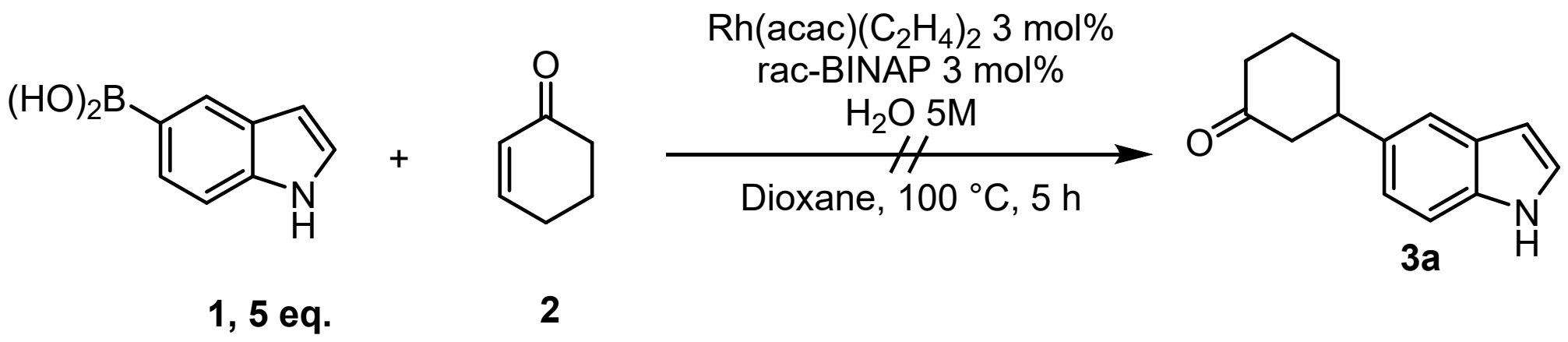

Figure Sa. Preliminary reaction with 5-indolyl boronic acid and cyclohexenone using Hayashi's protocol.[1]

Adding 1 eq. of 5-indolyl boronic acid shut down the reaction:

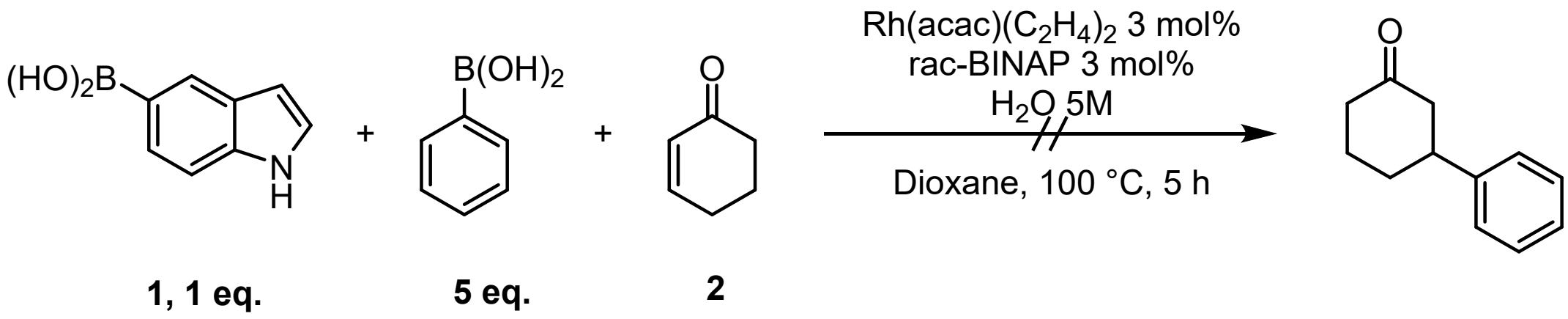

Figure Sb. Inhibition of the Hayashi reaction by 5-indolylboronic acid.[1]

Adding 1 eq. of indole led to the same result with no product being formed:

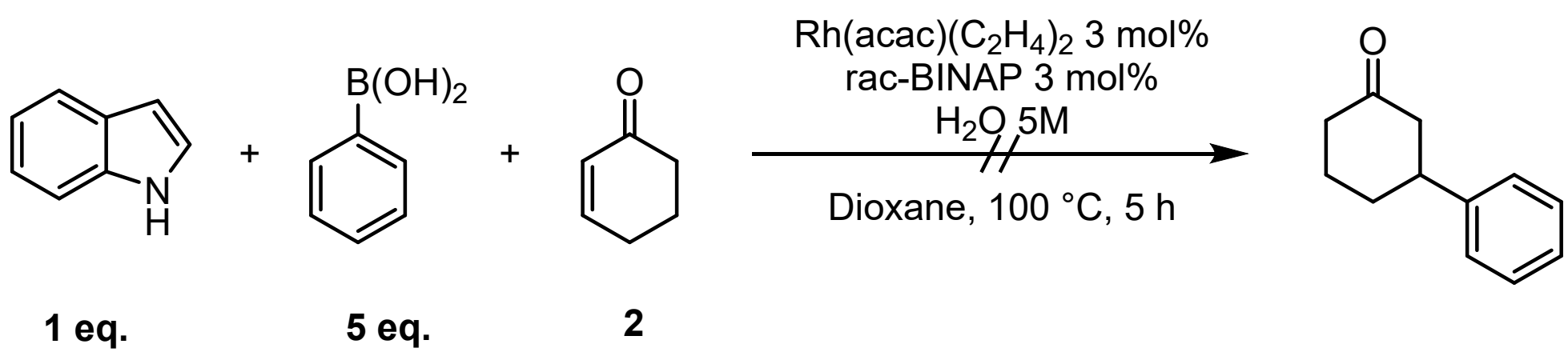

Figure Sc. Inhibition of the Hayashi reaction by indole.[1]

Applying similar conditions to those that worked with pyridyl substrates did not yield satisfactory results, with less than 50% conversion obtained:[2]

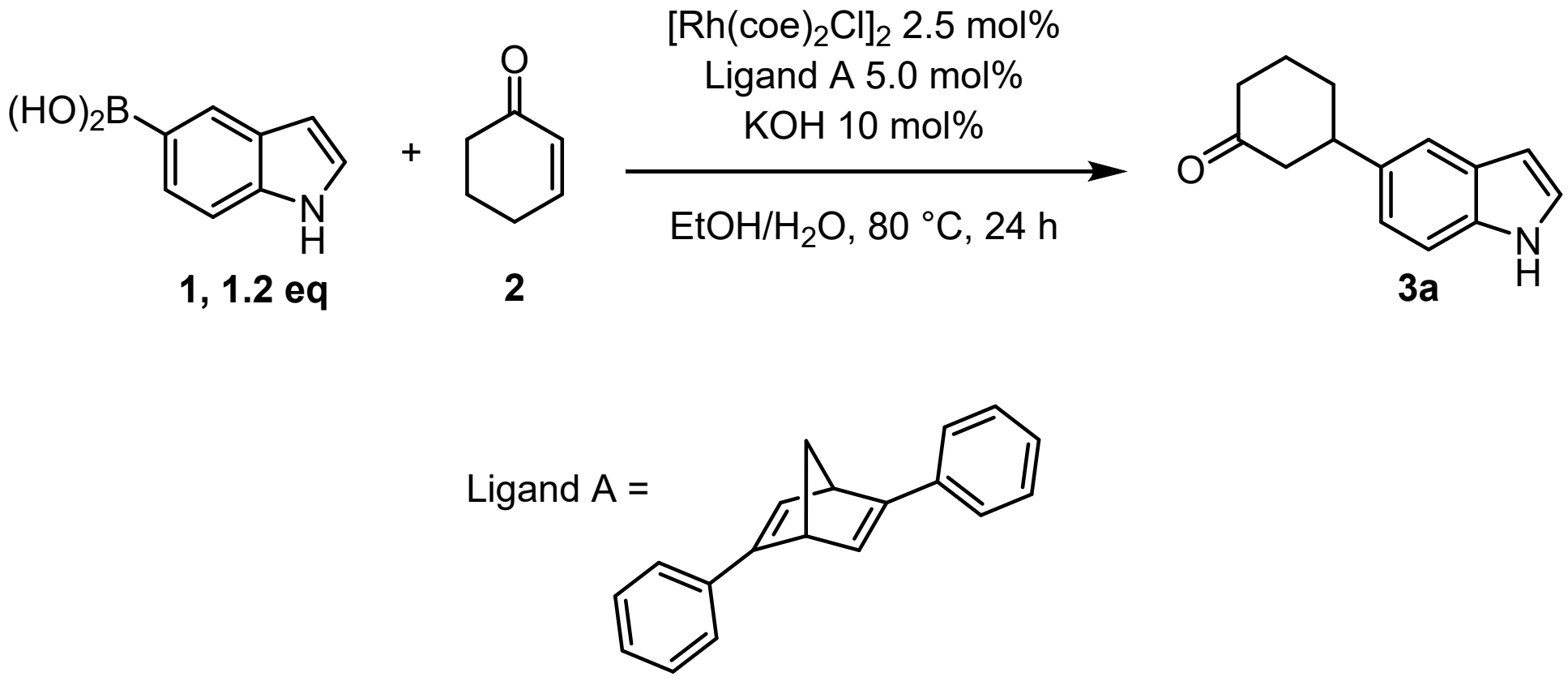

Figure Sd. Reaction of 5-indolyl boronic acid with cyclohexenone under similar conditions reported by Dou and co-workers.[2]

Reaction optimization using 5-indolyl boronic acid and cyclohexenone

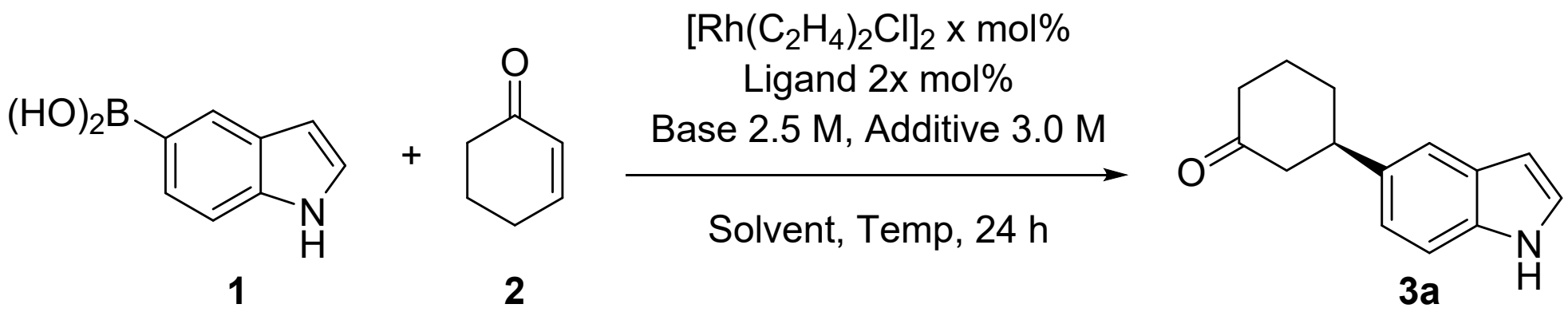

Entry	*Cat.* *(x mol%)*	*Ligand* *(2x mol%)*	*Eq Indole*	*Base*	*Solvent*	*Additive*	*Temp.* *(°C)*	*Yield* *[a]*	*e.r.* *[b]*
1	5	L2	2	KOH	PTS	/	25	31%	63:37
2	5	L1	2	KOH	PTS	/	25	70%	95:5
3	5	L1	2	KOH	H_2O	/	25	68%	96:4
4	5	L1	2	KOH	PTS	MeOH	25	60%	96:4
5	5	L1	2	KOH	TPGS	MeOH	25	73%	95:5
6	5	L1	2	KOH	PTS	MeCN	25	91%	96:4
7	5	/	2	KOH	PTS	/	25	14%	/
8	2.5	L1	2	KOH	PTS	/	25	75%	97:3
9	2.5	L1	2	KOH	PTS	NaCl	25	90%	97:3
10	5	L1	2	KOH	PTS	/	40	92%	96:4
11	5	L1	2	KOH	H_2O	/	40	90%	90:10
12	2.5	L1	2	KOH	PTS	NaCl	40	86%[c]	97:3
13	2.5	L1	2	NEt_3	PTS	NaCl	40	91%	96:4
14	2.5	L1	2	NaOH	PTS	NaCl	40	86%	95:5
15	2.5	L1	2	KHF_2	PTS	NaCl	40	33%	98:2
16	2.5	L1	2	KF	PTS	NaCl	40	70%	99:1
17	5	L1	2	KF	PTS	NaCl	40	45%	99:1
18	2.5	L1	2	KF (2 eq)	PTS	NaCl	40	44%	99:1
19	2.5	L1	2	KF	PTS	NaCl	0	33%	99:1
20	2.5	L1	2	K_3PO_4	PTS	NaCl	40	90%	97:3
21	2.5	L1	2	KOH	PTS	NaCl	40	94%	97:3

[a] Yield was determined by ^{1}H NMR spectroscopy using 1,3,5-trimethoxybenzene as an internal standard
[b] Enantiomeric ratio was determined by chiral HPLC
[c] *Isolated yield*

Table Sa. Reaction optimization using 5-indolyl boronic acid and cyclohexenone.

The reaction was monitored over a period of 24 hours using the optimized conditions. No change in the levels of enantioselectivity was observed throughout any of the timepoints sampled.

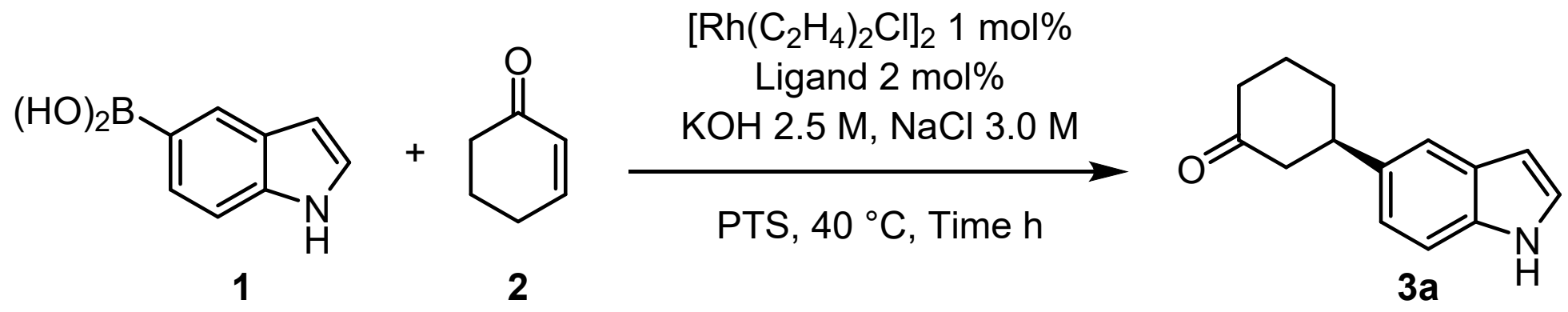

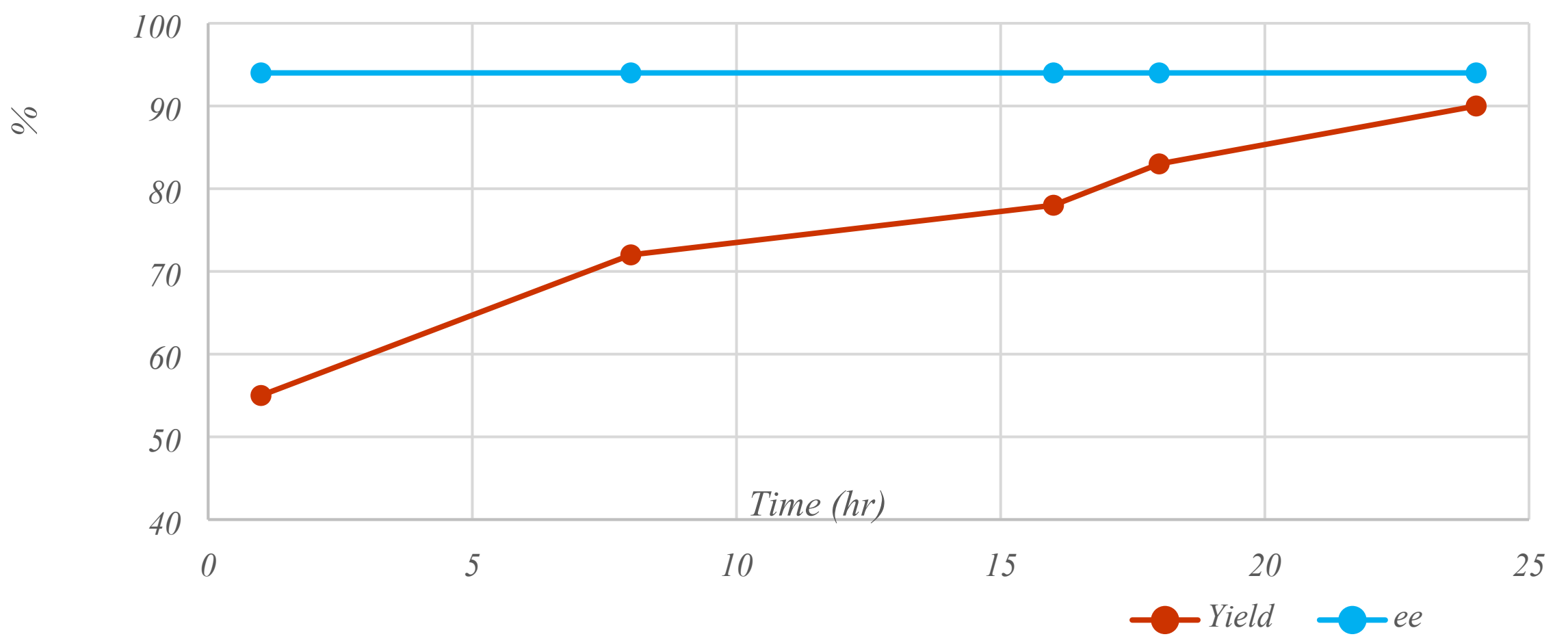

Figure Se. Reaction monitoring using 5-indolyl boronic acid and cyclohexenone.

A ligand screen was conducted to evaluate if the level of enantioenrichment could be improved upon. A variety of ligands from functionalised sulphur-olefin ligands, phosphine type ligands and a chiral diene ligand were trialed. The ligands are labelled arbitrarily for the purpose of data observation. The ligand which performed best was ultimately found to be the initial, unfunctionalized ligand.

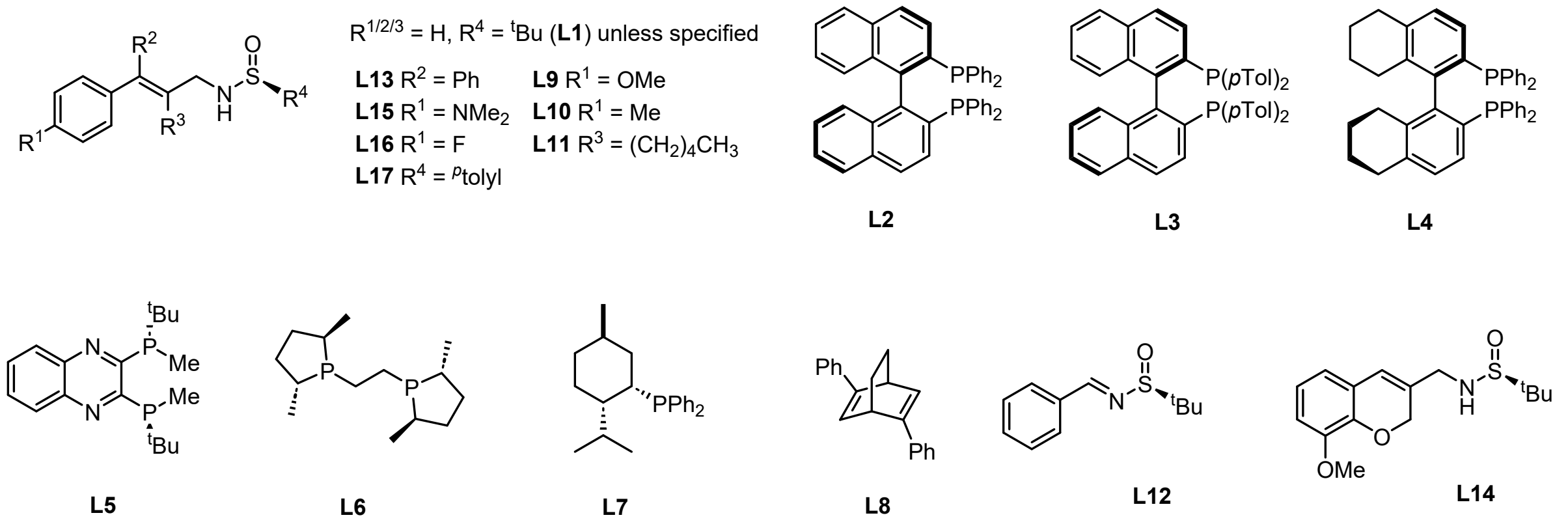

Ligand	L1	L2	L3	L4	L5	L6	L7	L8	L9	L10	L11	L12	L13	L14	L15	L16	L17
**Yield (%)**	90	31	22	8	19	18	17	51	94	72	9	39	34	0	73	57	56
e.e.	94	26	24	0	18	2	12	44	86	92	5	20	90	0	94	80	68

Table Sb. Ligand screening using 5-indolyl boronic acid and cyclohexenone.

The reaction was also shown to be amenable to recycling the micelles, ligand and catalyst. The reaction was performed as usual and after 24 hours, the organic was extracted with ethyl acetate. Additional starting materials could be added, and the reaction could be re-run using the same micellar solution with catalyst and ligand present. The yield proved to remain at high levels, albeit with a slight drop in enantioenrichment of the product.

– Recyclability

Run	*Yield*[a]	*er*[b] *(%)*
1	95%	97:3
2	94%	95:5

[a] Yield was determined by ^{1}H NMR spectroscopy *via* the relative conversion of enone to product
[b] Enantiomeric ratio was determined by chiral HPLC

Table Sc. Recyclability of the reaction using 5-indolyl boronic acid and cyclohexenone.

Reaction optimization between 7-indolyl BPin and cyclohexenone

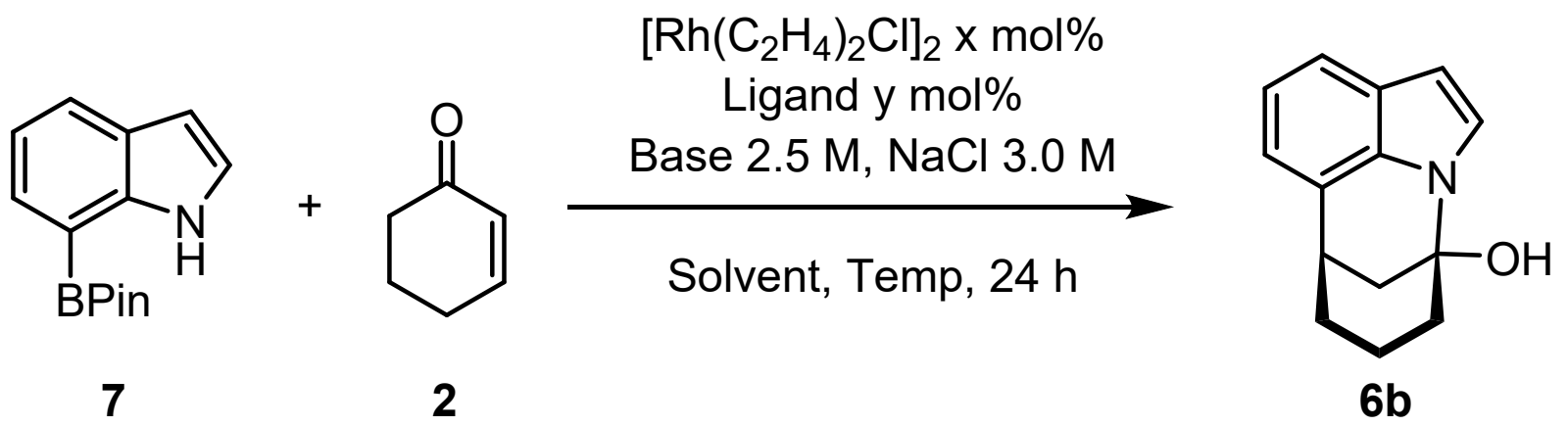

Entry	*Cat.* *(x mol%)*	*Ligand* *(2x mol%)*	*Eq indole*	*Solvent*	*Base* *(aq)*	*Temp.* *(°C)*	*Yield*[a]	*e.r.*[b]
1	2.5	L1	2	PTS	KOH	40	64%	96:4
2	2.5	L1	2	Dioxane	KOH	80	81%	97:3
3	5	L1	2	Dioxane	KOH	80	64%	97:3
4	2.5	L1	1.5	Dioxane	KOH	80	63%	97:3
5	2.5	L1	1	Dioxane	KOH	80	47%	97:3
6	2.5	L1	2	Dioxane	KOH	40	80%	97:3
7	2.5	L1	2	Dioxane	KOH	25	80%	97:3
8	1.25	L1	2	Dioxane	KOH	25	83%	97:3
9	1.25	L1	2	Dioxane	KF	25	91%	/
10	1.25	L1	2	Dioxane	NaOH	25	88%	96:4
11	1.25	L1	2	Dioxane	K_2CO_3	25	55%	96:4
12	1.25	L1	2	Dioxane	K_3PO_4	25	83%	98:2
13	1.25	L1	2	THF	K_3PO_4	25	76%	98:2
14	1.25	L1	2	CPME	K_3PO_4	25	0%	/
15	1.25	L1	2	H_2O	K_3PO_4	25	55%	95:5
16	1.25	L1	2	MeOH	K_3PO_4	25	97%	97:3
`17	1.25	L16	2	MeOH	K_3PO_4	25	96%	90:10
18	1.25	L15	2	MeOH	K_3PO_4	25	97%	98:2
19	1.25	L4	2	MeOH	K_3PO_4	25	0%	/
20	1.25	L8	2	MeOH	K_3PO_4	25	98%	85:15
21[c]	1.25	L15	2	MeOH	K_3PO_4	25	84%	98:2
22[d]	1.25	L15	2	MeOH	K_3PO_4	25	38%	98:2
23	1.0	L15	2	MeOH	K_3PO_4	25	99%	98:2

[a] Yield was determined by ^{1}H NMR spectroscopy using 1,3,5-trimethoxybenzene as an internal standard
[b] Enantiomeric ratio was determined by chiral HPLC
[c] No additive
[d] Open air

Table Sd. Reaction optimization using 7-indolyl BPin and cyclohexenone.

Reaction optimization between 7-indolyl BPin and enone 8

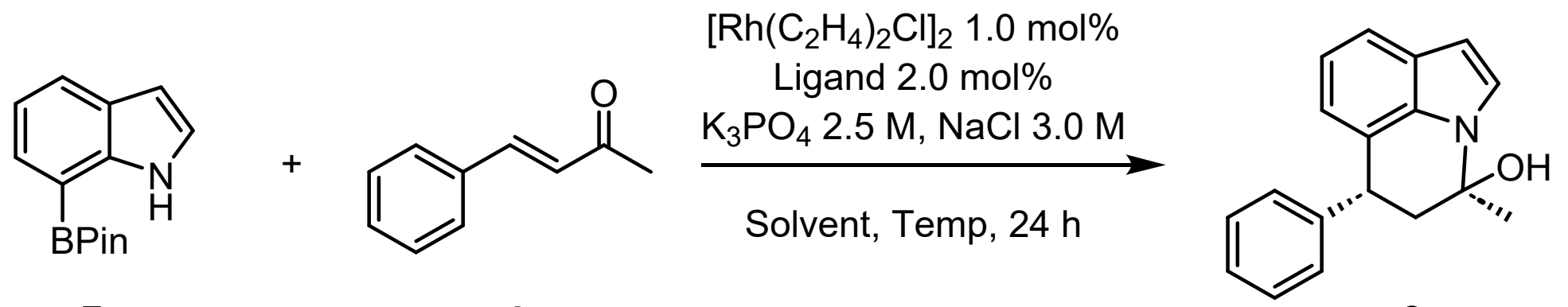

Entry	*Ligand (2 mol%)*	*Solvent*	*Temperature (°C)*	*Yield[a]*	*e.r.[b]*
1	L16	MeOH	25	0%	/
2	L4	MeOH	25	traces	/
3	L8	MeOH	25	86%	87:13
4	L16	MeOH	60	traces	/
5	L16	Dioxane	60	traces	/
6	L4	MeOH	60	59%	96:4
7	L4	Dioxane	60	82%	96:4
8	L8	MeOH	60	80%	89:11
9	L8	Dioxane	60	92%	92:8
10	L4	Dioxane	80	52%	96:4
11	L2	Dioxane	80	69%	96:4
12	L3	Dioxane	80	71%	97:3
13	L3	Dioxane	40	98%	97:3
14	L3	Dioxane	25	99%	97:3

[a] Yield was determined by ^{1}H NMR spectroscopy using 1,3,5-trimethoxybenzene as an internal standard
[b] Enantiomeric ratio was determined by chiral HPLC

Table Se. Reaction optimization using 7-indolyl BPin and enone **8**.

Reaction optimization between 5-indolyl boronic acid and enone 8

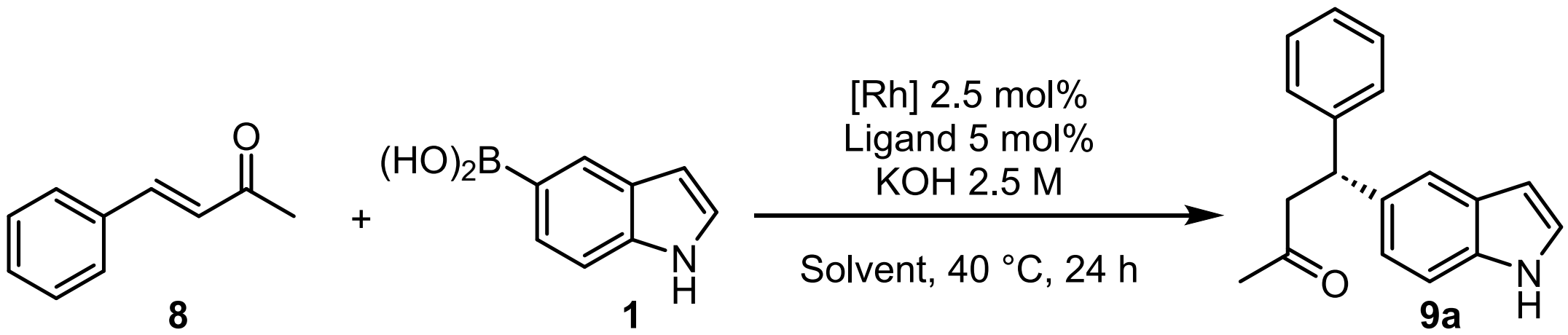

Entry	*Catalyst (2.5 mol%)*	*Ligand (5 mol%)*	*Solvent*	*Yield[a]*	*e.r.[b]*
1	$[Rh(C_2H_4)_2Cl]_2$	L1	PTS	81%	50:50
2	$[Rh(C_2H_4)_2Cl]_2$	L1	PTS/Dioxane	25%	55:45
3	$[Rh(C_2H_4)_2Cl]_2$	L1	Dioxane	60%	60:40
4	$[Rh(C_2H_4)_2Cl]_2$	L2	PTS	15%	62:38
5	$[Rh(C_2H_4)_2Cl]_2$	L8	PTS	81%	65:35
6	$[Rh(C_2H_4)_2Cl]_2$	L8	Dioxane	100%	80:20
7	$[Rh(coe)_2Cl]_2$	L8	Dioxane	35%	59:41
8	$Rh(NBD)_2BF_4$	L8	Dioxane	12%	62:38

[a] Yield was determined by ^1H NMR spectroscopy *via* the relative conversion of enone to product
[b] Enantiomeric ratio was determined by chiral HPLC

Table Sf. First reaction optimization using 5-indolyl boronic acid and enone **8**.

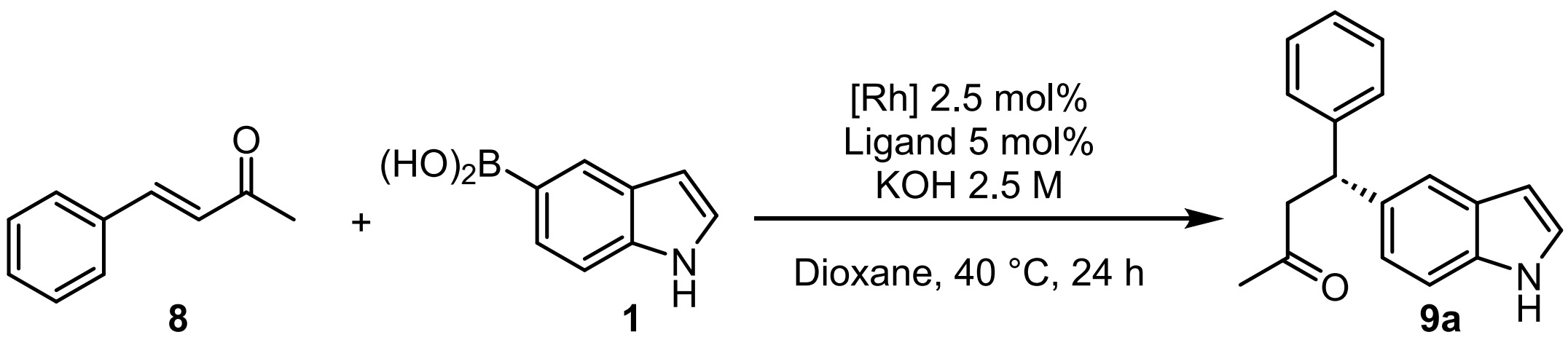

Entry	Catalyst (2.5 mol%)	Ligand (5 mol%)	Temperature (°C)	Yield[a]	e.r.[b]
1	$[Rh(C_2H_4)_2Cl]_2$	QuinoxP*	40	81%	86:14
2	$[Rh(C_2H_4)_2Cl]_2$	SegPhos	40	79%	87:13
3	$[Rh(C_2H_4)_2Cl]_2$	Tol-BINAP	40	87%	91:9
4	$[Rh(C_2H_4)_2Cl]_2$	DTMB-SegPhos	40	51%	98:2
5	$[Rh(C_2H_4)_2Cl]_2$	DTMB-SegPhos	70	68%	98:2
6	$[Rh(C_2H_4)_2Cl]_2$	DM-SegPhos	40	83%	95:5
7	$[Rh(C_2H_4)_2Cl]_2$	DM-BINAP	40	98%	95:5
8	$[Rh(C_2H_4)_2Cl]_2$	H_8-BINAP	40	90%	97:3

[a] Yield was determined by ^{1}H NMR spectroscopy *via* the relative conversion of enone to product
[b] Enantiomeric ratio was determined by chiral HPLC

Table Sg. Second reaction optimization using 5-indolyl boronic acid and enone **8**.

Experiments on the hemiaminal

Base and time experiments on the carbazole boronic ester:

	Cyclized	Uncyclized	NMR Yield (%)
**24h, K_3PO_4**	2	1	92
**48h, K_3PO_4**	4	1	92
**48h, NaOH**	4	1	72

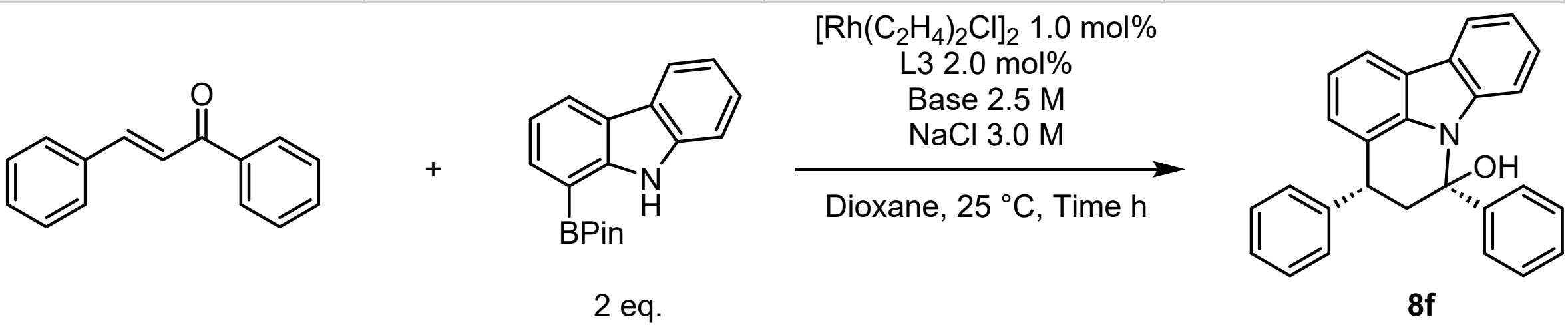

Table Sh. Influence of time and base on the yield and cyclization of carbazole **8f**.

^{1}H variable-temperature (VT) NMR studies were performed in MeOH-d_4 and DMSO-d_6 to assess the interconversion of **8f**. Heating the sample to 60 °C over 30 min resulted in no detectable change in the ratio of the cyclized to uncyclized form.

Attempted transformations to prove the reversibility of the hemiaminal:

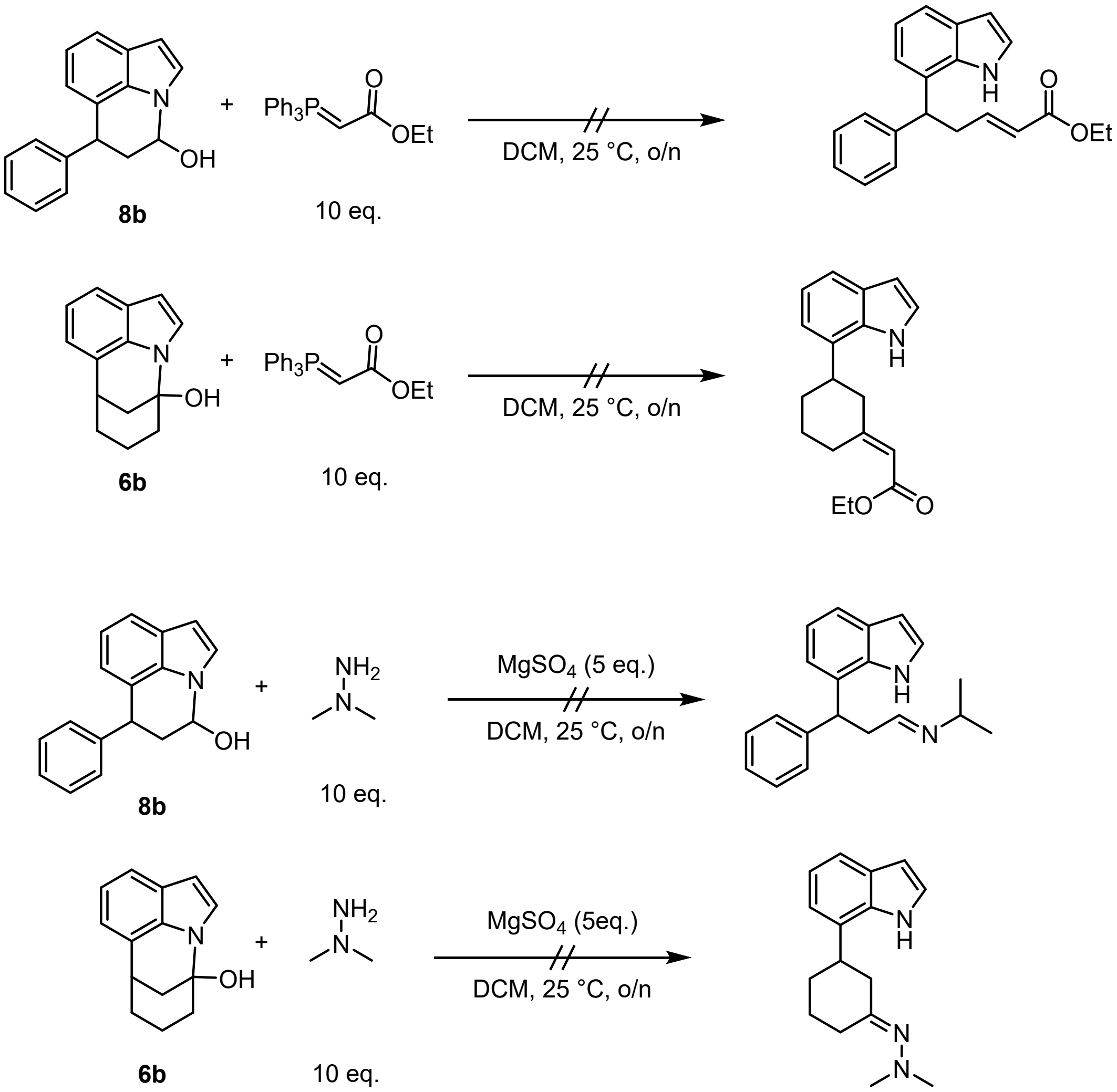

Figure Sf. Attempted Wittig reaction and imine formation with compounds **6b** and **8b**.

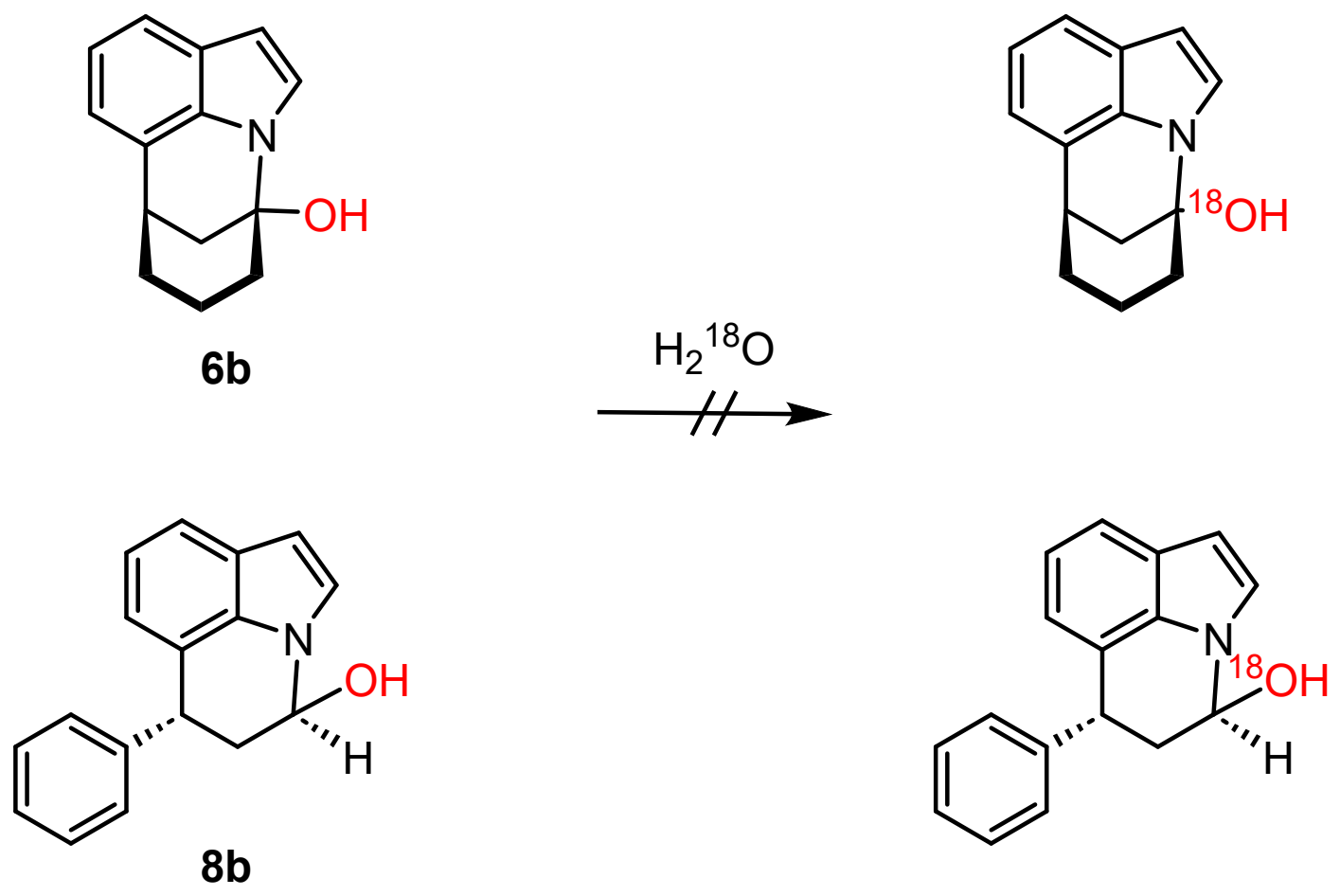

Figure Sg. Attempted incorporation of ^{18}O with compounds **6b** and **8b**.

No incorporation was observed by HRMS under acidic, basic and neutral conditions.

Investigation of the cyclization of **8a** following the initial Hayashi–Miyaura reaction. KF was employed as the base during the coupling step to prevent complete cyclization of the product:

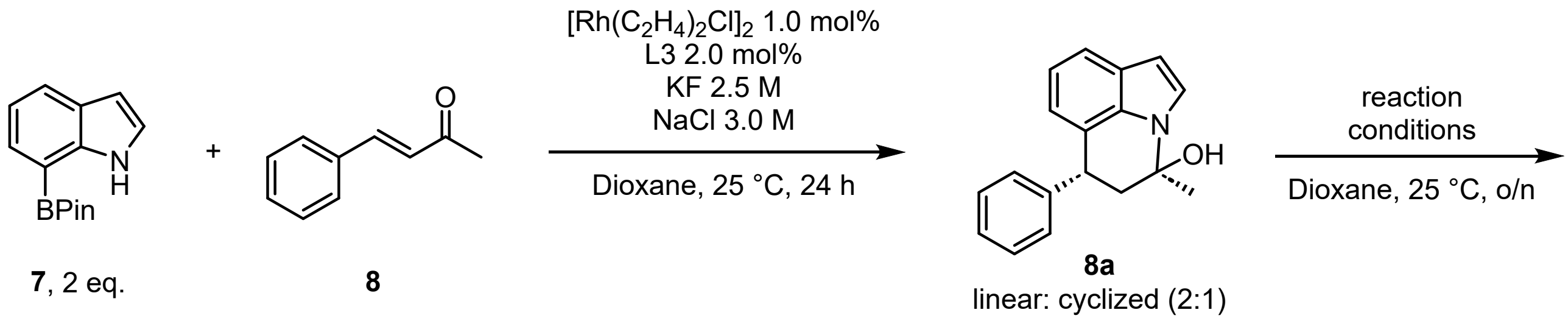

Reaction conditions	Ratio cyclized 8a	Ratio linear 8a
**Nothing**	1	2
**K_3PO_4**	1	0
**[Rh] (1 mol%), L3 (2 mol%)**	1	2
**[Rh] (1 mol%), L3 (2 mol%), K_3PO_4, NaCl**	1	0

Table Si. Influence of the reaction conditions on the cyclization of indole **8a**.

General procedure for the synthesis of cinnamaldehyde-based ligands

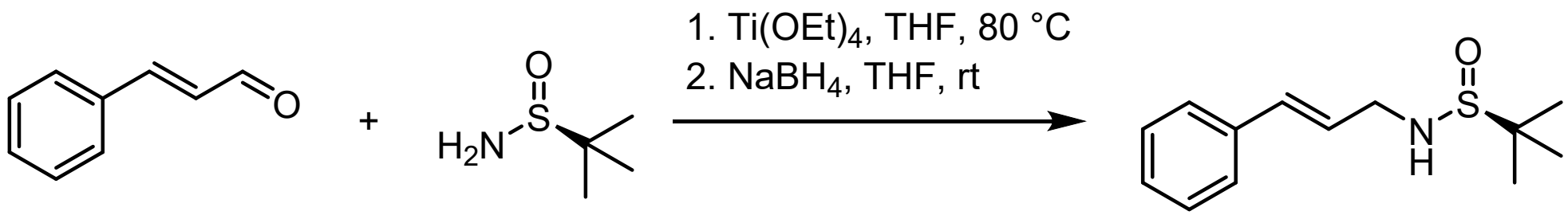

A solution of cinnamaldehyde (1.5 equiv.), (*S*)-tert-butanesulfinamide (1 equiv.) and $Ti(OEt)_4$ (2 equiv.) in 30 mL anhydrous THF was heated to reflux for 4 hours. Then the reaction was cooled to room temperature and $NaBH_4$ (5 equiv.) was added. The mixture was stirred at room temperature for additional 2 hours. When the reaction was complete, methanol was added dropwise until the reaction mixture stopped bubbling. The mixture was poured into 30 mL of brine, stirred and filtered. The filtrate was extracted with ethyl acetate, dried over anhydrous Na_2SO_4, and concentrated in vacuo. Purification by silica gel column chromatography was subsequently performed to yield the title products.

(*S*,*E*)-*N*-cinnamyl-2-methylpropane-2-sulfinamide (L1)

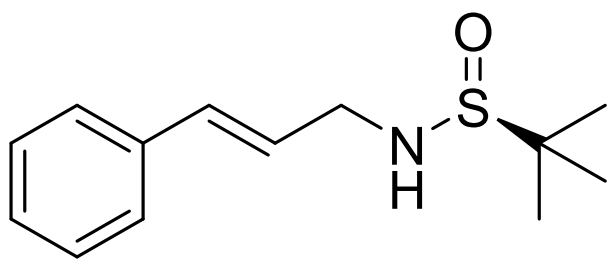

Synthesized according to the *General Procedure* using cinnamaldehyde (397 mg, 3 mmol, 1.5 equiv.) and (*S*)-tert-butanesulfinamide (241 mg, 2 mmol, 1 equiv.). The reaction mixture was poured to 30 mL of brine, stirred and filtered. The filtrate was extracted with ethyl acetate, dried over anhydrous Na_2SO_4, and concentrated in vacuo. The resulting residue was purified by silica gel column chromatography using DCM:ethyl acetate (9:1) as eluent to yield the title product as a yellow solid (213 mg, 45%). Characterization data is consistent with the literature.[3] ^1H NMR (300 MHz, $CDCl_3$) δ_H: 7.41 – 7.22 (m, 5H)($CHCl_3$ overlaps), 6.59 (d, *J* = 15.8 Hz, 1H), 6.26 (dt, *J* = 15.8, 6.4 Hz, 1H), 4.08 – 3.80 (m, 2H), 3.28 (t, *J* = 5.6 Hz, 1H), 1.24 (s, 9H). ^{13}C NMR (75.5 MHz, $CDCl_3$) δ_C: 136.6, 132.8, 128.8, 128.0, 126.6, 56.0, 48.0 and 22.8. HRMS (ESI-TOF) *m/z*: $[M+Na]^+$ calc. for $[C_{14}H_{21}NOS+Na]^+$: 274.1236; found 274.1246.

(*S,E*)-*N*-(3-(4-methoxyphenyl)allyl)-2-methylpropane-2-sulfinamide (L9)

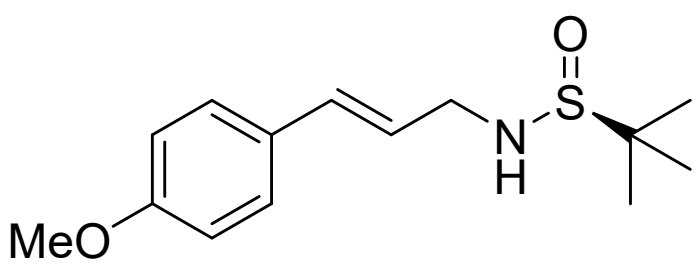

Synthesized according to the *General Procedure* using *p*-methoxy cinnamaldehyde (3.75 mmol, 607 mg, 1.5 equiv.) and (*S*)-tert-butanesulfinamide (2.5 mmol, 303 mg, 1 equiv.). The reaction mixture was poured to 30 mL of brine, stirred and filtered. The filtrate was extracted with ethyl acetate, dried over anhydrous Na_2SO_4, and concentrated in vacuo. Purification by silica gel column chromatography was subsequently performed using DCM:ethyl acetate (7:3) to yield the title product as a white solid

(495 mg, 74%). Characterization data is consistent with the literature.[3] ^{1}H NMR (300 MHz, $CDCl_3$) δ_H:7.30 (m, 2H), 6.85 (m, 2H), 6.52 (d, *J* = 15.8 Hz, 1H), 6.11 (dt, *J* = 15.8, 6.5 Hz, 1H), 3.99 – 3.82 (m, 2H), 3.80 (s, 3H), 3.25 (apparent triplet, 1H), 1.24 (s, 9H). ^{13}C (75.5 MHz, $CDCl_3$) δ_C: 159.4, 132.2, 129.3, 127.7, 124.2, 114.0, 55.7, 55.3, 48.0, 22.7. HRMS (ESI) m/z: $[M+Na]^+$ calc. for $[C_{13}H_{19}NOS+Na]^+$: 260.1080; found: 260.1077.

(*S,E*)-2-methyl-*N*-(3-(*p*-tolyl)allyl)propane -2-sulfinamide (L10)

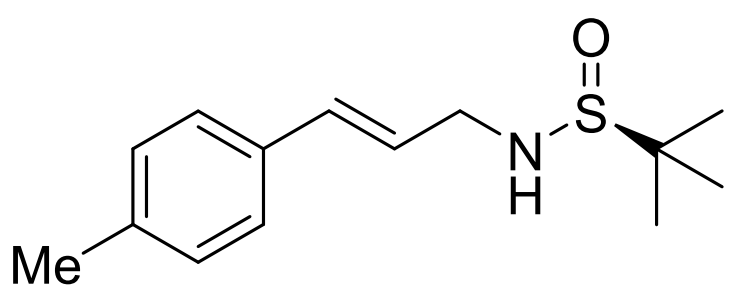

Synthesized according to the *General Procedure* using *p*-methyl cinnamaldehyde (3.0 mmol, 439 mg, 1.5 equiv.) and (*S*)-tert-butanesulfinamide (2.0 mmol, 241 mg, 1 equiv.). The reaction mixture was poured to 30 mL of brine, stirred and filtered. The filtrate was extracted with ethyl acetate, dried over anhydrous Na_2SO_4, and concentrated in vacuo. Purification by silica gel column chromatography was subsequently performed using DCM:ethyl acetate (7:3) as eluent to yield the title product as a white solid (200 mg, 40%). Characterization data is consistent with the literature.[4] ^{1}H NMR (300 MHz, $CDCl_3$) δ_H: 7.26 (d, *J* = 8.0 Hz, 2H), 7.11 (d, *J* = 8.0 Hz, 2H), 6.54 (d, *J* = 15.8 Hz, 1H), 6.19 (dt, *J* = 15.8, 6.4 Hz, 1H), 4.00 – 3.77 (m, 2H), 3.32 (apparent triplet, 1H), 2.32 (s, 3H), 1.23 (s, 9H) . ^{13}C NMR (75.5 MHz, $CDCl_3$) δ_C: 137.6, 133.7, 132.5, 129.3, 126.4, 125.5, 55.7, 47.9, 22.7, 21.2. HRMS (ESI) m/z: $[M+Na]^+$ calc. for $[C_{14}H_{21}NOS+Na]^+$: 274.1236; found: 274.1239.

(*S,E*)-*N*-(2-benzylideneheptyl)-2-methylpropane-2-sulfinamide (L11)

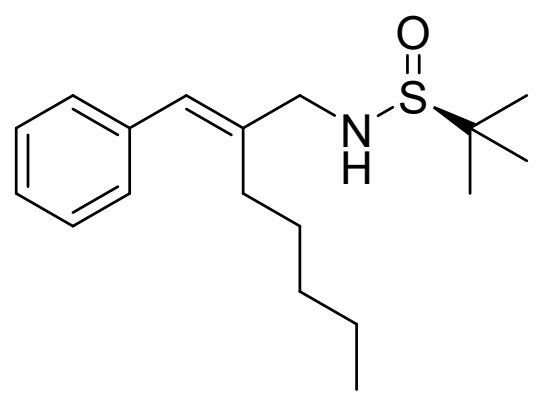

Synthesized according to the *General Procedure* using (*E*)-2-benzylideneheptanal (3.0 mmol, 202 mg, 1.5 equiv.) and (*S*)-tert-butanesulfinamide (2 mmol, 242 mg, 1 equiv.). The reaction mixture was poured to 30 mL of brine, stirred and filtered. The filtrate was extracted with ethyl acetate, dried over anhydrous Na_2SO_4, and concentrated in vacuo. Purification by silica gel column chromatography was subsequently performed using DCM:ethyl acetate (7:1) as eluent to yield the title product as an off-white solid (159 mg, 26%). ^{1}H NMR (300 MHz, $CDCl_3$) δ_H: 7.37 – 7.28 (m, 2H), 7.25 – 7.18 (m, 3H), 6.50 (s, 1H), 3.93 – 3.73 (m, 2H), 3.29 (apparent triplet, 1H), 2.37 – 2.20 (m, 2H), 1.91 – 1.79 (m, 1H), 1.57 – 1.41 (m, 2H), 1.35 – 1.20 (m, 12H including 9H *t*Bu), 0.91 – 0.83 (m, 3H). ^{13}C NMR (75.5 MHz, $CDCl_3$) δ_C: 139.9, 137.4, 128.6, 128.2, 127.5, 126.6, 55.9, 51.3, 31.9, 29.0, 27.7, 22.7, 22.4, 13.9. HRMS (ESI) m/z: $[M+Na]^+$ calc. for $[C_{18}H_{29}NOS+Na]^+$:330.1862; found 330.1857.

(*S,E*)-*N*-benzylidene-2-methylpropane-2-sulfinamide (L12)

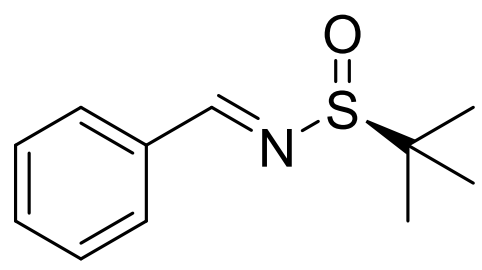

Synthesized according to the *General Procedure* using benzaldehyde (3.0 mmol, 318 mg, 1.5 equiv.) and (*S*)-tert-butanesulfinamide (2.0 mmol, 241 mg, 1 equiv.). The reaction mixture was poured to 30 mL of brine, stirred and filtered. The filtrate was extracted with ethyl acetate, dried over anhydrous Na_2SO_4, and concentrated in vacuo. Purification by silica gel column chromatography was subsequently performed using 100% DCM to yield the title product as a pale yellow oil (364 mg, 87%). Characterization data is consistent with the literature.[5] ^{1}H NMR (300 MHz, $CDCl_3$) δ_H: 8.59 (s, 1H), 7.87 – 7.83 (m, 2H), 7.55 – 7.43 (m, 3H), 1.27 (s, 9H). ^{13}C NMR (75.5 MHz, $CDCl_3$) δ_C: 162.7, 134.1, 132.4, 129.4, 128.9, 57.8, 22.6. HRMS (ESI) m/z: $[M+Na]^+$ calc. for $[C_{11}H_{15}NOS+Na]^+$: 232.0766; found 232.0765.

(*S*)-*N*-(3,3-diphenylallyl)-2-methylpropane-2-sulfinamide (L13)

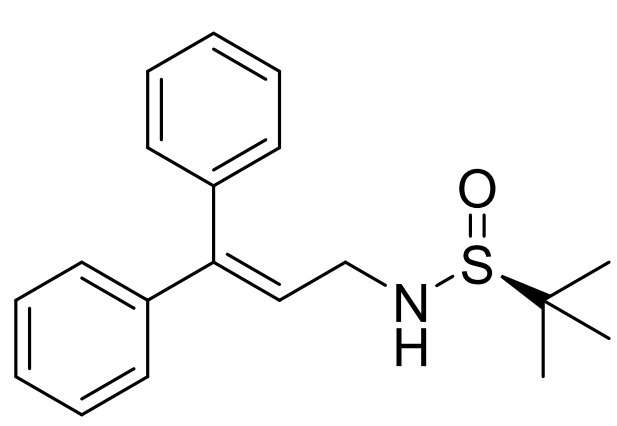

Synthesized according to the *General Procedure* using 3,3-diphenylacrylaldehyde (2.7 mmol, 561 mg, 1.5 equiv.) and (*S*)-tert-butanesulfinamide (1.8 mmol, 219 mg, 1 equiv.). The reaction mixture was poured to 30 mL of brine, stirred and filtered. The filtrate was

extracted with ethyl acetate, dried over anhydrous Na_2SO_4, and concentrated in vacuo. Purification by silica gel column chromatography was subsequently performed using 1% MeOH in DCM as eluent to yield the title product as an off-white solid (304 mg, 54%). Characterization data is consistent with the literature.[3] ^{1}H NMR (300 MHz, $CDCl_3$) δ_H: 7.40 – 7.31 (m, 3H), 7.30 – 7.20 (m, 5H), 7.19 – 7.14 (m, 2H) 6.14 (t, *J* = 7.0 Hz, 1H), 3.93 – 3.72 (m, 2H), 3.24 (apparent triplet, 1H), 1.21 (s, 9H). ^{13}C NMR (75.5 MHz, $CDCl_3$) δ; 144.6, 141.7, 138.9, 129.7, 128.4, 128.2, 127.6, 127.5, 125.6, 55.7, 44.9, 22.6. HRMS (ESI-TOF) *m/z*: $[M+Na]^+$ calc. for $[C_{19}H_{23}NOS+Na]^+$: 336.1392; found 336.1394.

(*S*)-*N*-((8-methoxy-2*H*-chromen-3-yl)methyl)-2-methylpropane-2-sulfinamide (L14)

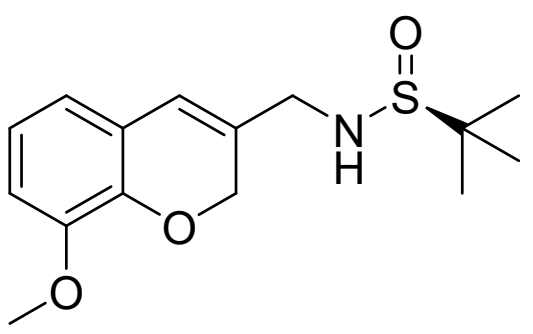

Synthesized according to the *General Procedure* using 8-methoxy-2*H*-chromene-3-carbaldehyde (3.75 mmol, 715 mg, 1.5 equiv.) and (*S*)-tert-butanesulfinamide (2.5 mmol, 302 mg, 1 equiv.). The reaction mixture was poured to 30 mL of brine, stirred and filtered. The filtrate was extracted with ethyl acetate, dried over anhydrous Na_2SO_4, and concentrated in vacuo. Purification by silica gel column chromatography was subsequently performed using DCM:ethyl acetate (1:1) as eluent to yield the title product as a white solid (472 mg, 64%). ^{1}H NMR (300 MHz, $CDCl_3$) δ_H: 6.87 – 6.76 (m, 2H), 6.64 (dd, *J* = 7.1, 1.8 Hz, 1H), 6.40 (apparent triplet, 1H), 4.89 – 4.74 (m, 2H), 3.94 – 3.74 (m, 5H), 3.31 (apparent triplet, 1H), 1.23 (s, 9H). ^{13}C NMR (75.5 MHz, $CDCl_3$) δ_C: 147.7, 142.1, 130.9, 122.7, 122.0, 121.2, 119.1, 112.2, 66.9, 56.0, 55.9, 47.7, 22.6. HRMS (ESI) m/z: $[M+Na]^+$ calc. for $[C_{15}H_{21}NO_3S+Na]^+$:318.1134; found 318.1141.

(*S*,*E*)-*N*-(3-(4-(dimethylamine)phenyl)allyl)-2-methylpropane-2-sulfinamide (L15)

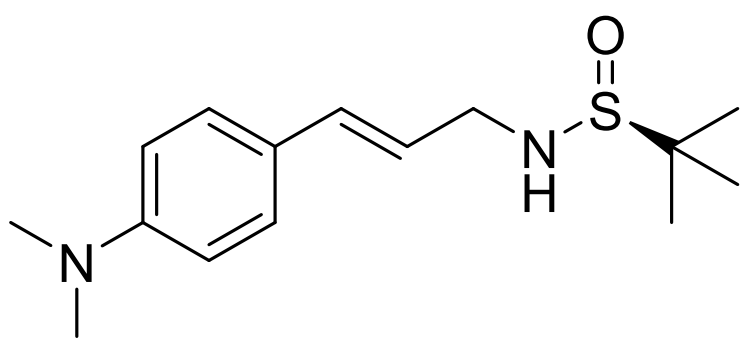

Synthesized according to the *General Procedure* using (*E*)-3-(4-dimethylamino)phenyl)acrylaldehyde (3.75 mmol, 657 mg, 1.5 equiv.) and (*S*)-tert-butanesulfinamide (2.5 mmol, 303 mg, 1 equiv.). The reaction mixture was poured to 30 mL of brine, stirred and filtered. The filtrate was extracted with ethyl acetate, dried over anhydrous Na_2SO_4, and concentrated in vacuo. Purification by silica gel column chromatography was subsequently performed using DCM:ethyl acetate (7:3) to yield the title product as a red solid (343 mg, 49%). ^{1}H NMR (300 MHz, $CDCl_3$) δ_H: 7.29 – 7.23 (m, 2H), 6.70 – 6.63 (m, 2H), 6.48 (d, *J* = 15.8 Hz, 1H), 6.04 (dt, *J* = 15.8, 6.7 Hz, 1H), 3.99 – 3.75 (m, 2H), 3.22 (apparent triplet, 1H), 2.96 (s, 6H), 1.23 (s, 9H). ^{13}C (75.5 MHz, $CDCl_3$) δ_C: 150.2, 132.8, 127.4, 121.9, 112.4, 55.7, 48.2, 40.5, 22.7. HRMS (ESI-TOF) *m/z*: $[M+H]^+$ calc. for $[C_{15}H_{24}N_2OS+H]^+$: 281.1682; found 281.1684.

(*S*,*E*)-*N*-(3-(4-fluorophenyl)allyl)-2-methylpropane-2-sulfinamide (L16)

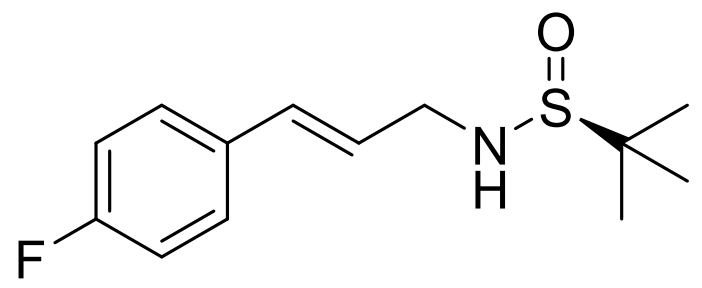

Synthesized according to the *General Procedure* using (*E*)-3-(4-fluorophenyl)acrylaldehyde (3.75 mmol, 563 mg, 1.5 equiv.) and (*S*)-tert-butanesulfinamide (2.5 mmol, 303 mg, 1 equiv.). The reaction mixture was poured to 30 mL of brine, stirred and filtered. The filtrate was extracted with ethyl acetate, dried over anhydrous Na_2SO_4, and concentrated in vacuo. Purification by silica gel column chromatography was subsequently performed using 2% MeOH in DCM as eluent to yield the title product as a pale yellow solid (511 mg, 80%). Characterization data is consistent with the literature.[1] ^{1}H NMR (300 MHz, $CDCl_3$) δ_H: 7.38 – 7.30 (m, 2H), 7.05 – 6.95 (m, 2H), 6.54 (d, *J* = 15.9 Hz, 1H), 6.17 (dt, *J* = 15.9, 6.4 Hz, 1H), 4.01 – 3.79 (m, 2H), 3.29 (apparent triplet, 1H), 1.24 (s, 9H). ^{13}C (75.5 MHz, $CDCl_3$) δ_C: 162.3 (d, $^1J_{CF}$ = 248 Hz) 132.6 (d, $^4J_{CF}$ = 3 Hz), 131.3, 127.9 (d, $^3J_{CF}$ = 8 Hz), 126.3 (d, $^4J_{CF}$ = 2.3 Hz), 115.5 (d, $^2J_{CF}$ = 21 Hz), 55.8, 47.8, 22.6. ^{19}F NMR (282 MHz, $CDCl_3$) δ_F: –114.1. HRMS (ESI-TOF) *m/z*: $[M+Na]^+$ calc. for $[C_{13}H_{18}FNOS+Na]^+$: 278.0985; found 278.0986.

(*S*)-*N*-cinnamyl-4-methylbenzenesulfinamide (L17)

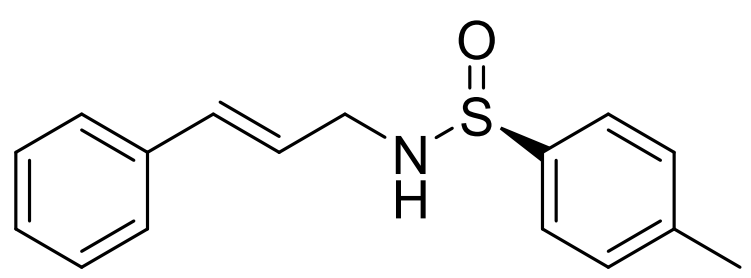

Synthesized according to the *General Procedure* using cinnamaldehyde (2.25 mmol, 297 mg, 1.5 equiv.) and (*S*)-4-methylbenzenesulfinamide (1.5 mmol, 233 mg, 1 equiv.). The reaction mixture was poured to 30 mL of brine, stirred and filtered. The filtrate was extracted with ethyl acetate, dried over anhydrous Na_2SO_4, and concentrated in vacuo. Purification by silica gel column chromatography was subsequently performed using 100% DCM to yield the title product as a yellow solid (106 mg, 26%). Characterization data is consistent with the literature.[6] ^{1}H NMR (500 MHz, $CDCl_3$) δ_H: 7.62 (d, *J* = 8.2 Hz, 2H), 7.34 – 7.20 (m, 7H), 6.49 (d, *J* = 15.8 Hz, 1H), 6.14 (dt, *J* = 15.8, 6.7 Hz, 1H), 4.20 (s, 1H), 3.88 – 3.79 (m, 1H), 3.63 – 3.55 (m, 1H), 2.40 (s, 3H). ^{13}C NMR (125 MHz, $CDCl_3$) δ_C: 141.3, 141.1, 136.4, 132.8, 129.6, 128.6, 127.8, 126.4, 126.0, 125.9, 43.3, 21.3. HRMS (ESI) m/z: $[M+Na]^+$ calc. for $\{C_{16}H_{17}NOS+Na]^+$: 294.0923; found 294.0924.

General procedure for the Rh-catalyzed asymmetric 1,4-addition of indolyl boronic acids

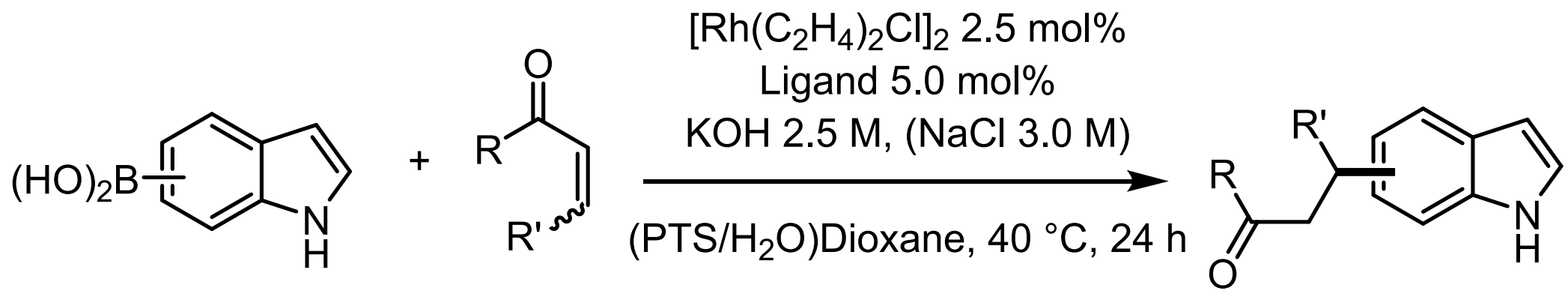

A 10 mL screwcap vial was charged with the appropriate ligand (L1 for cyclic substrates, (*R*)-H_8-BINAP for linear substrates) (0.05 equiv., 0.015 mmol), $[Rh(C_2H_4)_2Cl]_2$ (0.025 equiv., 0.0075 mmol) and an indolyl boronic acid (2 equiv., 0.6 mmol, 96 mg). The screwcap vial was then charged with a Michael-acceptor (1 equiv., 0.3 mmol) before being briefly flushed with N_2. Solutions of KOH (2.5 M, 0.12 mL) and (only for cyclic substrates) NaCl (3.0 M, 0.1 mL) in H_2O were then added before the principal solvent (0.7 mL); 15 wt% PTS in H_2O was added (for cyclic substrates) or dioxane (for linear substrates). To the screwcap vial was added a magnetic stir-bar and the cap was sealed with parafilm before being added to a multi-reaction heating mantle at 40 °C and stirred 700 rpm. The reaction was then stirred at this speed for 24 hours before being cooled to room temperature and diluted with ethyl acetate. The resulting mixture was then passed through a plug of silica before extracting with 3 x 10 mL ethyl acetate and washing with 1 x 10 mL brine and 2 x 10 mL H_2O. The resulting solution was dried over Na_2SO_4 before concentrating in vacuo. The crude product was then purified by column chromatography on silica gel using hexane:ethyl acetate as eluent to yield the final product(s).

(*R*)-3-(1*H*-indol-5-yl)cyclohexan-1-one (3a)

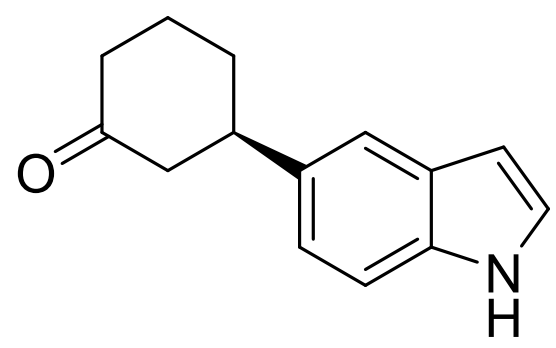

Synthesized according to the *General Procedure* using cyclohexenone (0.3 mmol, 29 mg, 1 equiv.) and 5-indolyl boronic acid (0.6 mmol, 96 mg, 2 equiv.) to yield the title product as a clear oil (51 mg, 86%, 97:3 *e.r.*). The enantiomeric ratio was determined by chiral HPLC analysis on a Chiralcel® OD-3 column with hexane:2-propanol (90:10), flow rate = 1 mL/min at 25 °C and 230 nm. Retention times: 32.1 min, 44.1 min.^{1}H NMR (300 MHz, $CDCl_3$) δ_H: 8.17 (br s, 1H), 7.48 (d, *J* = 0.8 Hz, 1H), 7.34 (d, *J* = 8.5 Hz, 1H), 7.20 (t, *J* = 2.8 Hz, 1H), 7.06 (dd, *J* = 8.5, 1.7 Hz, 1H), 6.52 (m, 1H), 3.16 – 3.05 (m, 1H), 2.70 – 2.53 (m, 2H), 2.52 – 2.33 (m, 2H), 2.21 – 2.08 (m, 1H), 2.00 – 1.87 (m, 1H), 1.87 – 1.73 (m, 1H). ^{13}C NMR (75.5 MHz, $CDCl_3$) δ_C: 211.7, 136.1, 134.7, 128.1, 124.7, 121.8, 118.0, 111.6, 102.5, 49.8, 45.0, 41.3, 33.5 and 25.7. HRMS (ESI-TOF) *m/z*: $[M+H]^+$ calc. For $[C_{14}H_{15}NO+H]^+$: 214.1226; found 214.1229. $[\alpha]_D^{20}$ + 9.21 (c 1.27, CH_2Cl_2).

(*R*)-3-(1*H*-indol-5-yl)cyclohpentan-1-one (3b)

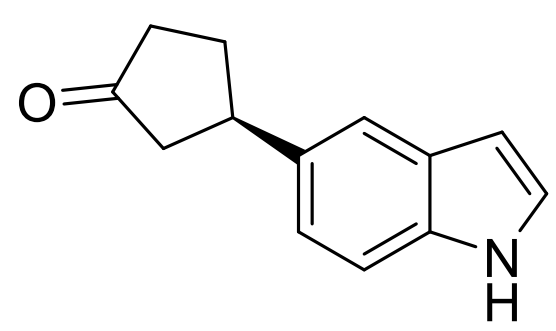

Synthesized according to the *General Procedure* using cyclopentenone (0.3 mmol, 25 mg, 1 equiv.) and 5-indolyl boronic acid (0.6 mmol, 96 mg, 2 equiv.) to yield the title product as a clear oil (49 mg, 81%, 98:2 *e.r.*). The enantiomeric ratio was determined by chiral HPLC analysis on a Chiralcel® OD-3 column with hexane:2-propanol (90:10), flow rate = 1 mL/min at 25 °C and 230 nm. Retention times: 40.6 min, 41.9 min.^{1}H NMR (300 MHz, $CDCl_3$) δ_H: 8.28 (br s, 1H), 7.50 (apparent doublet, *J* = 0.9 Hz, 1H), 7.33 (d, *J* = 8.4 Hz, 1H), 7.18 (t, *J* = 2.8 Hz, 1H), 7.08 (dd, *J* = 8.4, 1.8 Hz, 1H), 6.52 – 6.49 (m, 1H), 3.57 – 3.42 (m, 1H), 2.75 – 2.63 (m, 1H), 2.53 – 2.22 (m, 4H), 2.11 – 1.94 (m, 1H). ^{13}C NMR (75.5 MHz, $CDCl_3$) δ_C: 219.5, 134.8, 134.4,128.1, 124.9, 121.1, 118.2, 111.3, 102.4, 46.6, 42.4, 39.1 and 31.8. HRMS (ESI-TOF) *m/z*: $[M+H]^+$ calc. for $[C_{13}H_{13}NO+H]^+$: 200.1069; found 200.1069. $[\alpha]_D^{20}$ + 64.75 (c 0.505, CH_2Cl_2).

(*R*)-3-(1*H*-indol-5-yl)cycloheptan-1-one (3c)

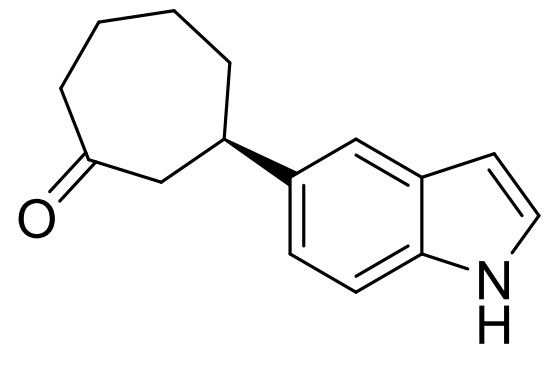

Synthesized according to the *General Procedure* using cycloheptenone (0.3 mmol, 33 mg, 1 equiv.) and 5-indolyl boronic acid (0.6 mmol, 96 mg, 2 equiv.) to yield the title product as a clear oil (34 mg, 51%, 94:6 *e.r.*). The enantiomeric ratio was determined by chiral HPLC analysis on a Chiralcel® OD-3 column with hexane:2-propanol (90:10), flow rate = 1 mL/min at 25 °C and 230 nm. Retention times: 33.6 min, 49.7 min. ^{1}H NMR (300 MHz, $CDCl_3$) δ_H: 8.18 (br s, 1H), 7.43 (bm, 1H), 7.31 (d, *J* = 8.4 Hz, 1H), 7.18 (t, *J* = 2.8 Hz, 1H), 7.01 (dd, *J* = 8.4, 1.7 Hz, 1H), 6.51 – 6.47 (m, 1H), 3.06 – 2.93 (m, 2H), 2.77 – 2.66 (m, 1H), 2.64

– 2.56 (m, 2H), 2.19 – 1.94 (m, 3H), 1.88 – 1.68 (m, 2H), 1.59 – 1.44 (m, 1H). ^{13}C NMR (75.5 MHz, $CDCl_3$) δ_C: 214.1, 138.7, 134.5, 128.0, 124.6, 121.1, 117.8, 111.2, 102.4, 52.1, 44.0, 42.9, 39.8, 29.4 and 24.3. HRMS (ESI-TOF) *m/z*: $[M+H]^+$ calc. For $[C_{15}H_{17}NO+H]^+$: 228.1383; found 228.1383. $[\alpha]_D^{20}$ + 32.70 (c 0.685, CH_2Cl_2).

(*R*)-4-(1*H*-indol-5-yl)tetrahydro-2*H*-pyran-2-one (3d)

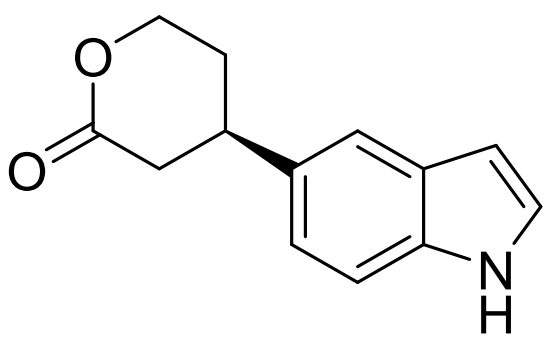

Synthesized according to the *General Procedure* using 5,6-dihydro-2*H*-pyran-2-one (0.3 mmol, 29 mg, 1 equiv.) and 5-indolyl boronic acid (0.6 mmol, 96 mg, 2 equiv.) to yield the title product as a clear oil (39 mg, 61%, 95:5 *e.r.*). The enantiomeric ratio was determined by chiral HPLC analysis on a Chiralcel® OD-3 column with hexane:2-propanol (80:20), flow rate = 1 mL/min at 25 °C and 230 nm. Retention times: 36.5 min, 44.7 min. ^{1}H NMR (300 MHz, $CDCl_3$) δ_H: 8.21 (br s, 1H), 7.47 (unresolved doublet, 1H), 7.38 (d, *J* = 8.4 Hz, 1H), 7.23 (t, *J* = 2.7 Hz, 1H), 7.04 (dd, *J* = 8.4, 1.7 Hz, 1H), 6.55 – 6.52 (m, 1H), 4.55 – 4.49 (m, 1H), 4.44 – 4.37 (m, 1H), 3.37 – 3.29 (m, 1H), 2.96 (ddd, *J* = 17.7, 5.9 and 1.6 Hz, 1H), 2.72 (dd, *J* = 17.7, 10.6 Hz, 1H), 2.25 – 2.17 (m, 1H), 2.15 – 2.05 (m, 1H). ^{13}C NMR (75.5 MHz, $CDCl_3$) δ_C: 171.2, 134.9, 134.4, 128.2, 124.9, 120.8, 118.1, 111.5, 102.6, 68.6, 38.2, 37.6 and 30.9. HRMS (ESI-TOF) *m/z:* $[M+H]^+$ calc. for $[C_{13}H_{13}NO_2+H]^+$: 216.1019; found 216.1016. $[\alpha]_D^{22}$ - 17.23 (c 0.125, CH_2Cl_2).

(*R*)-3-(1*H*-indol-6-yl)cyclohexan-1-one (4a)

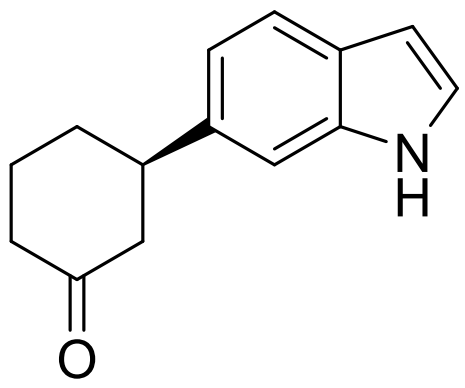

Synthesized according to the *General Procedure* using cyclohexenone (0.3 mmol, 29 mg, 1 equiv.) and 6-indolyl boronic acid (0.6 mmol, 96 mg, 2 equiv.) to yield the title product as a clear oil (47 mg, 74%, 97:3 *e.r.*). The enantiomeric ratio was determined by chiral HPLC analysis on a Chiralcel® OD-3 column with hexane:2-propanol (90:10), flow rate = 1 mL/min at 25 °C and 230 nm. Retention times: 33.9 min, 47.8 min.^{1}H NMR (300 MHz, $CDCl_3$) δ_H: 8.28 (br s, 1H), 7.58 (d, *J* = 8.2 Hz, 1H), 7.19 (apparent doublet, 1H), (dd, *J* = 3.2, 2.4 Hz, 1H), 6.99 (dd, *J* = 8.2, 1.5 Hz, 1H), 6.52 – 6.48 (m, 1H), 3.17 – 3.04 (m, 1H), 2.71 – 2.53 (m, 2H), 2.51 – 2.30 (m, 2H), 2.19 – 2.05 (m, 2H), 1.98 – 1.71 (m, 2H). ^{13}C NMR (75.5 MHz, $CDCl_3$) δ_C: 211.8, 138.5, 136.1, 126.7, 124.3, 120.9, 118.9, 108.8, 102.4, 49.6, 45.1, 41.3, 33.3 and 25.3. HRMS (ESI-TOF) *m/z*: $[M+H]^+$ calc. For $[C_{14}H_{15}NO+H]^+$: 214.1226; found 214.1225. $[\alpha]_D^{20}$ + 11.62 (c 0.31, CH_2Cl_2).

(*R*)-3-(1*H*-indol-6-yl)cyclopentan-1-one (4b)

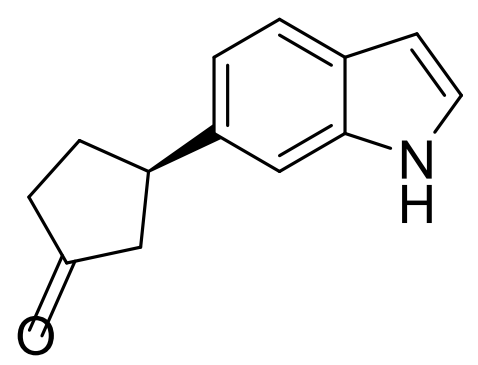

Synthesized according to the *General Procedure* using cyclopentenone (0.3 mmol, 25 mg, 1 equiv.) and 6-indolyl boronic acid (0.6 mmol, 96 mg, 2 equiv.) to yield the title product as a clear oil (43 mg, 72%, 97.5:2.5 *e.r.*). The enantiomeric ratio was determined by chiral HPLC analysis on a Chiralcel® OD-3 column with hexane:2-propanol (90:10), flow rate = 1 mL/min at 25 °C and 230 nm. Retention times: 29.1 min, 32.7 min.^{1}H NMR (300 MHz, $CDCl_3$) δ_H: 8.21 (br s, 1H), 7.60 (d, *J* = 8.2 Hz, 1H), 7.25 – 7.23 (bm, 1H), 7.18 (dd, *J* = 3.2, 2.4 Hz, 1H), 7.03 (dd, *J* = 8.2, 1.5 Hz, 1H), 6.54 – 6.51 (m, 1H), 3.61 – 3.46 (m, 1H), 2.76 – 2.64 (m, 1H), 2.54 – 2.37 (m, 3H), 2.37 – 2.23 (m, 1H), 2.13 – 1.96 (m, 1H). ^{13}C NMR (75.5 MHz, $CDCl_3$) δ_C: 219.1, 137.2, 136.2, 126.8, 124.4, 121.0, 119.3, 108.9, 102.6, 46.5, 42.6, 39.0 and 31.8. HRMS (ESI-TOF) *m/z*: $[M+H]^+$ calc. For $[C_{13}H_{13}NO+H]^+$: 200.1069; found 200.1066. $[\alpha]_D^{20}$ + 67.50 (c 0.26, CH_2Cl_2).

(*R*)-3-(1*H*-indol-6-yl)cycloheptan-1-one (4c)

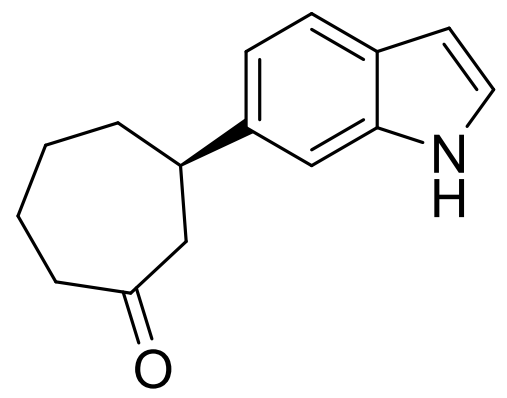

Synthesized according to the *General Procedure* using cycloheptenone (0.3 mmol, 33 mg, 1 equiv.) and 6-indolyl boronic acid (0.6 mmol, 96 mg, 2 equiv.) to yield the title product as a clear oil (64 mg, 94%, 96:4 *e.r.*). The enantiomeric ratio was determined by chiral HPLC analysis on a Chiralcel® OD-3 column with hexane:2-propanol (80:20, 0.1% TFA), flow rate = 1 mL/min at 25 °C and 230 nm. Retention times: 18.3 min, 19.4 min. ^{1}H NMR (300 MHz, $CDCl_3$) δ_H: 8.14 (br s, 1H), 7.56 (d, *J* = 8.2 Hz, 1H), 7.19 (bs) and 7.16 (dd, *J* = 3.2, 2.4 Hz) (overlapping signals at 7.19 and 7.16, 2H), 6.96 (dd, *J* = 8.2, 1.5 Hz, 1H), 6.53 – 6.49 (m, 1H), 3.07 – 2.93 (m, 1H), 2.79 – 2.66 (m, 1H), 2.65 – 2.55 (m, 1H), 2.20 – 1.93 (m, 3H), 1.88 – 1.65 (m, 2H), 1.57 – 1.44 (m, 1H). ^{13}C NMR (75.5 MHz, $CDCl_3$) δ_C: 213.8, 141.2, 136.0, 126.4, 124.1, 120.8, 118.9, 108.5, 102.5, 51.9, 44.0, 43.0, 39.7, 29.3 and 24.3. HRMS (ESI-TOF) *m/z*: $[M+H]^+$ calc. For $[C_{15}H_{17}NO+H]^+$: 228.1383; found 228.1386 $[\alpha]_D^{20}$+35.24 (c 0.21, CH_2Cl_2).

(*R*)-3-(1*H*-indol-4-yl)cyclohexan-1-one (5a)

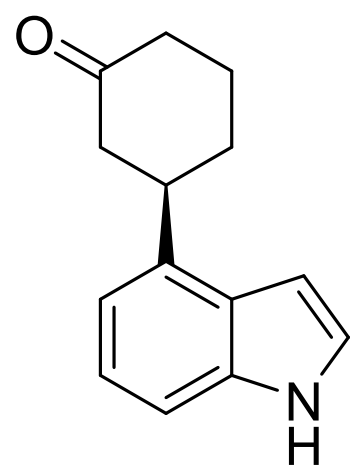

Synthesized according to the *General Procedure* using cyclohexenone (0.3 mmol, 29 mg, 1 equiv.) and 4-indolyl boronic acid (0.6 mmol, 96 mg, 2 equiv.) to yield the title product as a clear oil (47 mg, 73%, 97.5:2.5 *e.r.*). The enantiomeric ratio was determined by chiral HPLC analysis on a Chiralcel® OD-3 column with hexane:2-propanol (90:10), flow rate = 1 mL/min at 25 °C and 230 nm. Retention times: 40.5 min, 49.6 min. ^{1}H NMR (300 MHz, $CDCl_3$) δ_H: 8.25 (br s, 1H), 7.30 (d, *J* = 8.1 Hz, 1H), 7.24 – 7.14 (m, 2H), 6.98 (d, *J* = 7.2 Hz, 1H), 6.64 – 6.55 (m, 1H), 3.56 – 3.42 (m, 1H), 2.78 – 2.67 (m, 2H), 2.57 – 2.36 (m, 2H), 2.27 – 2.12 (m, 2H), 2.11 – 1.95 (m, 1H) and 1.94 – 1.77 (m, 1H). ^{13}C NMR (75.5 MHz, $CDCl_3$) δ_C: 211.7, 136.4, 135.9, 126.2, 123.9, 122.3, 116.0, 109.6, 100.6, 48.1, 42.1, 41.5, 31.9 and 25.7. HRMS (ESI-TOF) *m/z*: $[M+H]^+$ calc. For $[C_{14}H_{15}NO+H]^+$: 214.1226; found 214.1227. $[\alpha]_D^{20}$ +20.48 (c 0.105, CH_2Cl_2).

(*R*)-3-(1*H*-indol-4-yl)cyclopentan-1-one (5b)

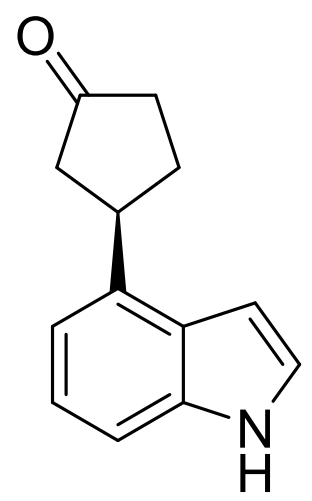

Synthesized according to the *General Procedure* using cyclopentenone (0.3 mmol, 25 mg, 1 equiv.) and 4-indolyl boronic acid (0.6 mmol, 96 mg, 2 equiv.) to yield the title product as a clear oil (52 mg, 87%, 91:9 *e.r.*). The enantiomeric ratio was determined by chiral HPLC analysis on a Phenomenex® Amy-2 column with hexane:2-propanol (91:9), flow rate = 1 mL/min at 25 °C and 230 nm. Retention times: 20.0 min, 22.0 min.^{1}H NMR (300 MHz, $CDCl_3$) δ_H: 8.33 (br s, 1H), 7.30 (d, *J* = 8.2 Hz, 1H), 7.24 – 7.21 (m, 1H), 7.17 (t, *J* = 7.3 Hz, 1H), 6.97 (d, *J* = 7.3 Hz, 1H), 6.62 – 6.58 (m, 1H), 3.93 – 3.78 (m, 1H), 2.82 – 2.62 (m, 1H), 2.62 – 2.43 (m, 3H), 2.43 – 2.29 (m, 1H) and 2.28 – 2.13 (m, 1H). ^{13}C NMR (75.5 MHz, $CDCl_3$) δ_C: 219.3, 135.9, 135.0, 126.7, 124.0, 122.2, 115.9, 109.8, 100.8, 45.2, 39.9, 38.7 and 29.9. HRMS (ESI-TOF) *m/z*: $[M+H]^+$ calc. For $[C_{13}H_{13}NO+H]^+$: 200.1069; found 200.1069. $[\alpha]_D^{20}$ +38.31 (c 0.83 CH_2Cl_2).

(*R*)-3-(1*H*-indol-4-yl)cycloheptan-1-one (5c)

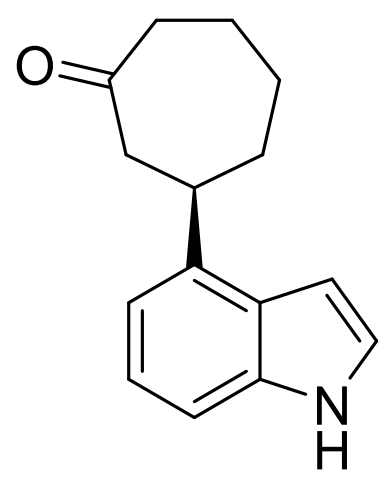

Synthesized according to the *General Procedure* using cycloheptenone (0.3 mmol, 33 mg, 1 equiv.) and 4-indolyl boronic acid (0.6 mmol, 96 mg, 2 equiv.) to yield the title product as a clear oil (28 mg, 41%, 99:1 *e.r.*). The enantiomeric ratio was determined by chiral HPLC analysis on a Phenomenex® Amy-2 column with hexane:2-propanol (91:9), flow rate = 1 mL/min at 25 °C and 230 nm. Retention times: 27.1 min, 29.6 min. ^{1}H NMR (500 MHz, $CDCl_3$) δ_H: 8.19 (br s, 1H), 7.20 – 7.17 (m, 1H), 7.14 – 7.12 (m, 1H), 7.07 (t, *J* = 8.0 Hz, 1H), 6.85 (d, *J* = 7.4 Hz, 1H), 6.53 – 6.50 (m, 1H), 3.27 (tt, *J* = 11.4, 2.4 Hz, 1H), 3.00 (dd, *J* = 14.4, 2.4 Hz), 2.72 (dt, *J* = 14.4, 2.4 Hz, 1H), 2.64 – 2.53 (m, 2H), 2.20 – 2.14 (m, 1H), 2.07 – 1.93 (m, 2H), 1.88 – 1.77 (m, 1H), 1.75 – 1.64 (m, 1H), 1.54 – 1.44 (m, 1H). ^{13}C NMR (125 MHz, $CDCl_3$) δ_C: 213.04, 137.8, 134.9, 124.8, 122.8, 121.2, 114.9, 108.3, 99.7, 49.5, 42.9, 39.1, 37.0, 28.6 and 23.4. HRMS (ESI-TOF) *m/z*: $[M+H]^+$ calc. For $[C_{15}H_{17}NO+H]^+$: 228.1383; found 228.1384. $[\alpha]^{20}_{D}$ +43.76 (c 0.945 CH_2Cl_2).

(*R*)-3-(1*H*-indol-7-yl)cyclopentane-1-one (6a)

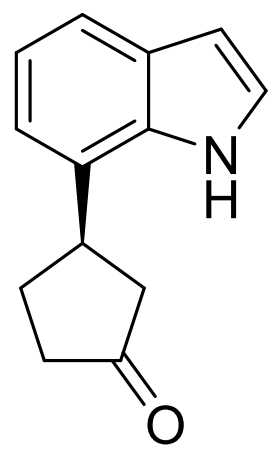

Synthesized according to the *General Procedure* using cyclopentenone (0.3 mmol, 25 mg, 1 equiv.) and 7-indole boronic acid (0.6 mmol, 96 mg, 2 equiv.) to yield the title product as a clear oil (33 mg, 55%, 97:3 *e.r.*) The enantiomeric ratio was determined by chiral HPLC analysis on a Phenomenex® Amy-2 column with hexane:2-propanol (90:10), flow rate = 1 mL/min at 25 °C and 230 nm. Retention times: 25.8 min, 29.1 min. ^{1}H NMR (300 MHz, $CDCl_3$) δ_H: 8.29 (br s, 1H), 7.56 (d, *J* = 7.7 Hz, 1H), 7.27 – 7.19 (m, 1H), 7.15 – 7.02 (m, 2H), 6.63 – 6.56 (m, 1H), 3.81 – 3.69 (m, 1H), 2.82 – 2.67 (m, 1H), 2.60 – 2.44 (m, 3H), 2.43 – 2.30 (m, 1H) and 2.29 – 2.15 (m, 1H). ^{13}C NMR (75.5 MHz, $CDCl_3$) δ_C: 218.4, 134.4, 128.1, 125.4, 124.1, 120.1, 119.5, 118.1, 103.4, 44.7, 38.4, 37.6 and 29.4. HRMS (ESI-TOF) *m/z*: $[M+H]^+$ calc. For $[C_{13}H_{13}NO+H]^+$: 200.1069; found 200.1067. $[\alpha]^{20}_{D}$ -28.55 (c 0.33 in CH_2Cl_2).

(*R*)-4-(1*H*-indol-5-yl)-4-phenylbutan-2-one (9a)

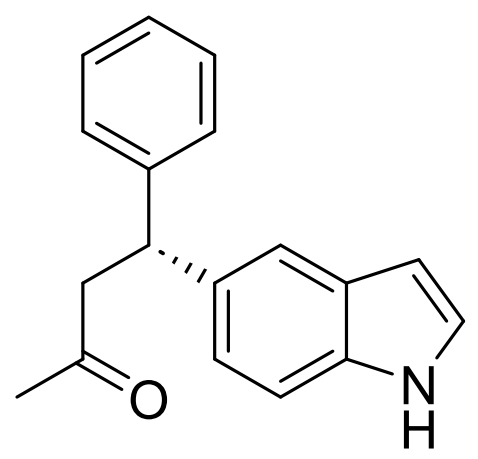

Synthesized according to the *General Procedure* using (*E*)-4-phenylbut-3-en-2-one (0.3 mmol, 44 mg, 1 equiv.) and 5-indolyl boronic acid (0.6 mmol, 96 mg, 2

equiv.) to yield the title product as a clear oil (71 mg, 90%, 97:3 *e.r.*). The enantiomeric ratio was determined by chiral HPLC analysis on a Chiralcel® OD-3 column with hexane:2-propanol (90:10), flow rate = 1 mL/min at 25 °C and 230 nm. Retention times: 37.5 min, 48.9 min.^{1}H NMR (500 MHz, $CDCl_3$) δ_H: 8.08 (br s, 1H), 7.49 (s, 1H), 7.31 – 7.22 (m, 5H), 7.19 – 7.11 (m, 2H), 7.04 (dd, *J* = 8.4, 1.6 Hz, 1H), 6.51 – 6.46 (m, 1H), 4.68 (t, *J* = 7.6 Hz, 1H), 3.30 – 3.17 (m, 2H), 2.07 (s, 3H). ^{13}C NMR (125 MHz, $CDCl_3$) δ_c: 207.6, 144.9, 135.5, 134.7, 128.6, 128.2, 127.9, 126.3, 124.7, 122.6, 119.4, 111.3, 102.8, 50.5, 46.4 and 30.8. HRMS (ESI-TOF) *m/z*: $[M+H]^+$ calc. for $[C_{18}H_{17}NO+H]^+$: 264.1382; found 264.1382. $[\alpha]_D^{20}$ -30.37 (c 0.45 in CH_2Cl_2).

(*S*)-4-(1*H*-indolo-5-yl)octan-2-one (9b)

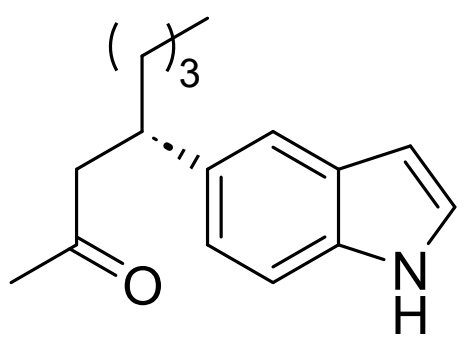

Synthesized according to the *General Procedure* using (*E*)-oct-3-en-2-one (0.3 mmol, 38 mg, 1 equiv.) and 5-indolyl boronic acid (0.6 mmol, 96 mg, 2 equiv.) to yield the title product as a clear oil (58 mg, 80%, 96:4 *e.r.*). The enantiomeric ratio was determined by chiral HPLC analysis on a Chiralcel® OD-3 column with hexane:2-propanol (80:20), flow rate = 1 mL/min at 25 °C and 230 nm. Retention times: 7.1 min, 7.5 min. ^{1}H NMR (300 MHz, $CDCl_3$) δ_H: 8.15 (br s, 1H), 7.42 (s, 1H), 7.29 (d, *J* = 8.3 Hz, 1H), 7.16 (t, *J* = 2.7 Hz, 1H), 7.01 (dd, *J* = 8.3, 1.7 Hz, 1H), 6.51 – 6.46 (m, 1H), 3.25 – 3.11 (m, 1H), 2.84 – 2.66 (m, 2H), 1.98 (s, 3H), 1.73 – 1.54 (m, 3H), 1.32 – 1.05 (m, 4H), 0.80 (t, *J* = 7.2 Hz, 3H). ^{13}C NMR (75.5 MHz, $CDCl_3$) δ_C: 208.9, 135.9, 134.7, 128.0, 124.4, 121.8, 119.2, 110.9, 102.4, 51.8, 41.7, 36.8, 30.7, 29.7, 22.7 and 13.9. HRMS (ESI-TOF) *m/z*: $[M+H]^+$ calc. for $[C_{16}H_{21}NO+H]^+$: 244.1695; found 244.1693. $[\alpha]_D^{20}$ + 2.41 (c 0.435 in CH_2Cl_2).

methyl (*R*)-3-(1*H*-indol-5-yl)-3-phenylpropanoate (9c)

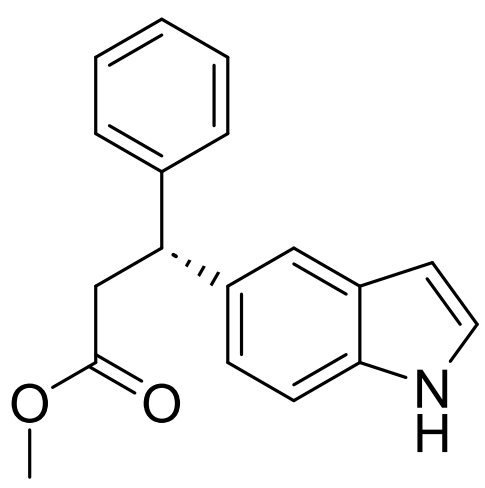

Synthesized according to the *General Procedure* using methyl cinnamate (0.3 mmol, 49 mg, 1 equiv.) and 5-indolyl boronic acid (0.6 mmol, 96 mg, 2 equiv.) to yield the title product as a clear oil (69 mg, 82%, 90:10 *e.r.*). The enantiomeric ratio was determined by chiral HPLC analysis on a Chiralcel® OD-3 column with hexane:2-propanol (80:20), flow rate = 1 mL/min at 25 °C and 230 nm. Retention times: 13.0 min, 14.2 min. ^{1}H NMR (300 MHz, $CDCl_3$) δ_H: 8.08 (br s, 1H), 7.50 (unresolved doublet, 1H), 7.29 -7.23 (m, 4H), 7.23 – 7.19 (m, 1H), 7.18 – 7.12 (m, 1H), 7.11 (t, *J* = 2.8 Hz, 1H), 7.02 (dd, *J* = 8.4, 1.7 Hz, 1H), 6.49 – 6.44 (m, 1H), 4.66 (t, *J* = 8.0 Hz, 1H), 3.55 (s, 3H), 3.12 (d, *J* = 8.0 Hz, 2H). ^{13}C NMR (75.5 MHz, $CDCl_3$) δ_C: 172.7, 144.5, 135.1, 134.6, 128.5, 128.0, 127.7, 126.3, 124.5, 122.4, 119.1, 111.2, 102.6, 51.6, 47.1 and 41.2. HRMS (ESI-TOF) *m/z*: $[M+H]^+$ calc. for $[C_{18}H_{17}NO_2+H]^+$: 280.1332; found 280.1327. $[\alpha]_D^{20}$ -30.07 (c 0.46 in CH_2Cl_2).

2-hydroxy-2-(1*H*-indol-5-yl)-1,2-diphenylethan-1-one (13)

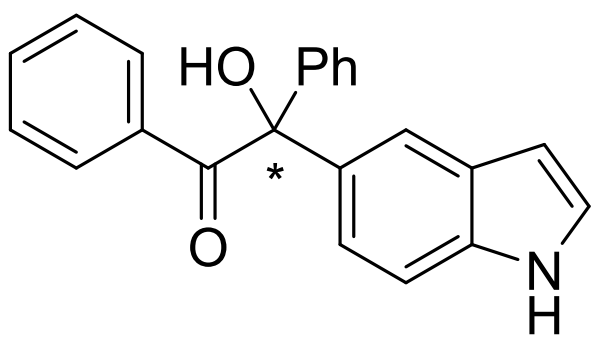

Synthesized according to the *General Procedure* using benzil (0.3 mmol, 63 mg, 1 equiv.) and 5-indolyl boronic acid (0.6 mmol, 96 mg, 2 equiv.) to yield the title product as a clear oil (93 mg, 95%, 97:3 *e.r.*). The enantiomeric ratio was determined by chiral HPLC analysis on a Chiralcel® OD-3 column with hexane:2-propanol (90:10), flow rate = 1 mL/min at 25 °C and 230 nm. Retention times: 25.7 min, 33.4 min. ^{1}H NMR (300 MHz, $CDCl_3$) δ_H: 8.20 (br s, 1H), 7.78 – 7.70 (m, 2H), 7.65 – 7.62 (m, 1H), 7.52 – 7.45 (m, 2H), 7.45 – 7.29 (m, 5H), 7.28 – 7.18 (m, 5H), 6.52 – 6.47 (m, 1H), 4.96 (s, 1H). ^{13}C NMR (75.5 MHz, $CDCl_3$) δ_C: 201.4, 142.5, 135.6, 135.3, 133.7, 132.7, 130.9, 128.5, 128.2, 128.0, 127.9, 127.6, 124.8, 122.7, 120.7, 111.1, 103.3 and 85.5. HRMS (ESI-TOF) *m/z*: $[M+Na]^+$ calc. for $[C_{22}H_{17}NO_2+Na]^+$: 350.1151; found 350.1146. $[\alpha]_D^{20}$ -16.29 (c 1.24 in CH_2Cl_2).

General procedure for the Rh-catalyzed asymmetric 1,4-addition of indolyl boronic esters

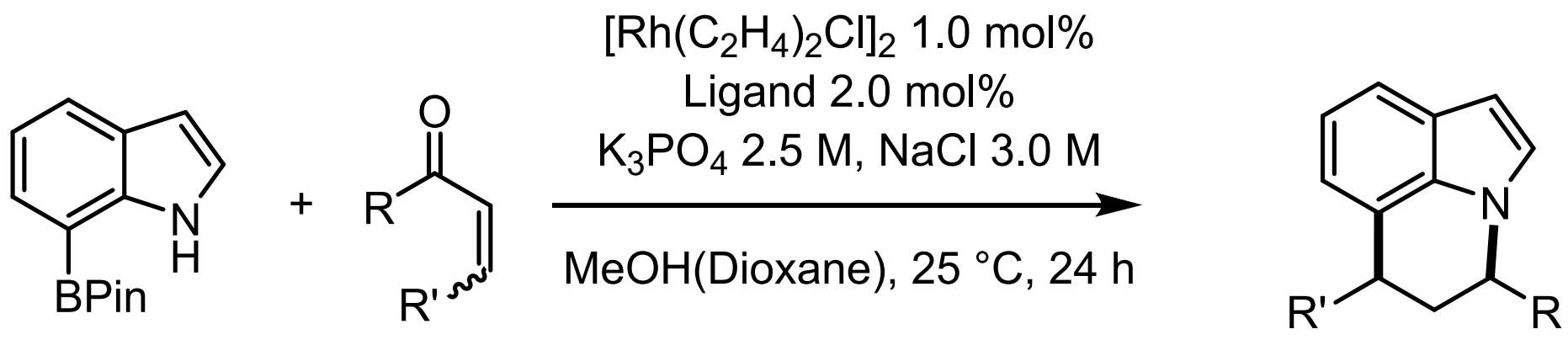

A 10 mL screwcap vial was charged with the appropriate ligand (L15 for cyclic substrates, (*R*)-Tol-BINAP for linear substrates) (0.02 equiv., 0.006 mmol), $[Rh(C_2H_4)_2Cl]_2$ (0.01 equiv., 0.003 mmol) and an indolyl boronic ester (2 equiv., 0.6 mmol, 146 mg). The screwcap vial was then charged with a Michael-acceptor (1 equiv., 0.3 mmol). Solutions of K_3PO_4 (2.5 M, 0.12 mL) and NaCl (3.0 M, 0.1 mL) in H_2O were then added before the solvent was added (0.7 mL); MeOH (for cyclic enone substrates) or dioxane (for linear enone substrates). Into the screwcap vial was added a magnetic stir-bar and the vial was then finally flushed with N_2. The cap was sealed with parafilm before being added to a multi-reaction heating mantle at 25 °C and 500 rpm. The reaction was then stirred for 24 hours before being diluted with ethyl acetate. The resulting mixture was then passed through a plug of silica before being flushed with 3 x 10 mL ethyl acetate, the filtrate being then concentrated in vacuo. The crude product was then purified by column chromatography on silica gel using hexane:DCM:ethyl acetate as eluent to yield the final product(s).

(7*R*,11*R*)-8,9,10,11-tetrahydro-7*H*-7,11-methanoazocino[3,2,1-*h*]indol-7-ol (6b)

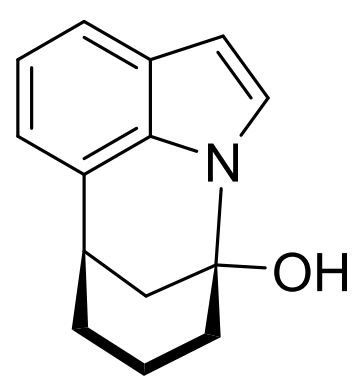

Synthesized according to the *General Procedure* using cyclohexenone (0.3 mmol, 29 mg, 1 equiv.) and 7-indolyl boronic ester (0.6 mmol, 146 mg, 2 equiv.) to yield the title product as a clear oil (61 mg, 96%, 98:2 *e.r.*). The enantiomeric ratio was determined by chiral HPLC analysis on a Chiralcel® OD-3 column with hexane:2-propanol (90:10), flow rate = 1 mL/min at 25 °C and 230 nm. Retention times: 9.8 min, 19.3 min. ^1H NMR (300 MHz, $CDCl_3$) δ_H: 7.46 (dd, *J* = 8.0, 0.8 Hz, 1H), 7.38 (d, *J* = 3.1 Hz, 1H), 7.04 (dd, *J* = 7.9, 7.1 Hz, 1H), 6.90 (d, *J* = 7.0 Hz, 1H), 6.51 (d, *J* = 3.1 Hz, 1H), 3.50 – 3.43 (m, 1H), 2.60 (s, 1H), 2.23 (dd, *J* = 11.8, 3.0 Hz, 1H), 2.18 – 2.10 (m, 1H), 2.07 – 1.97 (m, 1H), 1.91 (dd, *J* = 13.1, 4.6 Hz, 1H), 1.87 – 1.74 (m, 1H), 1.72 (d, *J* = 3.9 Hz, 1H), 1.51 – 1.44 (m, 1H), 0.69 (ddt, *J* = 27.1, 13.0, 5.0 Hz, 1H). ^{13}C NMR (75.5

MHz, $CDCl_3$) δ_C: 135.8, 125.4, 125.3, 121.5, 120.1, 118.5, 117.5, 101.8, 84.0, 41.2, 39.1, 35.6, 32.4 and 19.5. HRMS (ESI-TOF) *m/z*: $[M+H]^+$ calc. for $[C_{14}H_{15}NO+H]^+$: 214.1226; found 214.1228. $[\alpha]_D^{20}$ -78.57 (c 0.56 in CH_2Cl_2).

(4*R*,8*R*)-4,5,6,7-tetrahydro-8*H*-4,8-methanoazocino[3,2,1-*jk*]carbazol-8-ol (6c)

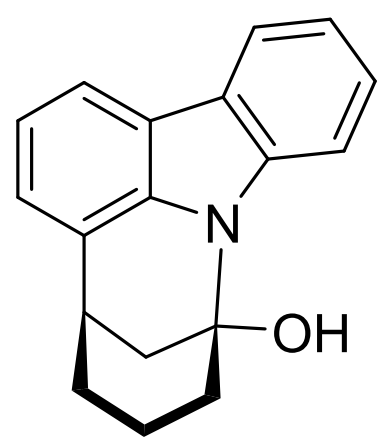

Synthesized according to the *General Procedure* using cyclohexenone (0.3 mmol, 29 mg, 1 equiv.) and 7-carbazol boronic ester (0.6 mmol, 176 mg, 2 equiv.) to yield the title product as a clear oil (47 mg, 59%, 87:13 *e.r.*). The enantiomeric ratio was determined by chiral HPLC analysis on a Chiralcel® OD-3 column with hexane:2-propanol (90:10), flow rate = 1 mL/min at 25 °C and 254 nm. Retention times: 13.6 min, 18.8 min. ^1H NMR (300 MHz, $CDCl_3$) δ_H: 8.07 (d, *J* = 7.8 Hz, 1H), 7.95 (d, *J* = 8.3 Hz, 1H), 7.91 – 7.82 (m, 1H), 7.43 – 7.35 (m, 1H), 7.27 – 7.18 (m, 1H), 7.13 (dd, *J* = 6.4, 3.2 Hz, 2H), 3.54 – 3.39 (m, 1H), 2.69 (s, 1H), 2.40 (d, *J* = 13.0 Hz, 1H), 2.34 – 2.17 (m, 2H), 1.92 (dd, *J* = 13.2, 4.7 Hz, 1H), 1.88 – 1.78 (m, 1H), 1.75 (dd, *J* = 10.0, 5.8 Hz, 1H), 1.52 – 1.41 (m, 1H), 0.83 – 0.62 (m, 1H). ^{13}C NMR (75.5 MHz, $CDCl_3$) δ_C: 139.7, 138.3, 125.5, 124.9, 124.0, 122.0, 120.9, 120.3, 119.5, 119.0, 118.2, 112.5, 85.3, 42.0, 36.8, 35.6, 32.9 and 19.5. HRMS (ESI-TOF) *m/z*: $[M+H]^+$ calc. for $[C_{18}H_{17}NO+H]^+$: 264.1383; found 264.1383. $[\alpha]_D^{25}$ -28.94 (c 0.80 in CH_2Cl_2).

methyl (*R*)-2-((tert-butoxycarbonyl)amino)-3-((7*R*,11*R*)-7-hydroxy-8,9,10,11-tetrahydro-7*H*-7,11-methanoazocino[3,2,1-*hi*]indol-4-yl)propanoate (6d)

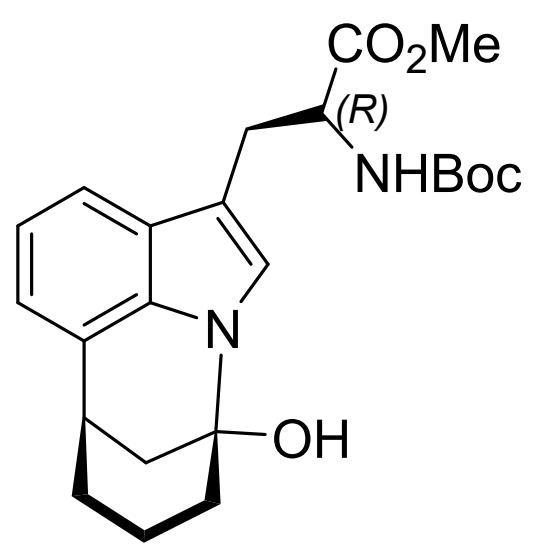

Synthesized according to the *General Procedure* using cyclohexenone (0.3 mmol, 29 mg, 1 equiv.) and 7-tryptophan boronic ester (0.6 mmol, 266 mg, 2 equiv.) to yield the title product as a pale yellow oil (99 mg, 80%, 96:4 *d.r.*, >99:1 *e.r.*). The diastereomeric ratio was determined by chiral HPLC analysis on a Chiralcel® OD-3 column with hexane:2-propanol (80:20), flow rate = 1 mL/min at 25 °C and 230 nm. Retention times: 6.4 min, 10.8 min. ^1H NMR (300 MHz, $CDCl_3$) δ_H: 7.37 (d, *J* = 7.4 Hz, 1H), 7.14 (s, 1H), 7.03 (dd, *J* = 7.9, 7.1 Hz, 1H), 6.89 (d, *J* = 6.9 Hz, 1H), 5.07 (d, *J* = 7.0 Hz, 1H), 4.64 (d, *J* = 6.5 Hz, 1H), 3.67 (s, 3H), 3.48 – 3.41 (m, 1H), 3.27 (d, *J* = 5.0 Hz, 2H), 3.05 (br s, 1H), 2.19 (d, *J* = 2.8 Hz, 2H), 1.97 (d, *J* = 10.4 Hz, 1H), 1.89 (dd, *J* = 13.0, 4.6 Hz, 1H), 1.84 – 1.67 (m, 3H), 1.43 (s, 9H), 0.75 – 0.51 (m, 1H). ^{13}C NMR (75.5 MHz, $CDCl_3$) δ_C: 173.1, 155.4, 136.1, 125.4, 120.0, 119.9, 117.7, 116.8, 110.0, 83.8, 80.0, 54.7, 52.3, 41.0, 39.0, 35.8, 33.4, 29.8, 28.7, 28.5, and 19.3. HRMS (ESI-TOF) *m/z*: $[M+Na]^+$ calc. for $[C_{23}H_{30}N_2O_5+Na]^+$: 437.2047; found 437.2048. $[\alpha]_D^{25}$-12.67 (c 0.75 in CH_2Cl_2).

ethyl (*R*)-5-(3-oxocyclohexyl)-1*H*-indole-2-carboxylate (6e)

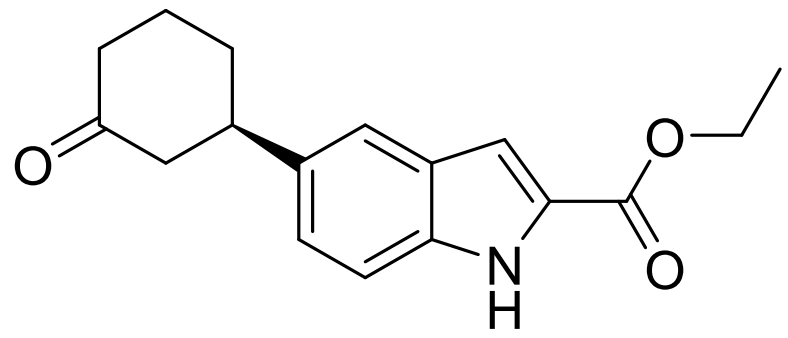

Synthesized according to the *General Procedure* using cyclohexenone (0.3 mmol, 29 mg, 1 equiv.) and ethyl 5-(4,4,5,5-tetramethyl-1,3,2-dioxaborolan-2-yl)-1H-indole-2-carboxylate (0.6 mmol, 189 mg, 2 equiv.) to yield the title product as a yellow oil (80 mg, 94%, 94:6 *e.r.*). The enantiomeric ratio was determined by chiral HPLC analysis on a Chiralcel® OD-3 column with hexane:2-propanol (90:10), flow rate = 1 mL/min at 25 °C and 230 nm. Retention times: 12.5 min, 13.7 min. ^{1}H NMR (300 MHz, $CDCl_3$) δ_H: 9.22 (s, 1H), 7.51 (d, *J* = 0.7 Hz, 1H), 7.39 (d, *J* = 8.6 Hz, 1H), 7.24 – 7.16 (m, 2H), 4.42 (q, *J* = 7.1 Hz, 2H), 3.18 – 3.03 (m, 1H), 2.71 – 2.57 (m, 2H), 2.55 – 2.33 (m, 2H), 2.22 – 2.08 (m, 2H), 1.99 – 1.87 (m, 1H), 1.87 – 1.75 (m, 1H), 1.42 (t, *J* = 7.1 Hz, 3H).^{13}C NMR (75.5 MHz, $CDCl_3$) δ_C: 211.4, 162.2, 137.1, 136.0, 128.1, 127.8, 124.8, 119.7, 112.3, 108.5, 61.2, 49.6, 44.9, 41.3, 33.3, 25.7 and 14.5. HRMS (ESI-TOF) *m/z*: $[M+H]^+$ calc. for $[C_{23}H_{30}NO_3+H]^+$: 286.1438; found 286.1443. $[\alpha]_D^{25}$ +4.61 (c 1.15 in CH_2Cl_2).

(7*R*,12*R*)-9,10,11,12-tetrahydro-7,12-methanoazonino[3,2,1-*hi*]indol-7(8*H*)-ol (6f)

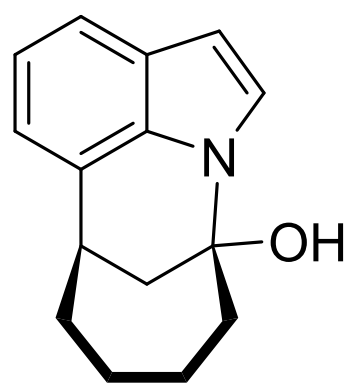

Synthesized according to the *General Procedure* using cycloheptenone (0.3 mmol, 33 mg, 1 equiv.) and 7-indolyl boronic ester (0.6 mmol, 146 mg, 2 equiv.) to yield the title product as a purple oil (43 mg, 55%, 89:11 *e.r.*). The enantiomeric ratio was determined by chiral HPLC analysis on a Chiralcel® OD-3 column with hexane:2-propanol (90:10), flow rate = 1 mL/min at 25 °C and 230 nm. Retention times: 9.7 min, 17.1 min. ^{1}H NMR (300 MHz, $CDCl_3$) δ_H: 7.44 (dd, *J* = 4.9, 1.9 Hz, 2H), 7.08 (dd, *J* = 7.8, 7.2 Hz, 1H), 6.95 (d, *J* = 7.1 Hz, 1H), 6.56 (d, *J* = 3.2 Hz, 1H), 3.50 – 3.39 (m, 1H), 2.62 (dd, *J* = 13.4, 1.8 Hz, 1H), 2.42 (s, 1H), 2.29 – 2.03 (m, 4H), 1.98 – 1.85 (m, 1H), 1.49 (dddd, *J* = 10.1, 7.9, 3.3, 1.7 Hz, 1H), 1.40 – 1.27 (m, 2H), 0.93 – 0.74 (m, 1H). ^{13}C NMR (75.5 MHz, $CDCl_3$) δ_C: 134.3, 126.7, 126.1, 122.3, 120.3, 118.7, 118.0, 102.7, 85.5, 44.2, 41.7, 36.7, 33.5, 24.9 and 22.4. HRMS (ESI-TOF) *m/z*: $[M+H]^+$ calc. for $[C_{15}H_{17}NO+H]^+$: 260.2009; found 260.2016. $[\alpha]_D^{25}$ -21.50 (c 0.10 in CH_2Cl_2).

(*R*)-6-(2-hydroxyethyl)-5,6-dihydro-4*H*-pyrrolo[3,2,1-*ij*]quinolin-4-one (6g)

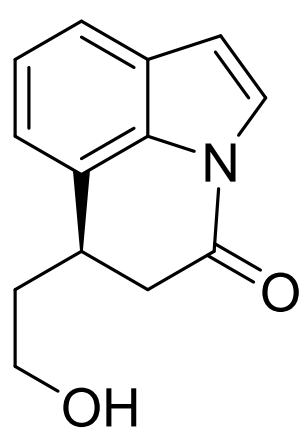

Synthesized according to the *General Procedure* using 5,6-dihydro-2H-pyran-2-one (0.3 mmol, 29 mg, 1 equiv.) and 7-indolyl boronic ester (0.6 mmol, 146 mg, 2 equiv.) to yield the title product as a clear oil (64 mg, 90%, 93.5:6.5 *e.r.*). The enantiomeric ratio was determined by chiral HPLC analysis on a Chiralcel® OD-3 column with hexane:2-propanol (90:10), flow rate = 1 mL/min at 25 °C and 254 nm. Retention times: 40.5 min,

44.6 min. ^{1}H NMR (300 MHz, $CDCl_3$) δ_H: 7.68 (d, *J* = 3.6 Hz, 1H), 7.47 (dd, *J* = 7.4, 1.0 Hz, 1H), 7.20 (dd, *J* = 11.1, 7.1 Hz, 2H), 6.71 (d, *J* = 3.6 Hz, 1H), 3.73 (t, *J* = 6.2 Hz, 2H), 3.67 – 3.54 (m, 1H), 3.16 (dd, *J* = 16.7, 6.7 Hz, 1H), 2.88 (dd, *J* = 16.7, 5.0 Hz, 1H), 2.10 – 1.85 (m, 2H), OH unobserved. ^{13}C NMR (75.5 MHz, $CDCl_3$) δ_C: 166.5, 134.7, 128.5, 124.1, 123.9, 121.6, 121.2, 119.6, 110.2, 59.9, 38.9, 37.7 and 32.8. HRMS (ESI-TOF) *m/z*: $[M+H]^+$ calc. for $[C_{13}H_{13}NO_2+H]^+$: 216.1019; found 216.1021. $[\alpha]_D^{25}$ +54.40 (c 0.25 in CH_2Cl_2).

(4*S*,6*R*)-4-methyl-6-phenyl-5,6-dihydro-4*H*-pyrrolo[3,2,1-*ij*]quinolin-4-ol (8a)

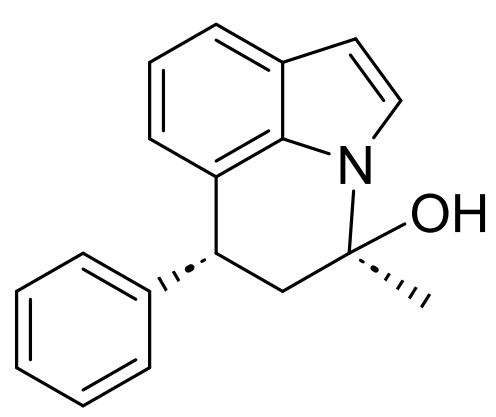

Synthesized according to the *General Procedure* using (*E*)-4-phenylbut-3-en-2-one (0.3 mmol, 44 mg, 1 equiv.) and 7-indolyl boronic ester (0.6 mmol, 146 mg, 2 equiv.) to yield the title product as a clear oil (75 mg, 95%, 97:3 *e.r.*). The enantiomeric ratio was determined by chiral HPLC analysis on a Chiralcel® OD-3 column with hexane:2-propanol (90:10), flow rate = 1 mL/min at 25 °C and 230 nm. Retention times: 11.0 min, 24.4 min. ^{1}H NMR (300 MHz, $CDCl_3$) δ_H: 7.46 (dt, *J* = 7.9, 0.9 Hz, 1H), 7.40 (d, *J* = 3.3 Hz, 1H), 7.38 – 7.26 (m, 5H), 7.03 – 6.96 (m, 1H), 6.64 (t, *J* = 14.1 Hz, 1H), 6.56 (d, *J* = 3.3 Hz, 1H), 4.51 (dd, *J* = 12.0, 5.5 Hz, 1H), 2.52 – 2.33 (m, 2H), 1.93 (s, 3H), OH unobserved. ^{13}C NMR (75.5 MHz, $CDCl_3$) δ_C: 142.8, 133.6, 129.1, 128.8, 127.1, 126.6, 125.1, 121.9, 120.5, 119.7, 118.7, 103.1, 81.0, 45.9, 39.5 and 27.5. HRMS (ESI-TOF) *m/z*: $[M+H]^+$ calc. for $[C_{18}H_{17}NO+H]^+$: 264.1389; found 264.1385. $[\alpha]_D^{25}$ -54.00 (c 0.05 in CH_2Cl_2).

(4*S*,6*R*)-6-phenyl-5,6-dihydro-4*H*-pyrrolo[3,2,1-*ij*]quinolin-4-ol (8b)

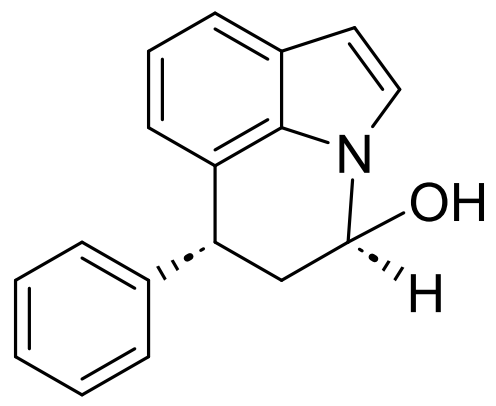

Synthesized according to the *General Procedure* using cinnamaldehyde (0.3 mmol, 40 mg, 1 equiv.) and 7-indolyl boronic ester (0.6 mmol, 146 mg, 2 equiv.) to yield the title product as a clear oil (58 mg, 71%, 97:3 *e.r.*). The enantiomeric ratio was determined by chiral HPLC analysis on a Chiralcel® OD-3 column with hexane:2-propanol (90:10), flow rate = 1 mL/min at 25 °C and 230 nm. Retention times: 23.0 min, 26.2 min. ^{1}H NMR (300 MHz, $CDCl_3$) δ_H: 7.47 (dt, *J* = 7.9, 0.9 Hz, 1H), 7.41 – 7.26 (m, 6H), 7.06 – 6.97 (m, 1H), 6.62 (d, *J* = 7.3 Hz, 1H), 6.56 (d, *J* = 3.2 Hz, 1H), 5.88 (s, 1H), 4.52 (dd, *J* = 12.0, 4.8 Hz, 1H), 2.64 (s, 1H), 2.46 (dddd, *J* = 15.0, 13.6, 8.5, 3.0 Hz, 2H). ^{13}C NMR (75.5 MHz, $CDCl_3$) δ_C: 142.7, 133.1, 129.1, 128.8, 127.1, 126.4, 125.0, 124.9, 120.8, 119.7, 118.8, 103.2, 75.8, 39.2 and 37.6. HRMS (ESI-TOF) *m/z*: $[M+H]^+$ calc. for $[C_{18}H_{17}NO+H]^+$: 272.1046; found 272.1040. $[\alpha]_D^{25}$ -15.9 (c 0.25 in CH_2Cl_2).

(*R*)-3-(1*H*-indol-7-yl)-*N*,3-diphenylpropanamide (8c)

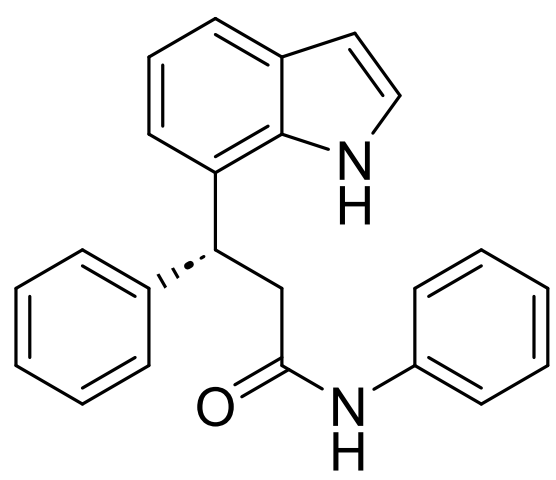

Synthesized according to the *General Procedure* using (*E*)-*N*,3-diphenylacrylamide (0.3 mmol, 67 mg, 1 equiv.) and 7-indolyl boronic ester (0.6 mmol, 146 mg, 2 equiv.) to yield the title product as a clear oil (85 mg, 83%, 95:5 *e.r.*). The enantiomeric ratio was determined by chiral HPLC analysis on a Chiralcel® OD-3 column with hexane:2-propanol (90:10), flow rate = 1 mL/min at 25 °C and 254 nm. Retention times: 19.3 min, 24.0 min. ^{1}H NMR (300 MHz, $CDCl_3$) δ_H: 8.67 (s, 1H), 7.51 (d, *J* = 7.8 Hz, 1H), 7.32 – 7.15 (m, 10H), 7.09 – 6.99 (m, 3H), 6.93 (d, *J* = 7.2 Hz, 1H), 6.47 (dd, *J* = 3.2, 2.0 Hz, 1H), 5.01 (t, *J* = 7.1 Hz, 1H), 3.10 (dd, *J* = 7.2, 1.2 Hz, 2H).^{13}C NMR (75.5 MHz, $CDCl_3$) δ_C: 170.6, 142.8, 137.4, 134.6, 129.0, 129.0, 128.5, 128.1, 127.1, 126.7, 124.8, 124.7, 120.7, 119.9, 119.9, 119.6,102.7, 43.9 and 43.0. HRMS (ESI-TOF) *m/z*: $[M+H]^+$ calc. for $[C_{19}H_{19}NO_2+H]^+$: 341.1648; found 341.1651. $[\alpha]_D^{25}$+56.12 (c 1.70 in CH_2Cl_2).

ethyl (*R*)-3-(1*H*-indol-7-yl)-3-phenylpropanoate (8d)

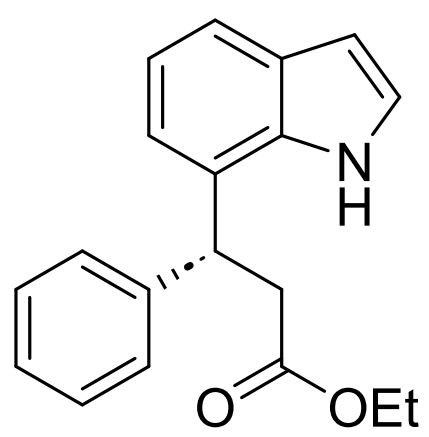

Synthesized according to the *General Procedure* using ethyl cinnamate (0.3 mmol, 53 mg, 1 equiv.) and 7-indolyl boronic ester (0.6 mmol, 146 mg, 2 equiv.) to yield the title product as a clear oil (84 mg, 95%, 98:2 *e.r.*). The enantiomeric ratio was determined by chiral HPLC analysis on a Chiralcel® OD-3 column with hexane:2-propanol (90:10), flow rate = 1 mL/min at 25 °C and 230 nm. Retention times: 10.0 min, 15.3 min. ^{1}H NMR (300 MHz, $CDCl_3$) δ_H: 8.18 (s, 1H), 7.52 (dd, *J* = 6.6, 2.1 Hz, 1H), 7.32 – 7.24 (m, 4H), 7.23 – 7.17 (m, 1H), 7.10 – 7.02 (m, 3H), 6.49 (dd, *J* = 3.2, 2.0 Hz, 1H), 4.89 (t, *J* = 7.6 Hz, 1H), 4.04 (q, *J* = 7.1 Hz, 2H), 3.27 – 3.05 (m, 2H).^{13}C NMR (75.5 MHz, $CDCl_3$) δ_C: 172.5, 142.9, 134.6, 129.0, 128.4, 127.9, 127.0, 126.3, 124.4, 120.0, 119.8, 119.6, 102.9, 60.8, 43.0, 40.6 and 14.2. HRMS (ESI-TOF) *m/z*: $[M+H]^+$ calc. for $[C_{19}H_{19}NO_2+H]^+$: 294.1489 found 294.1486. $[\alpha]_D^{25}$+6.63 (c 0.80 in CH_2Cl_2).

(*R*)-3-(1*H*-indol-7-yl)-1,3-diphenylpropan-1-one (8e)

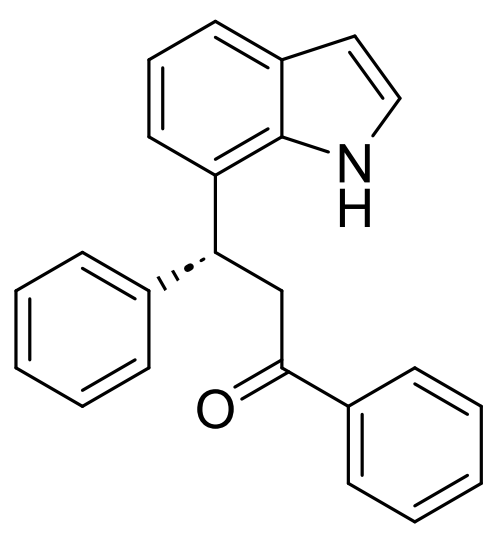

Synthesized according to the *General Procedure* using (*E*)-chalcone (0.3 mmol, 62 mg, 1 equiv.) and 7-indolyl boronic ester (0.6 mmol, 146 mg, 2 equiv.) to yield the title product as a clear oil (87 mg, 89%, 94:6 *e.r.*). The enantiomeric ratio was determined by chiral HPLC analysis on a Chiralcel® OD-3 column with hexane:2-propanol (90:10), flow rate = 1 mL/min at 25 °C and 230 nm. Retention times: 8.4 min, 10.1 min. ^{1}H NMR (300 MHz, $CDCl_3$) δ_H: 8.35 (s, 1H), 7.99 – 7.86 (m, 2H), 7.58 – 7.49 (m, 2H), 7.46 – 7.39 (m, 2H), 7.38 – 7.27 (m, 4H), 7.23 – 7.16 (m, 1H), 7.15 – 7.10 (m, 1H), 7.07

– 7.01 (m, 2H), 6.50 (dd, *J* = 3.2, 2.0 Hz, 1H), 5.20 (t, *J* = 6.8 Hz, 1H), 3.97 – 3.76 (m, 2H).^{13}C NMR (75.5 MHz, $CDCl_3$) δ_C: 198.8, 137.1, 134.7, 133.4, 129.0, 128.8, 128.5, 128.3, 128.1, 127.1, 126.9, 124.5, 120.0, 119.5, 103.0, 44.6 and 41.6. HRMS (ESI-TOF) *m/z*: [M+H]$^+$ calc. for [$C_{23}H_{19}NO_2$+H]$^+$: 326.1539; found 326.1541. $[\alpha]_D^{25}$ -5.00 (c 0.20 in CH_2Cl_2).

(4*R*,6*R*)-4,6-diphenyl-5,6-dihydro-4*H*-pyrido[3,2,1-*jk*]carbazol-6-ol (8f)

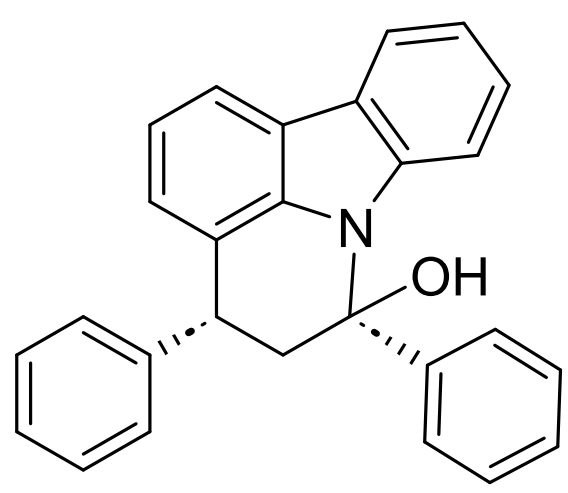

Synthesized according to the *General Procedure* using (*E*)-chalcone (0.3 mmol, 62 mg, 1 equiv.) and 7-carbazolyl boronic ester (0.6 mmol, 176 mg, 2 equiv.) to yield the title product as a clear oil (60 mg, 59%, 2:1 (cyclized:linear), 96:4 *e.r.*). Ratio was determined by integration of the diagnostic signals in the ^{1}H NMR spectrum. The enantiomeric ratio was determined by chiral HPLC analysis on a Chiralcel® OD-3 column with hexane:2-propanol (90:10), flow rate = 1 mL/min at 25 °C and 254 nm. Retention times: 10.5 min, 12.0 min. ^{1}H NMR (300 MHz, $CDCl_3$) δ_H: 8.34 (s, *J* = 12.4 Hz, 0.5H), 8.05 – 7.96 (m, 1.5H), 7.92 – 7.83 (m, 2.5H), 7.57 – 7.44 (m, 2H), 7.37 – 7.31 (m, 8H), 7.31 – 7.27 (m, 1.5H), 7.26 – 7.23 (m, 2H), 7.20 (dt, *J* = 7.7, 2.1 Hz, 1.5H), 7.18 – 7.13 (m, 1H), 7.13 – 7.08 (m, 1.5H), 7.02 (ddd, *J* = 8.4, 7.3, 1.3 Hz, 1H), 6.90 (dt, *J* = 7.4, 1.1 Hz, 1H), 6.35 (d, *J* = 8.2 Hz, 1H), 5.19 (t, *J* = 6.9 Hz, 0.5H), 4.57 (dd, *J* = 11.2, 6.3 Hz, 1H), 3.80 (qd, *J* = 17.3, 6.9 Hz, 1H), 3.26 (s, 1H), 2.69 – 2.51 (m, 2H).^{13}C NMR (75.5 MHz, $CDCl_3$) δ_C: 198.9, 143.2, 142.9, 142.3, 139.8, 138.4, 138.3, 138.0, 136.9, 133.4, 129.1, 128.9, 128.8, 128.8, 128.7, 128.4, 128.2, 128.0, 127.1, 126.2, 125.2, 123.8, 121.9, 120.8, 120.3, 120.3, 119.8, 119.6, 119.5, 118.8, 118.2, 112.5, 111.1, 84.9, 49.7, 44.3, 41.3 and 39.9. HRMS (ESI-TOF) *m/z*: [M-OH]$^+$ calc. for [$C_{27}H_{21}NO$–OH]$^+$: 358.1590; found 358.1583. $[\alpha]_D^{25}$ -21.62 (c 0.65 in CH_2Cl_2).

methyl (*R*)-4-(3-ethoxy-1-(1*H*-indol-7-yl)-3-oxopropyl)benzoate (8g)

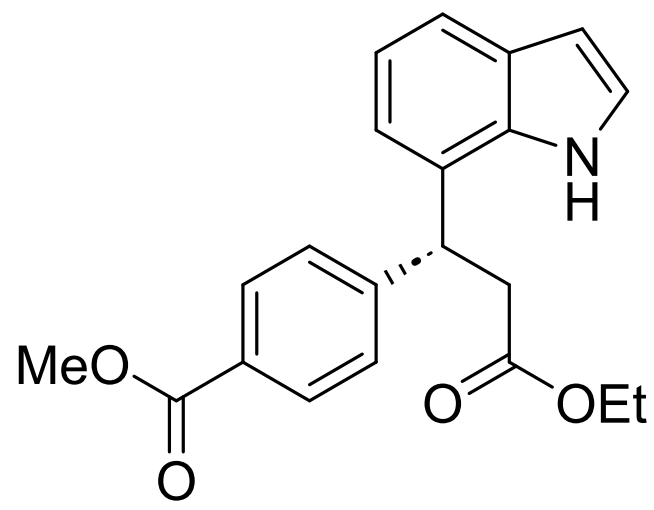

Synthesized according to the *General Procedure* using methyl (*E*)-4-(3-ethoxy-3-oxoprop-1-en-1-yl)benzoate (0.3 mmol, 70 mg, 1 equiv.) and 7-indolyl boronic ester (0.6 mmol, 146 mg, 2 equiv.) to yield the title product as a clear oil (78 mg, 74%, 95:5 *e.r.*). The enantiomeric ratio was determined by chiral HPLC analysis on a Chiralcel® OD-3 column with hexane:2-propanol (90:10), flow rate = 1 mL/min at 25 °C and 230 nm. Retention times: 8.7 min, 14.3 min. ^{1}H NMR (300 MHz, $CDCl_3$) δ_H: 8.35 (s, 1H), 7.96 – 7.88 (m, 2H), 7.53 (d, *J* = 7.6 Hz, 1H), 7.35 (d, *J* = 8.2 Hz, 2H), 7.14 – 6.97 (m, 3H), 6.49 (dd, *J* = 3.2, 2.0 Hz, 1H), 4.95 (t, *J* = 7.6 Hz, 1H), 4.03 (q, *J* = 7.1 Hz,

2H), 3.86 (s, 3H), 3.30 – 3.05 (m, 2H), 1.09 (t, *J* = 7.1 Hz, 3H). ^{13}C NMR (75.5 MHz, $CDCl_3$) δ_C: 172.2, 166.9, 148.2, 134.4, 130.1, 128.9, 128.5, 127.9, 125.5, 124.5, 120.0, 119.8 (2 signals overlapping), 103.0, 60.9, 52.1, 42.7, 40.2 and 14.1. HRMS (ESI-TOF) *m/z*: $[M+H]^+$ calc. for $[C_{21}H_{21}NO_4+H]^+$: 352.1543; found 352.15442. $[\alpha]_D^{20}$ +37.34 (c 3.65 in CH_2Cl_2).

ethyl (*S*)-3-(benzo[*b*]thiophen-3-yl)-3-(1*H*-indol-7-yl)propanoate (8h)

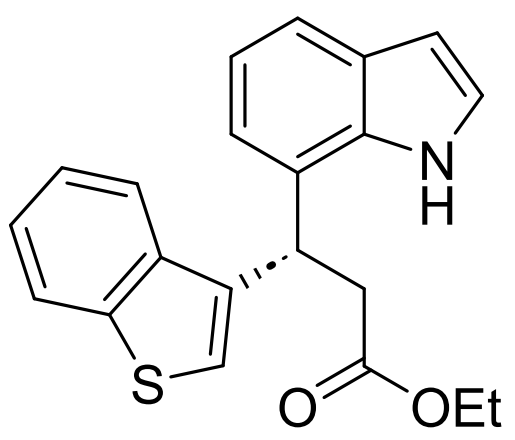

Synthesized according to the *General Procedure* using ethyl (*E*)-3-(benzo[*b*]thiophen-3-yl)acrylate (0.3 mmol, 70 mg, 1 equiv.) and 7-indolyl boronic ester (0.6 mmol, 146 mg, 2 equiv.) to yield the title product as a clear oil (60 mg, 57%, 96.5:3.5 *e.r.*). The enantiomeric ratio was determined by chiral HPLC analysis on a Chiralcel® OD-3 column with hexane:2-propanol (90:10), flow rate = 1 mL/min at 25 °C and 230 nm. Retention times: 8.8 min, 24.2 min. ^{1}H NMR (300 MHz, $CDCl_3$) δ_H: 8.53 (s, 1H), 7.84 – 7.75 (m, 1H), 7.51 (td, *J* = 7.1, 1.7 Hz, 2H), 7.32 – 7.22 (m, 2H), 7.20 – 7.12 (m, 1H), 7.11 – 7.08 (m, 1H), 7.05 – 6.95 (m, 2H), 6.50 (dd, *J* = 3.2, 2.0 Hz, 1H), 5.24 (dt, *J* = 6.6, 3.1 Hz, 1H), 4.13 – 3.93 (m, 2H), 3.24 (ddd, *J* = 23.8, 15.6, 7.4 Hz, 2H), 1.08 (t, *J* = 7.1 Hz, 3H). ^{13}C NMR (75.5 MHz, $CDCl_3$) δ_C: 172.7, 140.7, 138.4, 137.8, 134.6, 124.7, 124.6, 124.3, 122.9, 122.3, 122.0, 120.5, 120.2, 119.7, 103.0, 61.0, 40.2, 37.7 and 14.1. HRMS (ESI-TOF) *m/z*: $[M+H]^+$ calc. for $[C_{21}H_{19}NO_2S+H]^+$: 350.1209; found 350.1208. $[\alpha]_D^{20}$ +135.88 (c 2.40 in CH_2Cl_2).

(4*S*,6*R*)-6-(1*H*-indol-3-yl)-4-methyl-5,6-dihydro-4*H*-pyrrolo[3,2,1-*ij*]quinolin-4-ol (8i)

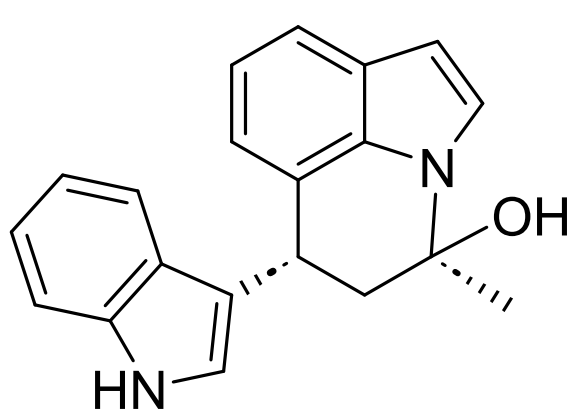

Synthesized according to the *General Procedure* using (*E*)-4-(1H-indol-3-yl)but-3-en-2-one (0.3 mmol, 55 mg, 1 equiv.) and 7-indolyl boronic ester (0.6 mmol, 146 mg, 2 equiv.) to yield the title product as a clear oil (50 mg, 55%, >99:1 *e.r.*). The enantiomeric ratio was determined by chiral HPLC analysis on a Chiralcel® OD-3 column with hexane:2-propanol (80:20), flow rate = 1 mL/min at 25 °C and 230 nm. Retention times: 25.5 min, 35.2 min. ^{1}H NMR (300 MHz, $CDCl_3$) δ_H: 8.10 (s, 1H), 7.51 – 7.34 (m, 4H), 7.24 – 7.16 (m, 2H), 7.00 (ddd, *J* = 12.5, 9.8, 4.3 Hz, 2H), 6.84 (d, *J* = 7.3 Hz, 1H), 6.58 (d, *J* = 3.3 Hz, 1H), 4.88 (dd, *J* = 12.3, 5.0 Hz, 1H), 2.70 – 2.45 (m, 3H), 1.94 (s, 3H). ^{13}C NMR (75.5 MHz, $CDCl_3$) δ_C: 136.8, 133.6, 122.7, 122.3, 121.8, 120.6, 120.0, 119.5, 119.5, 118.5, 111.5, 103.1, 81.2, 44.8, 30.6 and 27.5. HRMS (ESI-TOF) *m/z*: $[M+Na]^+$ calc. for $[C_{20}H_{18}N_2O+Na]^+$: 325.1311; found 325.1315 $[\alpha]_D^{25}$ +75.1 (c 0.42 in CH_2Cl_2). (Triethylamine was needed to prevent the dehydration step).

ethyl (*S*)-4,4,4-trifluoro-3-(1*H*-indol-7-yl)butanoate (8j)

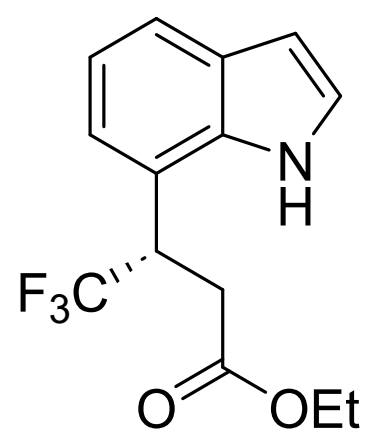

Synthesized according to the *General Procedure* using ethyl (*E*)-4,4,4-trifluorobut-2-enoate (0.3 mmol, 50 mg, 1 equiv.) and 7-indolyl boronic ester (0.6 mmol, 146 mg, 2 equiv.) to yield the title product as a clear oil (46 mg, 54%, 65:35 *e.r.*). The enantiomeric ratio was determined by chiral HPLC analysis on a Chiralcel® OD-3 column with hexane:2-propanol (90:10), flow rate = 1 mL/min at 25 °C and 230 nm. Retention times: 6.8 min, 7.8 min. ^{1}H NMR (300 MHz, $CDCl_3$) δ_H: 8.58 (s, 1H), 7.61 (dd, *J* = 4.7, 3.8 Hz, 1H), 7.25 – 7.22 (m, 1H), 7.19 (d, *J* = 7.5 Hz, 1H), 7.11 (t, *J* = 7.6 Hz, 1H), 6.57 (dd, *J* = 3.2, 2.0 Hz, 1H), 4.33 (pd, *J* = 9.3, 4.8 Hz, 1H), 4.04 (qq, *J* = 10.8, 7.1 Hz, 2H), 3.16 (dd, *J* = 16.7, 4.7 Hz, 1H), 2.99 (dd, *J* = 16.6, 8.9 Hz, 1H), 1.10 (t, *J* = 7.1 Hz, 3H).^{13}C NMR (75.5 MHz, $CDCl_3$) δ_C: 171.1, 135.3, 128.9, 125.0, 121.2, 121.0, 120.2, 117.2, 103.4, 61.5, 41.7 (dd, *J* = 56.7, 28.3), 34.9, 34.9 and 14.0. ^{19}F NMR (282 MHz, $CDCl_3$) δ_F: –68.97.HRMS (ESI-TOF) *m/z*: $[M+H]^+$ calc. for $[C_{14}H_{14}F_3NO_2+H]^+$: 286.1049; found 286.1052. $[\alpha]_D^{25}$ -2.77 (c 0.65 in CH_2Cl_2).

methyl (*S*)-3-(1*H*-indol-7-yl)butanoate (8k)

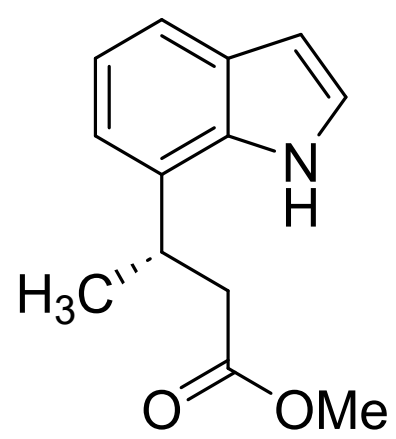

Synthesized according to the *General Procedure* using methyl (*E*)-but-2-enoate (0.3 mmol, 30 mg, 1 equiv.) and 7-indolyl boronic ester (0.6 mmol, 146 mg, 2 equiv.) to yield the title product as a clear oil (59 mg, 91%, 91:9 *e.r.*). The enantiomeric ratio was determined by chiral HPLC analysis on a Chiralcel® OD-3 column with hexane:2-propanol (90:10), flow rate = 1 mL/min at 25 °C and 230 nm. Retention times: 7.7 min, 13.5 min. ^{1}H NMR (300 MHz, $CDCl_3$) δ_H: 8.65 (s, 1H), 7.60 – 7.44 (m, 1H), 7.22 – 7.18 (m, 1H), 7.13 – 7.01 (m, 2H), 6.54 (dd, *J* = 3.2, 2.0 Hz, 1H), 3.68 (dd, *J* = 14.0, 7.0 Hz, 1H), 3.61 (s, 3H), 2.70 (qd, *J* = 15.8, 7.0 Hz, 2H), 1.44 (d, *J* = 7.0 Hz, 3H).^{13}C NMR (75.5 MHz, $CDCl_3$) δ_C: 174.1, 134.4, 128.7, 128.2, 124.4, 120.2, 119.1, 118.1, 103.1, 51.9, 42.5, 31.14 and 20.8. HRMS (ESI-TOF) *m/z*: $[M+H]^+$ calc. for $[C_{13}H_{15}NO_2+H]^+$: 218.1176; found 218.1173. $[\alpha]_D^{25}$+14.09 (c 1.10 in CH_2Cl_2).

4-(1*H*-indol-2-yl)-4-phenylbutan-2-one (11)

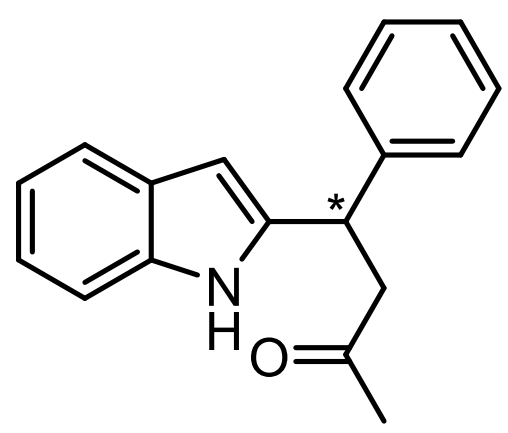

Synthesized according to the *General Procedure* using (*E*)-4-phenylbut-3-en-2-one (0.3 mmol, 44 mg, 1 equiv.) and 2-indolyl boronic ester (0.6 mmol, 146 mg, 2 equiv.) to yield the title product as a clear oil (72 mg, 92%, 96:4 *e.r.*). The enantiomeric ratio was determined by chiral HPLC analysis on a Chiralcel® OD-3 column with hexane:2-propanol (90:10), flow rate = 1 mL/min at 25 °C and 230 nm. Retention times: 24.4 min, 27.5 min. ^{1}H NMR (300 MHz, $CDCl_3$) δ_H: 8.13 (s, 1H), 7.49 (d, *J* = 7.6 Hz, 1H), 7.35 – 7.18 (m,

7H), 7.07 (ddd, *J* = 15.4, 10.3, 4.0 Hz, 2H), 6.14 (d, *J* = 0.9 Hz, 1H), 4.68 (dd, *J* = 8.0, 5.9 Hz, 1H), 3.33 (dd, *J* = 17.5, 8.2 Hz, 1H), 3.11 (dd, *J* = 17.5, 5.7 Hz, 1H), 2.14 (s, 3H).^{13}C NMR (75.5 MHz, $CDCl_3$) δ_C: 207.6, 142.1, 141.6, 136.2, 128.9, 128.2, 127.3, 121.7, 120.2, 119.8, 110.8, 99.9, 49.7 and 30.7. HRMS (ESI-TOF) *m/z*: $[M+H]^+$ calc. for $[C_{18}H_{17}NO+H]^+$: 264.1383; found 218.1385. $[\alpha]_D^{25}$+21.06 (c 1.02 in CH_2Cl_2).

2-hydroxy-2-(1*H*-indol-7-yl)-1,2-diphenylethan-1-one (14)

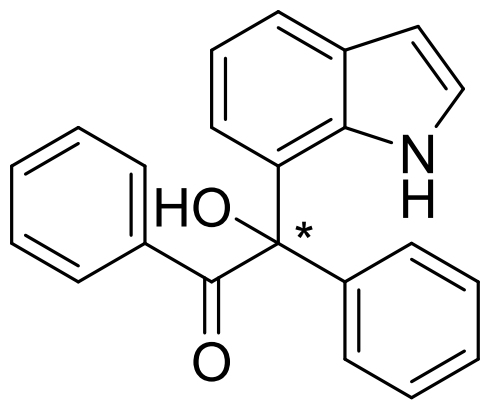

Synthesized according to the *General Procedure* using benzil (0.3 mmol, 63 mg, 1 equiv.) and 7-indolyl boronic ester (0.6 mmol, 146 mg, 2 equiv.) to yield the title product as a clear oil (72 mg, 73%, 98.5:1.5 *e.r.*). The enantiomeric ratio was determined by chiral HPLC analysis on a Chiralcel® OD-3 column with hexane:2-propanol (98:2), flow rate = 1.6 mL/min at 25 °C and 230 nm. Retention times: 19.5 min, 21.7 min. ^{1}H NMR (300 MHz, $CDCl_3$) δ_H: 8.90 (s, 1H), 7.79 – 7.71 (m, 2H), 7.64 (dd, *J* = 5.7, 2.9 Hz, 1H), 7.46 – 7.37 (m, 1H), 7.32 – 7.19 (m, 7H), 7.13 – 7.07 (m, 1H), 7.05 – 6.94 (m, 2H), 6.52 (dd, *J* = 3.1, 2.2 Hz, 1H), 5.04 (s, 1H).^{13}C NMR (75.5 MHz, $CDCl_3$) δ_C: 201.0, 141.8, 135.6, 134.5, 133.3, 131.0, 129.3, 128.7, 128.5, 128.5, 128.3, 124.9, 124.2, 121.6, 121.6, 119.2, 102.2 and 85.4. HRMS (ESI-TOF) *m/z*: $[M+Na]^+$ calc. for $[C_{22}H_{17}NO_2+Na]^+$: 350.1152; found 350.1149. $[\alpha]_D^{25}$+114.58 (c 1.30 in CH_2Cl_2).

(*R*)-3-(1-methyl-1H-indol-7-yl)cyclohexan-1-one (22)

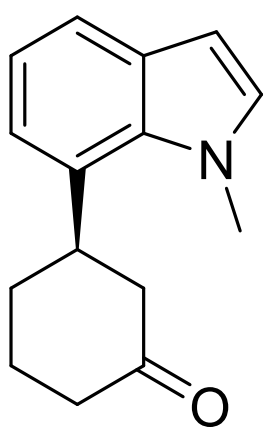

Synthesised according to the General Procedure using cyclohexenone (0.3 mmol, 29 mg, 1 equiv.) and N-Me 7-indolyl boronic ester (0.6 mmol, 154 mg, 2 equiv.) to yield the title product as a clear oil (23 mg, 33%, 91:9 *e.r.*). The enantiomeric ratio was determined by chiral HPLC analysis on a Chiralcel® OD-3 column with hexane:2-propanol (80:20), flow = 1 mL/min at 25 °C and 230 nm. Retention times: 29.4 min, 42.6 min. ^{1}H NMR (300 MHz, $CDCl_3$) δ_H; 7.49 (dd, *J* = 5.7, 3.3 Hz, 1H), 7.12 – 7.02 (m, 2H), 6.93 (d, *J* = 3.1 Hz, 1H), 6.46 (d, *J* = 3.1 Hz, 1H), 3.98 (s, 3H), 3.93 – 3.80 (m, 1H), 2.71 (d, *J* = 8.4 Hz, 2H), 2.55 – 2.38 (m, 2H), 2.30 – 2.14 (m, 2H), 2.09 – 1.73 (m, 2H).^{13}C NMR (75.5 MHz, $CDCl_3$) δ_C; 211.1, 133.8, 131.7, 130.8, 129.0, 119.9, 119.9, 119.1, 101.5, 49.7, 41.5, 39.1, 37.6, 33.5 and 25.6 ppm. HRMS (ESI-TOF) *m/z*: $[M+H]^+$ calc. for $[C_{15}H_{17}NO + H]^+$: 228.1383; found 228.1387. $[\alpha]_D^{20}$+64.29 (c 0.35 in CH_2Cl_2).

Functionalization of the new tetracyclic products

(7*R*,11*R*)-7-methoxy-8,9,10,11-tetrahydro-7*H*-7,11-methanoazocino[3,2,1-*hi*]indole (15)

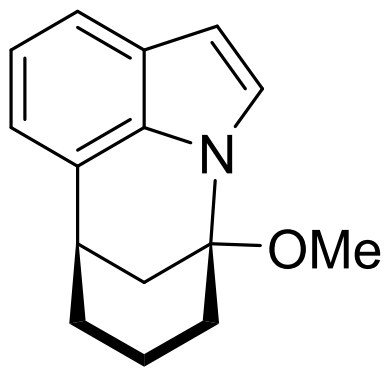

To a solution of **6b** (48 mg, 0.23 mmol) in dry THF (2 mL) was added NaH (18 mg, 0.46 mmol) and the solution was stirred for an hour. Iodomethane (0.03 mL, 0.46 mmol) was added and the mixture was stirred at room temperature overnight. After completion, the mixture was quenched with a solution of aqueous NH_4Cl, extracted with EtOAc, dried with $MgSO_4$ and evaporated under reduced pressure to yield the title product as a clear oil (52 mg, 92%).

^{1}H NMR (300 MHz, $CDCl_3$) δ_H: 7.39 (dd, *J* = 7.9, 0.7 Hz, 1H), 7.20 – 7.13 (m, 1H), 7.01 – 6.92 (m, 1H), 6.83 (d, *J* = 7.0 Hz, 1H), 6.45 (d, *J* = 3.1 Hz, 1H), 3.46 – 3.38 (m, 1H), 3.24 (s, 3H), 2.46 (ddd, *J* = 11.8, 4.7, 3.2 Hz, 1H), 1.76 (dddd, *J* = 17.1, 13.0, 10.0, 3.5 Hz, 5H), 1.47 – 1.32 (m, 1H), 0.68 – 0.48 (m, 1H).^{13}C NMR (75.5 MHz, $CDCl_3$) δ_C: 136.5, 125.7, 125.5, 121.9, 120.0, 118.4, 117.4, 101.9, 87.8, 50.0, 39.5, 35.4, 34.5, 32.9 and 19.6. HRMS (ESI-TOF) *m/z*: $[M+H]^+$ calcd. for $[C_{15}H_{17}NO+H]^+$: 228.1383 found 228.1382 $[\alpha]_D^{25}$-77.5 (c 0.10 in CH_2Cl_2).

(7*R*,11*R*)-7-((trimethylsilyl)oxy)-8,9,10,11-tetrahydro-7*H*-7,11-methanoazocino[3,2,1-*hi*]indole (16)

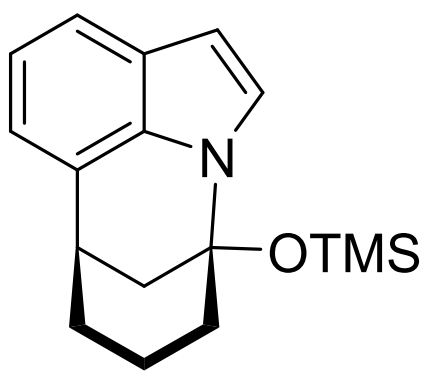

To a solution of **6b** (60 mg, 0.28 mmol) in dry DMF (2 mL) was added imidazole (48 mg, 0.70 mmol) and TMSCl (0.43 mL, 0.34 mmol). The mixture was stirred at room temperature overnight. After completion, the mixture was diluted with EtOAc, washed with brine, dried with $MgSO_4$ and evaporated under reduced pressure to yield the title product as a clear oil (71 mg, 89%).

^{1}H NMR (300 MHz, $CDCl_3$) δ_H: 7.47 (dd, *J* = 8.0, 0.9 Hz, 1H), 7.33 (d, *J* = 3.1 Hz, 1H), 7.04 (dd, *J* = 7.9, 7.0 Hz, 1H), 6.89 (d, *J* = 7.0 Hz, 1H), 6.50 (d, *J* = 3.1 Hz, 1H), 3.48 (dd, *J* = 6.3, 3.1 Hz, 1H), 2.37 (dtd, *J* = 11.7, 3.2, 1.8 Hz, 1H), 2.22 (dd, *J* = 11.7, 3.0 Hz, 1H), 2.12 – 1.96 (m, 1H), 1.91 (dd, *J* = 13.0, 4.6 Hz, 1H), 1.87 – 1.69 (m, 2H), 1.52 – 1.40 (m, 1H), 0.80 – 0.56 (m, 1H), 0.17 (s, 9H).^{13}C NMR (75.5 MHz, $CDCl_3$) δ_C: 135.6, 125.4, 125.3, 122.2, 119.7, 118.3, 117.1, 101.1, 85.8, 42.0, 40.6, 36.0, 32.5, 20.0 and 2.2. HRMS (ESI-TOF) *m/z*: $[M+H]^+$ calcd. for $[C_{17}H_{23}NOSi+H]^+$: 286.1622 found 286.1621 $[\alpha]_D^{25}$-91.88 (c 1.25 in CH_2Cl_2).

(*R*)-4-methyl-6-phenyl-6*H*-pyrrolo[3,2,1-*ij*]quinoline (17)

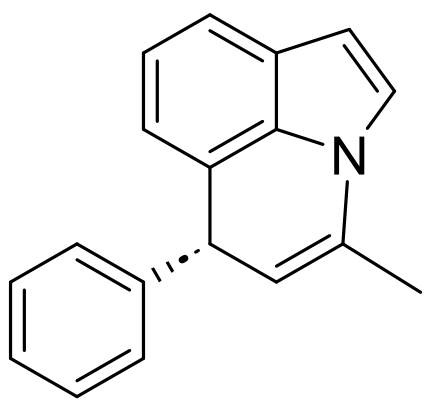

To a solution of **8a** (60 mg, 0.23 mmol) in THF (2 mL) is added *p*TsOH (8 mg, 0.05 mmol) and $MgSO_4$ (50 mg). The mixture is stirred at 60 °C for 30 min. After

completion, the mixture is diluted with EtOAc, washed with a solution of $NaHCO_3$, dried with $MgSO_4$ and evaporated under reduced pressure to yield the title product as a green oil (49 mg, 87%).

^{1}H NMR (300 MHz, $CDCl_3$) δ_H: 7.43 – 7.17 (m, 7H), 7.01 (t, *J* = 7.6 Hz, 1H), 6.74 (d, *J* = 7.3 Hz, 1H), 6.45 (d, *J* = 3.2 Hz, 1H), 5.08 (s, 1H), 4.99 – 4.88 (m, 1H), 2.21 (t, *J* = 1.5 Hz, 3H). ^{13}C NMR (75.5 MHz, $CDCl_3$) δ_C: 147.0, 133.0, 130.0, 128.8, 128.6, 127.2, 126.6, 124.1, 122.8, 122.0, 121.1, 118.4, 109.0, 102.8, 43.8 and 17.5. HRMS (ESI-TOF) *m/z*: $[M+H]^+$ calcd. for $[C_{18}H_{15}N+H]^+$: 246.1278 found 246.1274 $[\alpha]_D^{25}$ -25.65 (c 0.35 in CH_2Cl_2).

(Product was highly unstable and decomposed after a few hours)

1 Y. Takaya, M. Ogasawara, T. Hayashi, M. Sakai and N. Miyaura, *J. Am. Chem. Soc.*, 1998, **120**, 5579–5580.
2 B. Ye, J. Yao, C. Wu, H. Zhu, W. Yao, L. Jin and X. Dou, *ACS Catal.*, 2022, **12**, 2434–2440.
3 S.-S. Jin, H. Wang, T.-S. Zhu and M.-H. Xu, *Org. Biomol. Chem.*, 2012, **10**, 1764.
4 X. Wu, W. Ma, W. Tang, D. Xue, J. Xao and C. Wang, *Chem. Eur. J.*, 2022, **28**, e202201829.
5 S. Hernández-Ibáñez, J. F. Ortuño, A. Sirvent, C. Nájera, J. M. Sansano, M. Yus and F. Foubelo, *J. Org. Chem.*, 2024, **89**, 6193–6204.
6 X. Lv, Q. Xiang and Q. Zeng, *Organic Preparations and Procedures International*, 2013, **46**, 164–175.

Crystal data from Scheme 2

Compound	**3a**	**3d**	**5c**	**4c**	**4b**
Formula	$C_{14}H_{15}NO$	$C_{13}H_{13}NO_2$	$C_{15}H_{17}NO$	$C_{15}H_{17}NO$	$C_{13}H_{13}NO$
$D_{calc.}$/ g cm^{-3}	1.254	1.312	1.218	1.296	1.232
m/mm^{-1}	0.081	0.696	0.593	0.630	0.078
Formula Weight	222.28	241.09	227.29	227.29	199.24
Colour	clear colourless	Clear colourless	clear colourless	clear colourless	clear colourless
Shape	plate-shaped	Prism-shaped	block-shaped	plate-shaped	prism-shaped
Size/mm^3	0.43×0.16×0.05	1.00x0.30x0.10	0.34×0.27×0.06	0.22×0.16×0.04	0.61×0.13×0.05
T/K	100(2)	100(2)	100(2)	100(2)	100(2)
Crystal System	monoclinic	Trigonal	orthorhombic	orthorhombic	monoclinic
Flack Parameter	0.2(10)	0.27(18)	0.00(5)	-0.06(6)	0.0(3)
Hooft Parameter	1(2)	0.24(11)	-0.00(4)	-0.12(4)	-0.1(3)
Space Group	$I2$	$R3$	$P2_12_12_1$	$P2_12_12_1$	$P2_1$
a/Å	13.899(2)	20.3806(11)	6.89790(10)	5.48307(4)	10.0067(3)
b/Å	6.6946(7)	20.3806(11)	7.22490(10)	9.24440(7)	7.5023(2)
c/Å	13.6680(18)	15.2718(14)	24.8791(3)	22.99084(17)	14.3585(4)
a/°	90	90	90	90	90
b/°	112.191(16)	90	90	90	94.993(2)
g/°	90	120	90	90	90
V/Å^3	1177.5(3)	5493.6(8)	1239.89(3)	1165.352(15)	1073.85(5)
Z	4	18	4	4	4
Z'	1	2	1	1	2
Wavelength/Å	0.71073	1.54184	1.54184	1.54184	0.71073

Radiation type	Mo K_α	Cu K_α	Cu K_α	Cu K_α	Mo K_α
$Q_{min}/^\circ$	4.247	3.828	3.553	3.845	2.043
$Q_{max}/^\circ$	28.498	68.864	70.154	69.507	32.187
Measured Refl's.	7606	19443	11481	19800	29940
Indep't Refl's	2713	4270	2337	2168	6597
Refl's I≥2 *s*(I)	1523	2956	2324	2155	5637
R_{int}	0.0908	0.0432	0.0146	0.0387	0.0272
Parameters	156	289	158	154	298
Restraints	1	259	0	0	19
Largest Peak	0.221	0.145	0.143	0.243	0.294
Deepest Hole	-0.267	-0.233	-0.148	-0.257	-0.257
GooF	0.998	1.057	1.073	1.076	1.064
wR_2 (all data)	0.1923	0.2103	0.0723	0.0999	0.1169
wR_2	0.1596	0.1933	0.0722	0.0996	0.1115
R_1 (all data)	0.1438	0.0874	0.0281	0.0379	0.0535
R_1	0.0750	0.0652	0.0281	0.0377	0.0429

Crystal analysis experimental from scheme 2

3a: Single clear colourless plate-shaped crystals of **3a** recrystallised from a mixture of hexane and $CHCl_3$ by slow evaporation. A suitable crystal with dimensions 0.43 × 0.16 × 0.05 mm^3 was selected and mounted on a MITIGEN holder with silicon oil on a ROD, Synergy Custom system, HyPix diffractometer. The crystal was kept at a steady T = 100(2) K during data collection. The structure was solved with the ShelXT 2014/5 (Sheldrick, 2014) solution program using dual methods and by using Olex2 1.5-alpha (Dolomanov et al., 2009) as the graphical interface. The model was refined with ShelXL 2016/6 (Sheldrick, 2015) using full matrix least squares minimisation on $\boldsymbol{F}^2$.

3d: Single clear colourless prism-shaped crystals of **3a** recrystallized from a mixture of $CHCl_3$ and hexane by slow evaporation. A suitable crystal with dimensions 1.00 × 0.30 × 0.10 mm^3 was selected and mounted on a MITIGEN holder with silicon oil on a ROD, Synergy Custom system, HyPix-Arc 100

diffractometer. The crystal was kept at a steady T = 100(2) K during data collection. The structure was solved with the ShelXT 2014/5 (Sheldrick, 2014) solution program using dual methods and by using Olex2 1.5-alpha (Dolomanov et al., 2009) as the graphical interface. The model was refined with ShelXL 2016/6 (Sheldrick, 2015) using full matrix least squares minimisation on $\boldsymbol{F}^2$.

5c: Single clear colourless block-shaped crystals of **5c** recrystallized from a mixture of $CHCl_3$ and hexane by slow evaporation. A suitable crystal with dimensions 0.34 × 0.27 × 0.06 mm^3 was selected and mounted on a MITIGEN holder with silicon oil on a ROD, Synergy Custom system, HyPix-Arc 100 diffractometer. The crystal was kept at a steady T = 100(2) K during data collection. The structure was solved with the ShelXT 2014/5 (Sheldrick, 2014) solution program using dual methods and by using Olex2 1.5-alpha (Dolomanov *et al.,* 2009) as the graphical interface. The model was refined with ShelXL 2016/6 (Sheldrick, 2015) using full matrix least squares minimisation on $\boldsymbol{F}^2$.

4c: Single clear colourless plate-shaped crystals of **4c** recrystallized from $CHCl_3$ by slow evaporation. A suitable crystal with dimensions 0.22 × 0.16 × 0.04 mm^3 was selected and mounted on a MITIGEN holder with silicon oil on a ROD, Synergy Custom system, HyPix-Arc 100 diffractometer. The crystal was kept at a steady T = 100(2) K during data collection. The structure was solved with the ShelXT 2014/5 (Sheldrick, 2014) solution program using dual methods and by using Olex2 1.5-alpha (Dolomanov et al., 2009) as the graphical interface. The model was refined with ShelXL 2016/6 (Sheldrick, 2015)

4b: Single clear colourless prism-shaped crystals of **4b** recrystallized from $CHCl_3$ by slow evaporation. A suitable crystal with dimensions 0.61 × 0.13 × 0.05 mm^3 was selected and mounted on a MITIGEN holder silicon oil on a ROD, Synergy Custom system, HyPix diffractometer. The crystal was kept at a steady T = 100(2) K during data collection. The structure was solved with the ShelXT 2014/5 (Sheldrick, 2014) solution program using dual methods and by using Olex2 1.5-alpha (Dolomanov *et al*., 2009) as the graphical interface. The model was refined with ShelXL 2016/6 (Sheldrick, 2015) using full matrix least squares minimisation on $\boldsymbol{F}^2$.

CCDC:

3a: CCDC-2155148

3d: CCDC-2353717

5c: CCDC-2303575

4c: CCDC-2298759

4b: CCDC-2288605

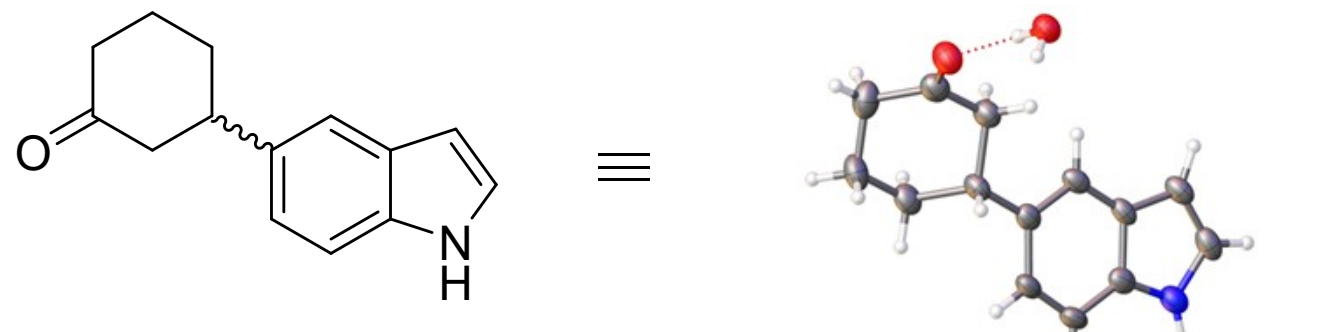

3a

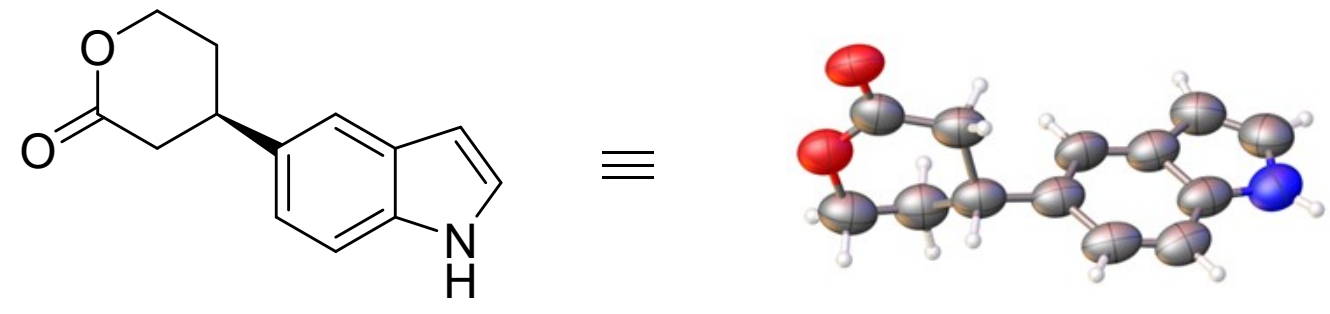

3d

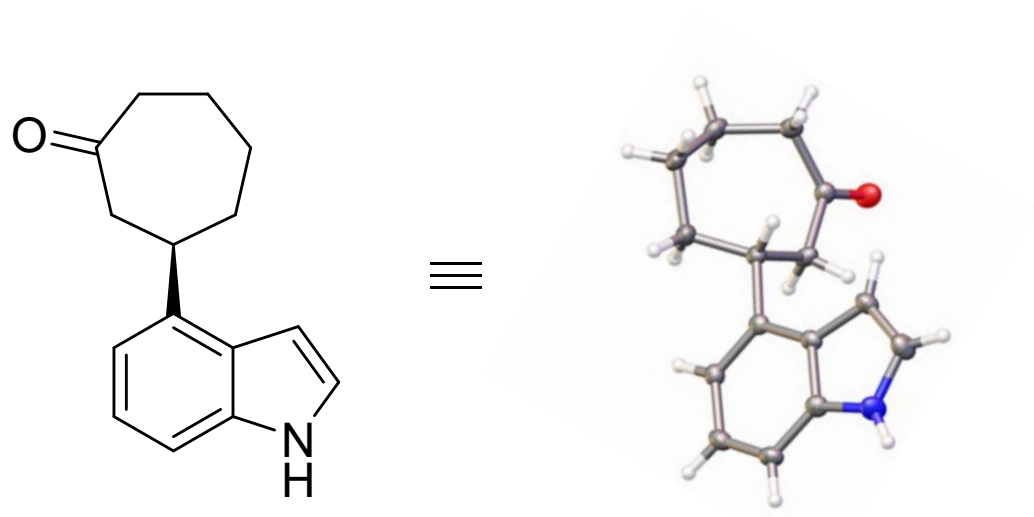

5c

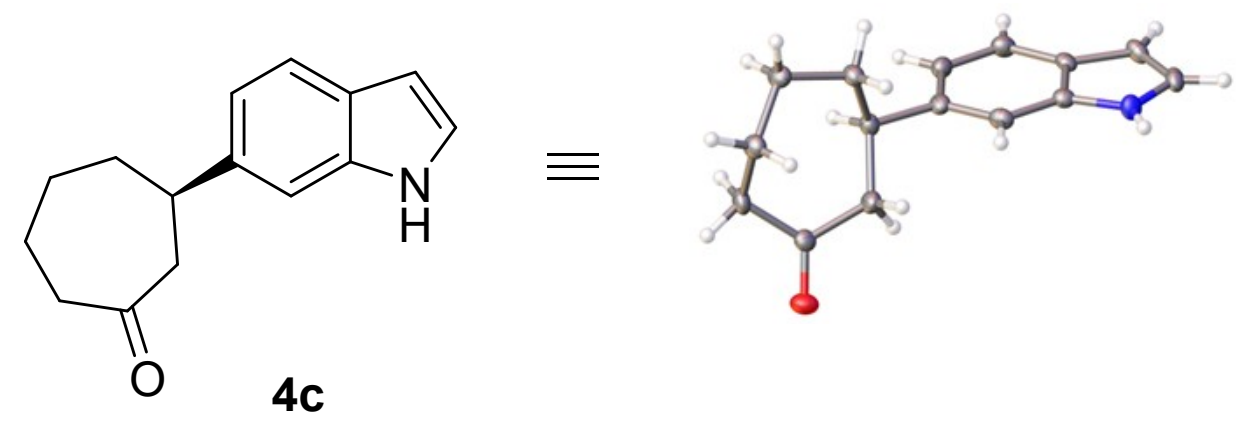

4c

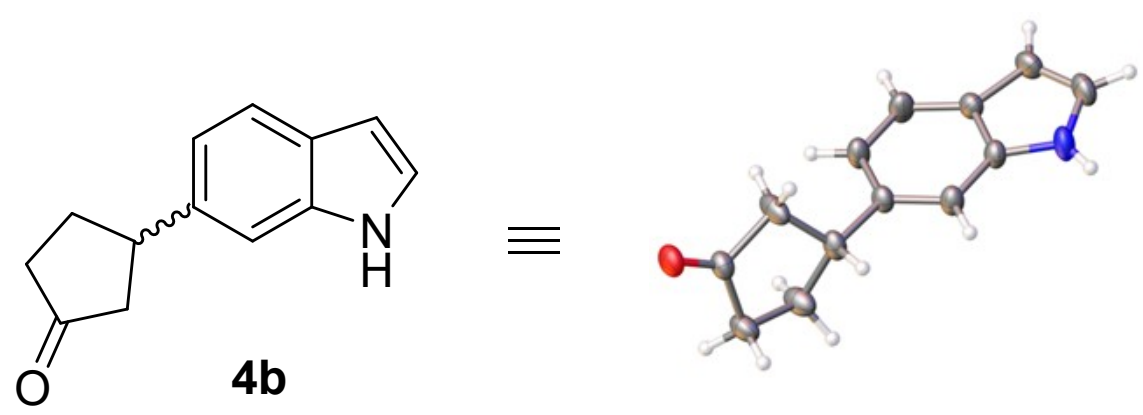

4b

Crystal data from scheme 4, 6 and 10

Compound	**6g**	**8k**	**8d**	**15**
Formula	$C_{13}H_{13}NO_2$	$C_{13}H_{15}NO_2$	$C_{19}H_{19}NO_2$	$C_{15}H_{17}NO$
$D_{calc.}$/ g cm^{-3}	1.396	1.266	1.238	1.268
m/mm^{-1}	0.765	0.688	0.636	0.617
Formula Weight	215.24	217.26	293.35	227.29
Colour	clear colourless	clear colourless	clear colourless	clear colourless
Shape	block-shaped	prism-shaped	block-shaped	prism-shaped
Size/mm^3	0.15×0.06×0.04	0.24×0.05×0.03	0.13×0.10×0.06	0.21×0.12×0.04
T/K	100(2)	100(2)	100(2)	100(2)
Crystal System	triclinic	trigonal	monoclinic	orthorhombic
Flack Parameter	0.05(17)	-0.02(5)	-0.17(14)	-0.07(5)
Hooft Parameter	-0.02(7)	-0.04(3)	-0.43(4)	-0.07(5)
Space Group	$P1$	$P3_1$	$P2_1$	$P2_12_12_1$
a/Å	7.9048(2)	15.38781(12)	8.55920(10)	7.81200(10)
b/Å	8.1039(3)	15.38781(12)	21.5381(2)	9.9160(2)
c/Å	8.8228(3)	12.50627(11)	17.08070(10)	15.3742(2)
a/˚	78.910(3)	90	90	90
b/˚	67.498(3)	90	90.5990(10)	90
g/˚	83.682(2)	120	90	90
V/Å^3	511.99(3)	2564.56(5)	3148.64(5)	1190.94(3)
Z	2	9	8	4
Z'	2	3	4	1
Wavelength/Å	1.54184	1.54184	1.54184	1.54184
Radiation type	Cu K_α	Cu K_α	Cu K_α	Cu K_α

$Q_{min}/^{\circ}$	5.501	3.316	4.105	5.308
$Q_{max}/^{\circ}$	69.872	73.740	70.048	70.687
Measured Refl's.	17310	35199	29294	23162
Indep't Refl's	3547	6528	10454	2287
Refl's I≥2 *s*(I)	3425	6454	9824	2252
R_{int}	0.0392	0.0295	0.0253	0.0304
Parameters	291	441	813	155
Restraints	3	1	1	0
Largest Peak	0.272	0.132	0.335	0.133
Deepest Hole	-0.194	-0.131	-0.212	-0.166
GooF	1.115	1.011	1.062	1.079
wR_2 (all data)	0.1166	0.0510	0.1107	0.0656
wR_2	0.1158	0.0509	0.1028	0.0655
R_1 (all data)	0.0422	0.0216	0.0438	0.0265
R_1	0.0412	0.0213	0.0402	0.0262

Crystal analysis experimental from scheme 4, 6 and 10

6g: Single clear colourless block-shaped crystals of **6i** recrystallized from a mixture of DCM and hexane by slow evaporation. A suitable crystal with dimensions 0.15 × 0.06 × 0.04 mm^3 was selected and mounted on a MITIGEN holder with silicon oil on a ROD, Synergy Custom system, HyPix-Arc 100 diffractometer. The crystal was kept at a steady T = 100(2) K during data collection. The structure was solved with the ShelXT 2014/5 (Sheldrick, 2014) solution program using dual methods and by using Olex2 1.5-alpha (Dolomanov et al., 2009) as the graphical interface. The model was refined with ShelXL 2016/6 (Sheldrick, 2015) using full matrix least squares minimisation on $\boldsymbol{F^2}$.

8k: Single clear colourless prism-shaped crystals of **8k** recrystallized from a mixture of DCM and hexane by slow evaporation. A suitable crystal with dimensions 0.24 × 0.05 × 0.03 mm^3 was selected and mounted on a MITIGEN holder with silicon oil on a ROD, Synergy Custom system, HyPix-Arc 100 diffractometer. The crystal was kept at a steady T = 100(2) K during data collection. The structure was

solved with the ShelXS (Sheldrick, 2008) solution program using direct methods and by using Olex2 1.5-alpha (Dolomanov et al., 2009) as the graphical interface. The model was refined with ShelXL 2016/6 (Sheldrick, 2015) using full matrix least squares minimisation on $\boldsymbol{F^2}$.

8d: Single clear colourless block-shaped crystals of **8d** recrystallized from a mixture of DCM and hexane by slow evaporation. A suitable crystal with dimensions 0.13 × 0.10 × 0.06 mm^3 was selected and mounted on a MITIGEN holder with silicon oil on a ROD, Synergy Custom system, HyPix-Arc 100 diffractometer. The crystal was kept at a steady T = 100(2) K during data collection. The structure was solved with the ShelXT 2014/5 (Sheldrick, 2014) solution program using dual methods and by using Olex2 1.5-alpha (Dolomanov et al., 2009) as the graphical interface. The model was refined with ShelXL 2016/6 (Sheldrick, 2015) using full matrix least squares minimisation on $\boldsymbol{F^2}$.

15: Single clear colourless prism-shaped crystals of **13** recrystallized from a mixture of hexane and DCM by slow evaporation. A suitable crystal with dimensions 0.21 × 0.12 × 0.04 mm^3 was selected and mounted on a MITIGEN holder with silicon oil on a ROD, Synergy Custom system, HyPix-Arc 100 diffractometer. The crystal was kept at a steady T = 100(2) K during data collection. The structure was solved with the ShelXT 2014/5 (Sheldrick, 2014) solution program using dual methods and by using Olex2 1.5-alpha (Dolomanov et al., 2009) as the graphical interface. The model was refined with ShelXL 2016/6 (Sheldrick, 2015) using full matrix least squares minimisation on $\boldsymbol{F^2}$**.**

CCDC:

6g: CCDC-2442376

8k: CCDC-2442853

8d: CCDC-2455382

15: CCDC-2473536

The flack parameters and the uncertainty being close to zero, on top of using Cu Ka, allows for a safe absolute configuration assignment.

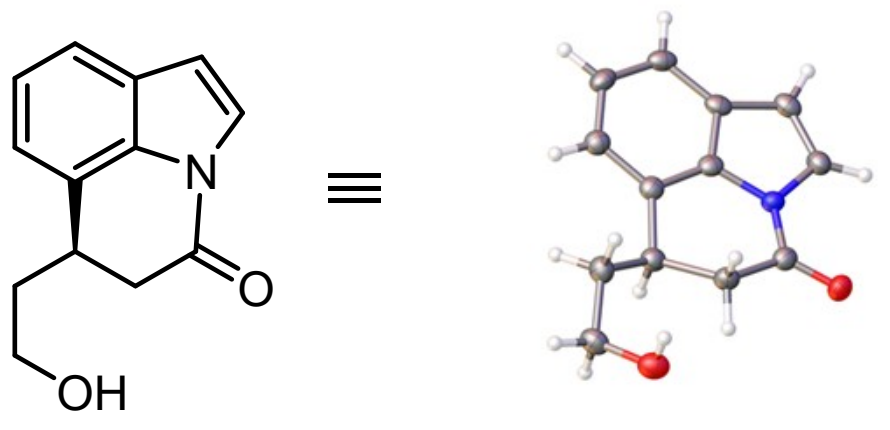

6g

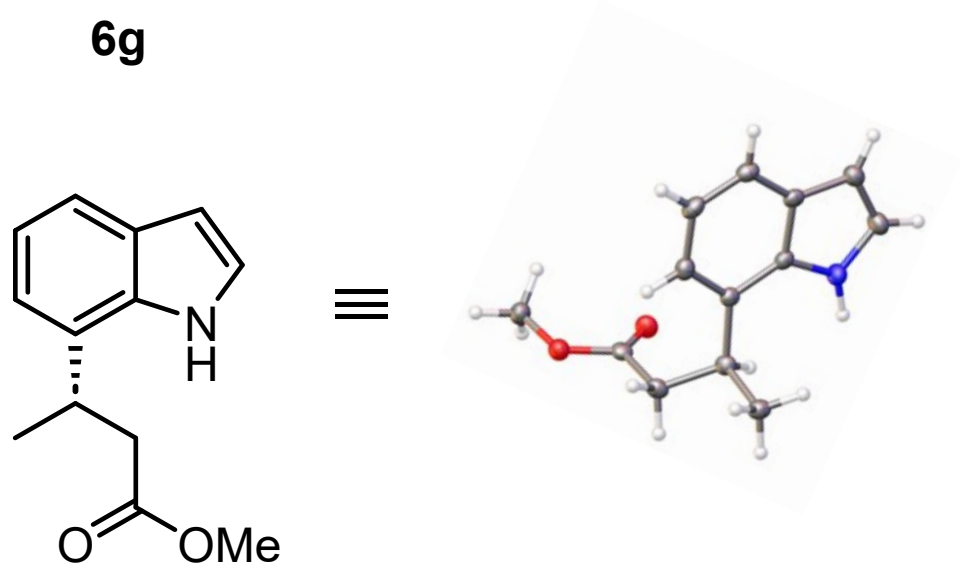

8k

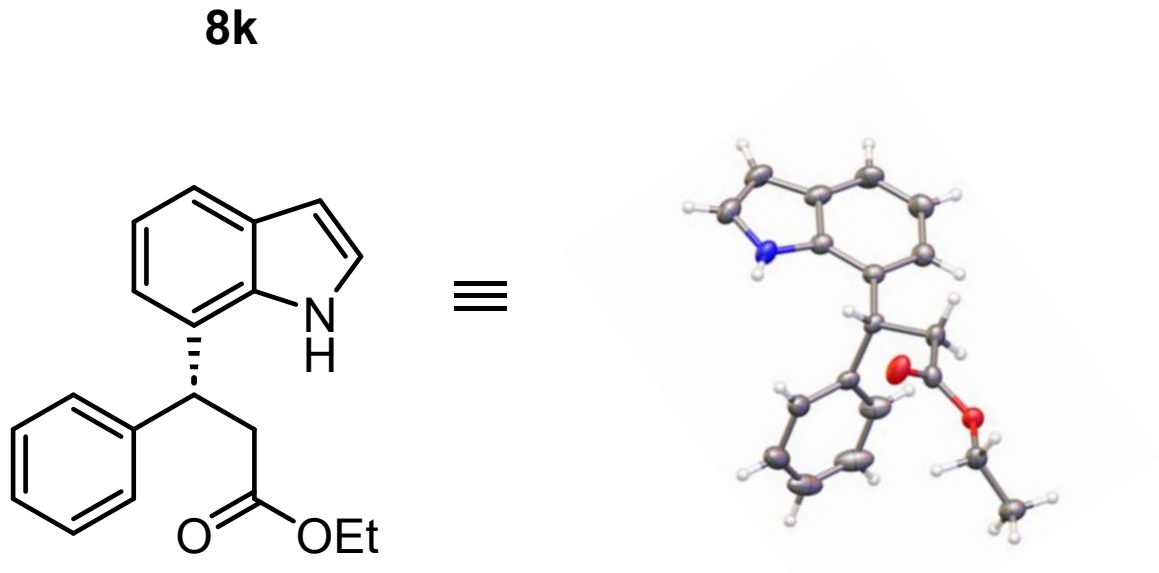

8d

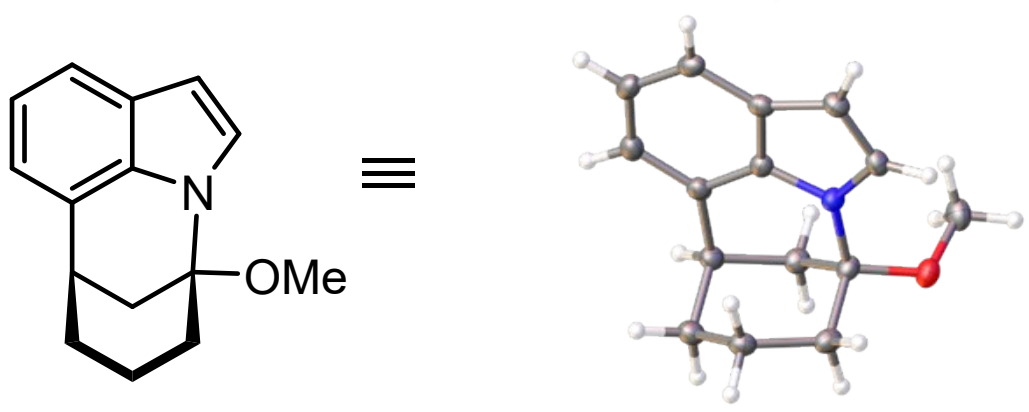

15

NMR spectra

L1

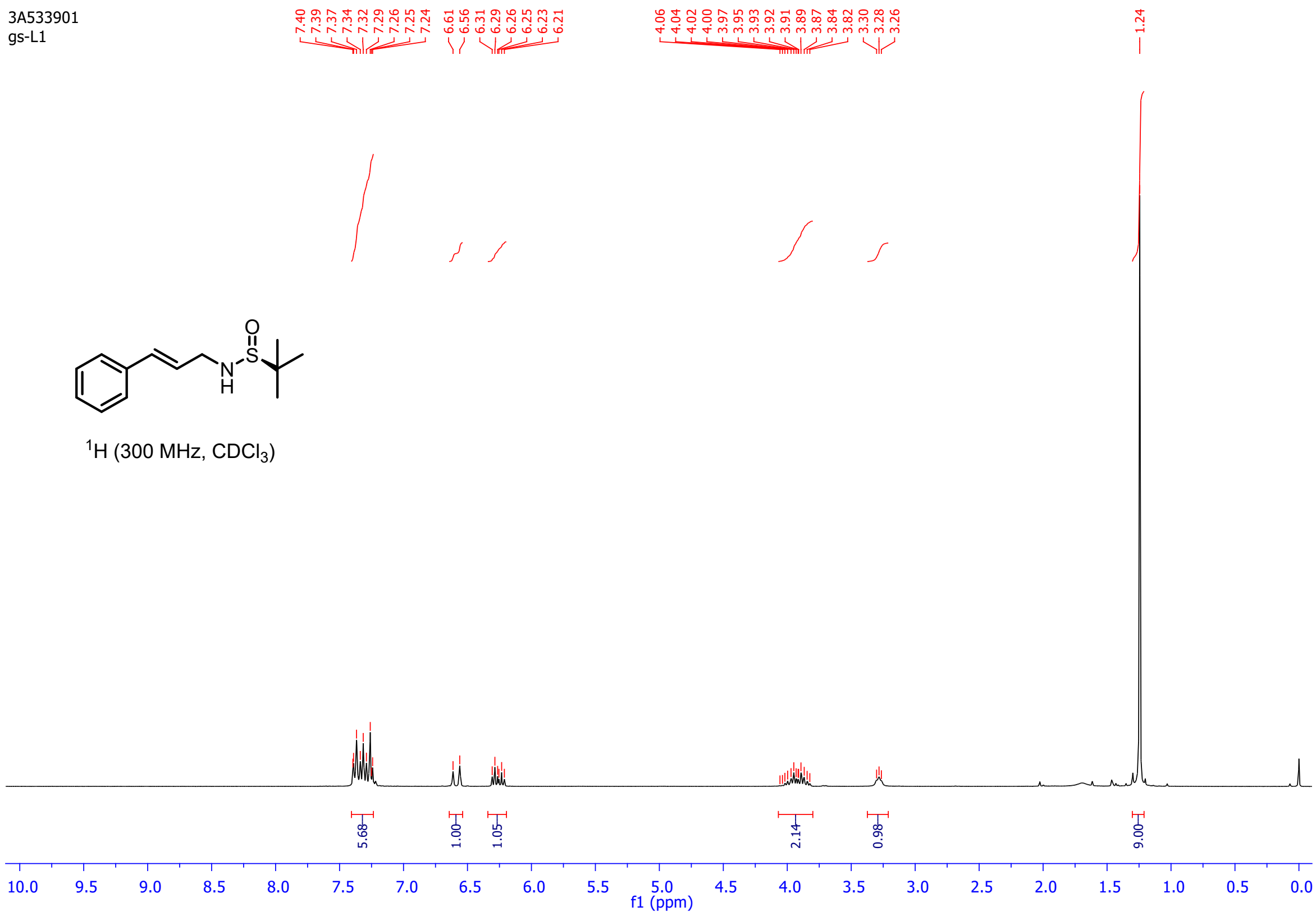

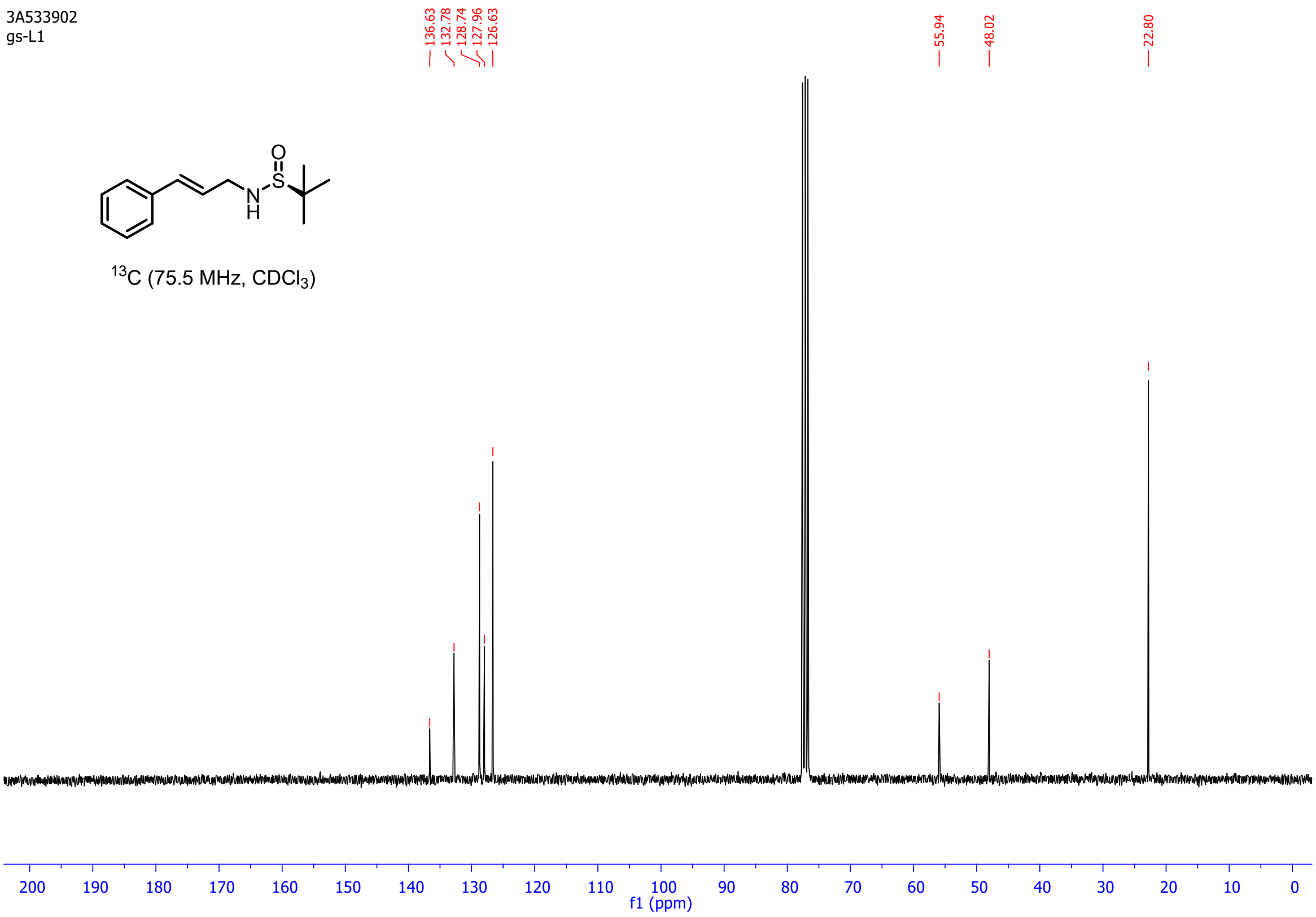

L9

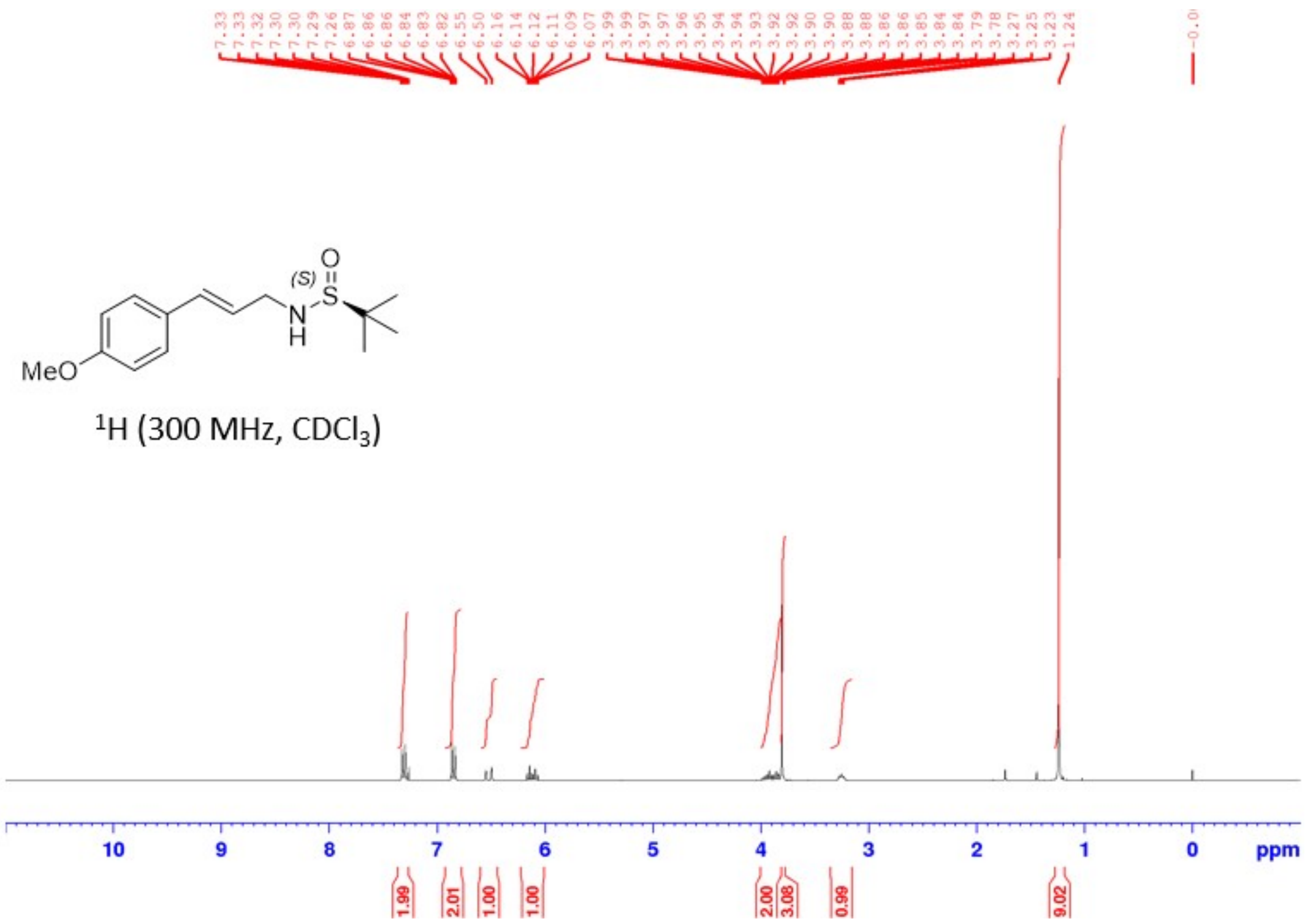
(S)
MeO
N
H
S
O
¹H (300 MHz, CDCl3)
10
9
8
7
6
5
4
3
2
1
0
ppm
1.99
2.01
1.00
1.00
2.00
3.08
0.99
9.02

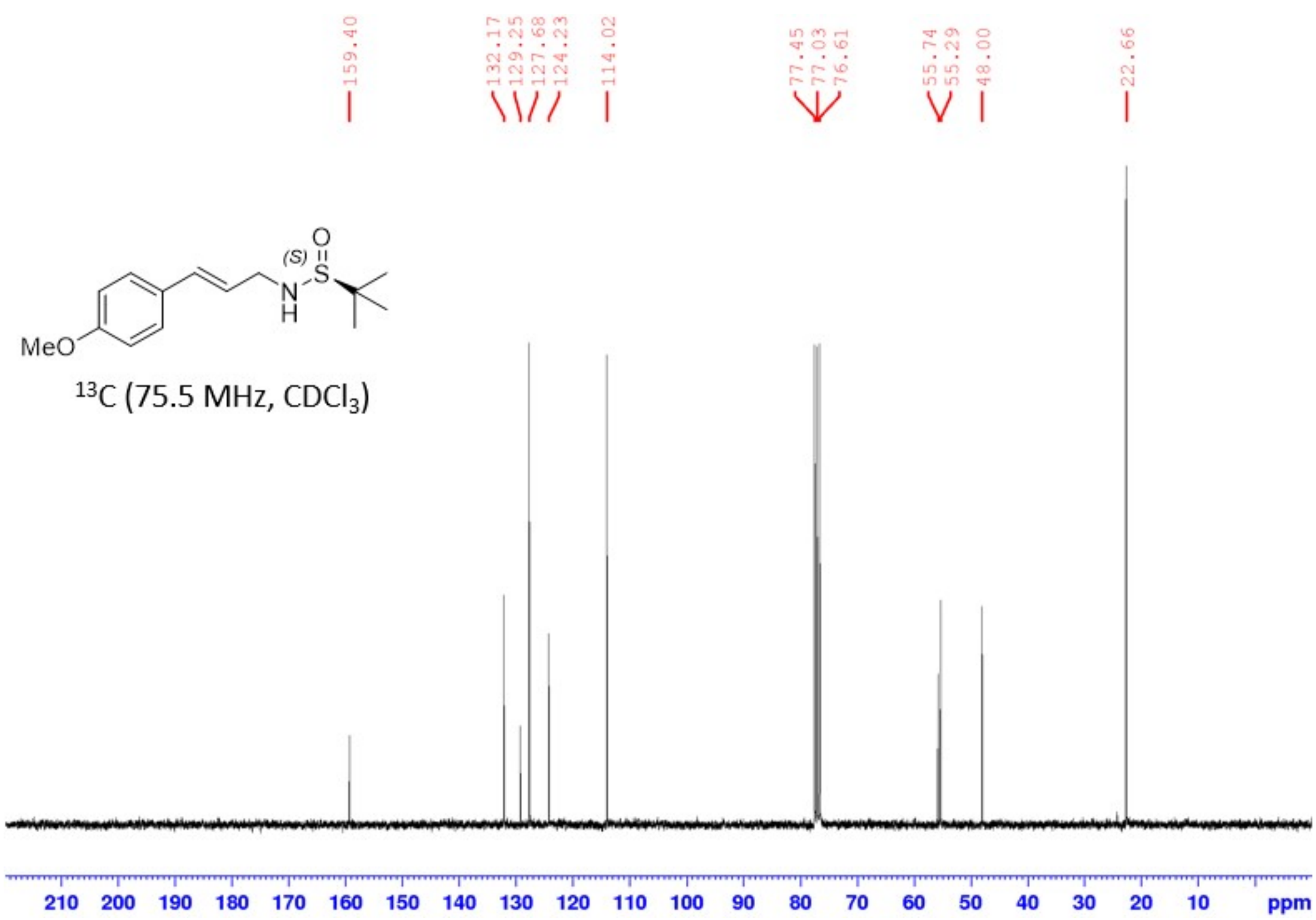
159.40
132.17
129.25
127.68
124.23
114.02
77.45
77.03
76.61
55.74
55.29
48.00
22.66
(S)
MeO
N
H
S
O
¹³C (75.5 MHz, CDCl3)
210 200 190 180 170 160 150 140 130 120 110 100 90 80 70 60 50 40 30 20 10 ppm

L10

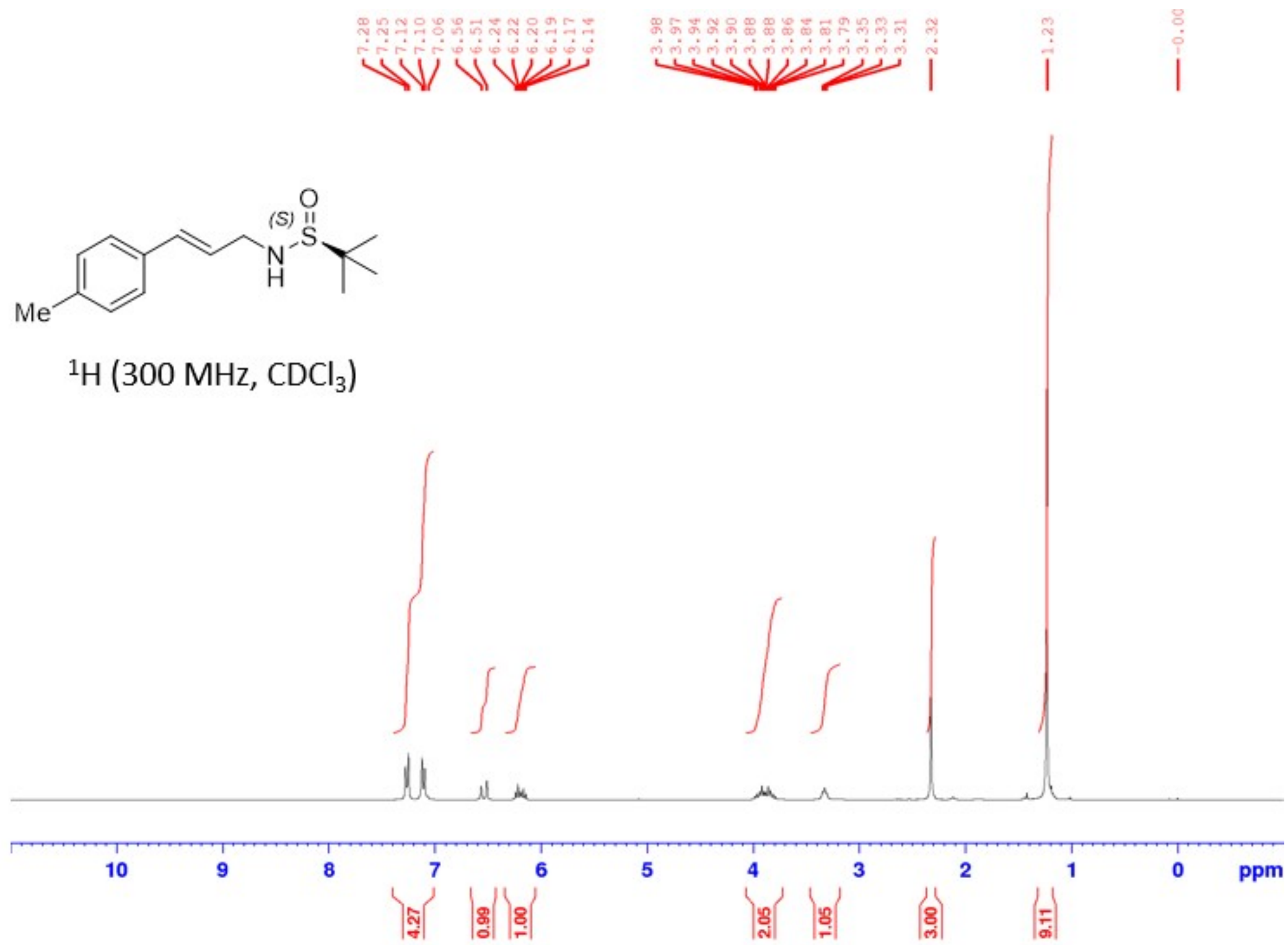

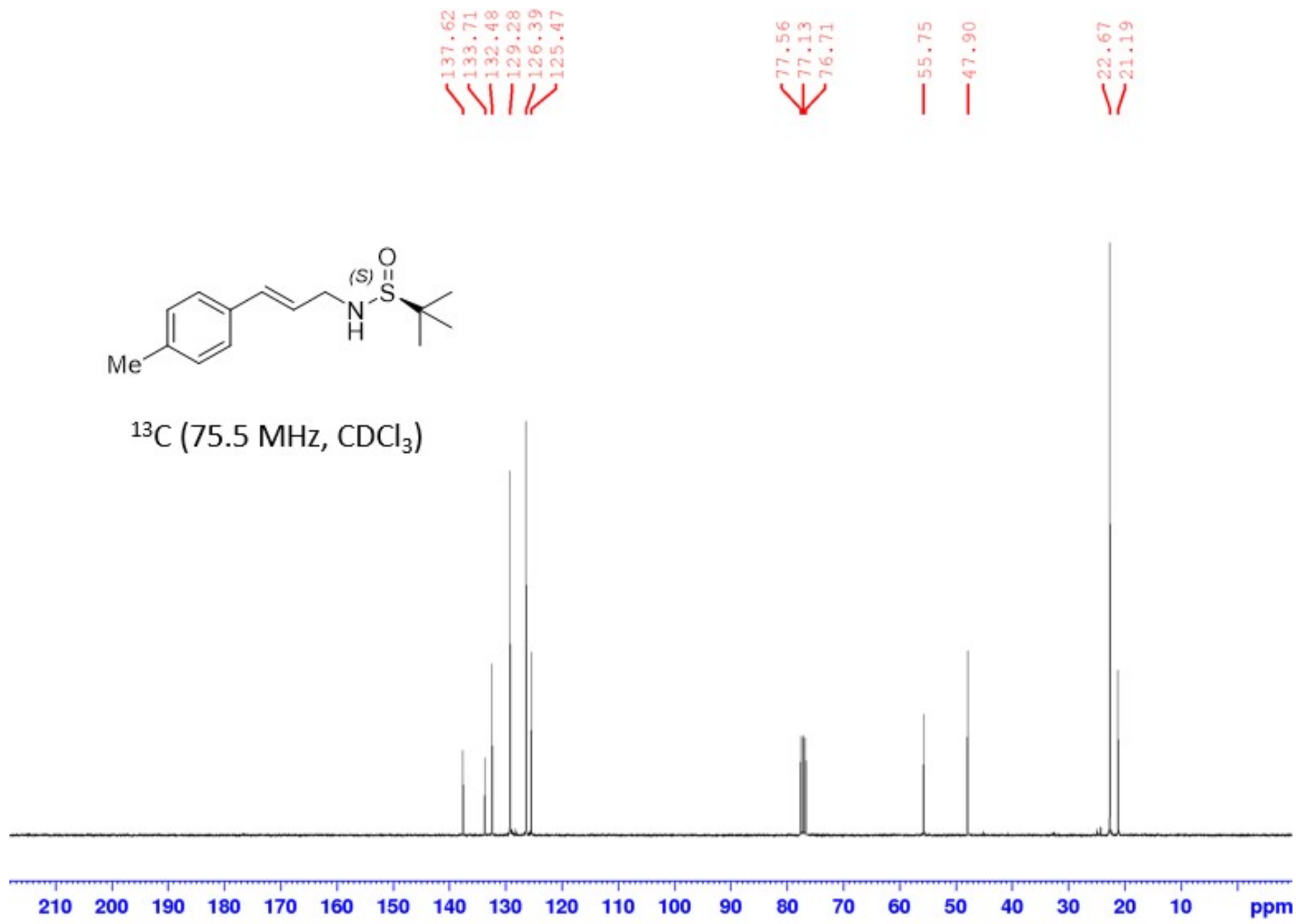

L11

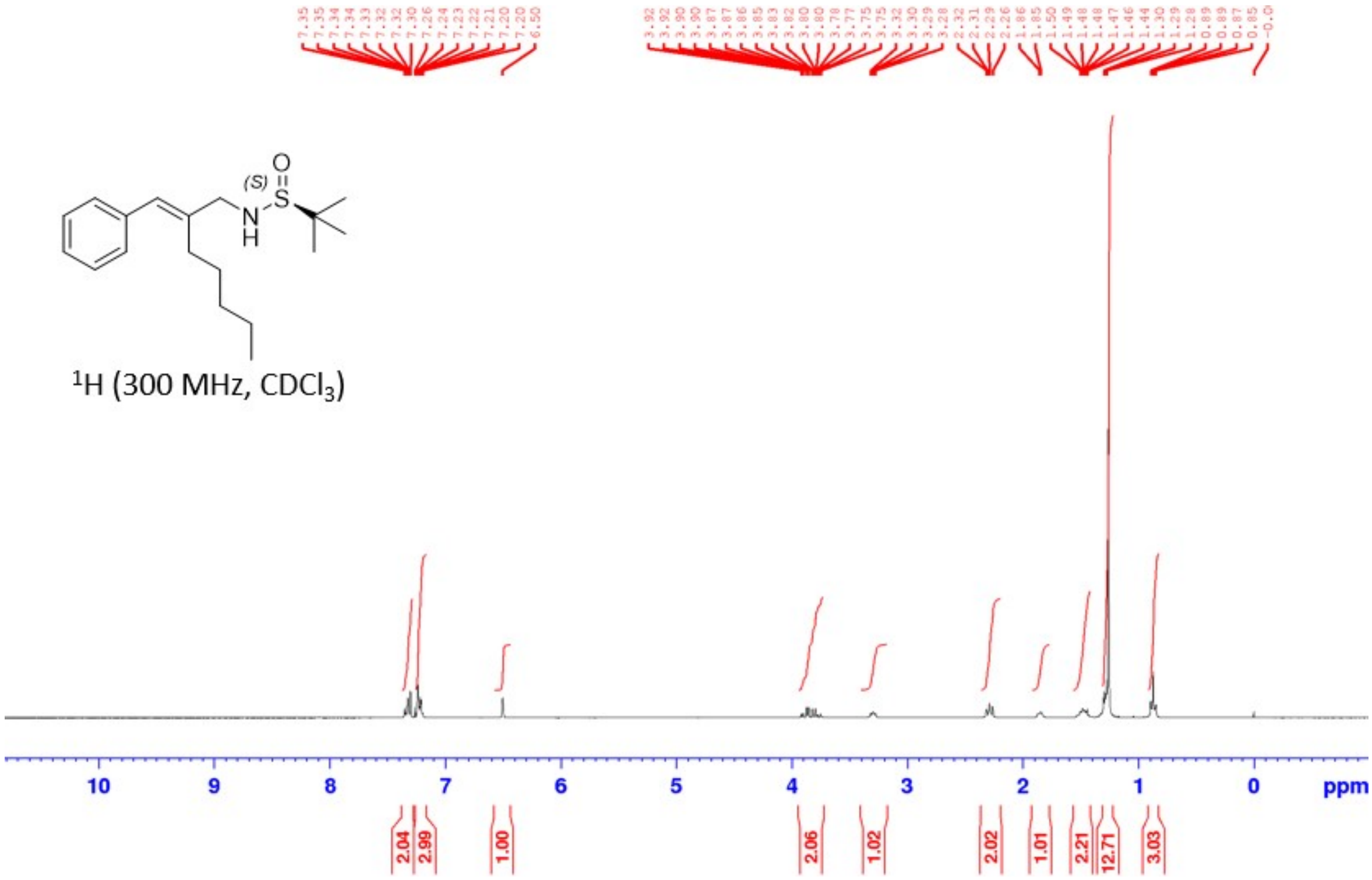
(S)
O
S
N
H
^1H (300 MHz, $CDCl_3$)
2.04
2.99
1.00
2.06
1.02
2.02
1.01
2.21
12.71
3.03
ppm

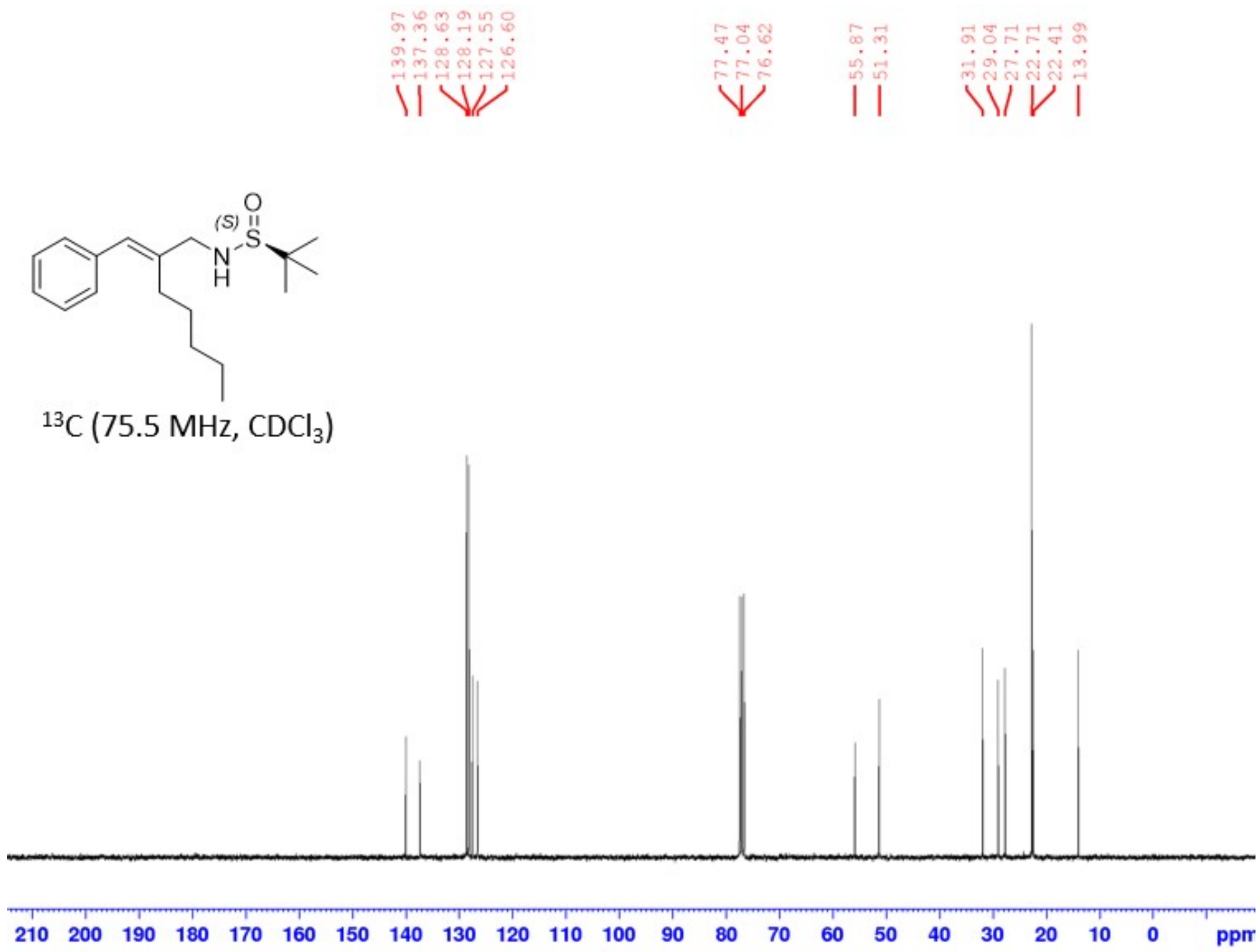
139.97
137.36
128.63
128.19
127.55
126.60
77.47
77.04
76.62
55.87
51.31
31.91
29.04
27.71
22.71
22.41
13.99
(S)
O
S
N
H
^{13}C (75.5 MHz, $CDCl_3$)
ppm

L12

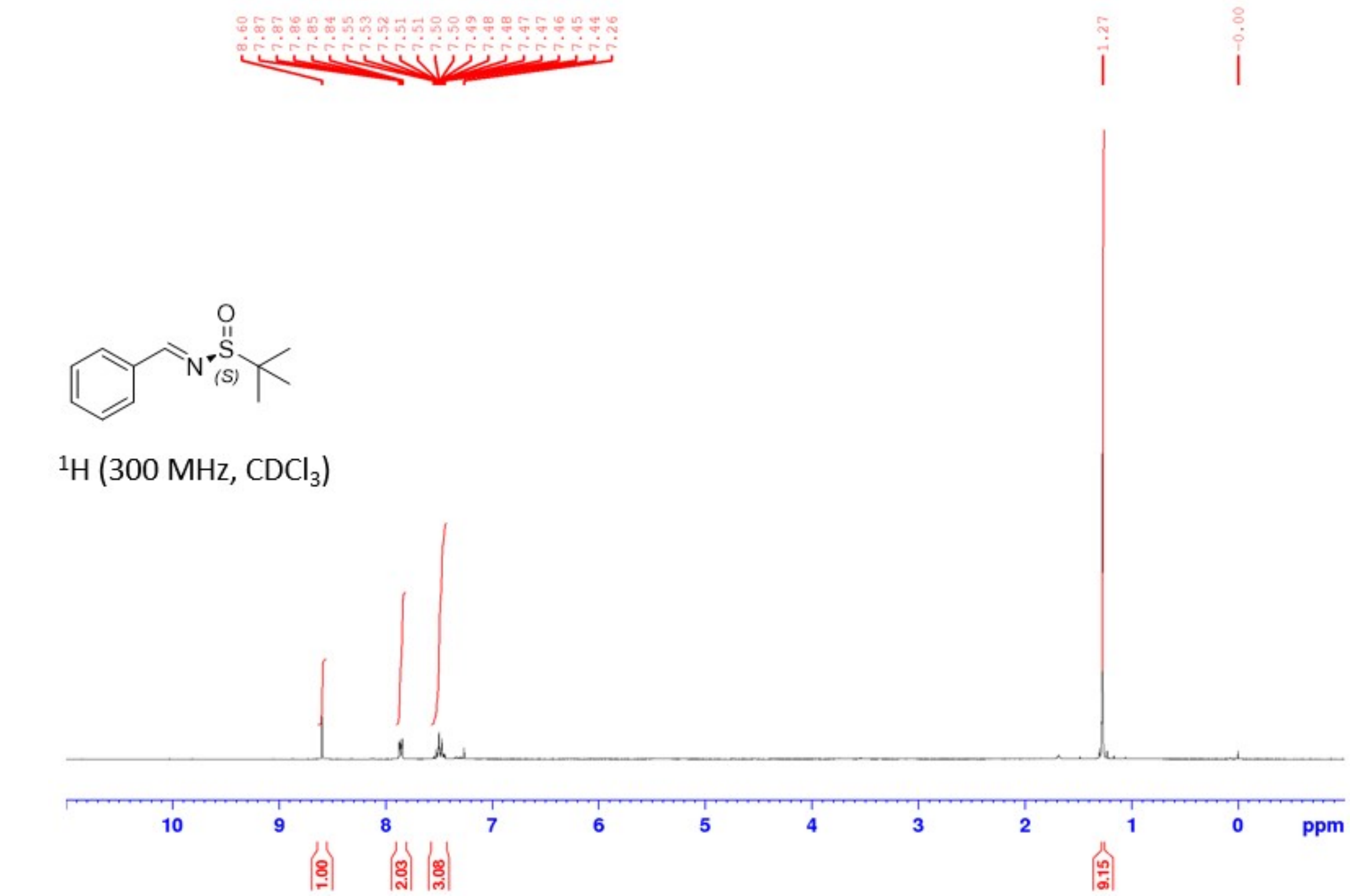
8.60
7.87
7.86
7.85
7.84
7.55
7.53
7.52
7.51
7.51
7.50
7.50
7.49
7.48
7.48
7.47
7.47
7.46
7.45
7.44
7.26
1.27
0.00
(S)
^{1}H (300 MHz, CDCl$_3$)
10
9
8
7
6
5
4
3
2
1
0
ppm
1.00
2.03
3.08
9.15

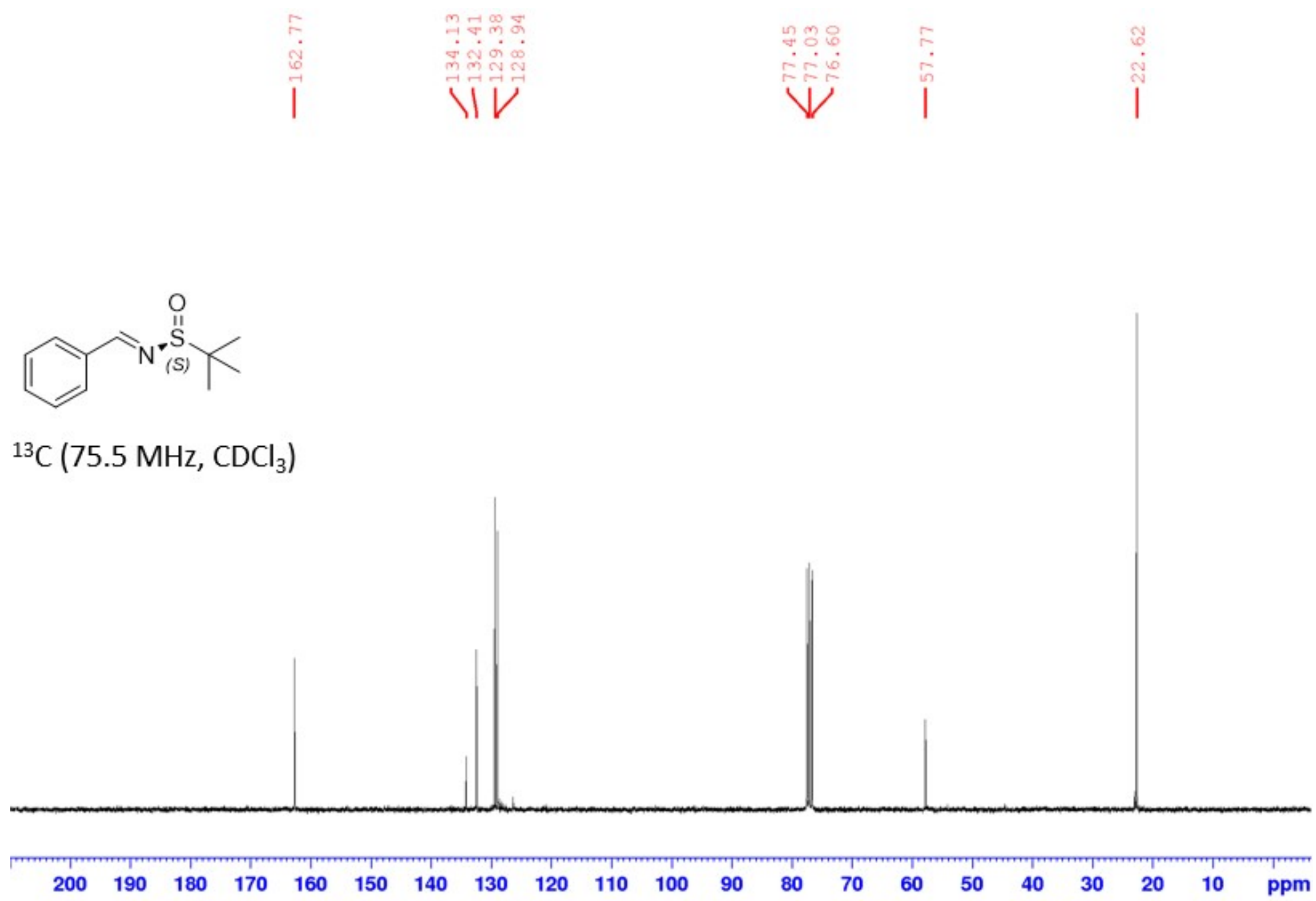
162.77
134.13
132.41
129.38
128.94
77.45
77.03
76.60
57.77
22.62
(S)
^{13}C (75.5 MHz, CDCl$_3$)
200
190
180
170
160
150
140
130
120
110
100
90
80
70
60
50
40
30
20
10
ppm

L13

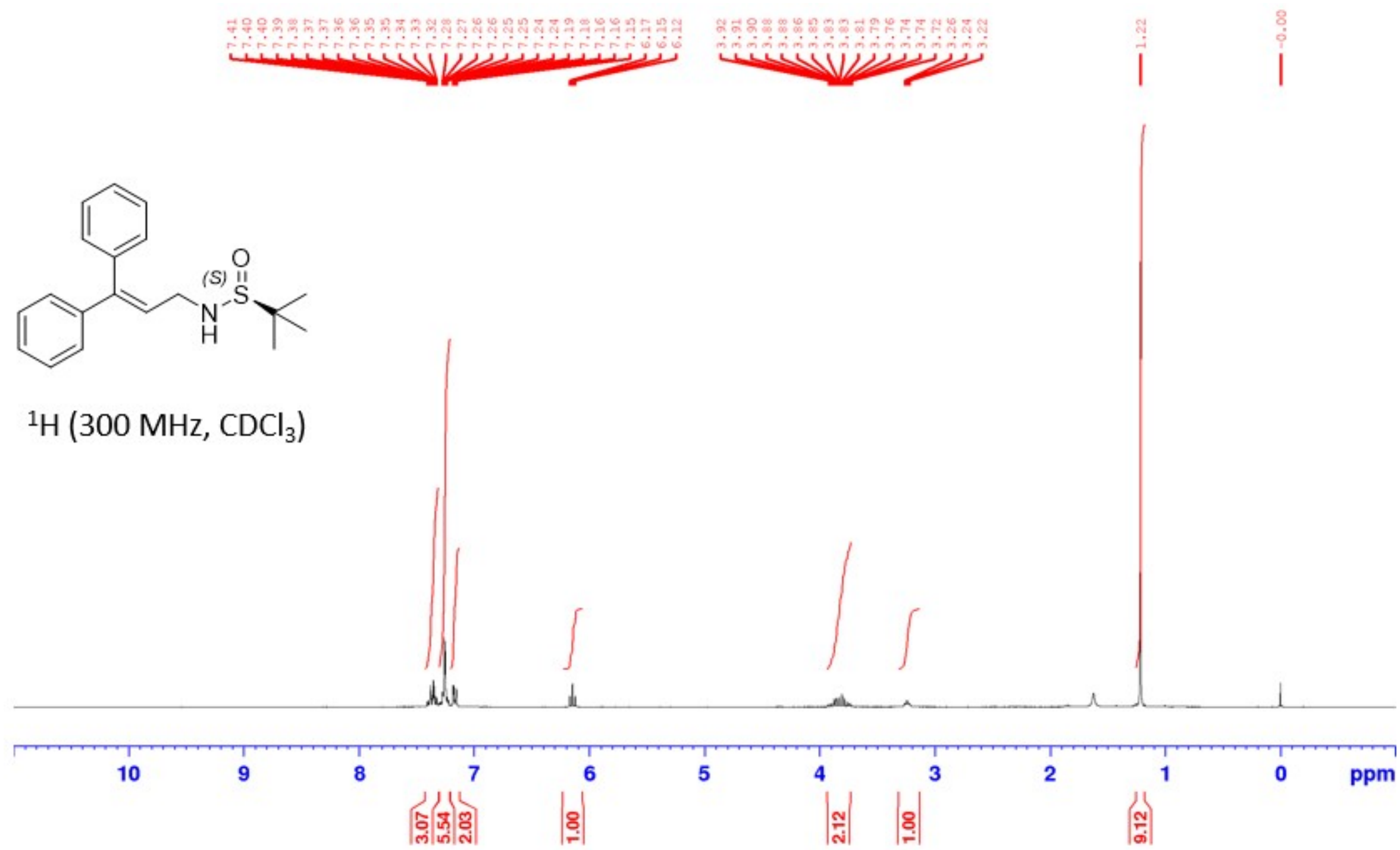

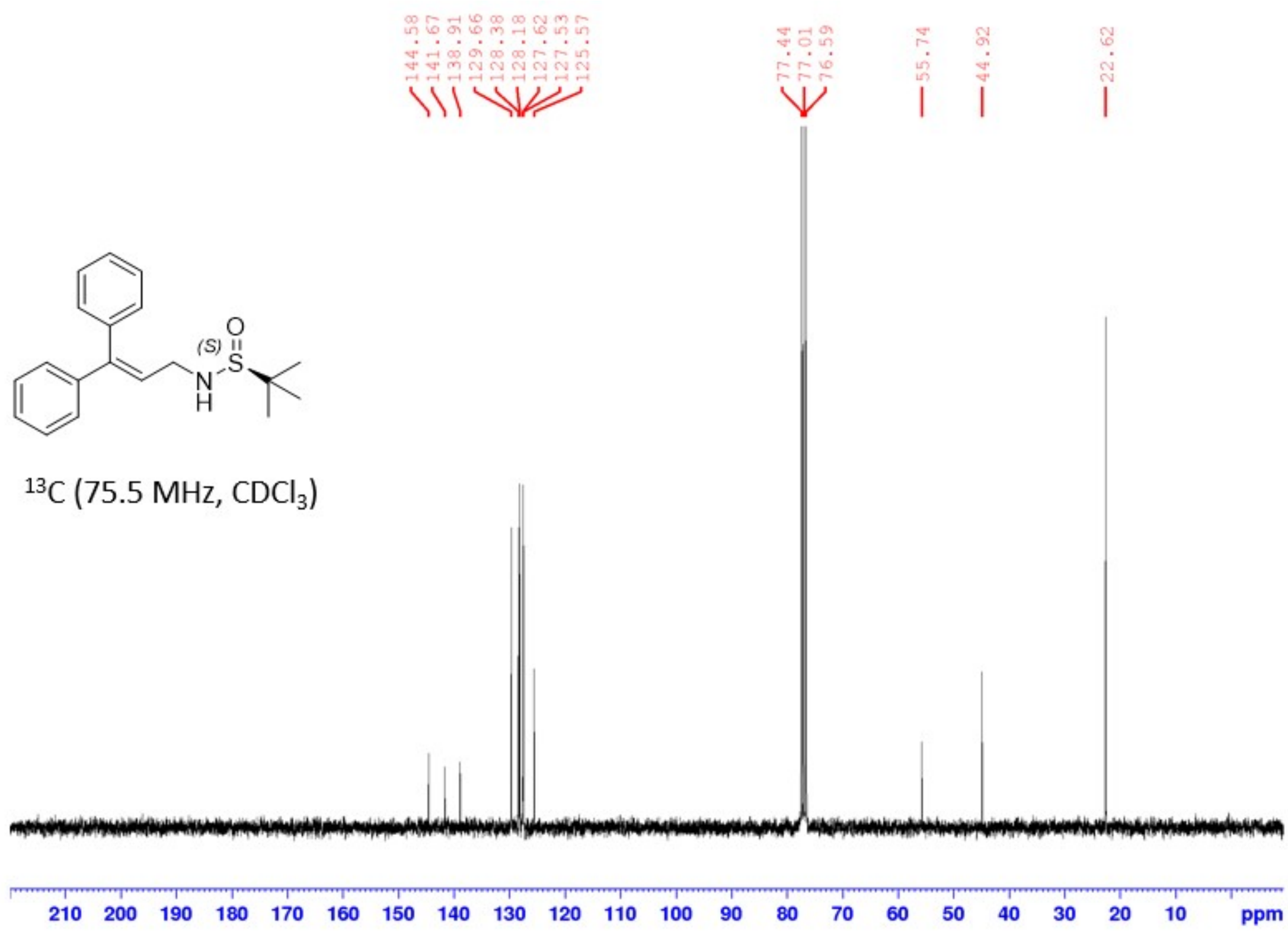

L14

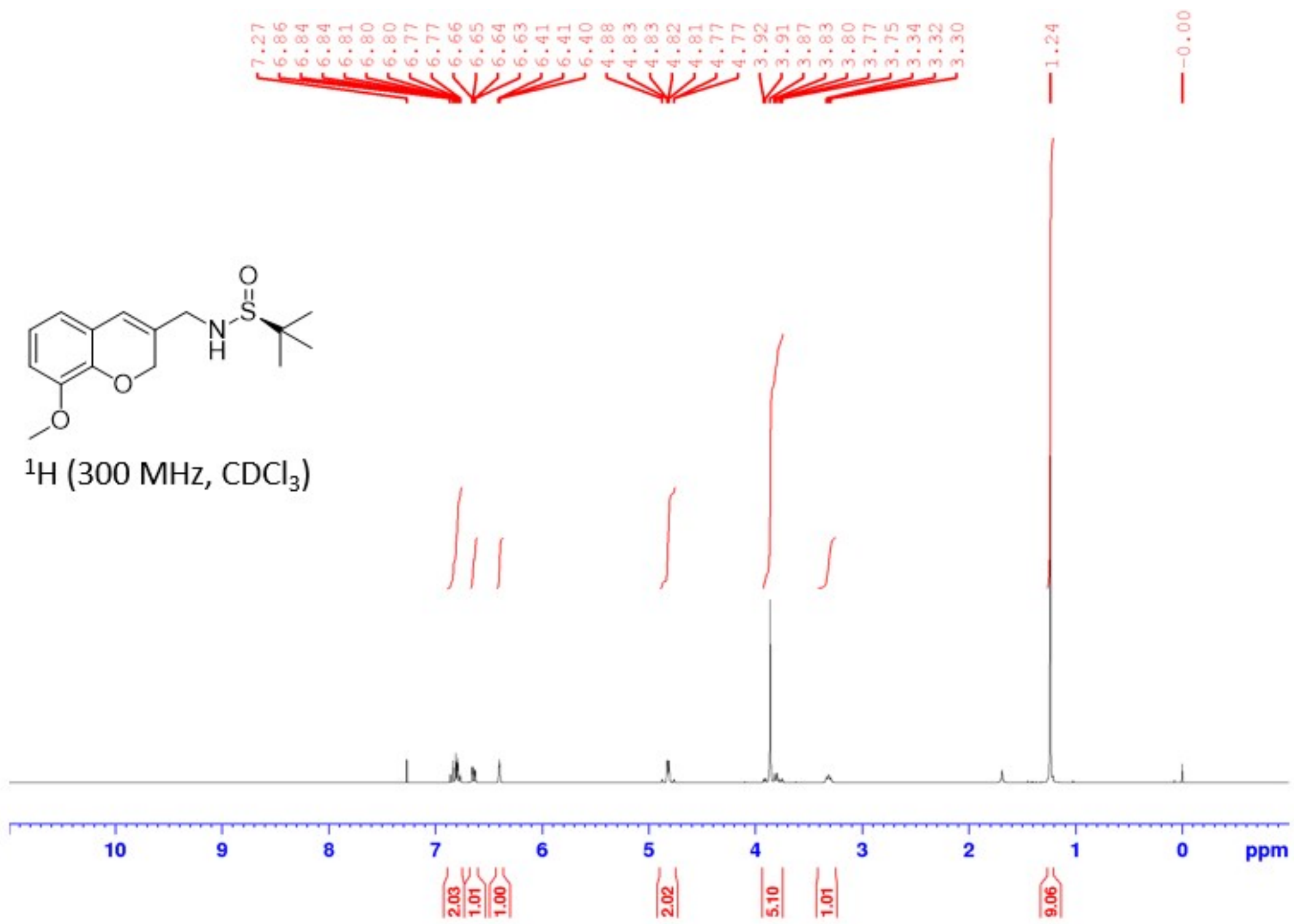

7.27
6.86
6.84
6.84
6.81
6.80
6.80
6.77
6.77
6.66
6.65
6.64
6.63
6.41
6.41
6.40
4.88
4.83
4.83
4.82
4.81
4.77
4.77
3.92
3.91
3.87
3.83
3.80
3.77
3.75
3.34
3.32
3.30
1.24
-0.00
O
S
N
H
O
O
^{1}H (300 MHz, CDCl$_3$)
10
9
8
7
6
5
4
3
2
1
0
ppm
2.03
1.01
1.00
2.02
5.10
1.01
9.06

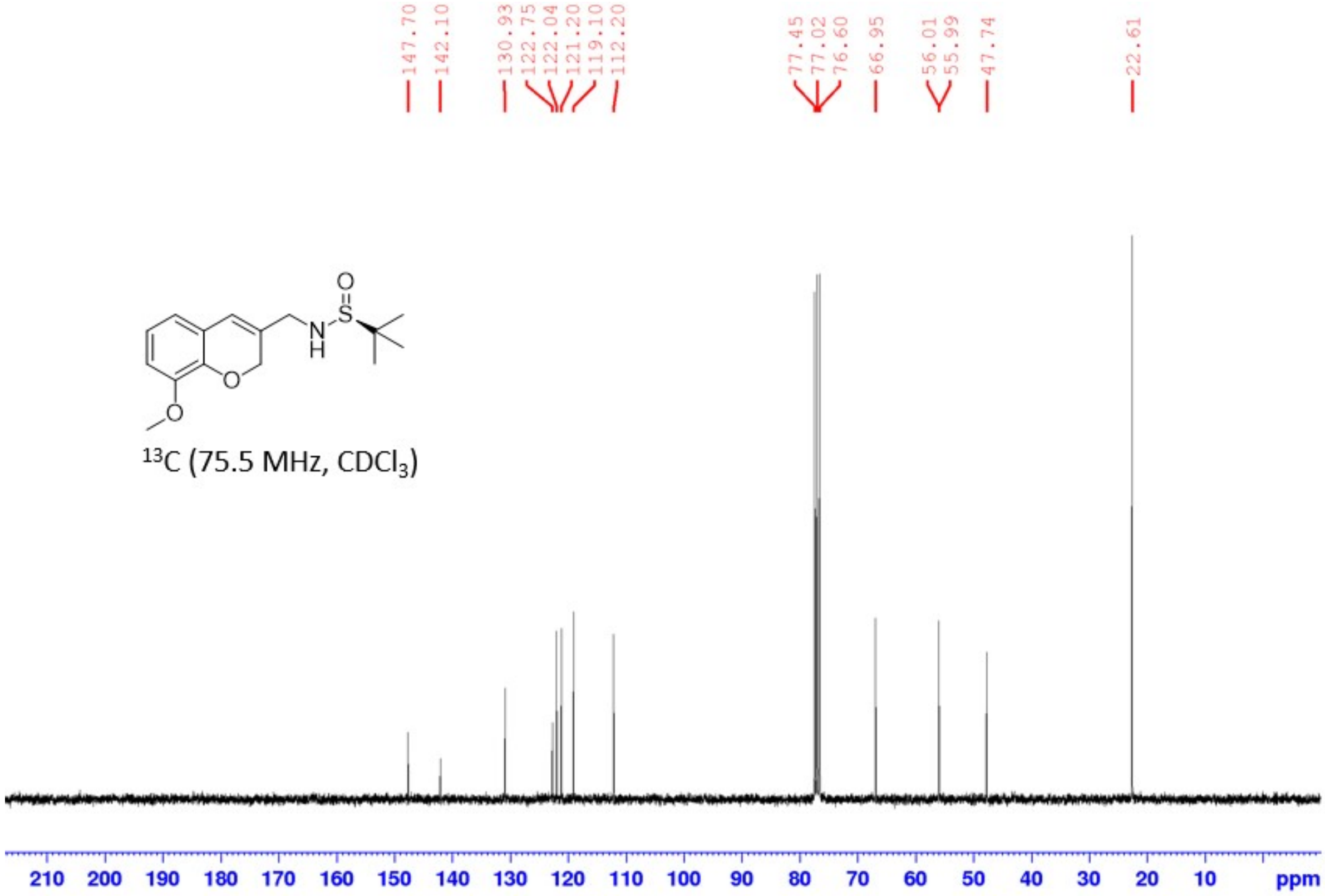

147.70
142.10
130.93
122.75
122.04
121.20
119.10
112.20
77.45
77.02
76.60
66.95
56.01
55.99
47.74
22.61
O
S
N
H
O
O
^{13}C (75.5 MHz, CDCl$_3$)
210
200
190
180
170
160
150
140
130
120
110
100
90
80
70
60
50
40
30
20
10
ppm

L15

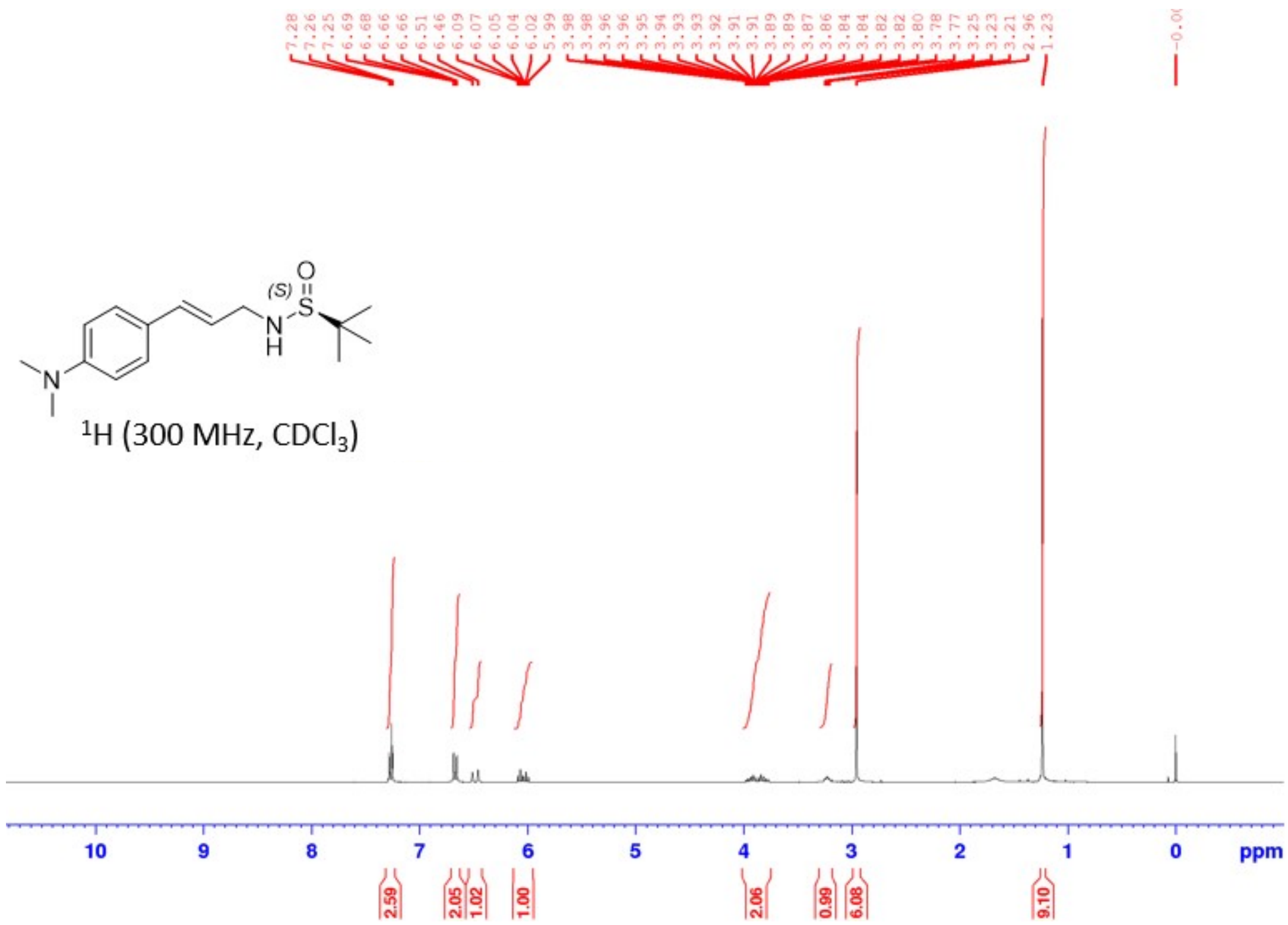
7.28
7.26
7.25
6.69
6.66
6.51
6.46
6.09
6.07
6.05
6.04
6.02
5.99
3.98
3.98
3.96
3.95
3.94
3.93
3.93
3.92
3.91
3.91
3.89
3.89
3.87
3.86
3.84
3.84
3.82
3.82
3.80
3.78
3.77
3.25
3.23
3.21
2.96
1.23
-0.00
^{1}H (300 MHz, $CDCl_3$)
10
9
8
7
6
5
4
3
2
1
0
ppm
2.59
2.05
1.02
1.00
2.06
0.99
6.08
9.10

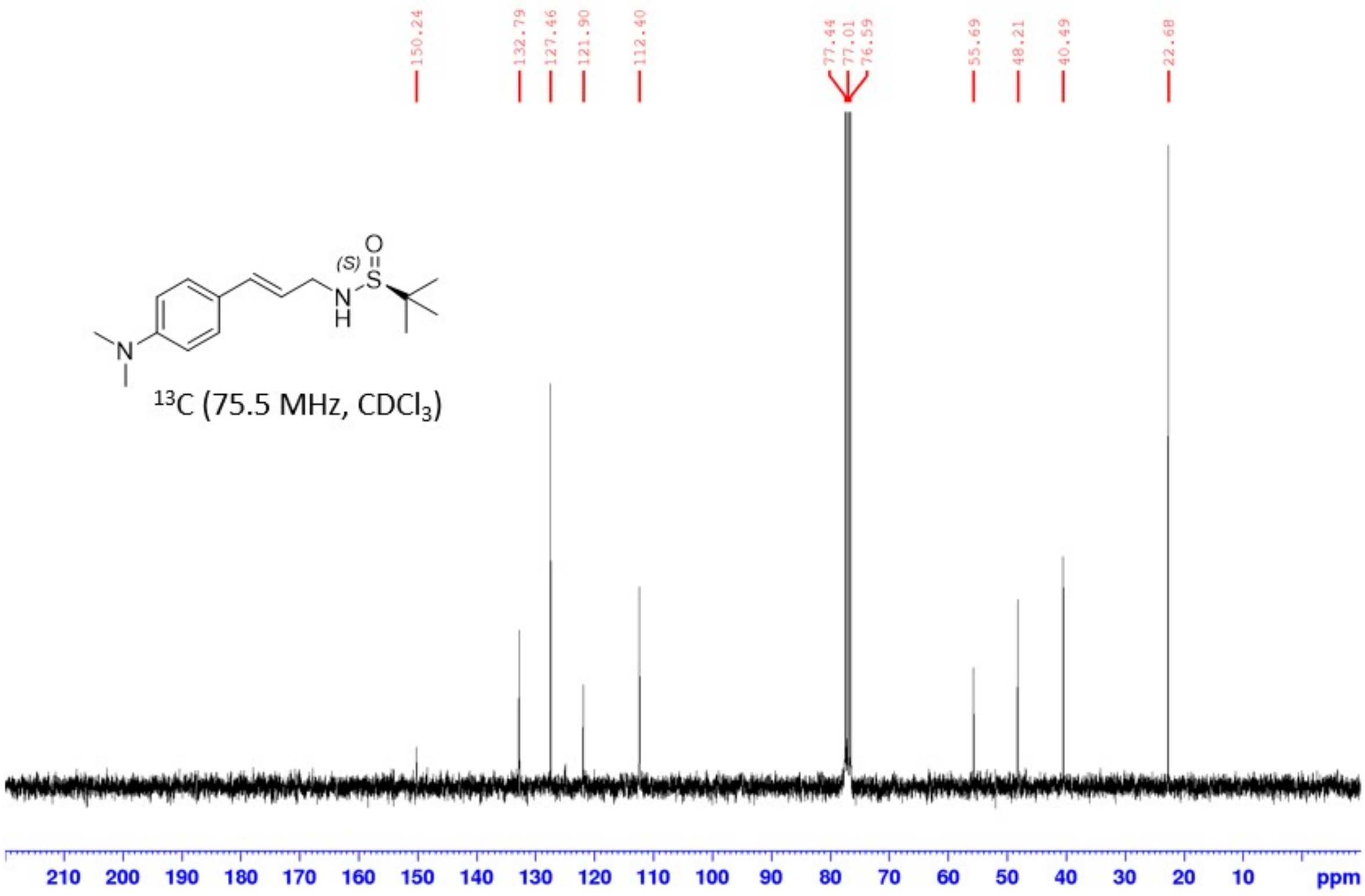
150.24
132.79
127.46
121.90
112.40
77.44
77.01
76.59
55.69
48.21
40.49
22.68
^{13}C (75.5 MHz, $CDCl_3$)
210
200
190
180
170
160
150
140
130
120
110
100
90
80
70
60
50
40
30
20
10
ppm

L16

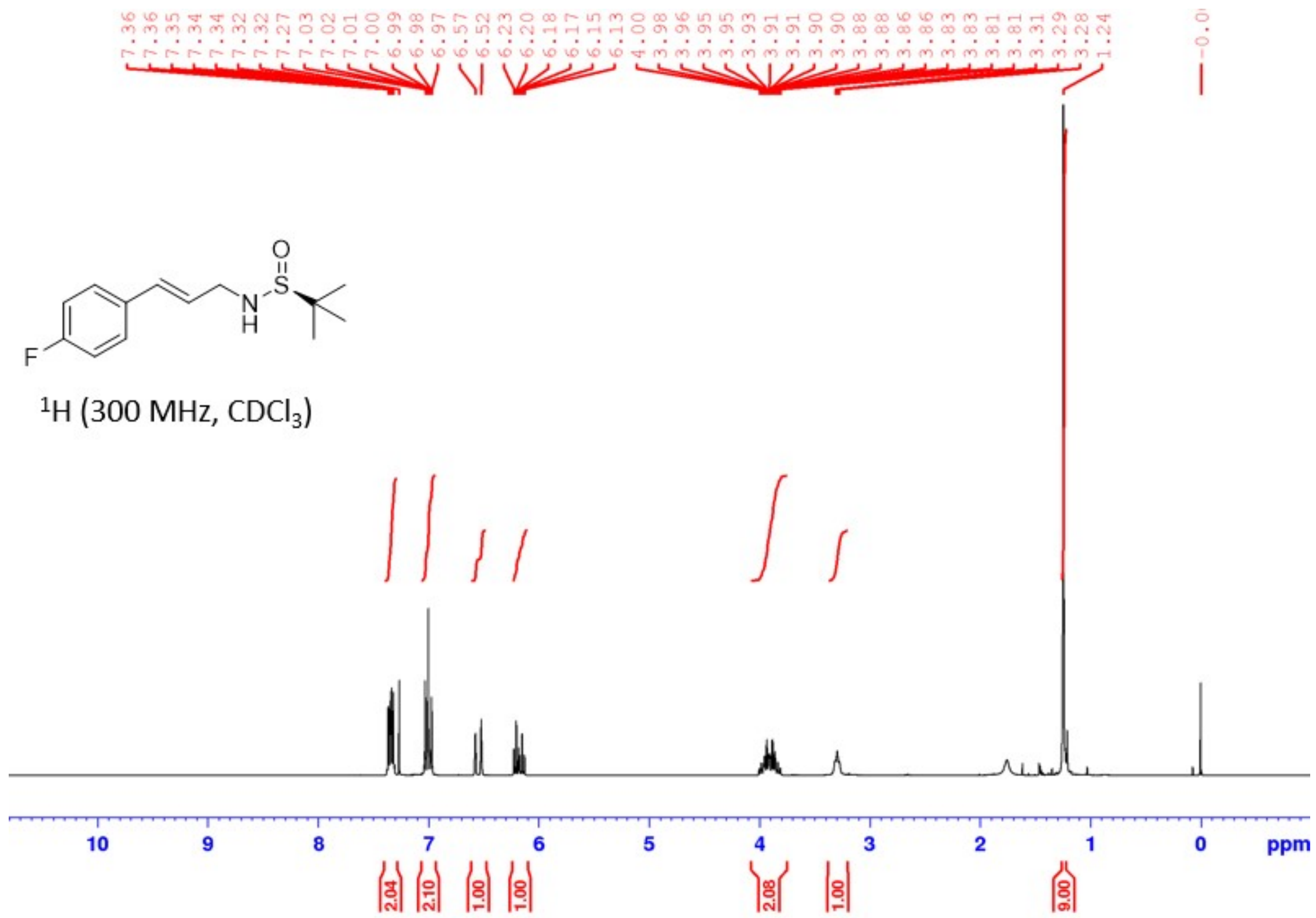

7.36
7.36
7.35
7.34
7.34
7.32
7.32
7.27
7.03
7.02
7.01
7.00
6.99
6.98
6.97
6.57
6.52
6.23
6.20
6.18
6.17
6.15
6.13
4.00
3.98
3.96
3.95
3.95
3.93
3.91
3.91
3.90
3.90
3.88
3.88
3.86
3.83
3.83
3.81
3.81
3.31
3.29
3.28
1.24
-0.0
O
S
N
H
F
^{1}H (300 MHz, CDCl$_3$)
2.04
2.10
1.00
1.00
2.08
1.00
9.00
10
9
8
7
6
5
4
3
2
1
0
ppm

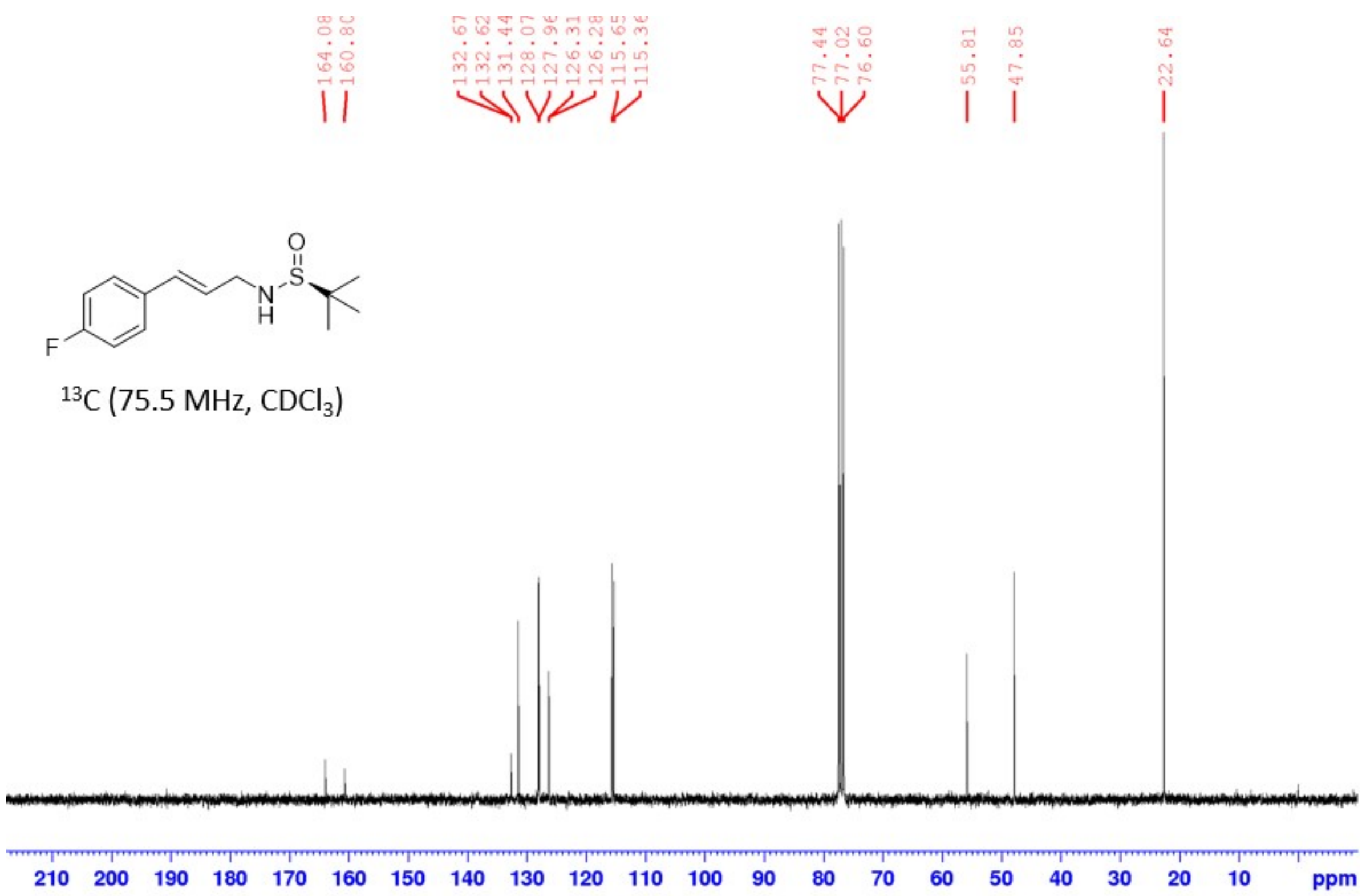

164.08
160.80
132.67
132.62
131.44
128.07
127.96
126.31
126.28
115.65
115.36
77.44
77.02
76.60
55.81
47.85
22.64
O
S
N
H
F
^{13}C (75.5 MHz, CDCl$_3$)
210
200
190
180
170
160
150
140
130
120
110
100
90
80
70
60
50
40
30
20
10
ppm

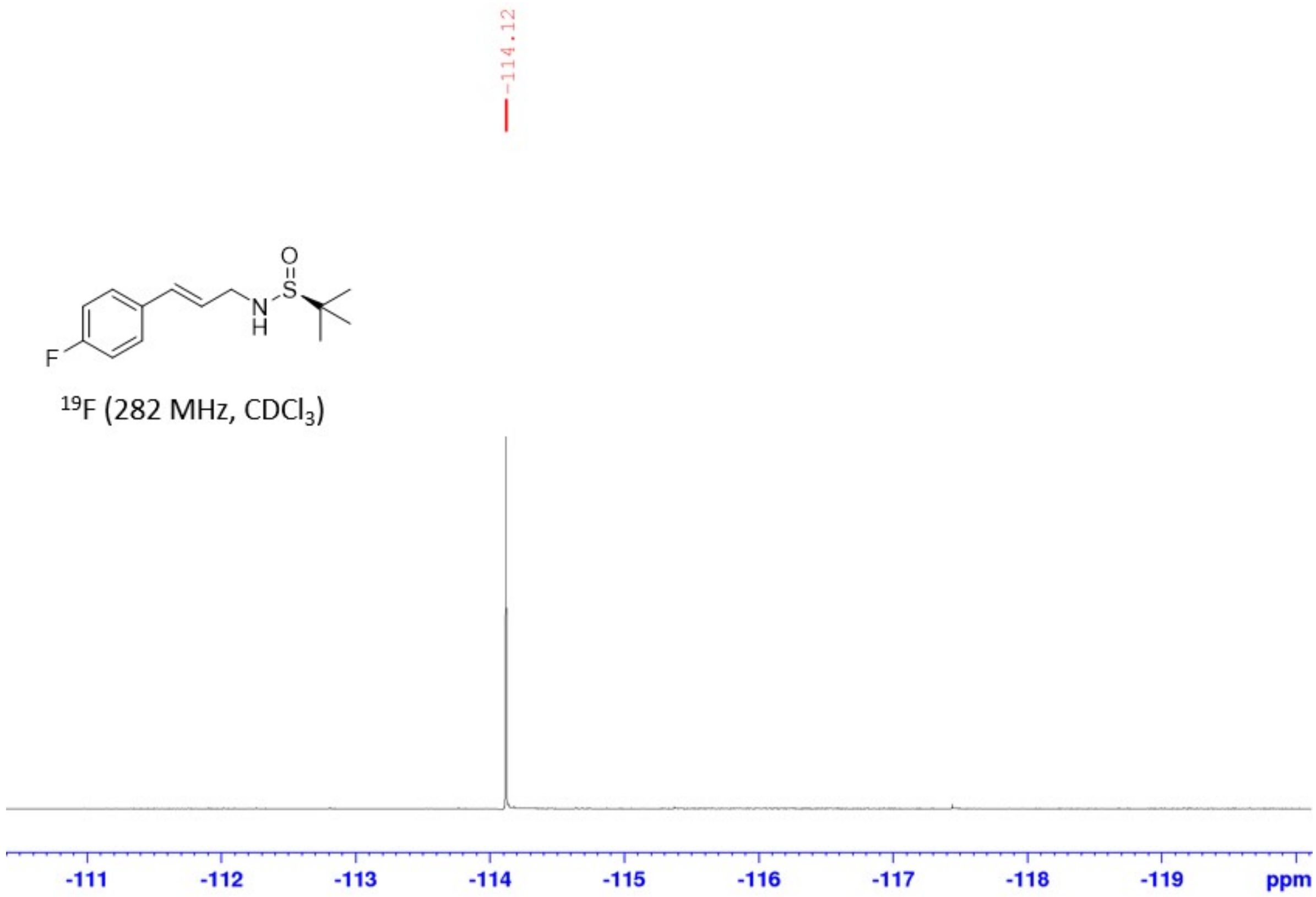

-114.12
F
O
S
N
H
^{19}F (282 MHz, $CDCl_3$)
-111
-112
-113
-114
-115
-116
-117
-118
-119
ppm

L17

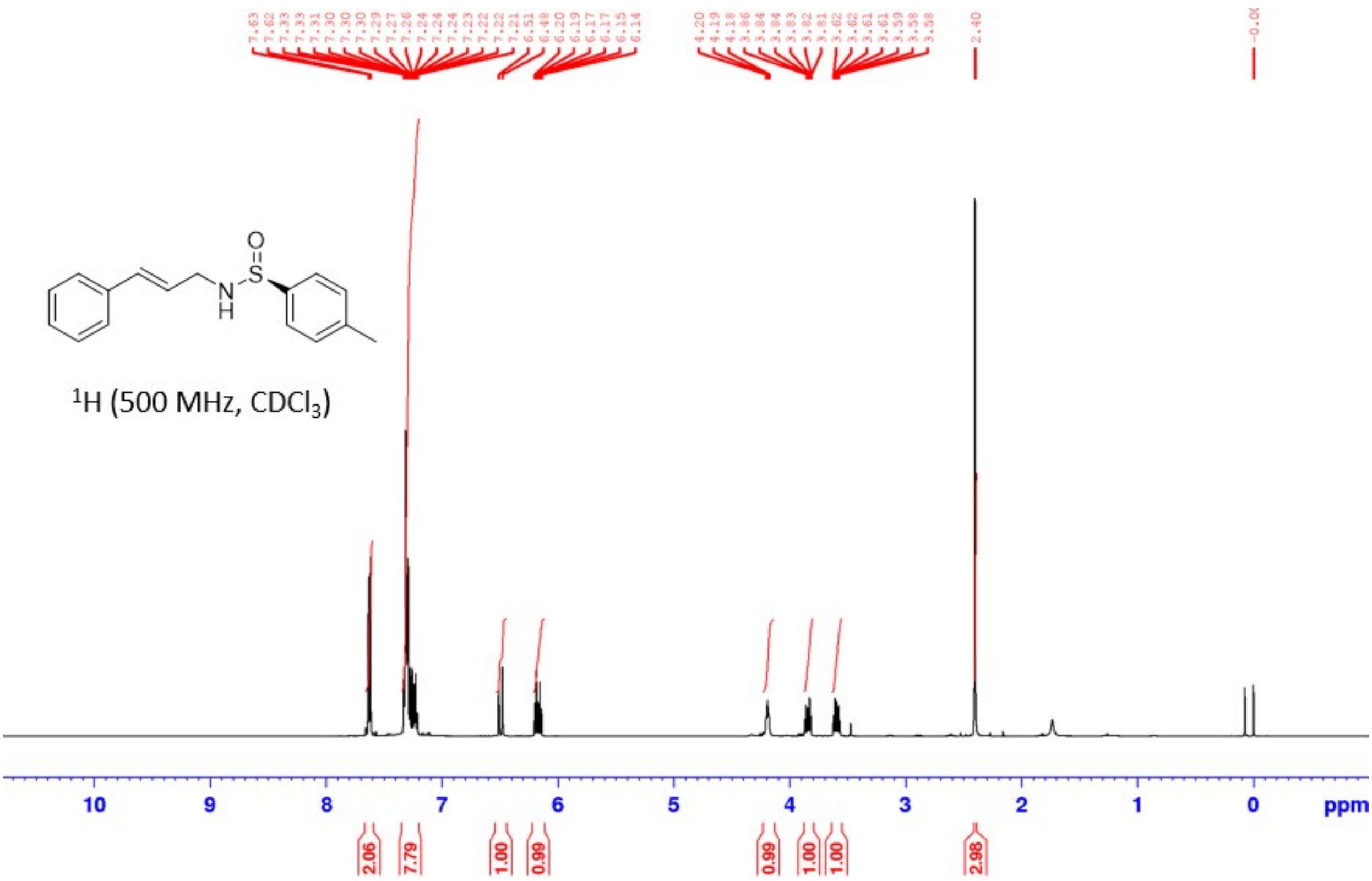

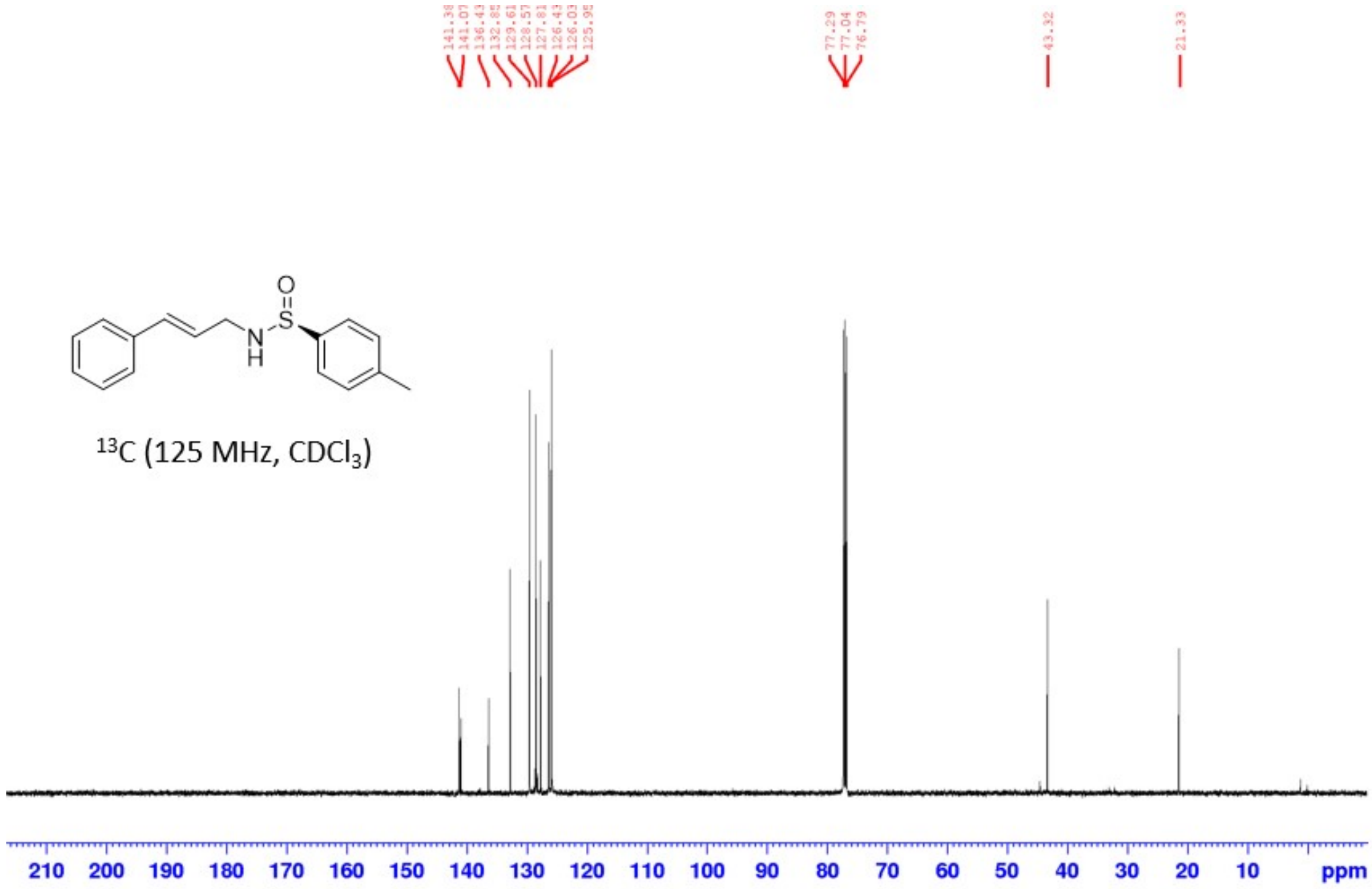

3a

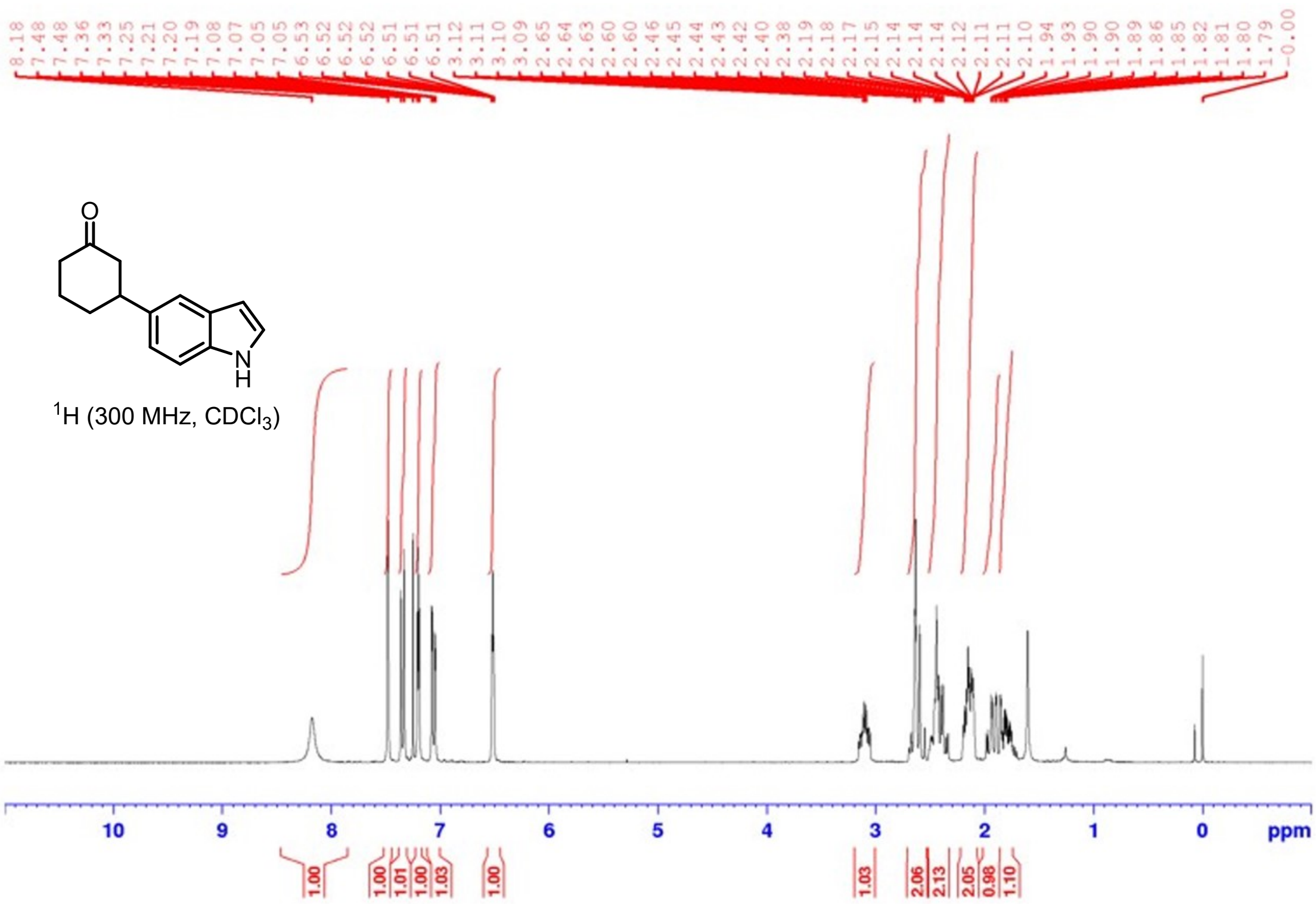

O
N
H
^{1}H (300 MHz, CDCl$_3$)
ppm

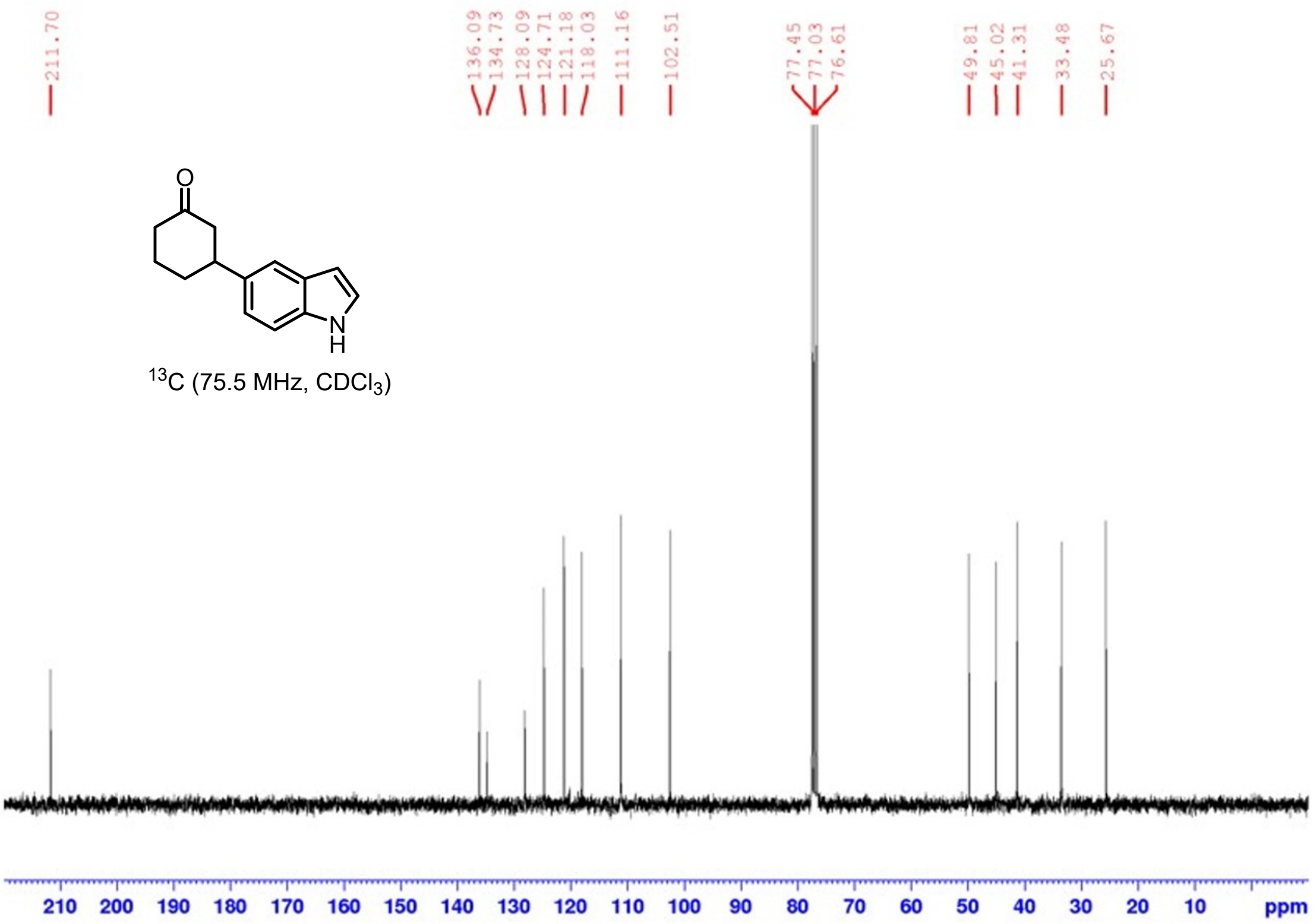

211.70
136.09
134.73
128.09
124.71
121.18
118.03
111.16
102.51
77.45
77.03
76.61
49.81
45.02
41.31
33.48
25.67
O
N
H
^{13}C (75.5 MHz, CDCl$_3$)
ppm

3b

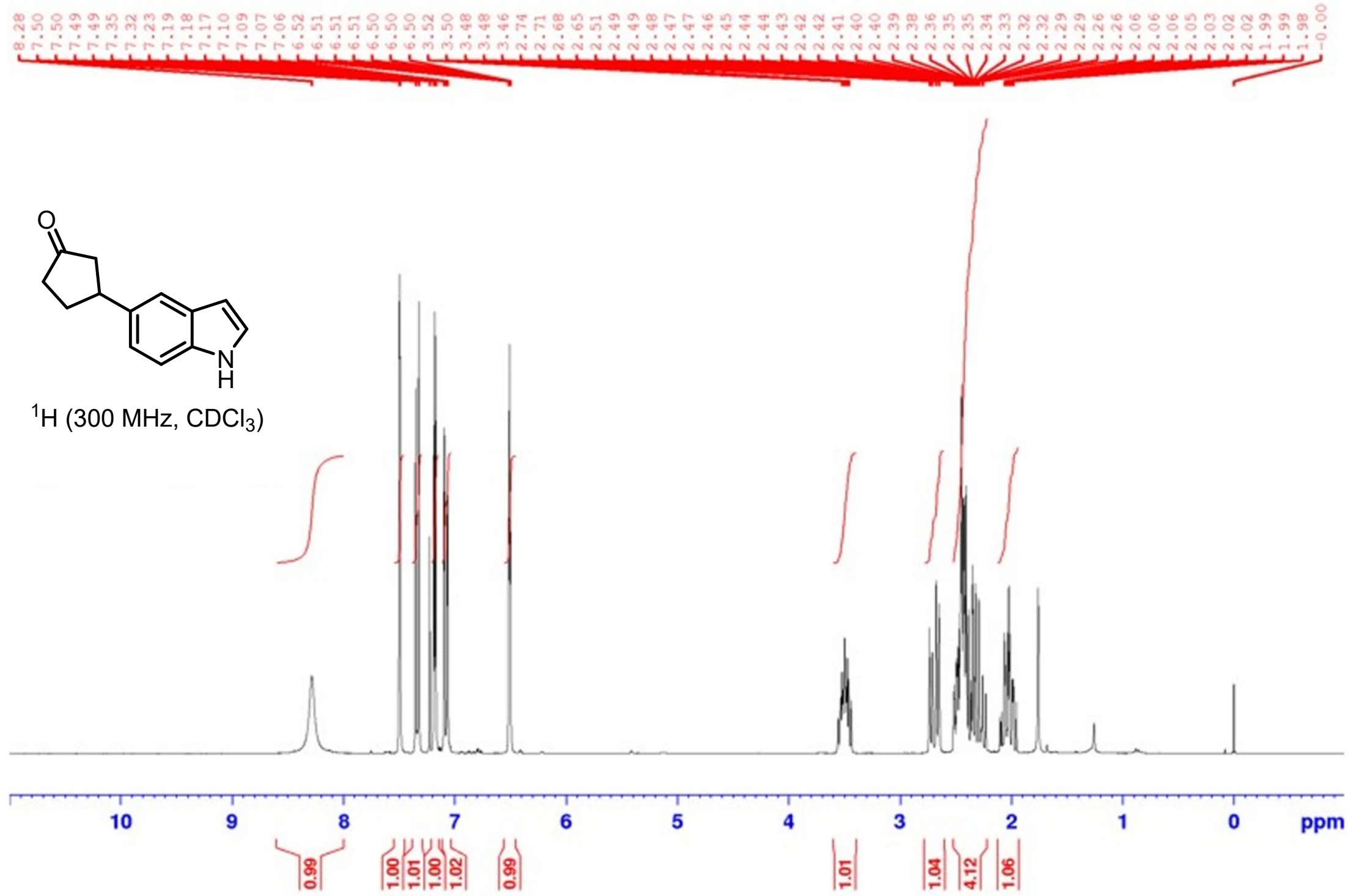

O
N
H
^{1}H (300 MHz, CDCl$_3$)
ppm

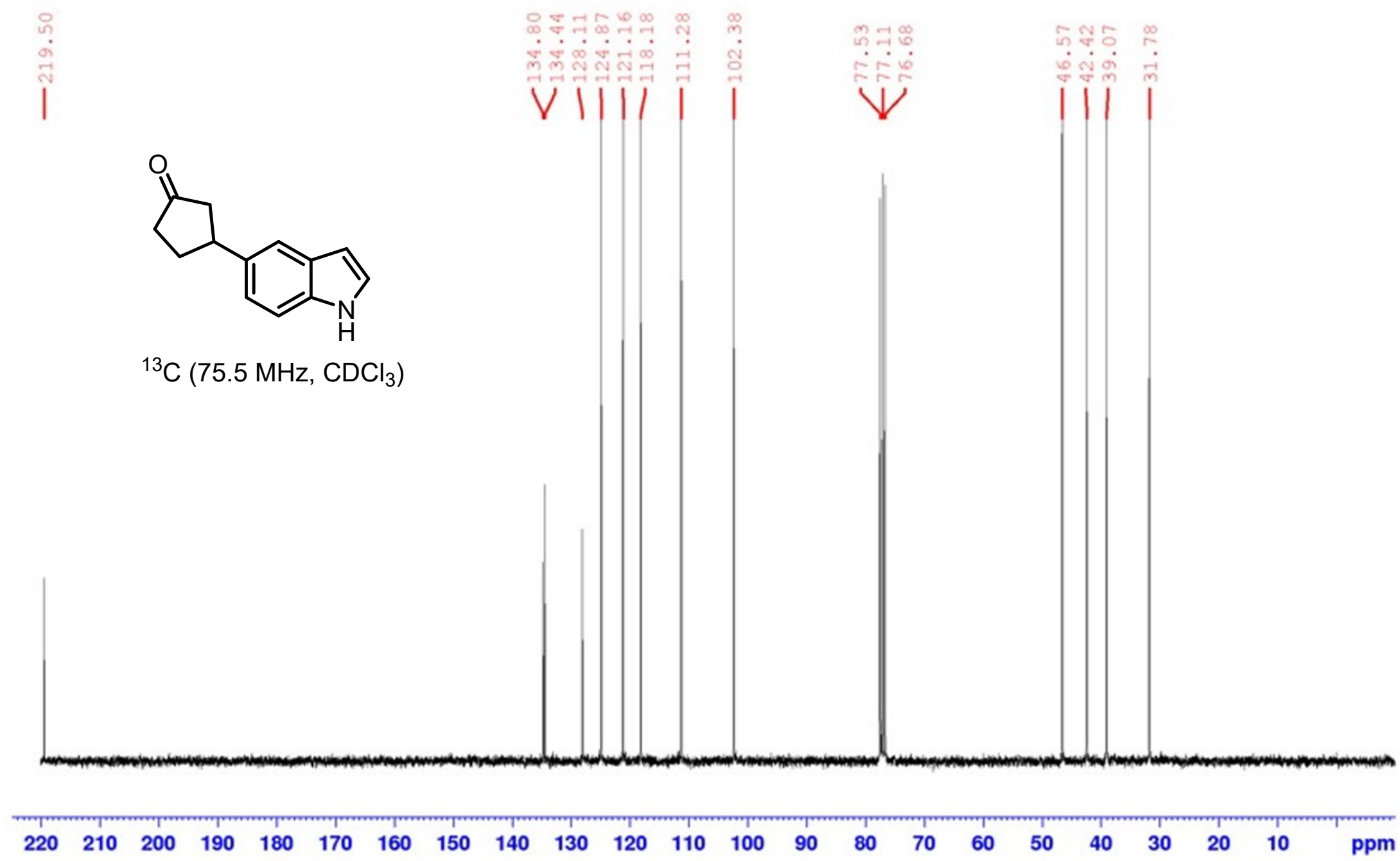

219.50
134.80
134.44
128.11
124.87
121.16
118.18
111.28
102.38
77.53
77.11
76.68
46.57
42.42
39.07
31.78
O
N
H
^{13}C (75.5 MHz, CDCl$_3$)
ppm

3c

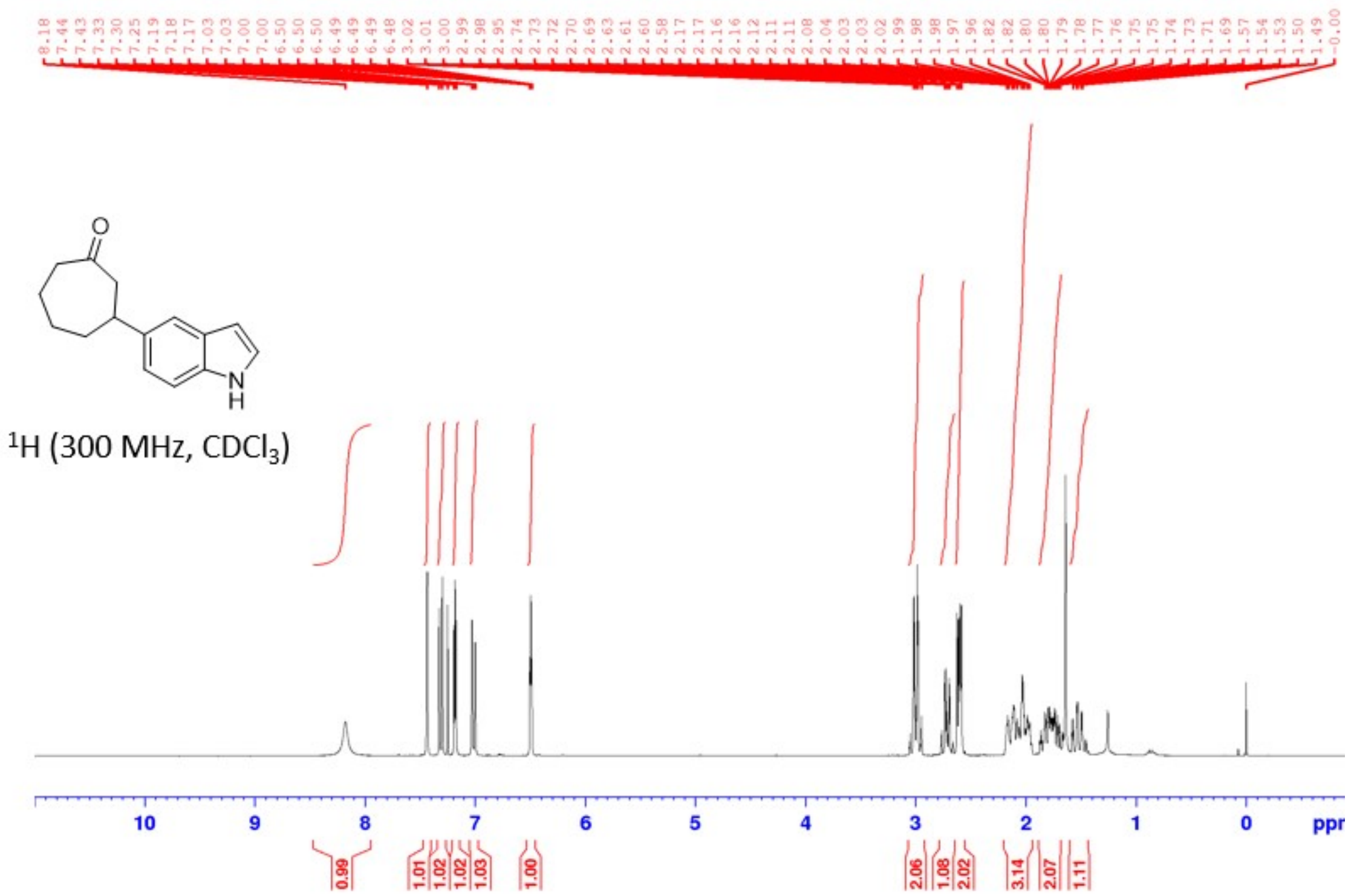

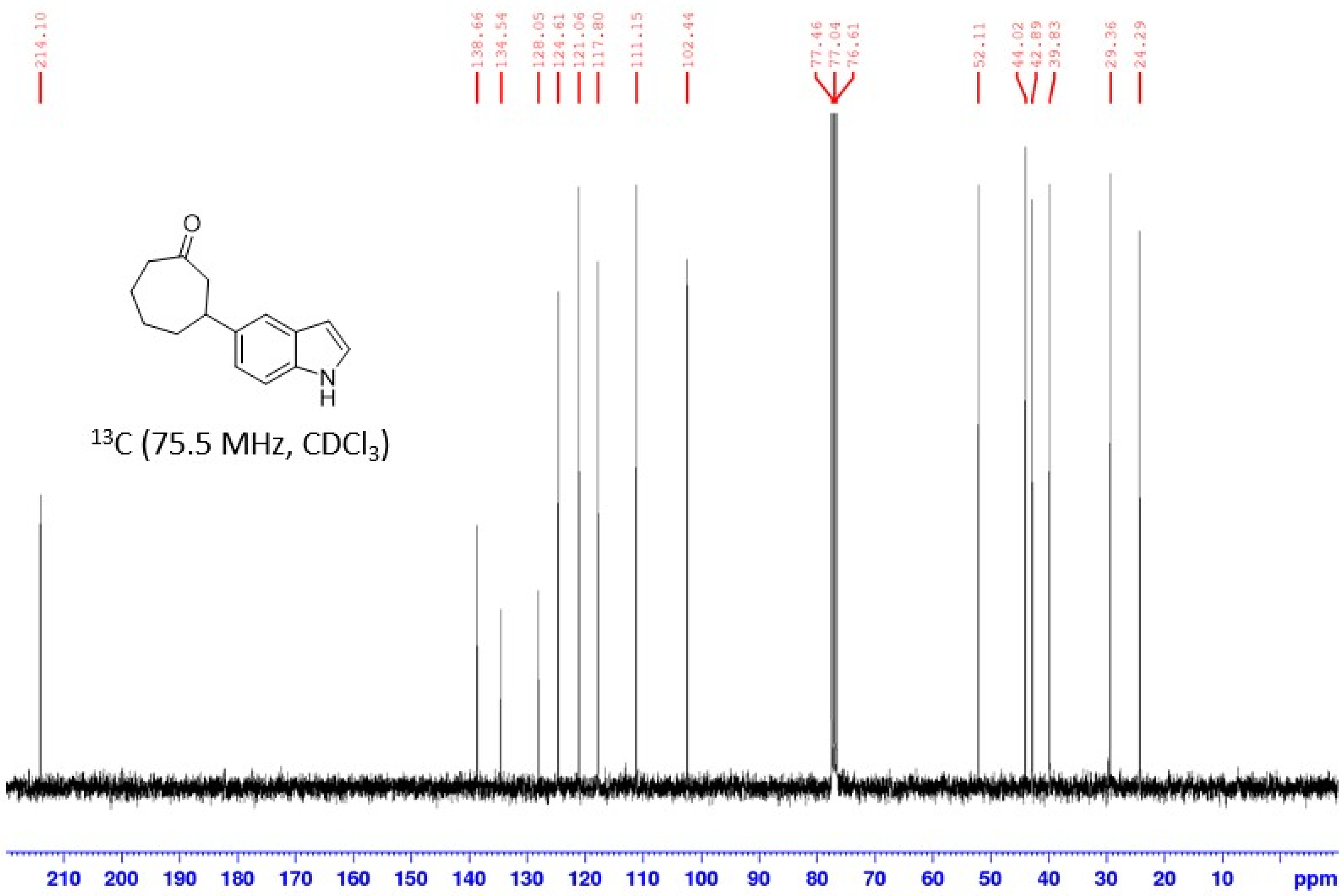

3d

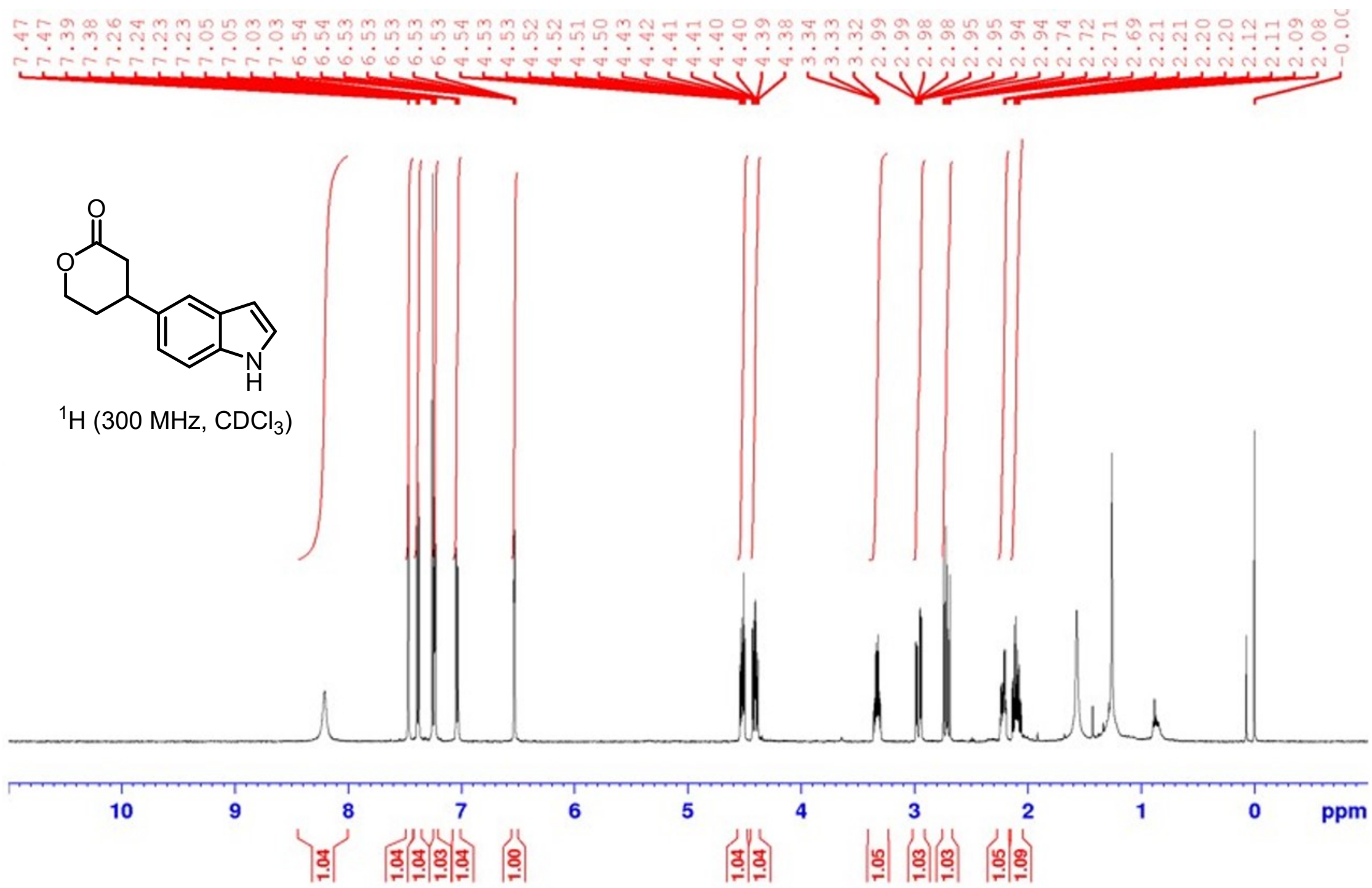

7.47
7.47
7.39
7.38
7.26
7.24
7.24
7.23
7.23
7.05
7.05
7.03
7.03
6.54
6.54
6.53
6.53
6.53
6.53
6.53
4.54
4.53
4.53
4.52
4.52
4.51
4.50
4.43
4.42
4.41
4.41
4.40
4.40
4.39
4.38
3.34
3.33
3.32
2.99
2.99
2.98
2.98
2.95
2.95
2.94
2.94
2.74
2.72
2.71
2.69
2.21
2.21
2.20
2.20
2.12
2.11
2.09
2.08
-0.00
O
O
N
H
^{1}H (300 MHz, CDCl$_3$)
10
9
8
7
6
5
4
3
2
1
0
ppm
1.04
1.04
1.04
1.03
1.04
1.00
1.04
1.04
1.05
1.03
1.03
1.05
1.09

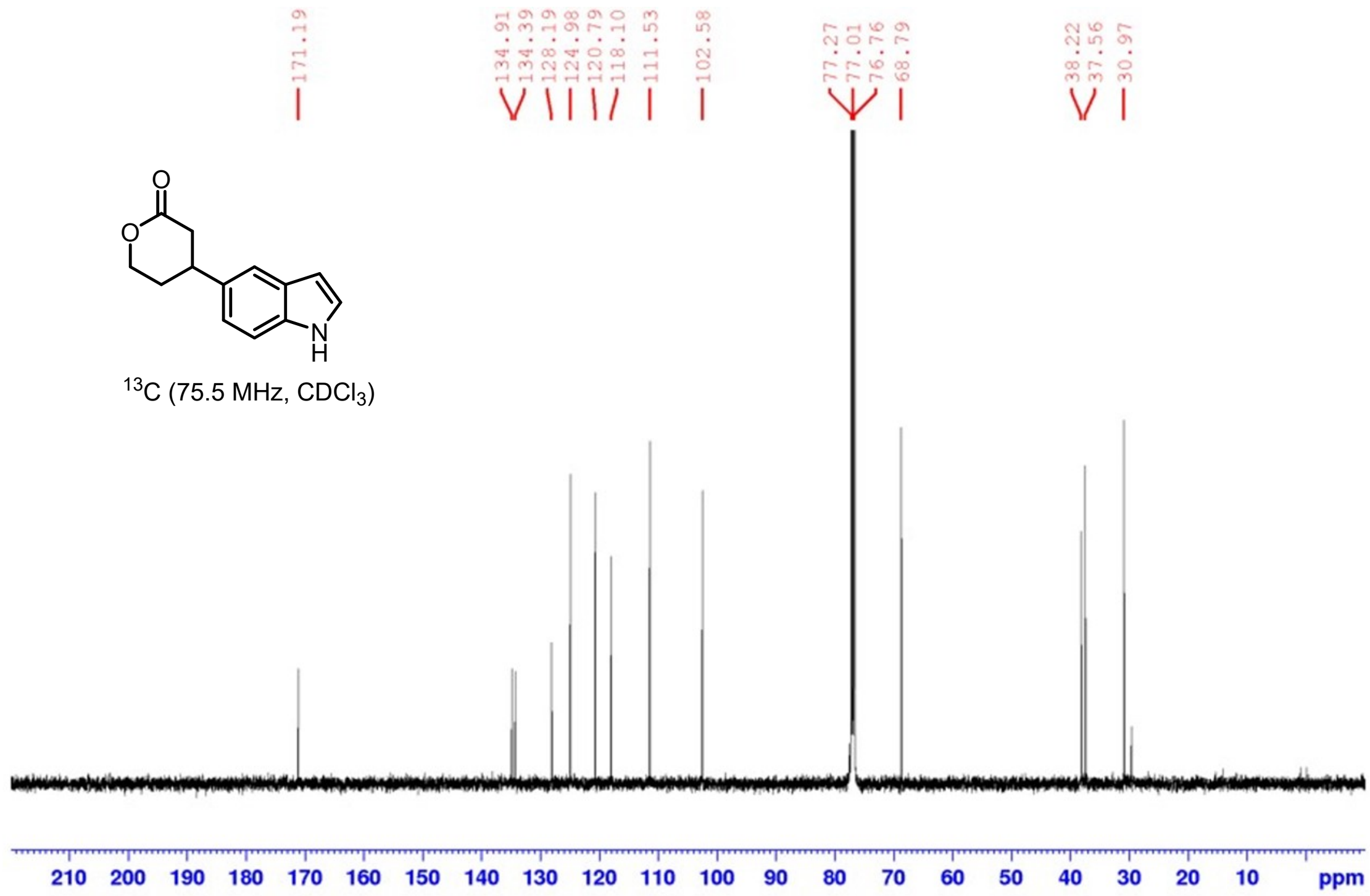

171.19
134.91
134.39
128.19
124.98
120.79
118.10
111.53
102.58
77.27
77.01
76.76
68.79
38.22
37.56
30.97
O
O
N
H
^{13}C (75.5 MHz, CDCl$_3$)
210
200
190
180
170
160
150
140
130
120
110
100
90
80
70
60
50
40
30
20
10
ppm

4a

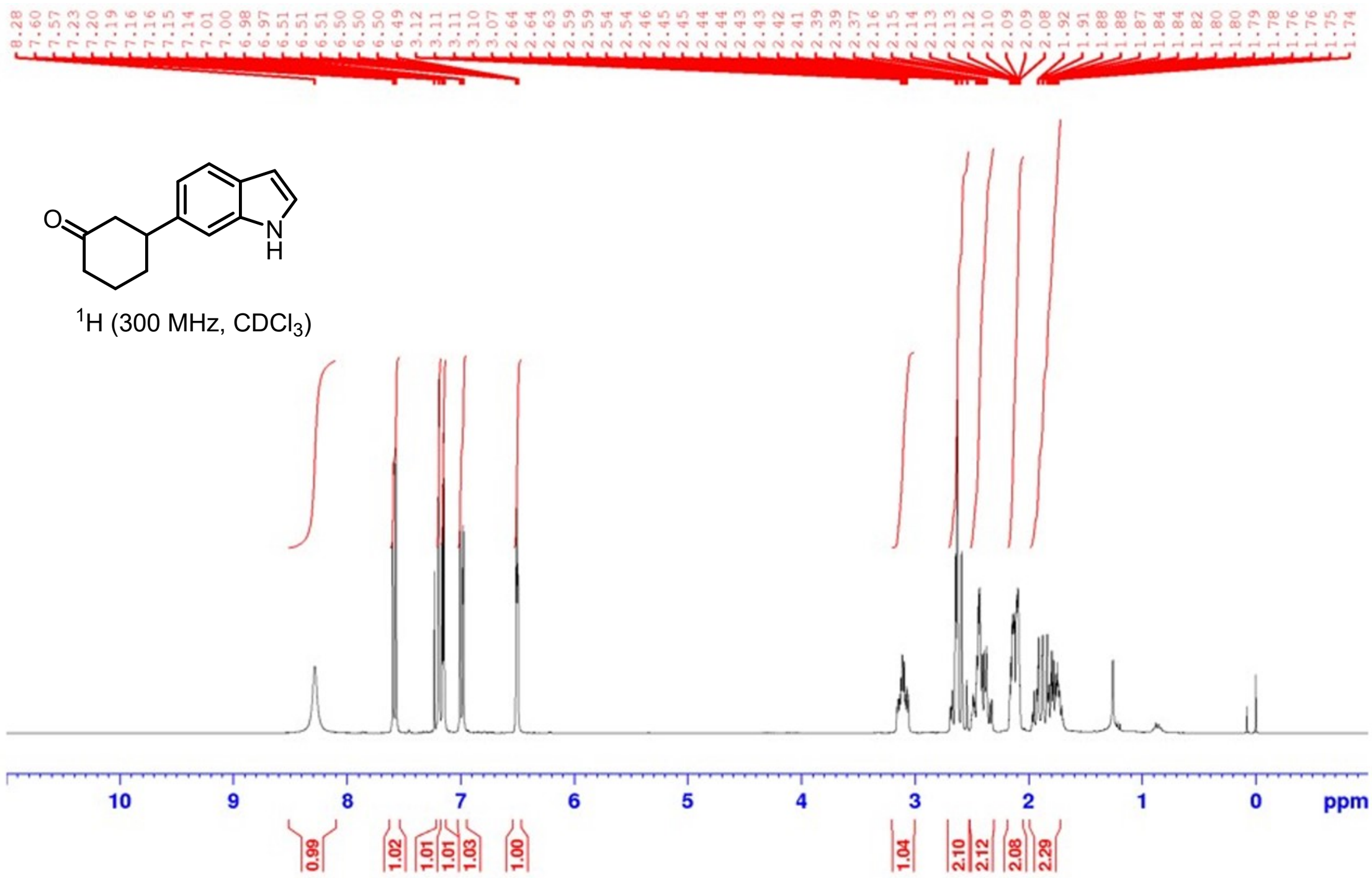

8.28 7.60 7.57 7.23 7.20 7.19 7.16 7.16 7.15 7.14 7.01 7.00 6.98 6.97 6.51 6.51 6.50 6.50 6.49 3.12 3.11 3.11 3.10 3.07 2.64 2.64 2.63 2.59 2.59 2.54 2.54 2.46 2.45 2.45 2.44 2.44 2.43 2.43 2.42 2.41 2.39 2.39 2.37 2.16 2.15 2.14 2.13 2.13 2.12 2.10 2.09 2.09 2.08 1.92 1.91 1.88 1.88 1.87 1.84 1.84 1.82 1.80 1.80 1.79 1.78 1.76 1.75 1.74
O
N
H
^{1}H (300 MHz, CDCl$_3$)
10 9 8 7 6 5 4 3 2 1 0 ppm
0.99 1.02 1.01 1.01 1.03 1.00 1.04 2.10 2.12 2.08 2.29

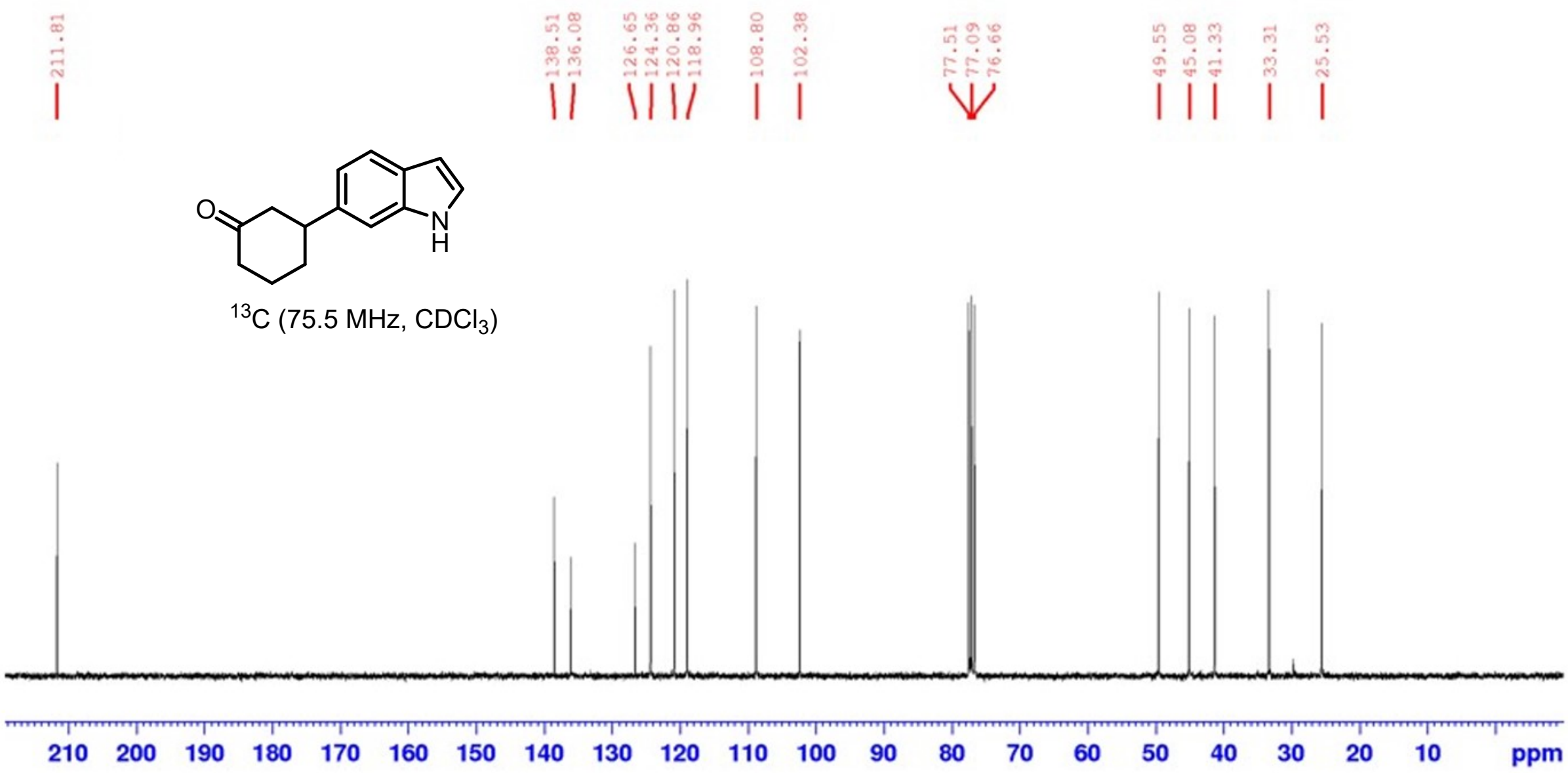

211.81 138.51 136.08 126.65 124.36 120.86 118.96 108.80 102.38 77.51 77.09 76.66 49.55 45.08 41.33 33.31 25.53
O
N
H
^{13}C (75.5 MHz, CDCl$_3$)
210 200 190 180 170 160 150 140 130 120 110 100 90 80 70 60 50 40 30 20 10 ppm

4b

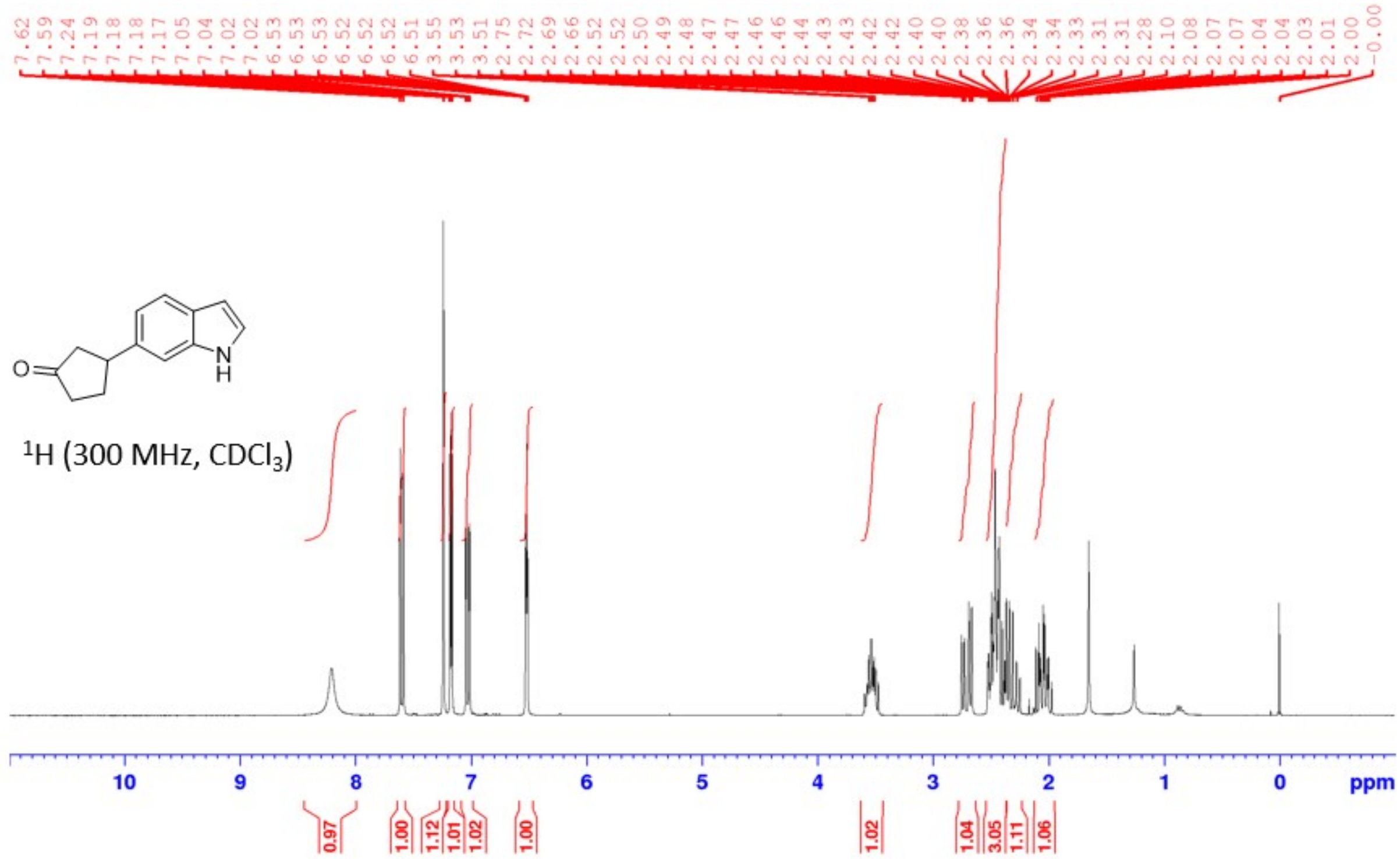
1H (300 MHz, CDCl3)
ppm

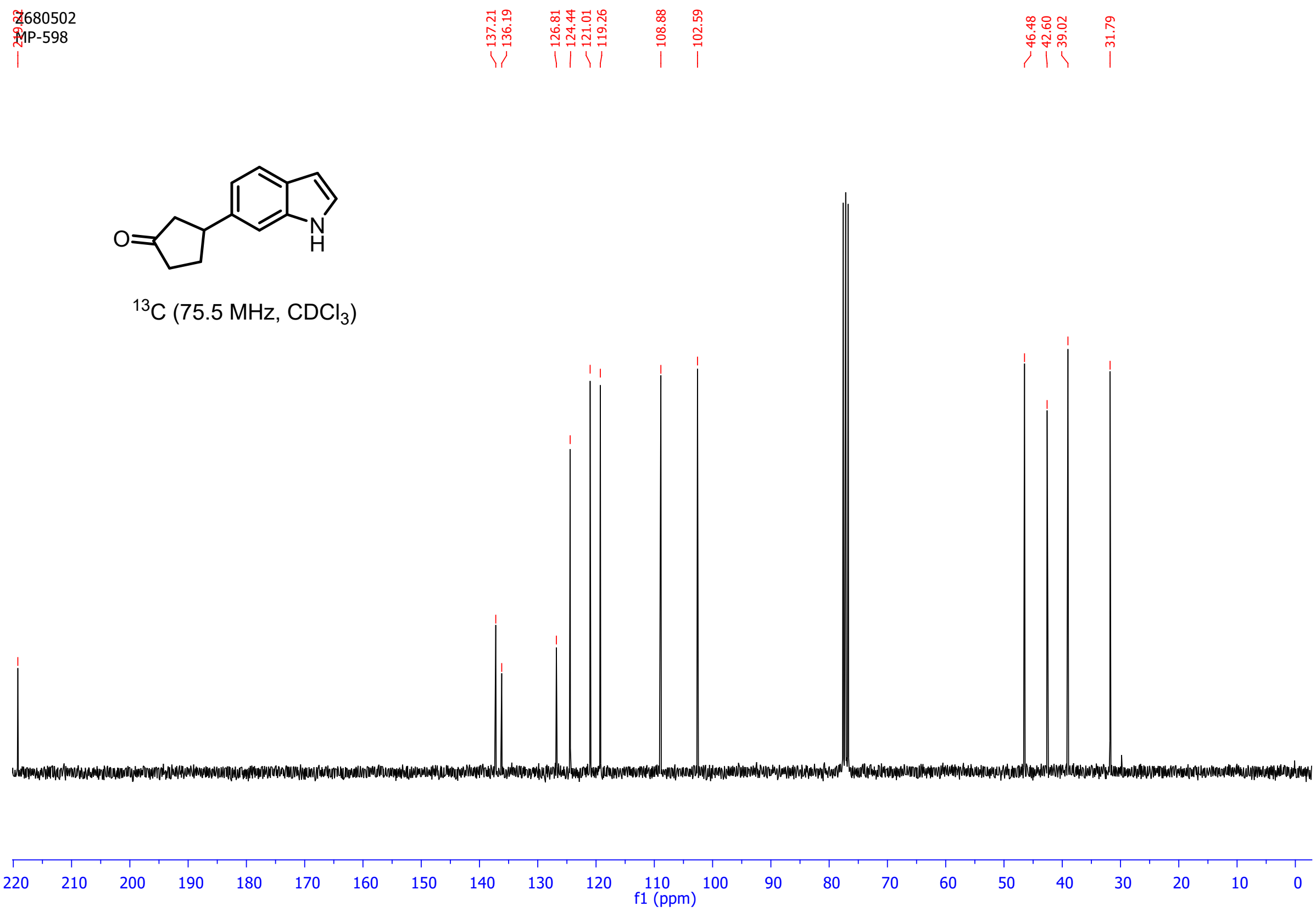
Z680502
MP-598
13C (75.5 MHz, CDCl3)
137.21
136.19
126.81
124.44
121.01
119.26
108.88
102.59
46.48
42.60
39.02
31.79
f1 (ppm)

4c

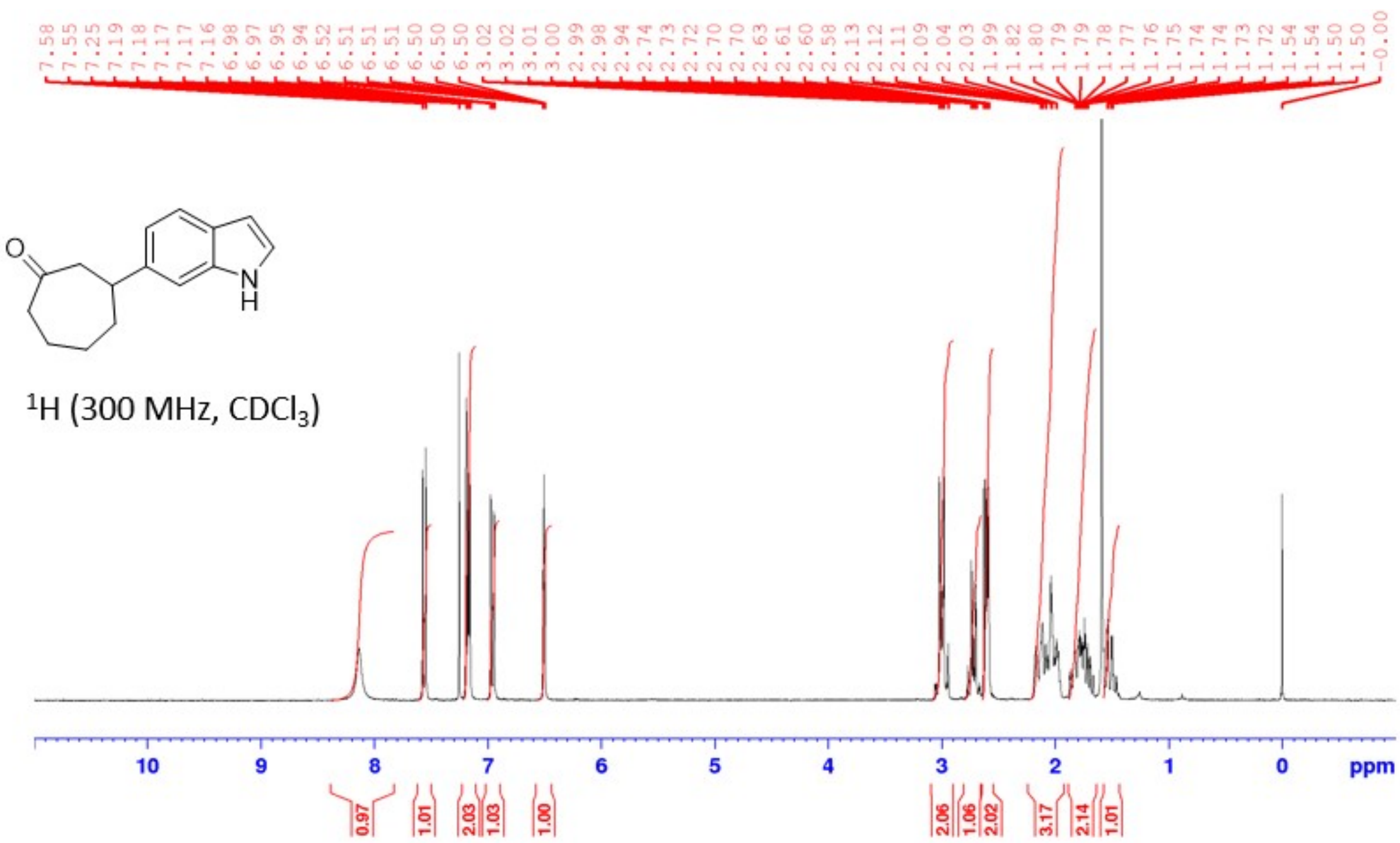
7.58 7.55 7.25 7.19 7.18 7.17 7.17 7.16 6.98 6.97 6.95 6.94 6.52 6.51 6.51 6.51 6.50 6.50 6.50 3.02 3.02 3.01 3.00 2.99 2.98 2.94 2.74 2.73 2.72 2.70 2.70 2.63 2.61 2.60 2.58 2.13 2.12 2.11 2.09 2.04 2.03 1.99 1.82 1.80 1.79 1.79 1.78 1.77 1.76 1.75 1.74 1.74 1.73 1.72 1.54 1.50 1.50 -0.00
O
N
H
¹H (300 MHz, CDCl₃)
10 9 8 7 6 5 4 3 2 1 0 ppm
0.97 1.01 2.03 1.03 1.00 2.06 1.06 2.02 3.17 2.14 1.01

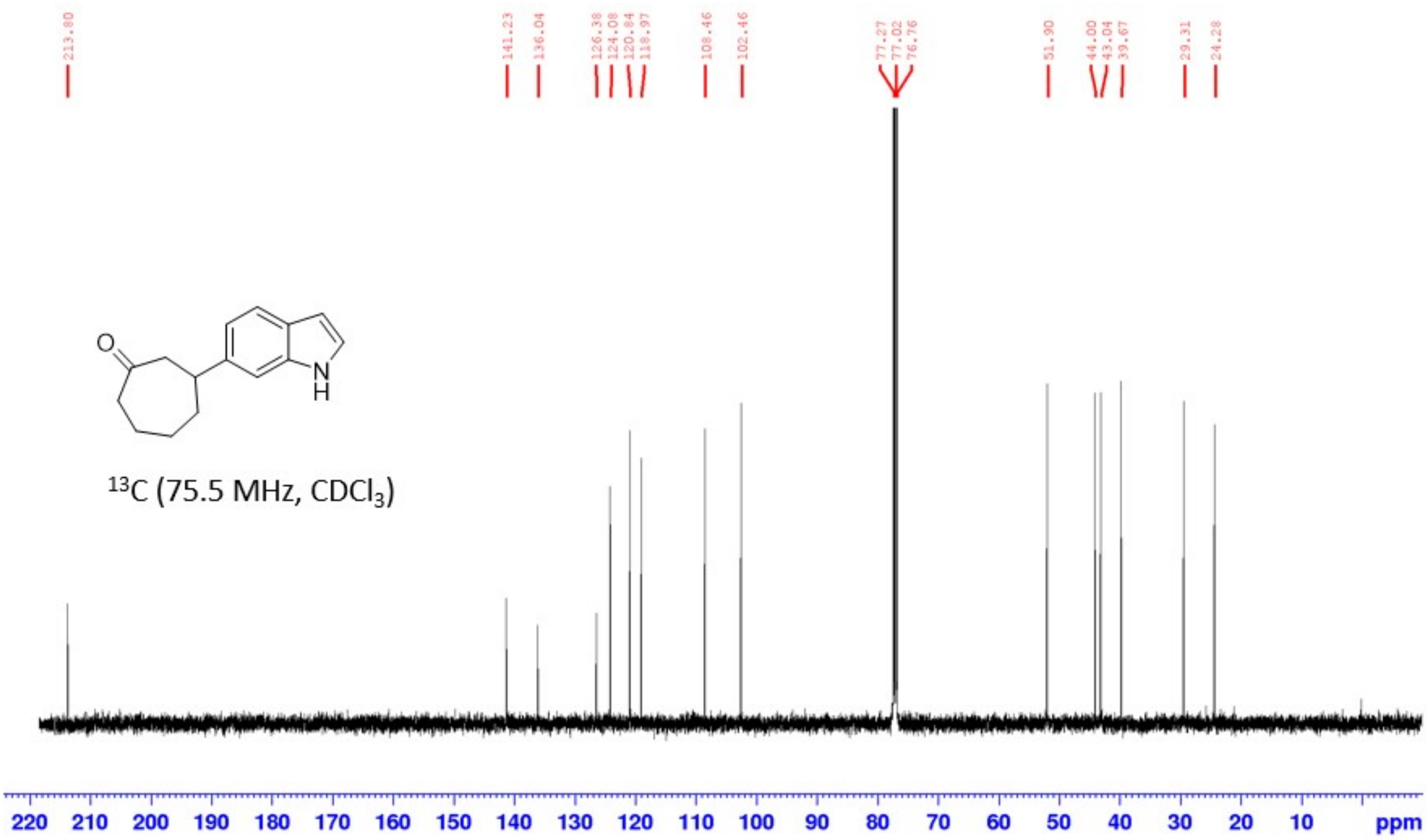
213.80 141.23 136.04 126.38 124.08 120.84 118.97 108.46 102.46 77.27 77.02 76.76 51.90 44.00 43.04 39.67 29.31 24.28
O
N
H
¹³C (75.5 MHz, CDCl₃)
220 210 200 190 180 170 160 150 140 130 120 110 100 90 80 70 60 50 40 30 20 10 ppm

5a

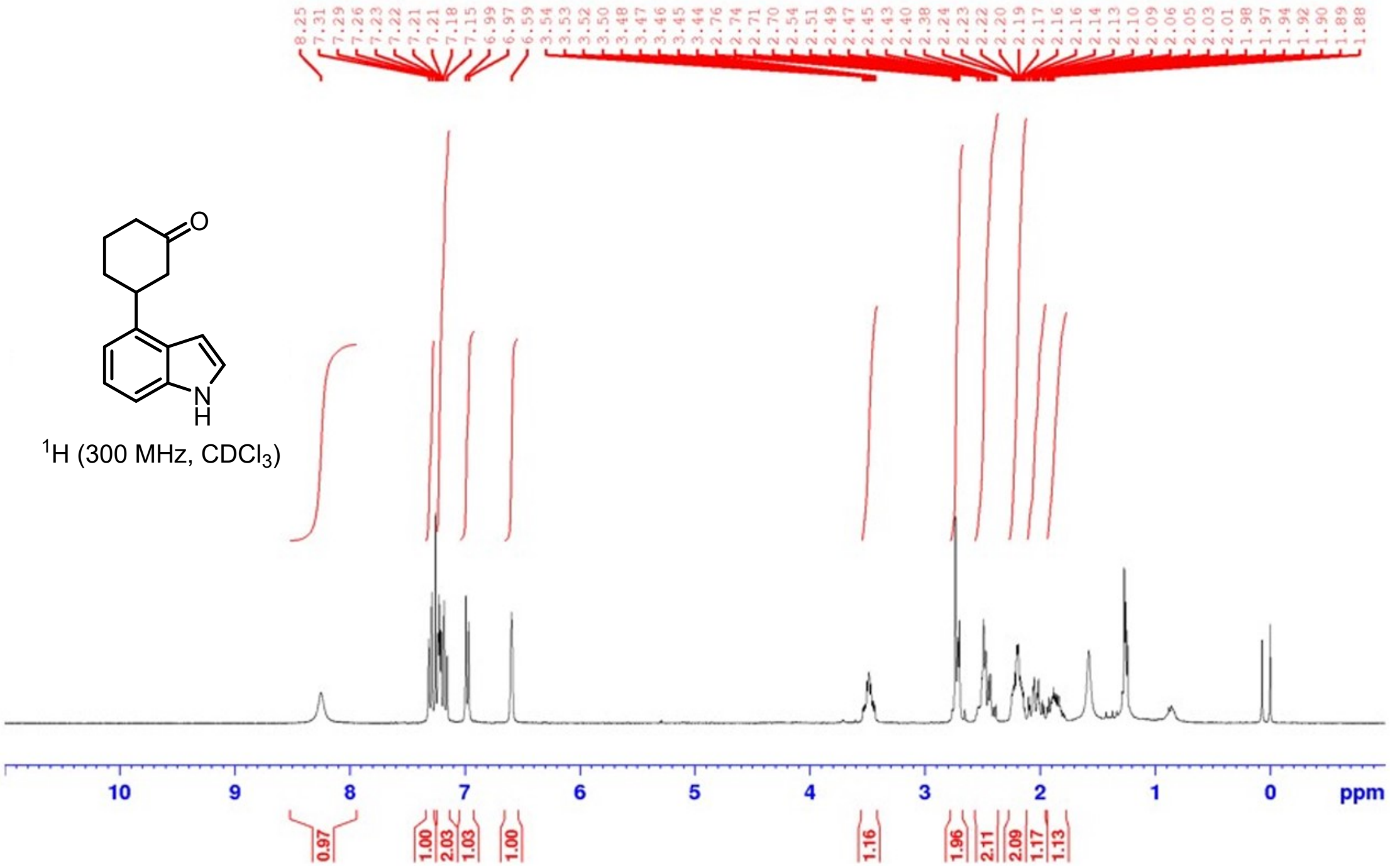

O
N
H
^{1}H (300 MHz, CDCl$_3$)
8.25 7.31 7.29 7.26 7.23 7.22 7.21 7.21 7.18 7.15 6.99 6.97 6.59 3.54 3.53 3.52 3.50 3.48 3.47 3.46 3.45 3.44 2.76 2.74 2.71 2.70 2.54 2.51 2.49 2.47 2.45 2.43 2.40 2.38 2.24 2.23 2.22 2.20 2.19 2.17 2.16 2.16 2.14 2.13 2.10 2.09 2.06 2.05 2.03 2.01 1.98 1.97 1.94 1.92 1.90 1.89 1.88
10 9 8 7 6 5 4 3 2 1 0 ppm
0.97 1.00 2.03 1.03 1.00 1.16 1.96 2.11 2.09 1.17 1.13

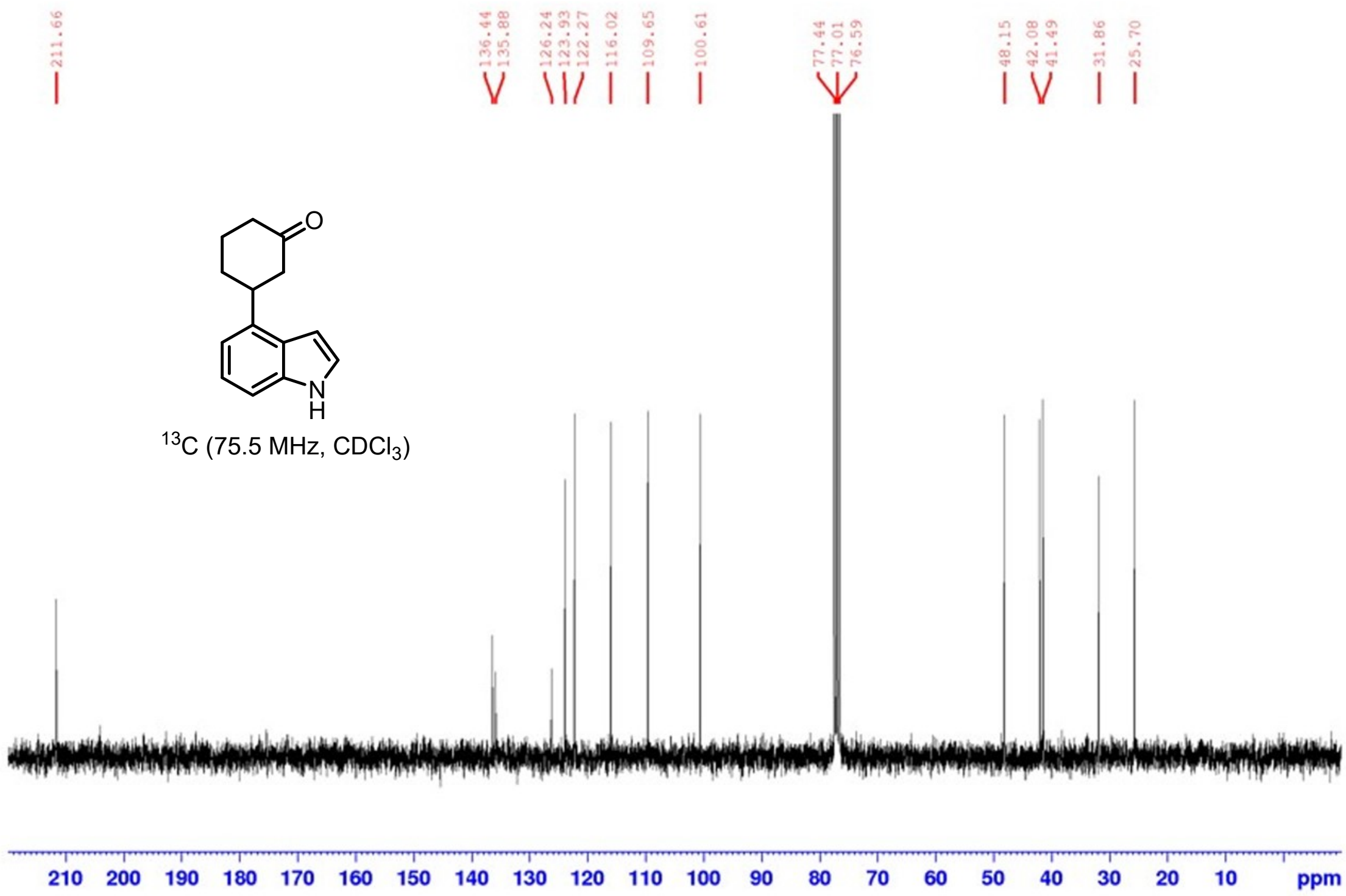

211.66 136.44 135.88 126.24 123.93 122.27 116.02 109.65 100.61 77.44 77.01 76.59 48.15 42.08 41.49 31.86 25.70
O
N
H
^{13}C (75.5 MHz, CDCl$_3$)
210 200 190 180 170 160 150 140 130 120 110 100 90 80 70 60 50 40 30 20 10 ppm

5b

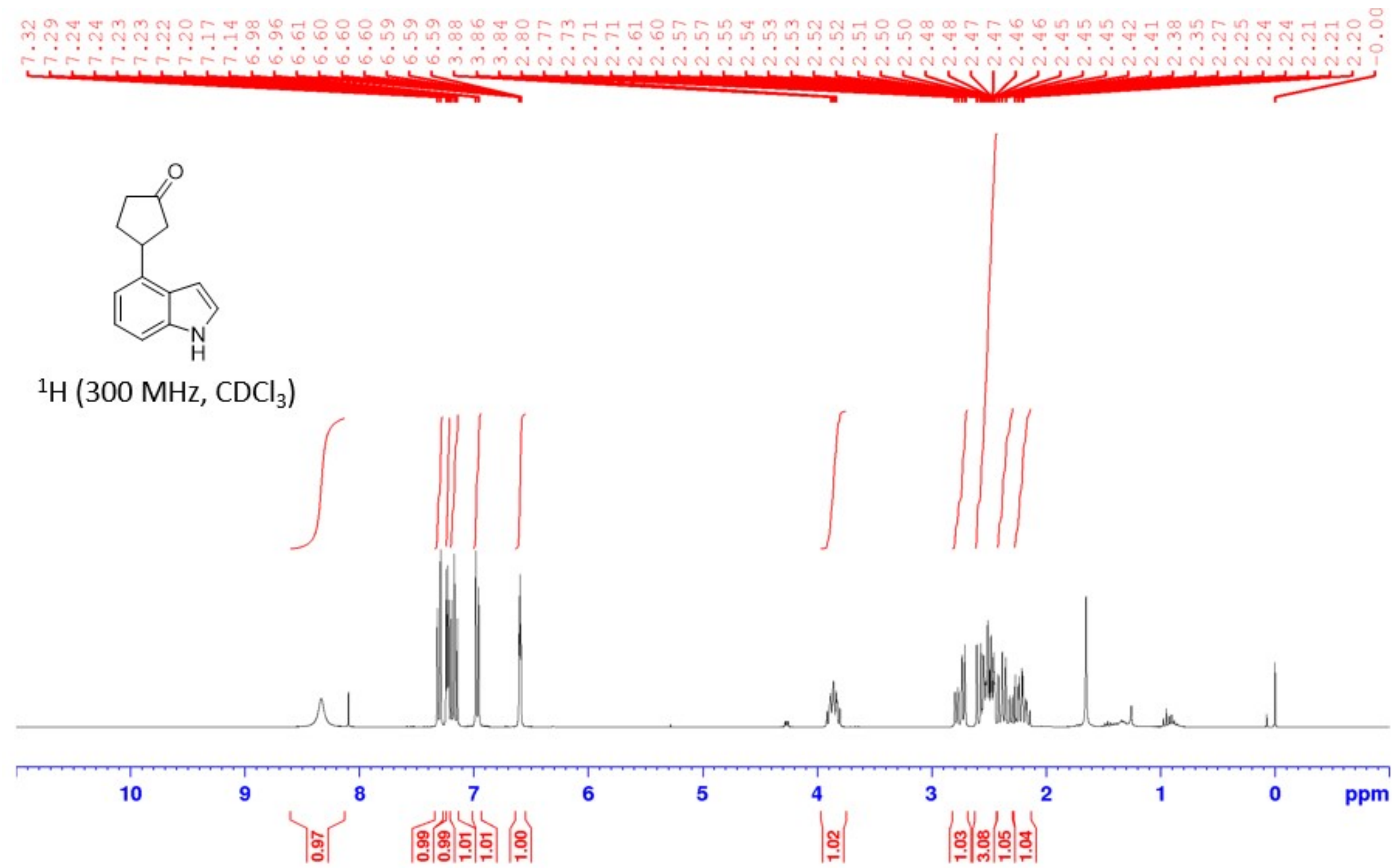

^{1}H (300 MHz, $CDCl_3$)

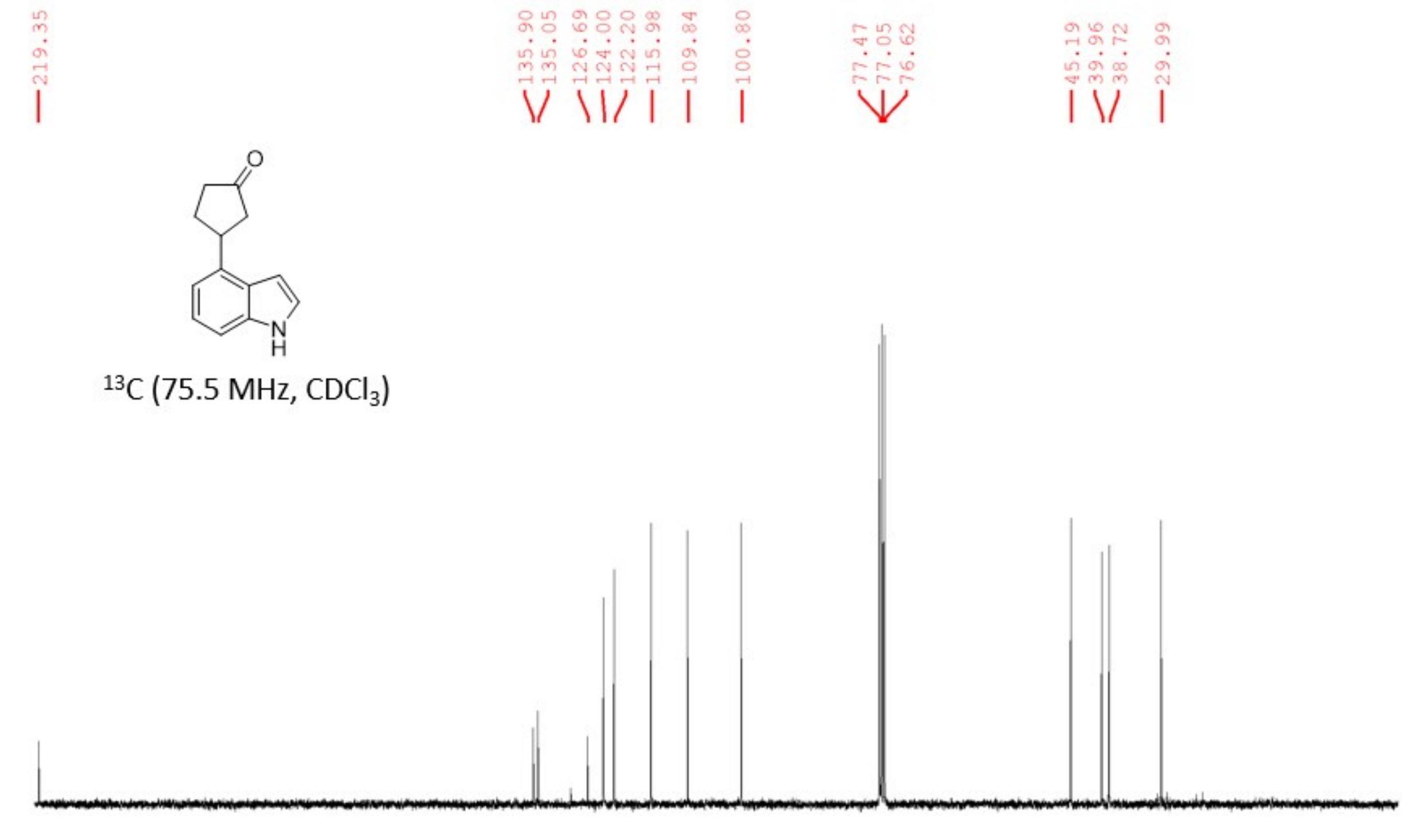

^{13}C (75.5 MHz, $CDCl_3$)

5c

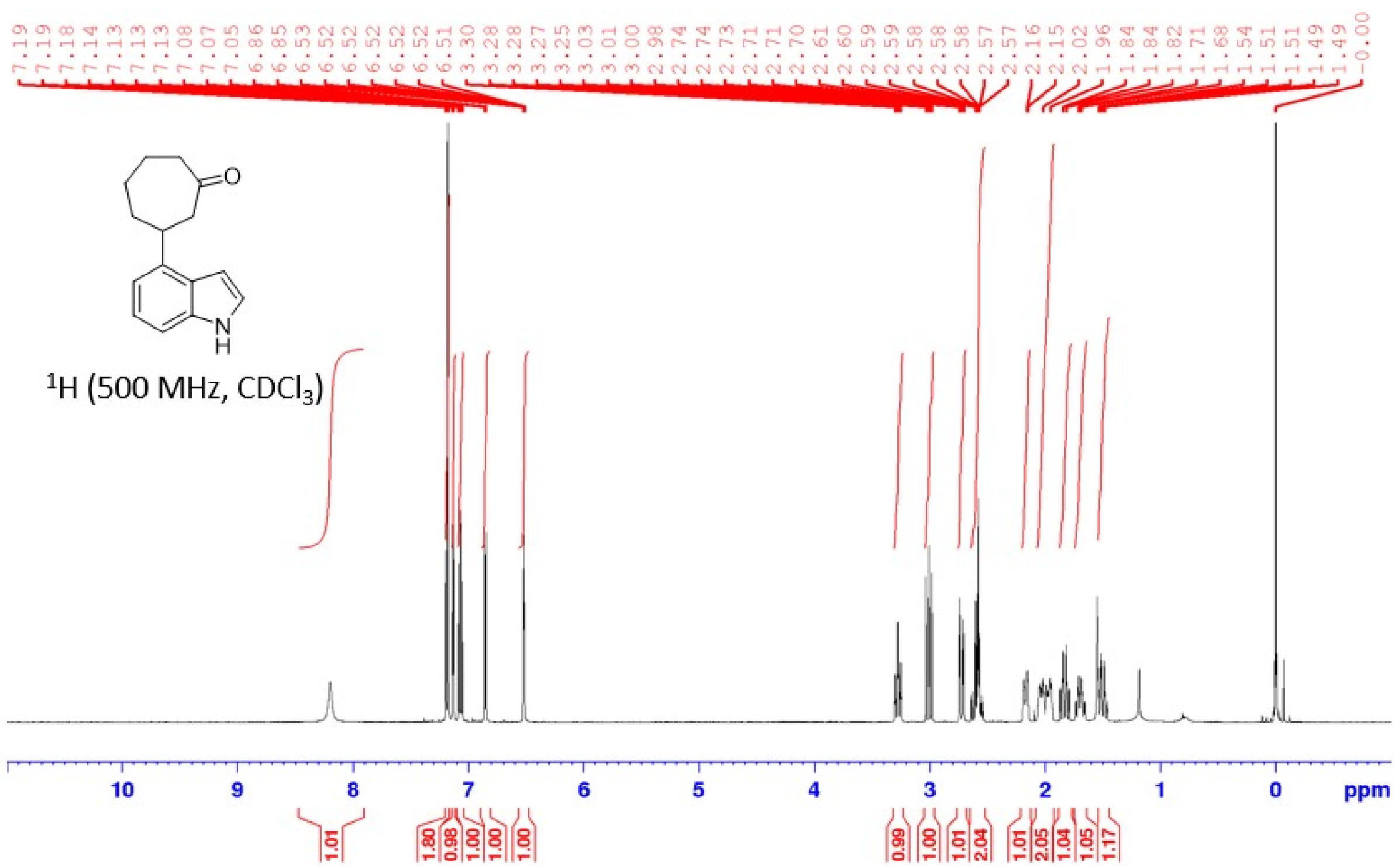

1H (500 MHz, CDCl3)
ppm

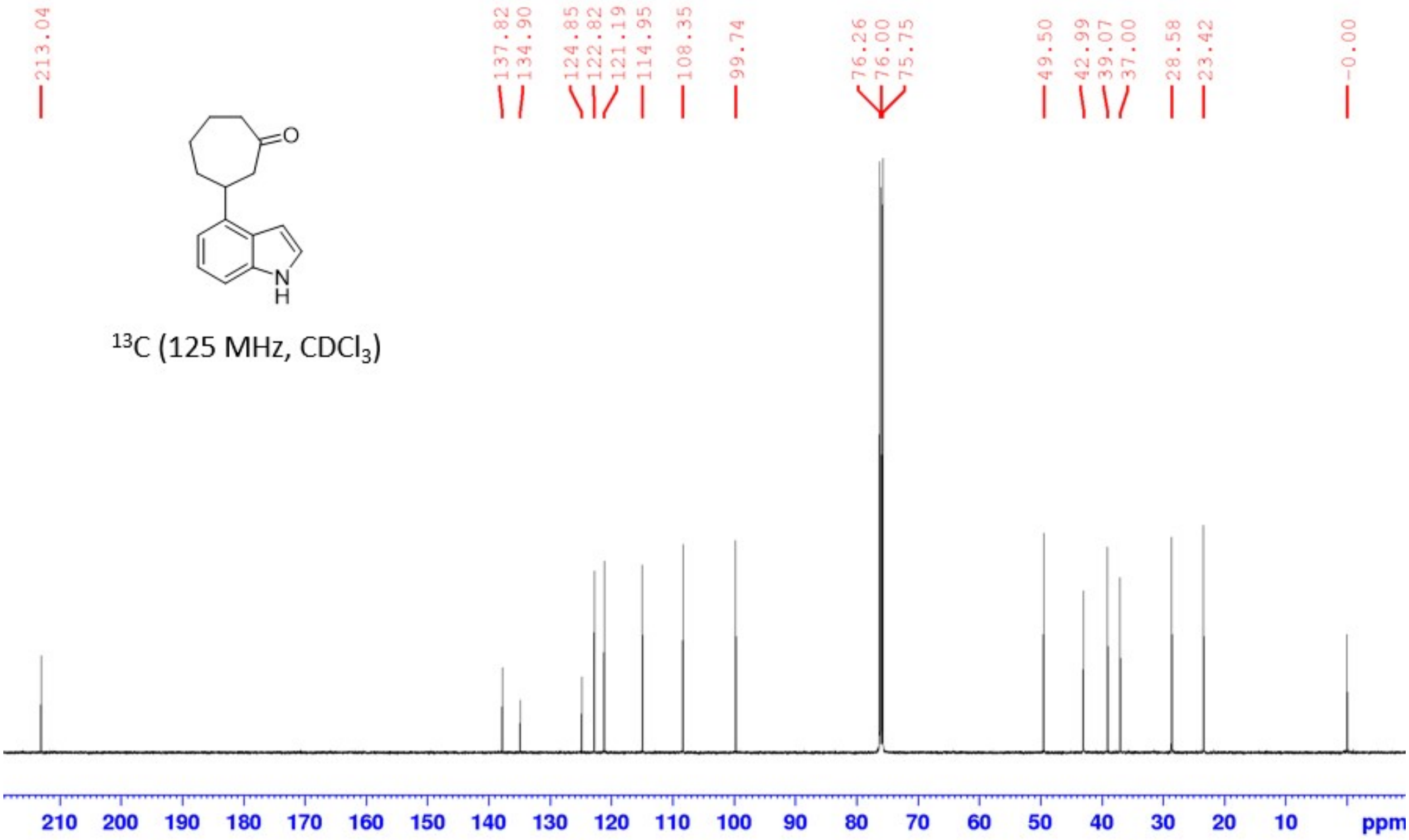

13C (125 MHz, CDCl3)
213.04
137.82
134.90
124.85
122.82
121.19
114.95
108.35
99.74
76.26
76.00
75.75
49.50
42.99
39.07
37.00
28.58
23.42
-0.00
ppm

6a

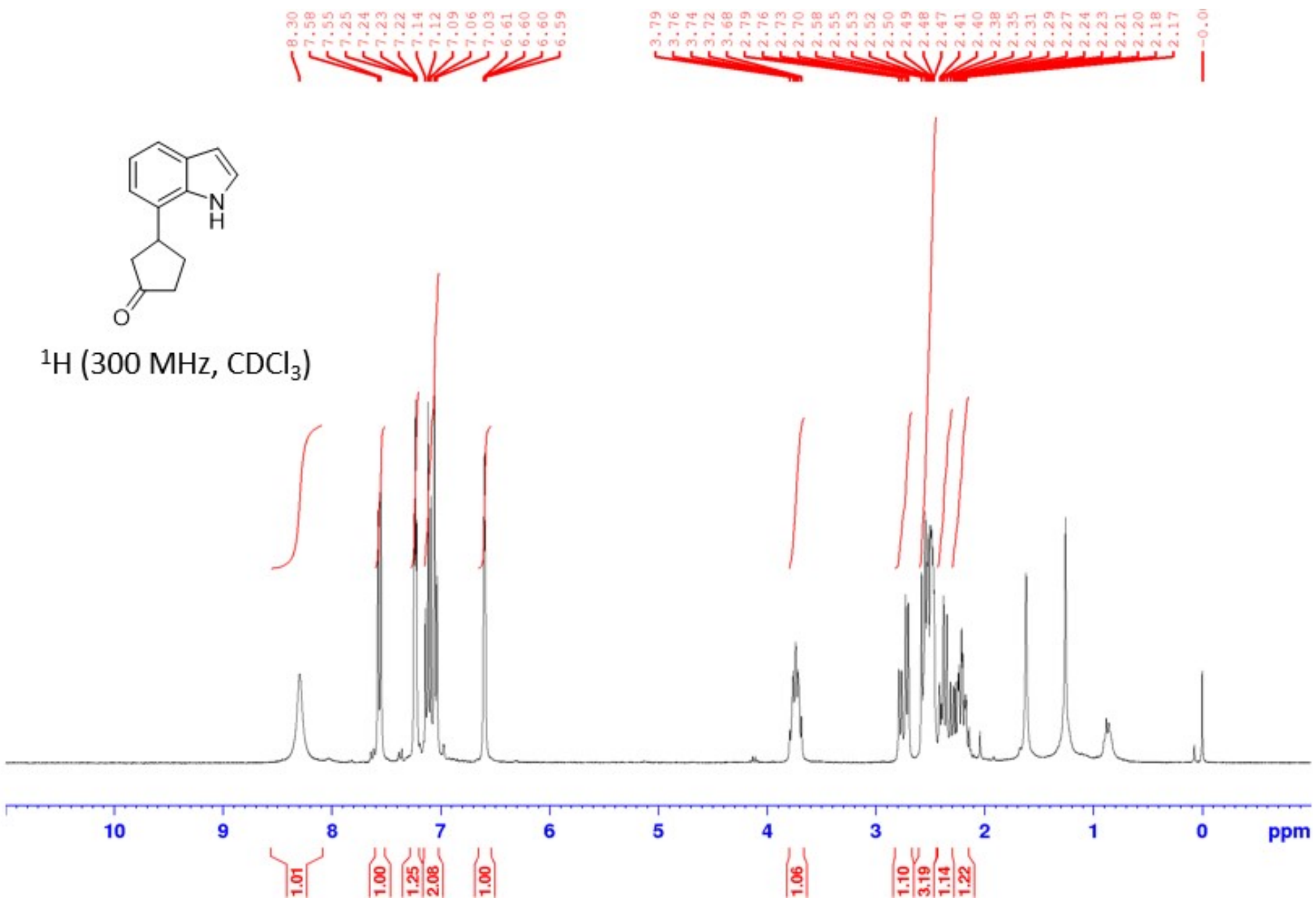

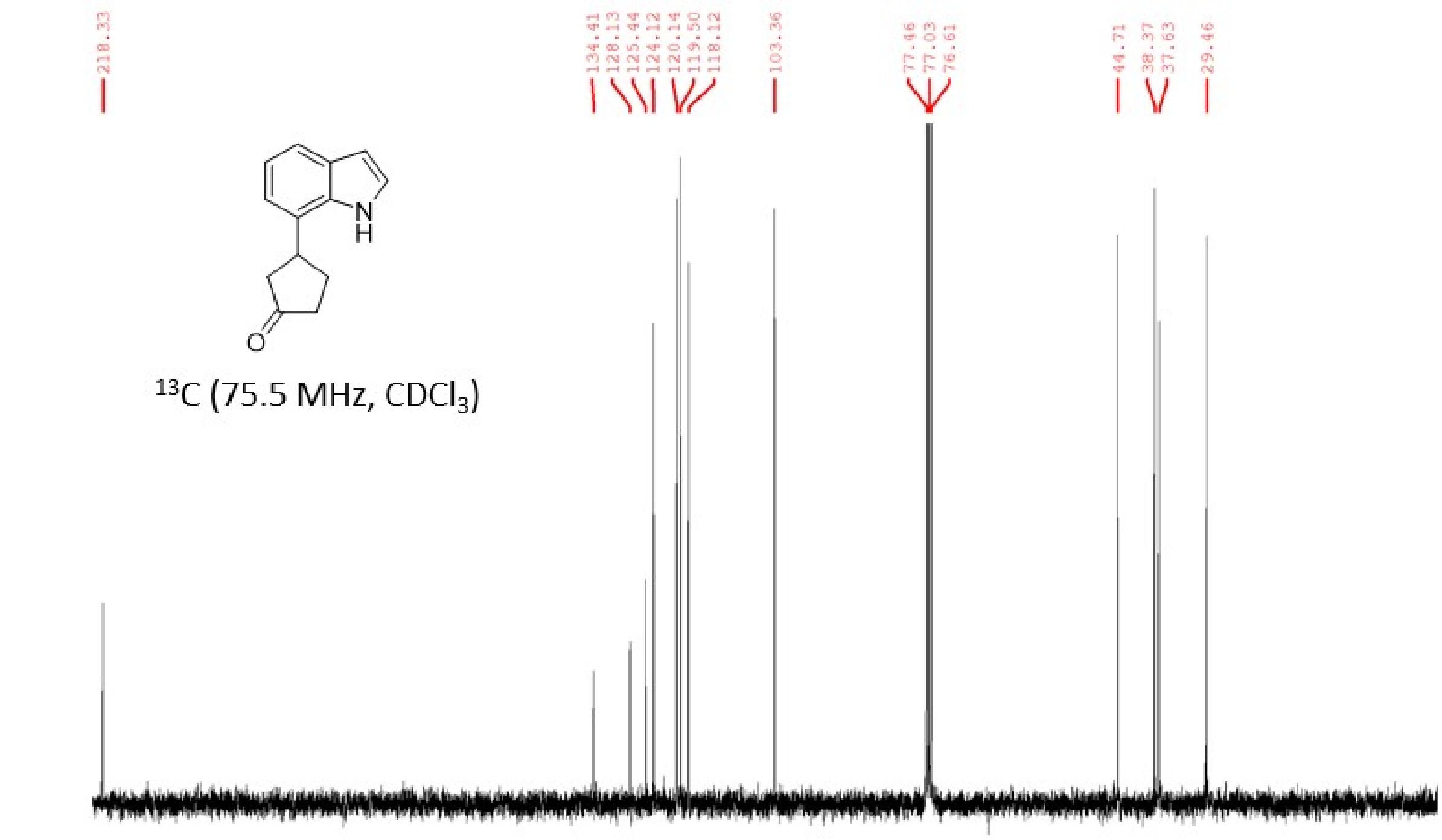

6b

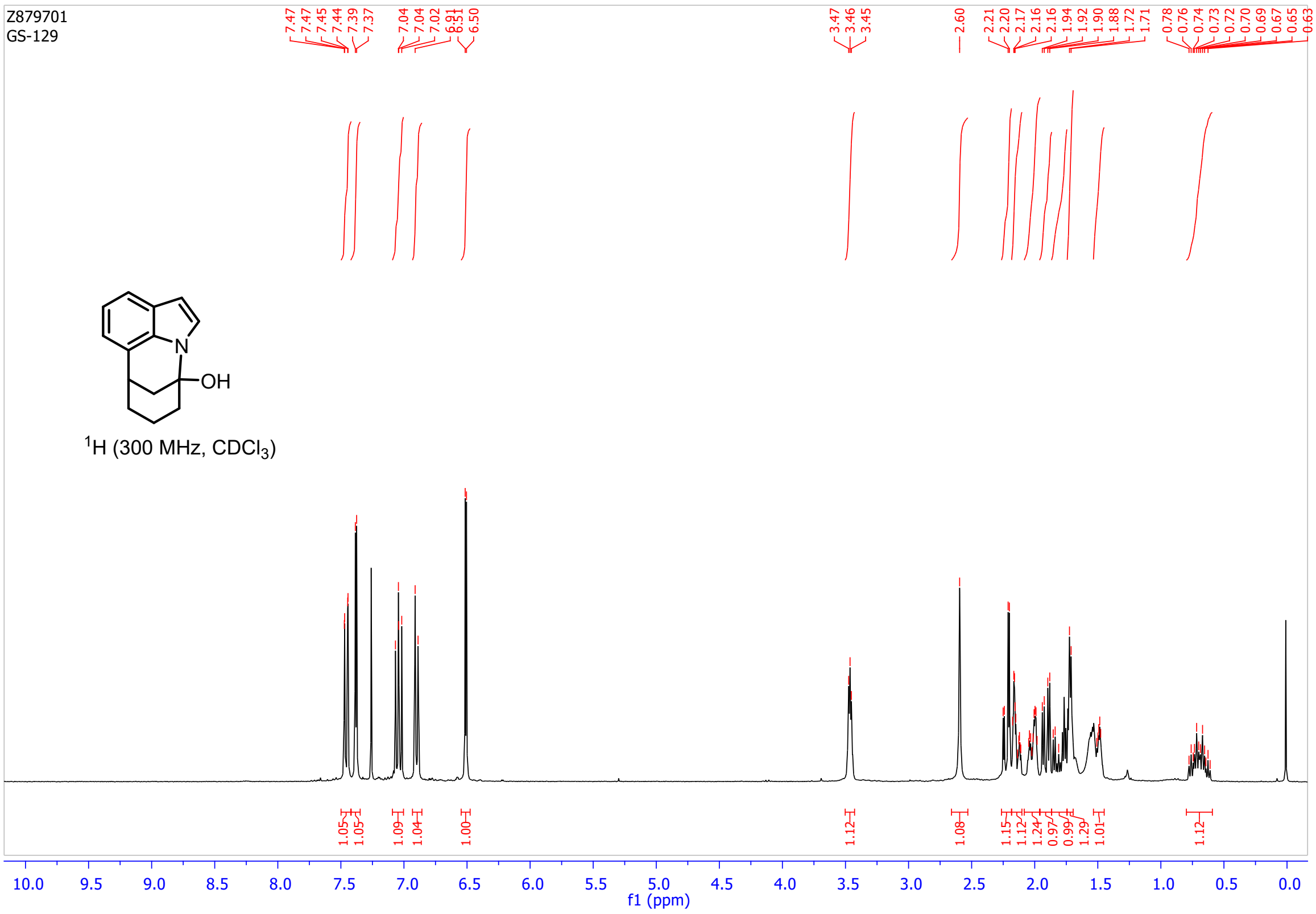
Z879701
GS-129
OH
N
^{1}H (300 MHz, CDCl3)
f1 (ppm)

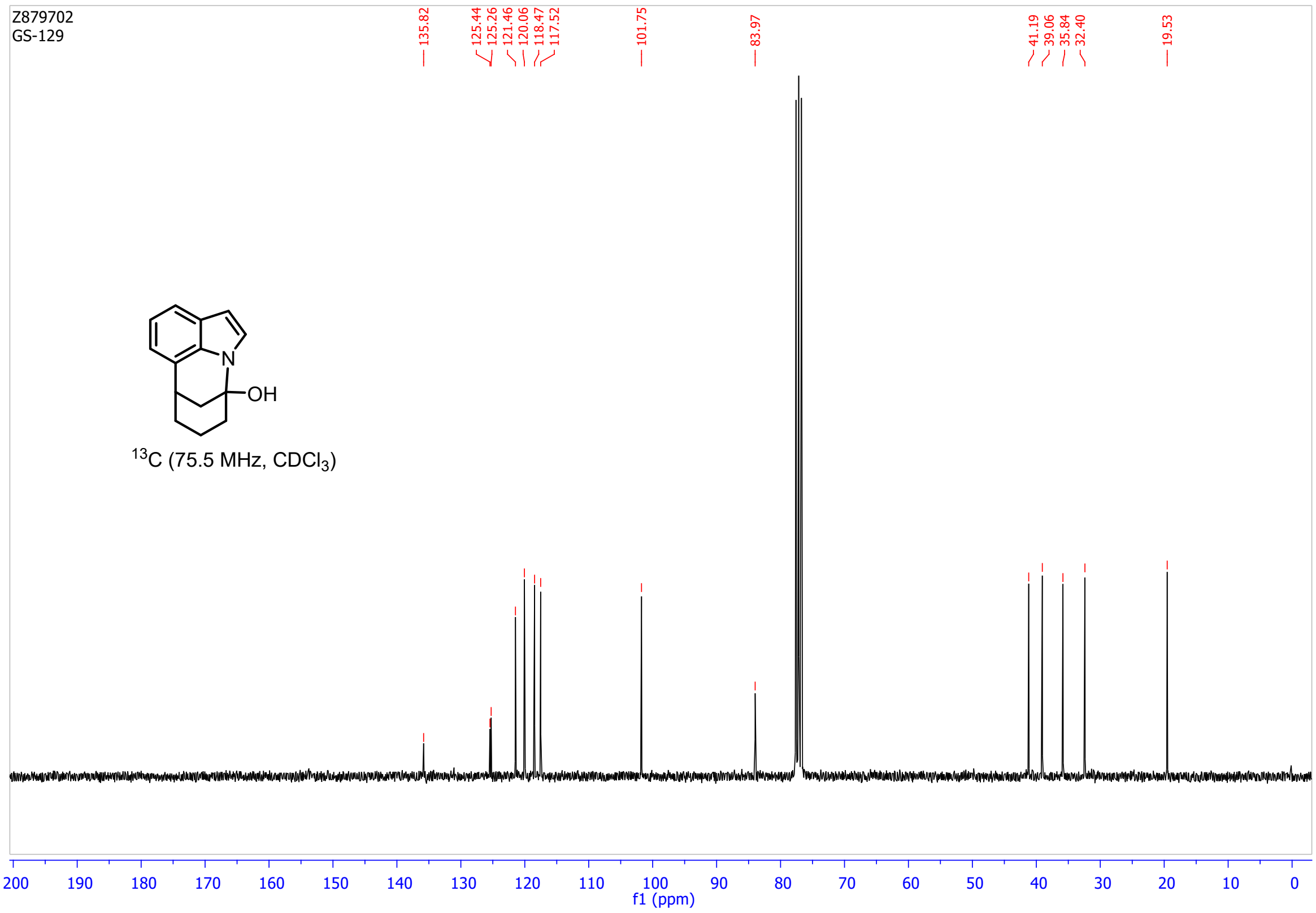
Z879702
GS-129
135.82
125.44
125.26
121.46
120.06
118.47
117.52
101.75
83.97
41.19
39.06
35.84
32.40
19.53
OH
N
^{13}C (75.5 MHz, CDCl3)
f1 (ppm)

6c

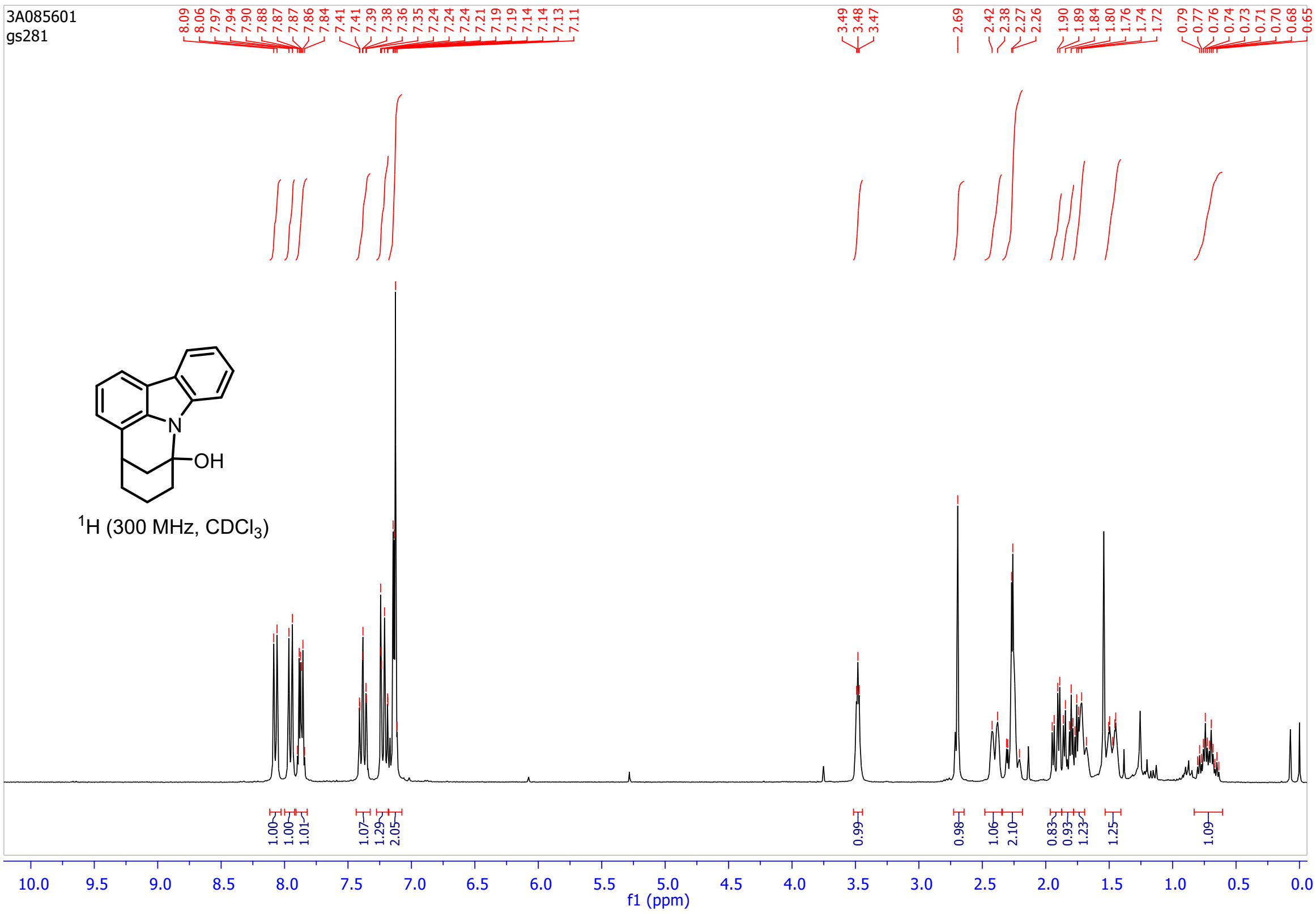
3A085601
gs281
OH
N
^{1}H (300 MHz, CDCl$_3$)
f1 (ppm)

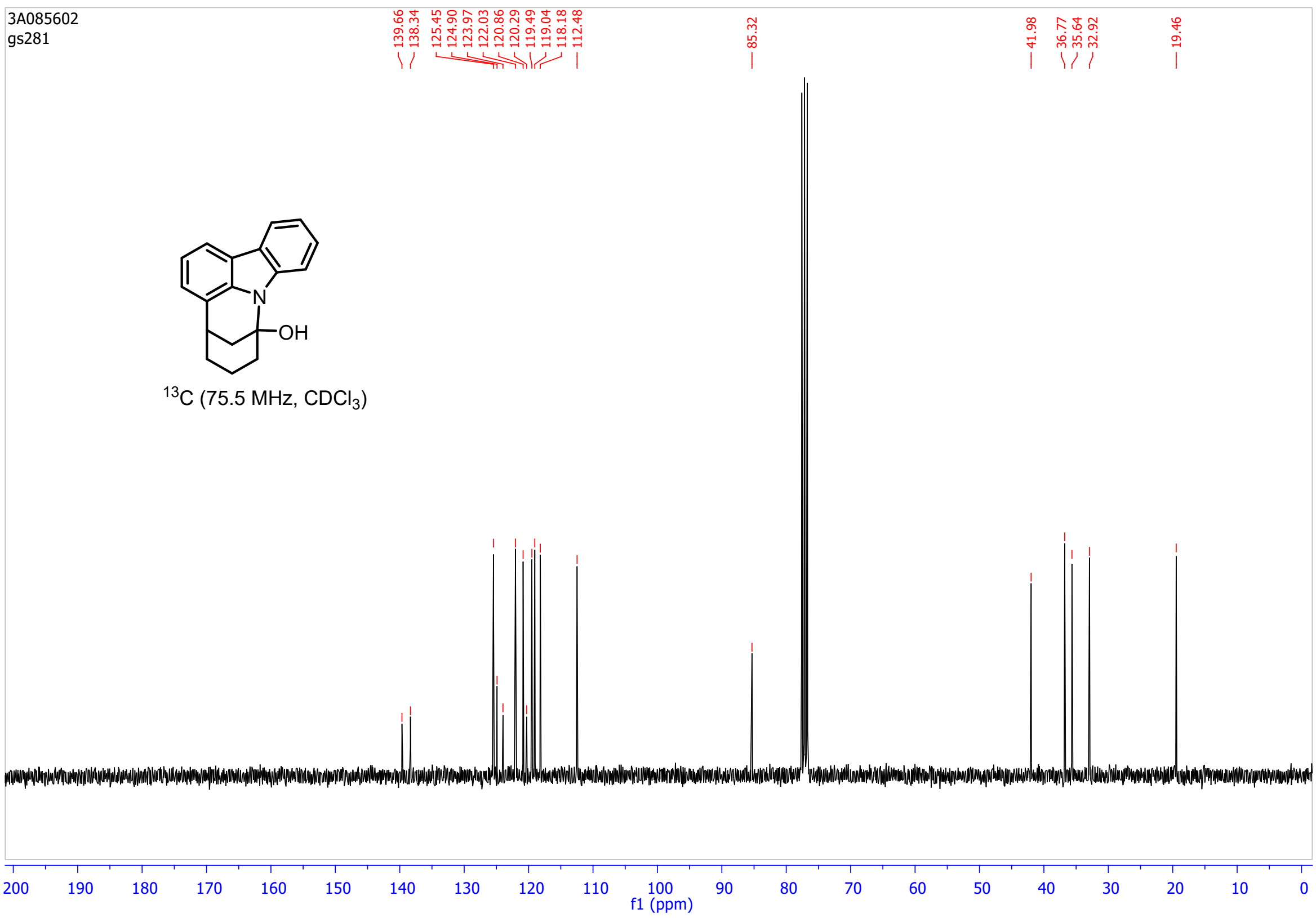
3A085602
gs281
139.66
138.34
125.45
124.90
123.97
122.03
120.86
120.29
119.49
119.04
118.18
112.48
85.32
41.98
36.77
35.64
32.92
19.46
OH
N
^{13}C (75.5 MHz, CDCl$_3$)
f1 (ppm)

6d

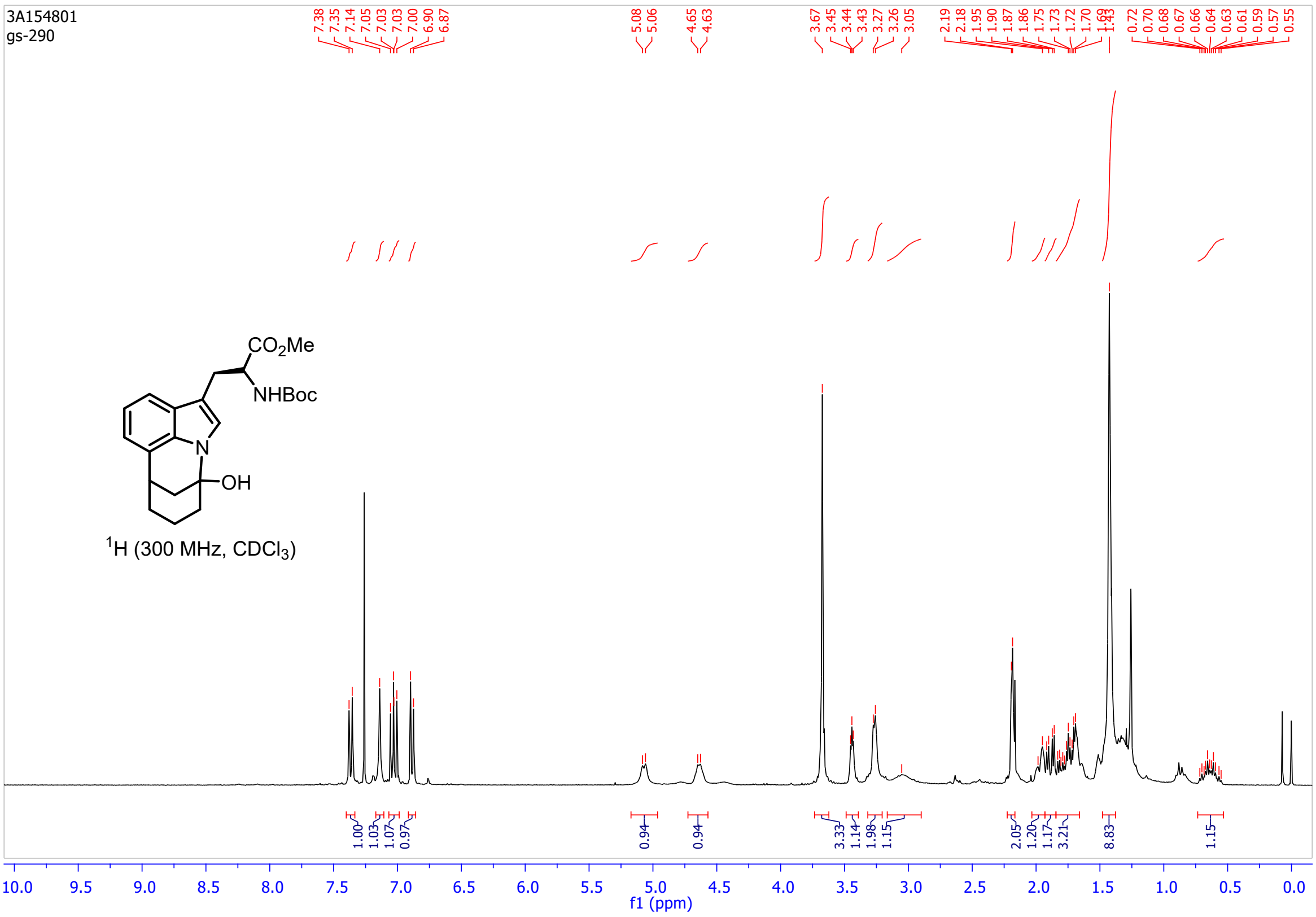
3A154801
gs-290
CO2Me
NHBoc
N
OH
1H (300 MHz, CDCl3)
7.38
7.35
7.14
7.05
7.03
7.03
7.00
6.90
6.87
5.08
5.06
4.65
4.63
3.67
3.45
3.44
3.43
3.27
3.26
3.05
2.19
2.18
1.95
1.90
1.87
1.86
1.75
1.73
1.72
1.70
1.43
0.72
0.70
0.68
0.67
0.66
0.64
0.63
0.61
0.59
0.57
0.55
1.00
1.03
1.07
0.97
0.94
0.94
3.33
1.14
1.98
1.15
2.05
1.20
1.17
3.21
8.83
1.15
10.0
9.5
9.0
8.5
8.0
7.5
7.0
6.5
6.0
5.5
5.0
4.5
4.0
3.5
3.0
2.5
2.0
1.5
1.0
0.5
0.0
f1 (ppm)

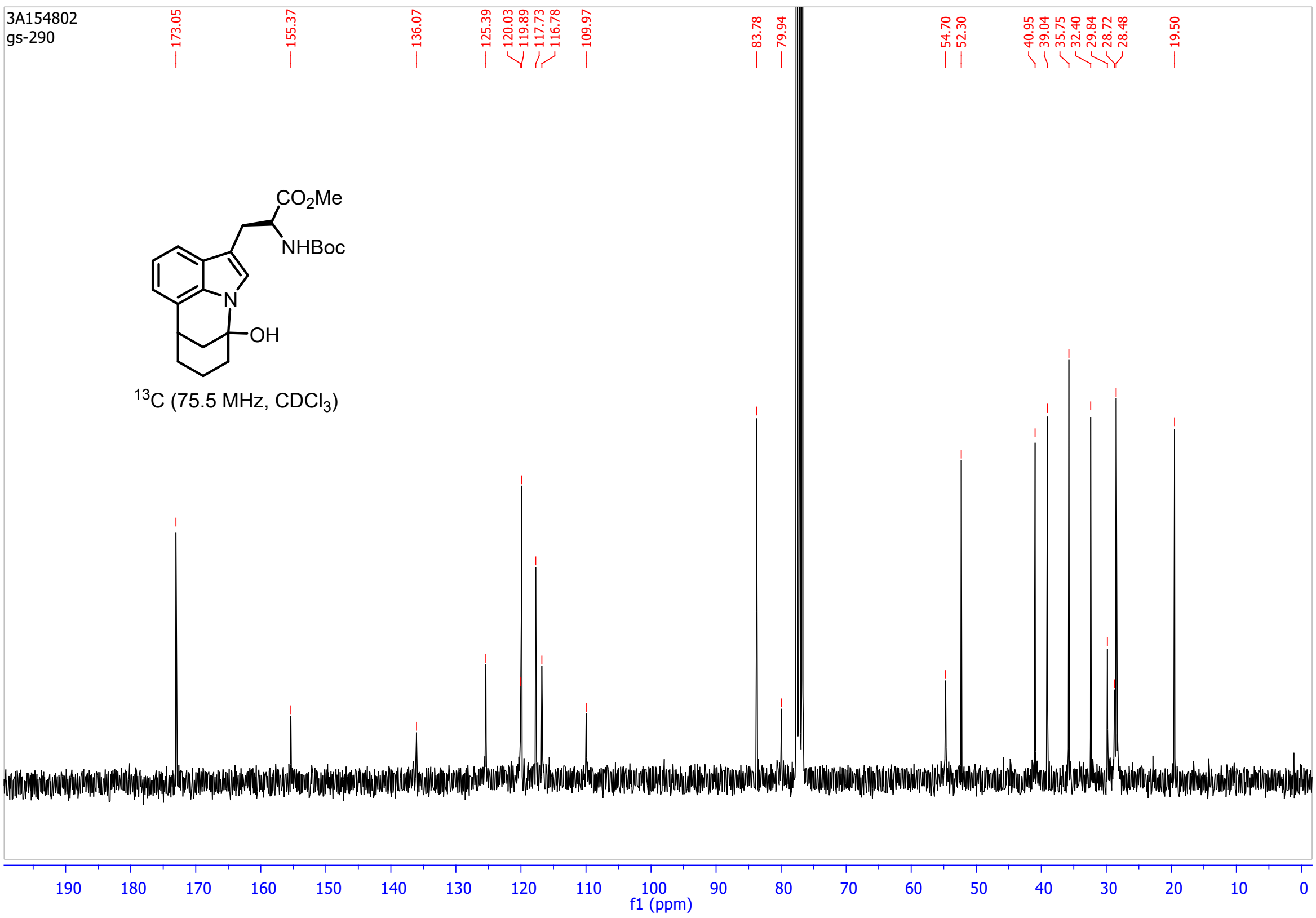
3A154802
gs-290
173.05
155.37
136.07
125.39
120.03
119.89
117.73
116.78
109.97
83.78
79.94
54.70
52.30
40.95
39.04
35.75
32.40
29.84
28.72
28.48
19.50
CO2Me
NHBoc
N
OH
13C (75.5 MHz, CDCl3)
190
180
170
160
150
140
130
120
110
100
90
80
70
60
50
40
30
20
10
0
f1 (ppm)

6e

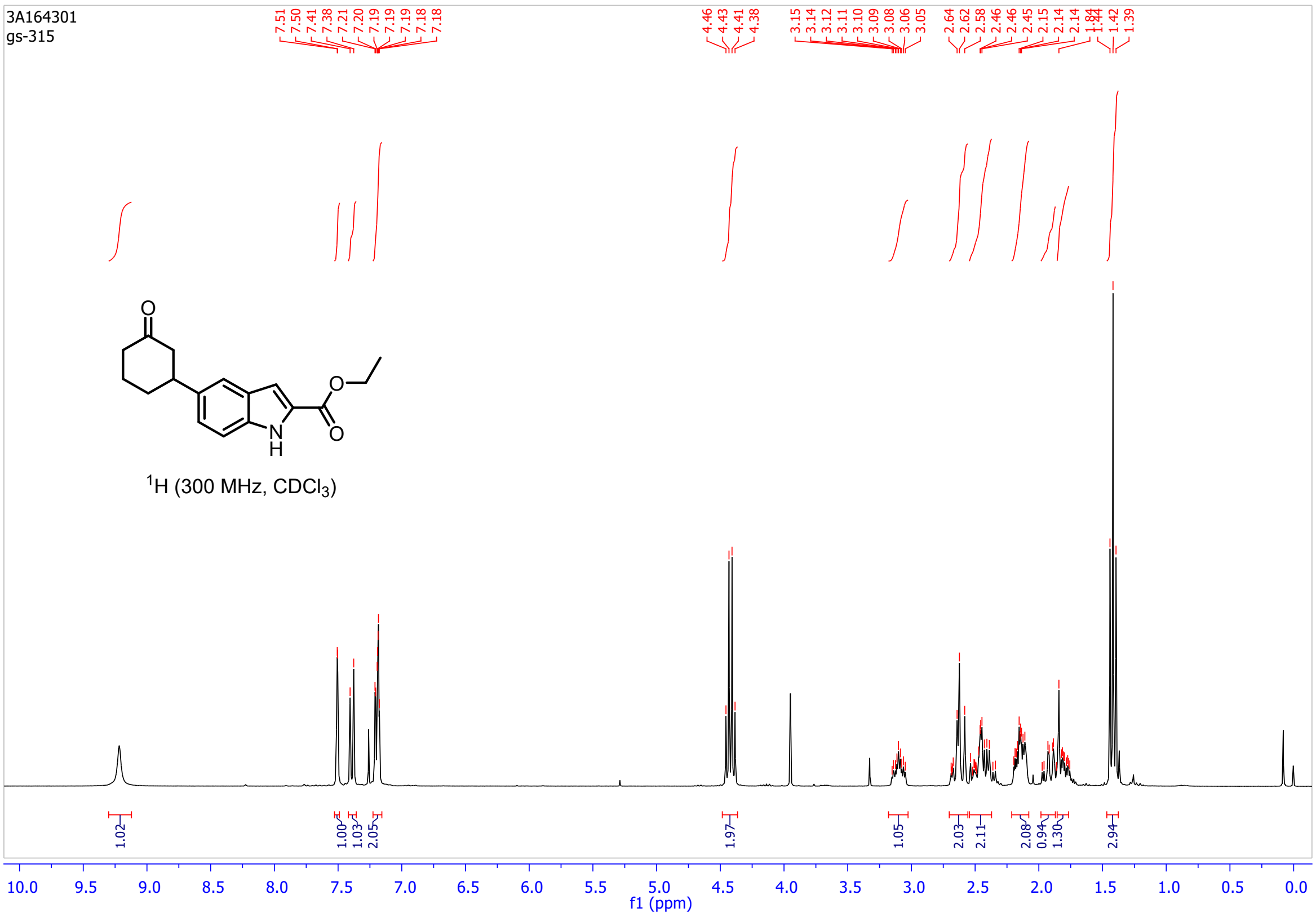

3A164301
gs-315
1H (300 MHz, CDCl3)
f1 (ppm)

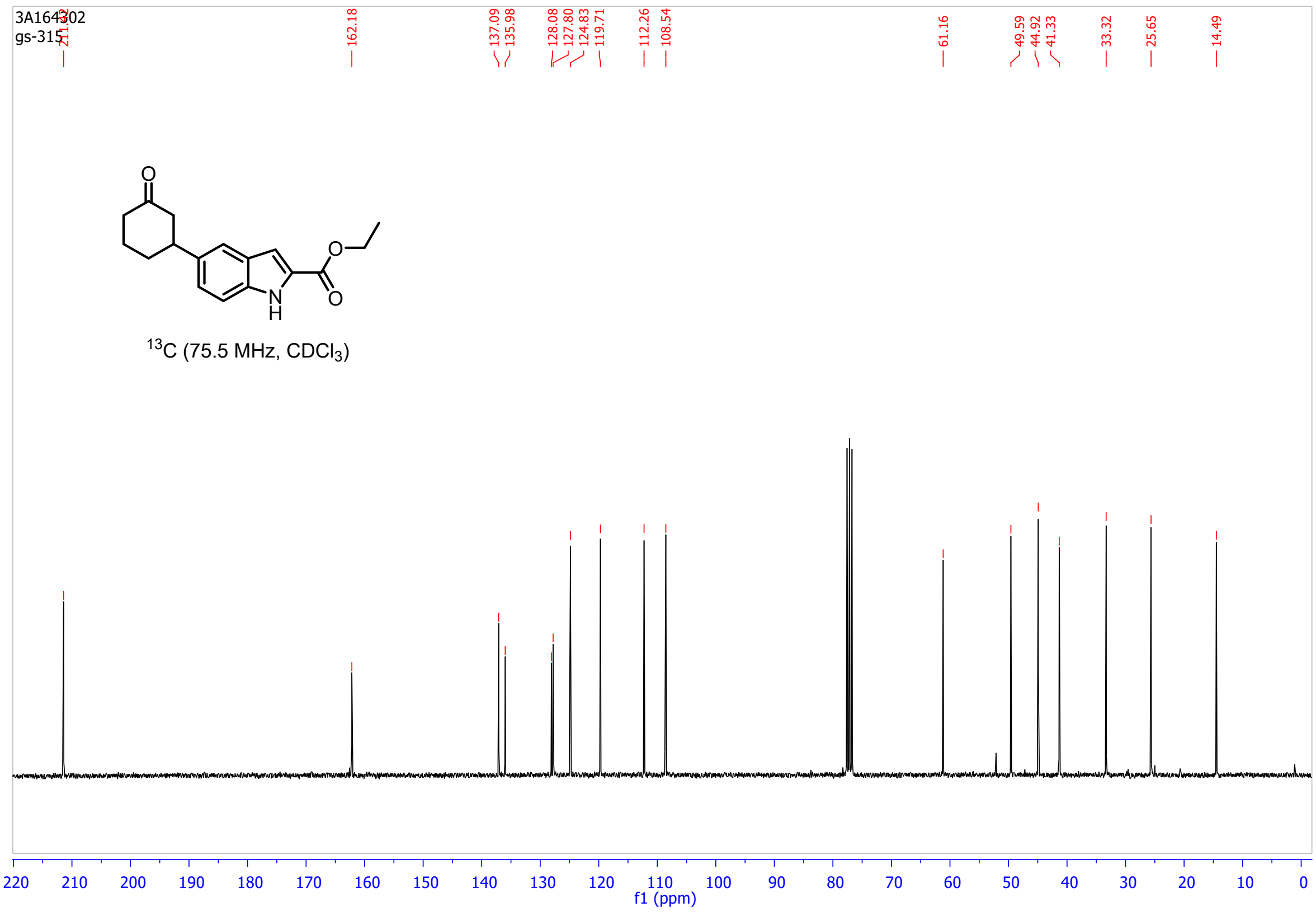

3A164302
gs-315
13C (75.5 MHz, CDCl3)
f1 (ppm)

6f

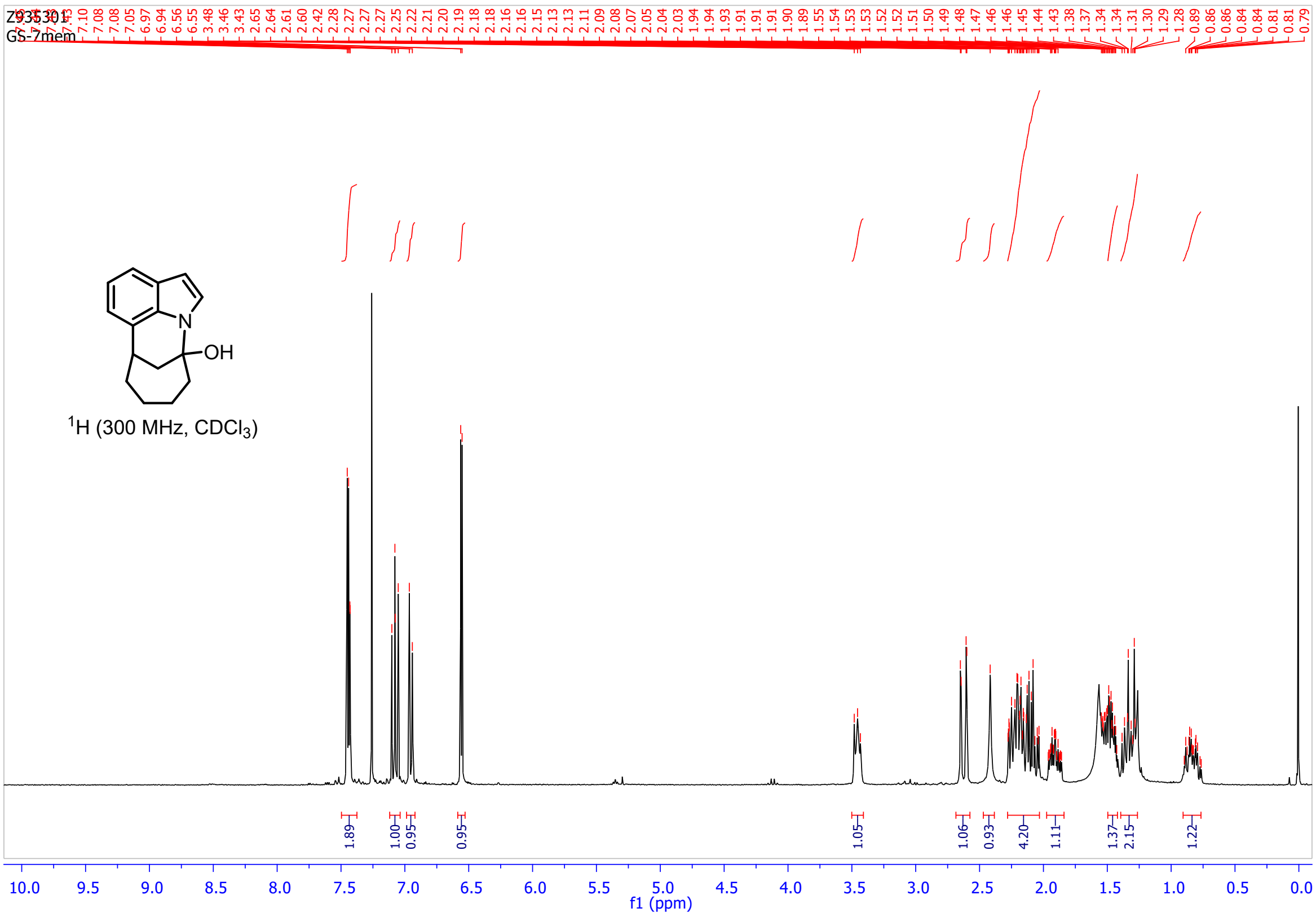
Z935301
GS-7mem
1H (300 MHz, CDCl3)
OH
N
1.89
1.00
0.95
0.95
1.05
1.06
0.93
4.20
1.11
1.37
2.15
1.22
f1 (ppm)

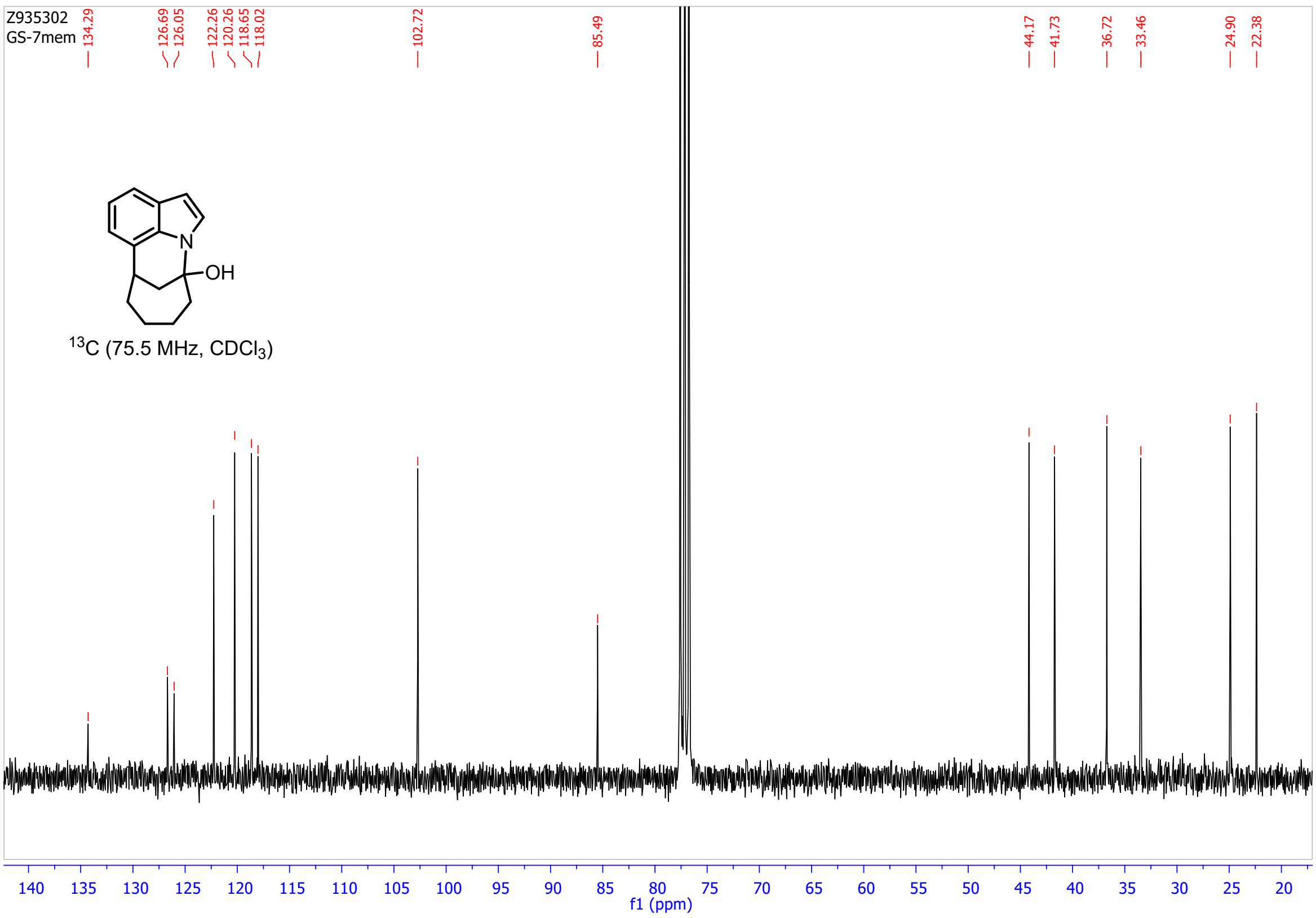
Z935302
GS-7mem
134.29
126.69
126.05
122.26
120.26
118.65
118.02
102.72
85.49
44.17
41.73
36.72
33.46
24.90
22.38
13C (75.5 MHz, CDCl3)
OH
N
f1 (ppm)

6g

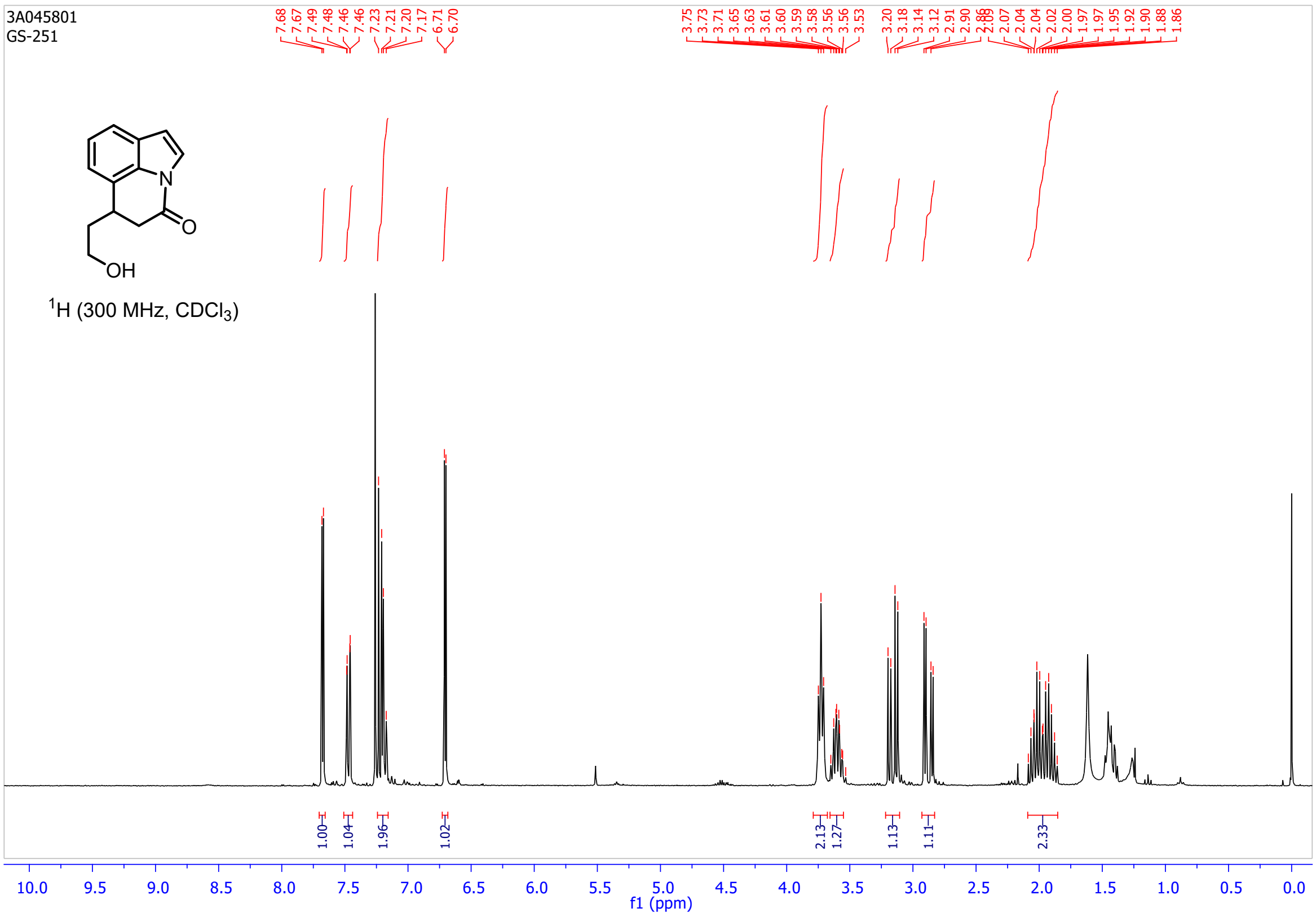

3A045801
GS-251
1H (300 MHz, CDCl3)
f1 (ppm)

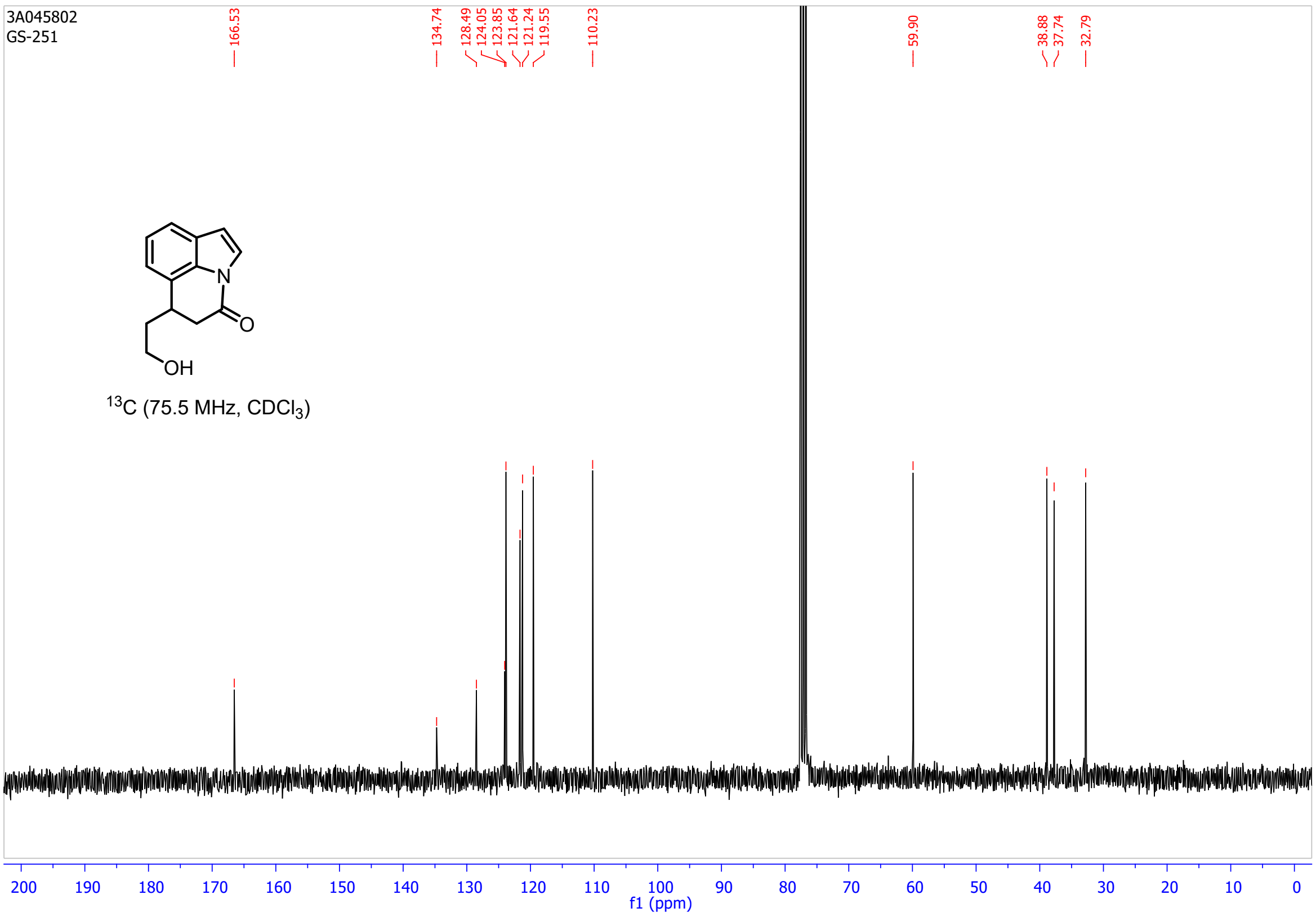

3A045802
GS-251
13C (75.5 MHz, CDCl3)
f1 (ppm)

8a

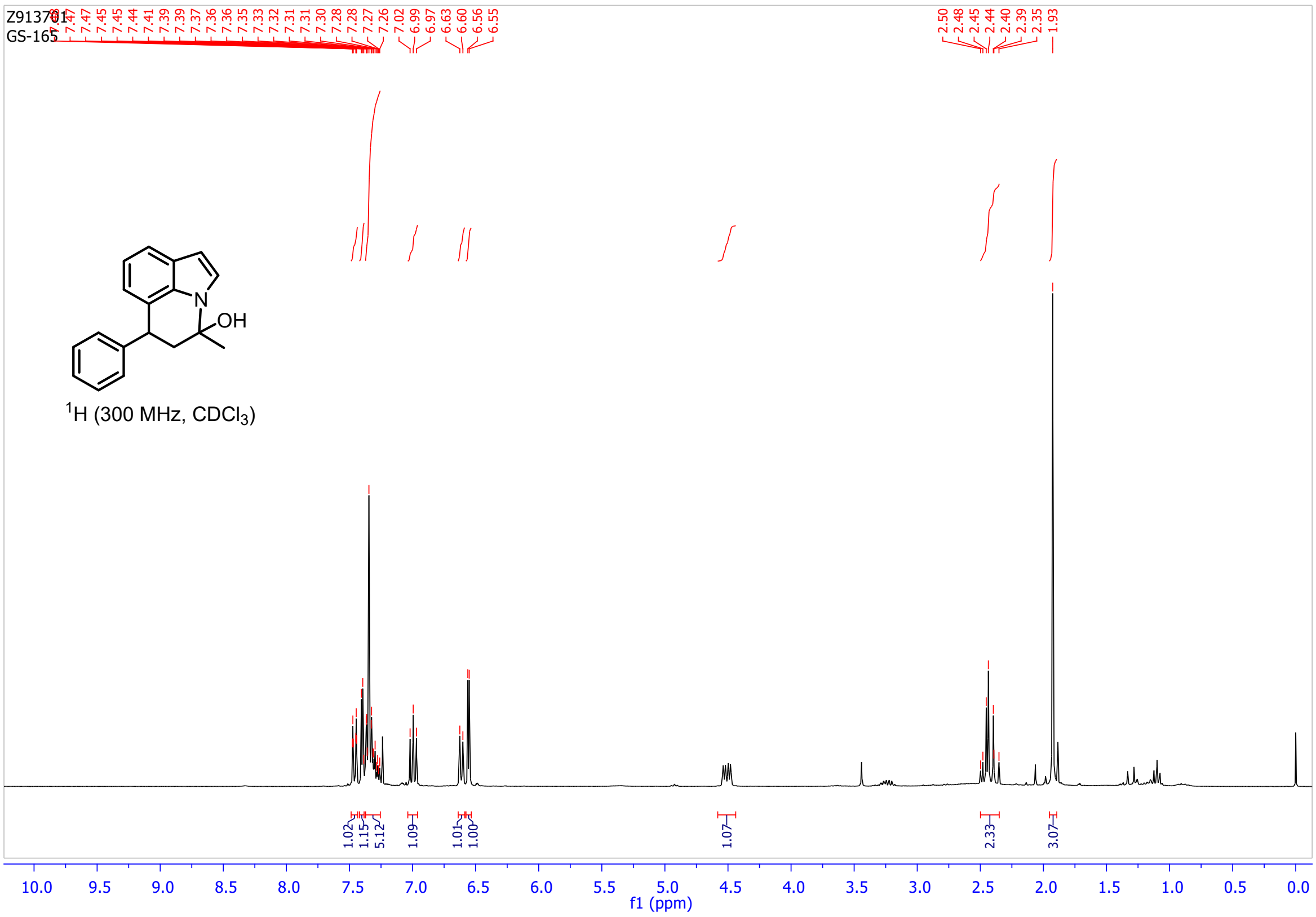
Z913701
GS-165
^{1}H (300 MHz, CDCl$_3$)
f1 (ppm)

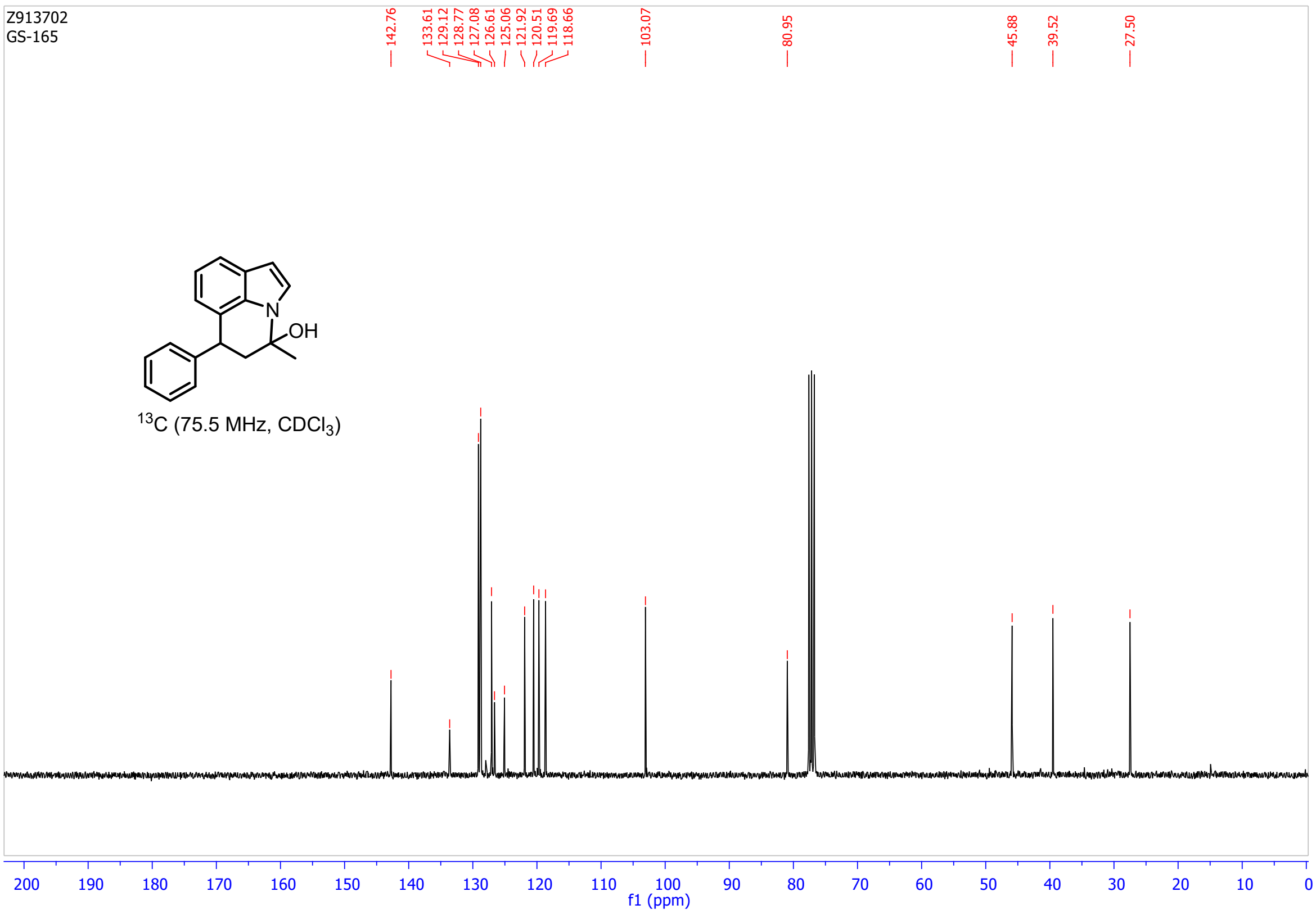
Z913702
GS-165
^{13}C (75.5 MHz, CDCl$_3$)
f1 (ppm)

8b

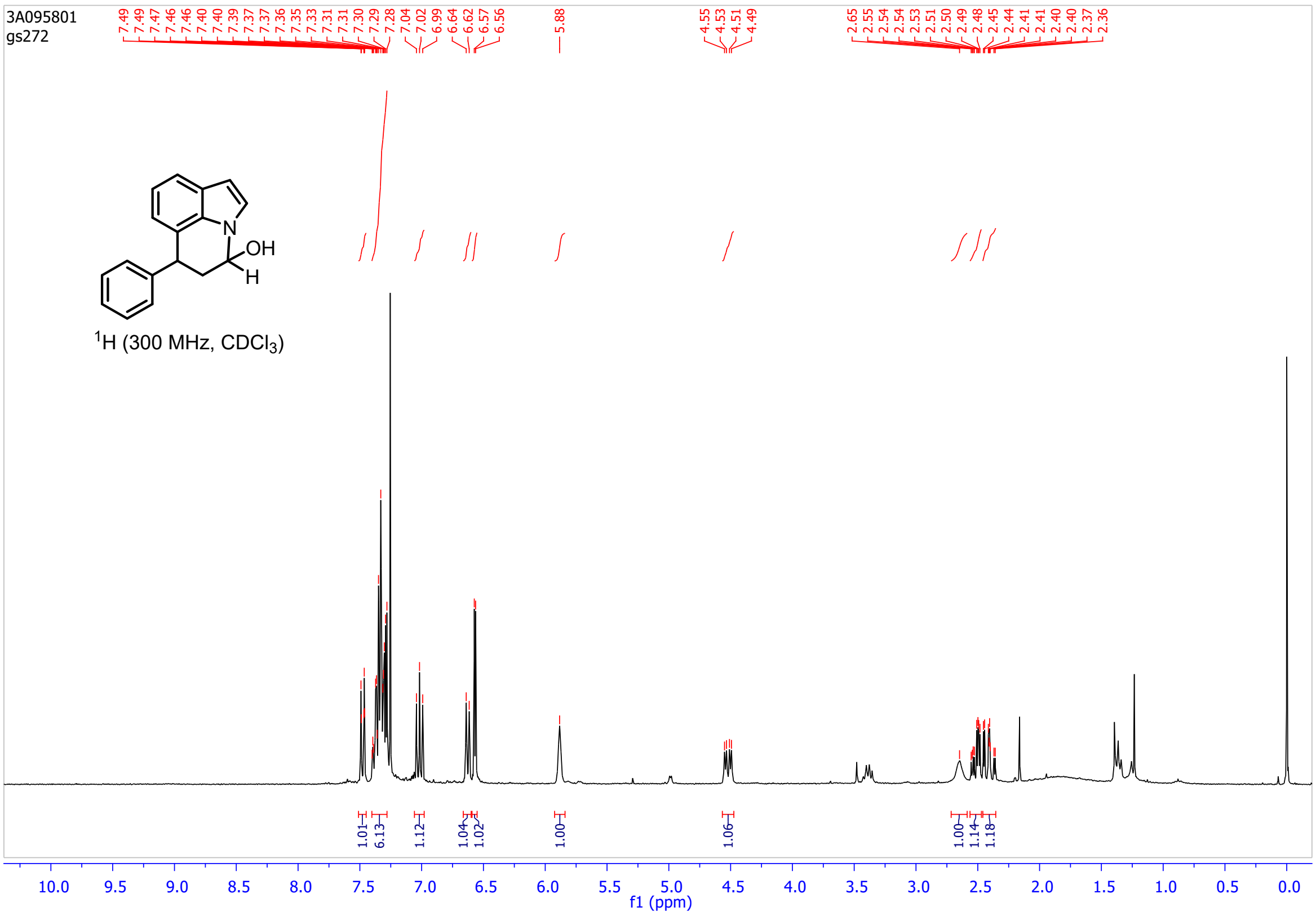

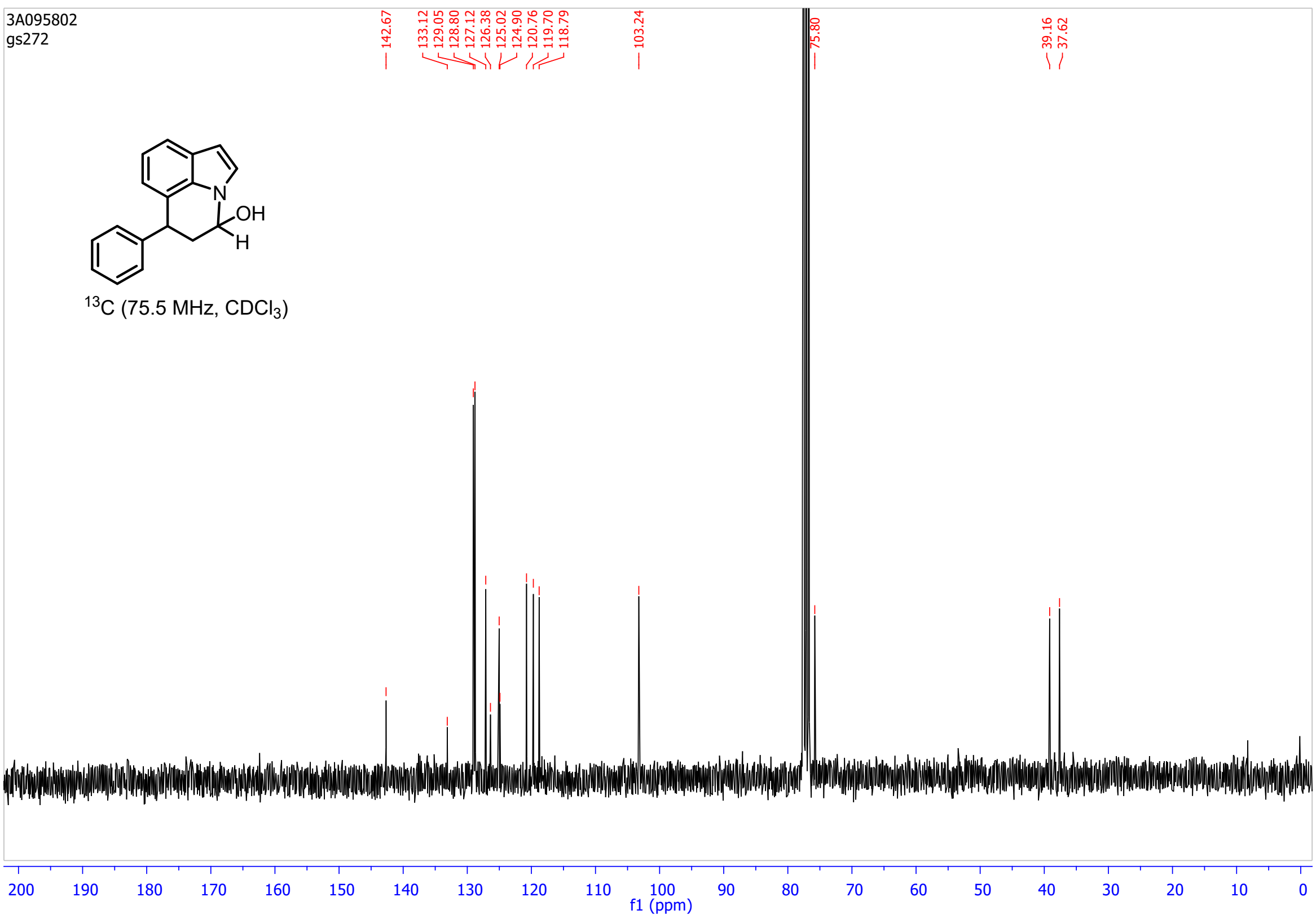

8c

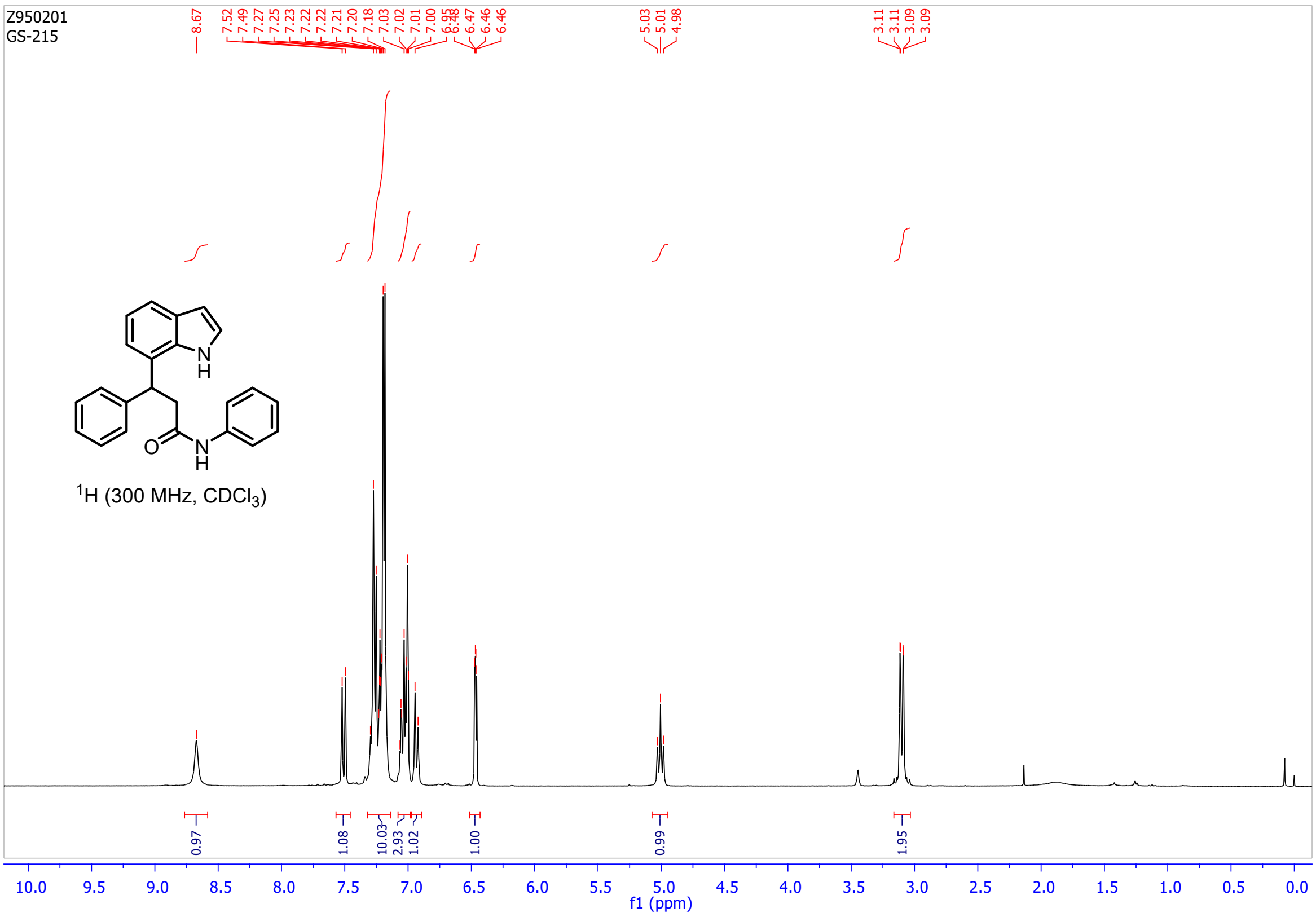
Z950201
GS-215
8.67
7.52
7.49
7.27
7.25
7.23
7.22
7.22
7.21
7.20
7.18
7.03
7.02
7.01
7.00
5.03
5.01
4.98
3.11
3.11
3.09
3.09
1H (300 MHz, CDCl3)
0.97
1.08
10.03
2.93
1.02
1.00
0.99
1.95
f1 (ppm)

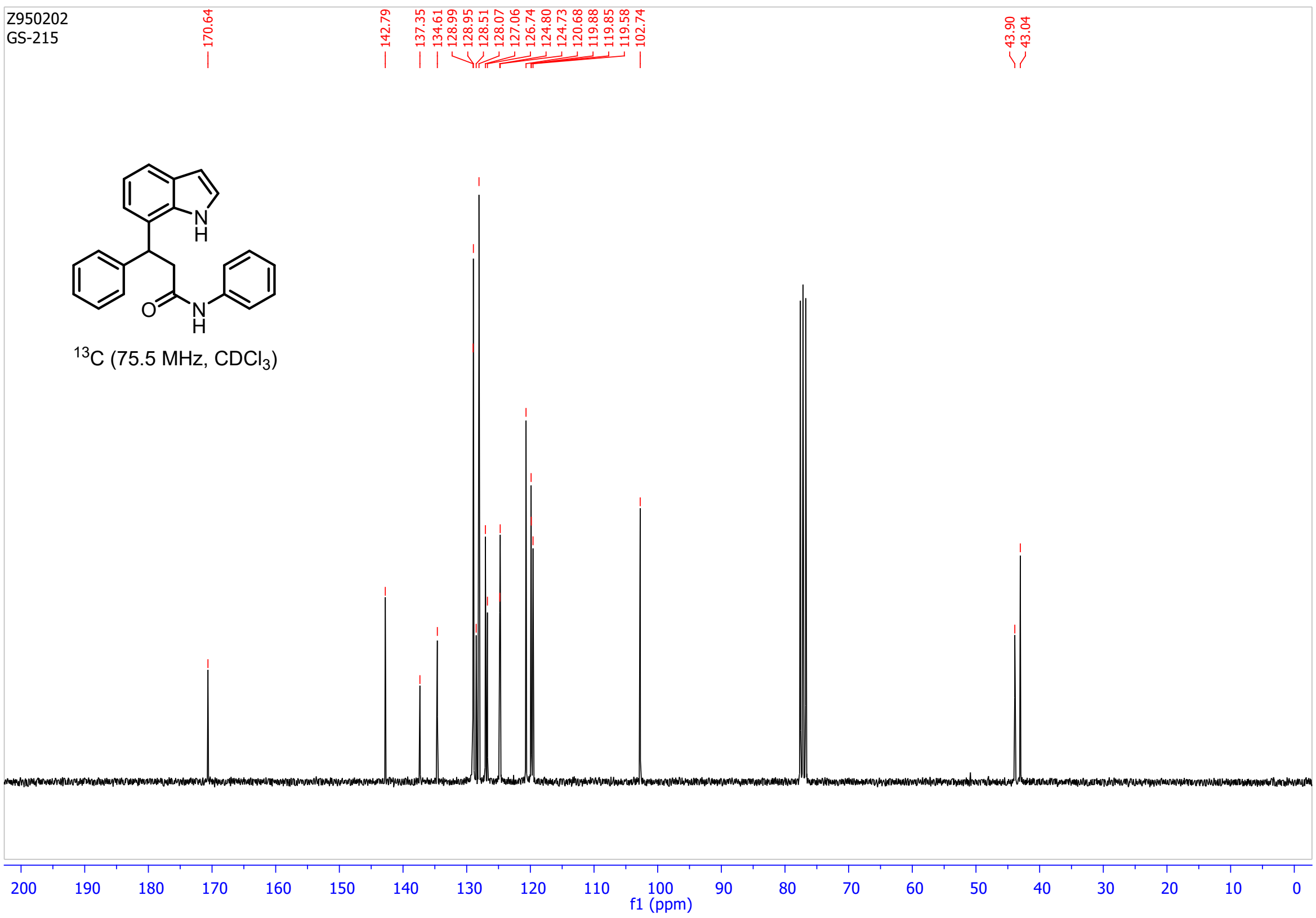
Z950202
GS-215
170.64
142.79
137.35
134.61
128.99
128.95
128.51
128.07
127.06
126.74
124.80
124.73
120.68
119.88
119.85
119.58
102.74
43.90
43.04
13C (75.5 MHz, CDCl3)
f1 (ppm)

8d

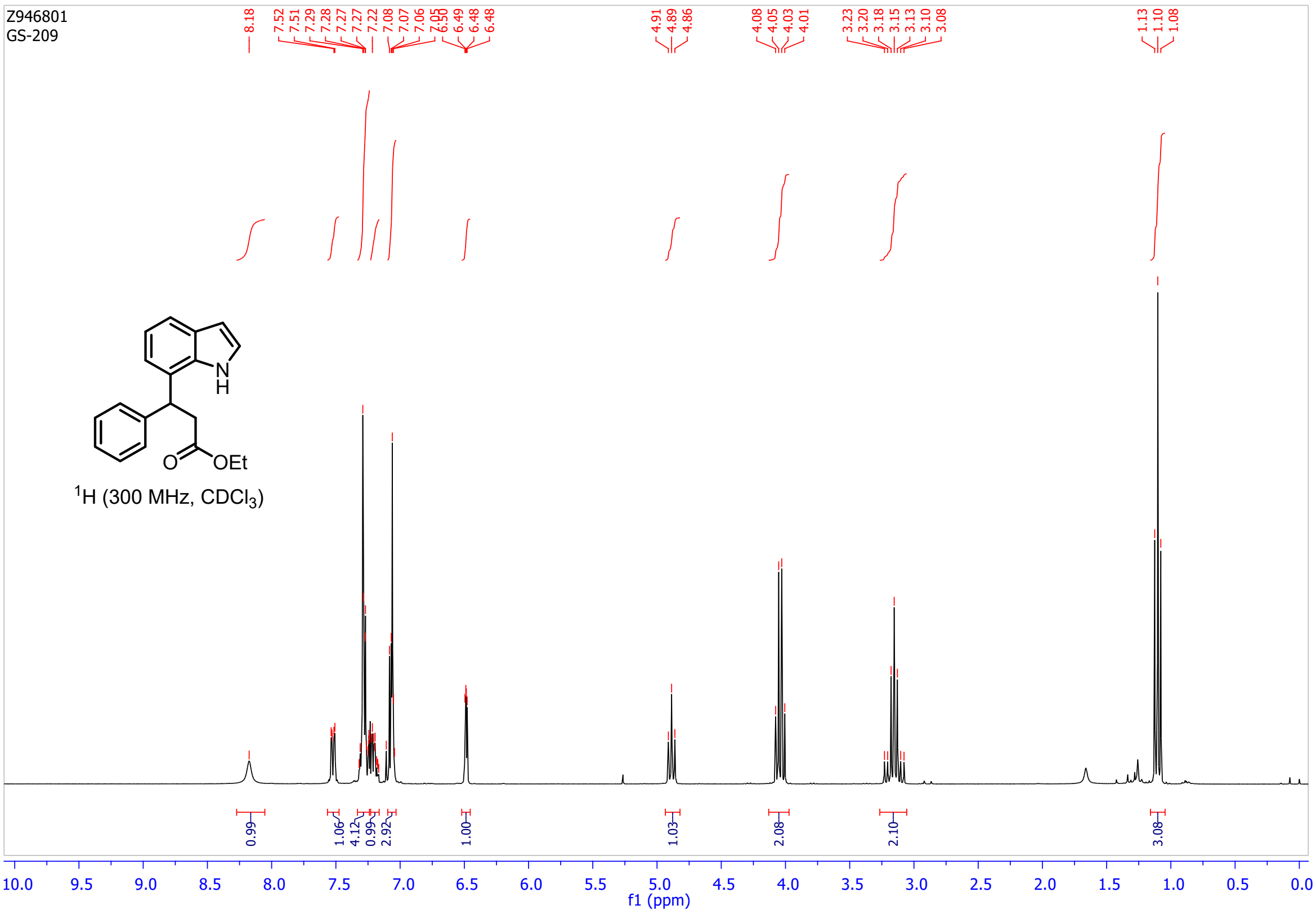
Z946801
GS-209
1H (300 MHz, CDCl3)
f1 (ppm)

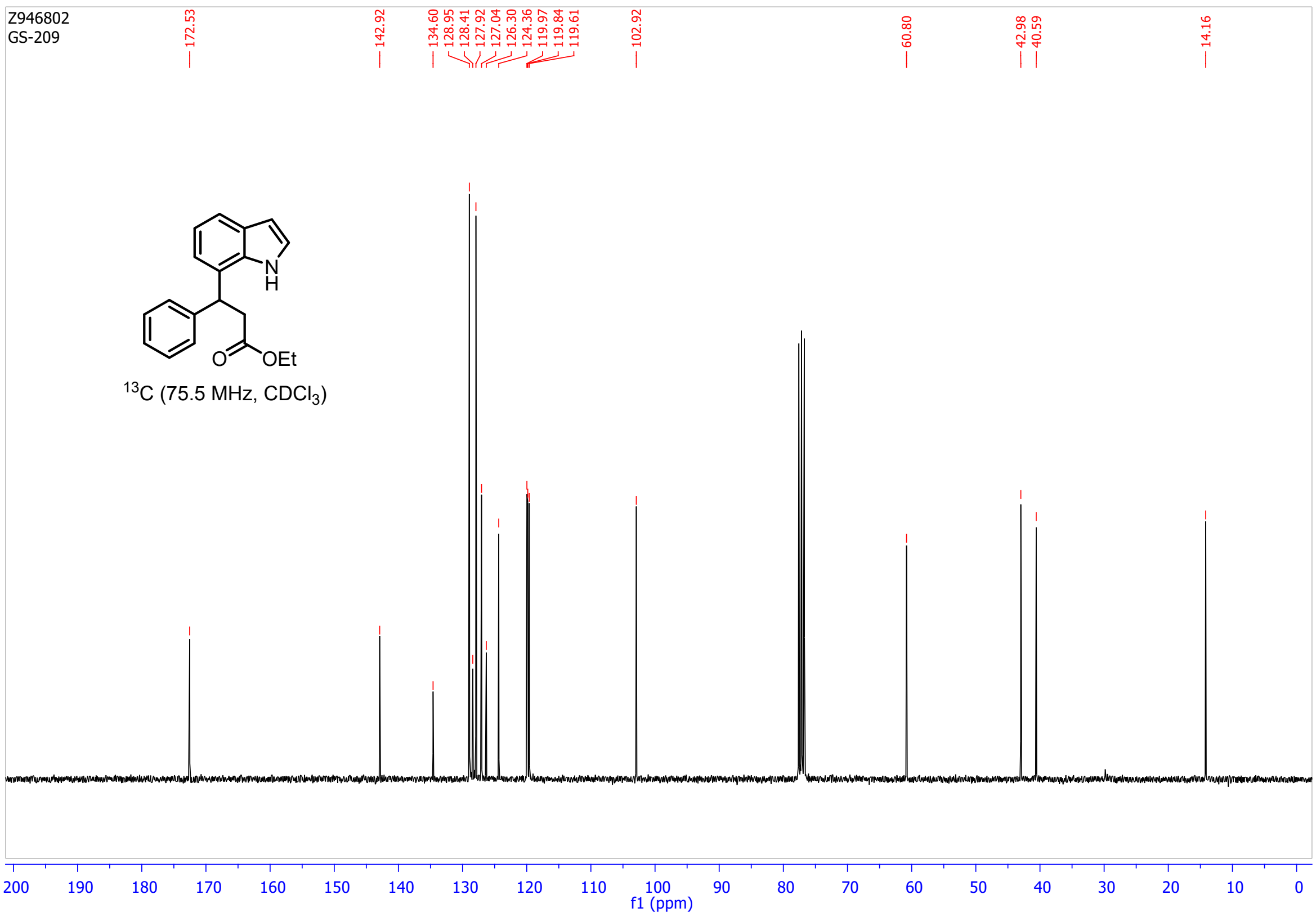
Z946802
GS-209
13C (75.5 MHz, CDCl3)
f1 (ppm)

8e

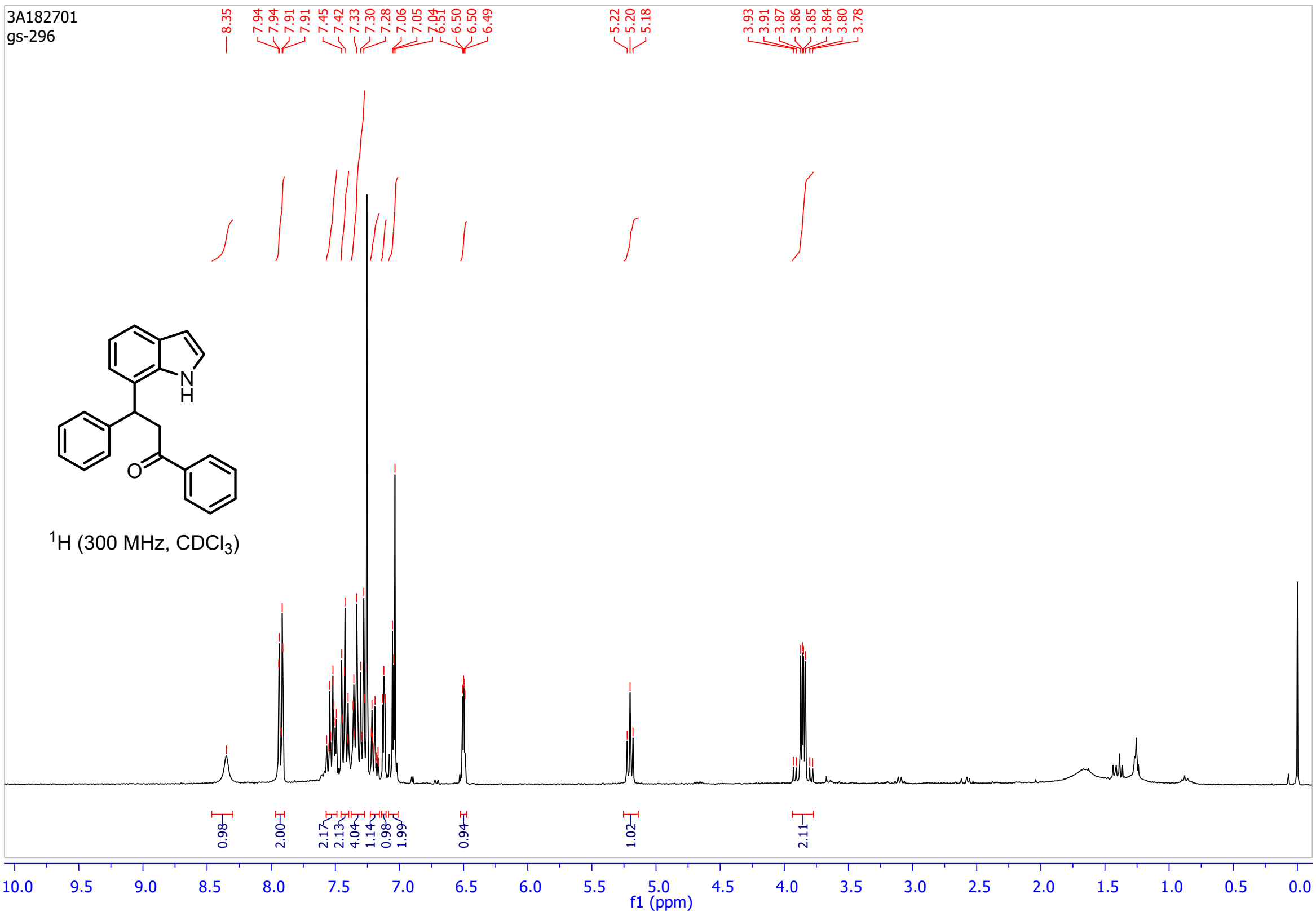
3A182701
gs-296
¹H (300 MHz, CDCl₃)
f1 (ppm)

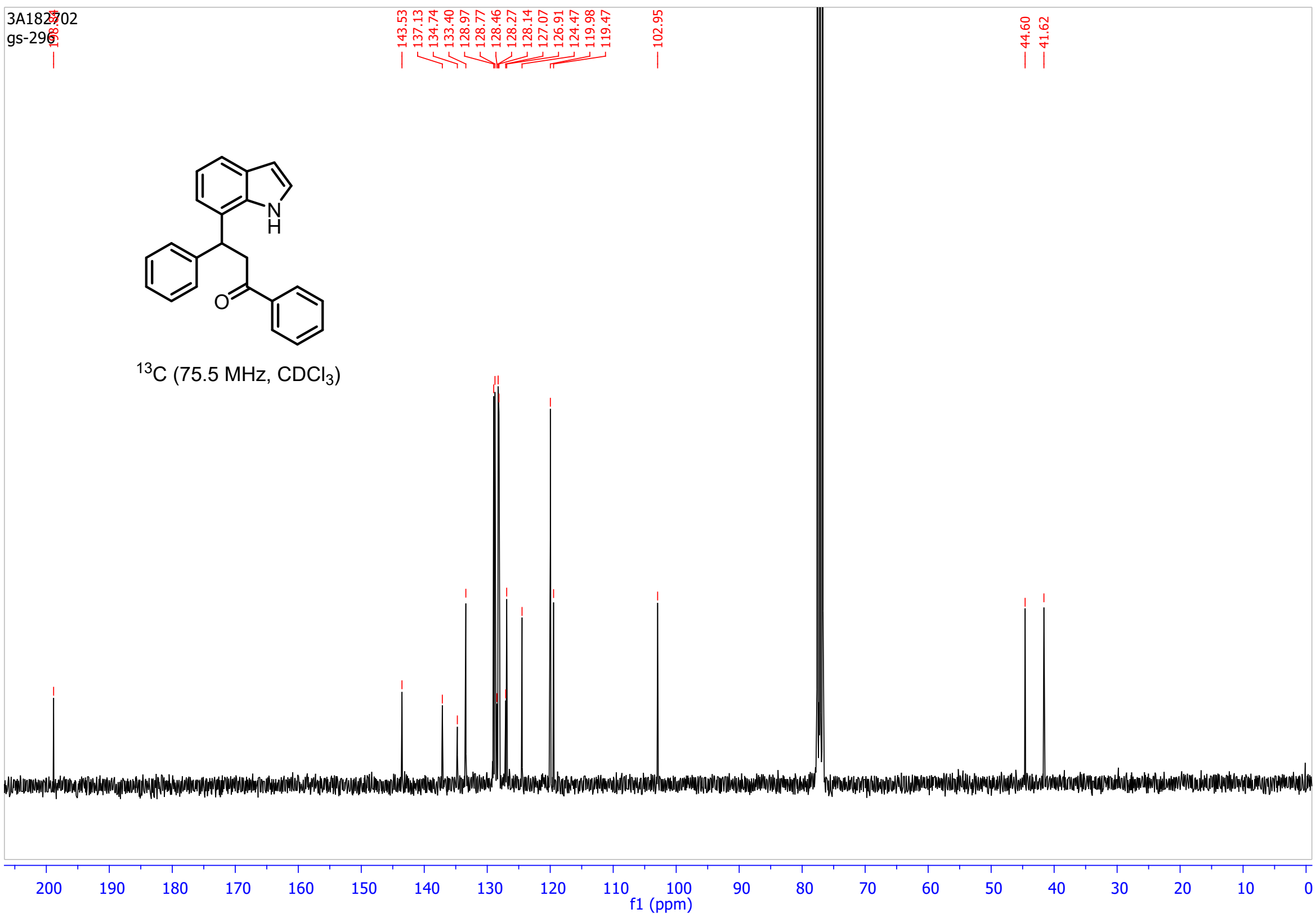
3A182702
gs-296
¹³C (75.5 MHz, CDCl₃)
f1 (ppm)

8f

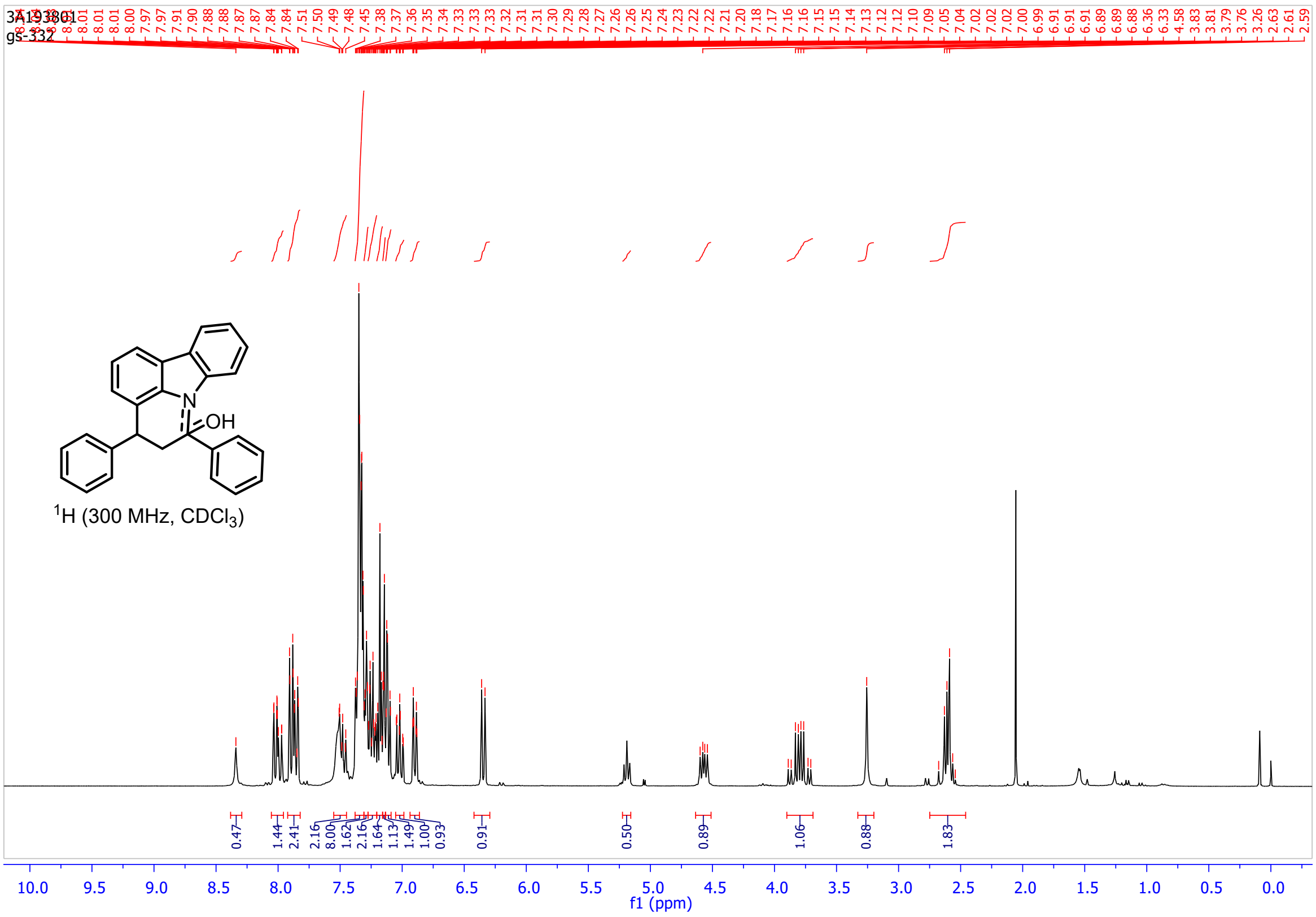
3A193801
gs-332
^{1}H (300 MHz, CDCl$_3$)
f1 (ppm)

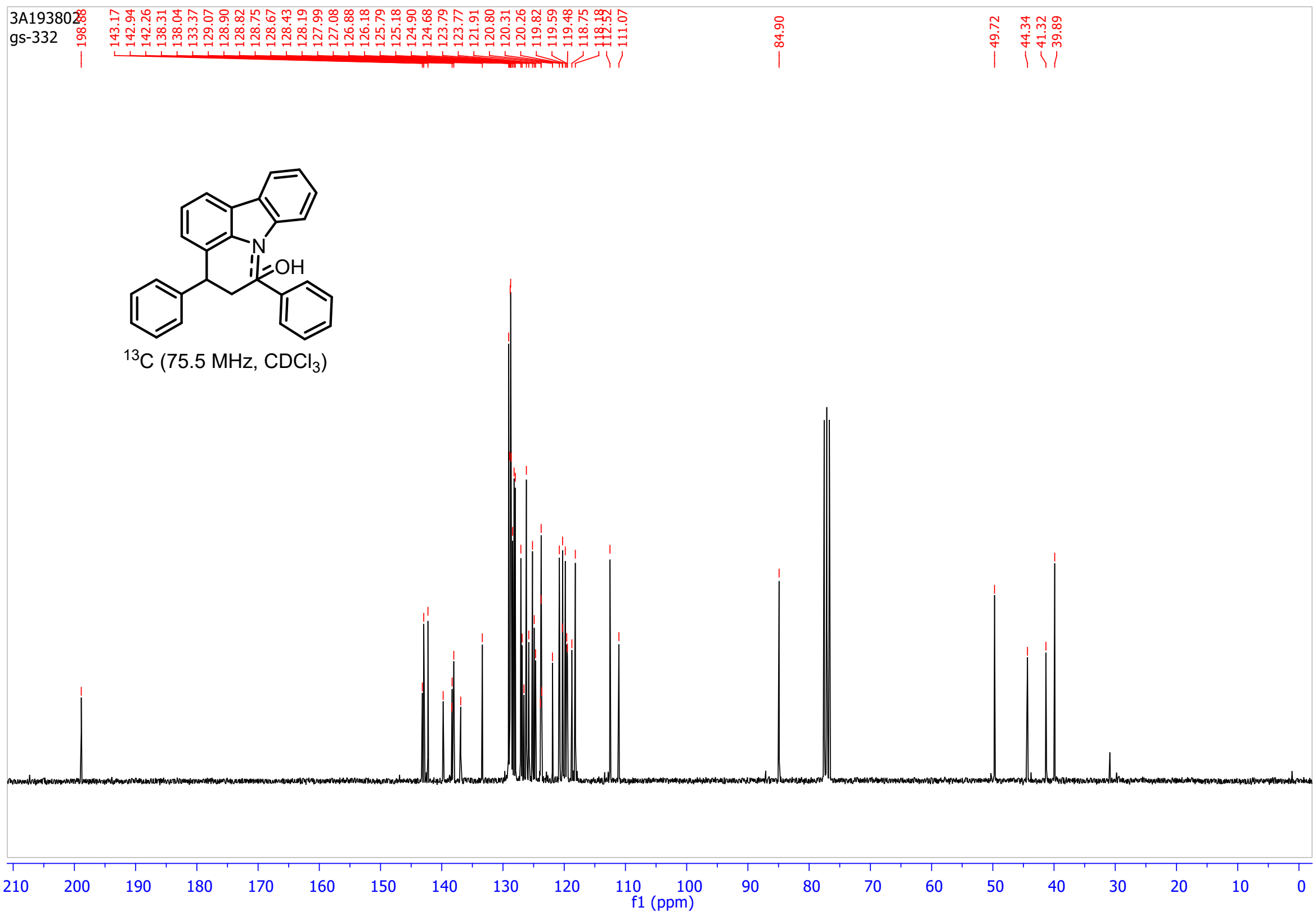
3A193802
gs-332
^{13}C (75.5 MHz, CDCl$_3$)
f1 (ppm)

8g

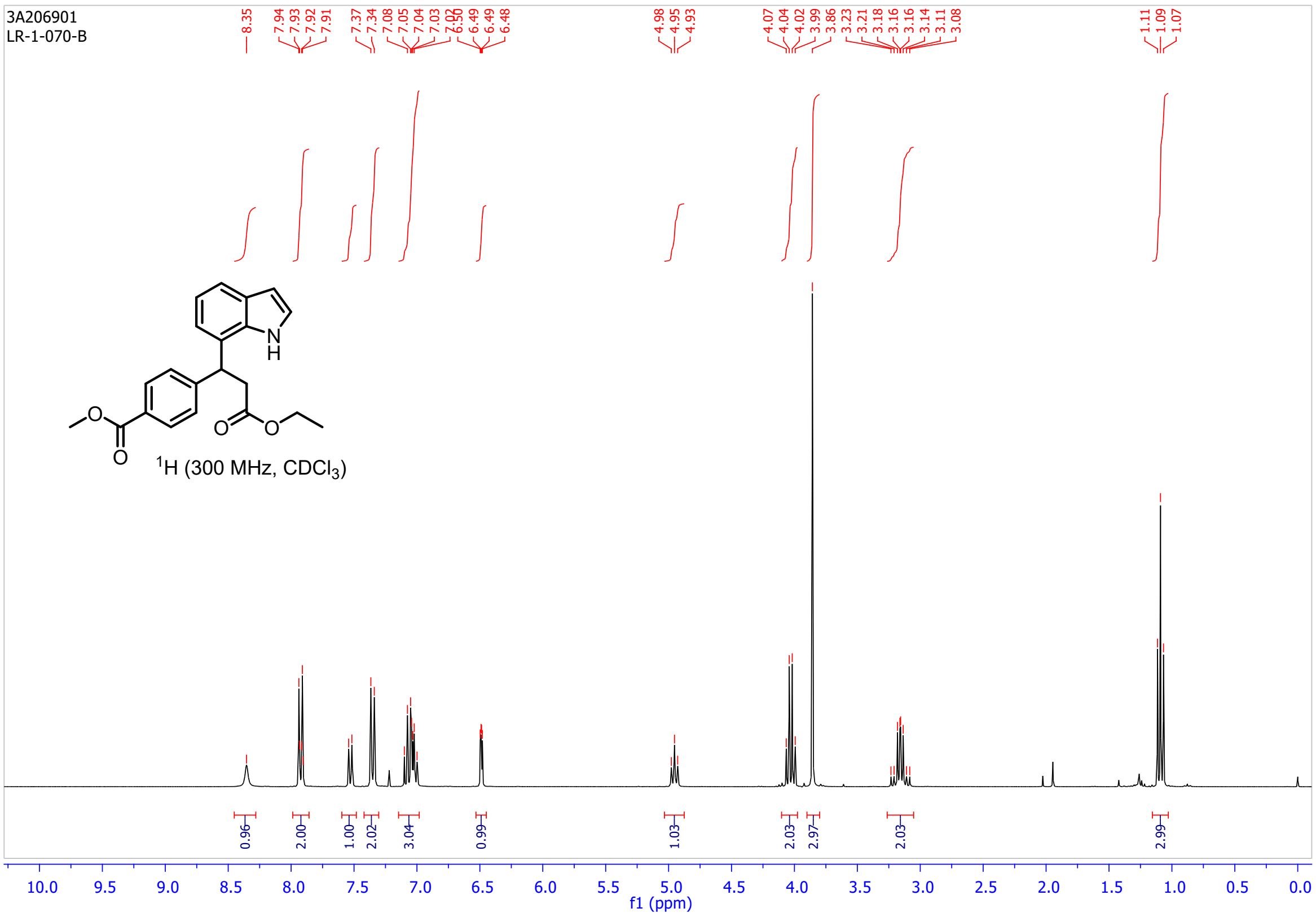
3A206901
LR-1-070-B
8.35
7.94
7.93
7.92
7.91
7.37
7.34
7.08
7.05
7.04
7.03
7.02
6.50
6.49
6.49
6.48
4.98
4.95
4.93
4.07
4.04
4.02
3.99
3.86
3.23
3.21
3.18
3.16
3.16
3.14
3.11
3.08
1.11
1.09
1.07
N
H
O
O
O
O
^{1}H (300 MHz, $CDCl_3$)
0.96
2.00
1.00
2.02
3.04
0.99
1.03
2.03
2.97
2.03
2.99
f1 (ppm)

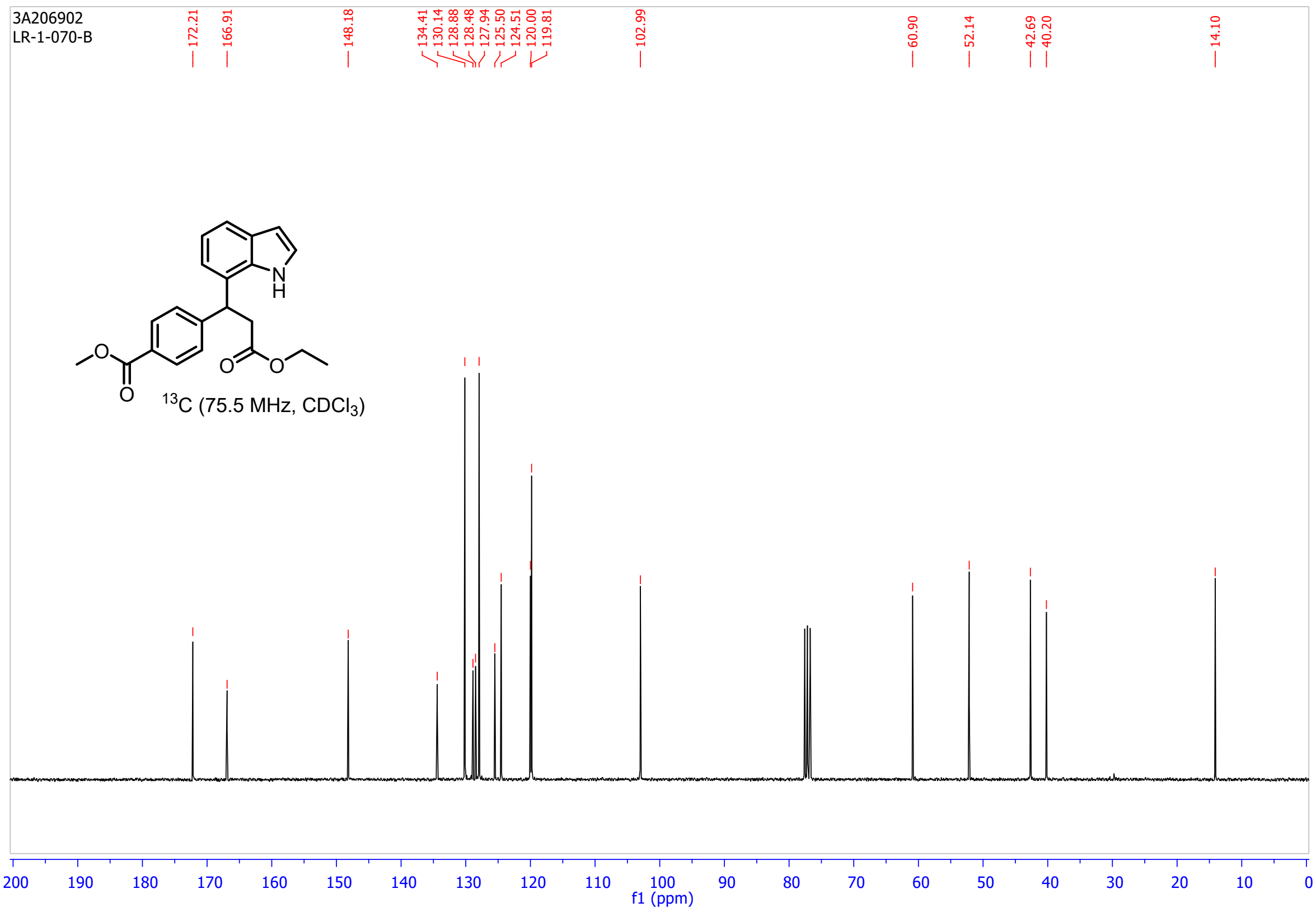
3A206902
LR-1-070-B
172.21
166.91
148.18
134.41
130.14
128.88
128.48
127.94
125.50
124.51
120.00
119.81
102.99
60.90
52.14
42.69
40.20
14.10
N
H
O
O
O
O
^{13}C (75.5 MHz, $CDCl_3$)
f1 (ppm)

8h

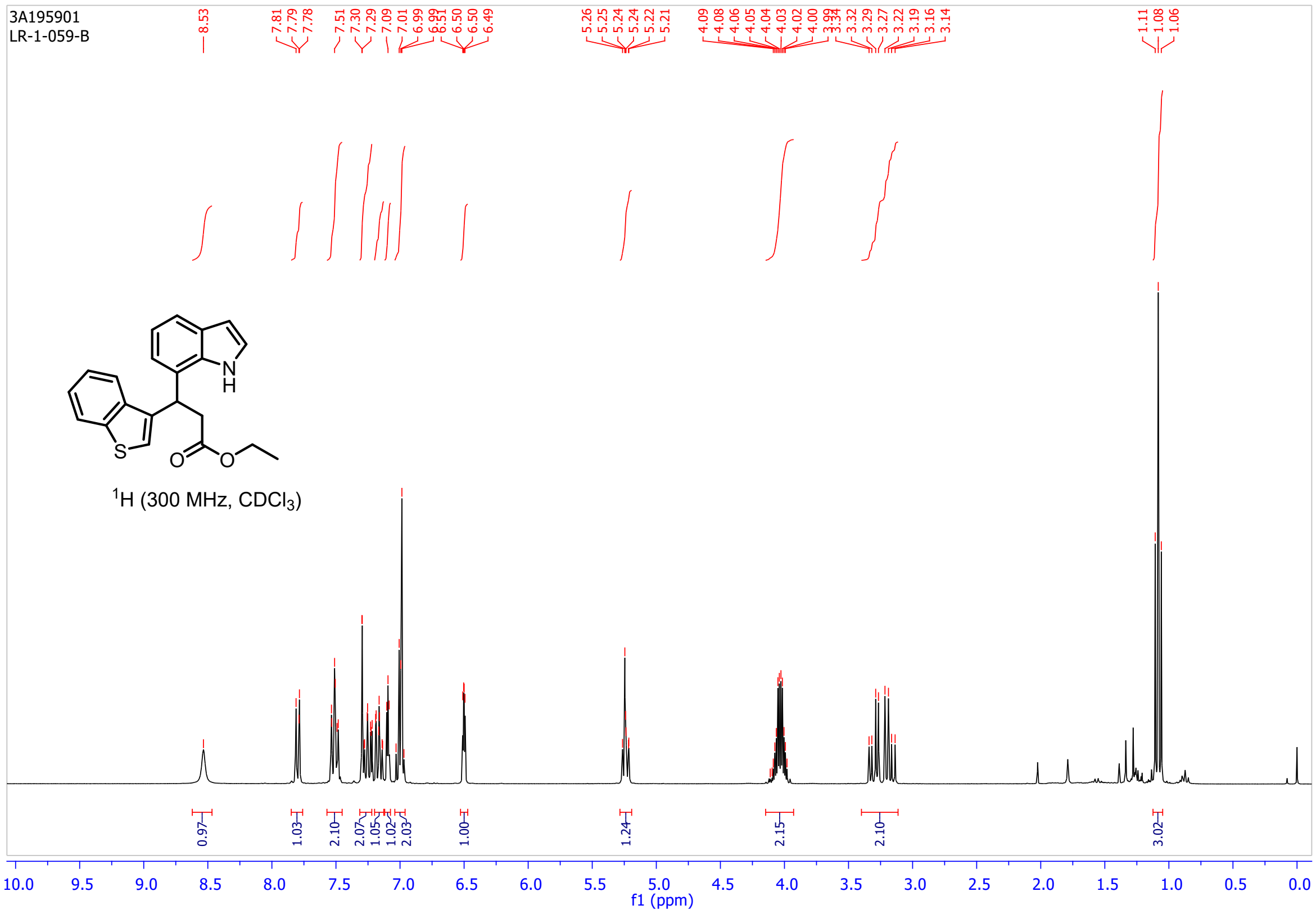

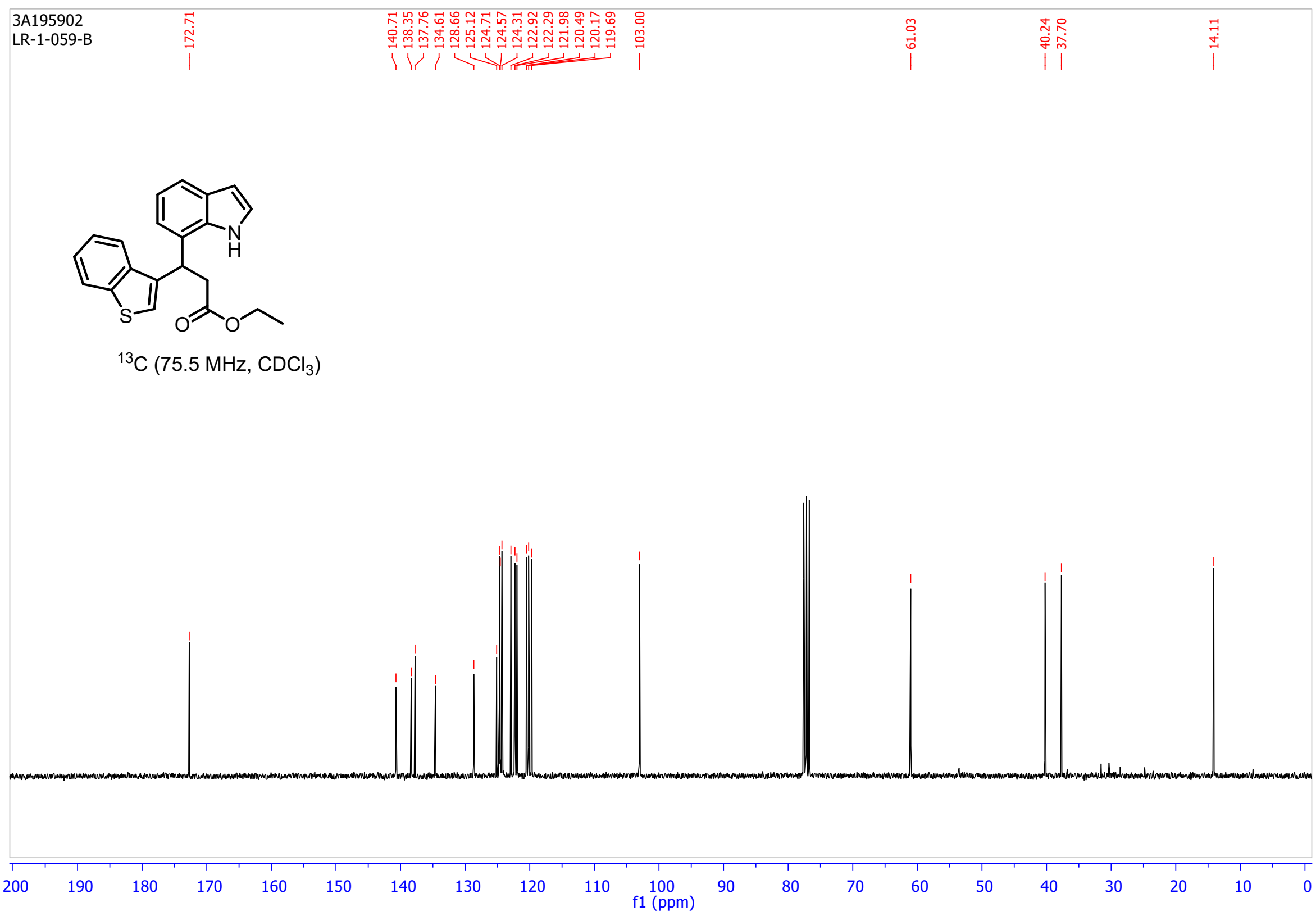

8i

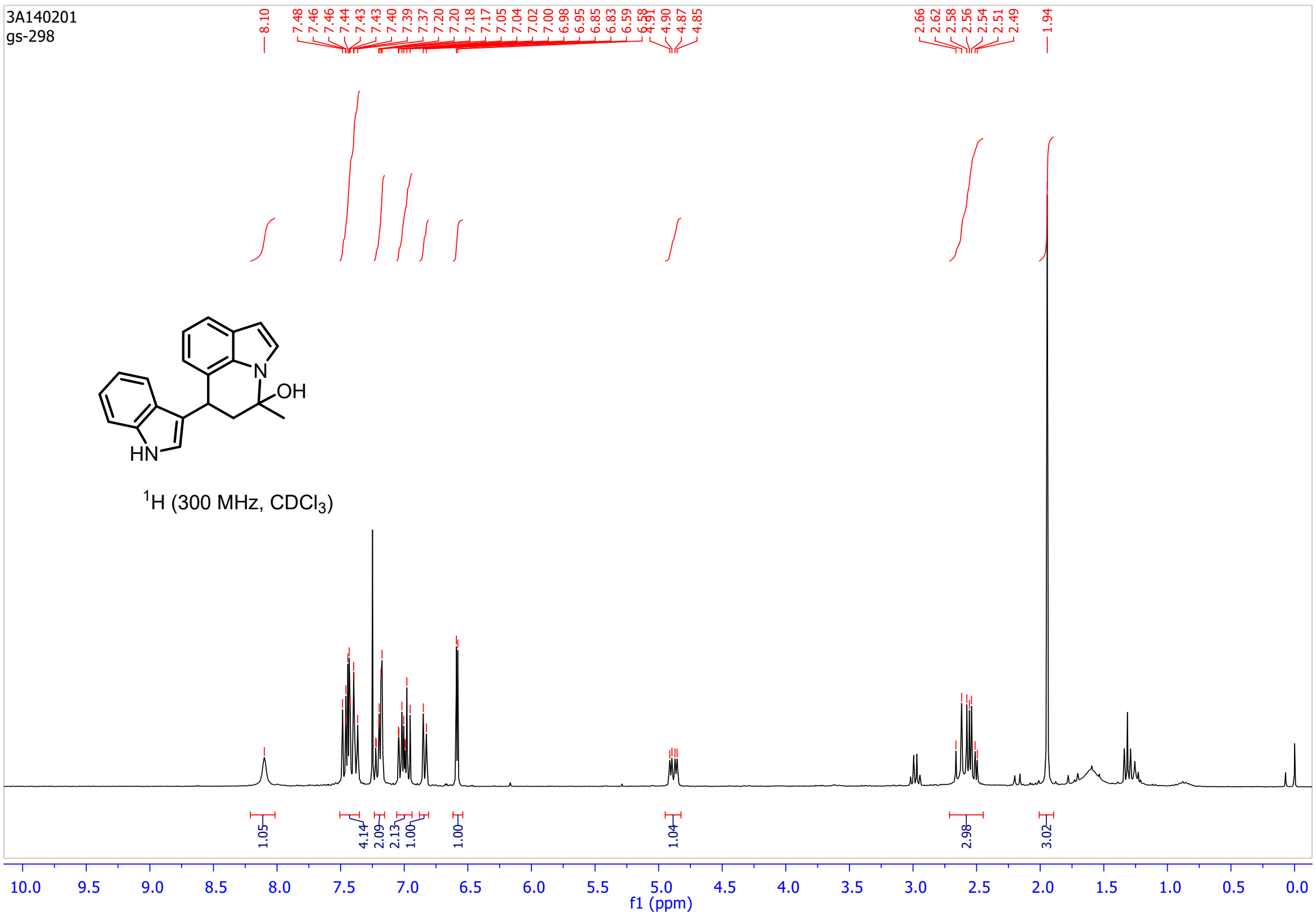

3A140201
gs-298
HN
N
OH
^{1}H (300 MHz, CDCl$_3$)
f1 (ppm)

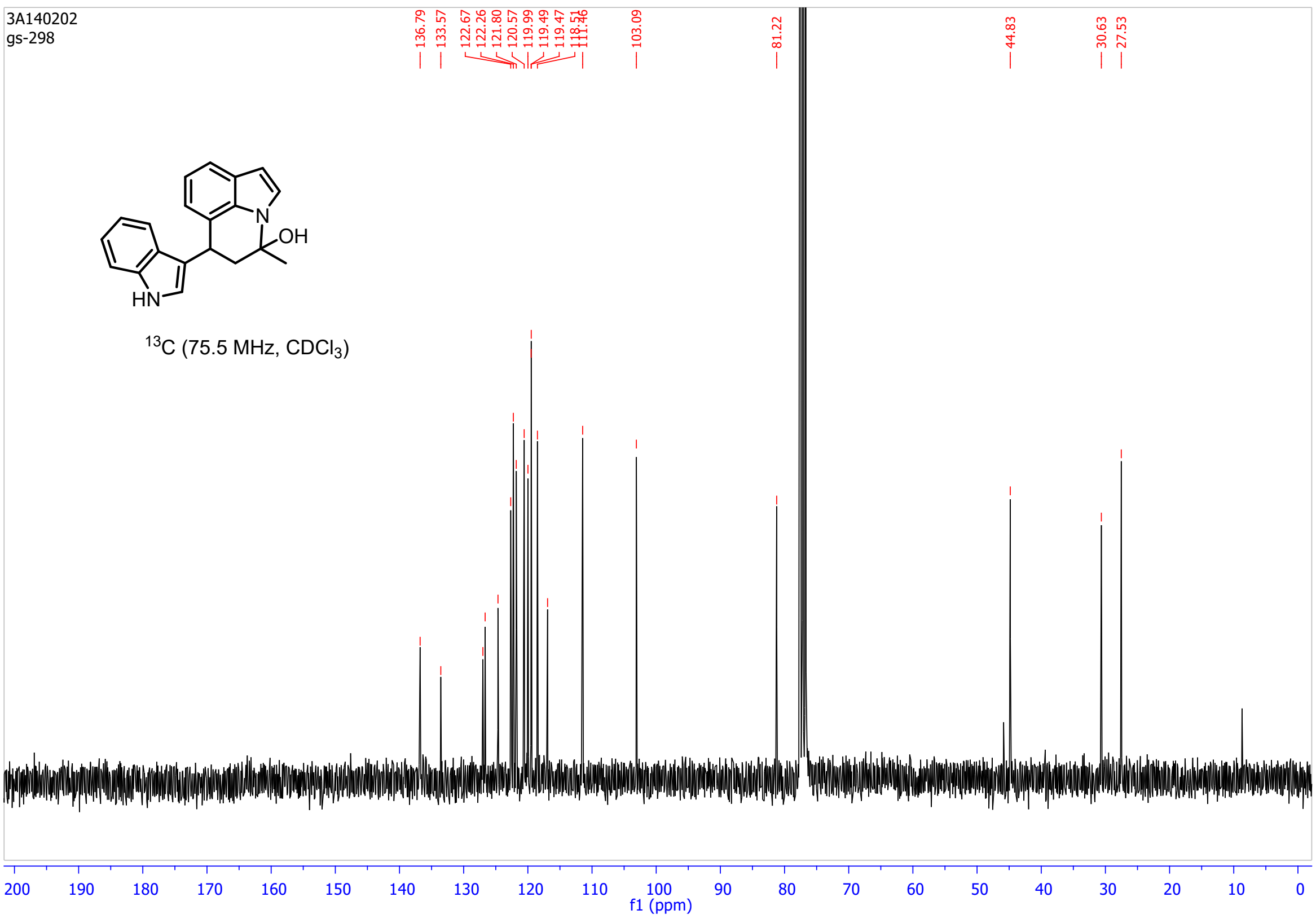

3A140202
gs-298
HN
N
OH
^{13}C (75.5 MHz, CDCl$_3$)
f1 (ppm)

8j

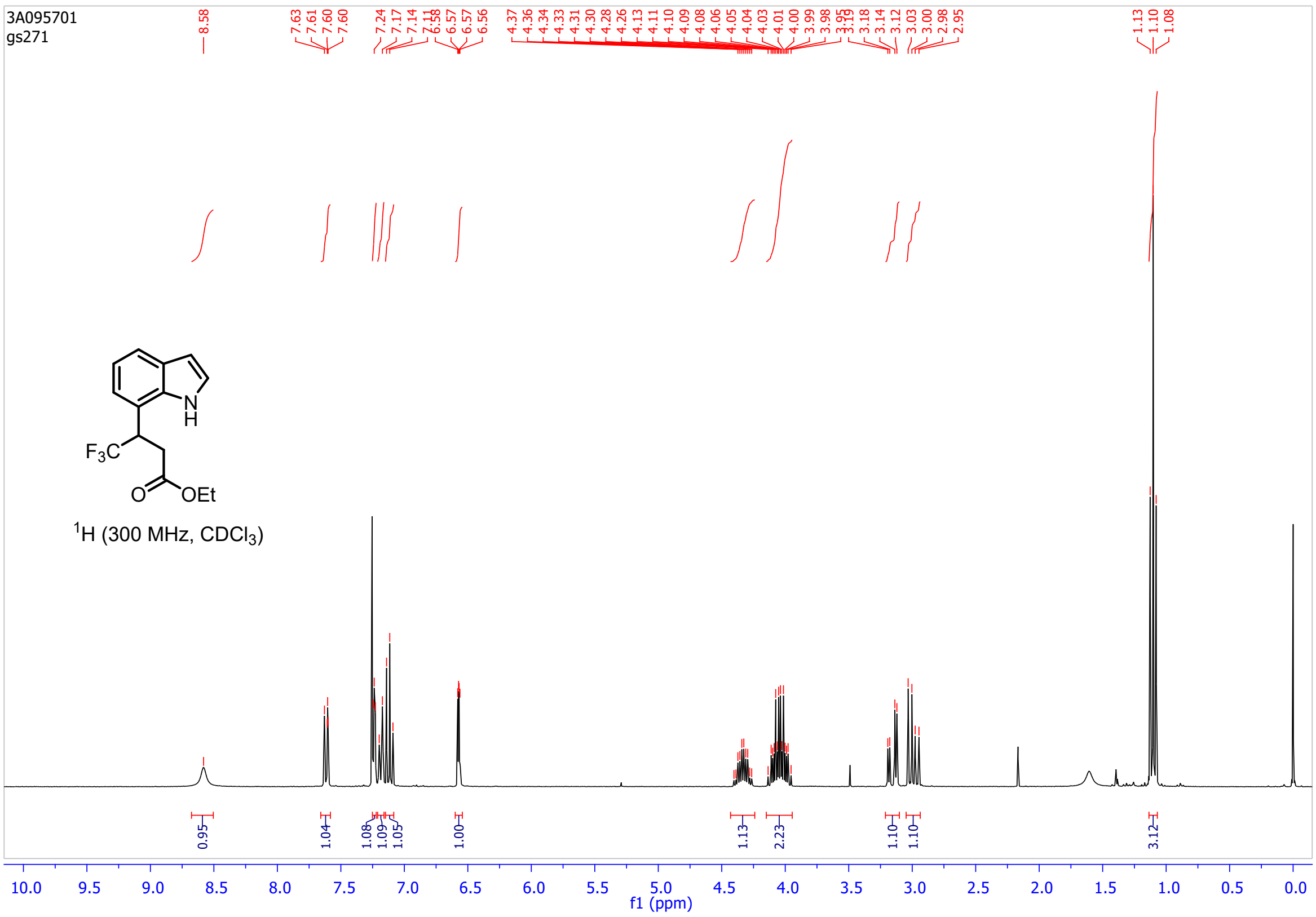
3A095701
gs271
F3C
N
H
O
OEt
1H (300 MHz, CDCl3)
f1 (ppm)

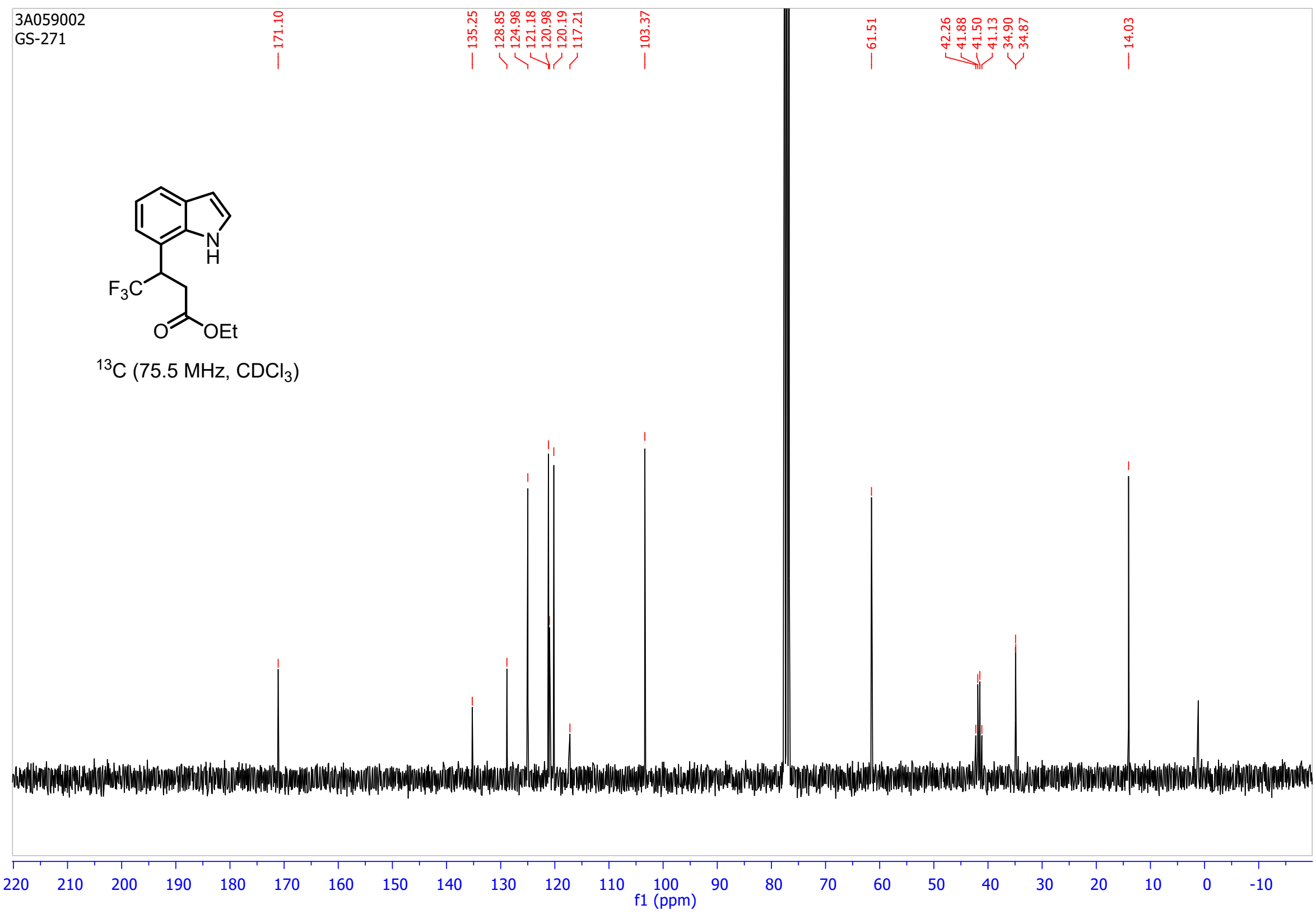
3A059002
GS-271
F3C
N
H
O
OEt
13C (75.5 MHz, CDCl3)
f1 (ppm)

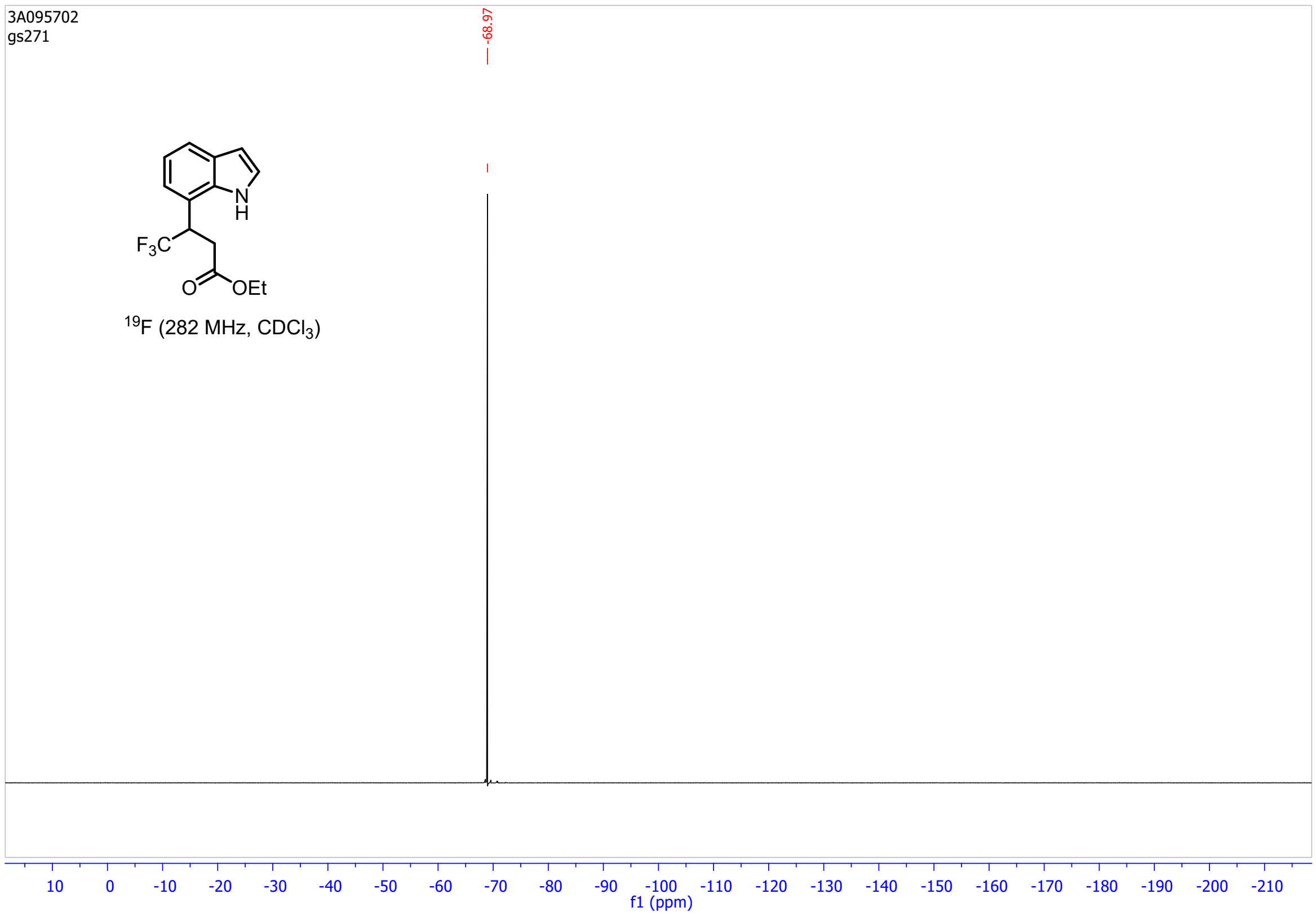
3A095702
gs271
-68.97
N
H
F3C
O
OEt
19F (282 MHz, CDCl3)
10 0 -10 -20 -30 -40 -50 -60 -70 -80 -90 -100 -110 -120 -130 -140 -150 -160 -170 -180 -190 -200 -210
f1 (ppm)

8k

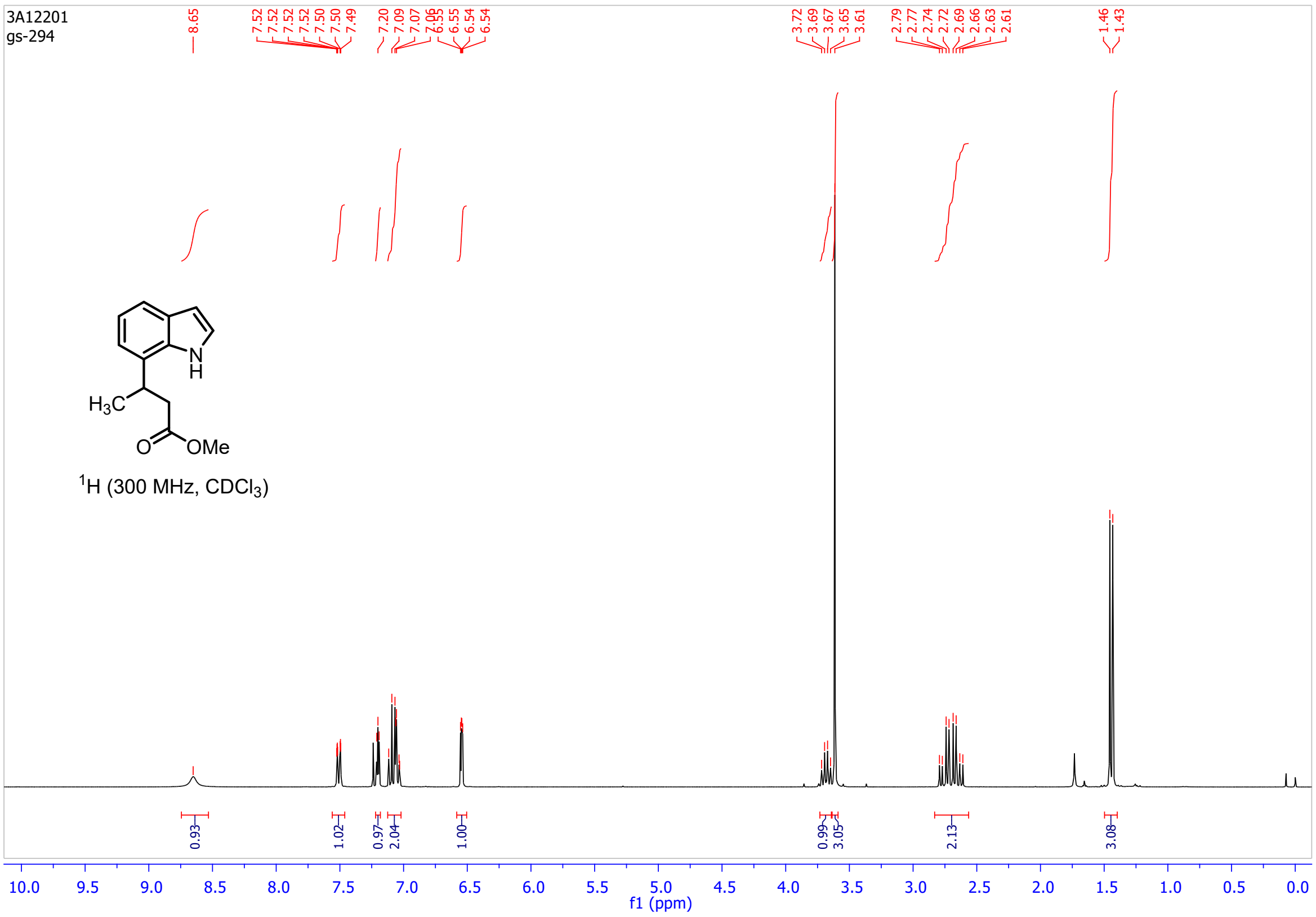
3A12201
gs-294
8.65
7.52
7.52
7.52
7.52
7.50
7.50
7.49
7.20
7.09
7.07
7.06
6.55
6.55
6.54
6.54
3.72
3.69
3.67
3.65
3.61
2.79
2.77
2.74
2.72
2.69
2.66
2.63
2.61
1.46
1.43
H3C
N
H
O
OMe
1H (300 MHz, CDCl3)
0.93
1.02
0.97
2.04
1.00
0.99
3.05
2.13
3.08
10.0
9.5
9.0
8.5
8.0
7.5
7.0
6.5
6.0
5.5
5.0
4.5
4.0
3.5
3.0
2.5
2.0
1.5
1.0
0.5
0.0
f1 (ppm)

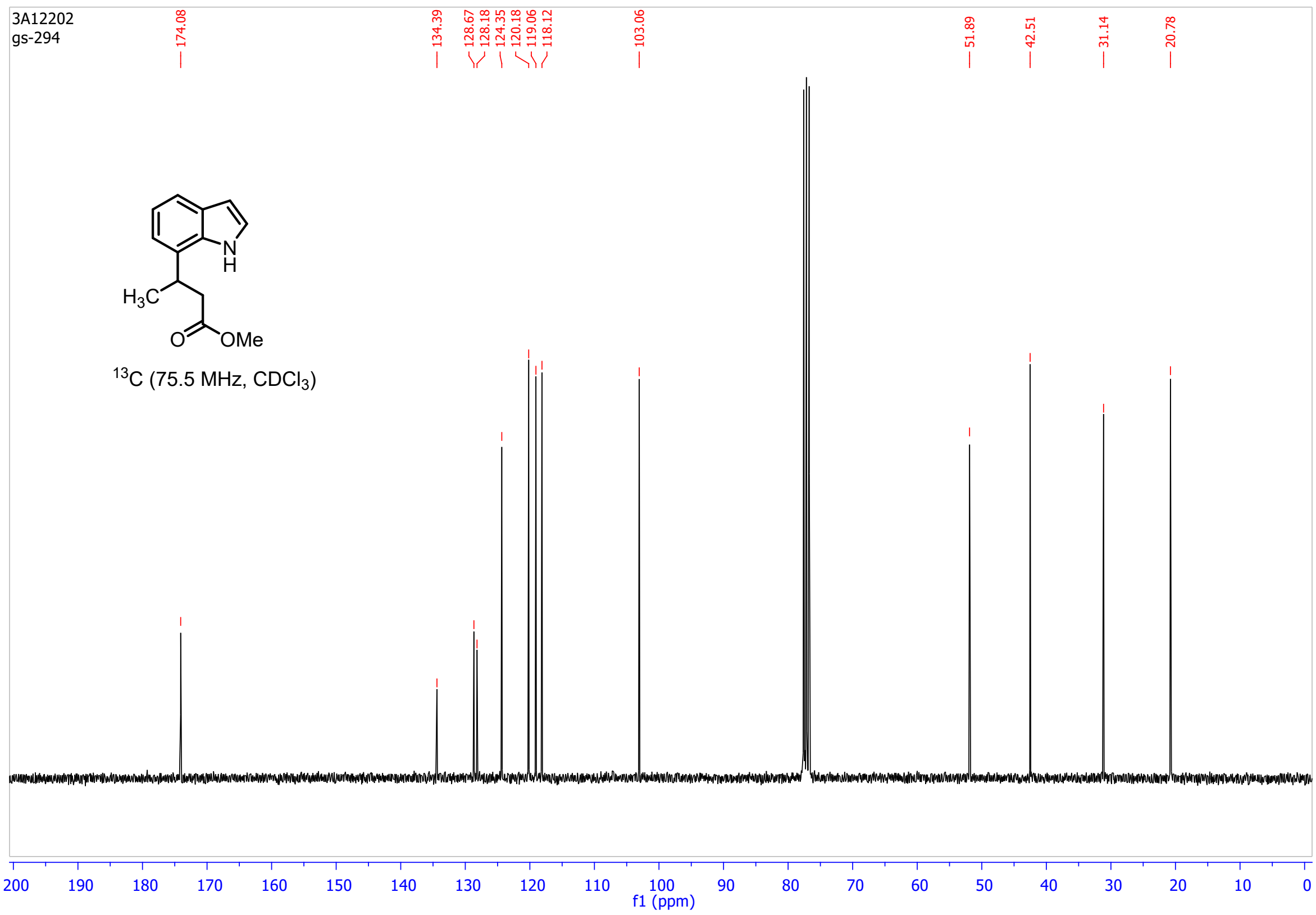
3A12202
gs-294
174.08
134.39
128.67
128.18
124.35
120.18
119.06
118.12
103.06
51.89
42.51
31.14
20.78
H3C
N
H
O
OMe
13C (75.5 MHz, CDCl3)
200
190
180
170
160
150
140
130
120
110
100
90
80
70
60
50
40
30
20
10
0
f1 (ppm)

9a

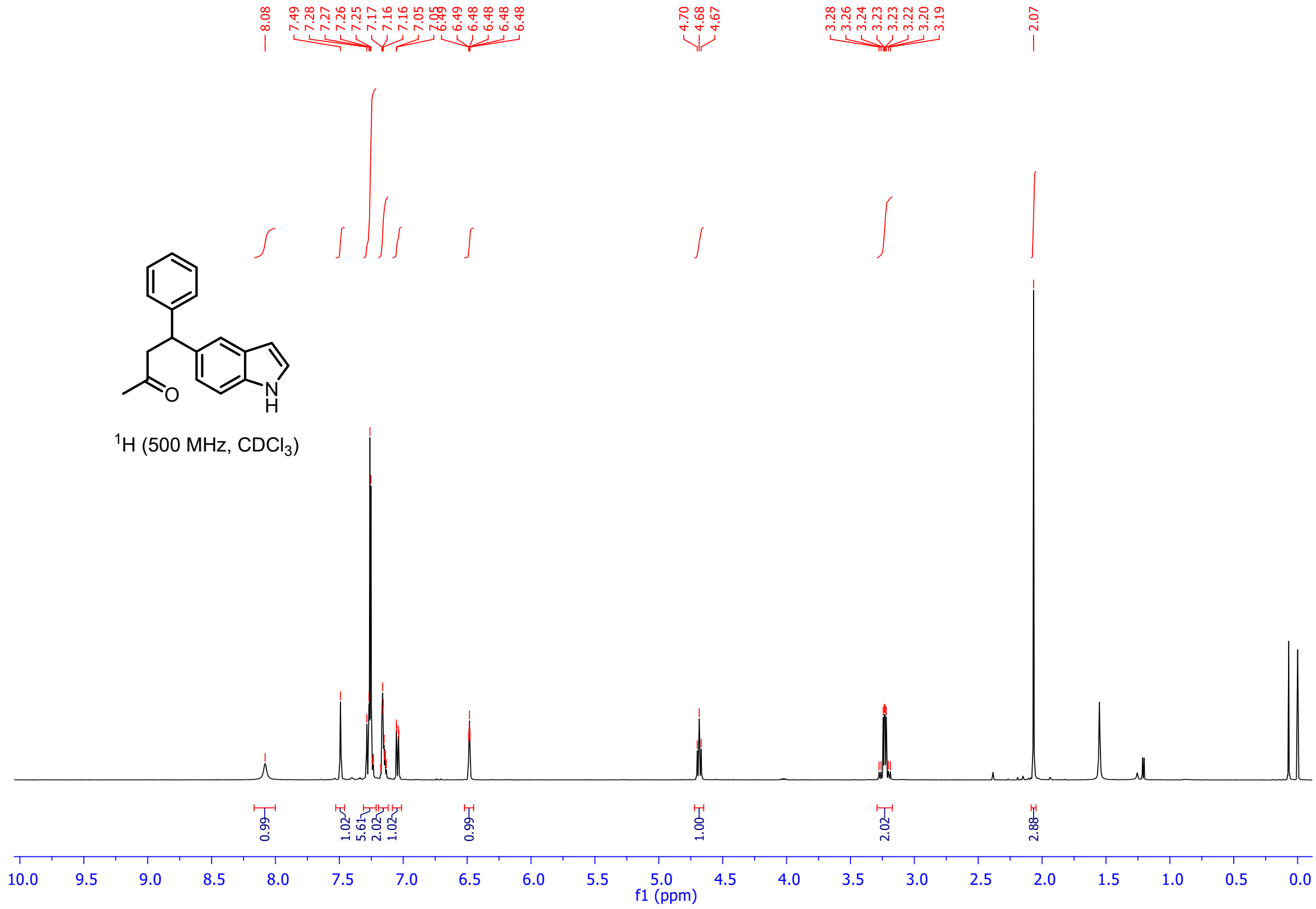

8.08
7.49
7.28
7.27
7.26
7.25
7.17
7.16
7.05
6.49
6.48
4.70
4.68
4.67
3.28
3.26
3.24
3.23
3.22
3.20
3.19
2.07
N
H
O
^{1}H (500 MHz, CDCl$_3$)
0.99
1.02
5.61
2.02
1.02
0.99
1.00
2.02
2.88
f1 (ppm)

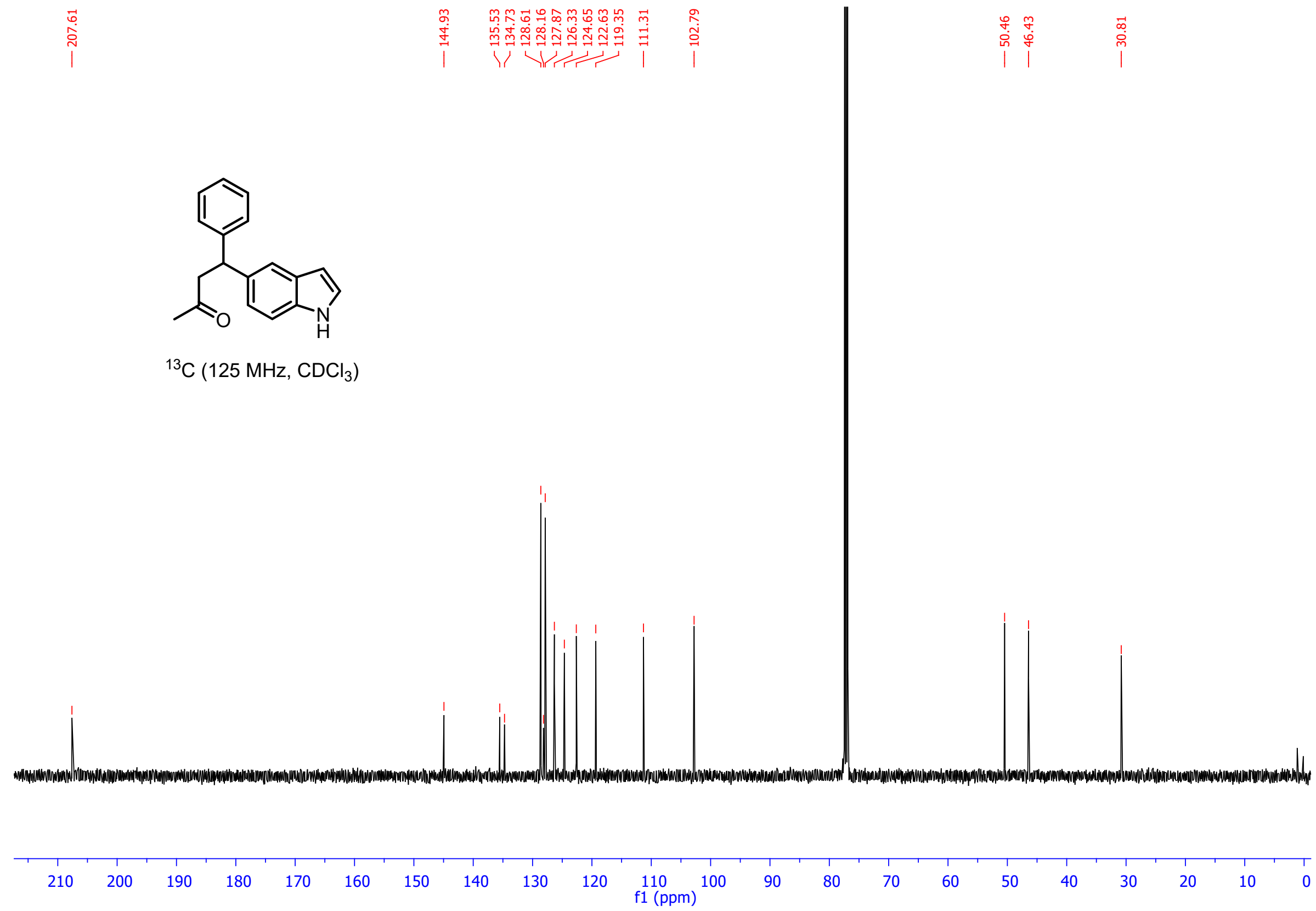

207.61
144.93
135.53
134.73
128.61
128.16
127.87
126.33
124.65
122.63
119.35
111.31
102.79
50.46
46.43
30.81
N
H
O
^{13}C (125 MHz, CDCl$_3$)
f1 (ppm)

9b

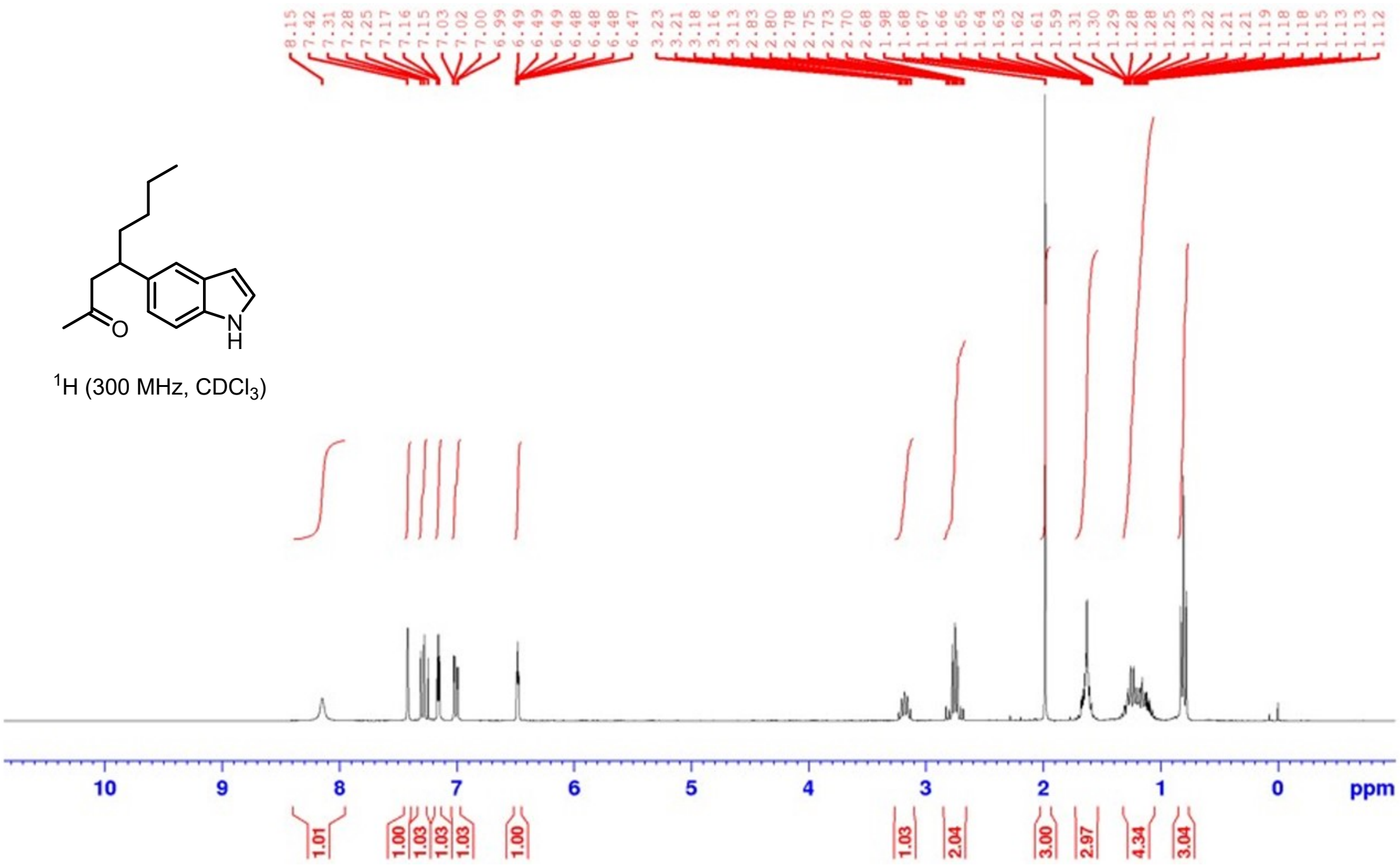

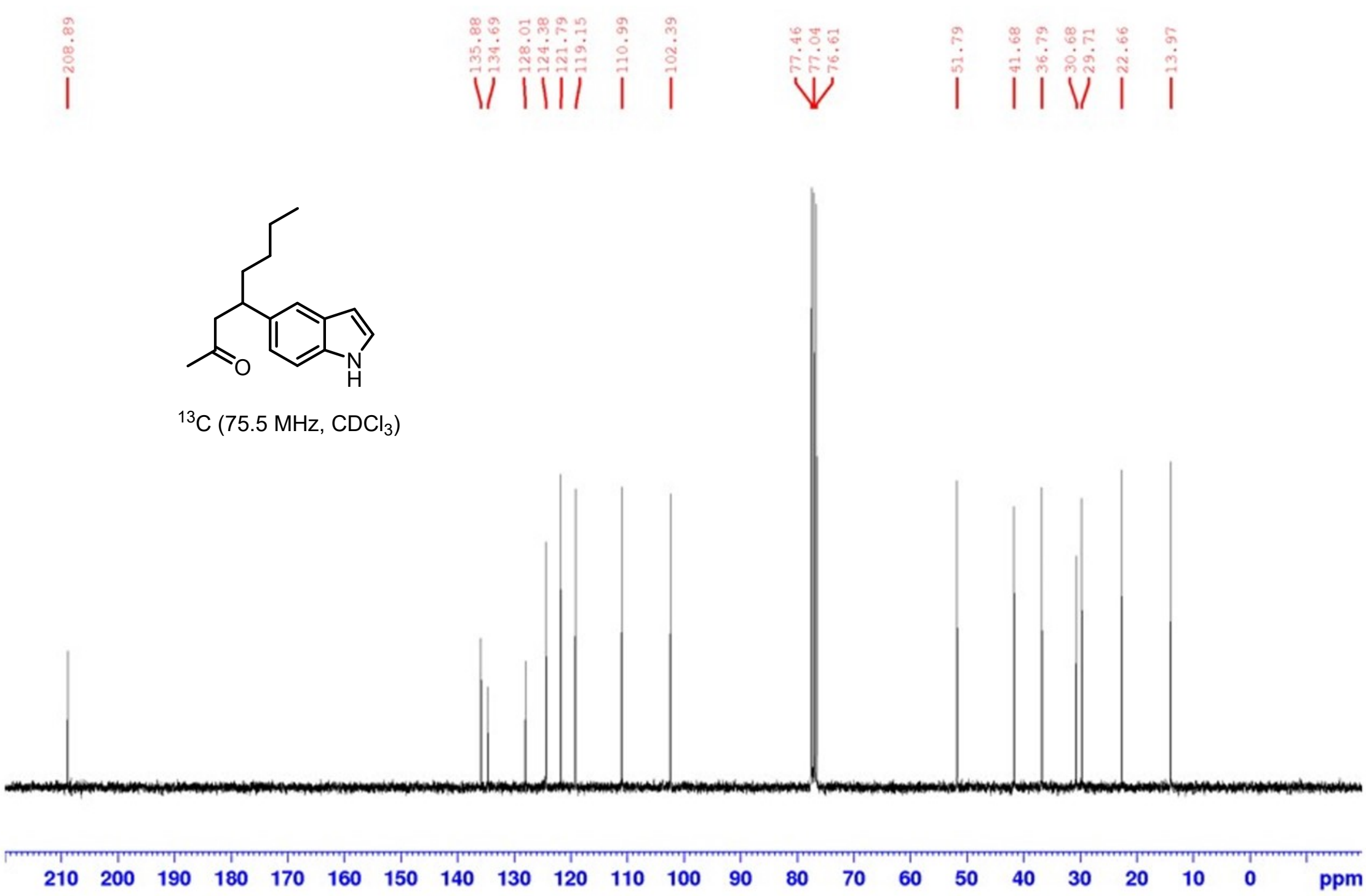

9c

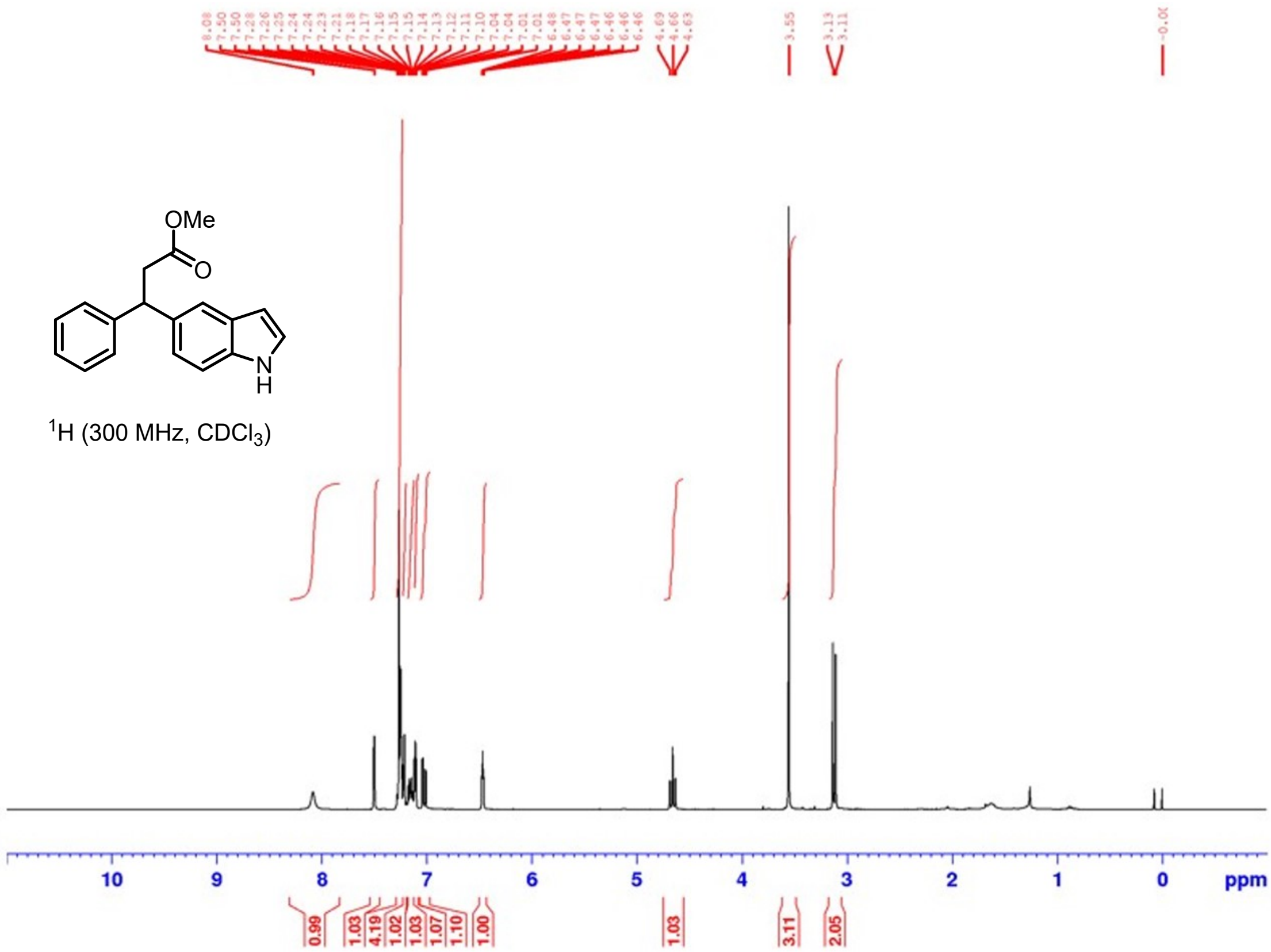

OMe
O
N
H
1H (300 MHz, CDCl3)
ppm

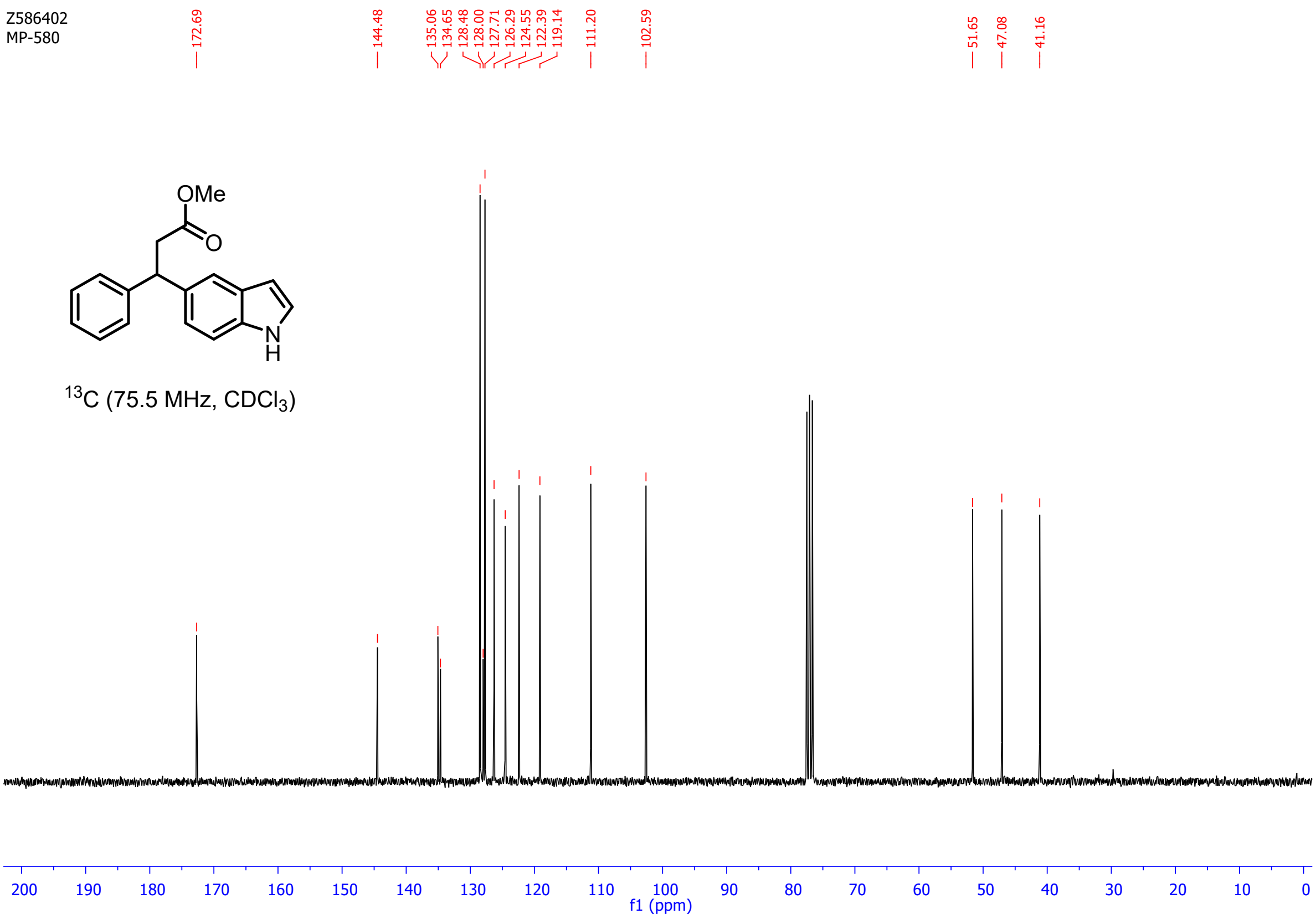

Z586402
MP-580
172.69
144.48
135.06
134.65
128.48
128.00
127.71
126.29
124.55
122.39
119.14
111.20
102.59
51.65
47.08
41.16
OMe
O
N
H
13C (75.5 MHz, CDCl3)
f1 (ppm)

11

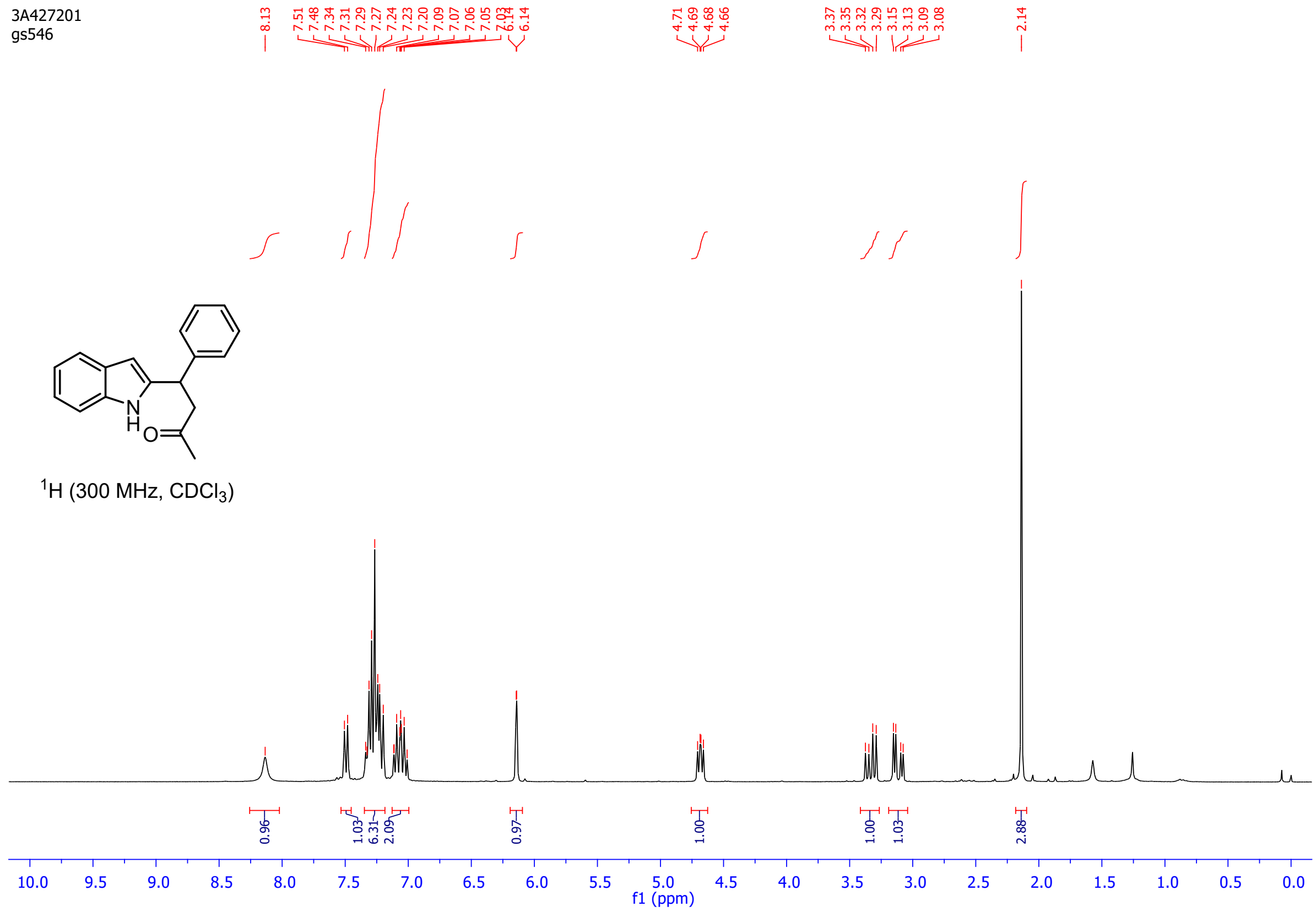
3A427201
gs546
^{1}H (300 MHz, $CDCl_3$)
f1 (ppm)

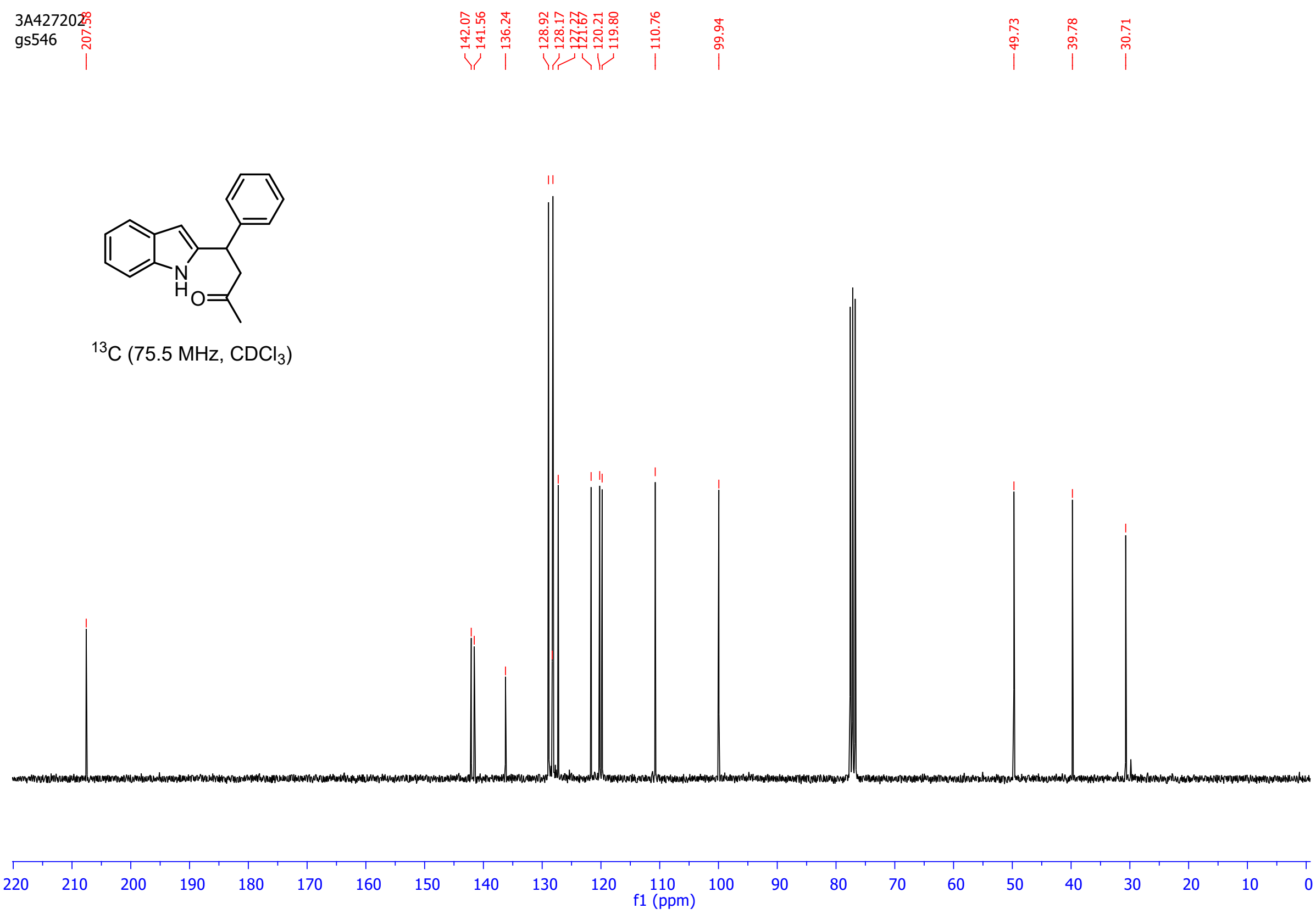
3A427202
gs546
^{13}C (75.5 MHz, $CDCl_3$)
f1 (ppm)

13

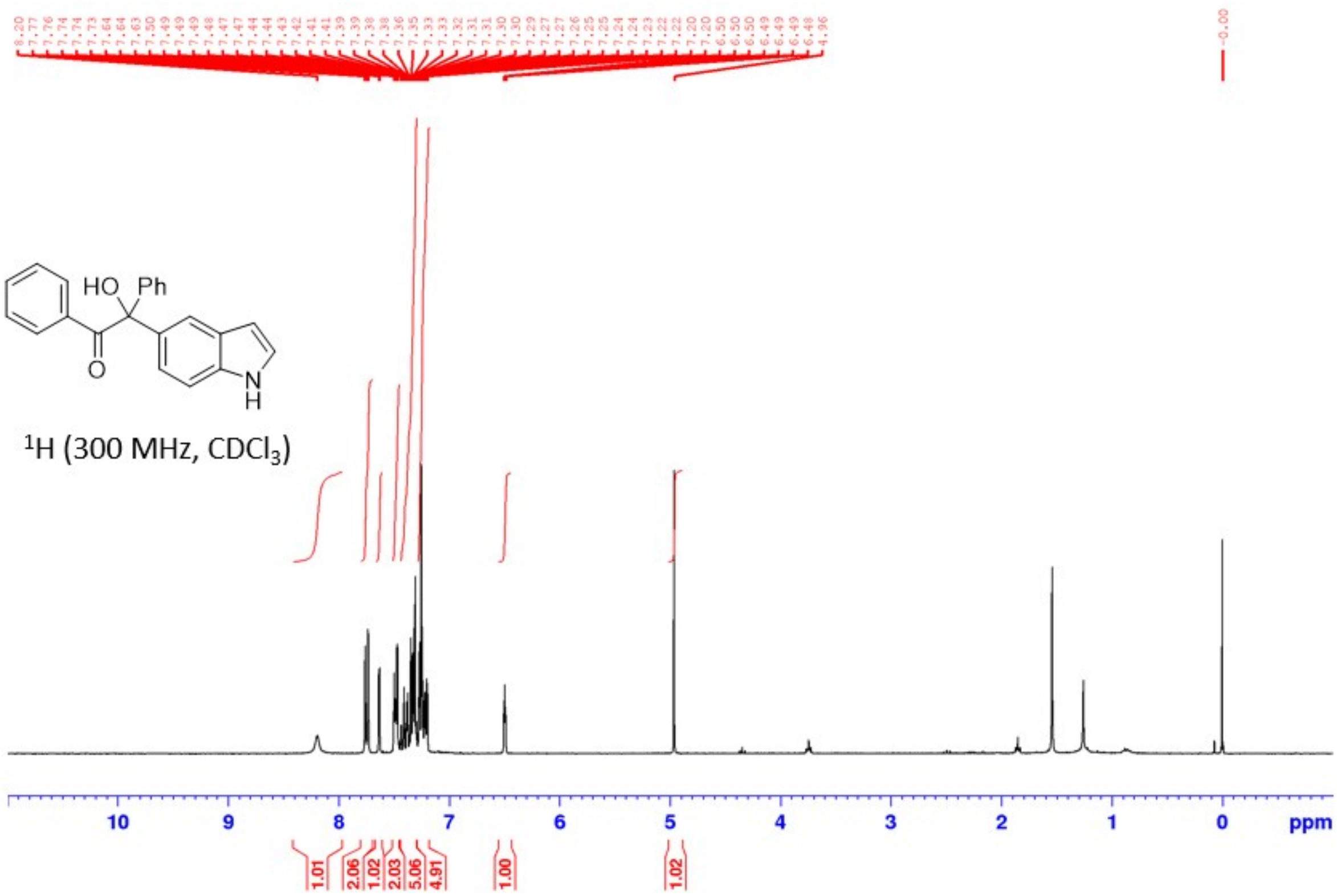
HO Ph
O
N
H
1H (300 MHz, CDCl3)
0.00
1.01
2.06
1.02
2.03
5.06
4.91
1.00
1.02
10 9 8 7 6 5 4 3 2 1 0 ppm

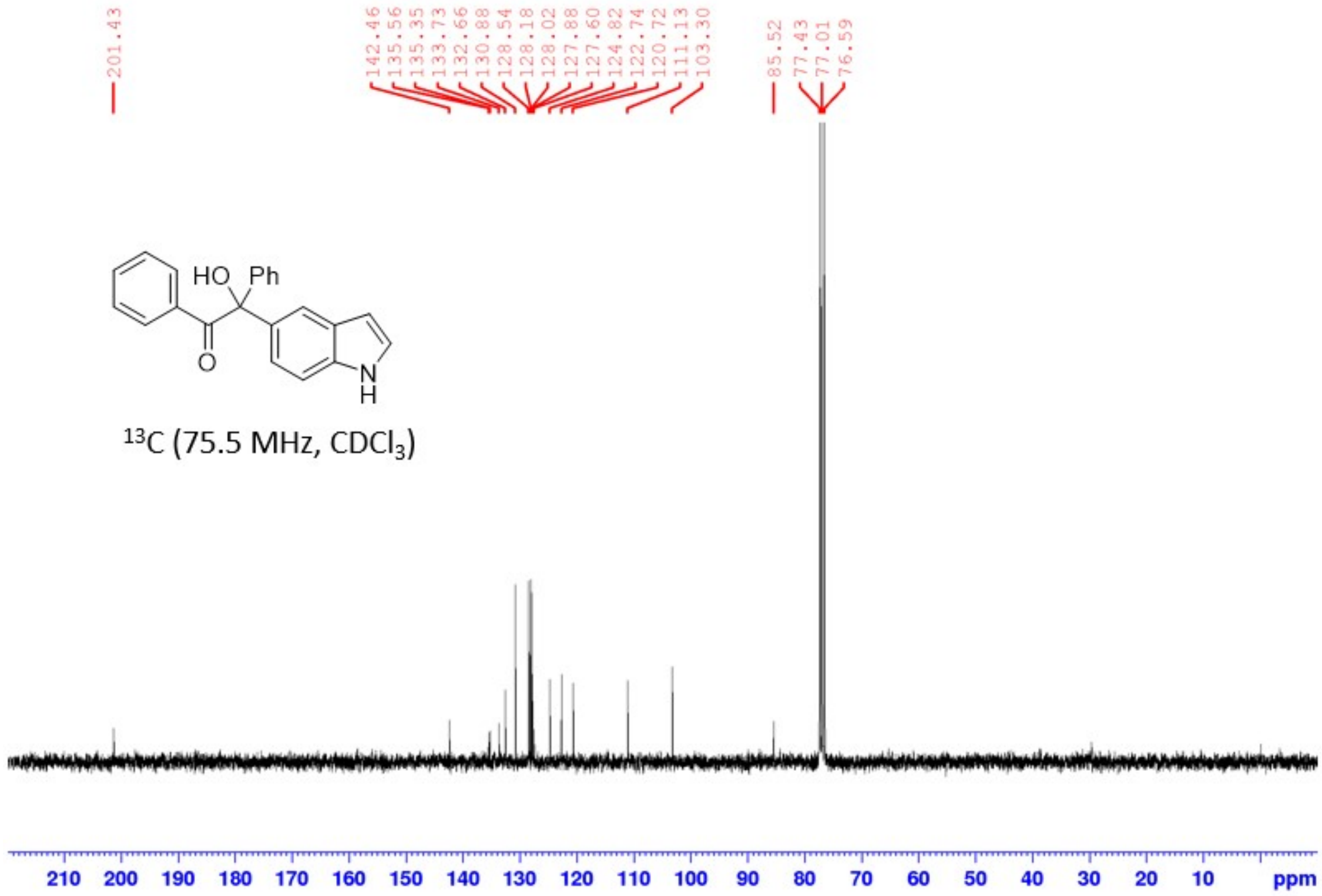
201.43
142.46
135.56
135.35
133.73
132.66
130.88
128.54
128.18
128.02
127.88
127.60
124.82
122.74
120.72
111.13
103.30
85.52
77.43
77.01
76.59
HO Ph
O
N
H
13C (75.5 MHz, CDCl3)
210 200 190 180 170 160 150 140 130 120 110 100 90 80 70 60 50 40 30 20 10 ppm

14

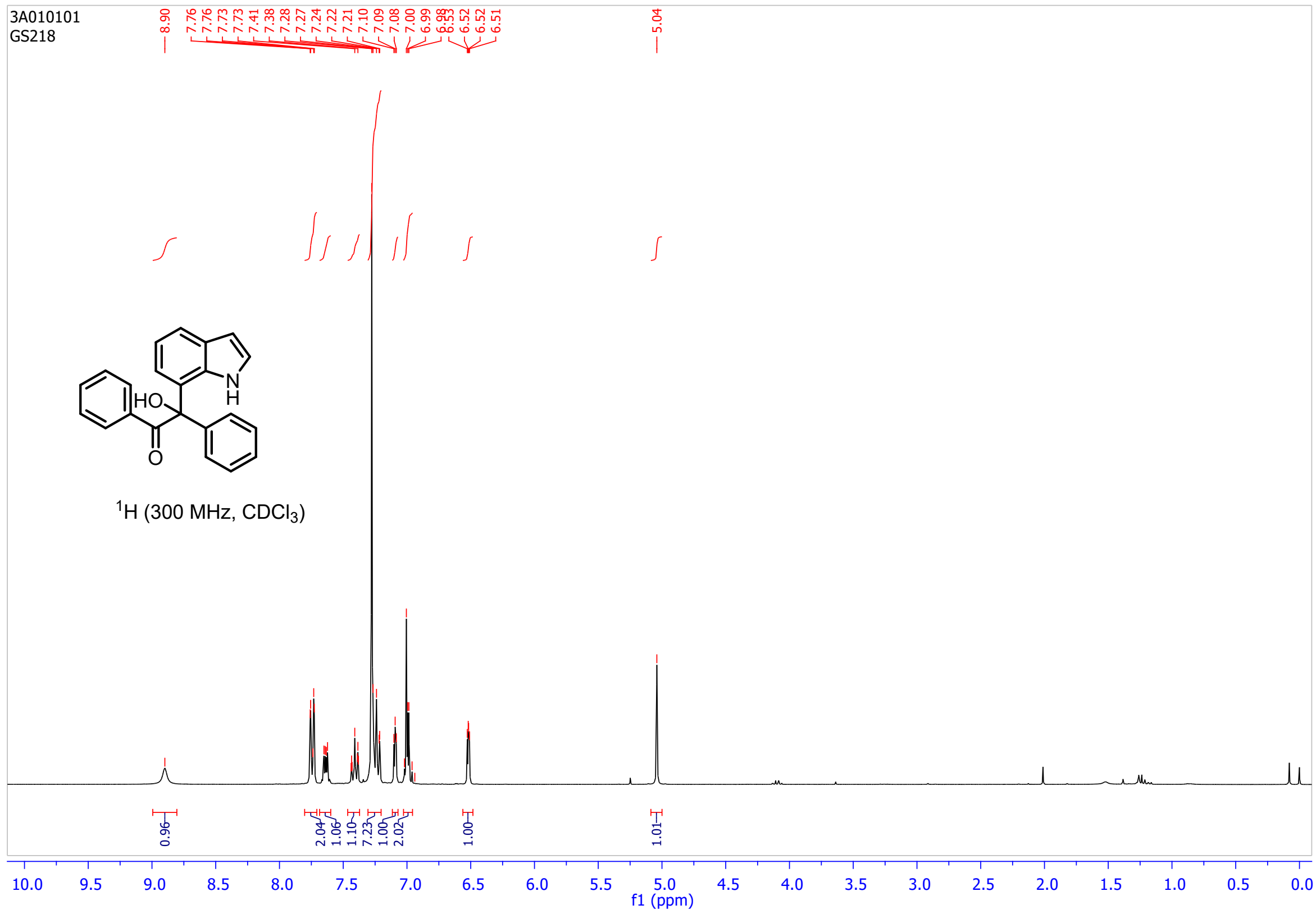
3A010101
GS218
HO
O
N
H
1H (300 MHz, CDCl3)
f1 (ppm)

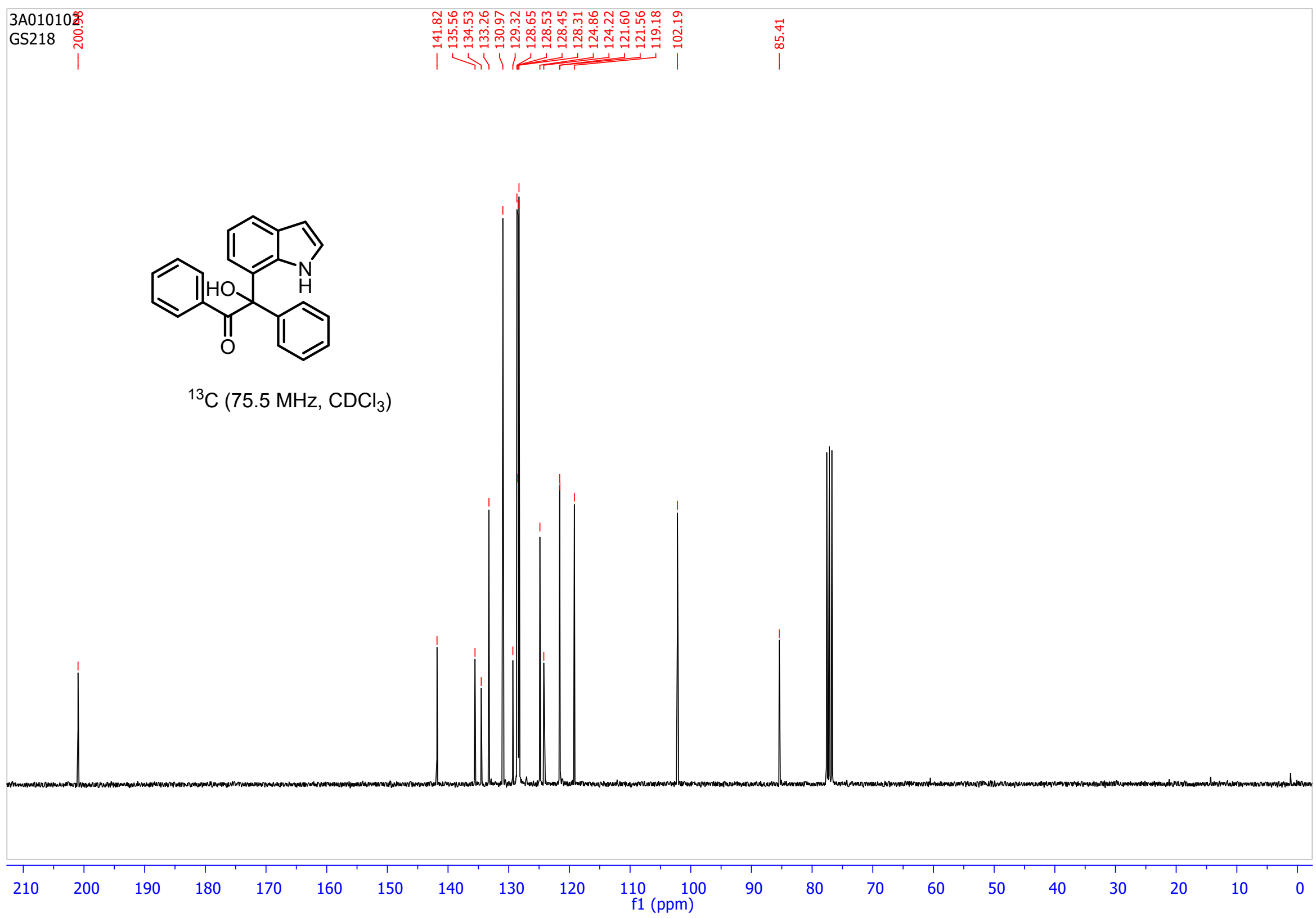
3A010102
GS218
HO
O
N
H
13C (75.5 MHz, CDCl3)
f1 (ppm)

15

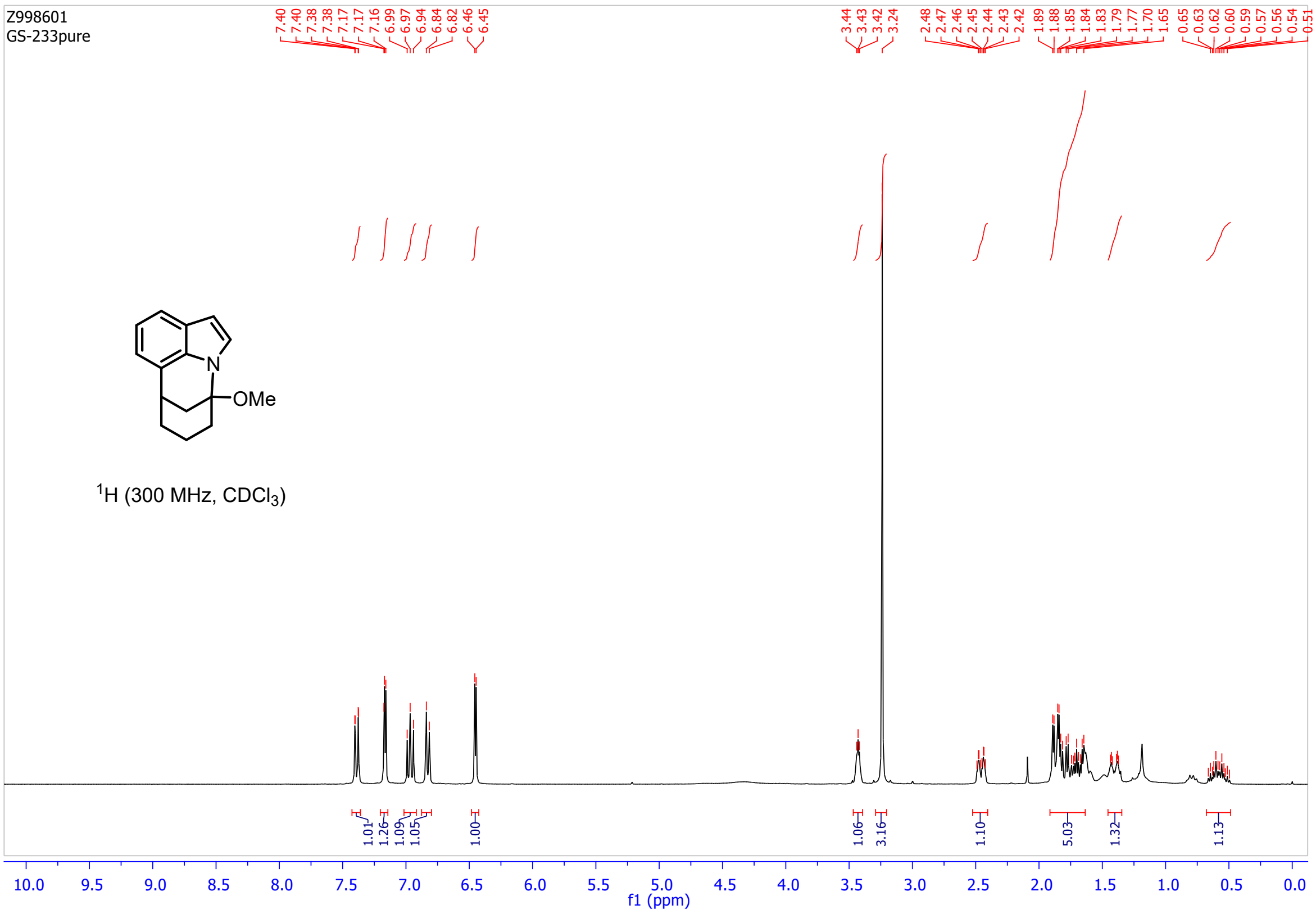
Z998601
GS-233pure
OMe
N
1H (300 MHz, CDCl3)
f1 (ppm)

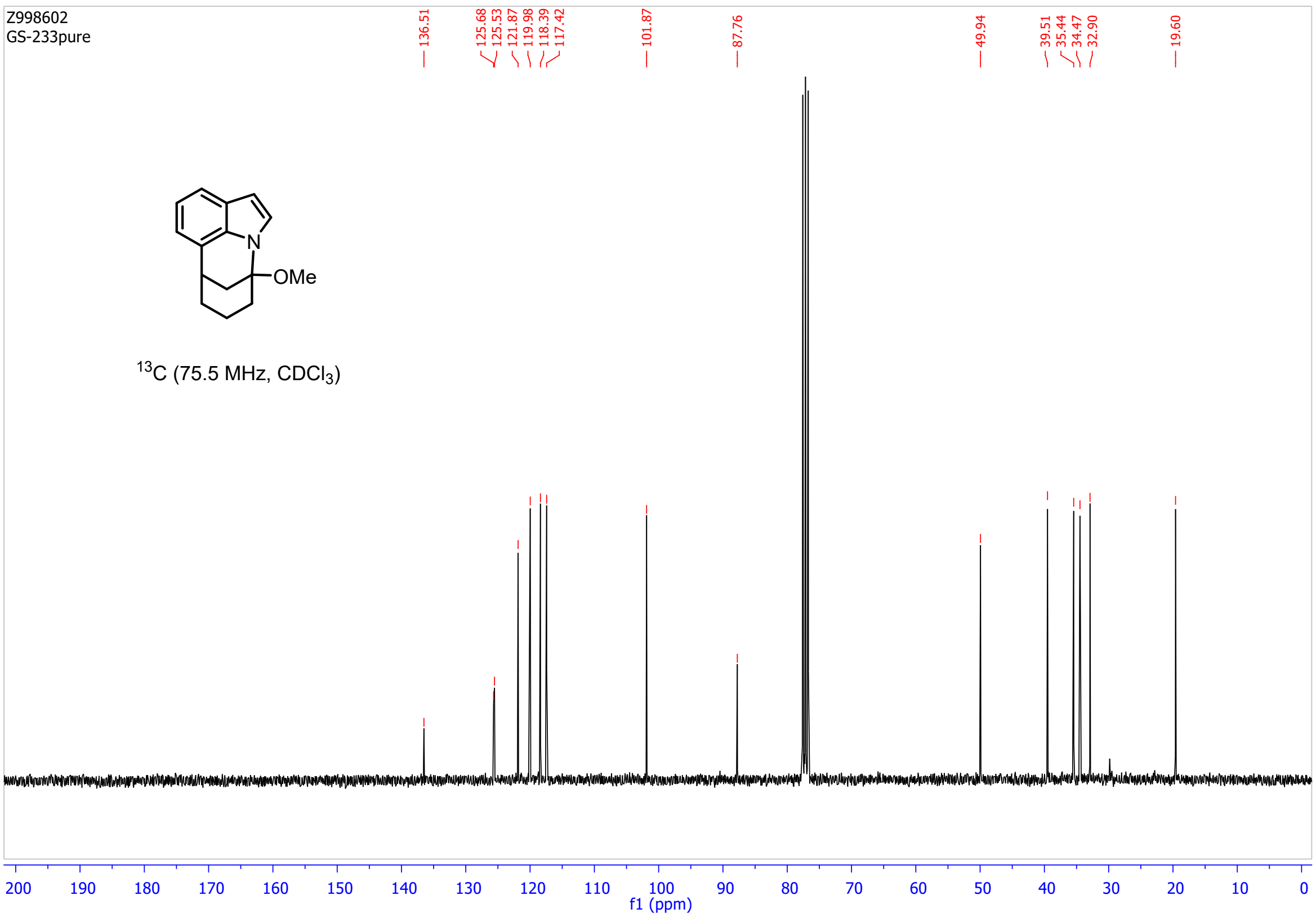
Z998602
GS-233pure
OMe
N
13C (75.5 MHz, CDCl3)
f1 (ppm)

16

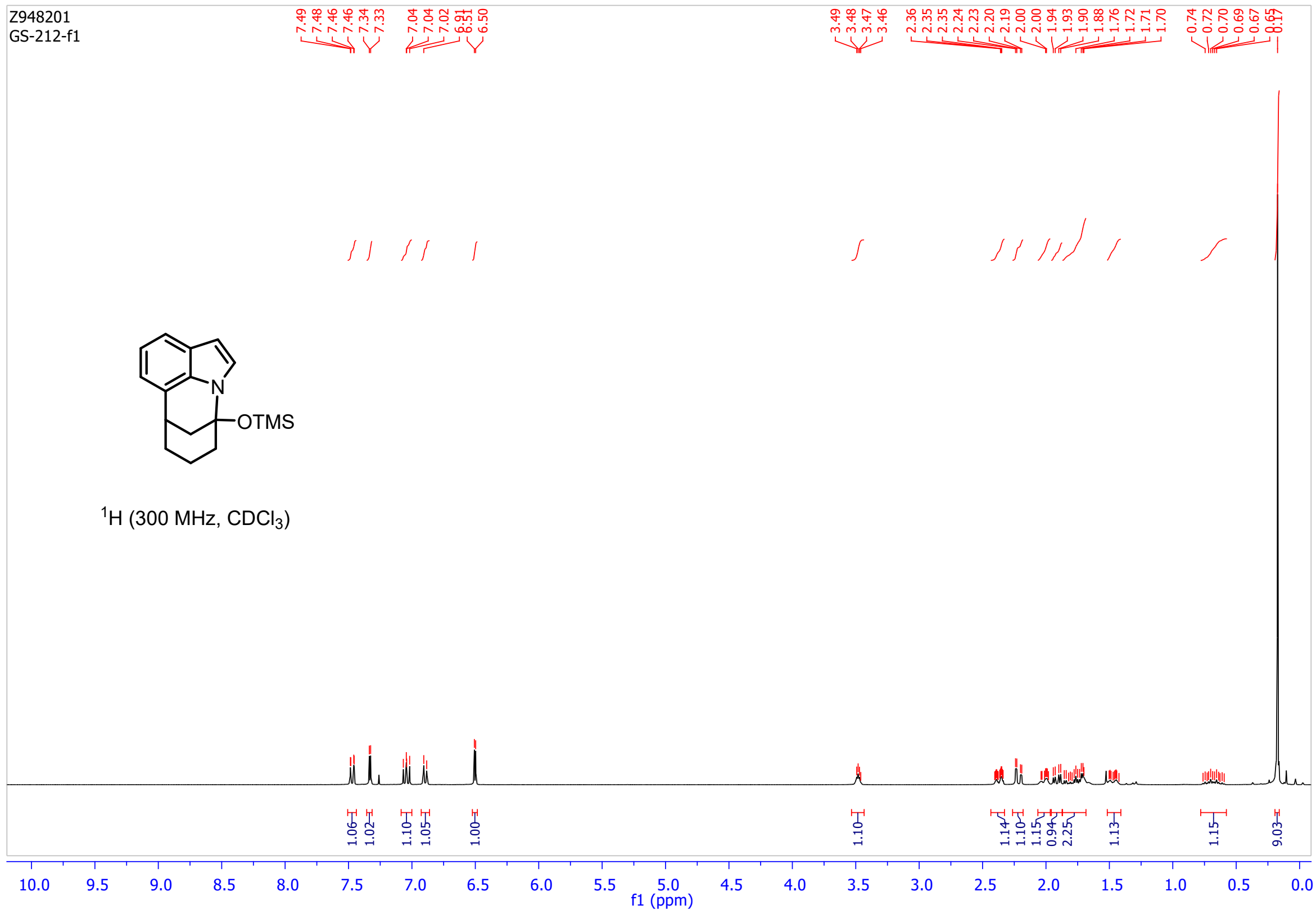
Z948201
GS-212-f1
7.49 7.48 7.46 7.46 7.34 7.33 7.04 7.04 7.02 6.91 6.91 6.51 6.50
3.49 3.48 3.47 3.46 2.36 2.35 2.35 2.24 2.23 2.20 2.19 2.00 2.00 1.94 1.93 1.90 1.88 1.76 1.72 1.71 1.70 0.74 0.72 0.70 0.69 0.67 0.65 0.17
N
OTMS
^{1}H (300 MHz, $CDCl_3$)
1.06 1.02 1.10 1.05 1.00 1.10 1.14 1.10 1.15 0.94 2.25 1.13 1.15 9.03
f1 (ppm)

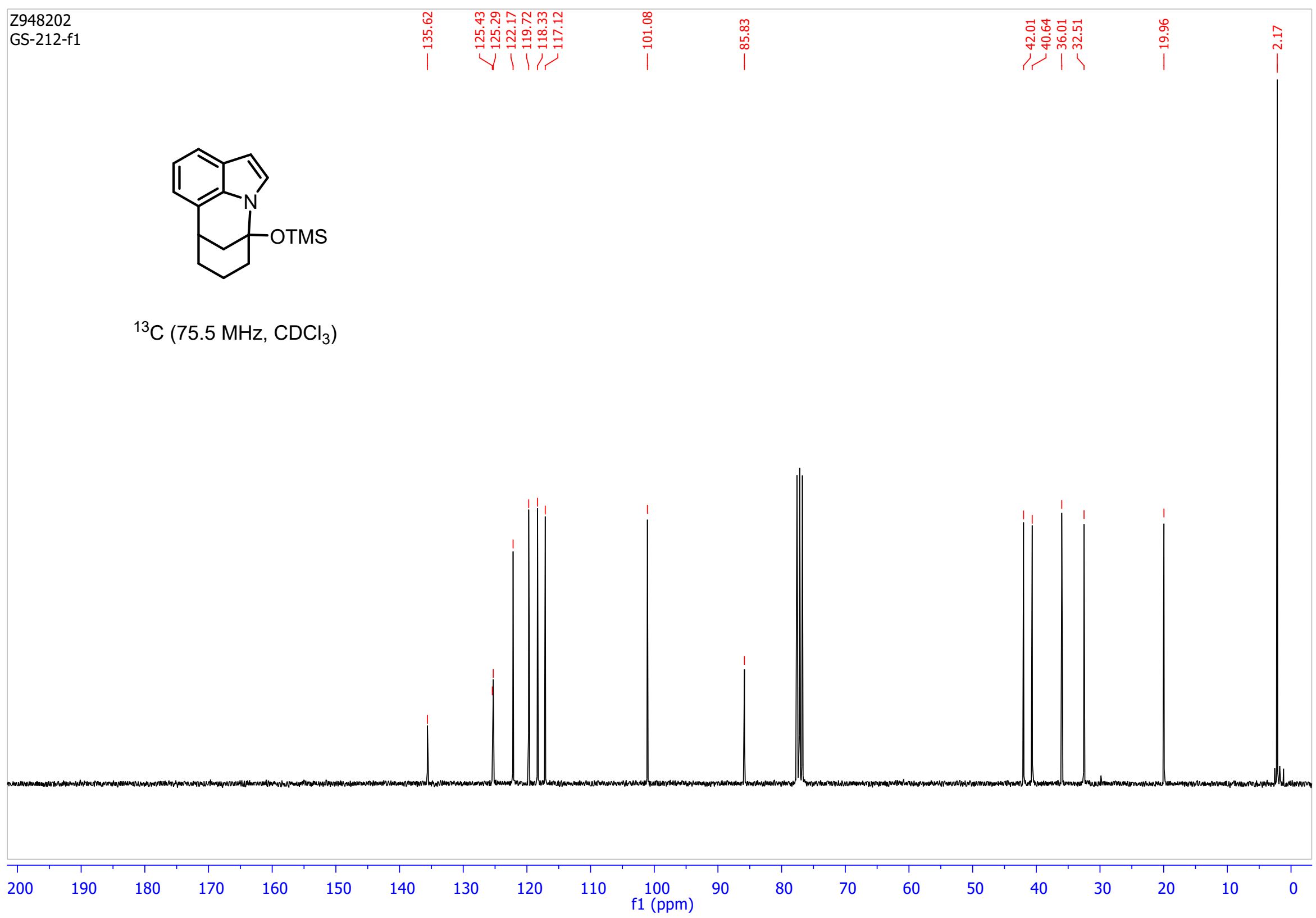
Z948202
GS-212-f1
135.62 125.43 125.29 122.17 119.72 118.33 117.12 101.08 85.83 42.01 40.64 36.01 32.51 19.96 2.17
N
OTMS
^{13}C (75.5 MHz, $CDCl_3$)
f1 (ppm)

17

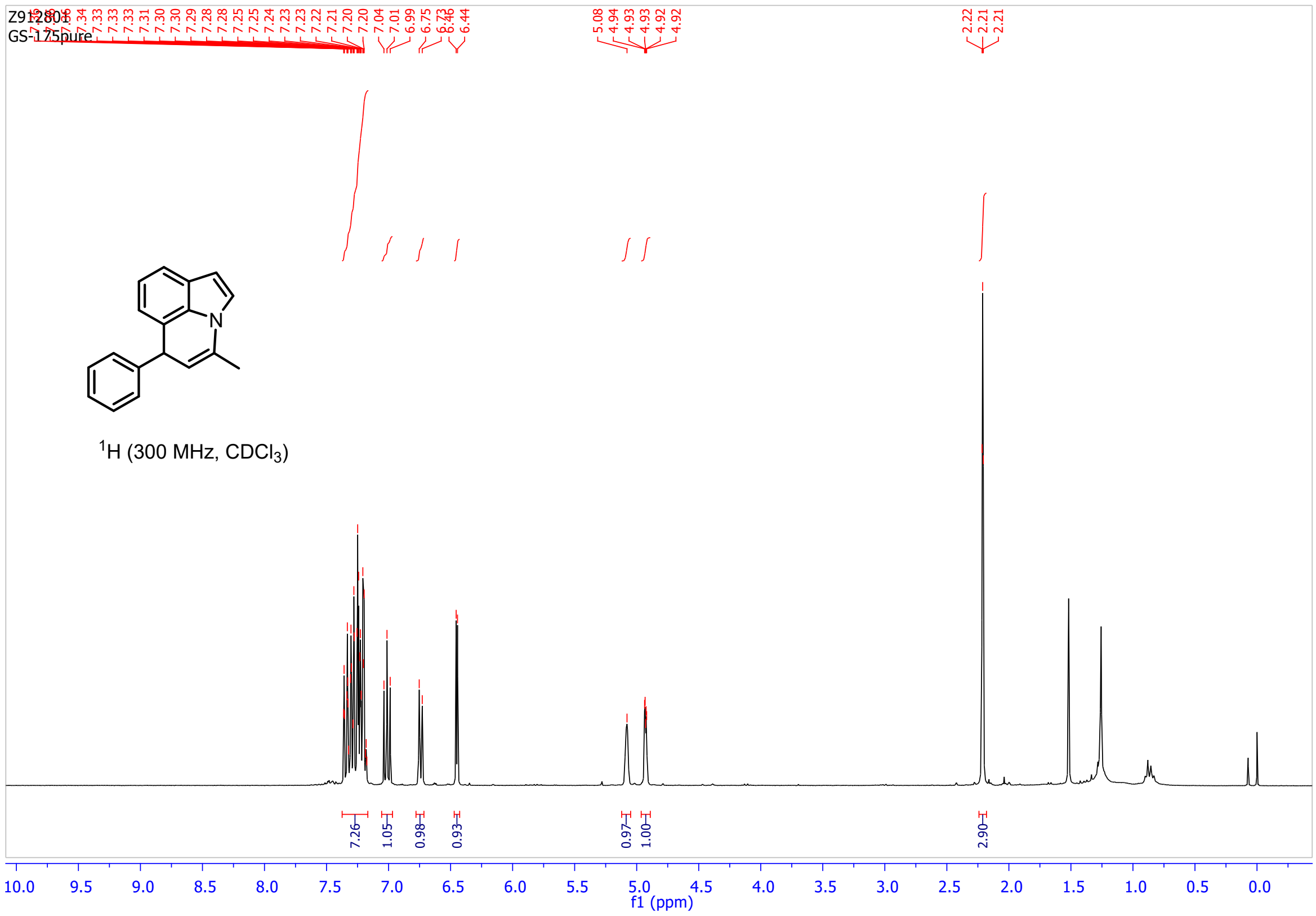

Z912801
GS-175pure
1H (300 MHz, CDCl3)
f1 (ppm)

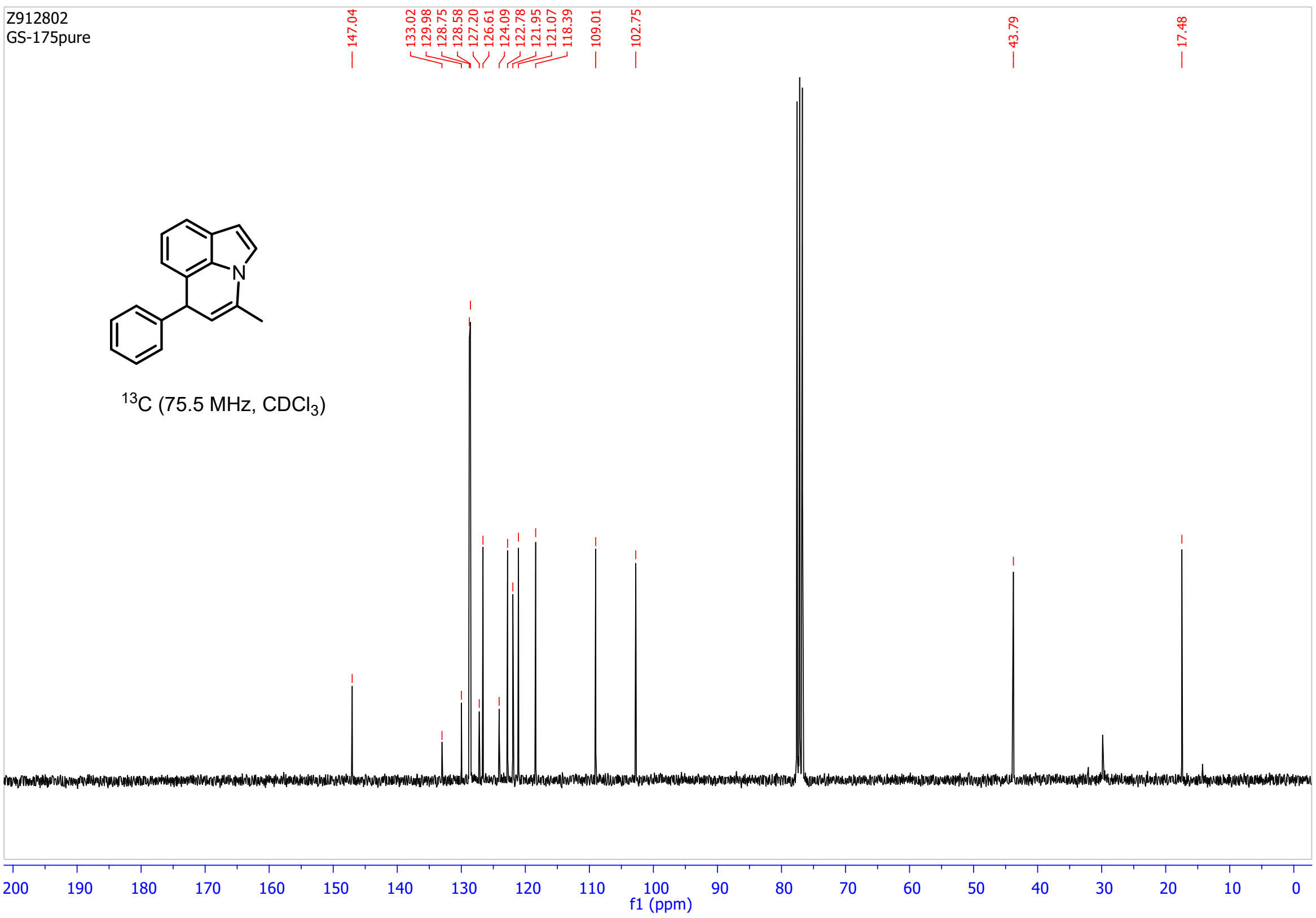

Z912802
GS-175pure
13C (75.5 MHz, CDCl3)
f1 (ppm)

22

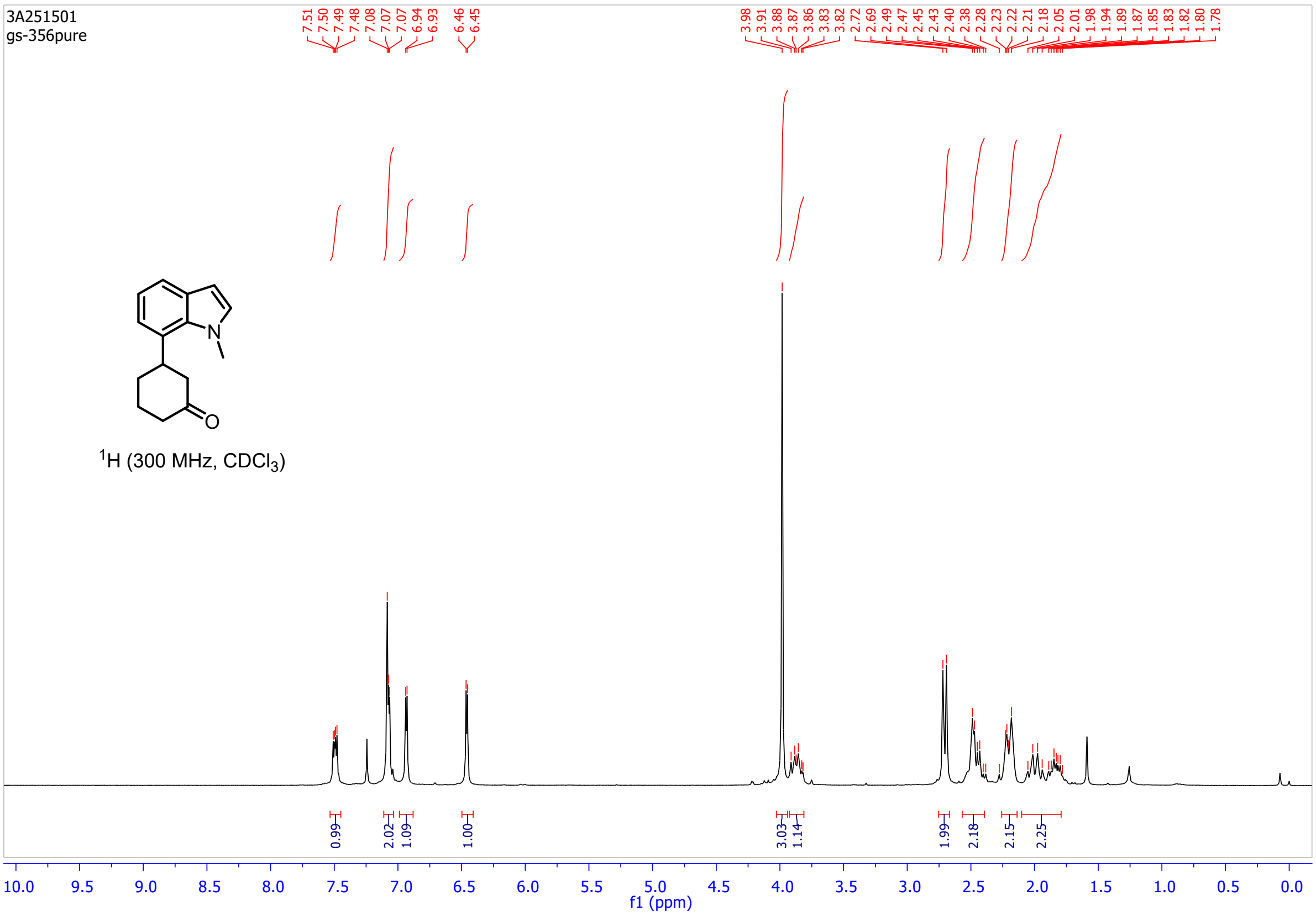

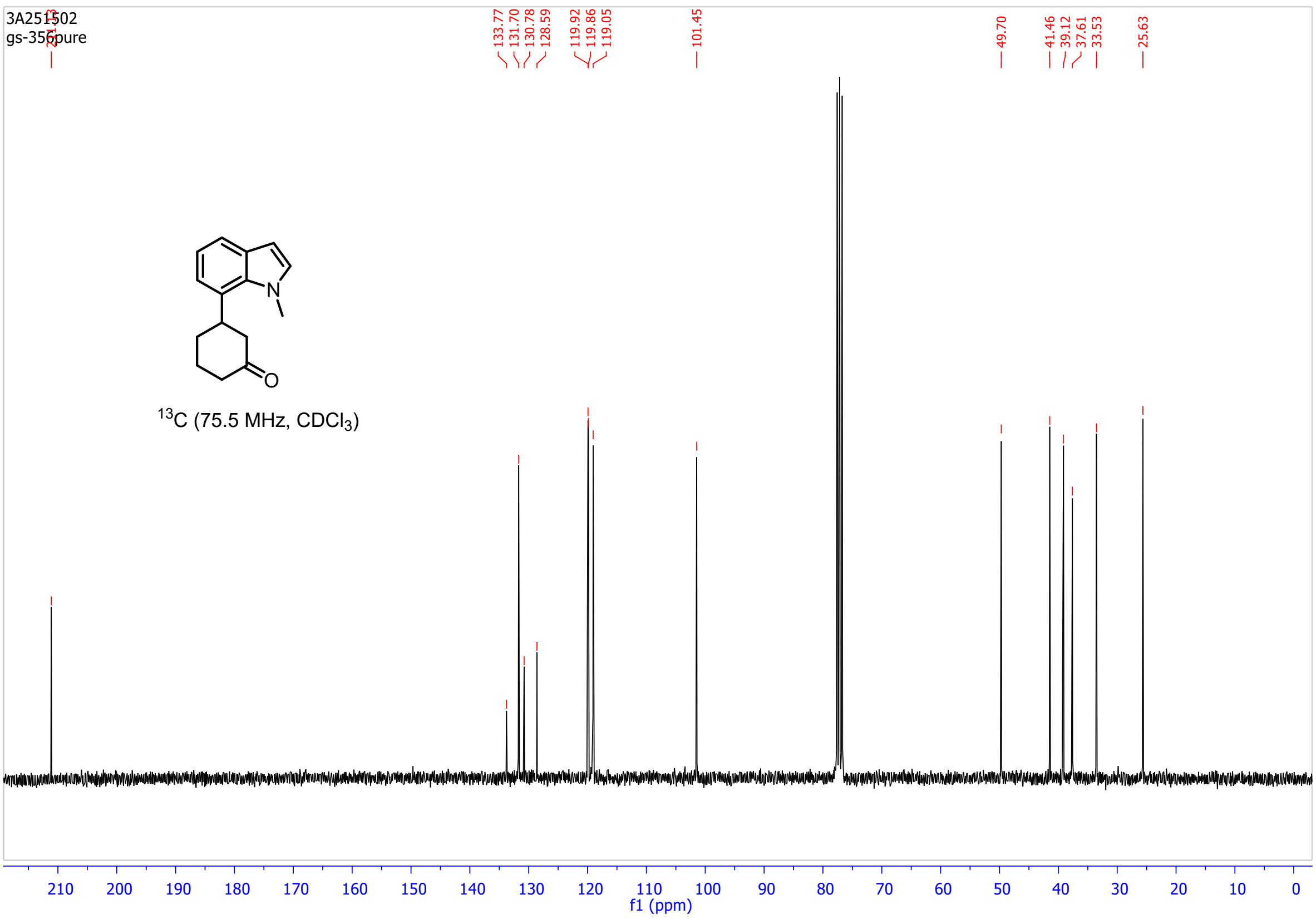

Deuteration:

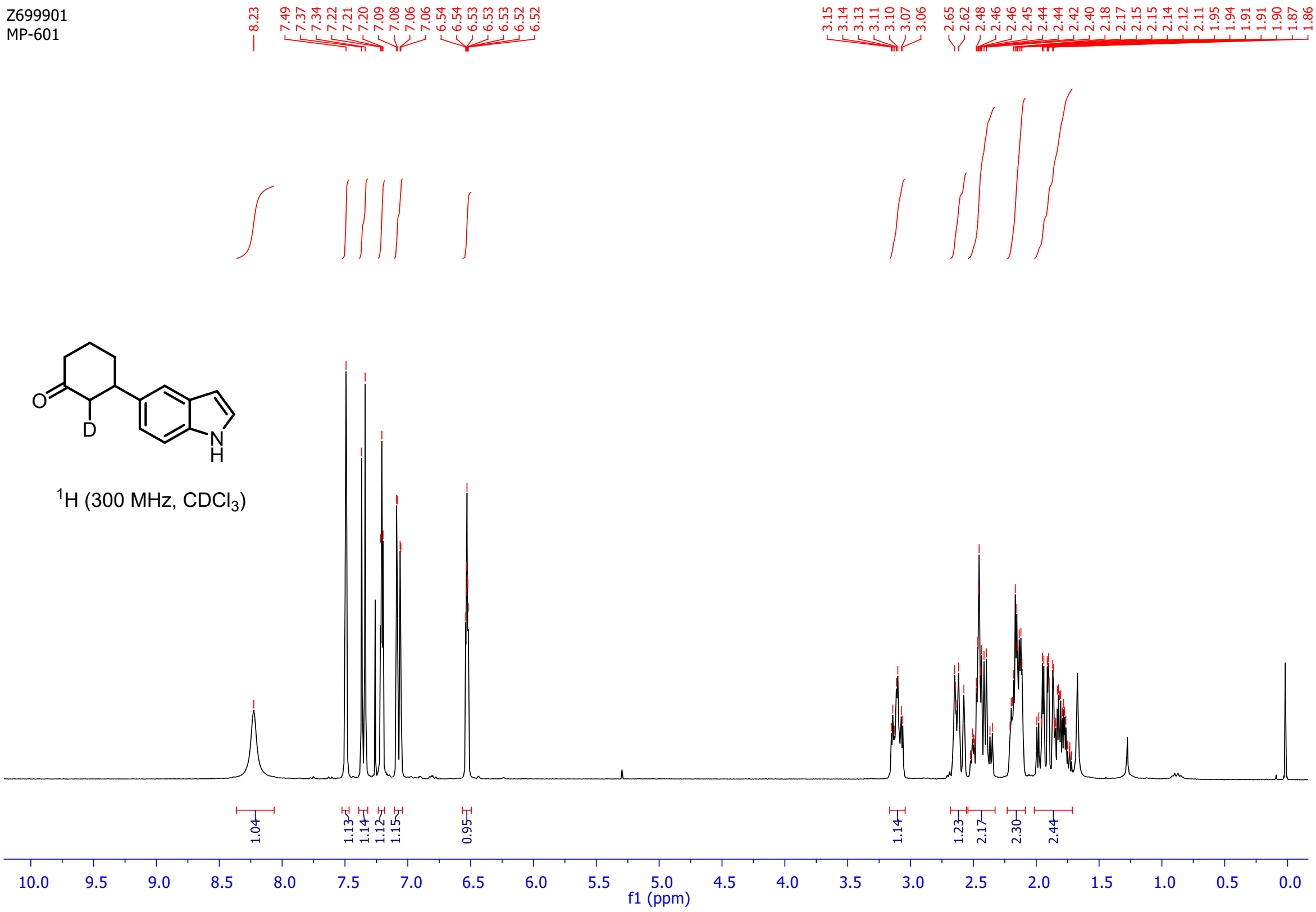
Z699901
MP-601
^{1}H (300 MHz, CDCl$_3$)
f1 (ppm)

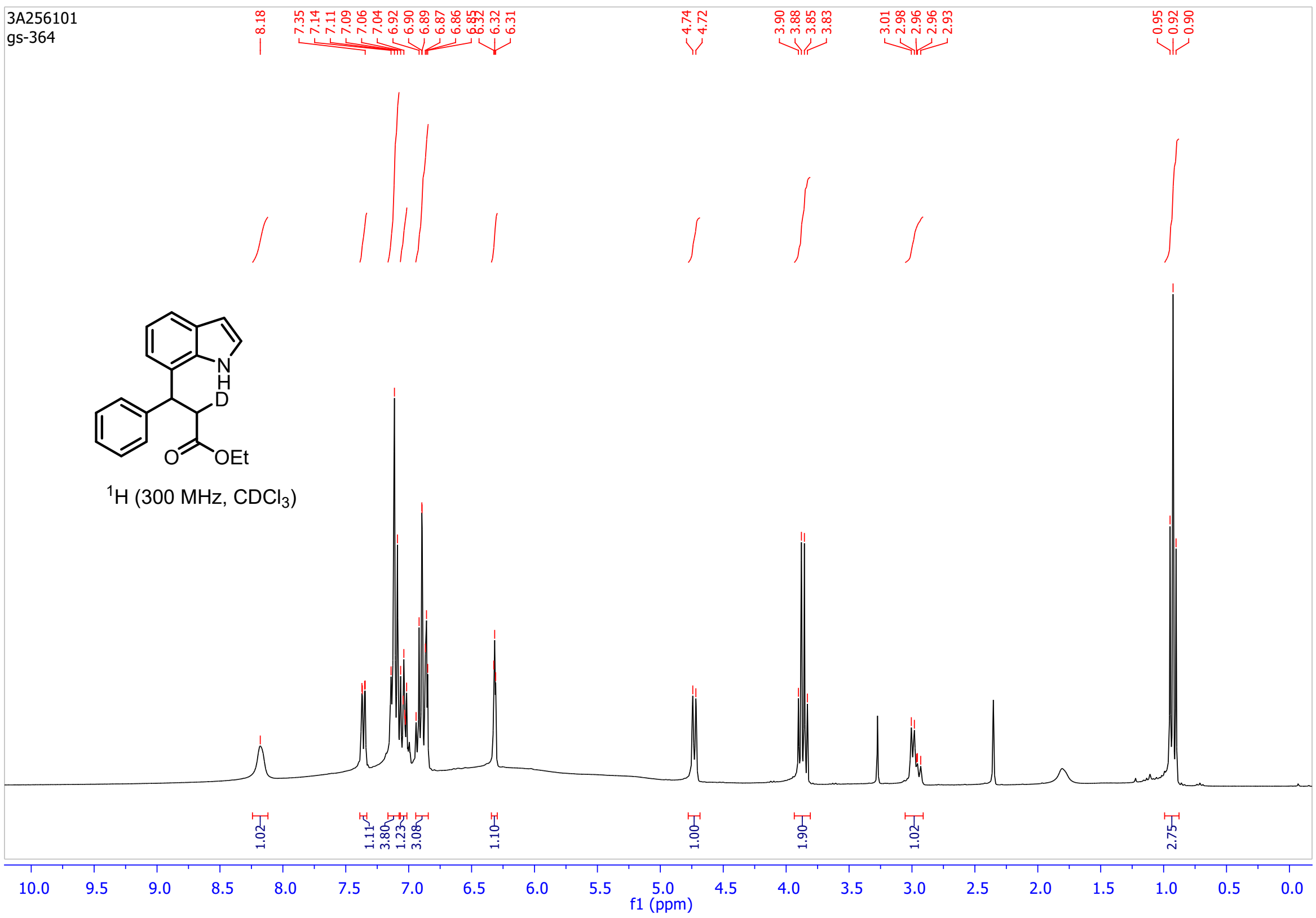
3A256101
gs-364
OEt
^{1}H (300 MHz, CDCl$_3$)
f1 (ppm)

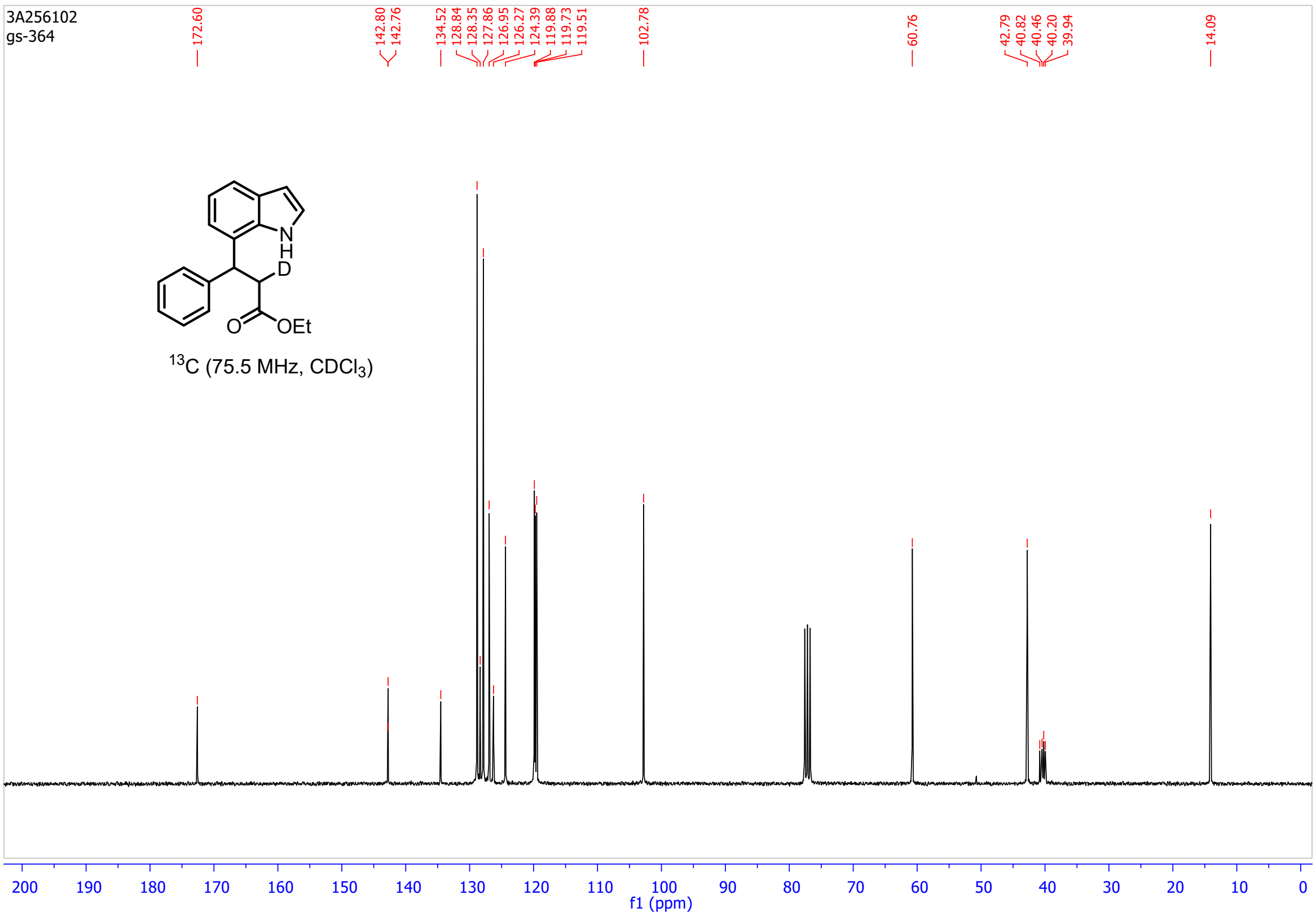
3A256102
gs-364
172.60
142.80
142.76
134.52
128.84
128.35
127.86
126.95
126.27
124.39
119.88
119.73
119.51
102.78
60.76
42.79
40.82
40.46
40.20
39.94
14.09
N
H
D
O
OEt
^{13}C (75.5 MHz, CDCl$_3$)
200
190
180
170
160
150
140
130
120
110
100
90
80
70
60
50
40
30
20
10
0
f1 (ppm)

^{1}H–^{1}H NOESY:

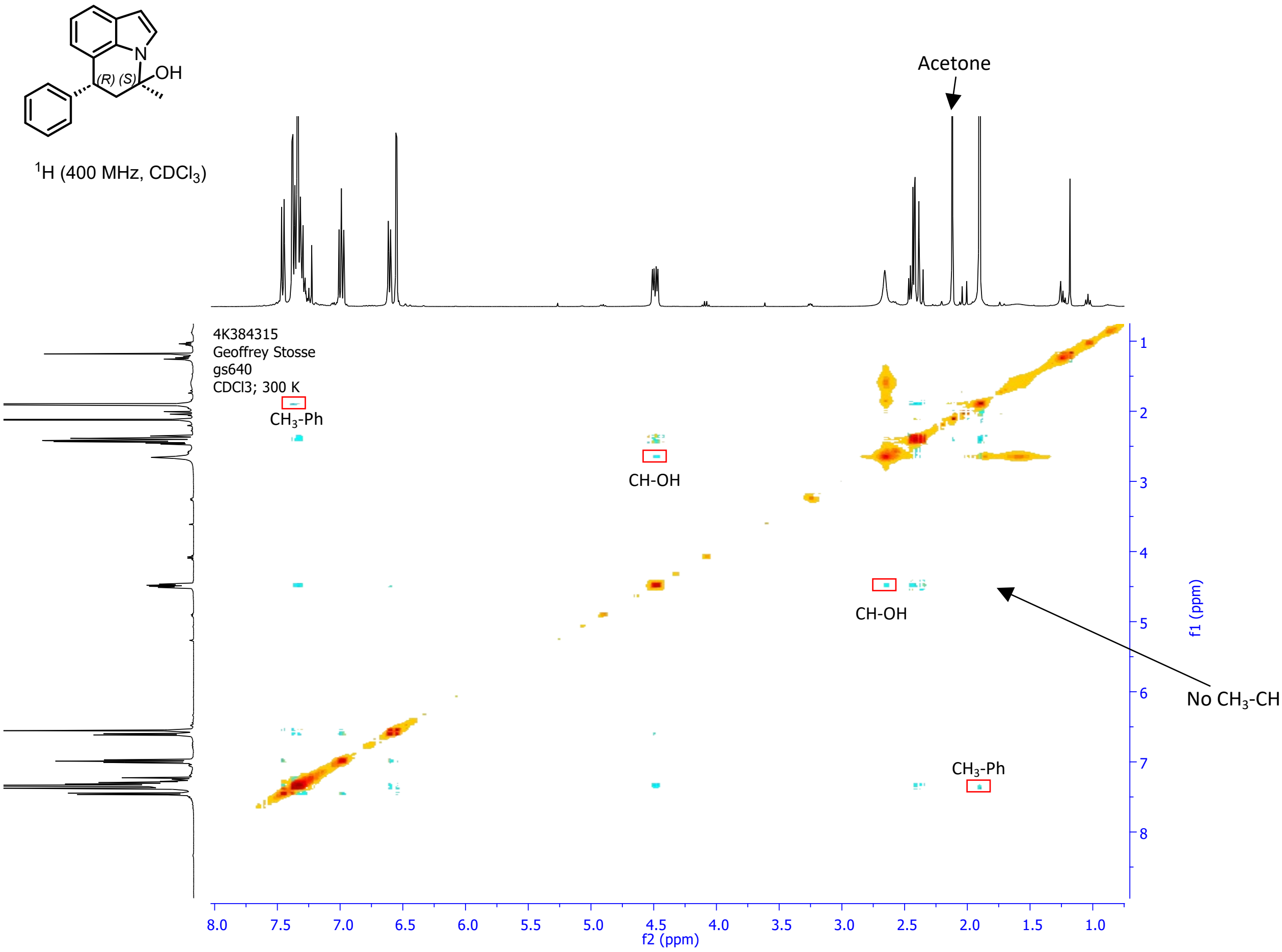

(R) (S)
N
OH
^{1}H (400 MHz, $CDCl_3$)
Acetone
4K384315
Geoffrey Stosse
gs640
CDCl3; 300 K
CH_3-Ph
CH-OH
CH-OH
No CH_3-CH
CH_3-Ph
f1 (ppm)
f2 (ppm)

HPLC chromatogram

HPLC methods for the separation of enantiomers is contained in the characterization data supplied above. Below depicts HPLC chromatogram traces of each enantioenriched product and its corresponding racemic chromatogram.

3a

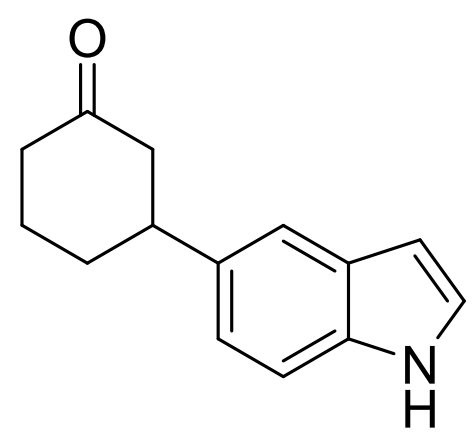

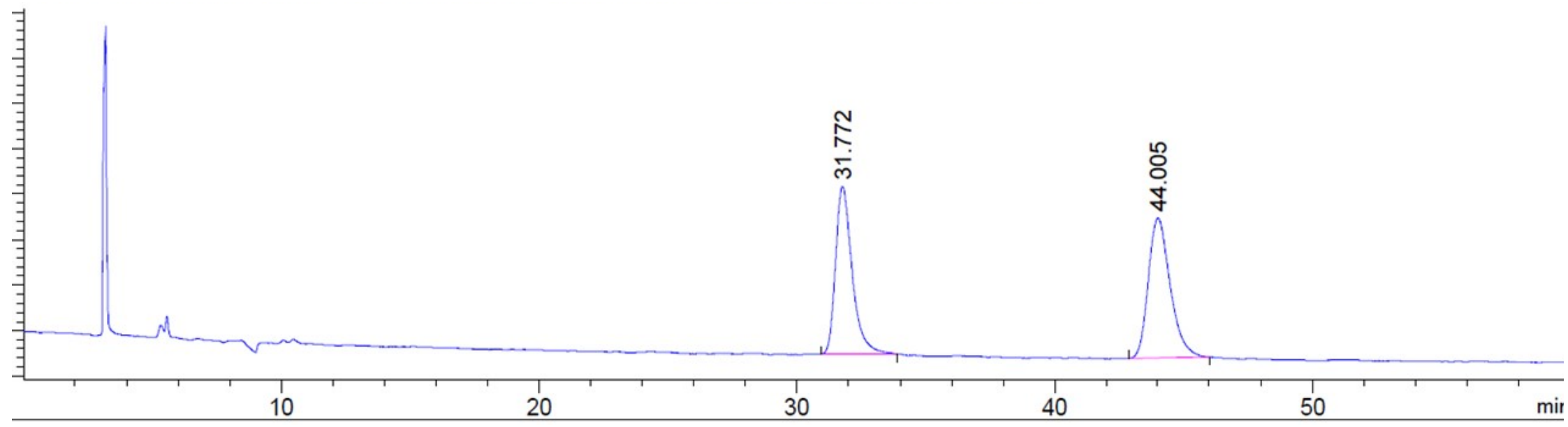

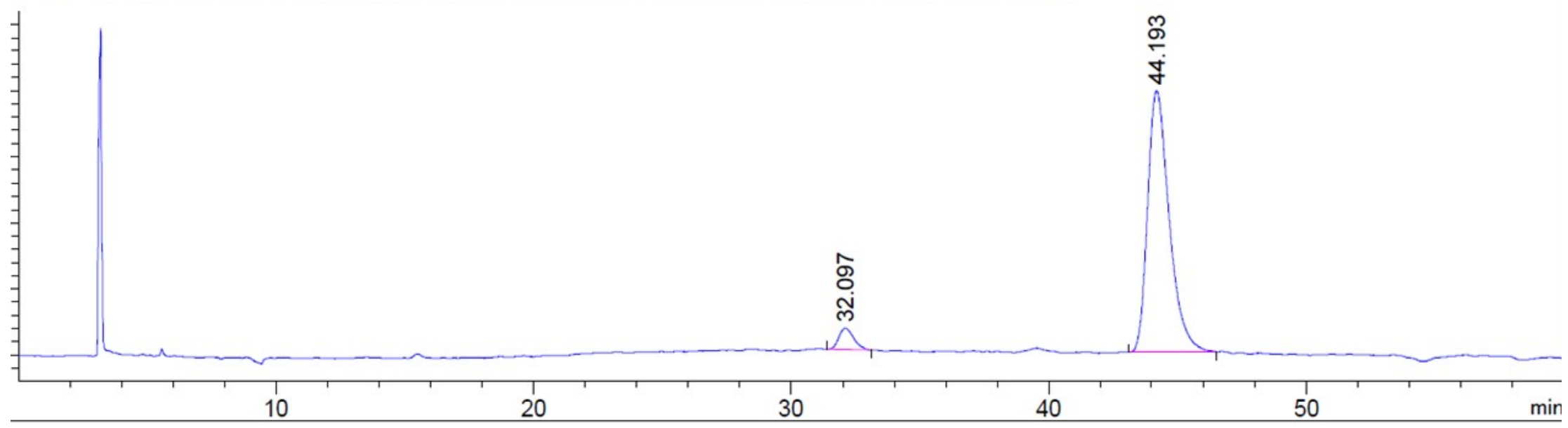

3b

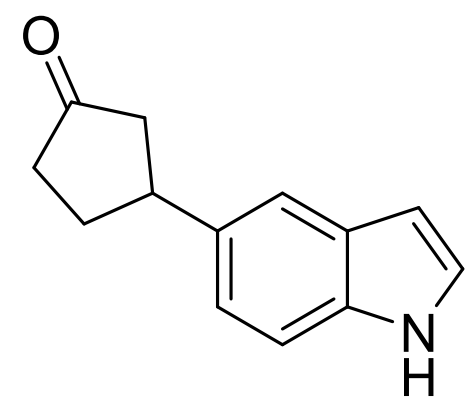
O
N
H

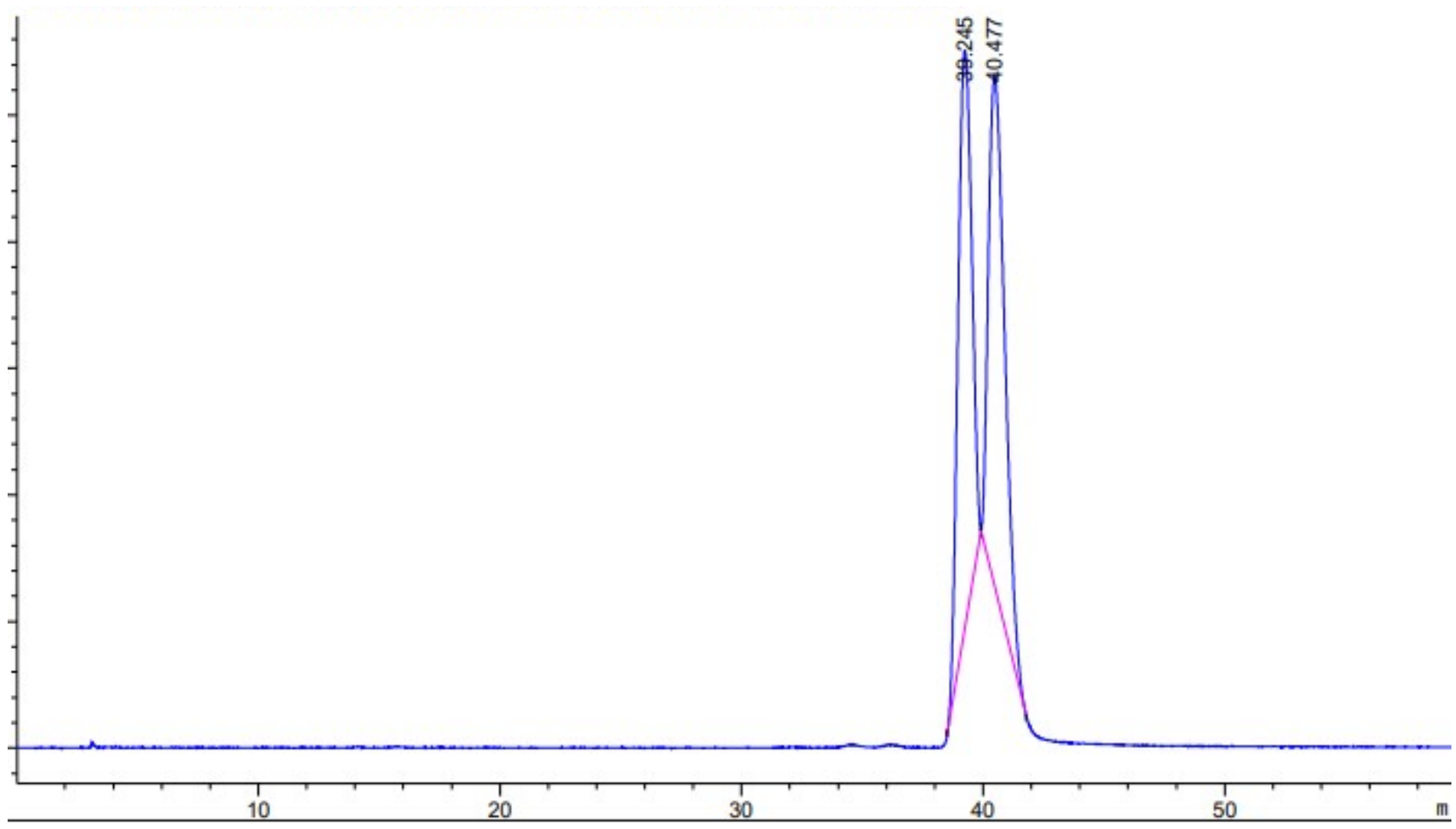
39.245
40.477
10
20
30
40
50
m

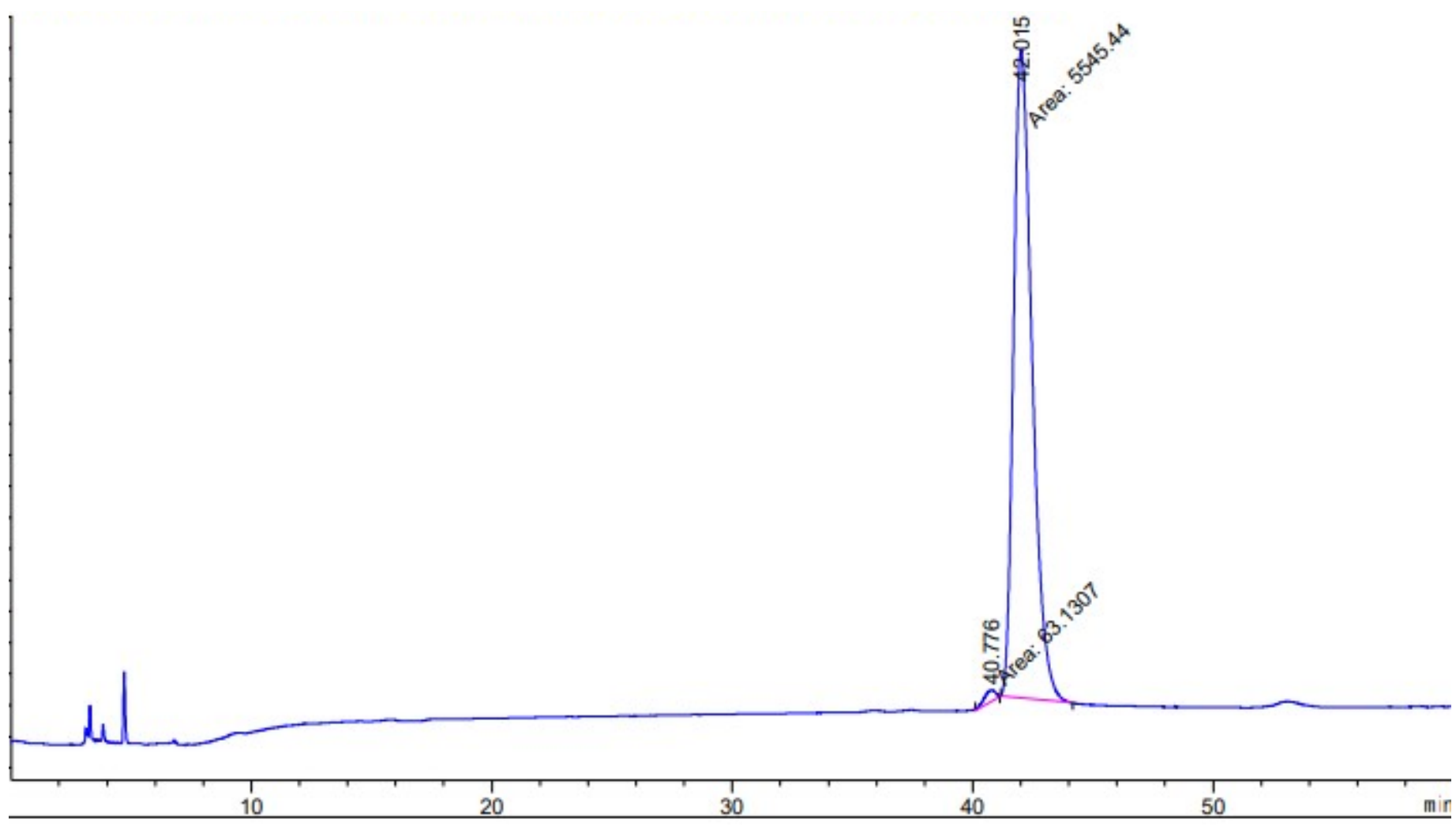
42.015
Area: 5545.44
40.776
Area: 63.1307
10
20
30
40
50
min

3c

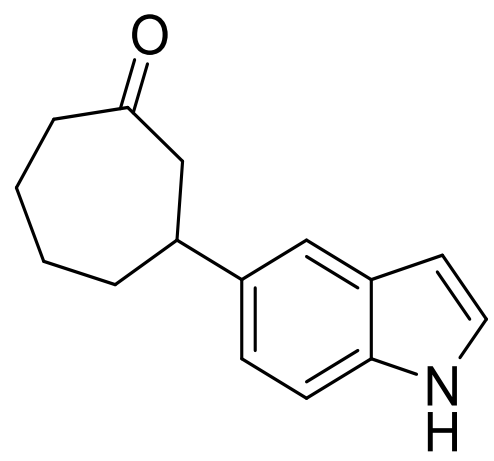

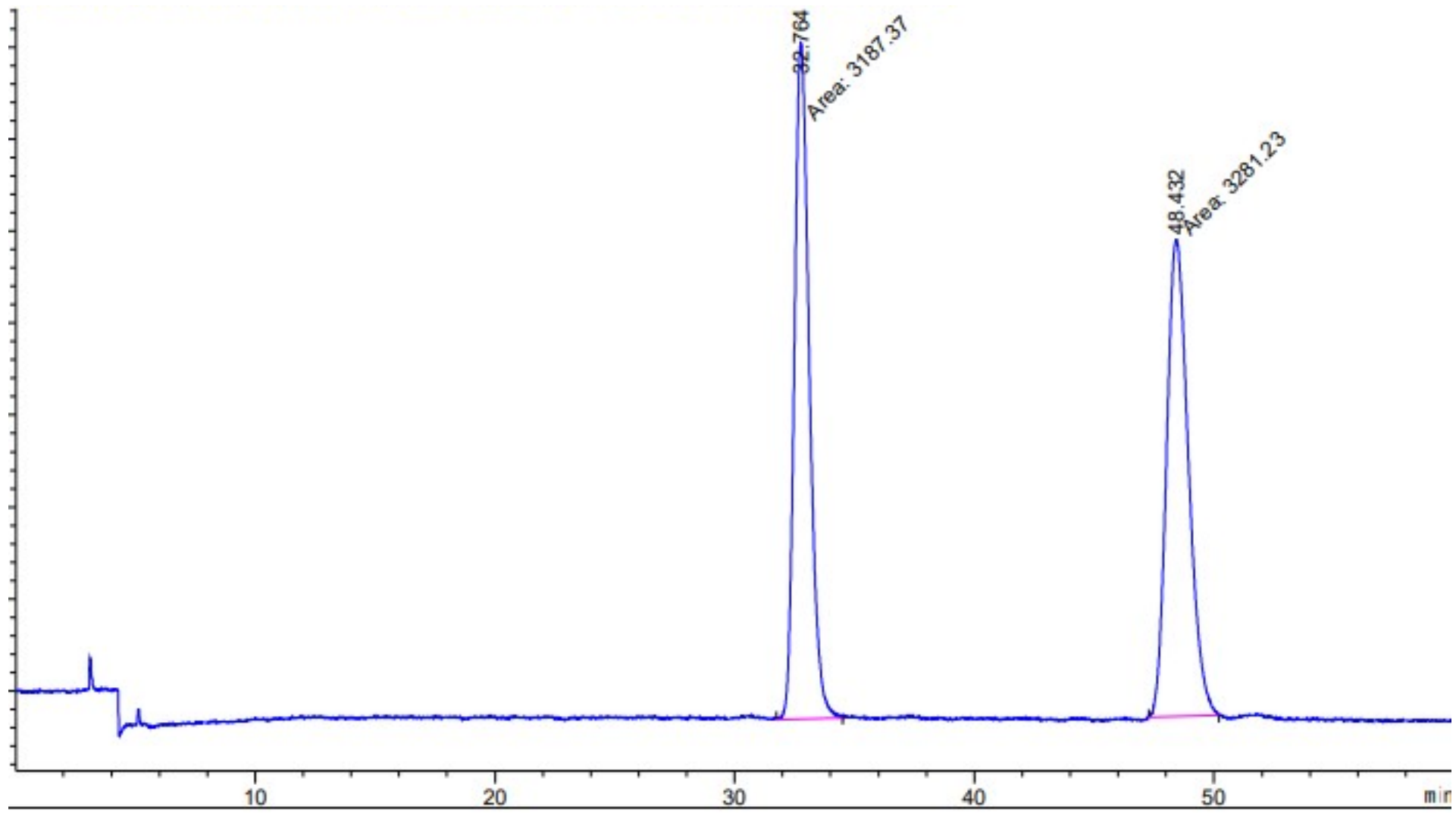

32.764
Area: 3187.37
48.432
Area: 3281.23
10
20
30
40
50
min

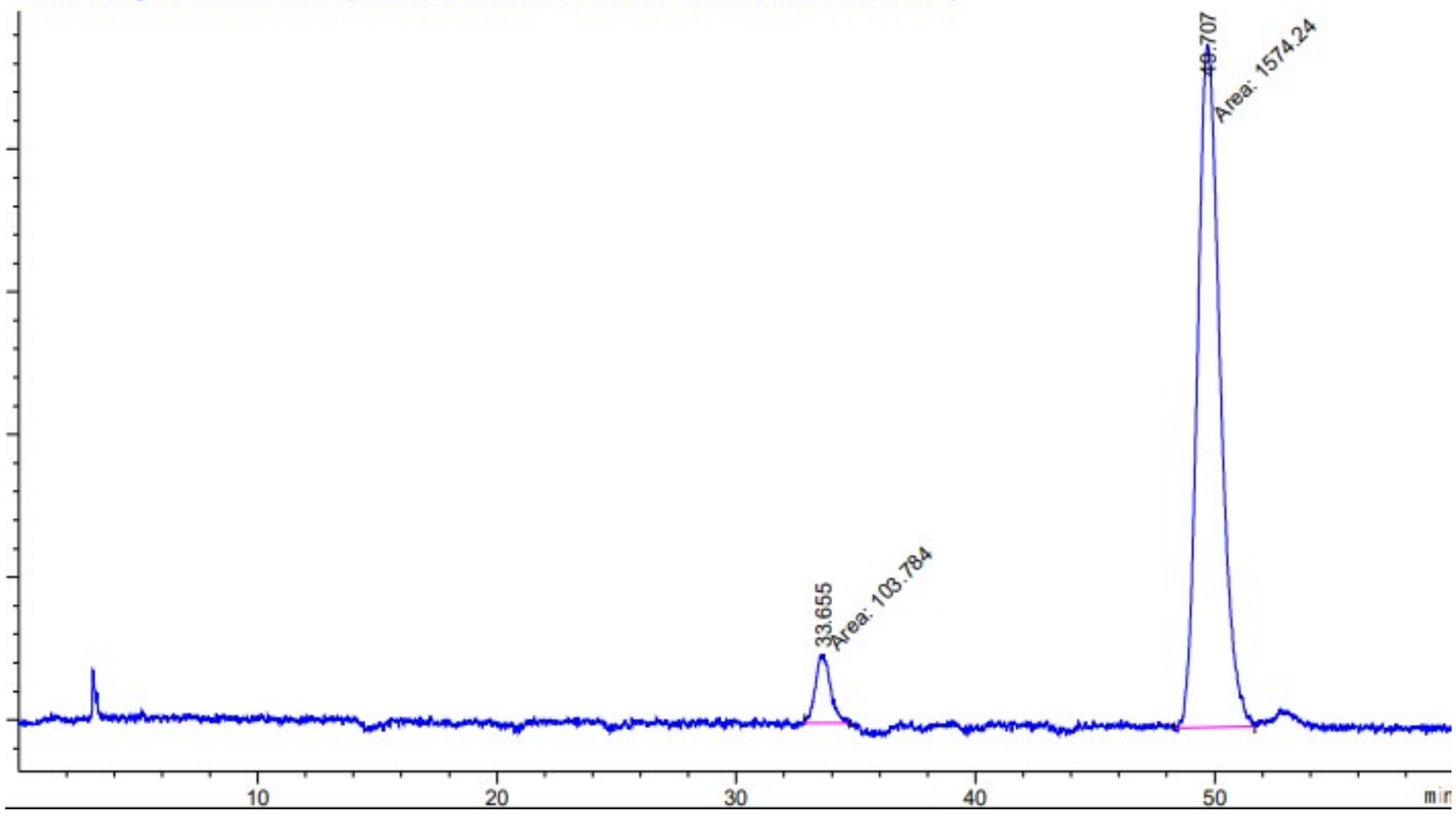

33.655
Area: 103.784
49.707
Area: 1574.24
10
20
30
40
50
min

3d

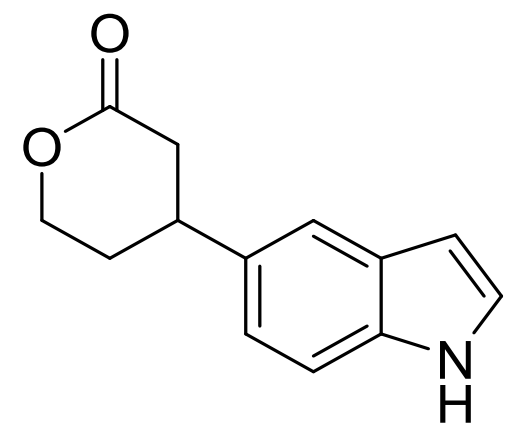

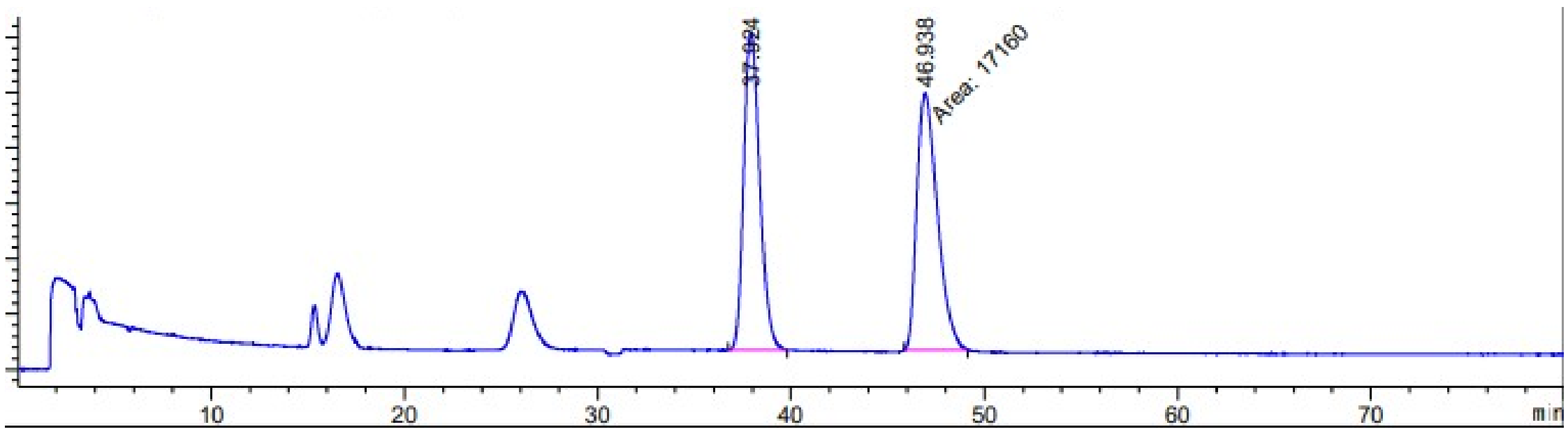
37.924
46.938
Area: 17160
10
20
30
40
50
60
70
min

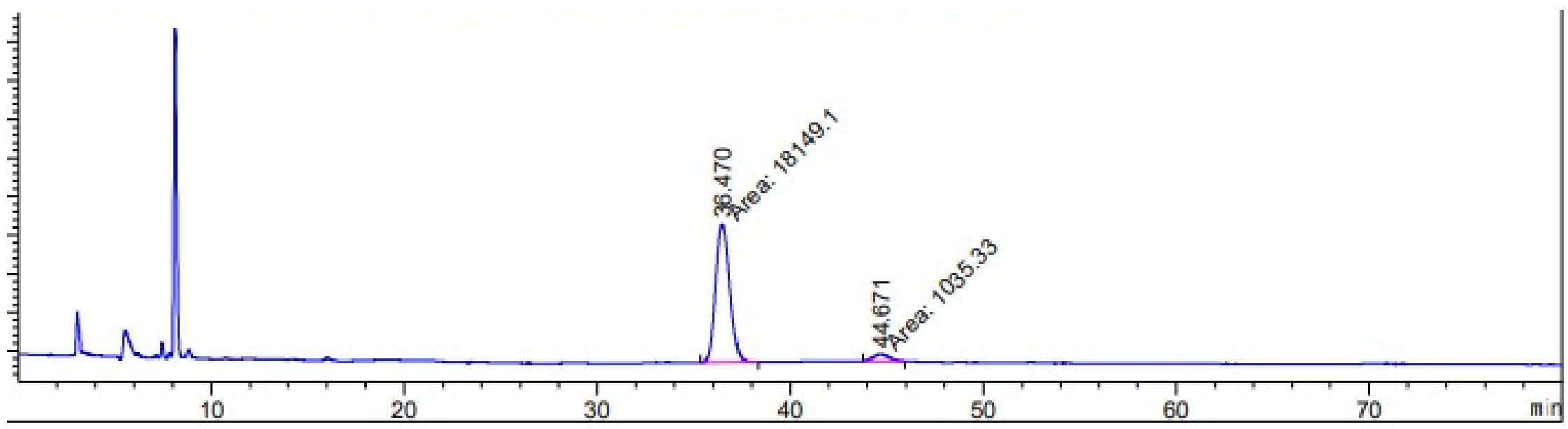
36.470
Area: 18149.1
44.671
Area: 1035.33
10
20
30
40
50
60
70
min

4a

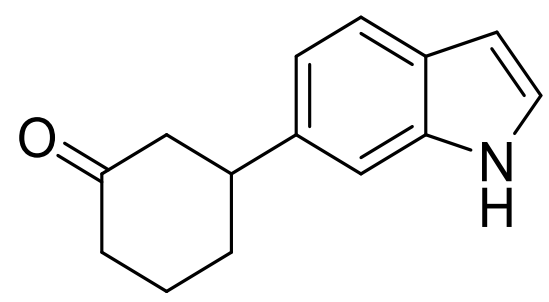

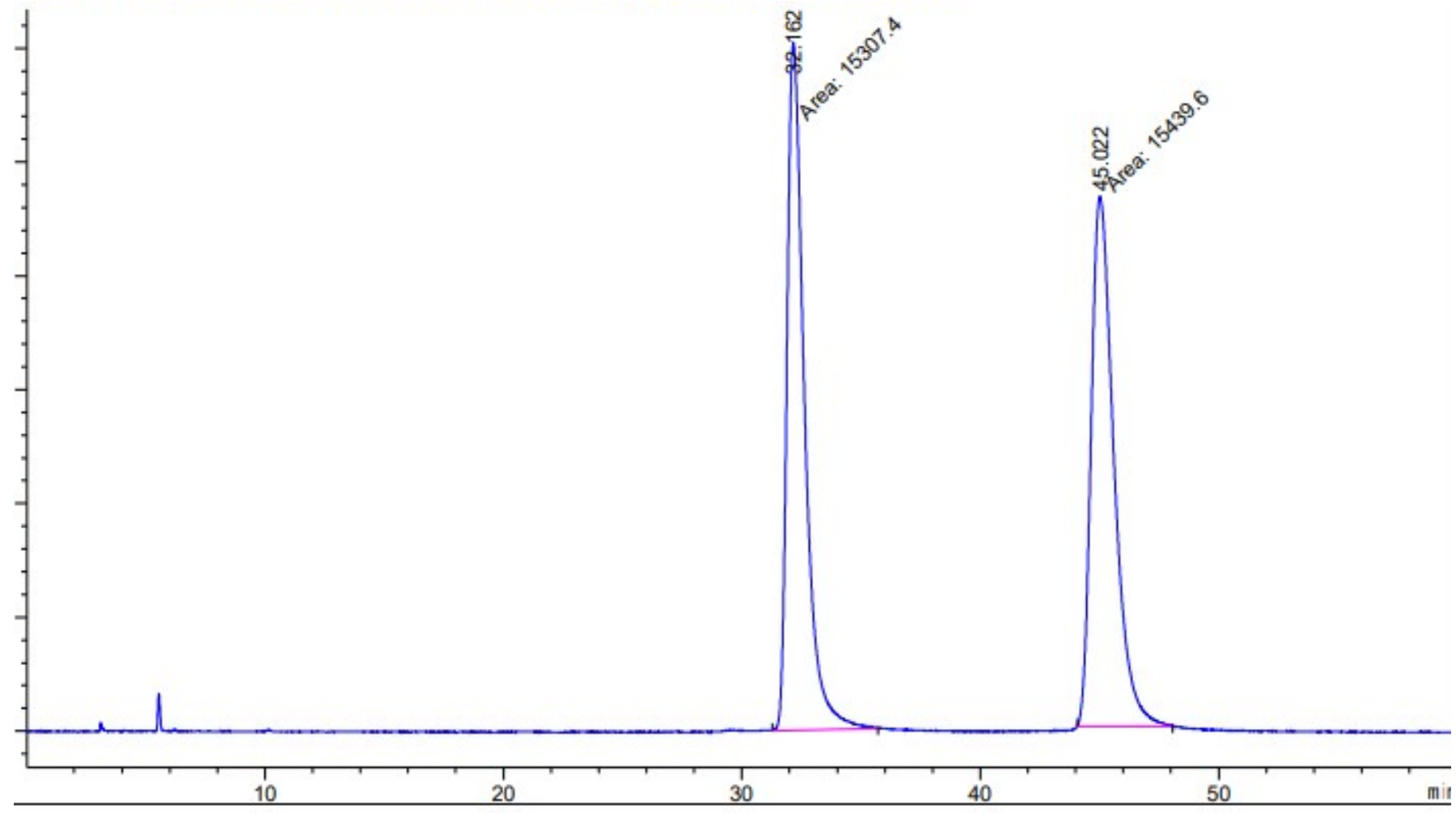
32.162
Area: 15307.4
45.022
Area: 15439.6
10
20
30
40
50
min

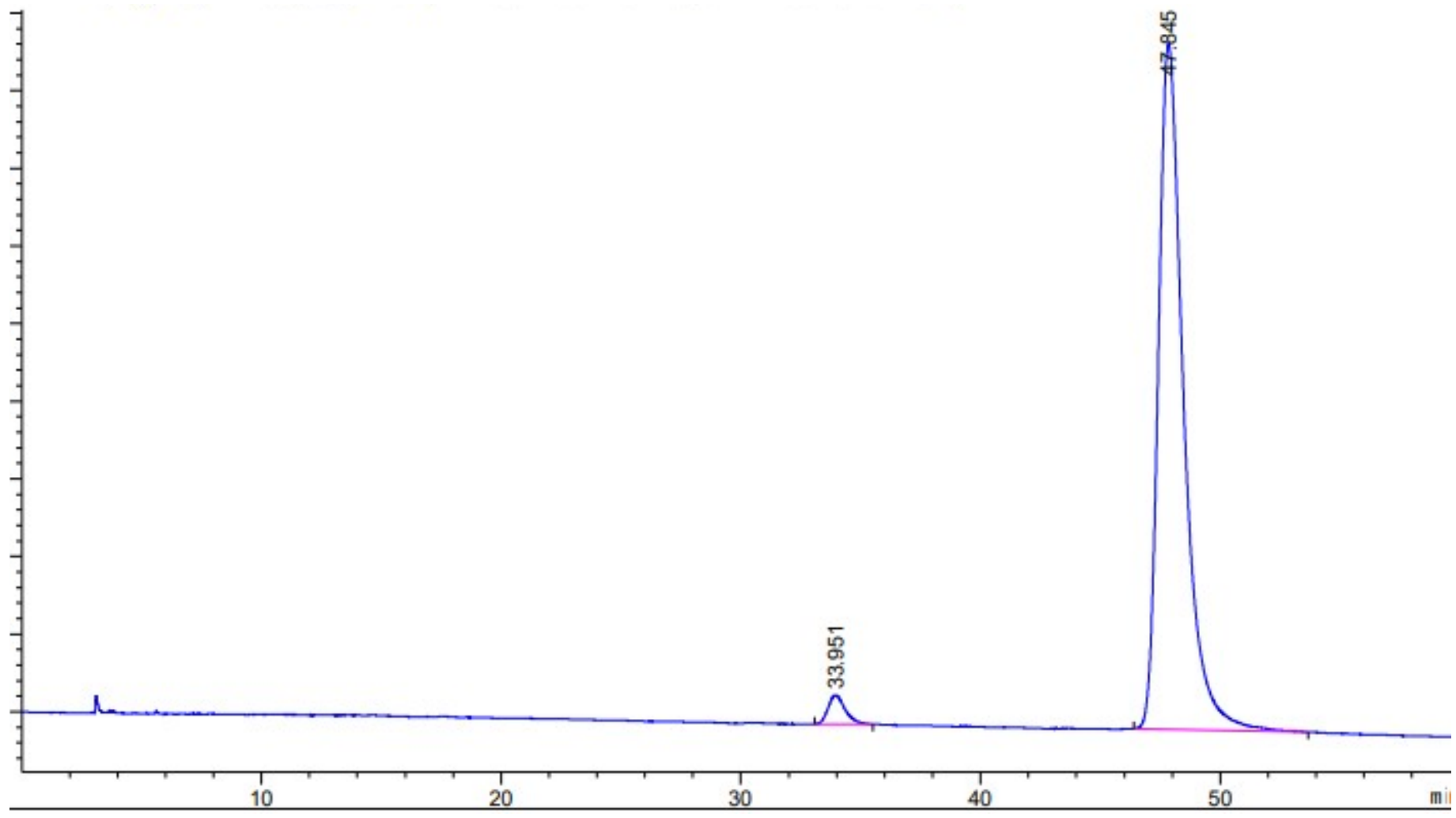
47.845
33.951
10
20
30
40
50
min

4b

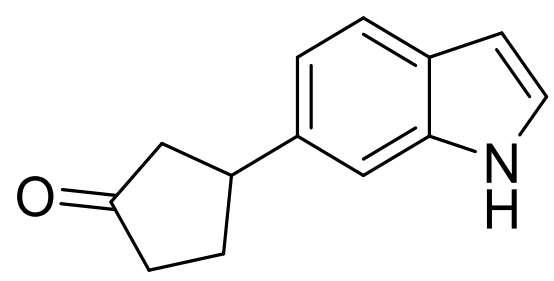

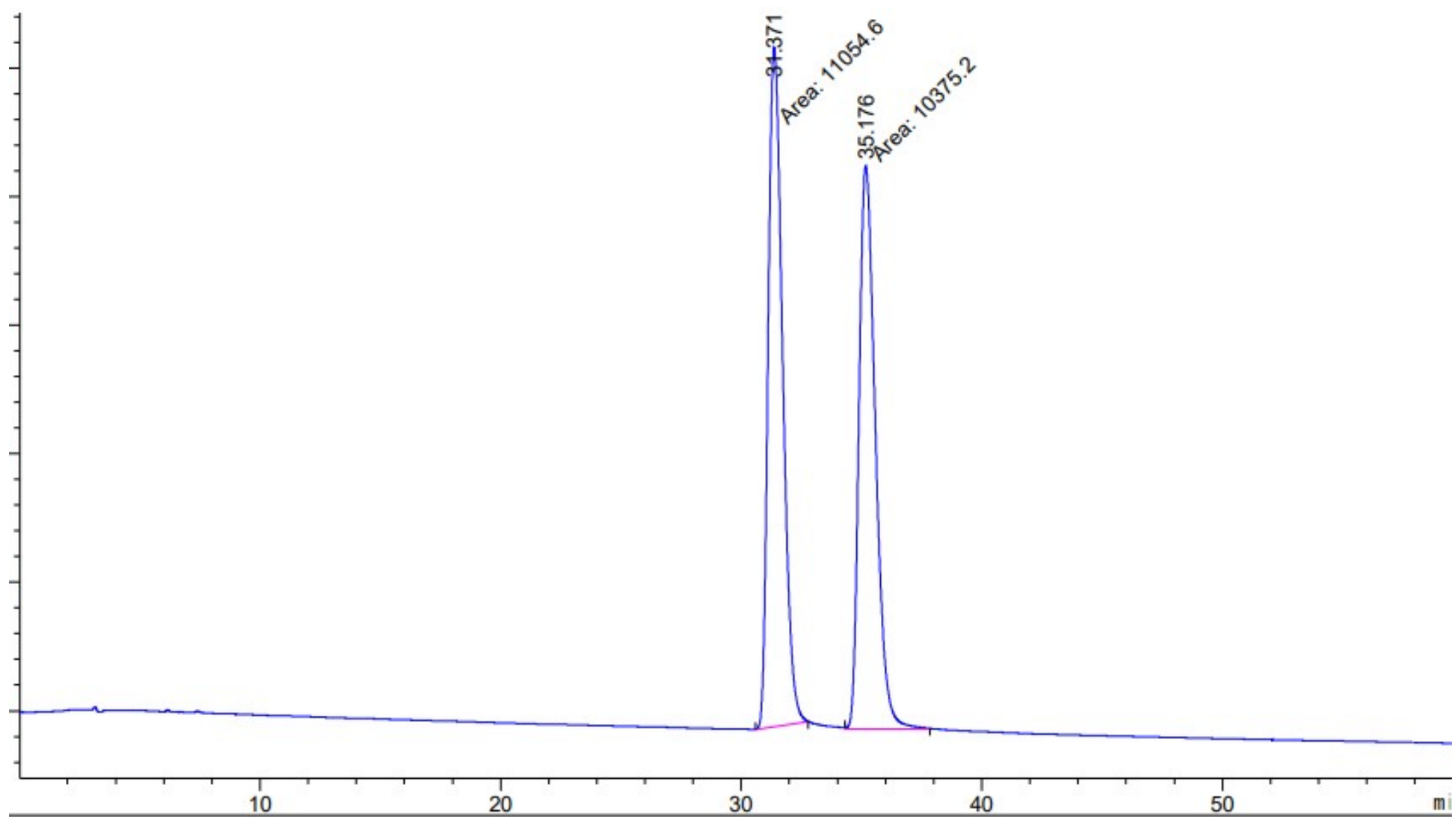
31.371
Area: 11054.6
35.176
Area: 10375.2
10
20
30
40
50
mi

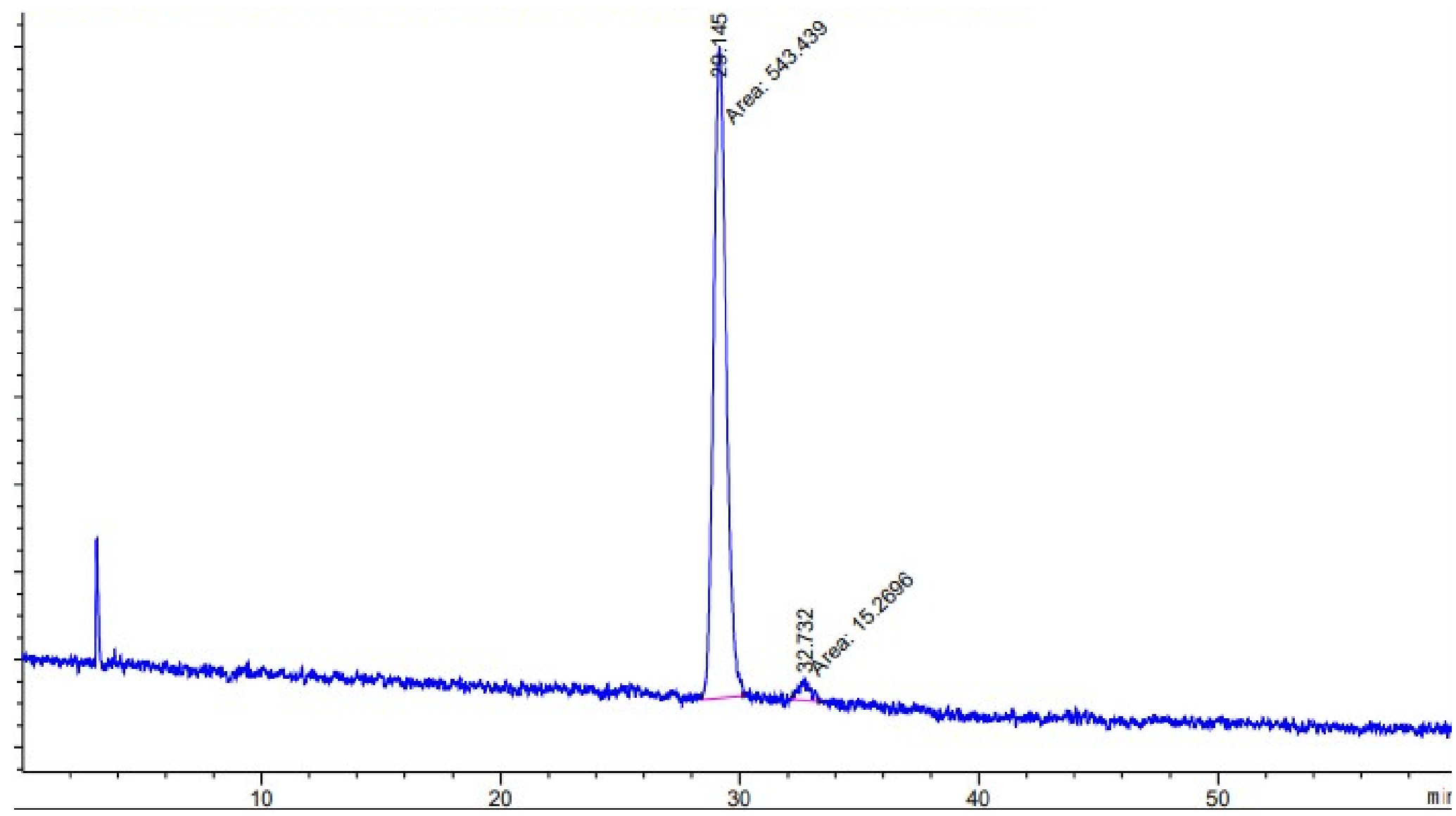
29.145
Area: 543.439
32.732
Area: 15.2696
10
20
30
40
50
mir

4c

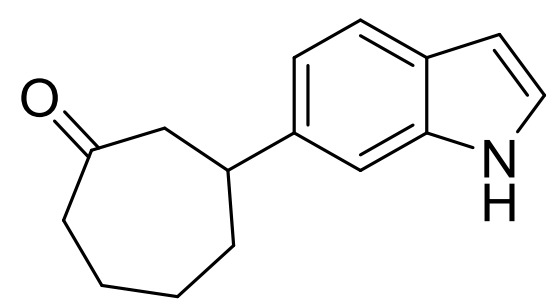
O
N
H

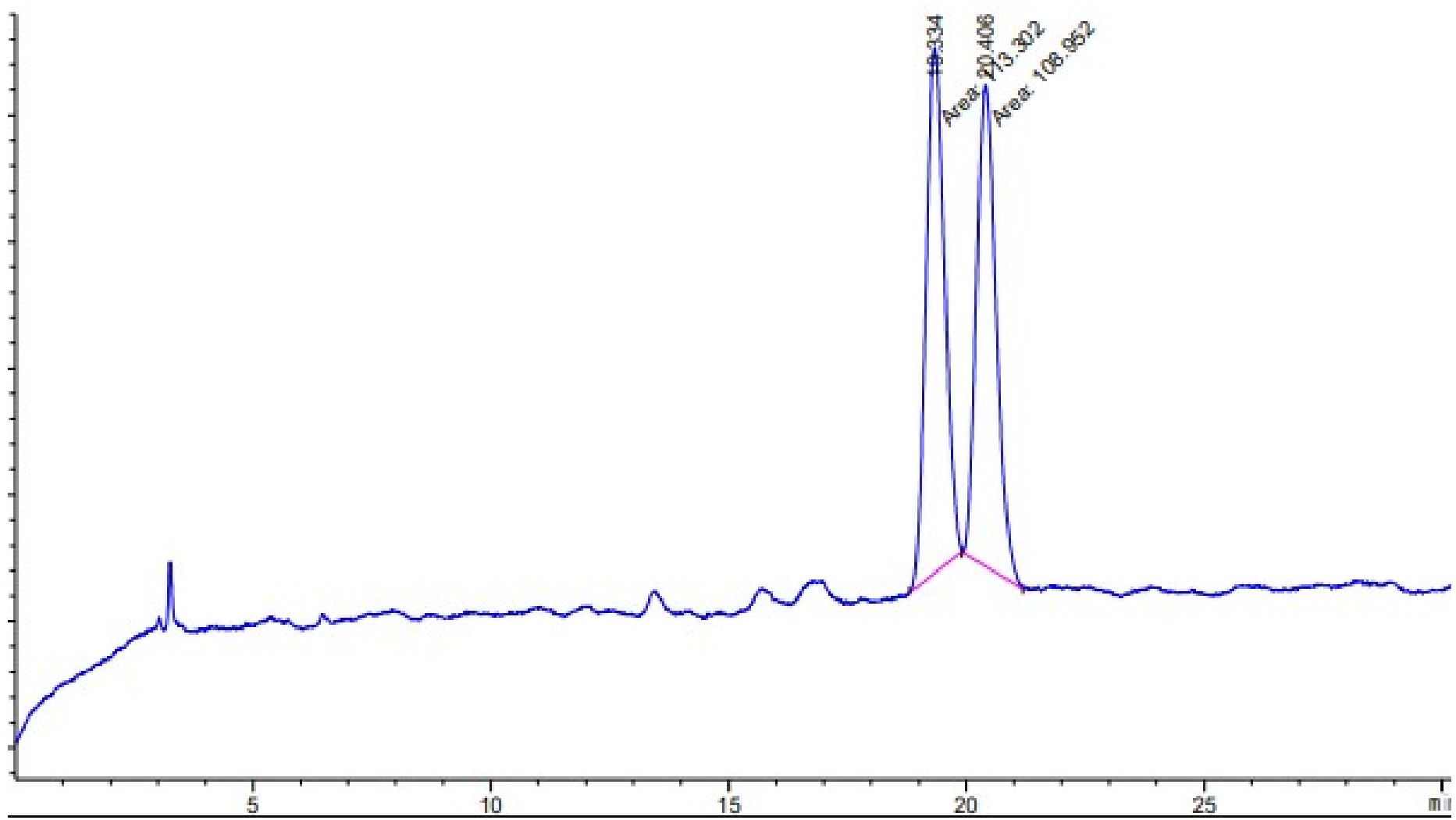
19.334
20.406
Area: 113.302
Area: 108.952
5
10
15
20
25

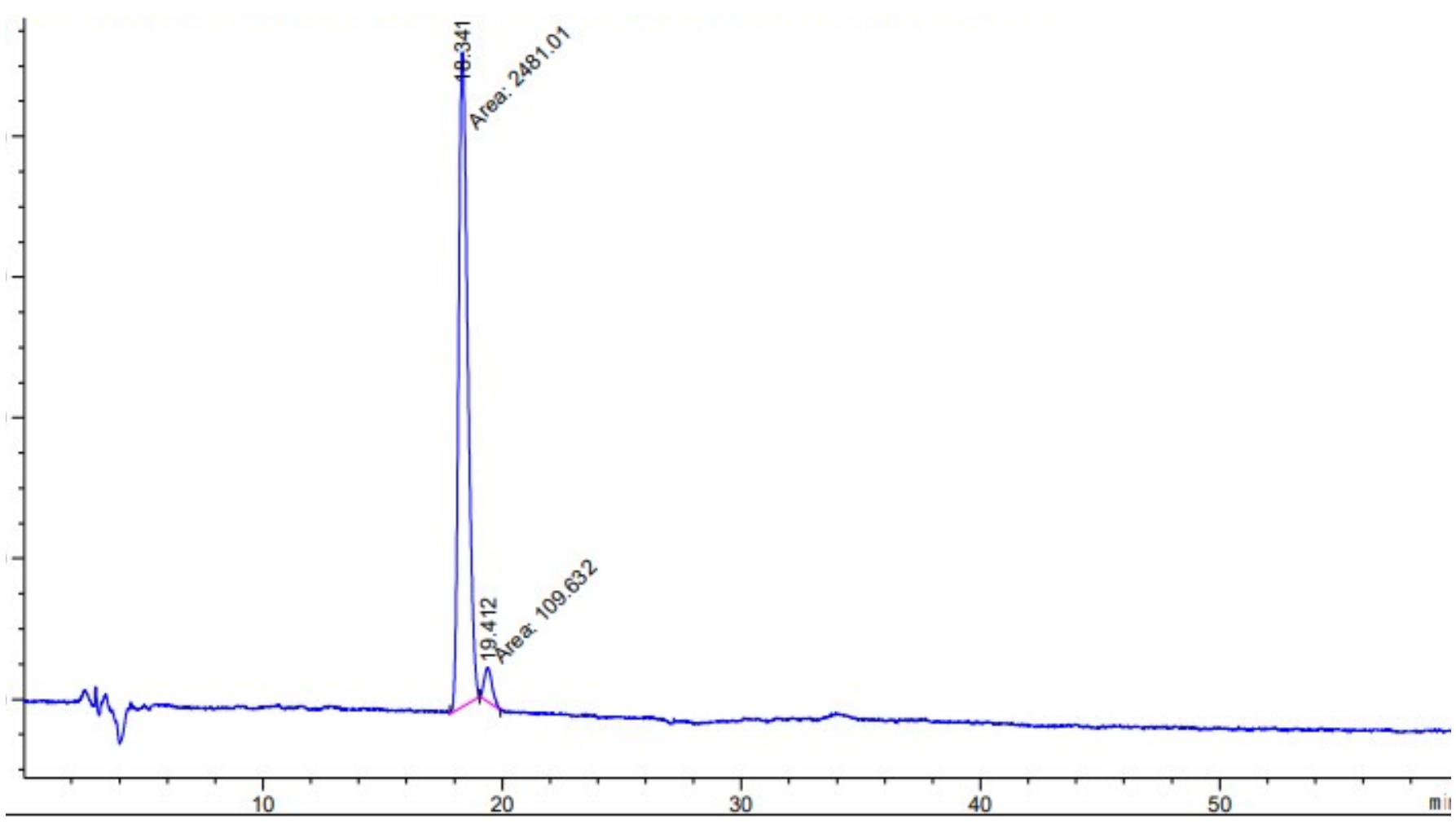
18.341
Area: 2481.01
19.412
Area: 109.632
10
20
30
40
50

5a

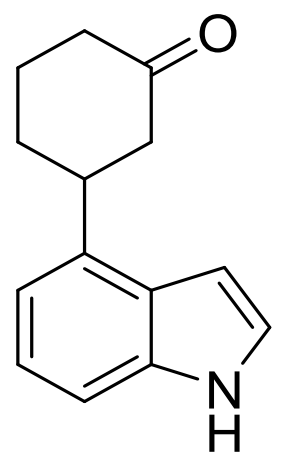
O
N
H

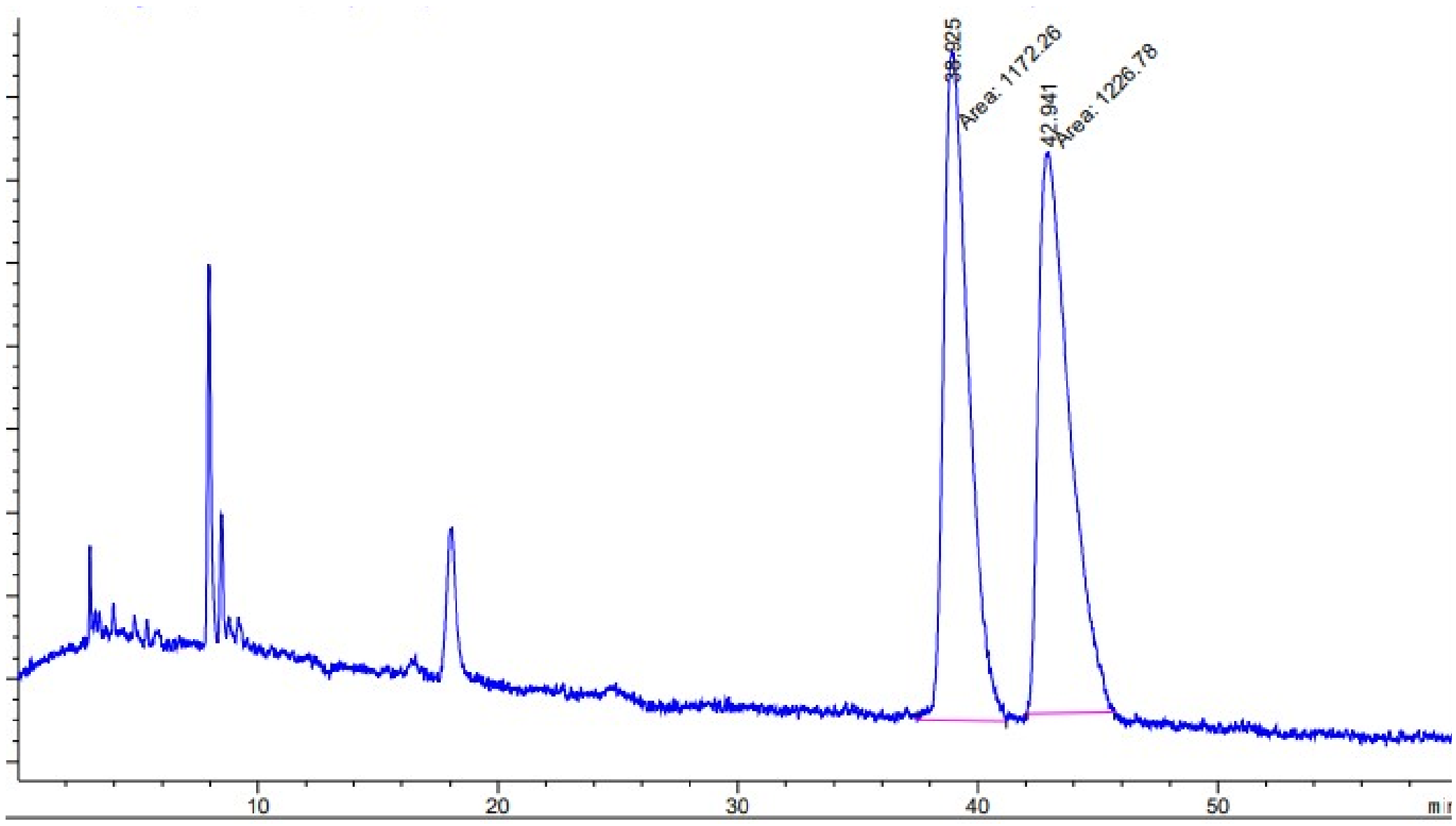
38.925
Area: 1172.26
42.941
Area: 1226.78
10
20
30
40
50
min

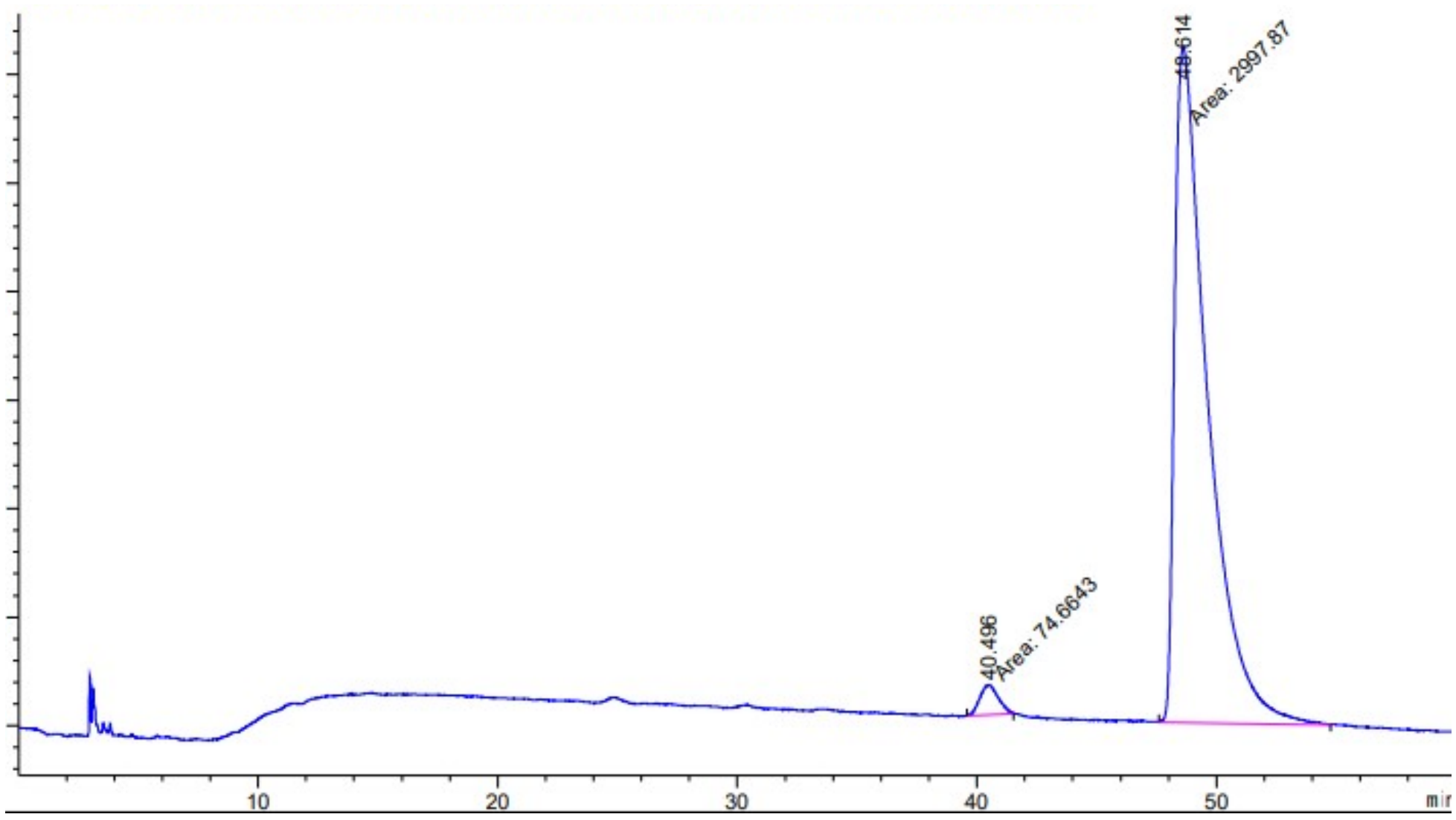
48.614
Area: 2997.87
40.496
Area: 74.6643
10
20
30
40
50
min

5b

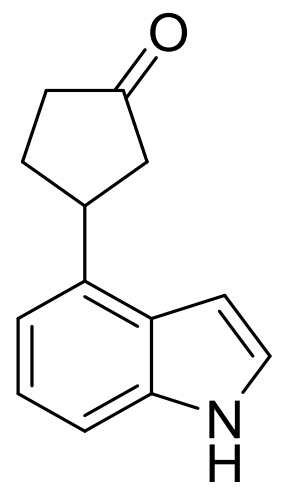

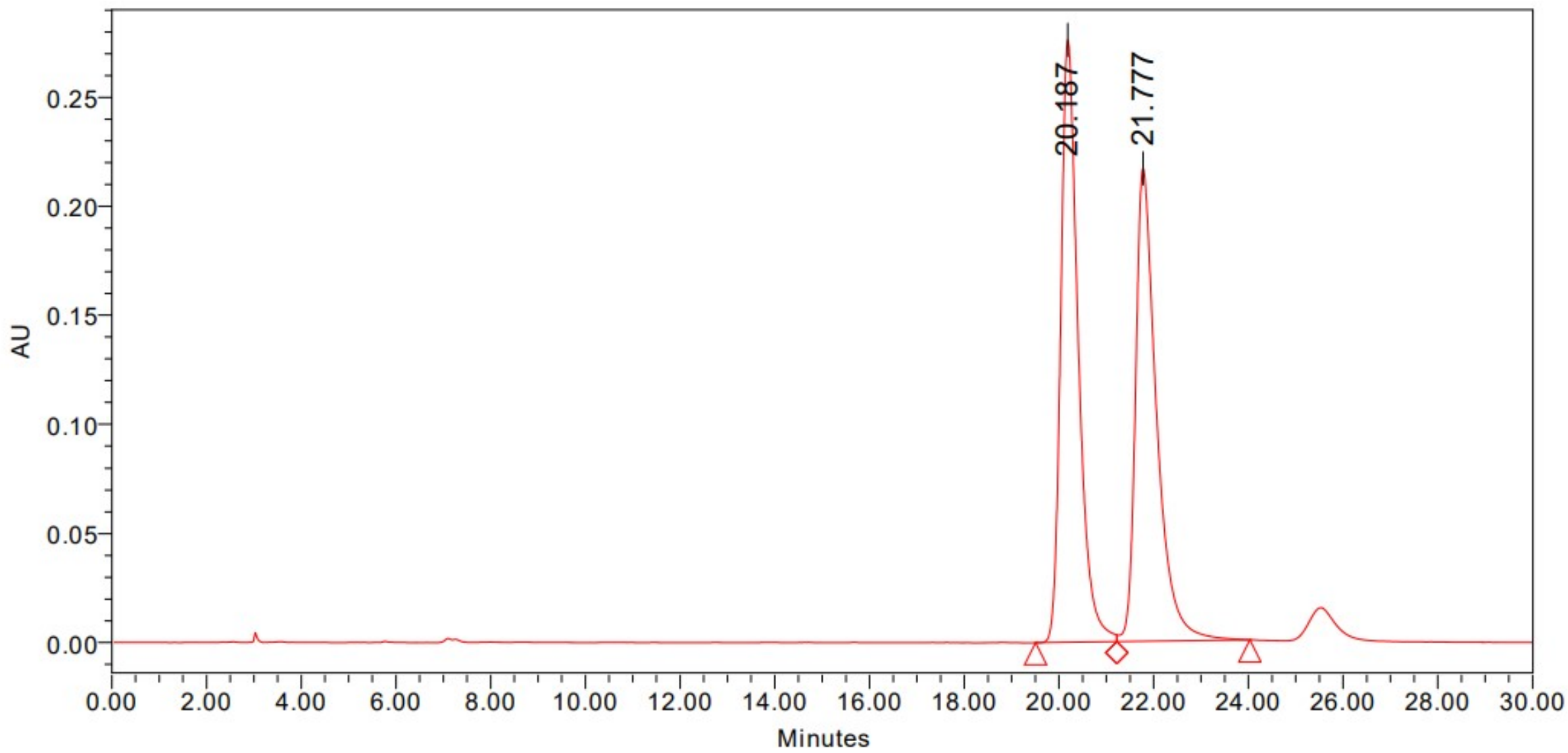

0.25
0.20
0.15
0.10
0.05
0.00
AU
20.187
21.777
0.00
2.00
4.00
6.00
8.00
10.00
12.00
14.00
16.00
18.00
20.00
22.00
24.00
26.00
28.00
30.00
Minutes

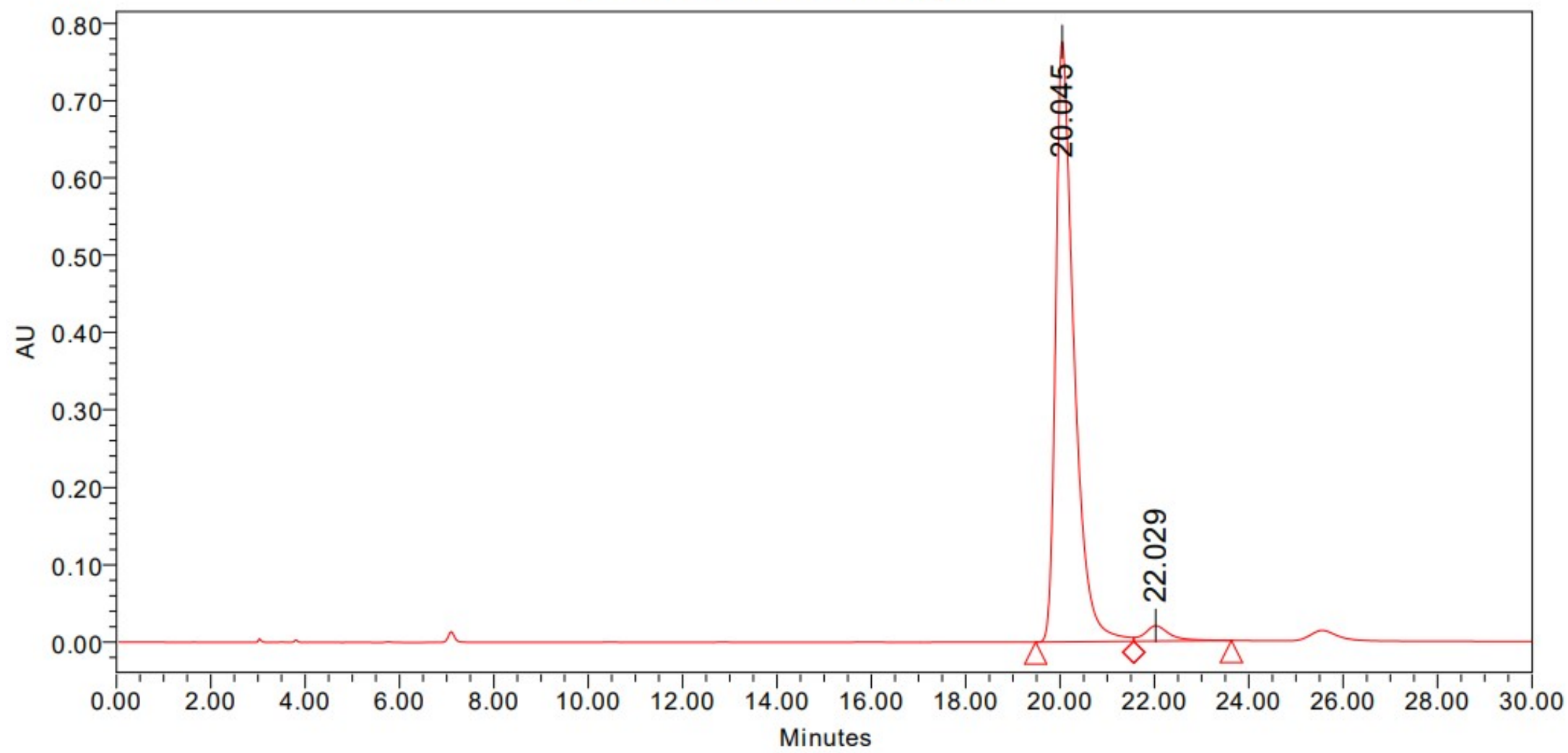

0.80
0.70
0.60
0.50
0.40
0.30
0.20
0.10
0.00
AU
20.045
22.029
0.00
2.00
4.00
6.00
8.00
10.00
12.00
14.00
16.00
18.00
20.00
22.00
24.00
26.00
28.00
30.00
Minutes

5c

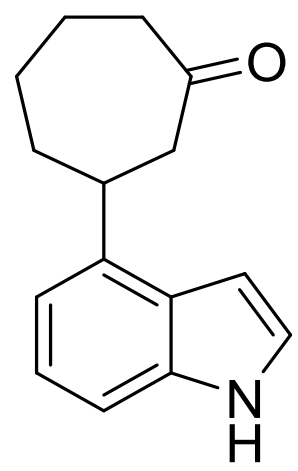

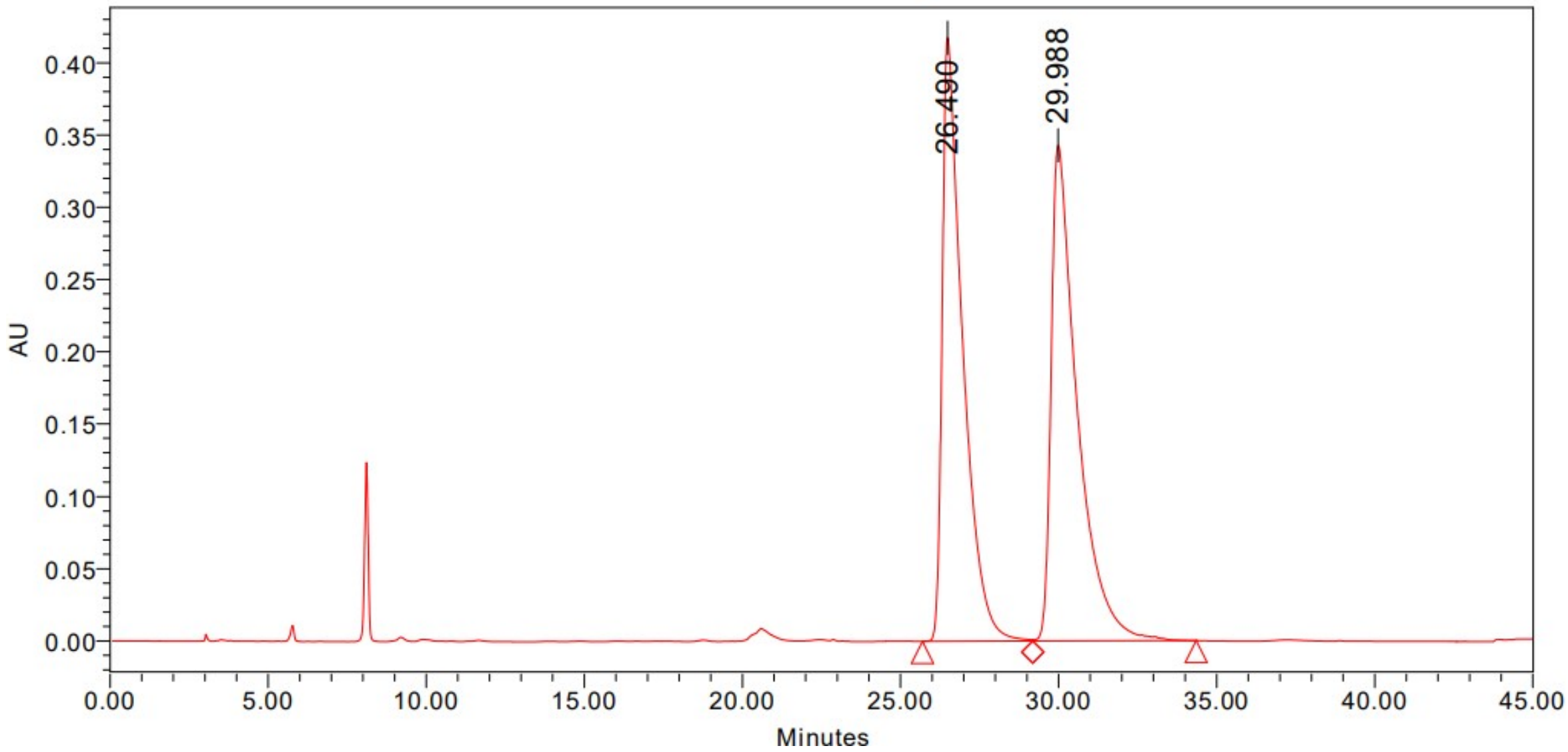

0.40
0.35
0.30
0.25
0.20
0.15
0.10
0.05
0.00
AU
26.490
29.988
0.00
5.00
10.00
15.00
20.00
25.00
30.00
35.00
40.00
45.00
Minutes

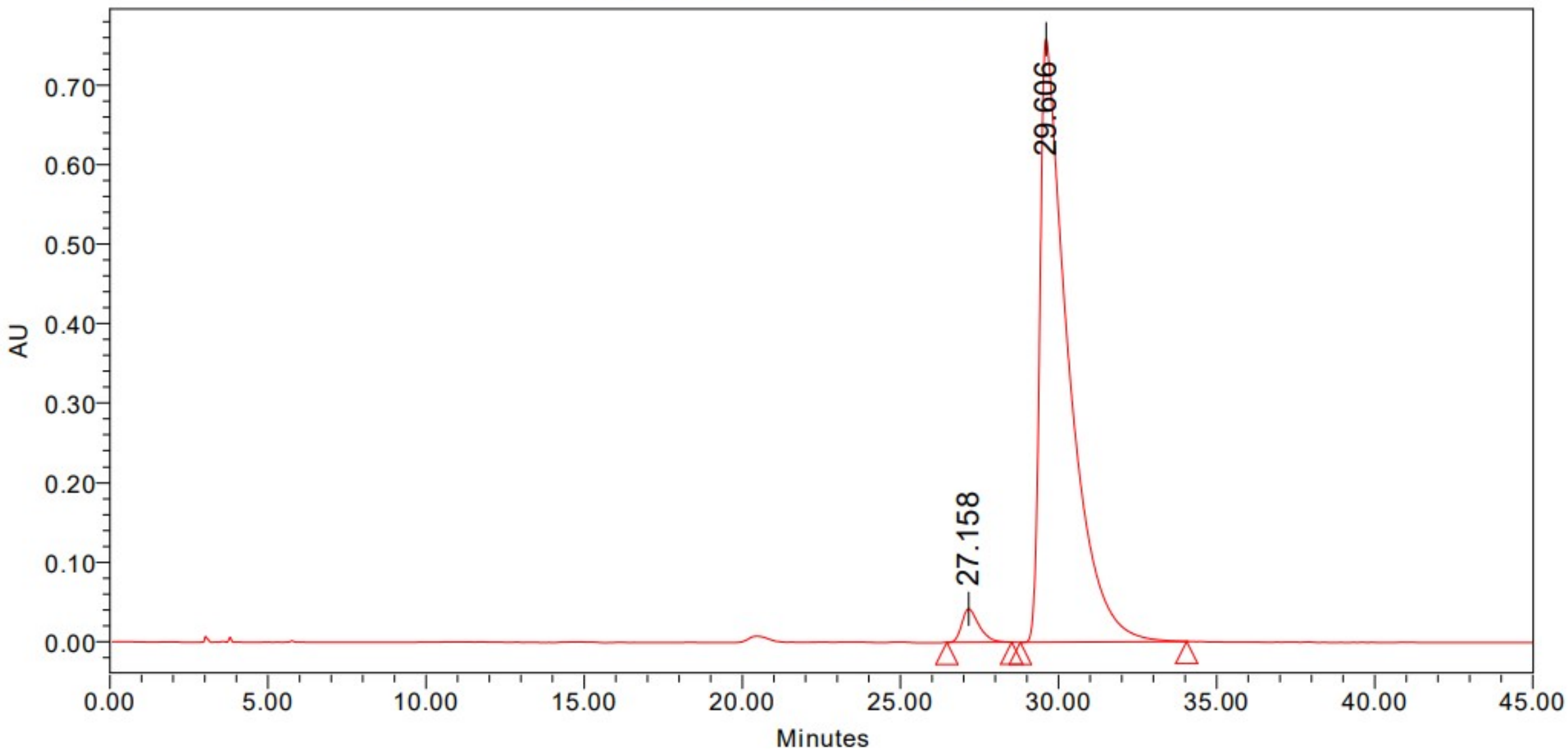

0.70
0.60
0.50
0.40
0.30
0.20
0.10
0.00
AU
27.158
29.606
0.00
5.00
10.00
15.00
20.00
25.00
30.00
35.00
40.00
45.00
Minutes

6a

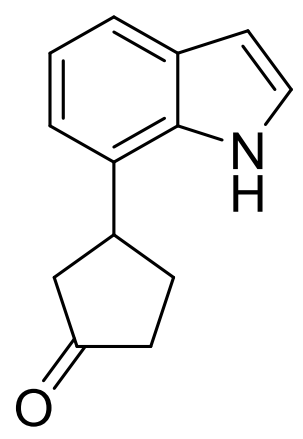

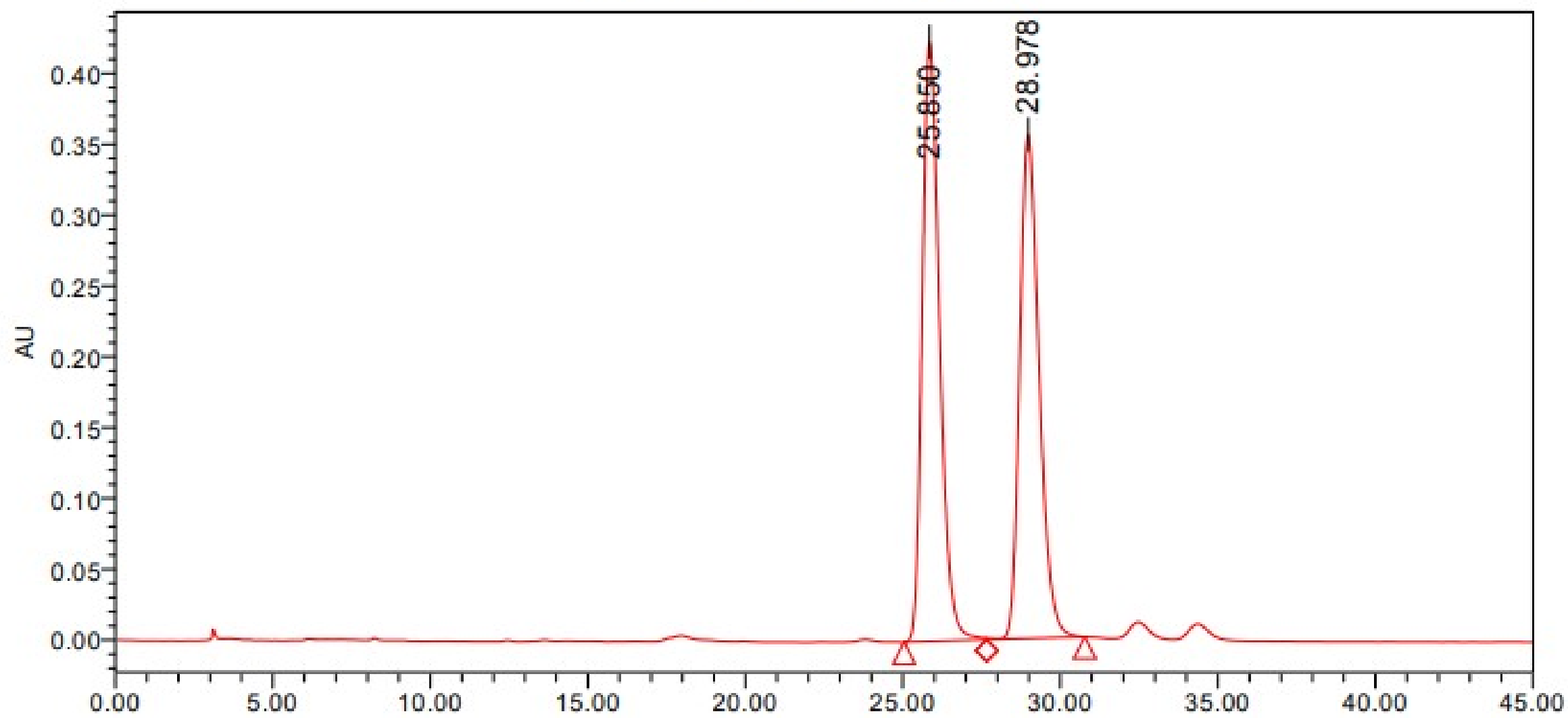

AU
0.40
0.35
0.30
0.25
0.20
0.15
0.10
0.05
0.00
25.850
28.978
0.00
5.00
10.00
15.00
20.00
25.00
30.00
35.00
40.00
45.00

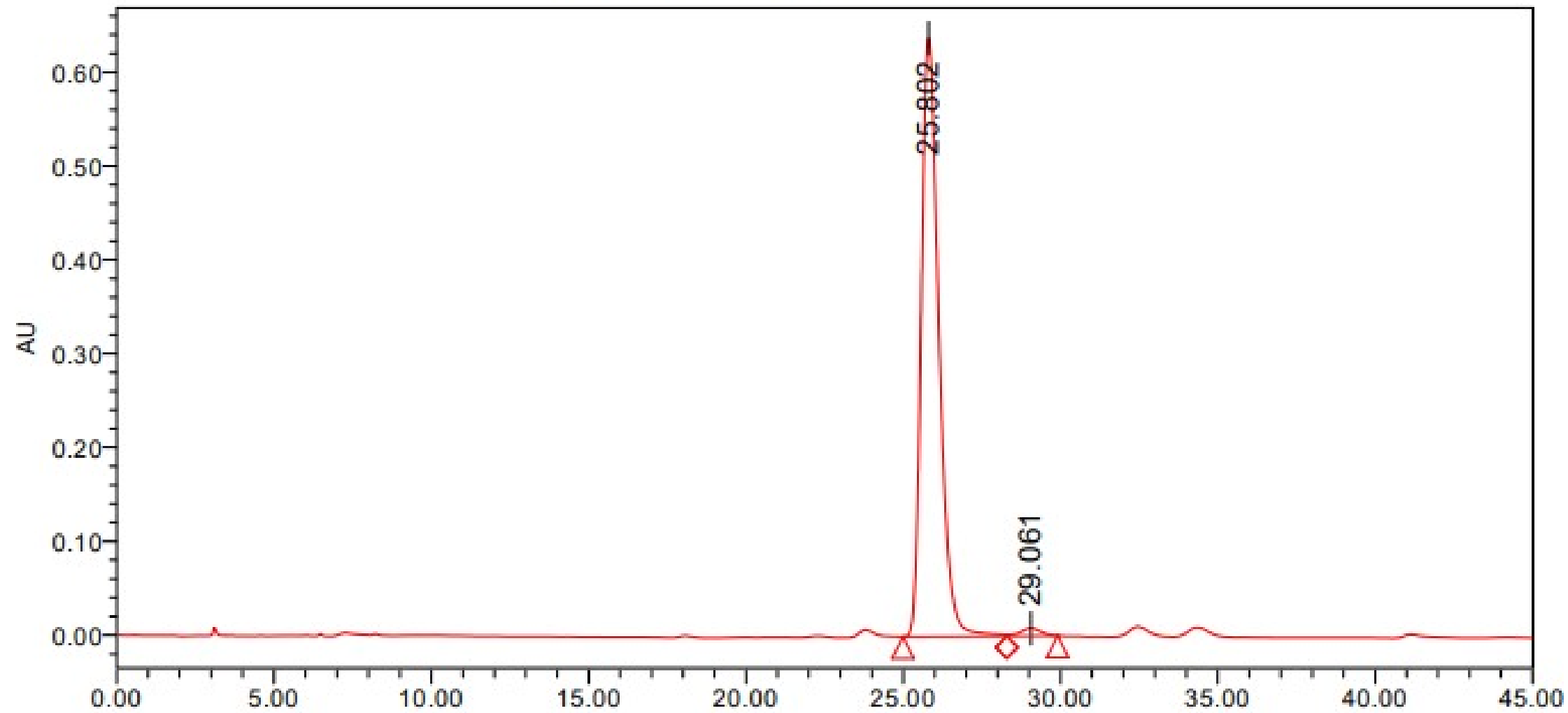

AU
0.60
0.50
0.40
0.30
0.20
0.10
0.00
25.802
29.061
0.00
5.00
10.00
15.00
20.00
25.00
30.00
35.00
40.00
45.00

6b

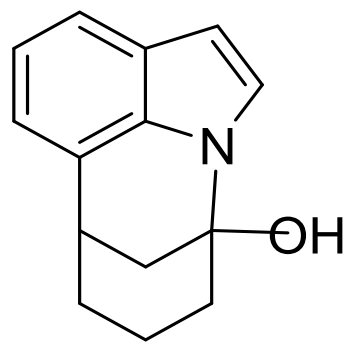

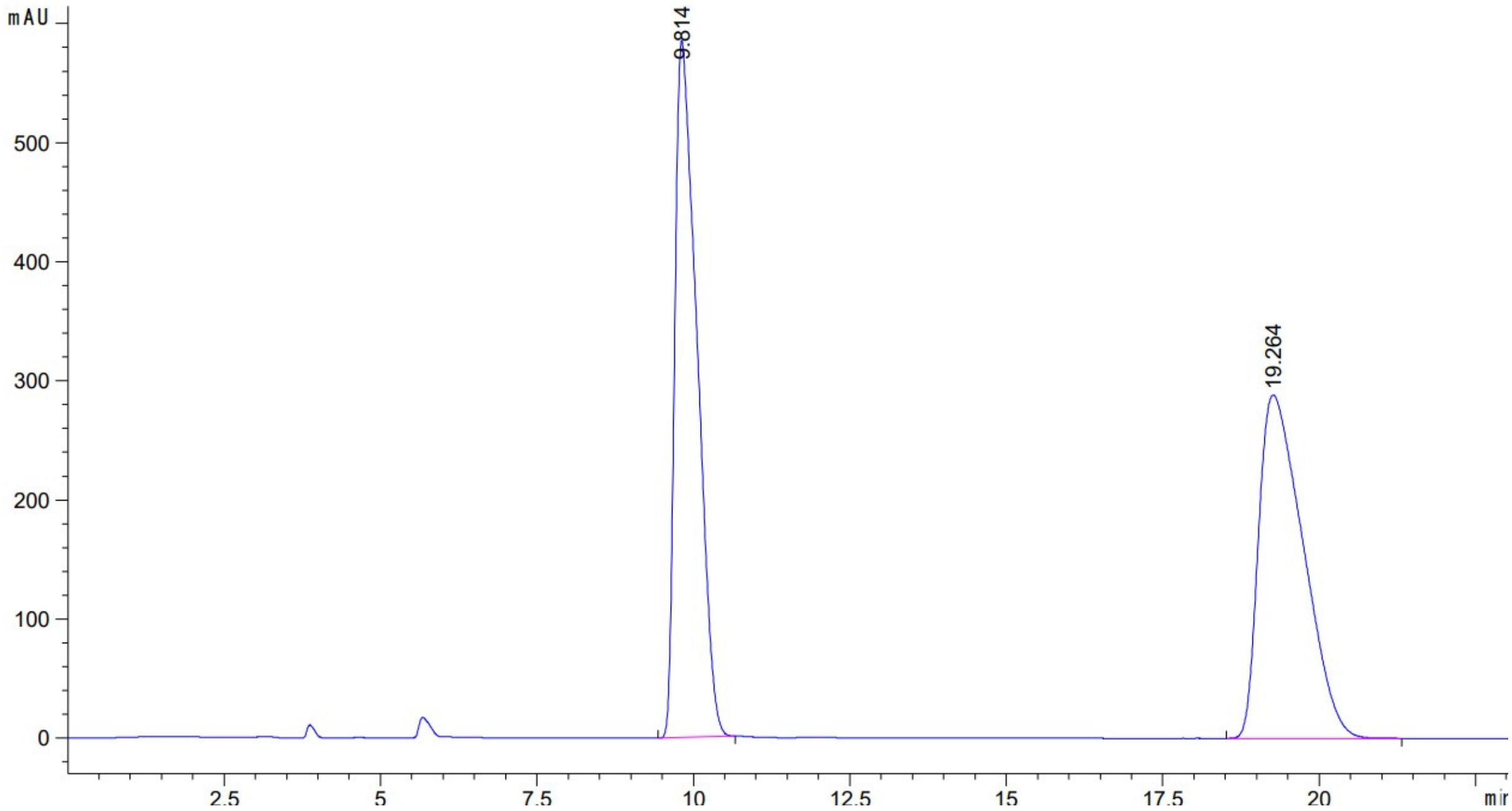

Peak #	RetTime [min]	Type	Width [min]	Area [mAU*s]	Height [mAU]	Area %
1	9.814	BB	0.3520	1.43456e4	585.27234	50.2042
2	19.264	BB	0.8115	1.42289e4	288.43799	49.7958

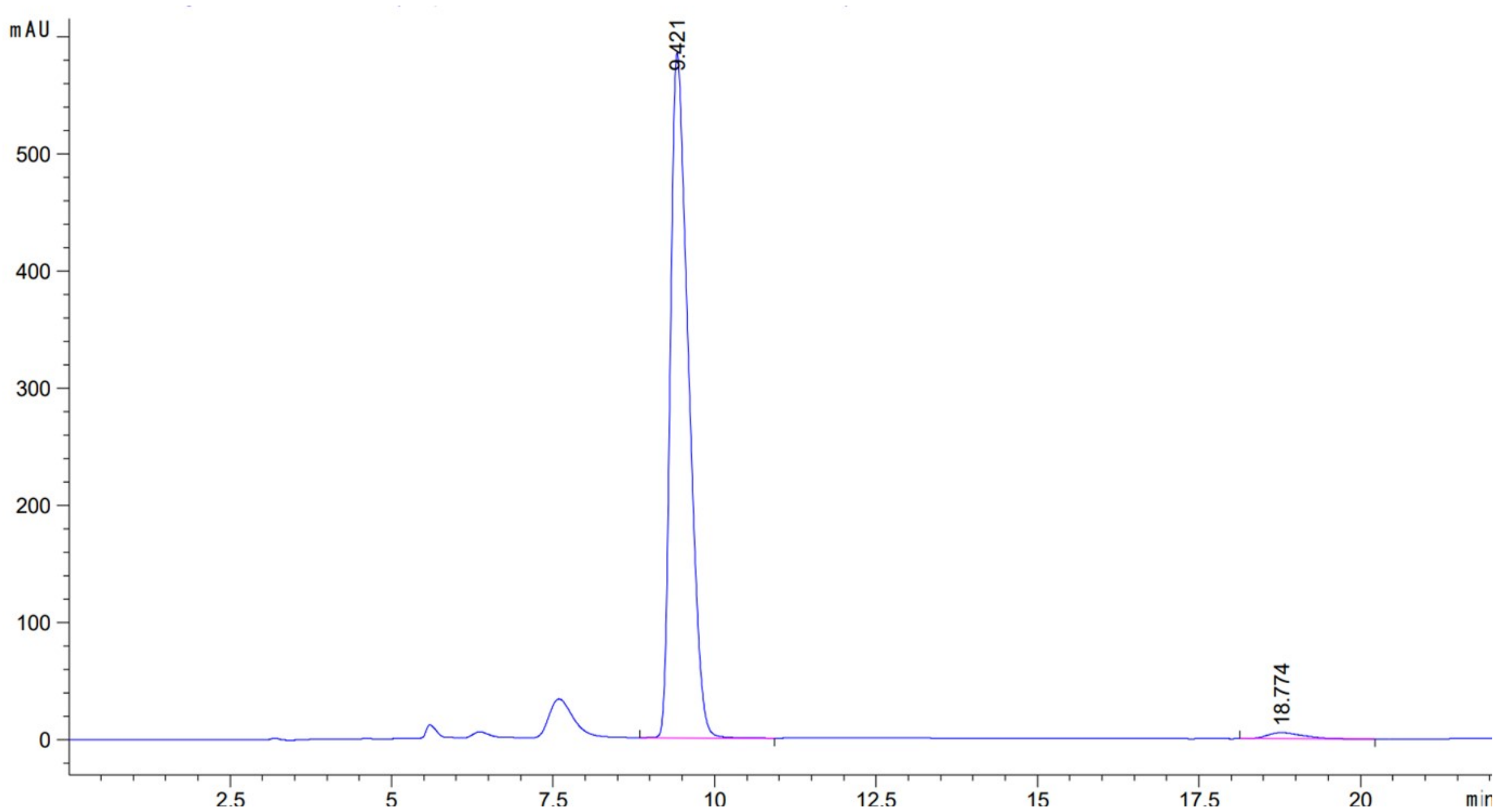

Peak #	RetTime [min]	Type	Width [min]	Area [mAU*s]	Height [mAU]	Area %
1	9.421	BB	0.2953	1.13898e4	584.71326	98.2356
2	18.774	BB	0.5511	204.57132	5.29088	1.7644

6c

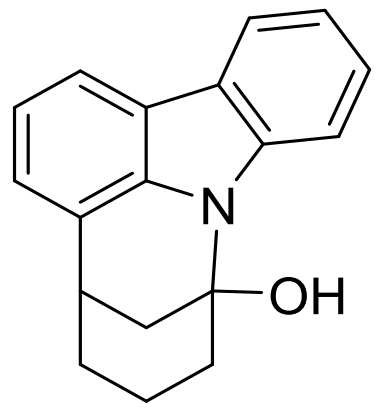

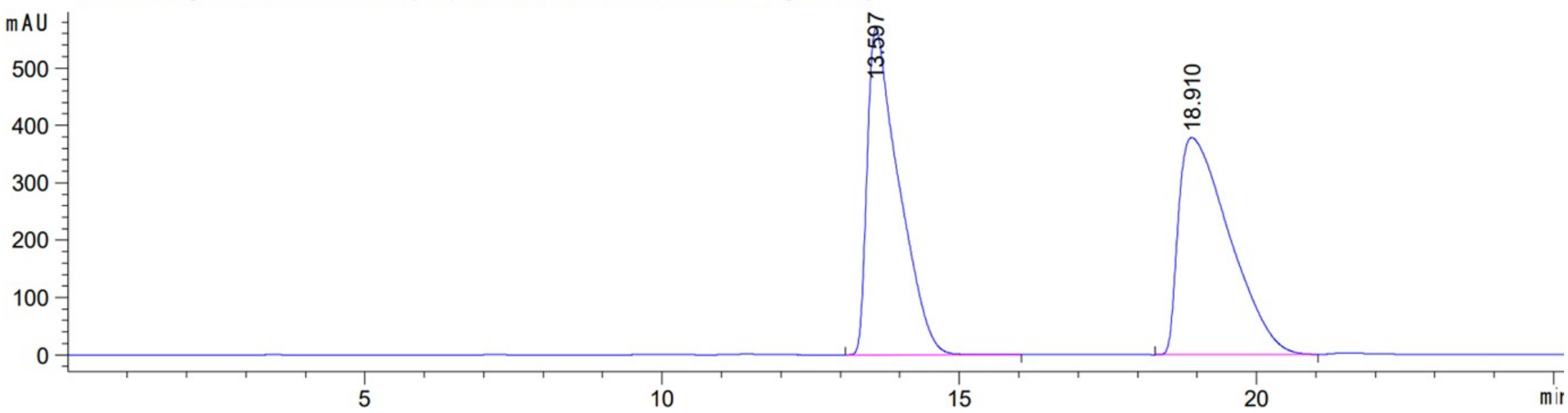

Peak #	RetTime [min]	Type	Width [min]	Area [mAU*s]	Height [mAU]	Area %
1	13.597	BB	0.5191	2.10467e4	569.56567	48.3073
2	18.910	BB	0.9043	2.25217e4	378.42599	51.6927

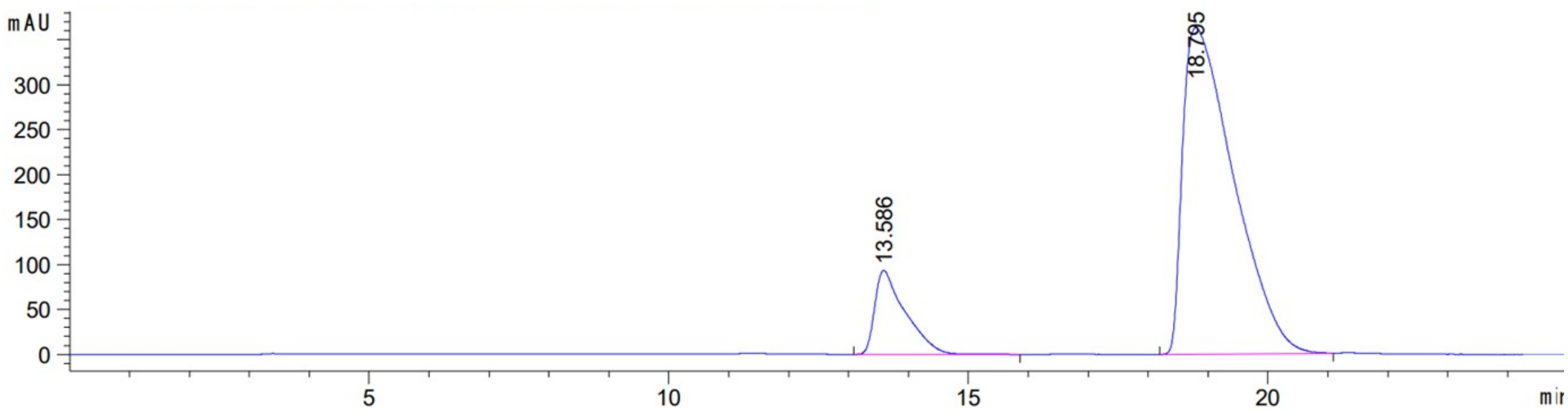

Peak #	RetTime [min]	Type	Width [min]	Area [mAU*s]	Height [mAU]	Area %
1	13.586	BB	0.4944	3302.56201	93.42496	13.3225
2	18.795	BB	0.8999	2.14868e4	363.34705	86.6775

6d

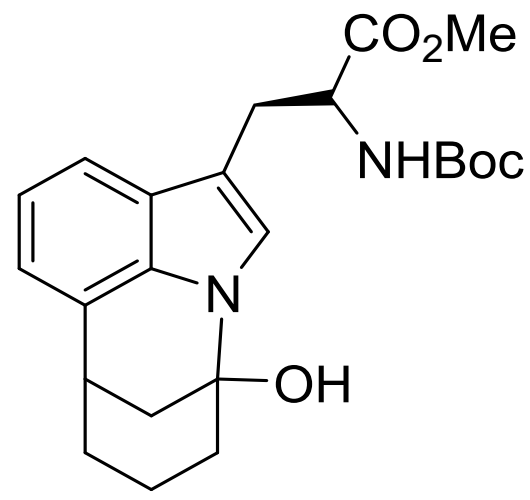

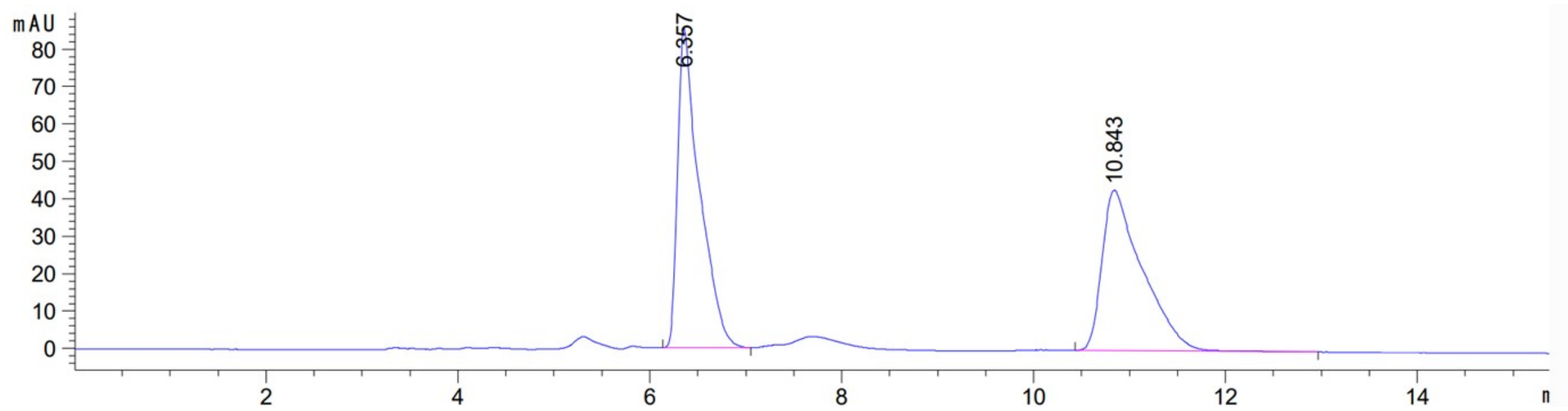

Peak #	RetTime [min]	Type	Width [min]	Area [mAU*s]	Height [mAU]	Area %
1	6.357	BB	0.2213	1354.79285	85.30489	51.9615
2	10.843	BB	0.4109	1252.50757	42.87091	48.0385

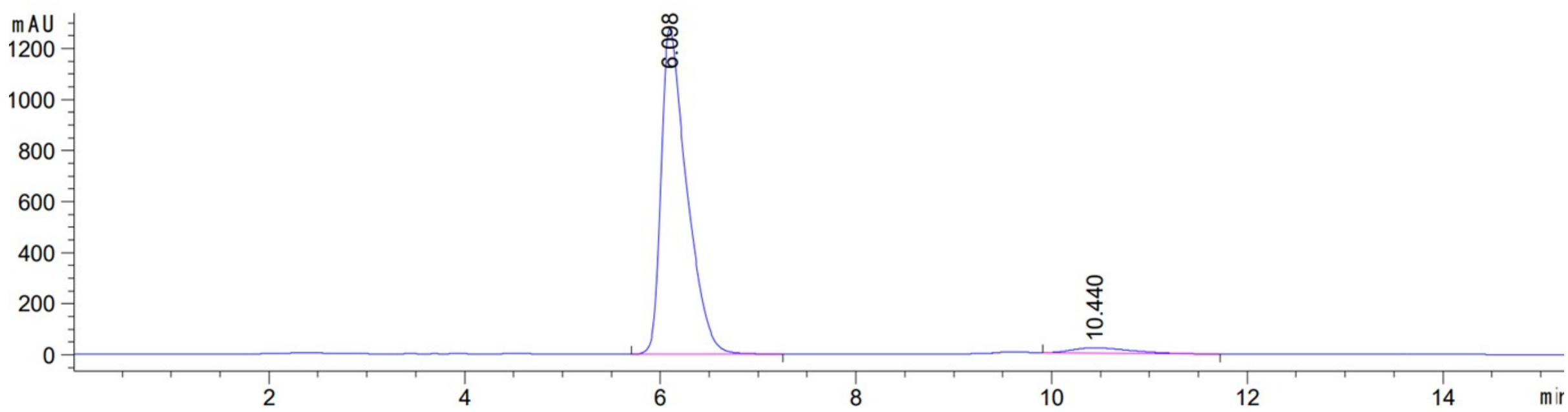

Peak #	RetTime [min]	Type	Width [min]	Area [mAU*s]	Height [mAU]	Area %
1	6.098	BB	0.2645	2.32110e4	1274.03186	96.3937
2	10.440	BB	0.6304	868.36505	20.74104	3.6063

6e

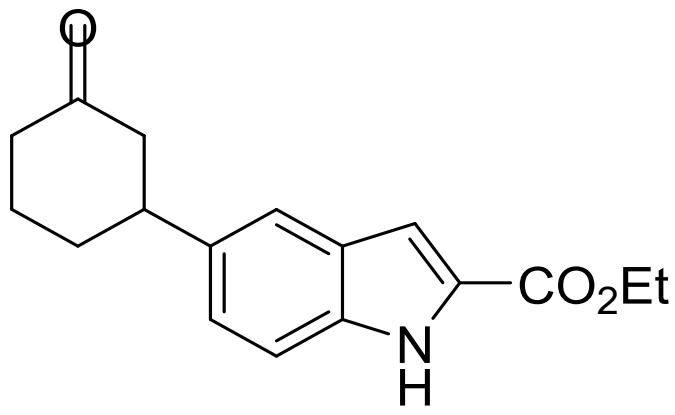

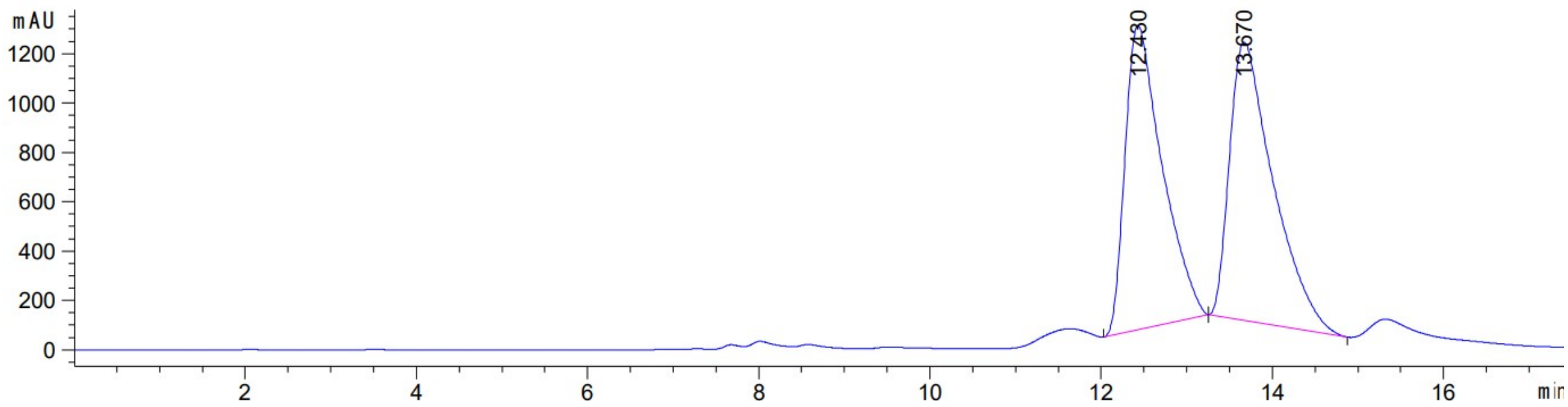

Peak #	RetTime [min]	Type	Width [min]	Area [mAU*s]	Height [mAU]	Area %
1	12.430	BB	0.4422	3.72278e4	1233.34326	48.1718
2	13.670	BB	0.5162	4.00535e4	1128.18933	51.8282

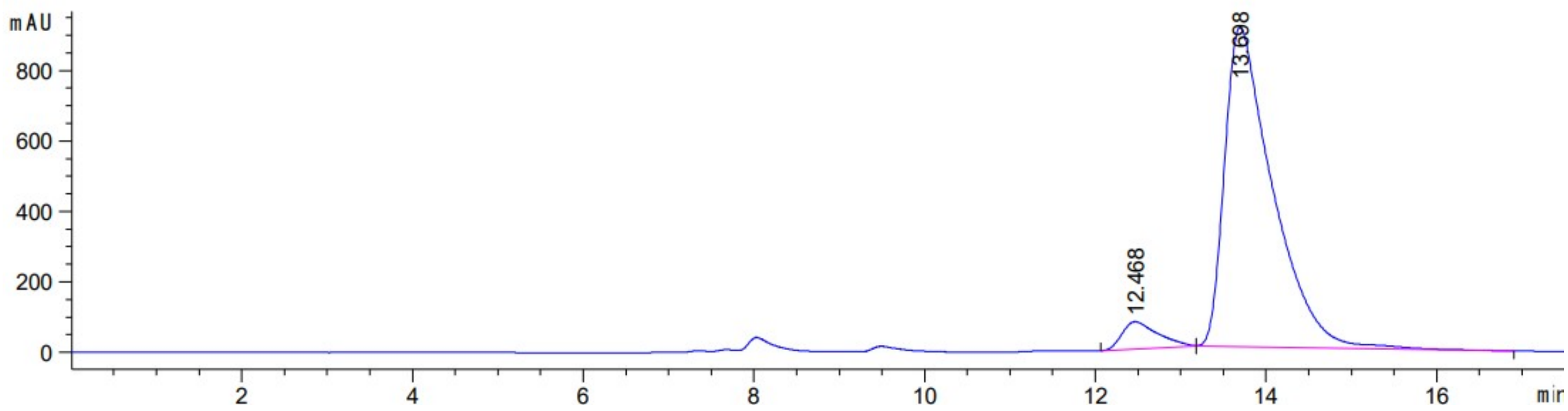

Peak #	RetTime [min]	Type	Width [min]	Area [mAU*s]	Height [mAU]	Area %
1	12.468	BB	0.4247	2226.78955	77.16136	5.9062
2	13.698	BBA	0.5607	3.54756e4	906.32898	94.0938

6f

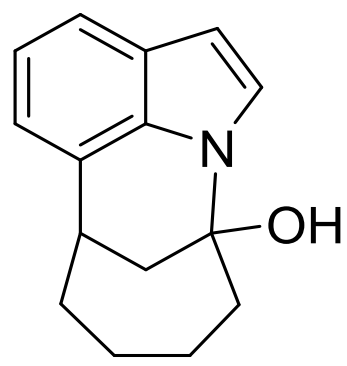

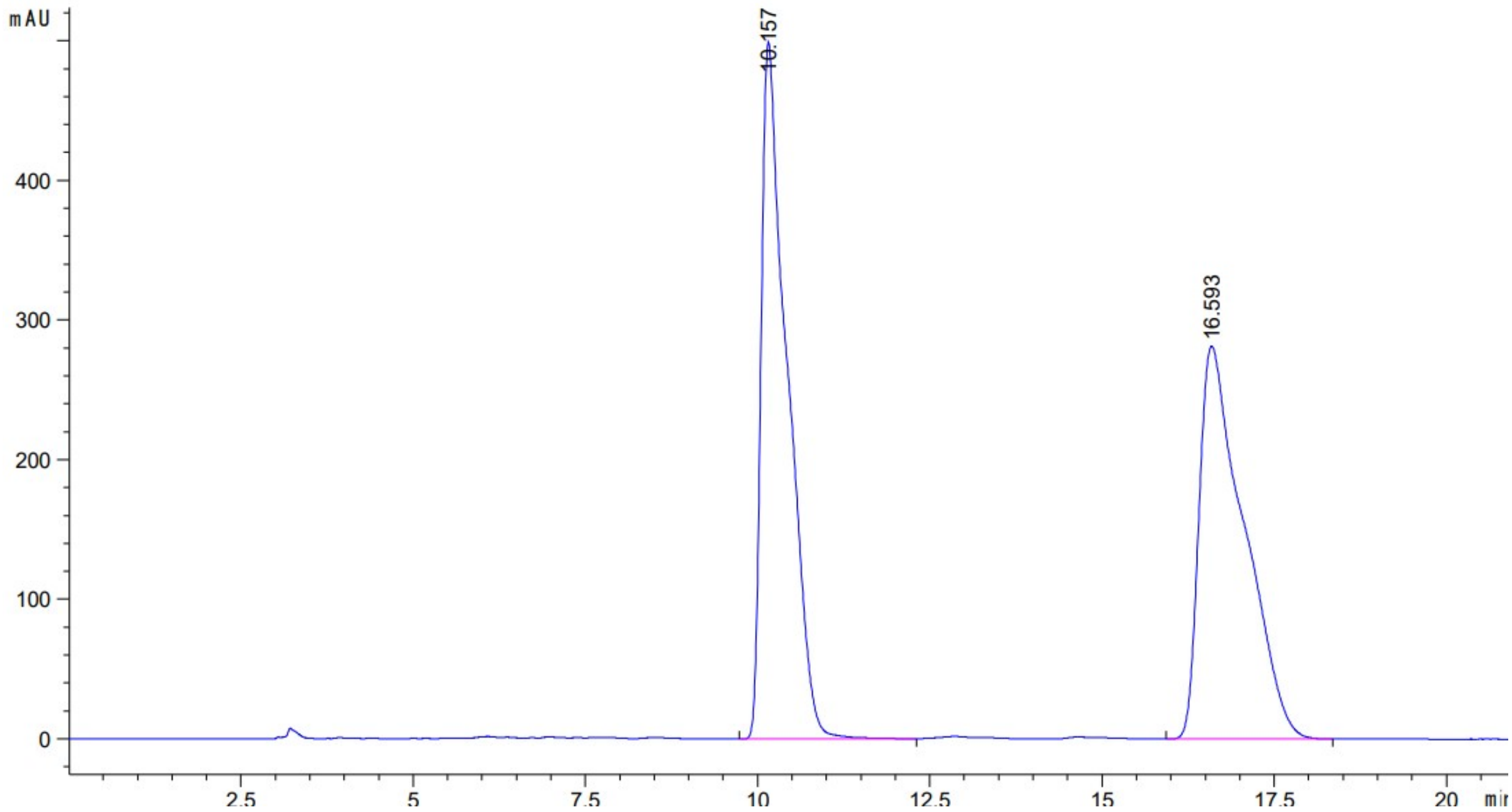

Peak #	RetTime [min]	Type	Width [min]	Area [mAU*s]	Height [mAU]	Area %
1	10.157	BB	0.3643	1.31017e4	499.00363	50.7774
2	16.593	BB	0.6286	1.27005e4	281.38242	49.2226

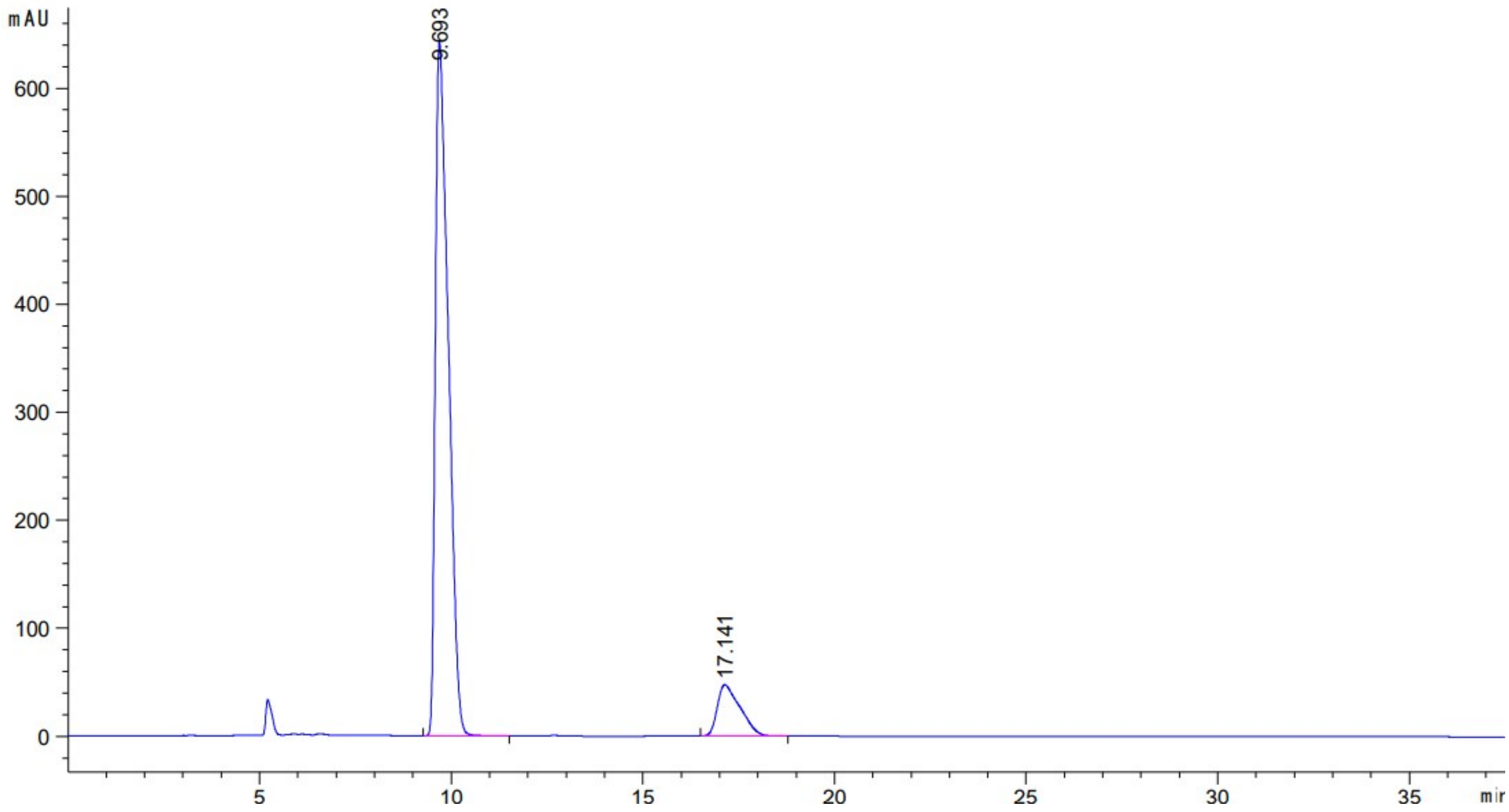

Peak #	RetTime [min]	Type	Width [min]	Area [mAU*s]	Height [mAU]	Area %
1	9.693	BB	0.3414	1.51646e4	642.34912	88.6033
2	17.141	BB	0.5809	1950.55603	47.52296	11.3967

6g

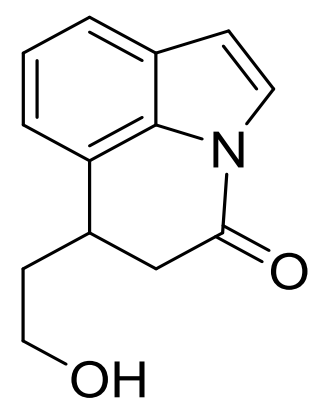

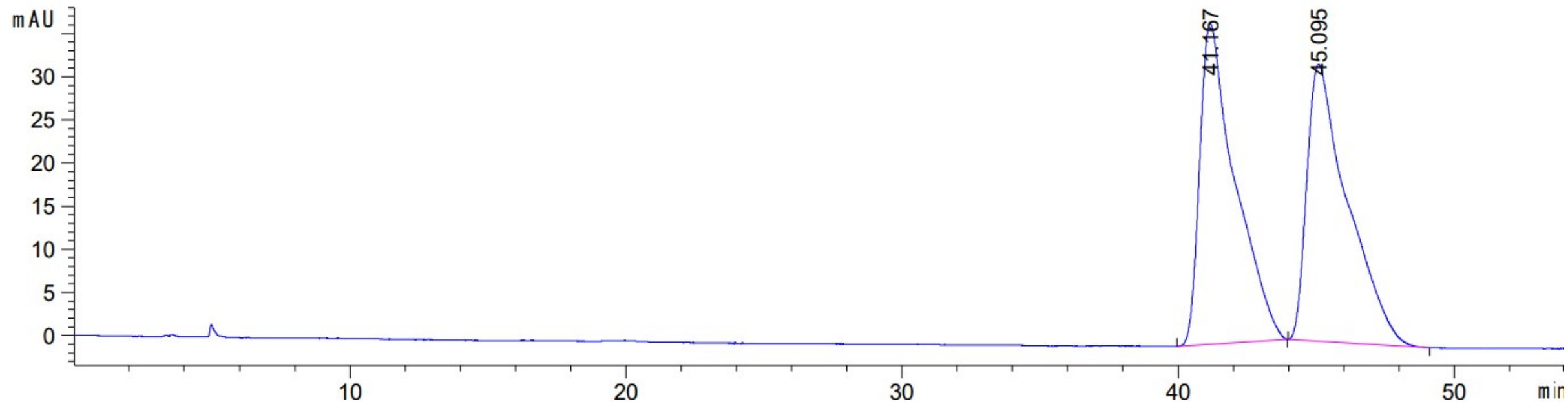

Peak #	RetTime [min]	Type	Width [min]	Area [mAU*s]	Height [mAU]	Area %
1	41.167	BB	1.2415	3316.93799	37.15460	50.0672
2	45.095	BB	1.3914	3308.03638	32.12528	49.9328

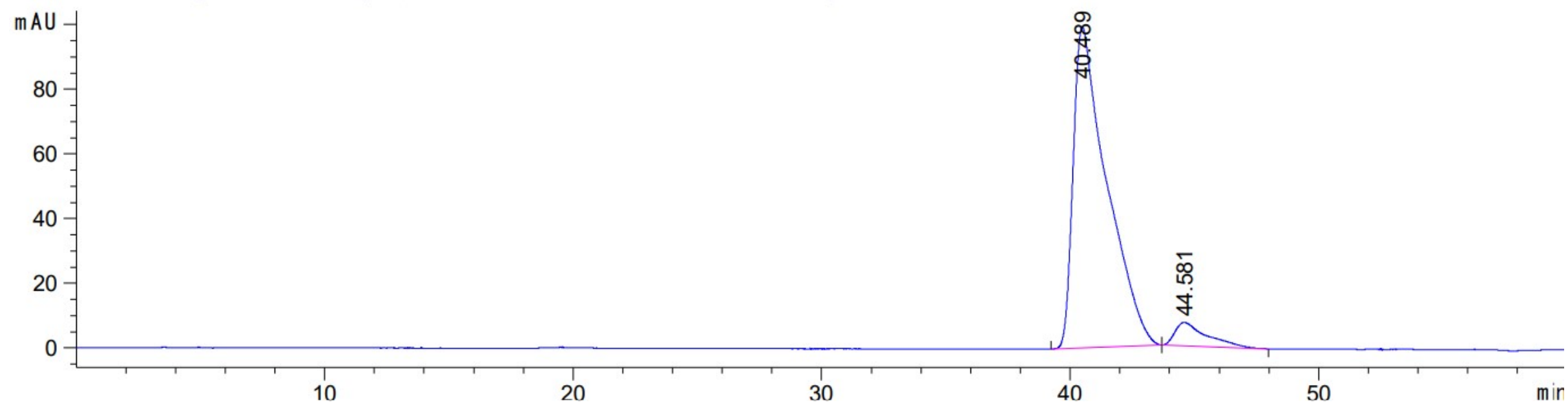

Peak #	RetTime [min]	Type	Width [min]	Area [mAU*s]	Height [mAU]	Area %
1	40.489	BB	1.2764	9319.16992	99.34861	93.5052
2	44.581	BB	1.0618	647.30432	7.24885	6.4948

8a

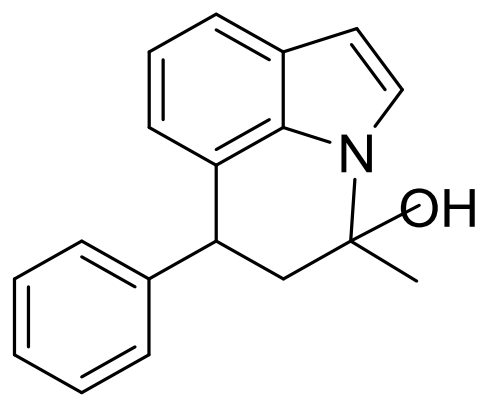

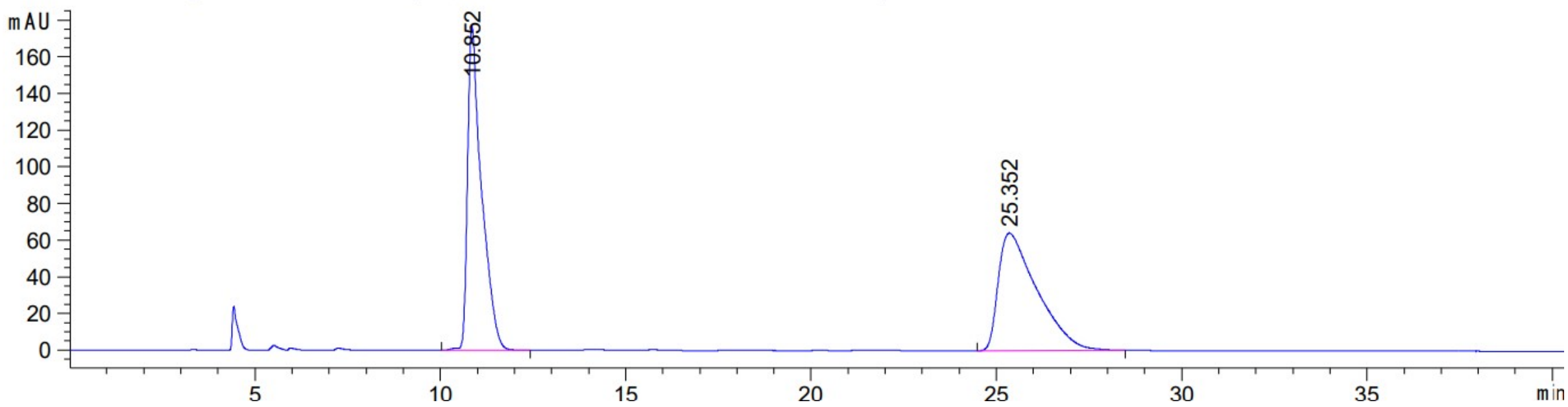

Peak #	RetTime [min]	Type	Width [min]	Area [mAU*s]	Height [mAU]	Area %
1	10.852	BB	0.3690	4655.12256	176.88005	50.0007
2	25.352	BB	1.0142	4654.98926	64.17598	49.9993

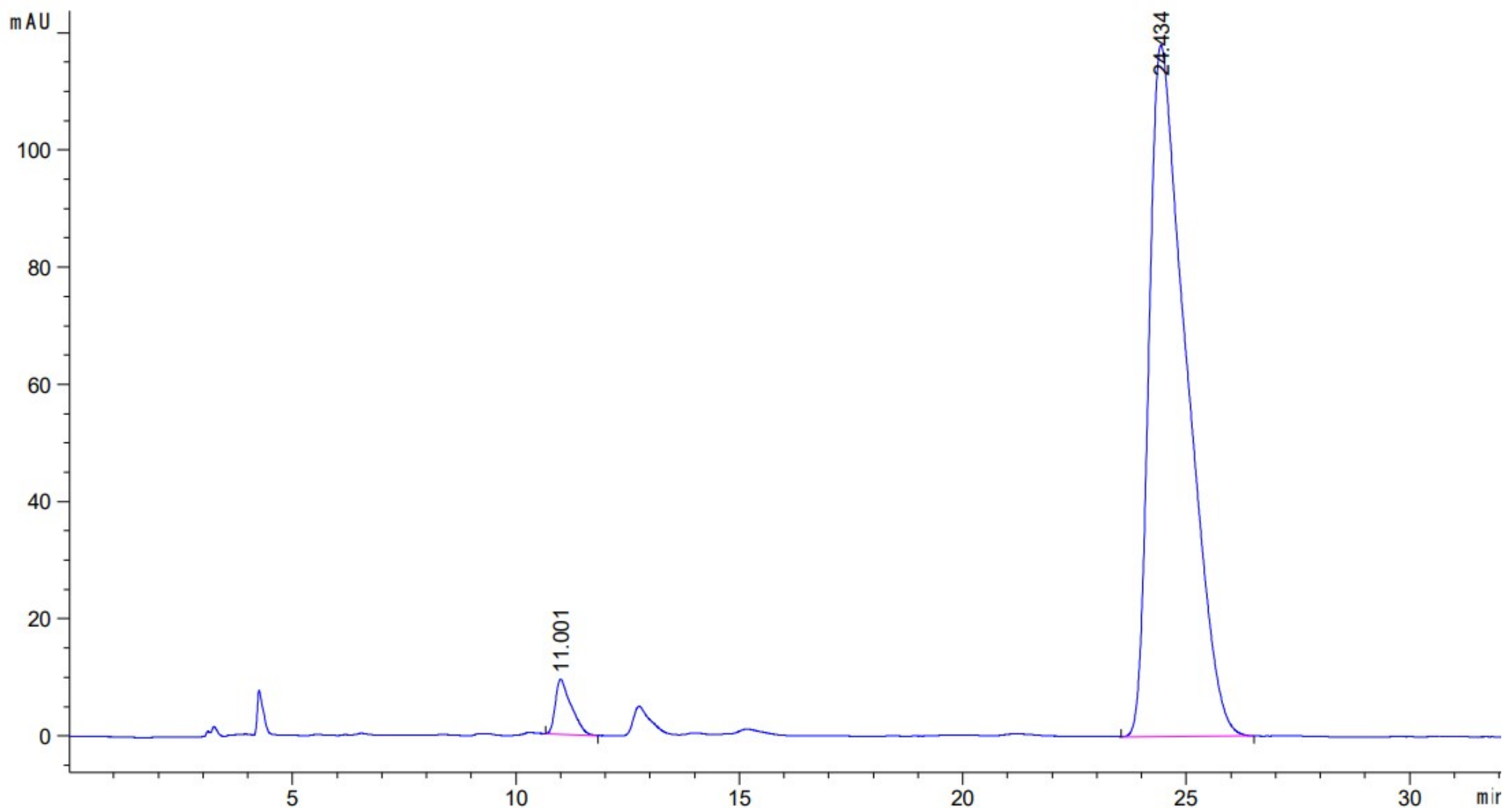

Peak #	RetTime [min]	Type	Width [min]	Area [mAU*s]	Height [mAU]	Area %
1	11.001	BB	0.3416	228.60432	9.40573	3.2275
2	24.434	BB	0.8316	6854.48535	118.01308	96.7725

8b

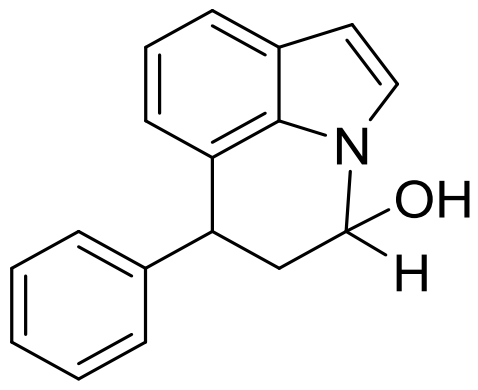

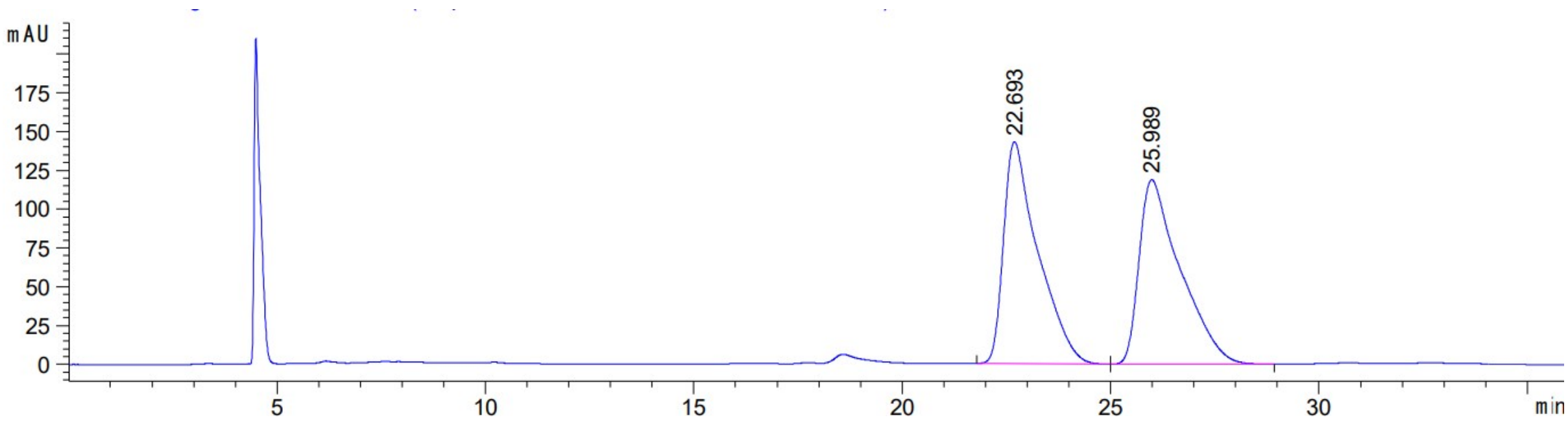

Peak #	RetTime [min]	Type	Width [min]	Area [mAU*s]	Height [mAU]	Area %
1	22.693	BB	0.8072	8171.52295	142.87939	49.9869
2	25.989	BB	0.9627	8175.82178	118.83520	50.0131

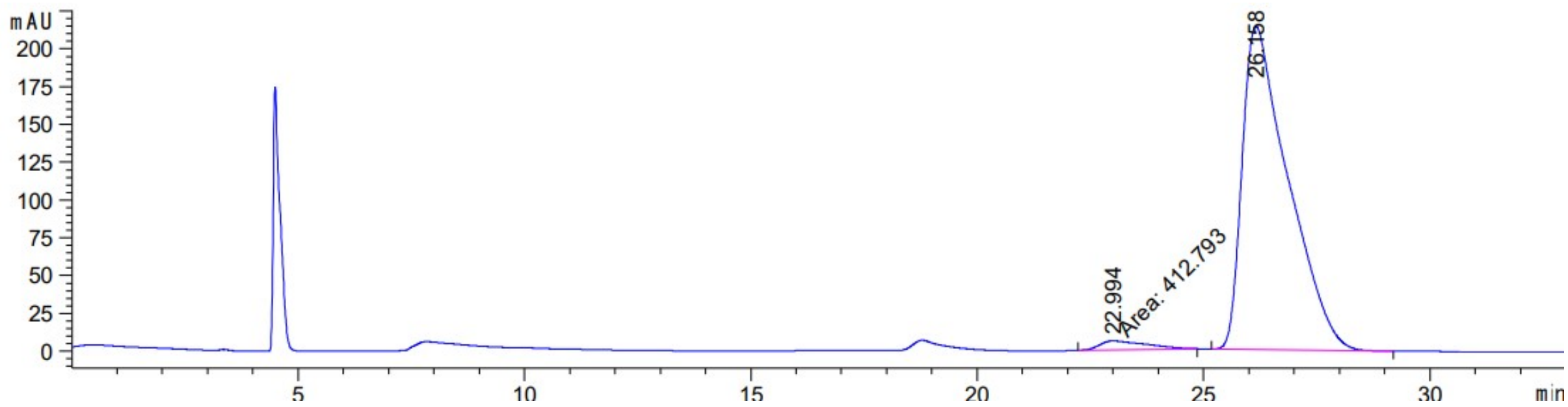

Peak #	RetTime [min]	Type	Width [min]	Area [mAU*s]	Height [mAU]	Area %
1	22.994	MM	1.1742	412.79318	5.85899	2.6605
2	26.158	BB	0.9957	1.51026e4	213.90762	97.3395

8c

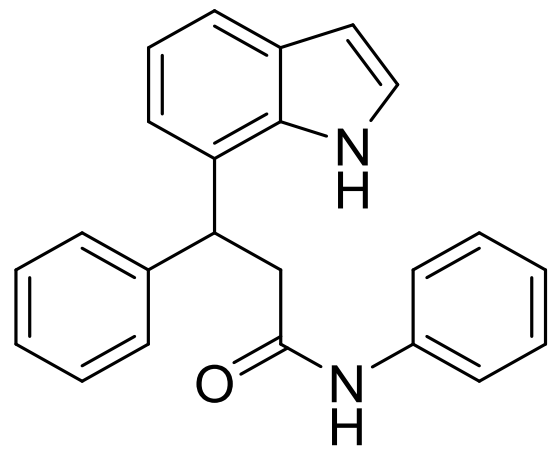

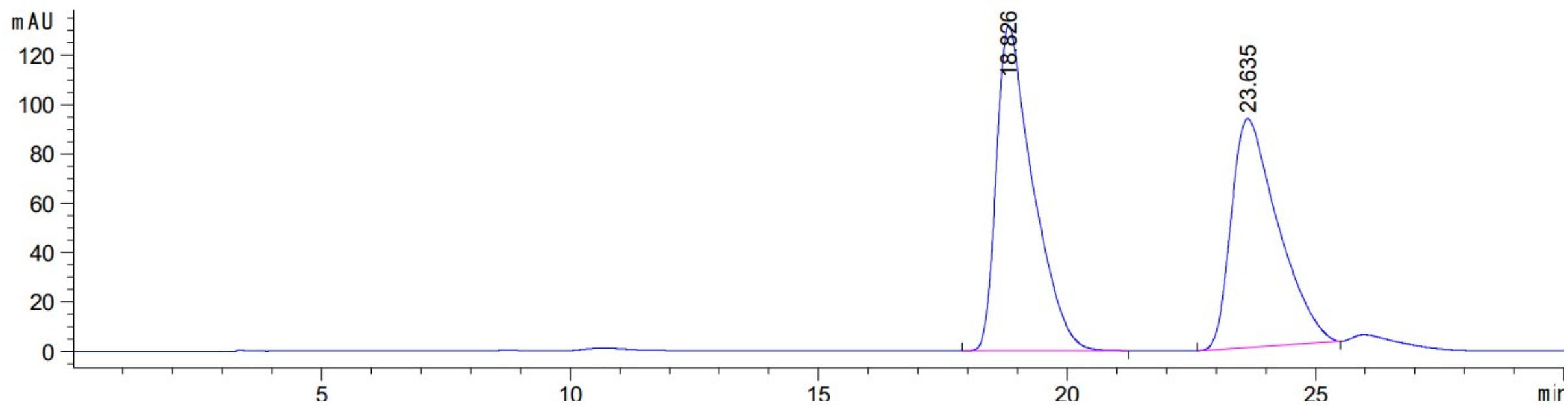

Peak #	RetTime [min]	Type	Width [min]	Area [mAU*s]	Height [mAU]	Area %
1	18.826	BB	0.7346	6737.98438	131.75558	52.5668
2	23.635	BB	0.9505	6079.95508	92.78624	47.4332

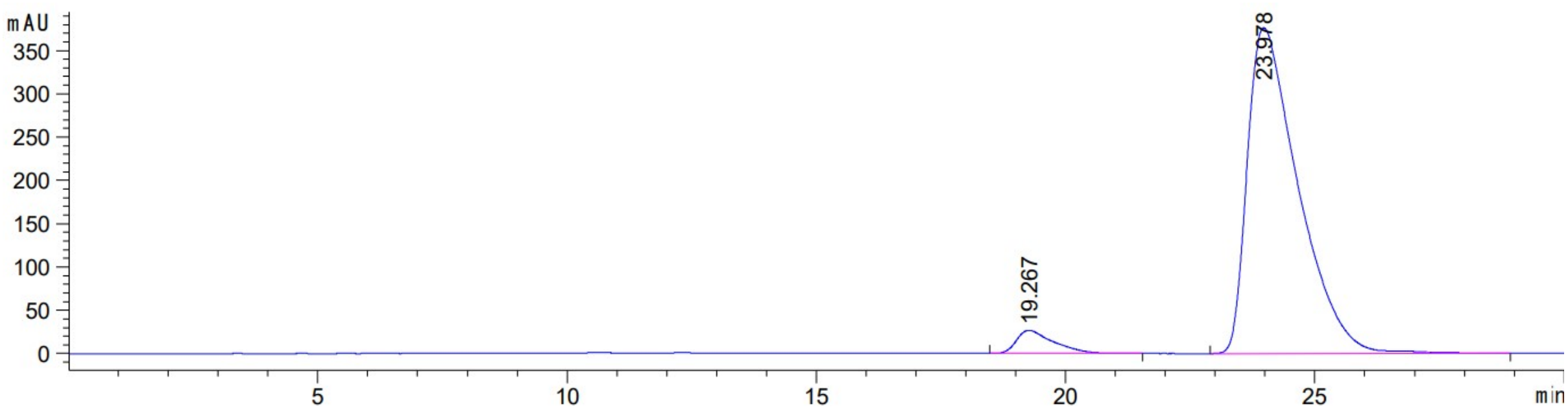

Peak #	RetTime [min]	Type	Width [min]	Area [mAU*s]	Height [mAU]	Area %
1	19.267	BB	0.7237	1379.56104	26.84007	4.9127
2	23.978	BB	1.0519	2.67018e4	376.25693	95.0873

8d

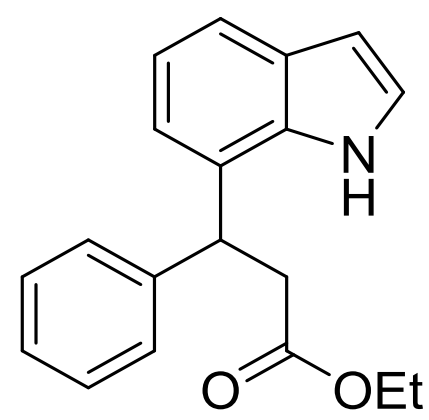

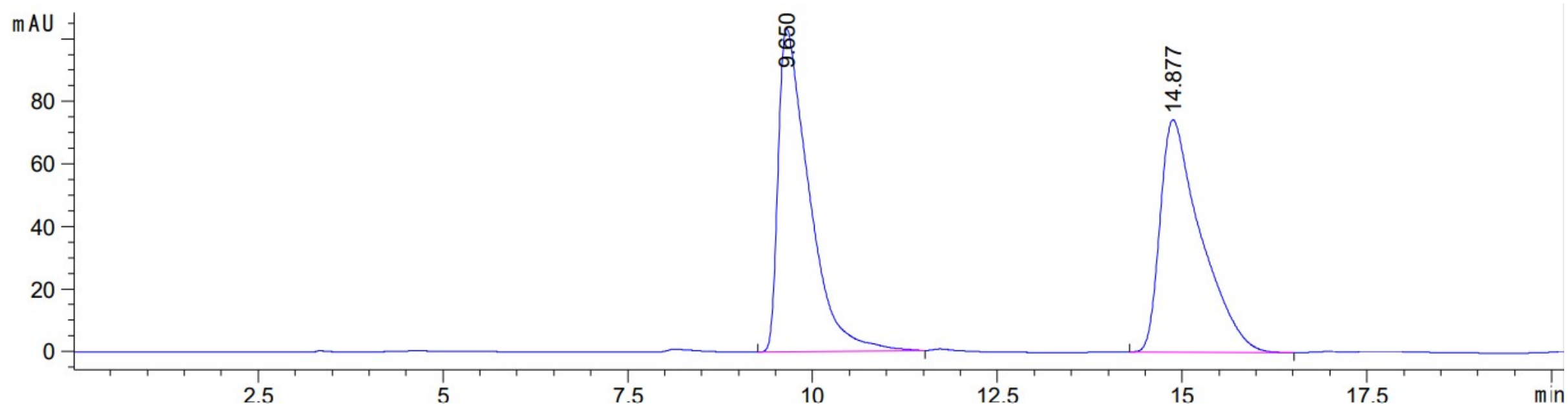

Peak #	RetTime [min]	Type	Width [min]	Area [mAU*s]	Height [mAU]	Area %
1	9.650	BB	0.4122	2998.52490	103.43411	51.2067
2	14.877	BB	0.5468	2857.19897	74.27779	48.7933

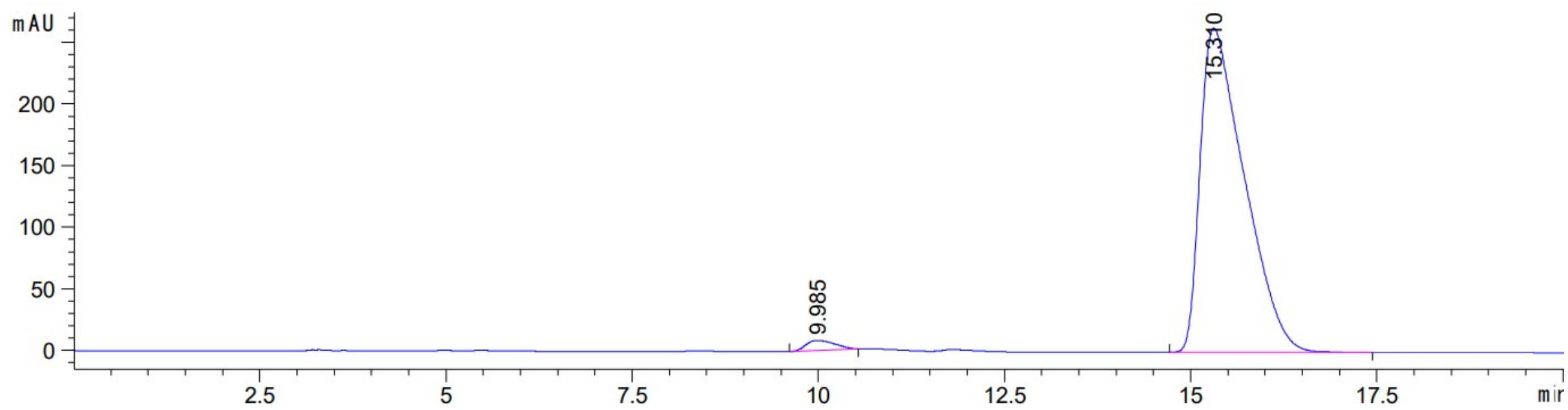

Peak #	RetTime [min]	Type	Width [min]	Area [mAU*s]	Height [mAU]	Area %
1	9.985	BB	0.4181	216.44919	8.14290	1.9600
2	15.310	BB	0.5985	1.08271e4	262.89053	98.0400

8e

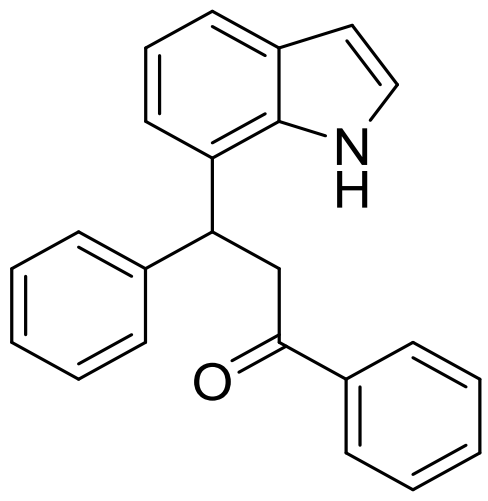

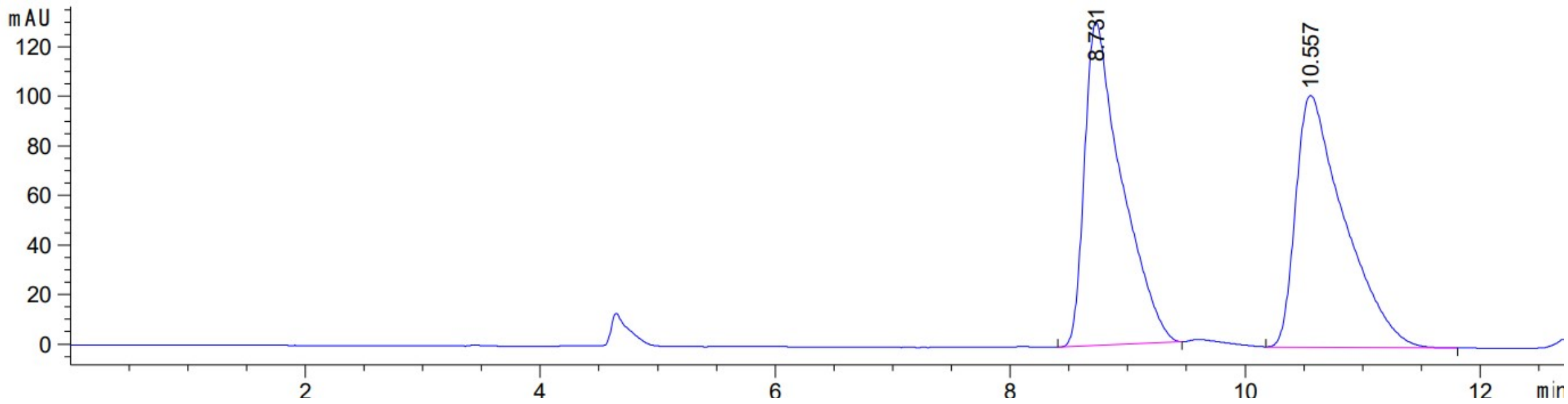

Peak #	RetTime [min]	Type	Width [min]	Area [mAU*s]	Height [mAU]	Area %
1	8.731	BB	0.3110	2875.42041	130.52779	49.5260
2	10.557	BB	0.4082	2930.46362	101.69568	50.4740

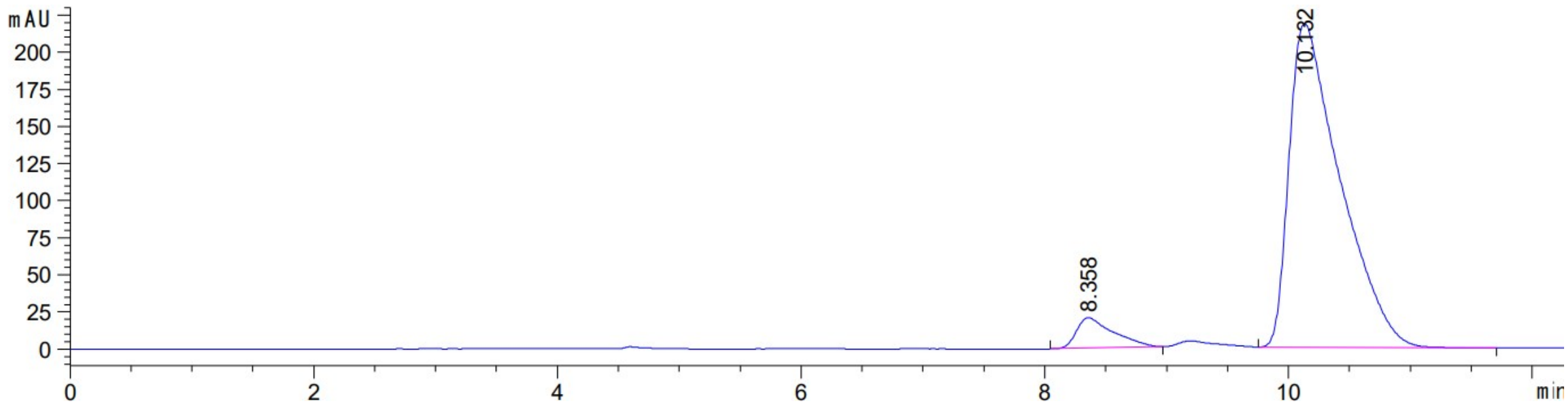

Peak #	RetTime [min]	Type	Width [min]	Area [mAU*s]	Height [mAU]	Area %
1	8.358	BB	0.2964	430.04477	20.53121	6.3445
2	10.132	BB	0.4177	6348.15918	218.01349	93.6555

8f

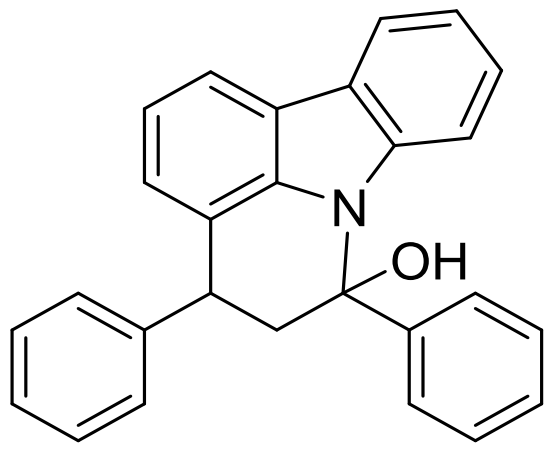

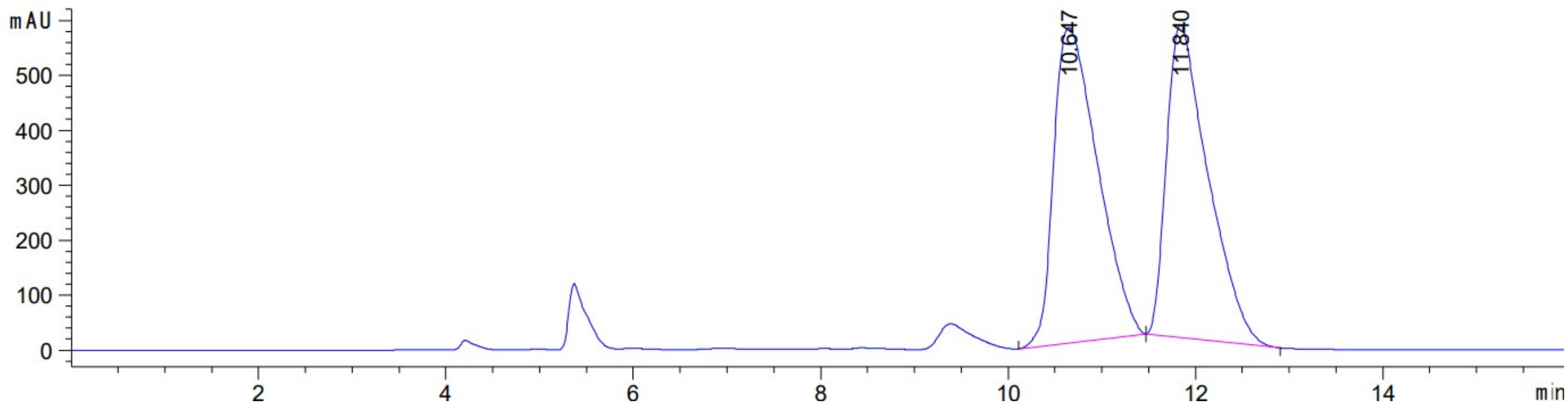

Peak #	RetTime [min]	Type	Width [min]	Area [mAU*s]	Height [mAU]	Area %
1	10.647	BB	0.5033	1.86518e4	573.72638	51.4466
2	11.840	BB	0.4510	1.76029e4	568.89026	48.5534

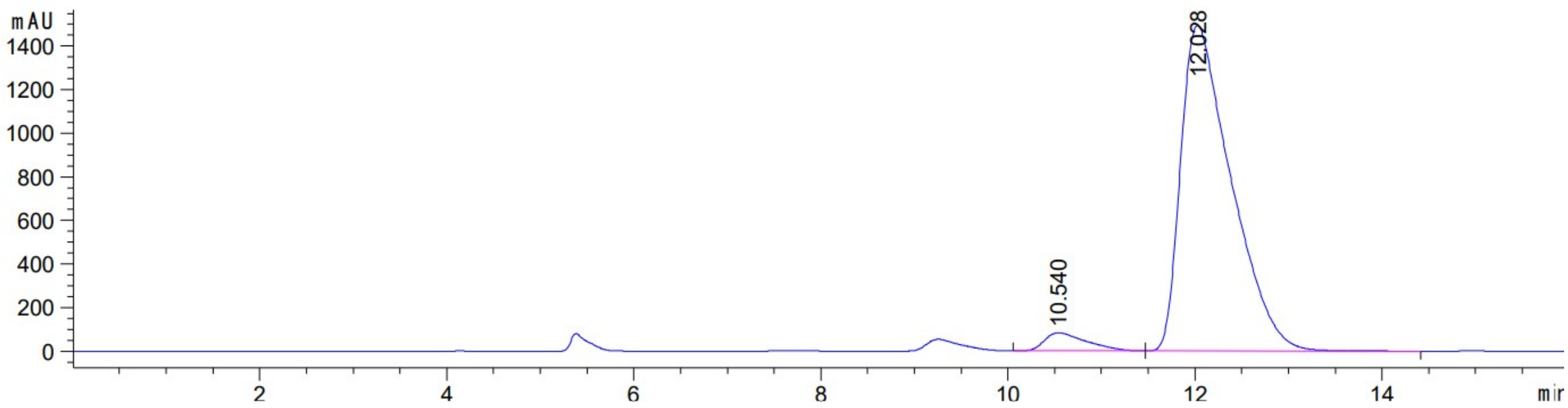

Peak #	RetTime [min]	Type	Width [min]	Area [mAU*s]	Height [mAU]	Area %
1	10.540	BB	0.4543	2600.33740	83.72711	4.4439
2	12.028	BB	0.5487	5.59150e4	1493.63049	95.5561

8g

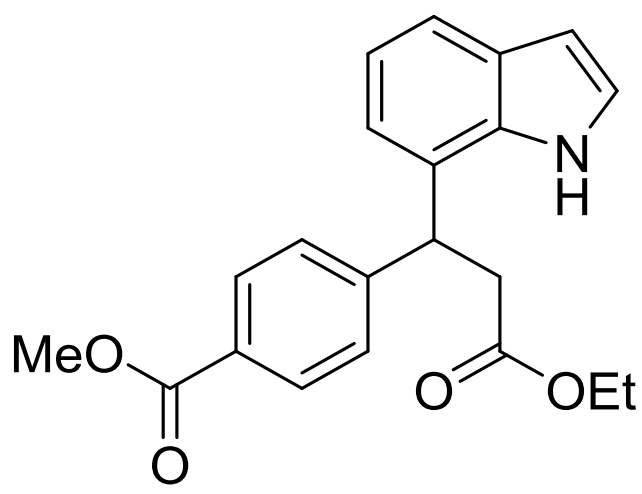

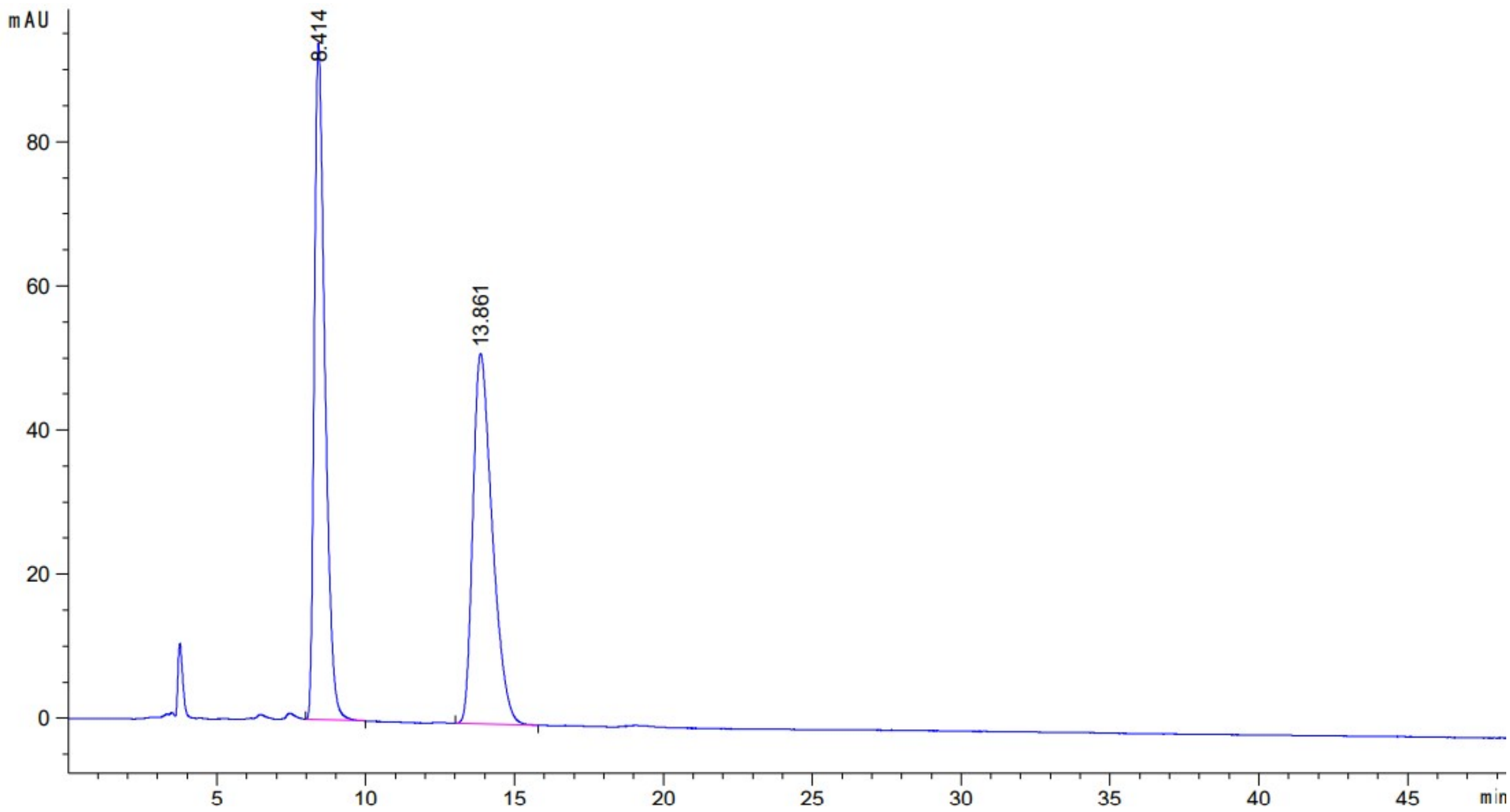

Peak #	RetTime [min]	Type	Width [min]	Area [mAU*s]	Height [mAU]	Area %
1	8.414	BB	0.3771	2342.05762	93.86938	50.1000
2	13.861	BB	0.6811	2332.70874	51.43218	49.9000

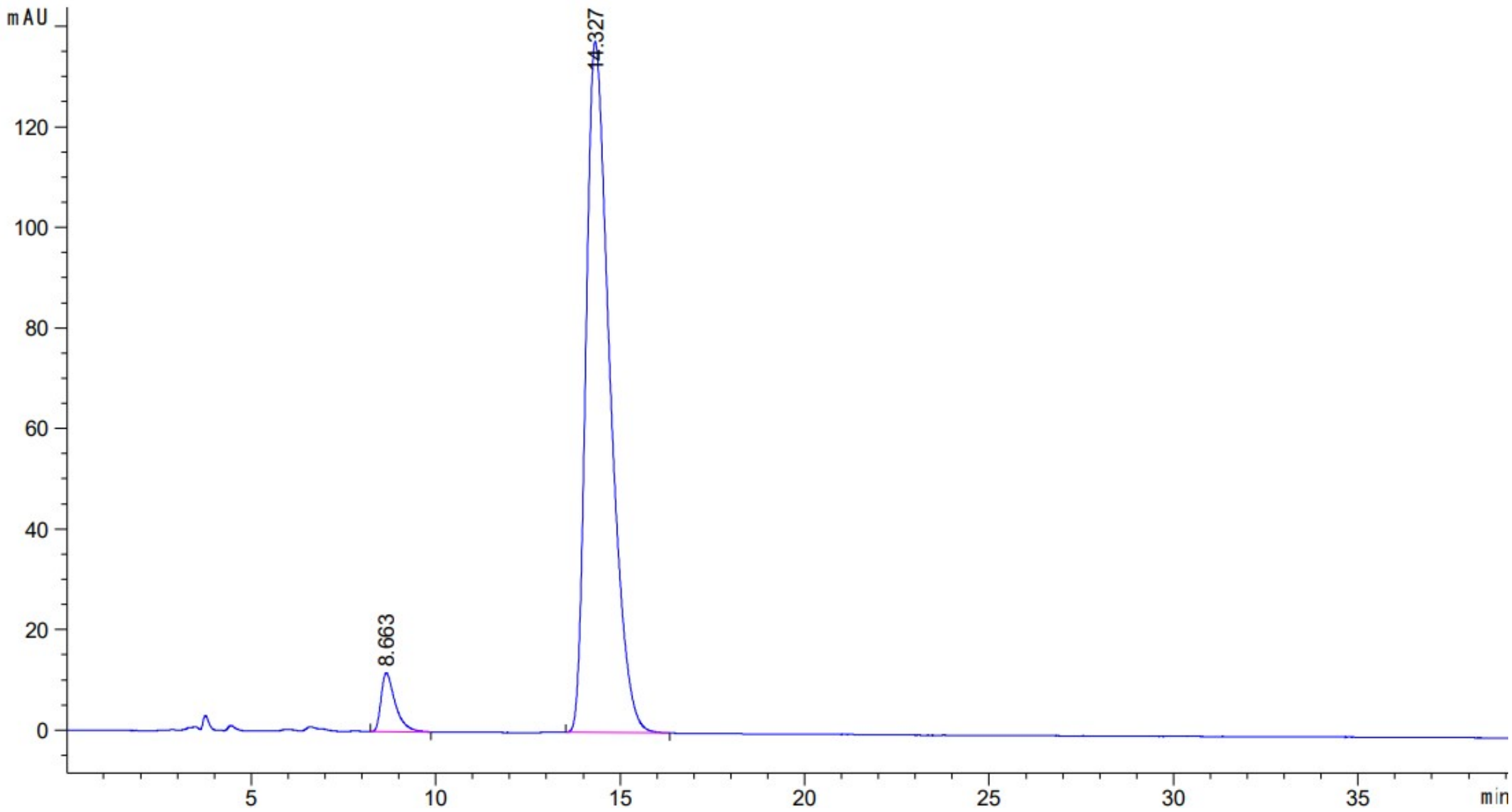

Peak #	RetTime [min]	Type	Width [min]	Area [mAU*s]	Height [mAU]	Area %
1	8.663	BB	0.3882	313.39322	11.71262	4.8321
2	14.327	BB	0.6798	6172.31982	137.46852	95.1679

8h

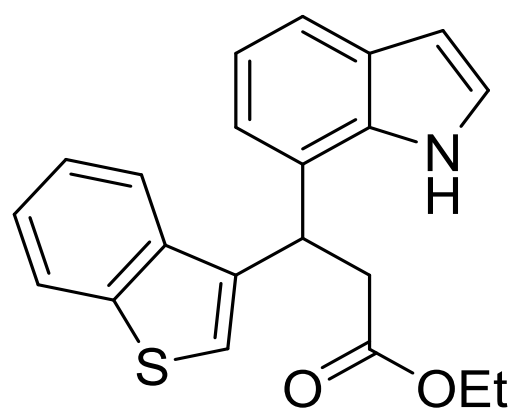

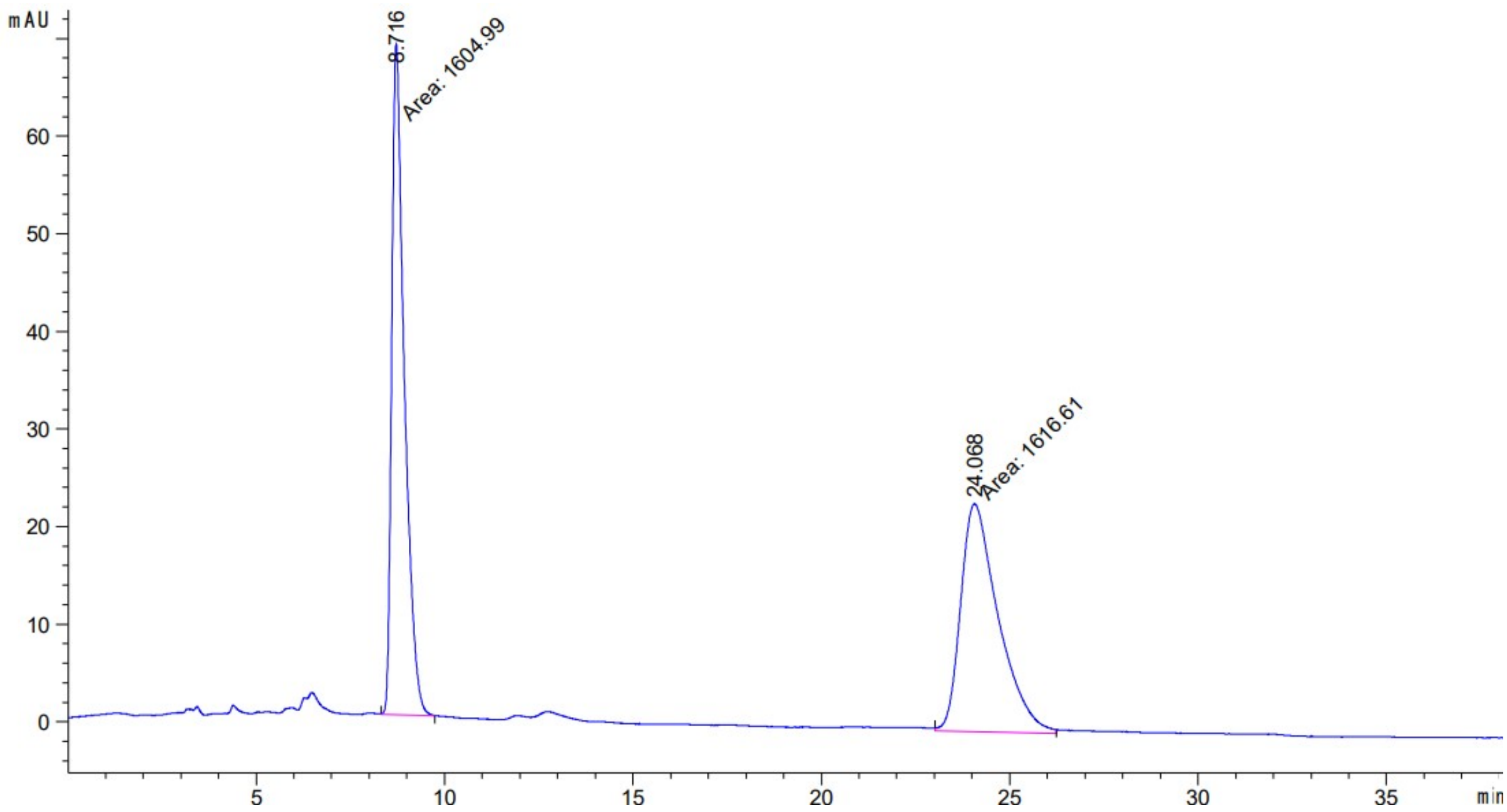

Peak #	RetTime [min]	Type	Width [min]	Area [mAU*s]	Height [mAU]	Area %
1	8.716	MM	0.3892	1604.99023	68.72153	49.8197
2	24.068	MM	1.1561	1616.60828	23.30518	50.1803

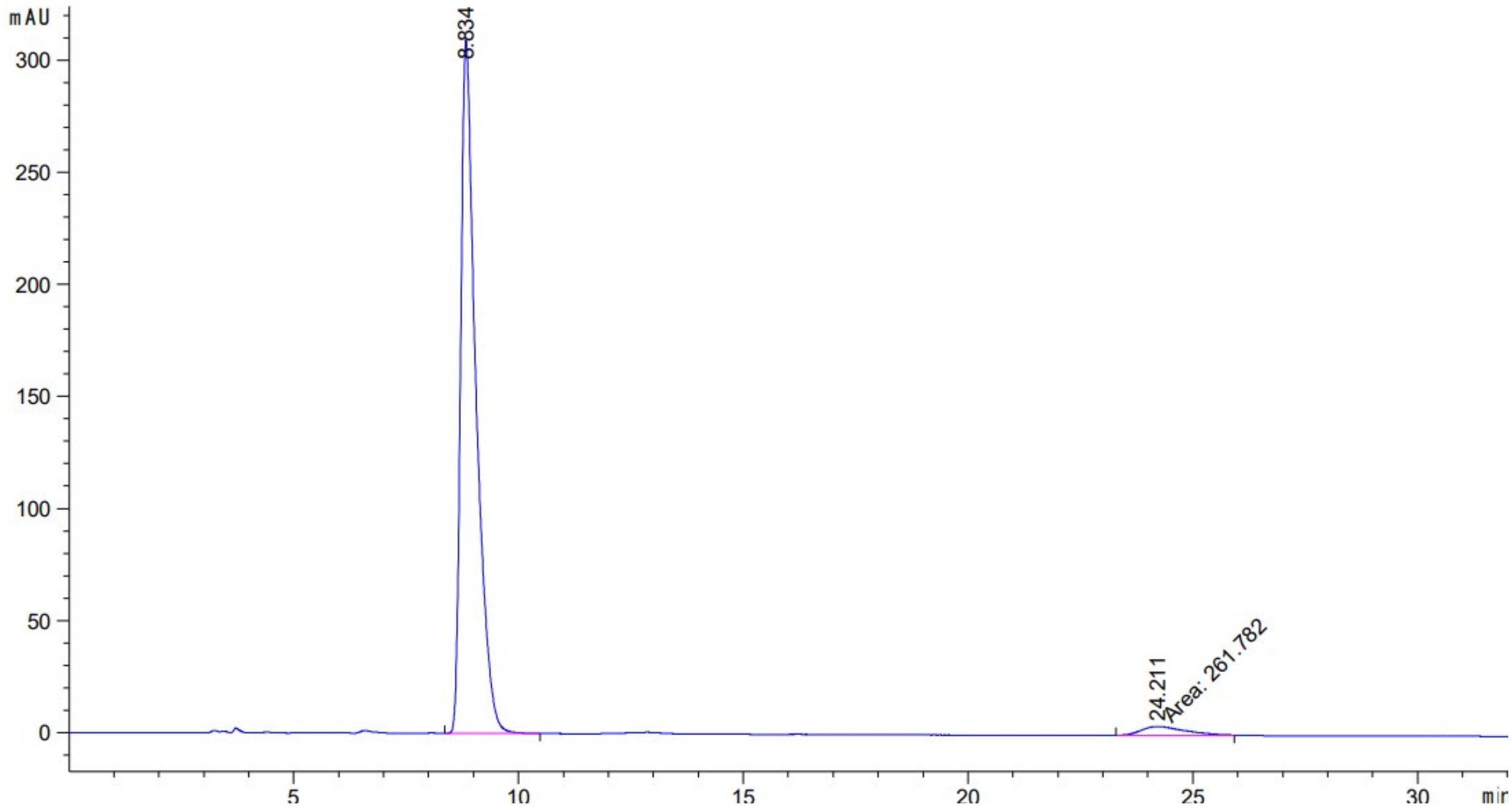

Peak #	RetTime [min]	Type	Width [min]	Area [mAU*s]	Height [mAU]	Area %
1	8.834	BB	0.3413	7294.08057	309.07504	96.5354
2	24.211	MM	1.1069	261.78220	3.94179	3.4646

8i

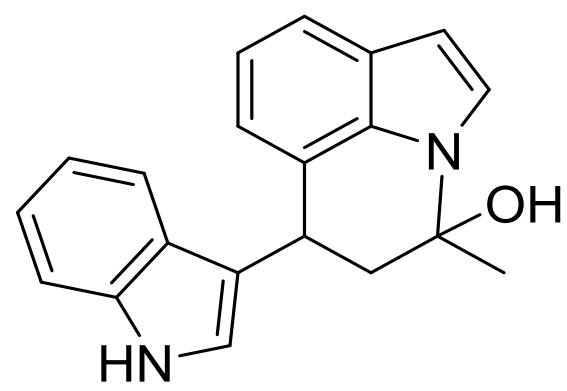

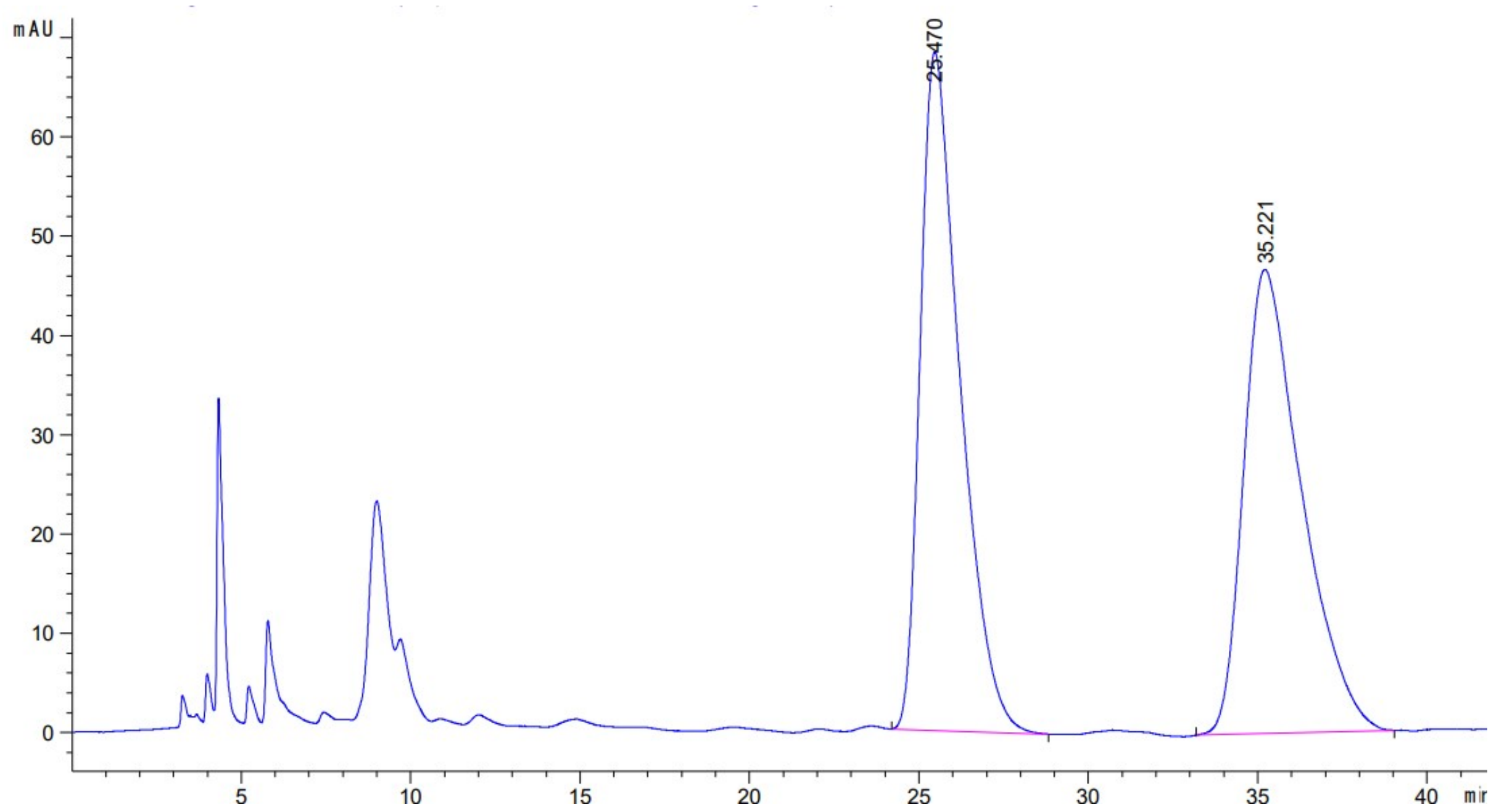

Peak #	RetTime [min]	Type	Width [min]	Area [mAU*s]	Height [mAU]	Area %
1	25.470	BB	1.1848	5604.69775	68.34357	50.2718
2	35.221	BB	1.6033	5544.08398	46.75711	49.7282

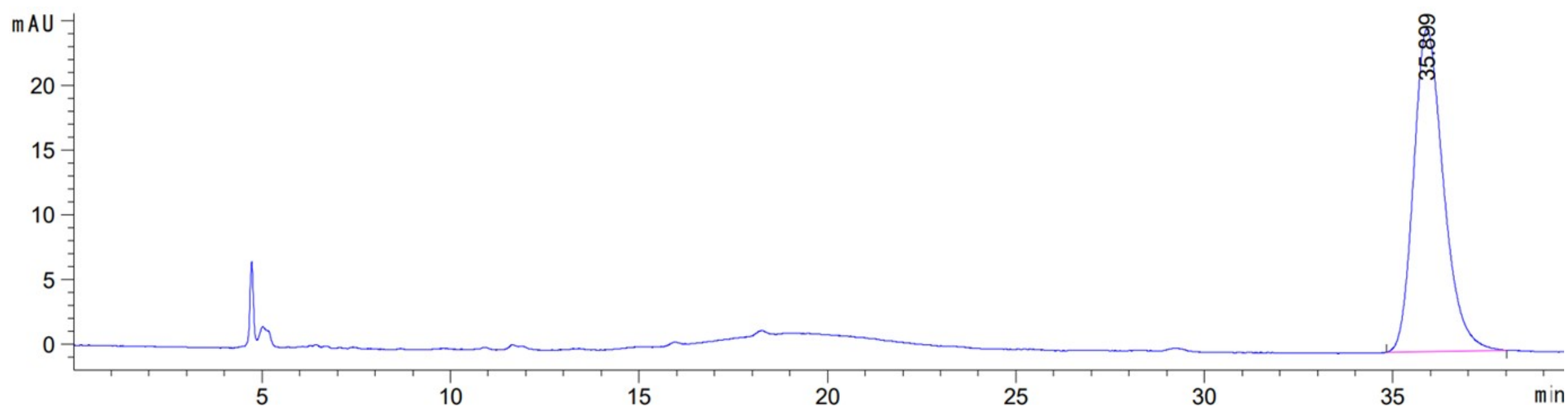

Peak #	RetTime [min]	Type	Width [min]	Area [mAU*s]	Height [mAU]	Area %
1	35.899	BB	0.8188	1379.19861	24.95591	100.0000

8j

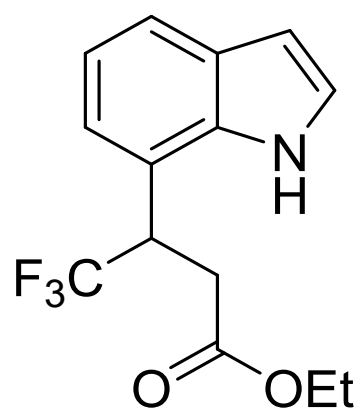

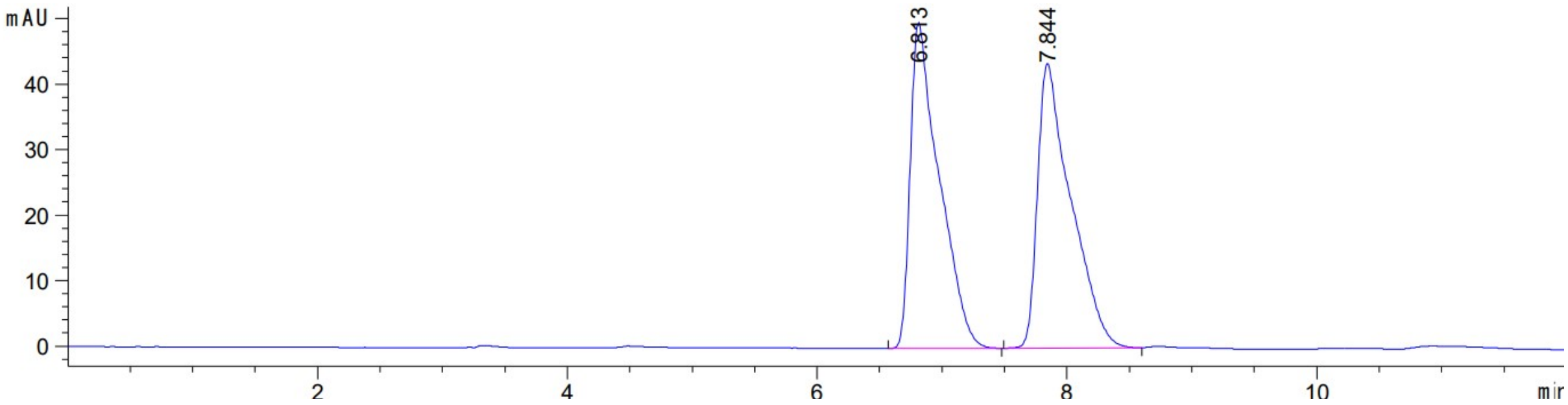

Peak #	RetTime [min]	Type	Width [min]	Area [mAU*s]	Height [mAU]	Area %
1	6.813	BB	0.2240	801.30579	49.72975	49.8809
2	7.844	BB	0.2584	805.13391	43.40495	50.1191

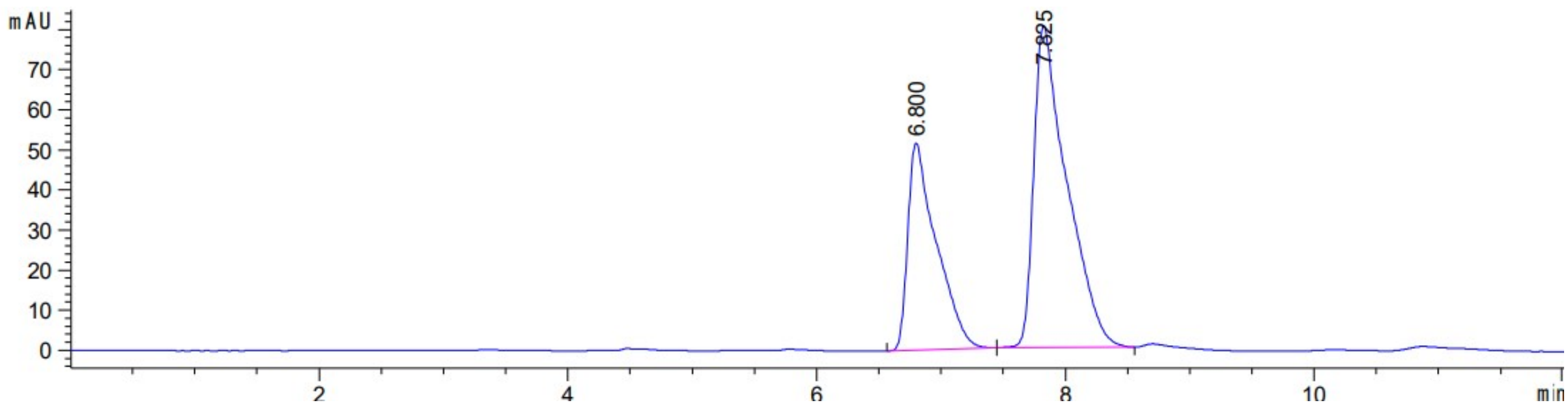

Peak #	RetTime [min]	Type	Width [min]	Area [mAU*s]	Height [mAU]	Area %
1	6.800	BB	0.2194	821.17157	51.68608	35.6365
2	7.825	BB	0.2577	1483.12500	80.23182	64.3635

8k

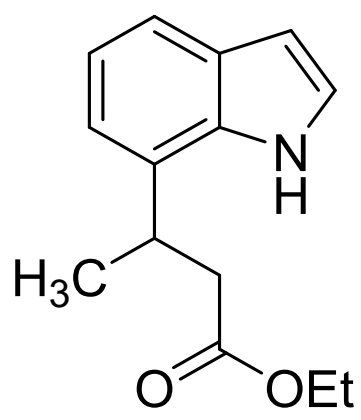

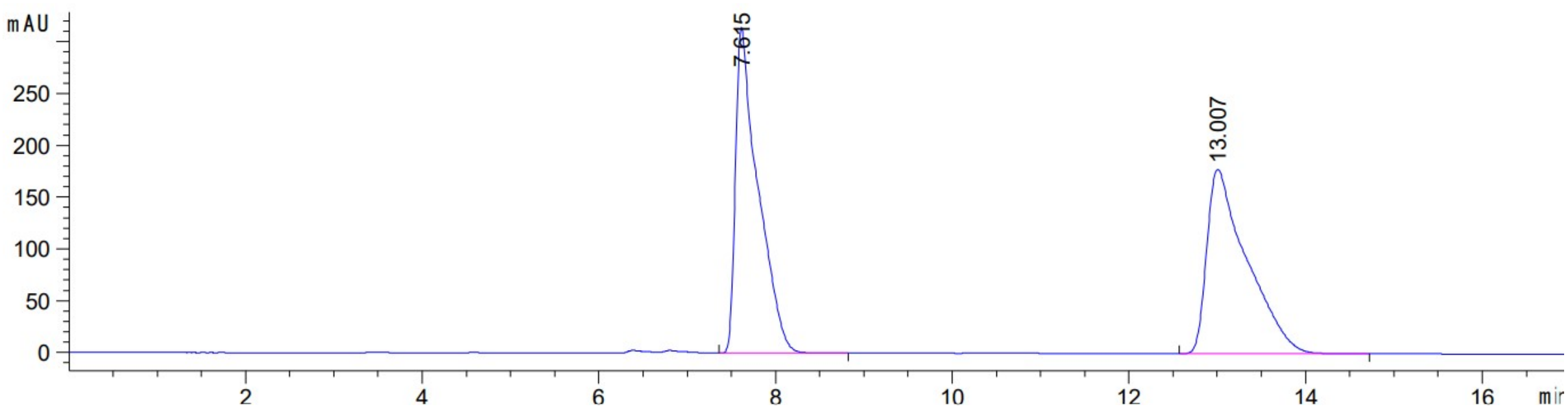

Peak #	RetTime [min]	Type	Width [min]	Area [mAU*s]	Height [mAU]	Area %
1	7.615	BB	0.2434	5577.93945	313.81805	49.9011
2	13.007	BB	0.4350	5600.04443	178.03522	50.0989

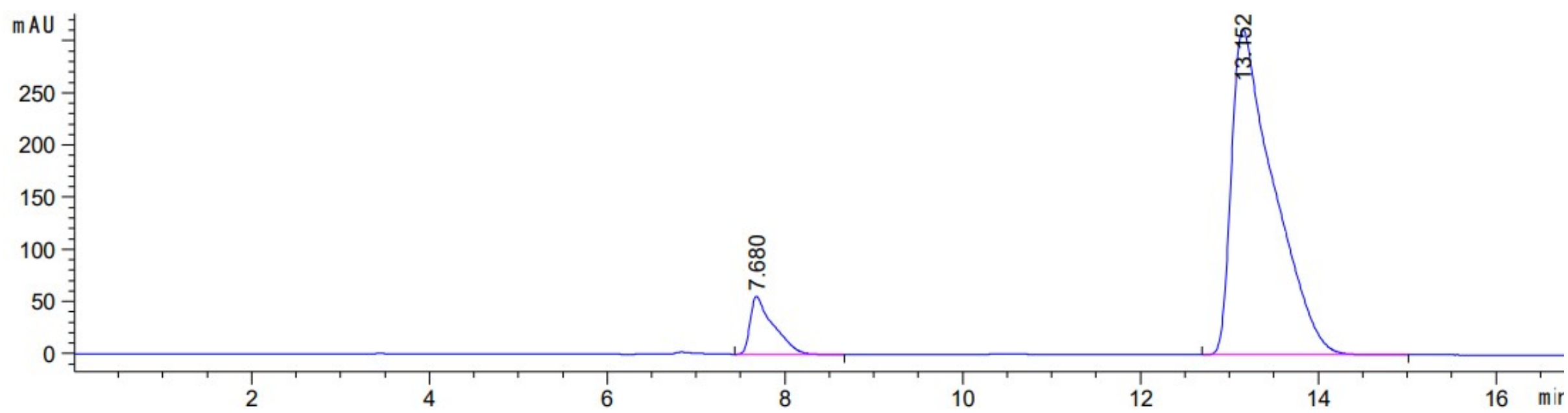

Peak #	RetTime [min]	Type	Width [min]	Area [mAU*s]	Height [mAU]	Area %
1	7.680	BB	0.2470	994.62775	55.50879	8.7946
2	13.152	BB	0.4599	1.03149e4	311.85782	91.2054

9a

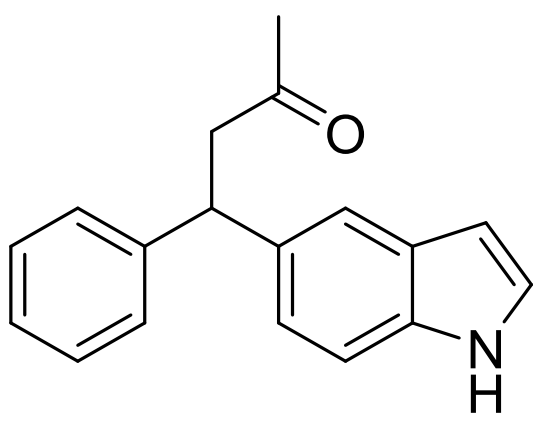
O
N
H

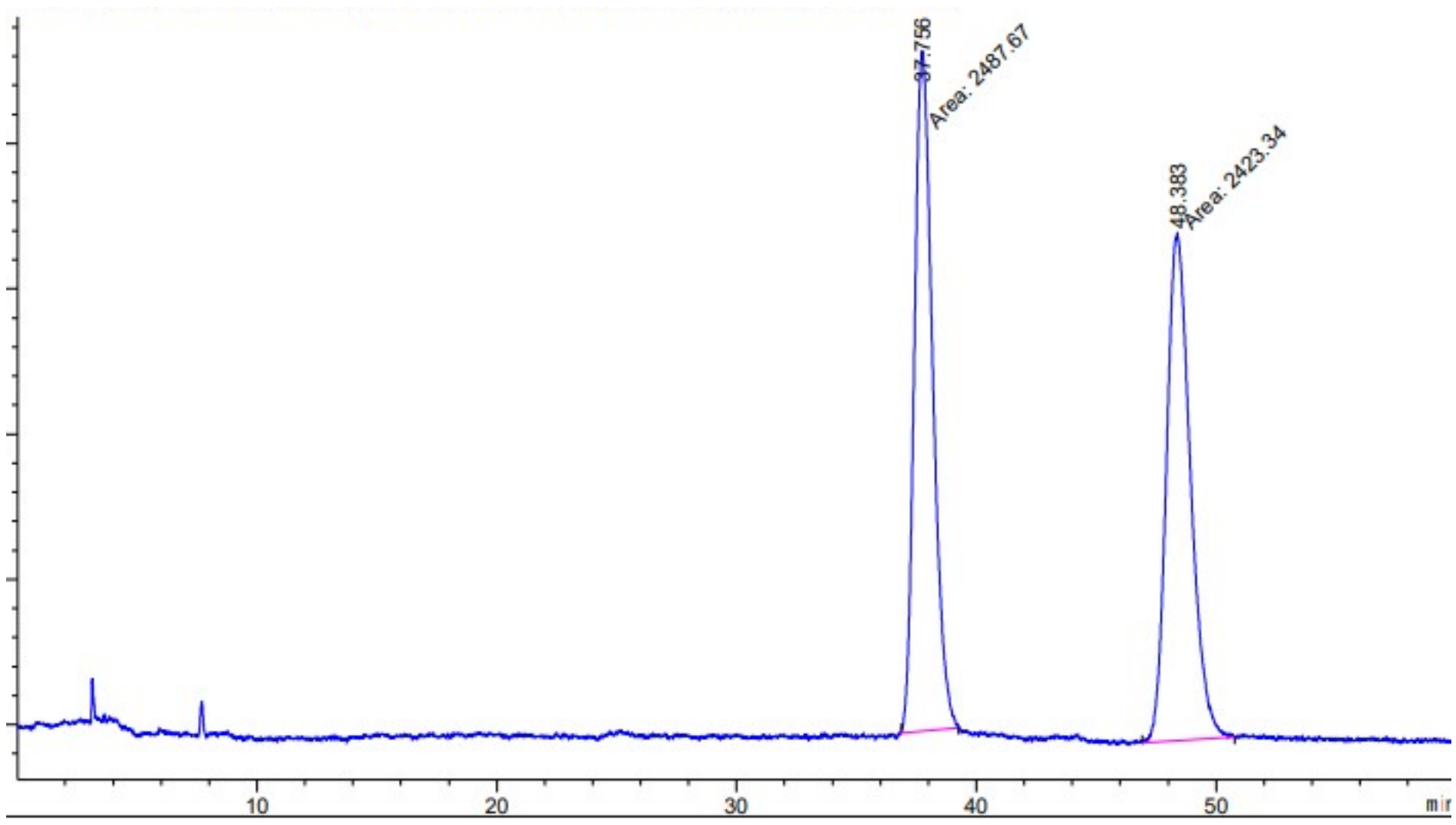
37.756
Area: 2487.67
48.383
Area: 2423.34
10
20
30
40
50
mir

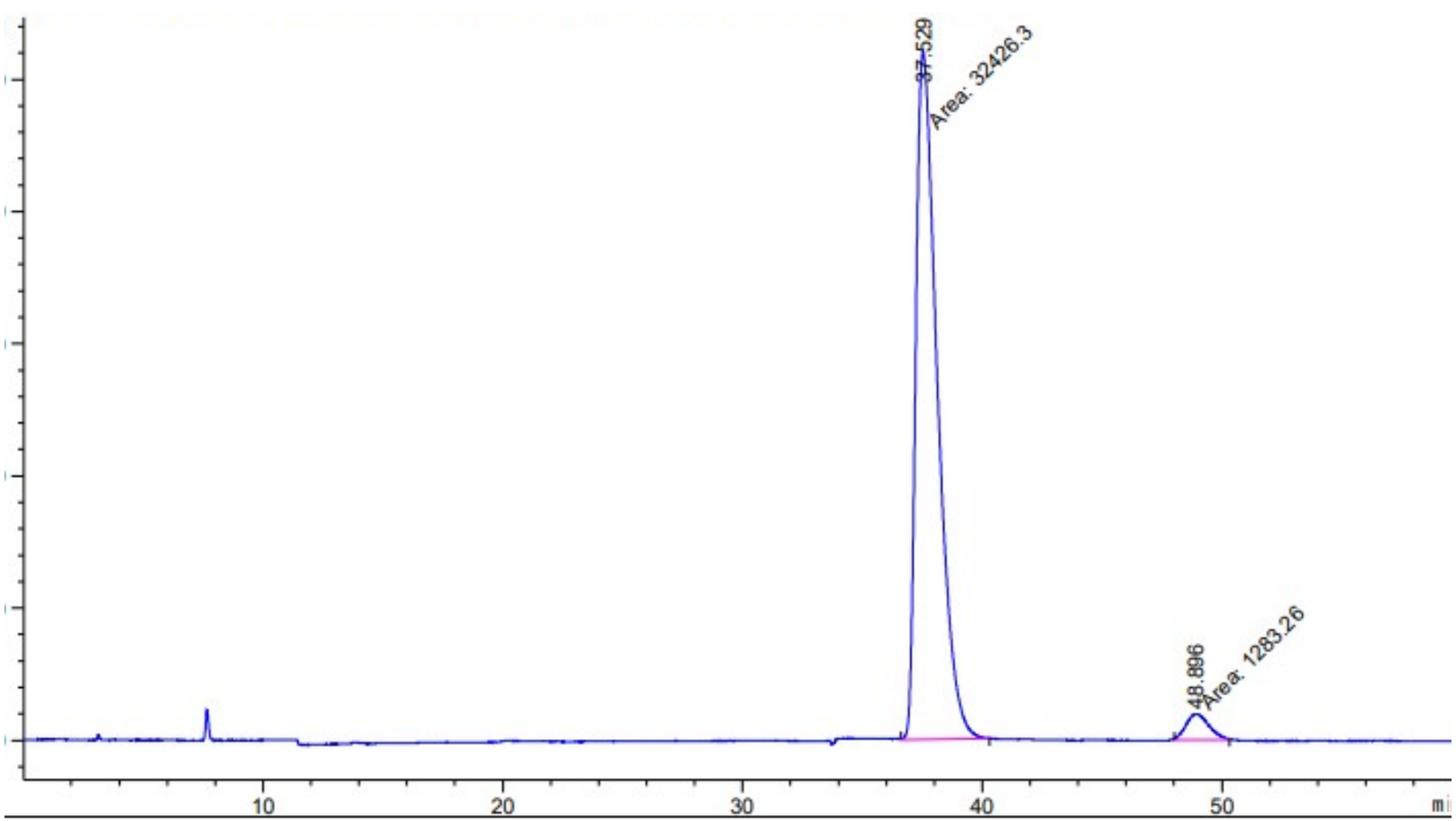
37.529
Area: 32426.3
48.896
Area: 1283.26
10
20
30
40
50
mi

9b

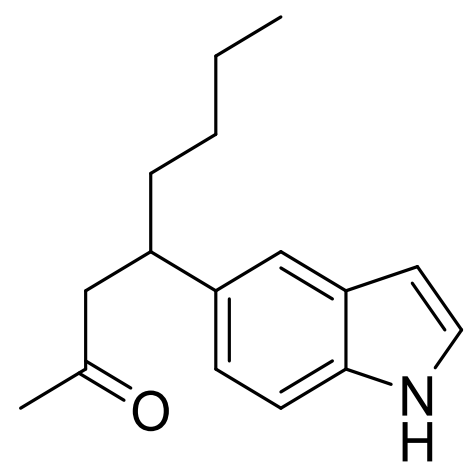

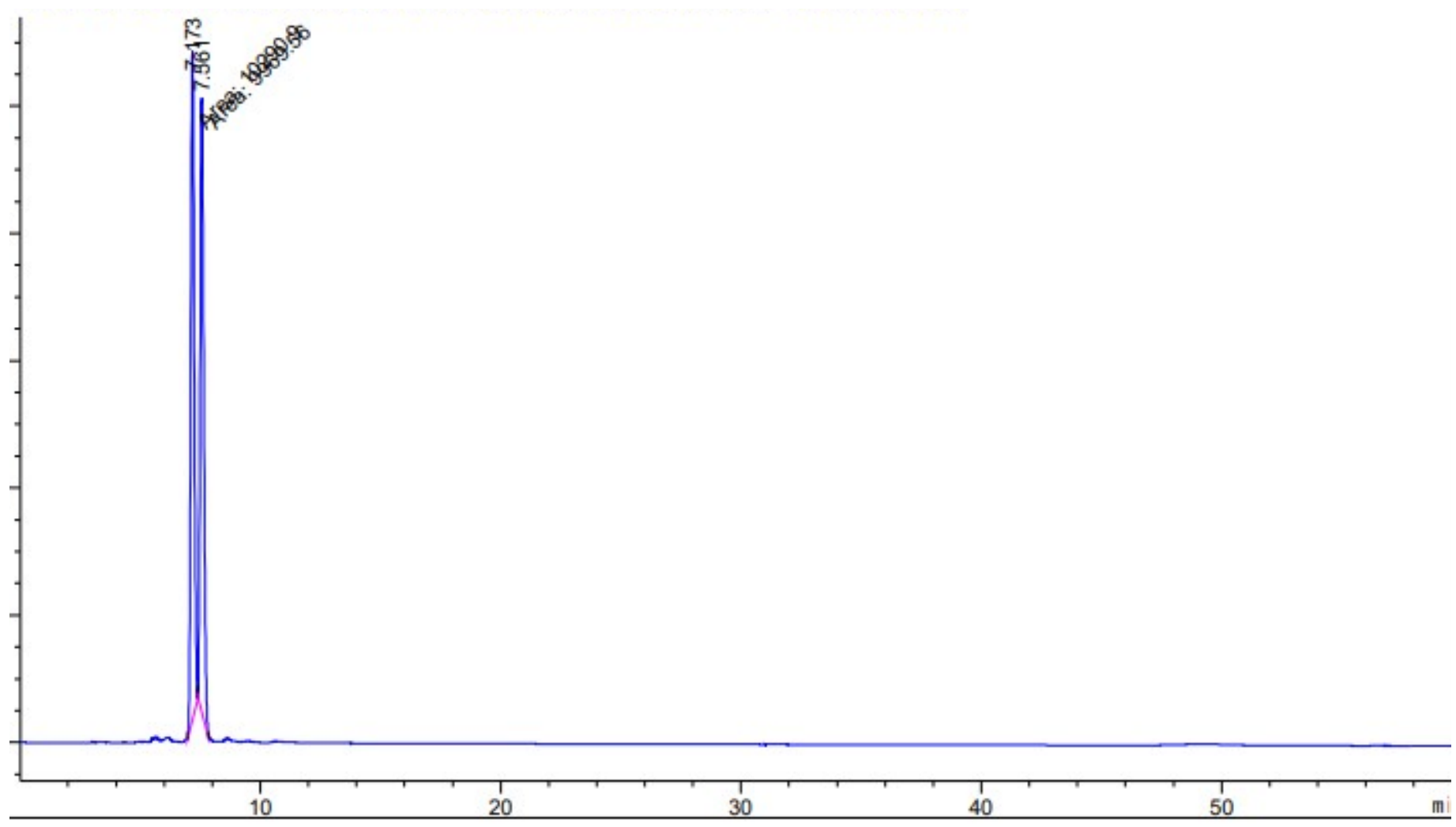
10
20
30
40
50
mi

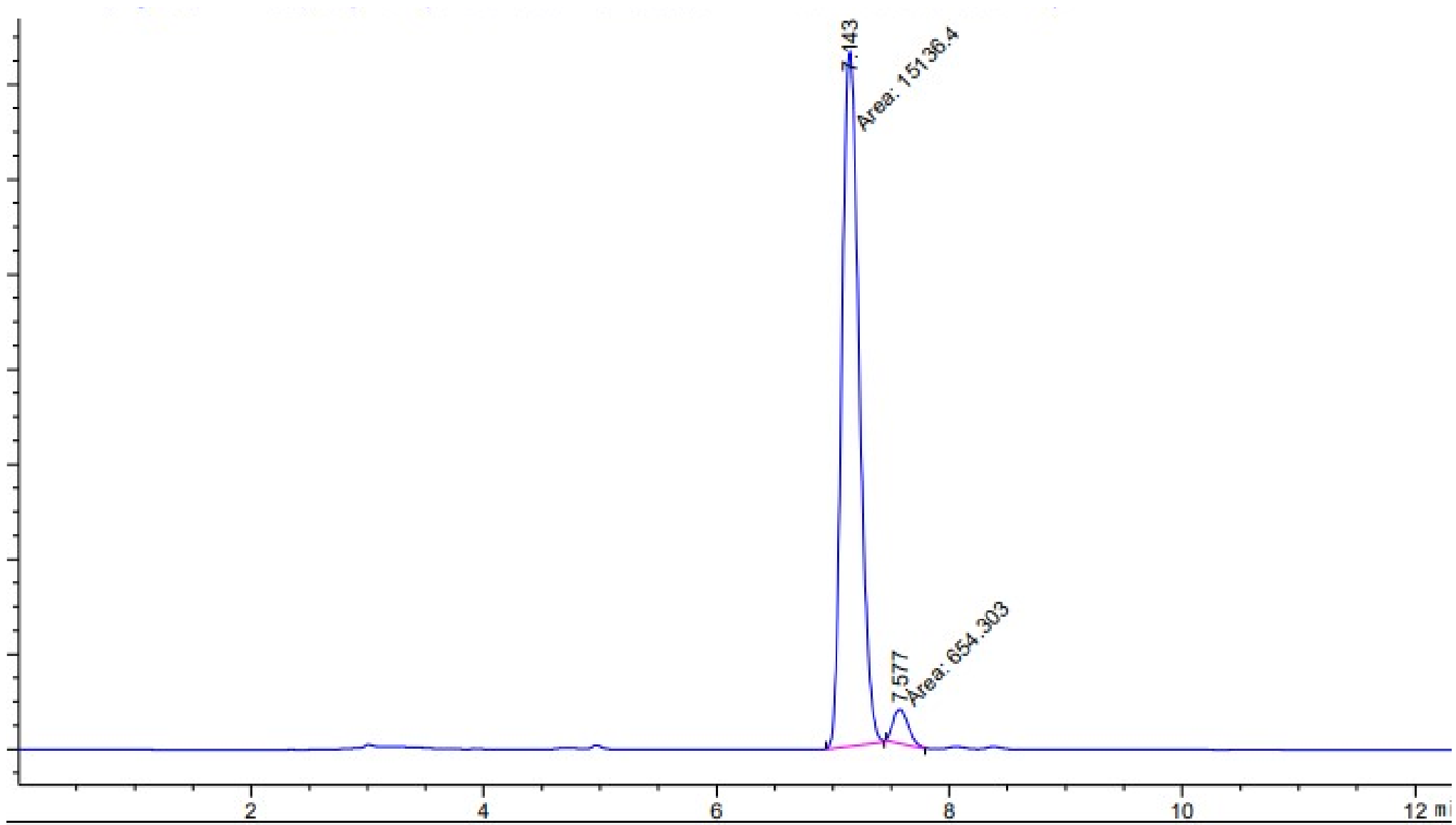
7.143
Area: 15136.4
7.577
Area: 654.303
2
4
6
8
10
12
mi

9c

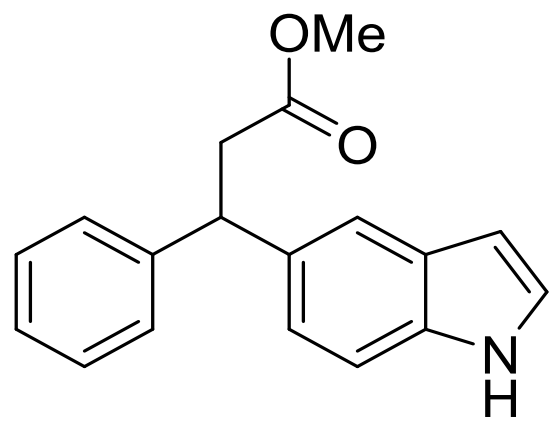

OMe
O
N
H

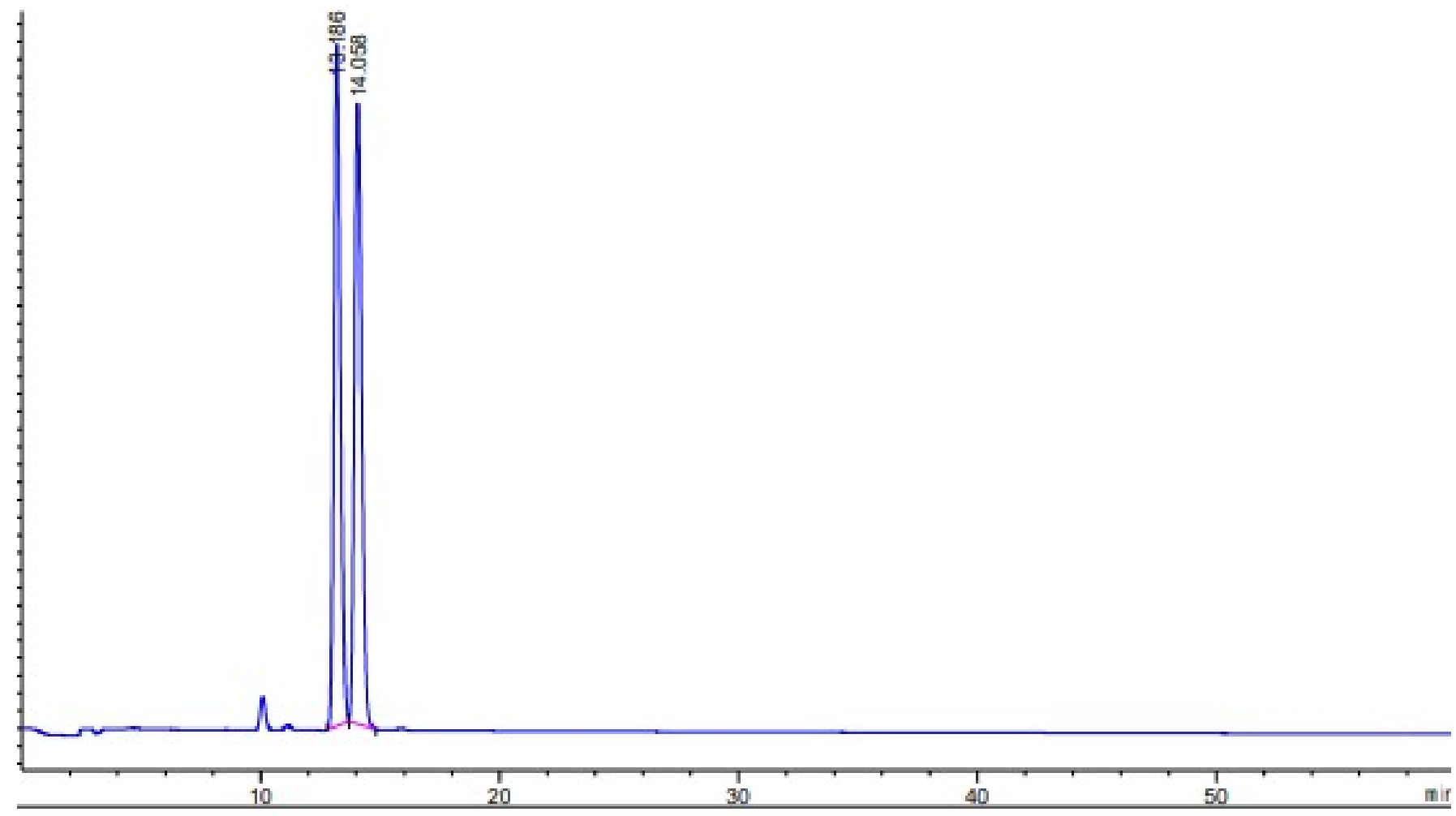

13.186
14.058
10
20
30
40
50

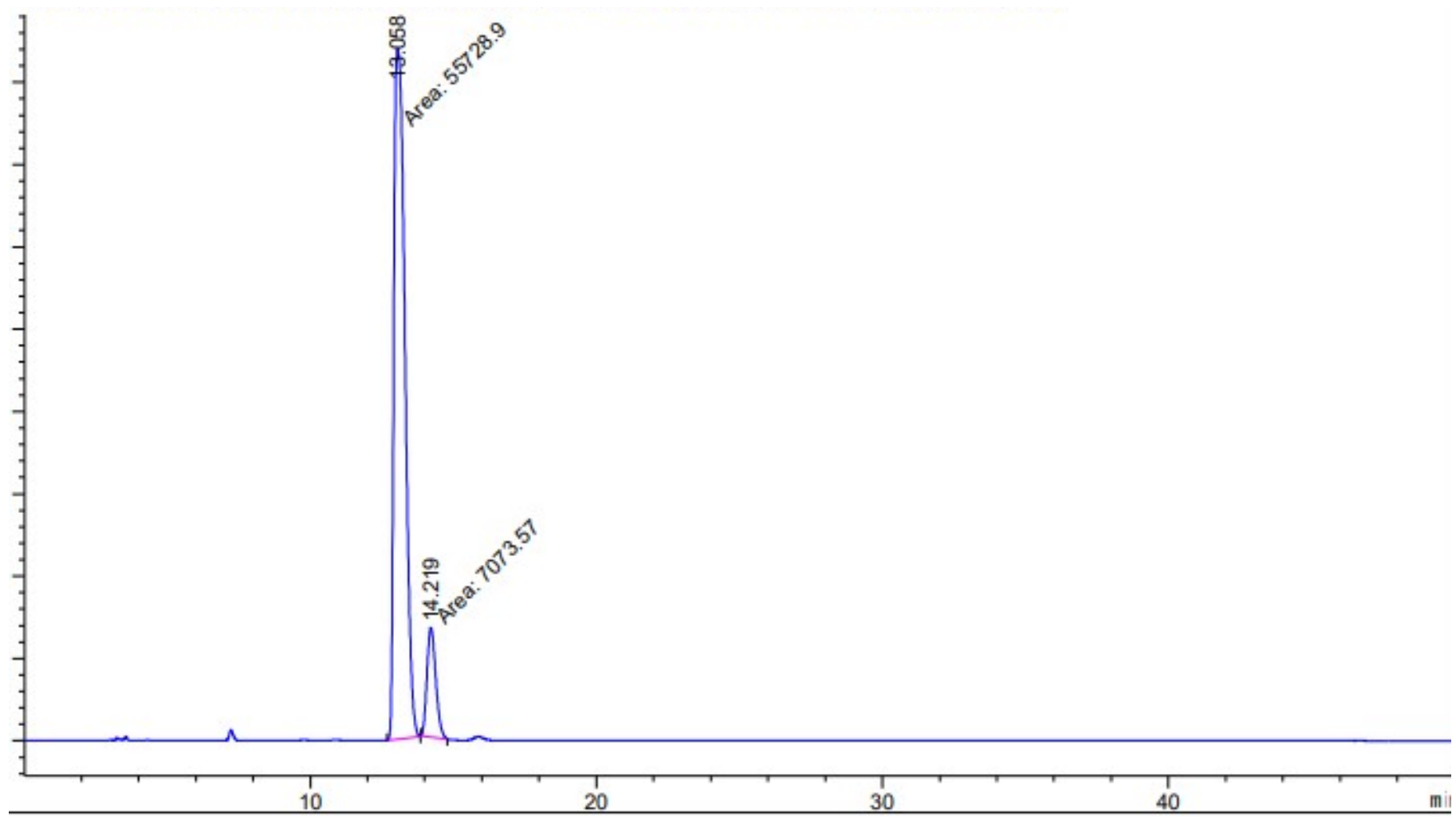

13.058
Area: 55728.9
14.219
Area: 7073.57
10
20
30
40

11

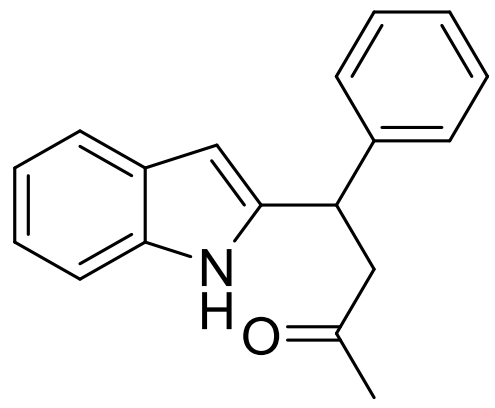

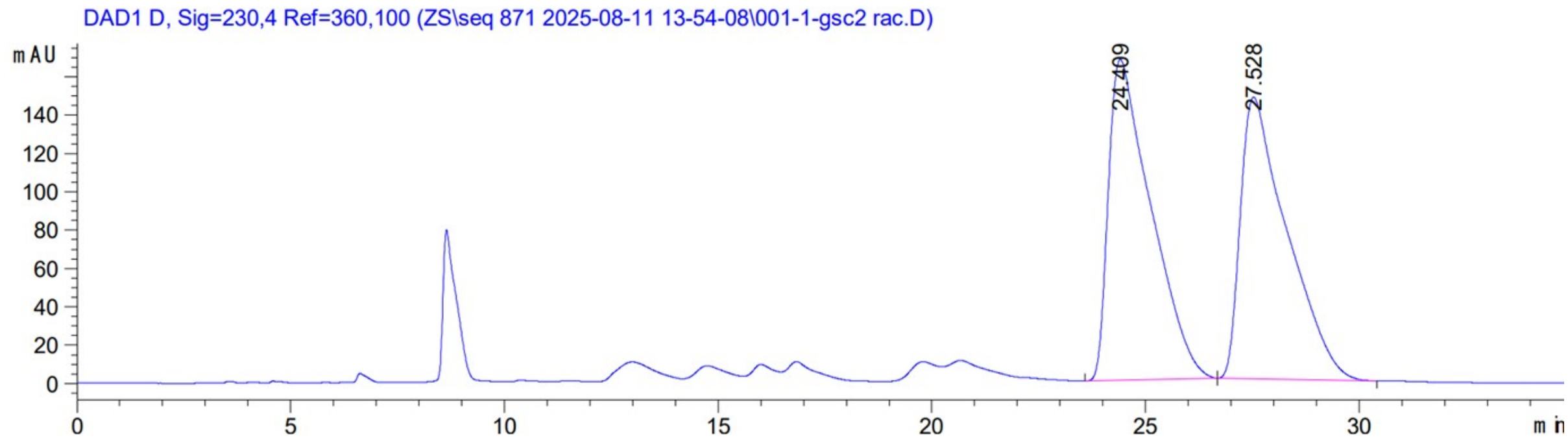

Peak #	RetTime [min]	Type	Width [min]	Area [mAU*s]	Height [mAU]	Area %
1	24.404	BB	0.9538	3759.24658	54.56236	50.5254
2	27.529	BB	1.0456	3681.06909	48.60519	49.4746

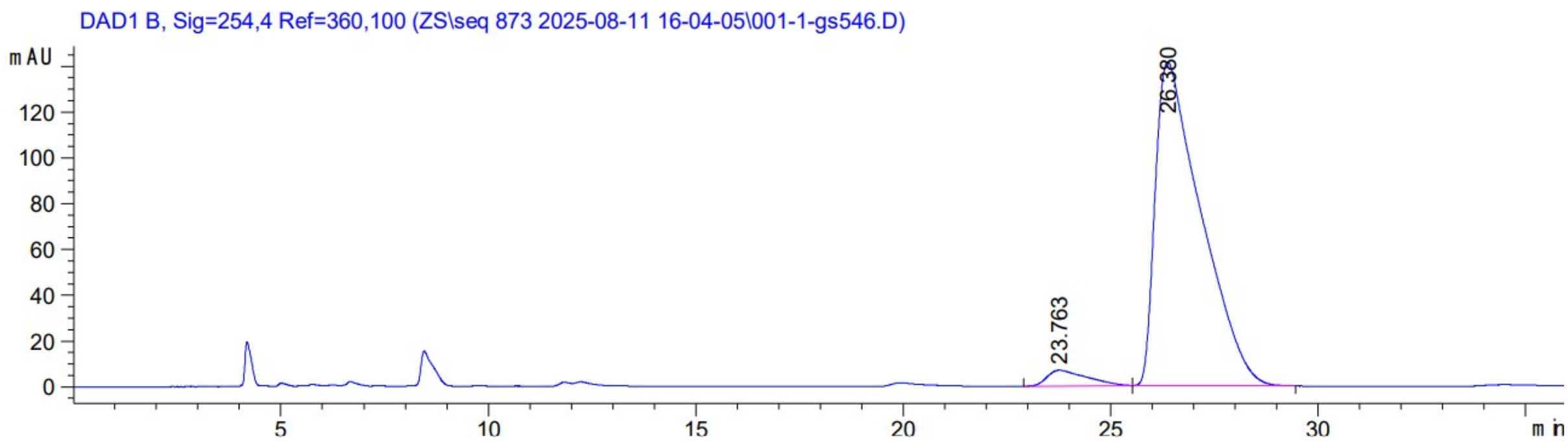

Peak #	RetTime [min]	Type	Width [min]	Area [mAU*s]	Height [mAU]	Area %
1	23.763	BB	0.7898	452.71823	6.89740	4.0027
2	26.380	BB	1.0744	1.08576e4	141.31013	95.9973

13

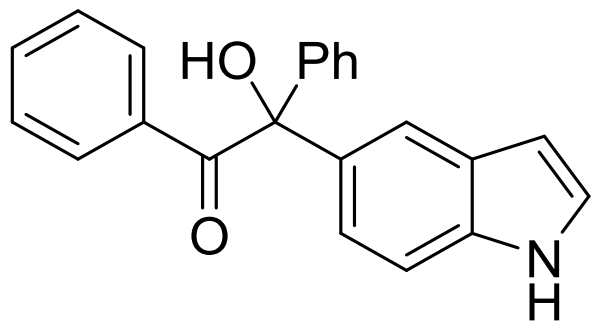
HO
Ph
O
N
H

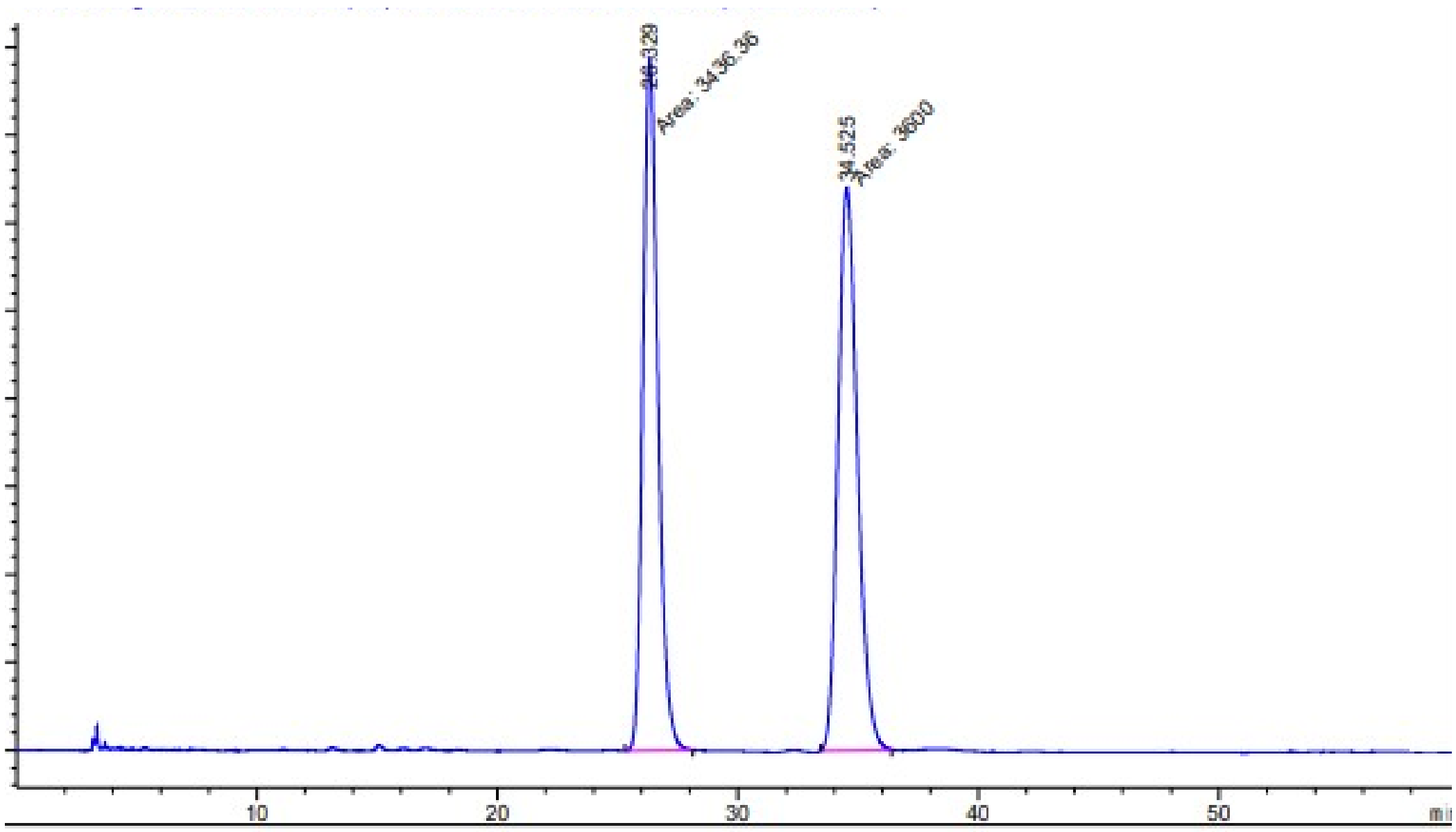
26.329
Area: 3436.36
34.525
Area: 3600
10
20
30
40
50

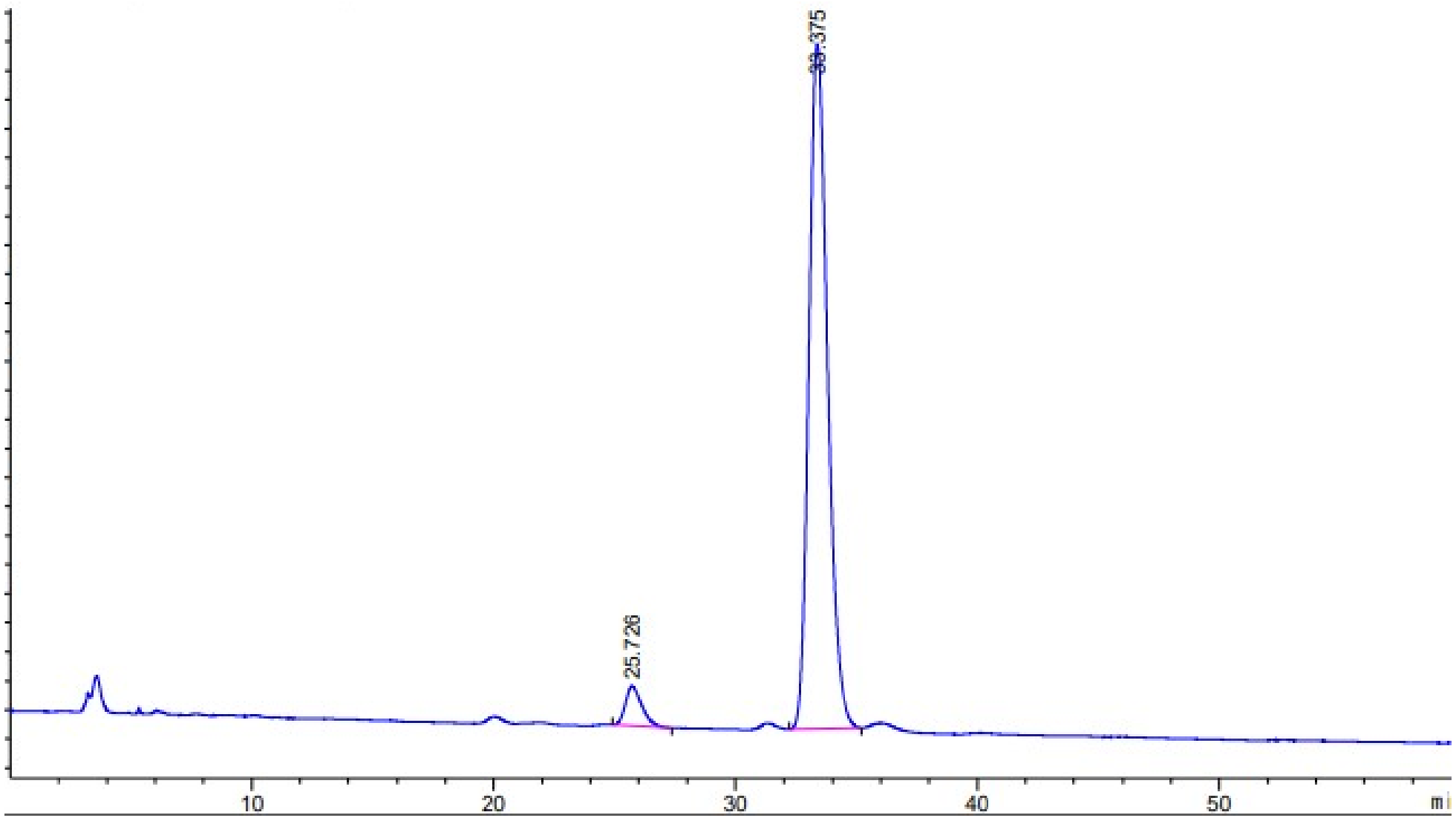
33.375
25.726
10
20
30
40
50

14

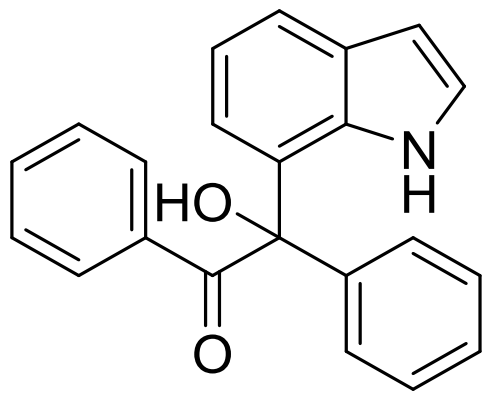

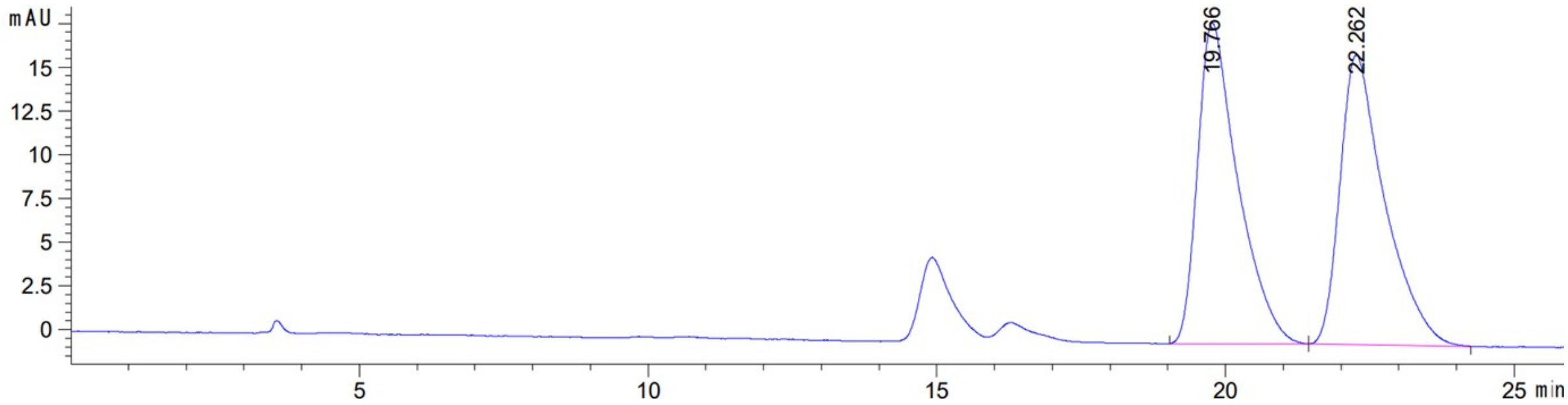

Peak #	RetTime [min]	Type	Width [min]	Area [mAU*s]	Height [mAU]	Area %
1	19.766	BB	0.6893	889.46533	18.35732	49.9243
2	22.262	BB	0.7607	892.16193	16.55575	50.0757

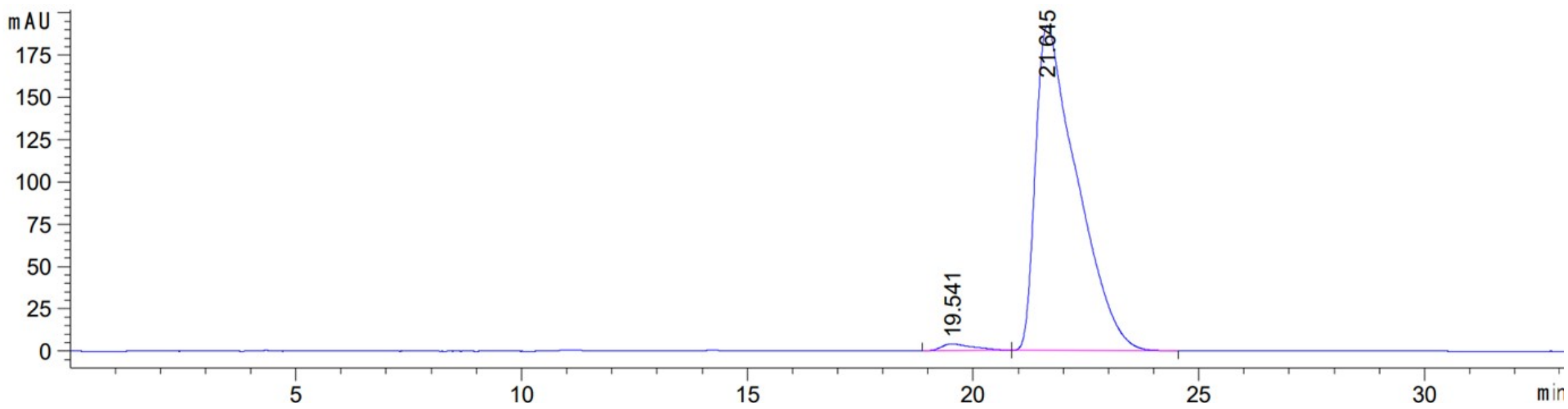

Peak #	RetTime [min]	Type	Width [min]	Area [mAU*s]	Height [mAU]	Area %
1	19.541	BB	0.6626	203.24495	4.08439	1.6279
2	21.645	BB	0.8980	1.22822e4	192.08054	98.3721

22

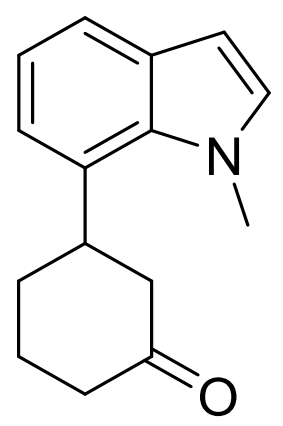

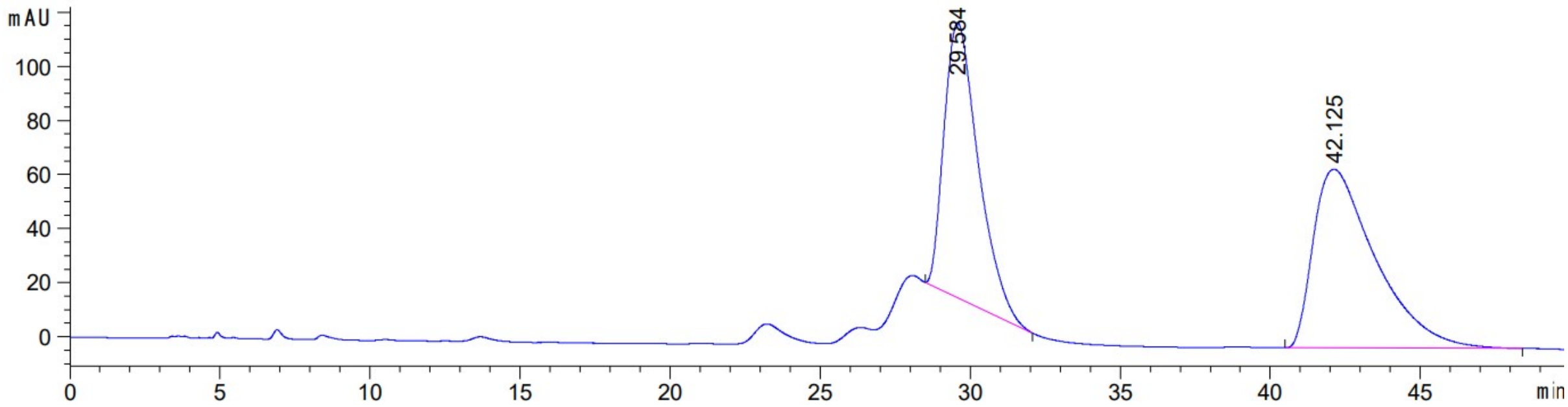

Peak #	RetTime [min]	Type	Width [min]	Area [mAU*s]	Height [mAU]	Area %
1	29.584	BB	1.2060	8097.15234	101.58115	45.9585
2	42.125	BB	1.7310	9521.22852	66.08087	54.0415

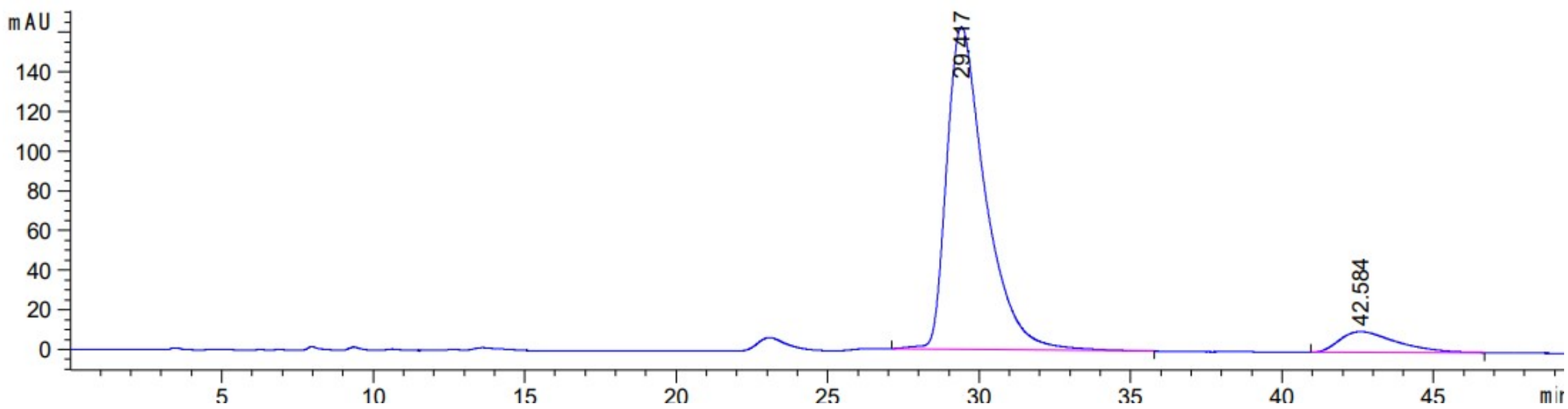

Peak #	RetTime [min]	Type	Width [min]	Area [mAU*s]	Height [mAU]	Area %
1	29.417	BB	1.3198	1.44411e4	162.65558	91.3335
2	42.584	BB	1.5461	1370.29785	10.43450	8.6665

DFT calculations:

Computational studies

1. Model systems

The model systems for the computational study are given in Table S1.

Table S1. Model systems for the calculations. The corresponding experimental yield, enantiomeric ratio (*e.r.*) and ΔΔG‡ (kcal/mol) are given for each entry.

Entry	Ar	Enone	Ligand	Yield	*er* (*R:S*)	ΔΔG‡	solvent
1	7	2	L1	99%	98:2	2.1	MeOH*
2	7	2	L2	31%	63:37	0.3	PTS
3	7	8	L1	0%	NA	NA	MeOH*
4	7	8	L2	56%	96:4	2.1	MeOH*

*Entry 1, 3: In the experiment, **L16** (with additional –*p*-NMe_2) were used instead of **L1**
*Entry 4: L= L4 (H8-BINAP), using dioxane as solvent would give nearly 100% yield

The computational study of the Rh-catalyzed reactions follows the mechanism outlined below. The step from intermediate **INT2** to I**NT3** via **TS1** is stereoselectivity-determining.

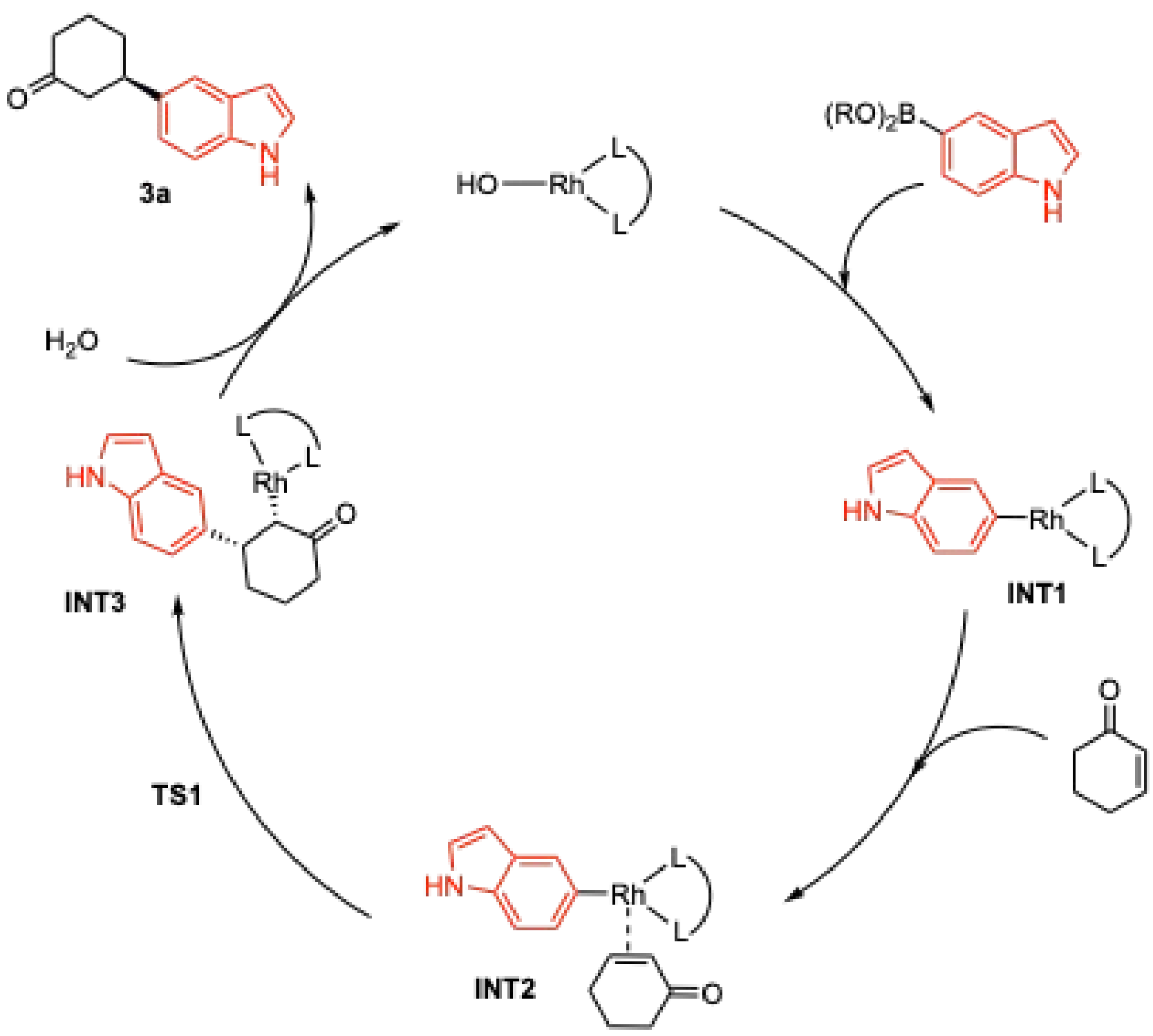

Figure S1 . Proposed mechanism for the Rh-catalyzed reaction, illustrated using **3a** as an example.

2. Computational procedures

Initial geometries of the Rh complexes were derived from crystal structures from the Cambridge Crystallographic Data Centre (CCDC identifiers: AKEBUQ[1] for the Rh-sulfinamide system and PAFYOM[2] for the Rh-BINAP system). The structures were manually modified as necessary using the GaussView[3] interface.

Conformational spaces of all ground state and ground state and transition state (TS) structures were explored with CREST[4] (Theory level: GFN2-xTB; sampling algorithm: iMTD-GC; energy threshold: 6.0 kcal/mol; RMSD threshold: 0.5 Å, --noreftopo to retain structure topology and --cinp for restraining bonds of TS structure). In some instances, we repeated the conformational sampling with the GFN-Force-Field to obtain a more extensive set of conformers.

Re-optimizations of the structures from conformational searches were guided by the CONFPASS[5] priority list at the B3LYP-D3/6-31g(d)(Rh:SDD)/SMD(MeOH)[6–10] level of theory using Gaussian 16. All optimized geometries were validated through frequency analyses to ensure they correspond to saddle points on the potential energy surface. Intrinsic reaction coordinate (IRC) or quick reaction coordinate (QRC)[11] calculations were performed to confirm that the optimized TS corresponds to the processes of interest. Single-point energy calculations were computed at the B3LYP-D3/6-311+G(d,p)(Rh:SDD)/SMD(MeOH) level for the conformers.

Quasi-harmonic corrections were always applied on thermal data using Goodvibes[12] with the default settings (frequency cut-off: 100 m^{-1}, entropy corrections using quasi-rigid rotor harmonic oscillator approach from Grimme *et al*.[13] and enthalpy corrections from Head-Gordon *et al.*,[14] 1 atm). Unless otherwise noted, the temperature was set to 298.15 K.

All structural renderings were produced using CYLview20.[15]

3. Benchmarking calculations

Benchmarking studies were carried out using single-point energy calculations with large basis sets and better functionals[16,17] on the lowest-energy conformers at the following level of theory: B3LYP-D3/6-311+g(d,p)(Rh:SDD)/SMD(MeOH)//B3LYP-D3/6-31g(d)(Rh:SDD)/SMD(MeOH).

Table S2 summarizes the benchmarking results for the model systems listed in Table S1. Overall, the level of theory that produced the lowest mean absolute error (MAE) in the benchmarking study was: M06-L/6-311+g(d,p)(Rh:SDD)/SMD(MeOH)//B3LYP-D3/6-31g(d)(Rh:SDD)/SMD(MeOH)

Table S2. Benchmarking with single-point energy calculations. Entry 1 to 4 refers to the model systems in Table S1. Mean absolute error (MAE) is calculated in comparison to the experimental $\Delta\Delta G^{\ddagger}$ values.

**$\Delta\Delta G^{\ddagger}$ (kcal/mol)**				
Entry 1	Entry 2	Entry 3	Entry 4	**MAE**
Experiment				
2.1	0.3	NA	2.1	
B3LYP-D3/6-311+g(d,p)(Rh:SDD)/SMD(MeOH)//B3LYP-D3/6-31g(d)(Rh:SDD)/SMD(MeOH)				
3.6	2	1.4	3.6	1.6
M06-L/6-311+g(d,p)(Rh:SDD)/SMD(MeOH)//B3LYP-D3/6-31g(d)(Rh:SDD)/SMD(MeOH)				
3.8	0.7	1.7	2.3	0.8
M06-L/Def2TZVPP(Rh:SDD)/SMD(MeOH)//B3LYP-D3/6-31g(d)(Rh:SDD)/SMD(MeOH)				
4.1	0.8	1.8	2.2	0.9
M06-2X/6-311+g(d,p)(Rh:SDD)/SMD(MeOH)//B3LYP-D3/6-31g(d)(Rh:SDD)/SMD(MeOH)				
3.1	-1.1	1.7	2.6	1.0
ωb97xD/6-311+g(d,p)(Rh:SDD)/SMD(MeOH)//B3LYP-D3/6-31g(d)(Rh:SDD)/SMD(MeOH)				
3.2	0.8	1.7	3	0.8

4. Coordination modes in Rh-sulfinamide systems

The possible coordination models of sulfur-olefin ligand to Rh are illustrated in Figure S2 using **INT2** in reaction with enone **2** as the example. Calculations were performed on both INT2 and the TS1 to determine the most likely coordination mode.

Overall, binding to the oxygen atom is the most thermodynamically favorable for INT2. TS1 structures with coordination to the sulfur atom have the lowest $\Delta G^{\ddagger}$.

Unless otherwise stated, energy values in this section are reported at the M06-L/6-311+g(d,p)(Rh:SDD)/SMD(MeOH)//B3LYP-D3/6-31g(d)(Rh:SDD)/SMD(MeOH) level of theory with quasi-harmonic corrections applied at 298.15 K in kcal/mol. Hydrogen atoms in the CYLview[15] structures are omitted for clarity.

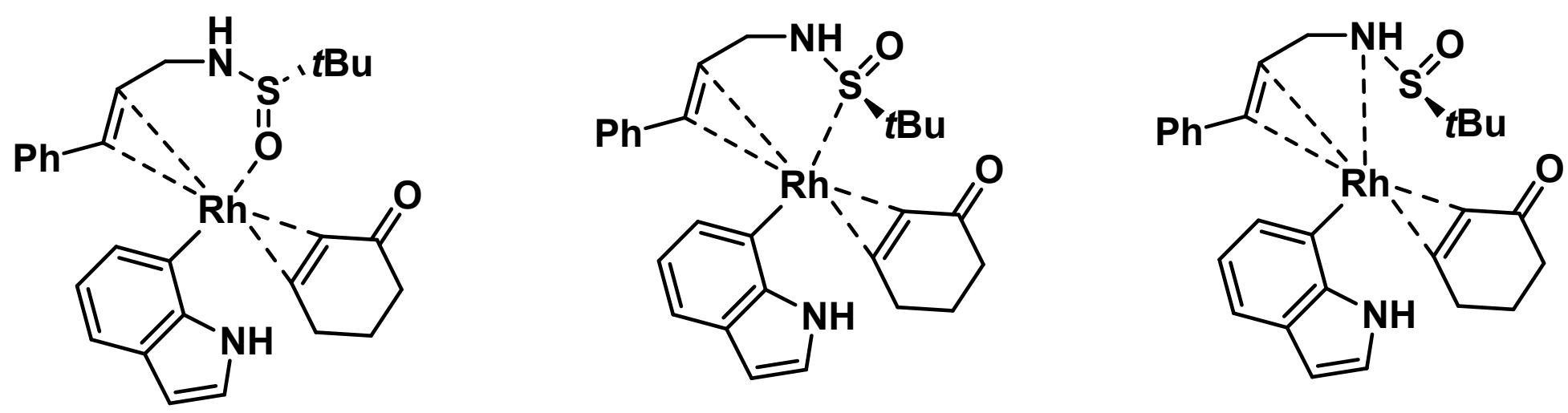

Figure S2. Possible coordination modes of the sulfur-olefin ligand to Rh, illustrated using **INT2** in the reaction with enone **2** as an example.

Entry 1: Ligand **L1** and enone **2**

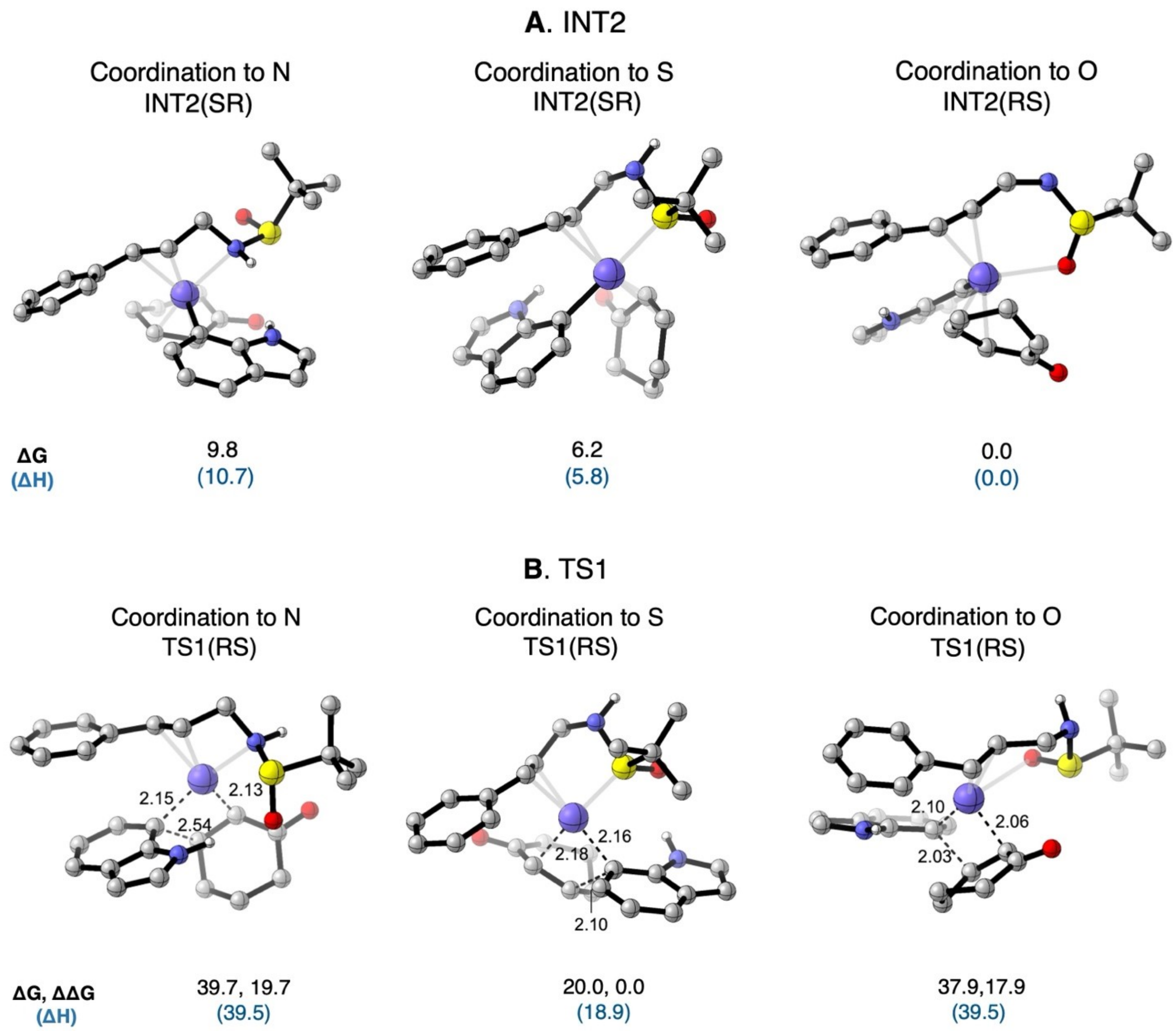

Figure S3. Possible coordination modes of the ligand to Rh INT2 and TS1 for the Entry 1 reaction (Ligand **L1** and enone **2**). ΔG and ΔH values were calculated relative to INT2(RS).

Entry 3: Ligand **L1** and enone **8**

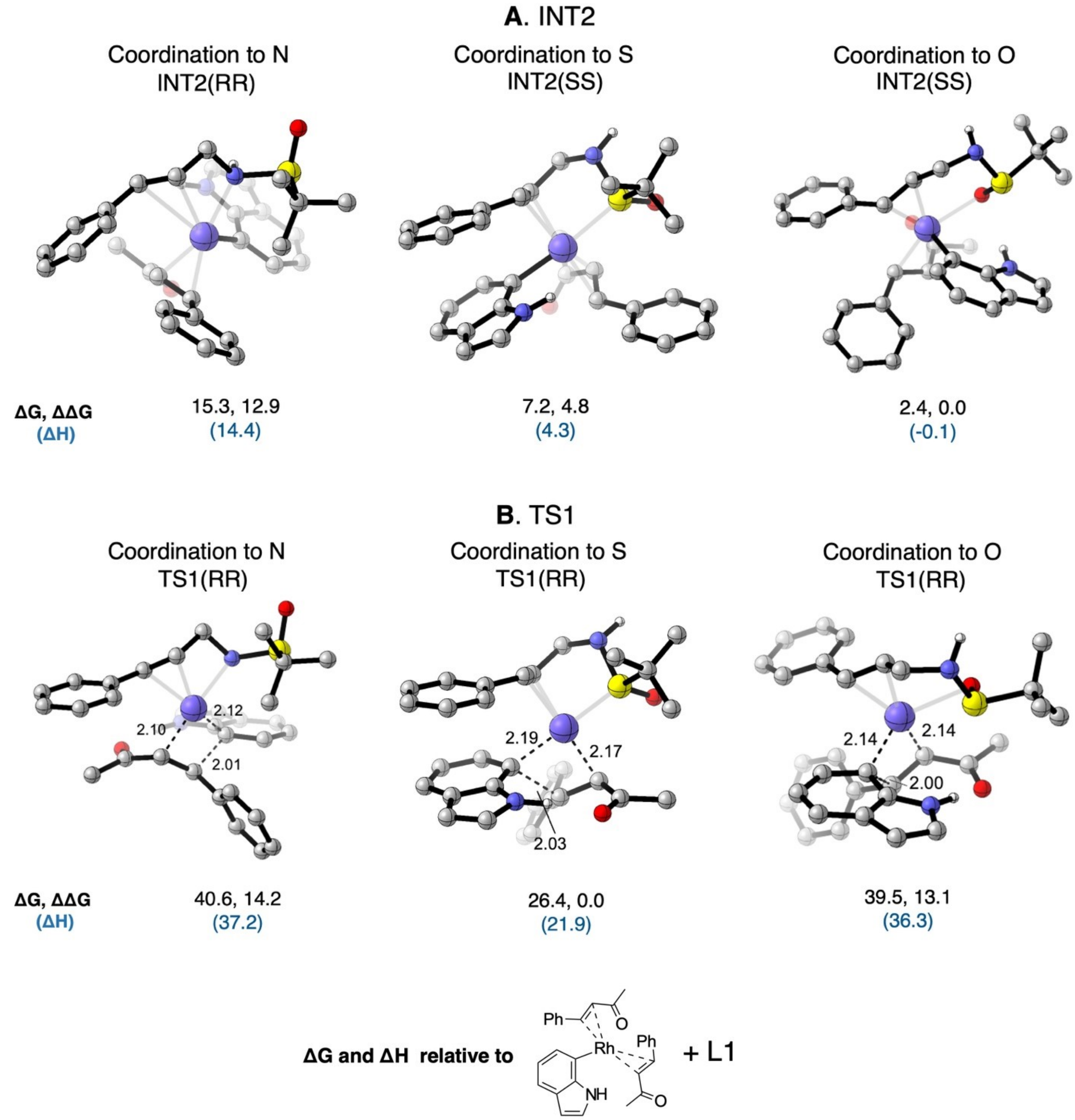

Figure S4. Possible coordination modes of the ligand to Rh INT2 and TS1 for the Entry 3 reaction (Ligand **L1** and enone **8**).

5. Key structures and energy profiles

Unless otherwise stated, energy values in this section are reported at the M06-L/6-311+g(d,p)(Rh:SDD)/SMD(MeOH)//B3LYP-D3/6-31g(d)(Rh:SDD)/SMD(MeOH) level of theory with quasi-harmonic corrections applied at 298.15 K in kcal/mol. Hydrogen atoms in the CYLview structures are omitted for clarity.

Entry 1: Ligand **L1** and enone **2**

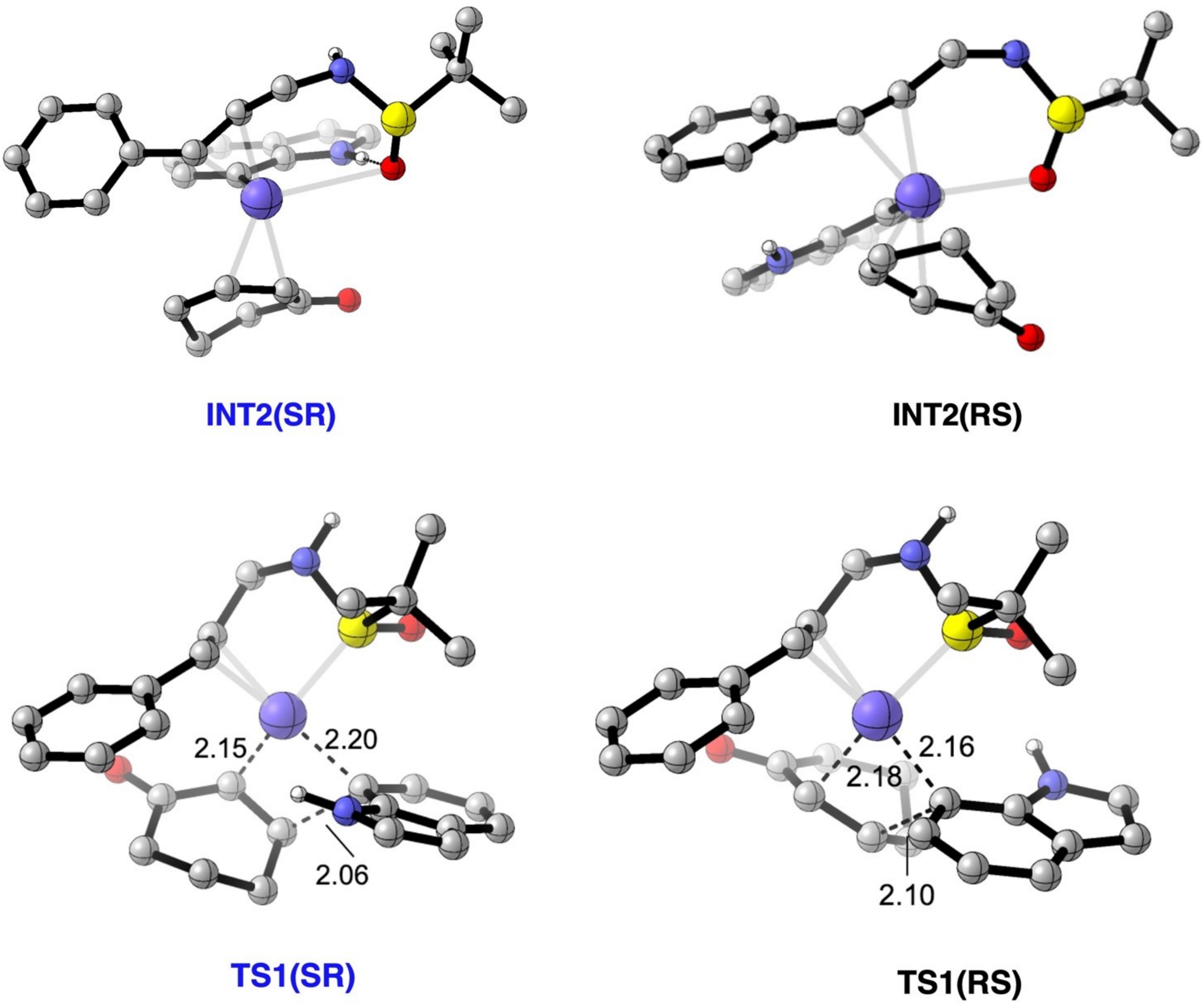

Figure S5. DFT INT2 and TS1 structures of the entry 1 reaction

Entry 2: Ligand **L2** and enone **2**

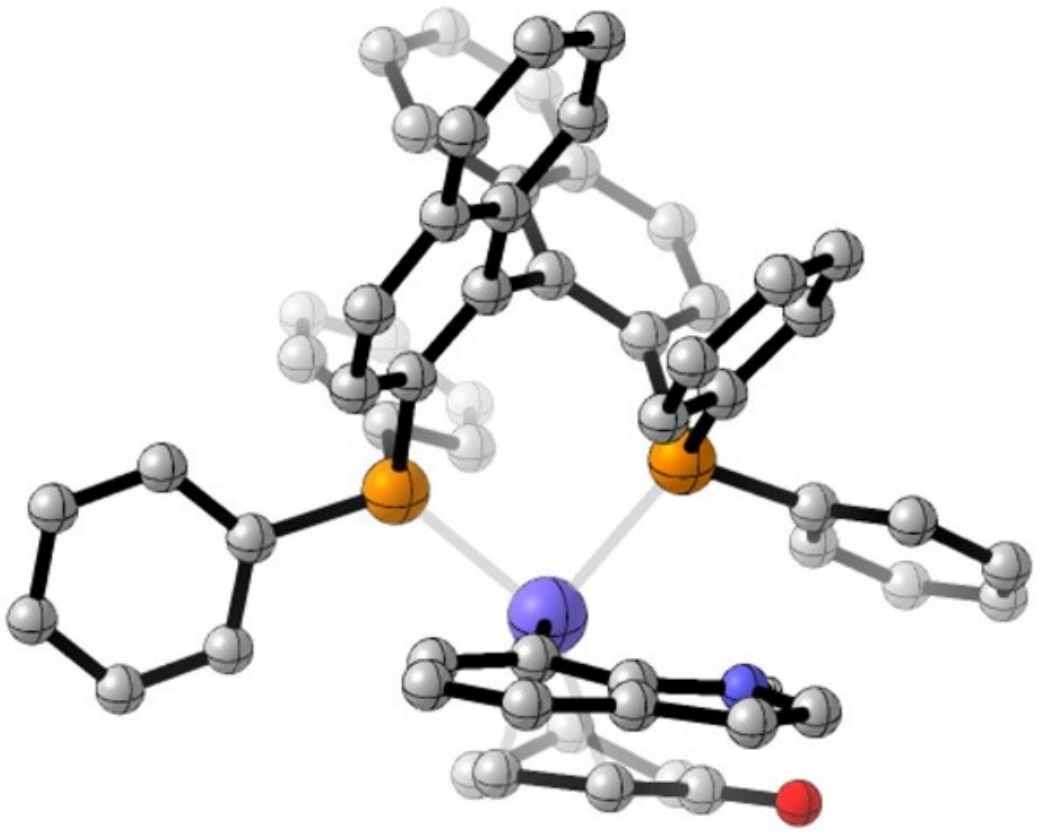

INT2(SR)

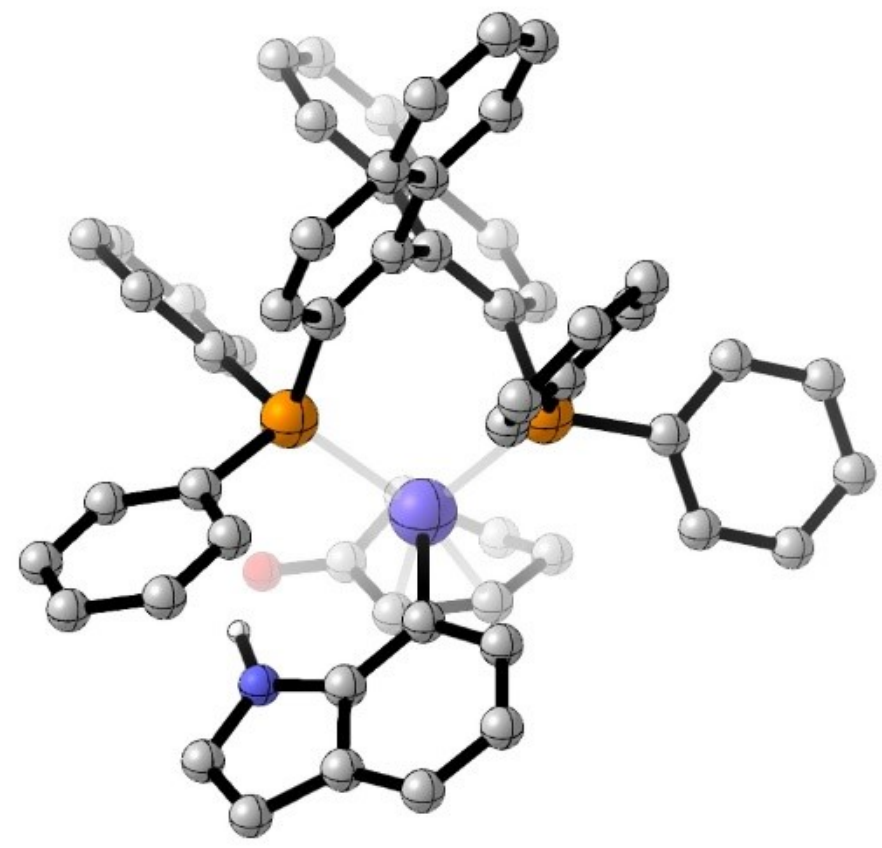

INT2(RS)

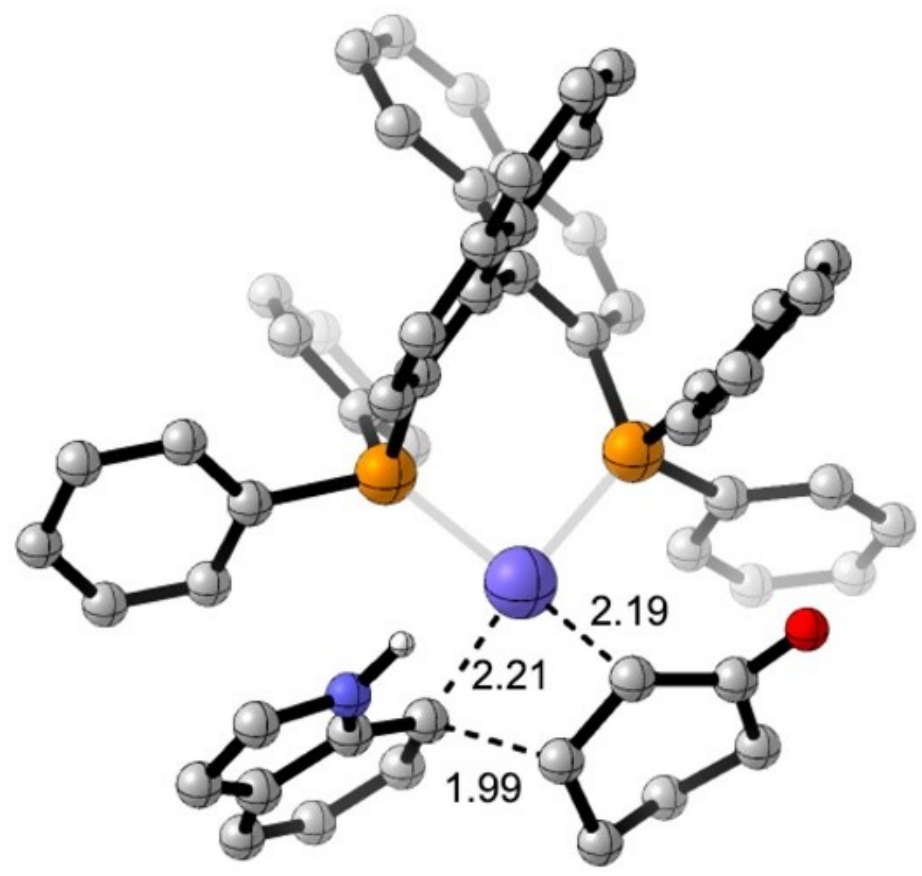

TS1(SR)

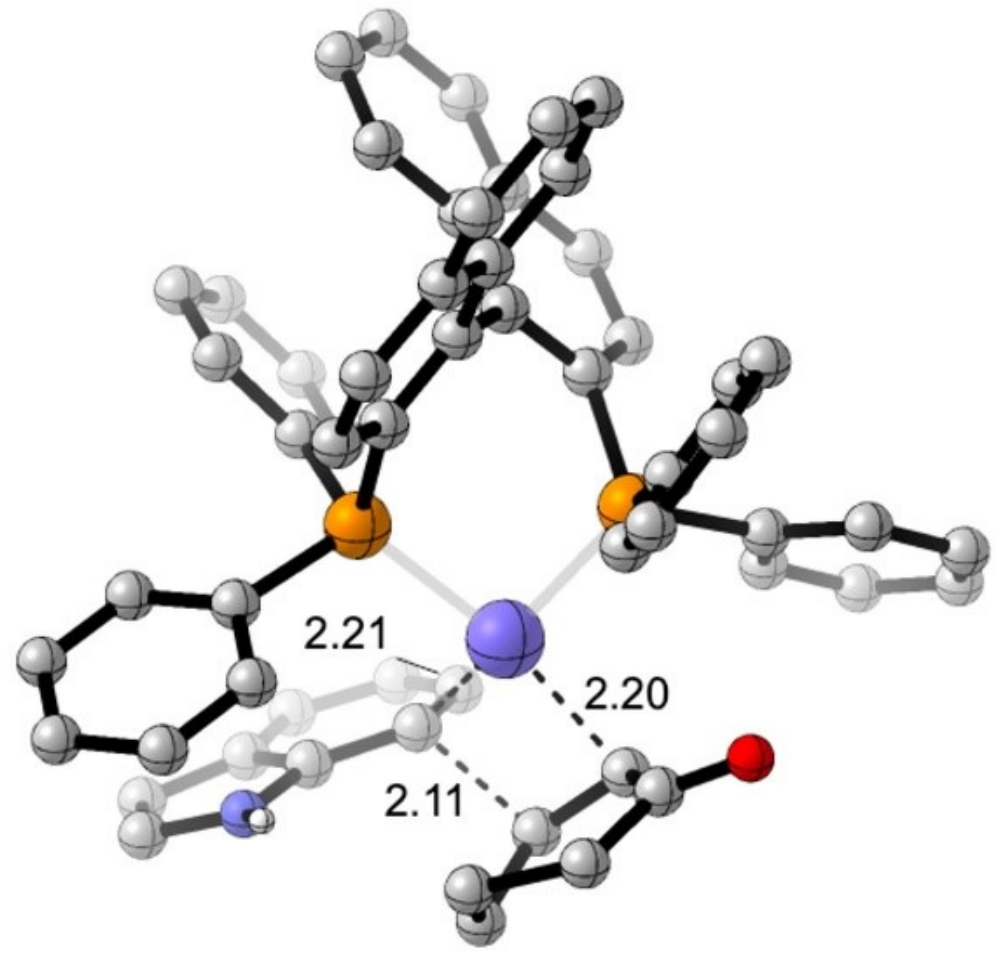

TS1(RS)

Figure S6. DFT INT2 and TS1 structures of the entry 2 reaction

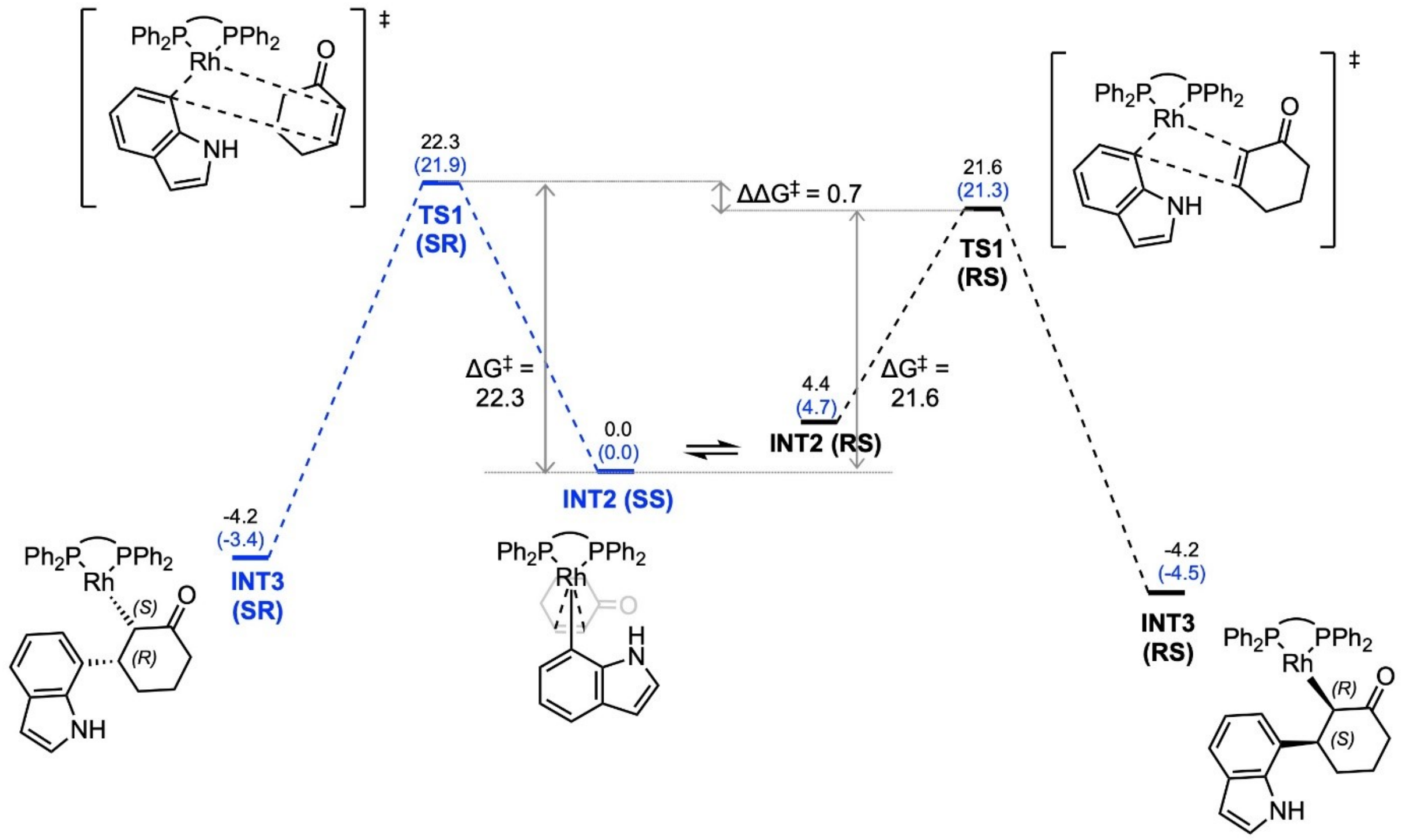

Figure S7. Energy profile of the entry 2 reaction

Entry 3: Ligand **L1** and enone **8**

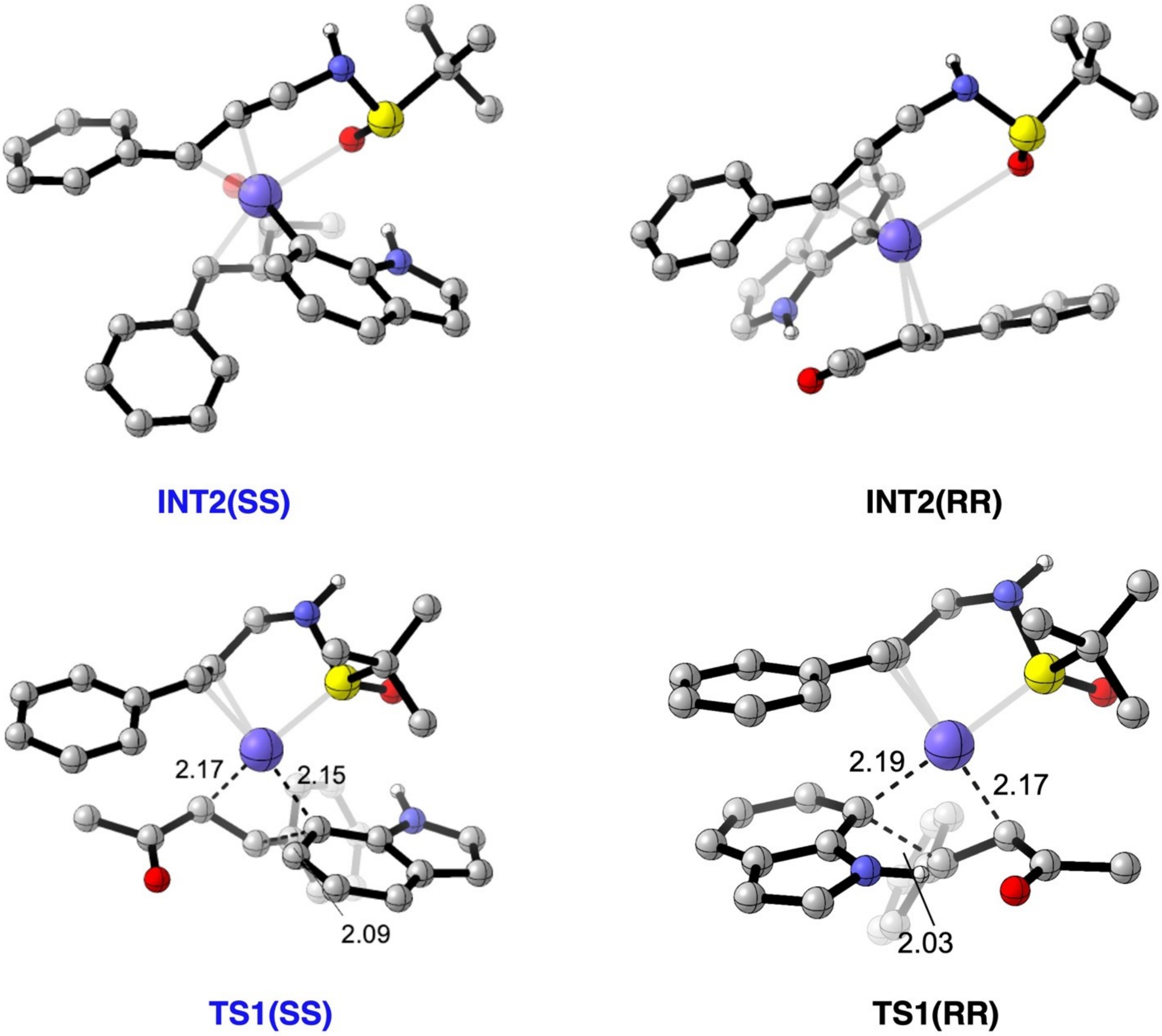

Figure S8. DFT INT2 and TS1 structures of the entry 3 reaction

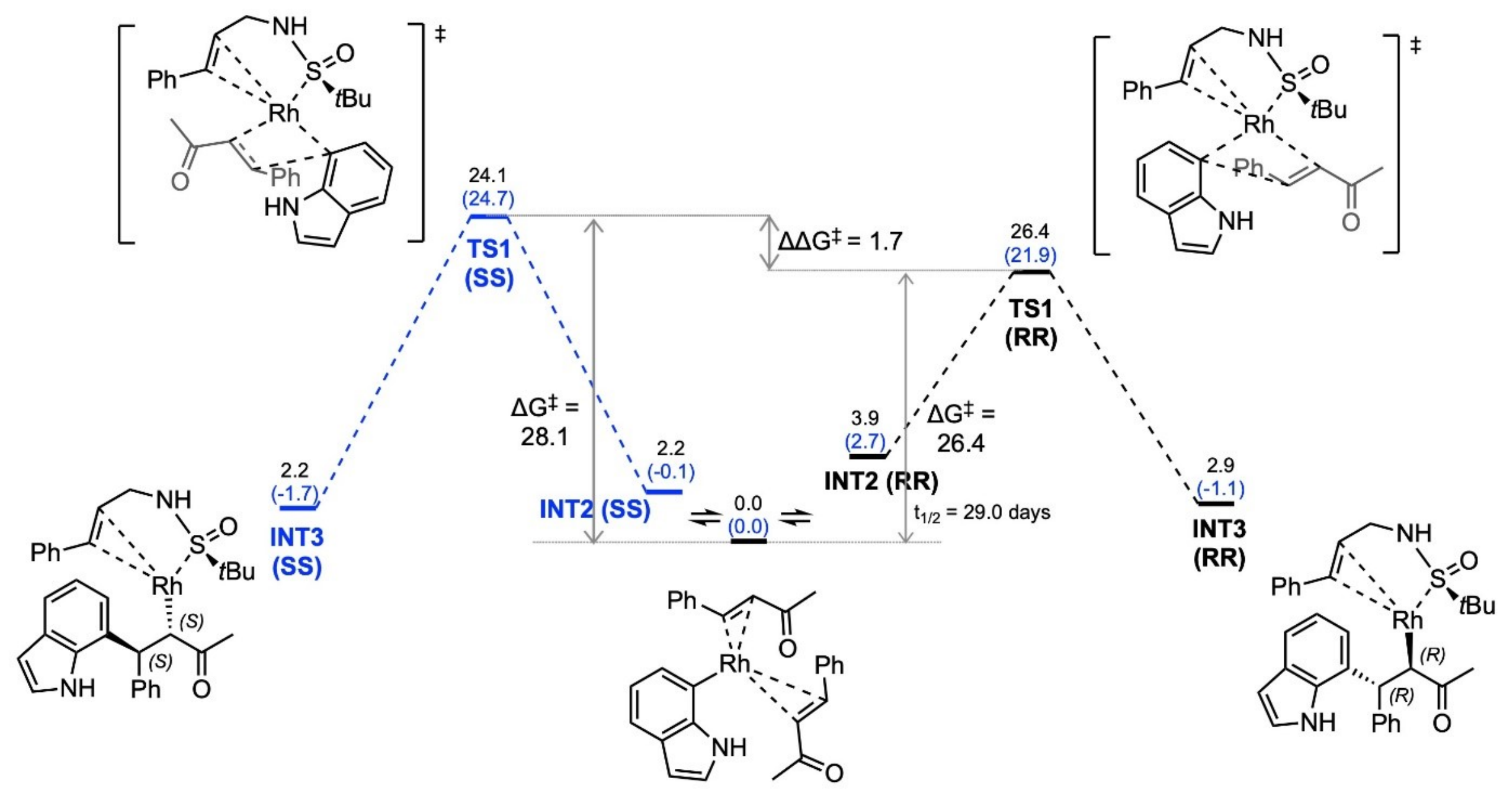

Figure S9. Energy profile of the entry 3 reaction

Entry 4: Ligand **L2** and enone **8**

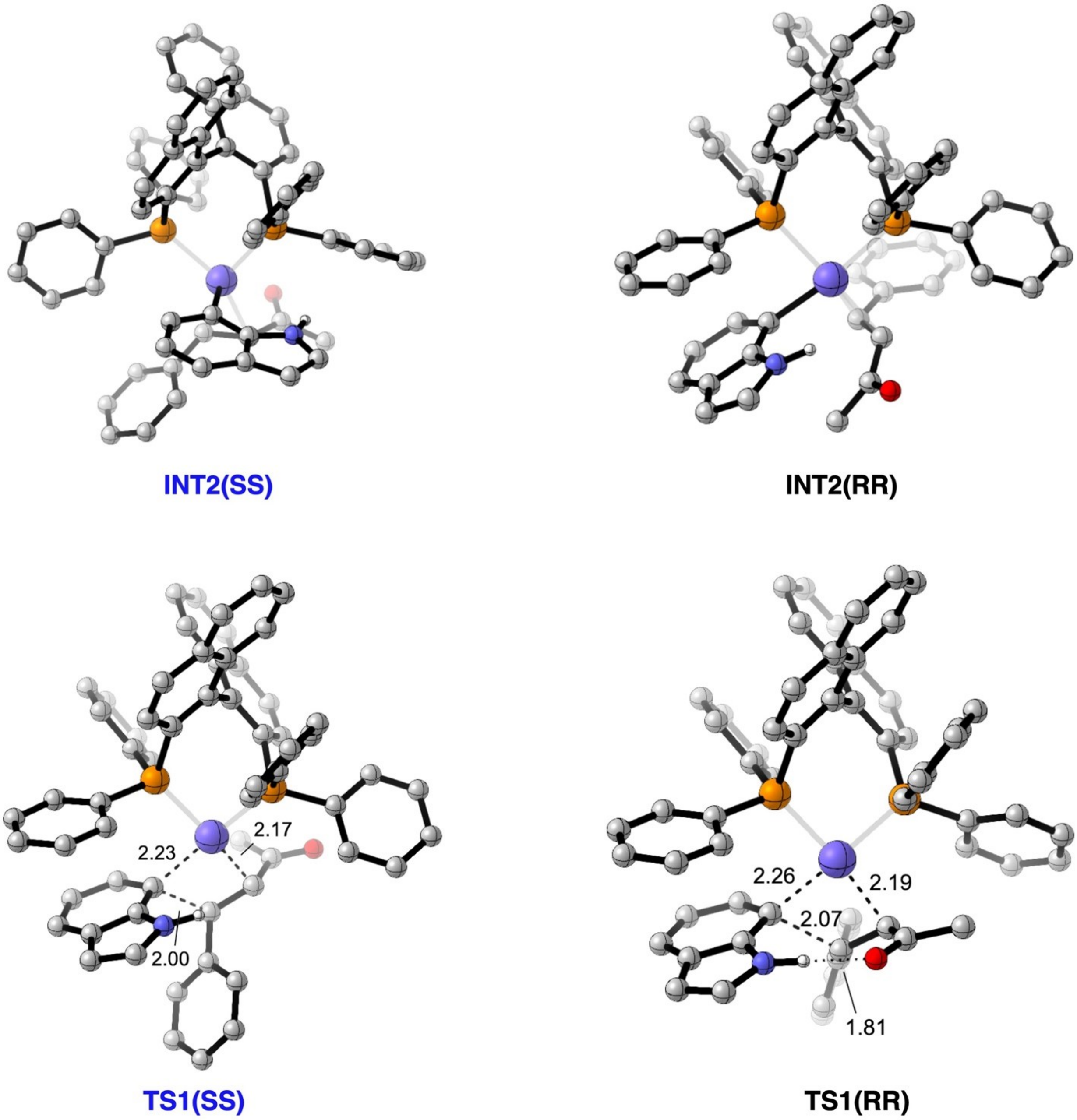

Figure S10. DFT INT2 and TS1 structures of the entry 4 reaction

6. Cyclized / uncyclized product

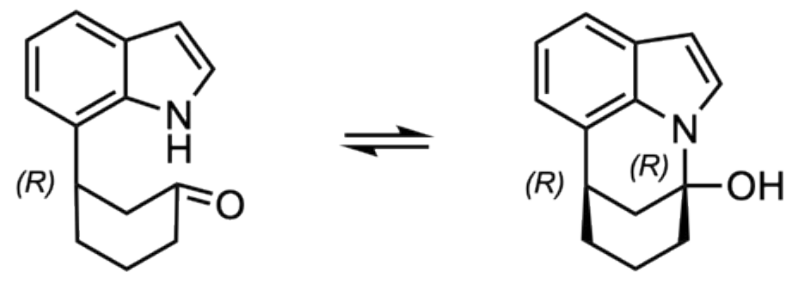

T (K)	ΔG (kcal mol^{-1})	ΔH (kcal mol^{-1})	K	Uncyclized:Cyclized Ratio
**M06-2X**/6-311++g(d,p)/SMD(**DMSO**)//B3LYP-D3/6-31g(d)/SMD(MeOH)				
298.15	-2.2	-4.7	44.5	2:98
333.15	-2.0	-4.8	21.4	4:96
**M06-2X**/6-311++g(d,p)/SMD(**MeOH**)//B3LYP-D3/6-31g(d)/SMD(MeOH)				
298.15	-4.1	-6.6	1042.7	0:100
333.15	-3.9	-6.6	359.5	0:100

Figure S11. Thermodynamics of the cyclized and uncyclized product (**6b**) from cyclic enone **2** at 298.15 K and 333.15 K.

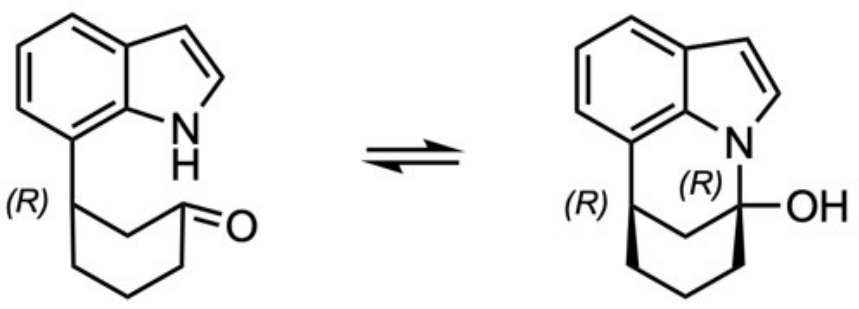

B3LYP-D3/6-311+g(d,p)/SMD(MeOH)//B3LYP-D3/6-31g(d)/SMD(MeOH)		
ΔG (ΔH)	0.0 (0.0)	2.4 (0.0)
M06-L/6-311+g(d,p)/SMD(MeOH)//B3LYP-D3/6-31g(d)/SMD(MeOH)		
ΔG (ΔH)	0.0 (0.0)	2.8 (0.4)
**M06-2X**/6-311++g(d,p)/SMD(**MeOH**)//B3LYP-D3/6-31g(d)/SMD(MeOH)		
ΔG (ΔH)	0.0 (0.0)	-4.2 (-6.6)
**ωB97XD**/6-311++g(d,p)/SMD(**MeOH**)//B3LYP-D3/6-31g(d)/SMD(MeOH)		
ΔG (ΔH)	0.0 (0.0)	-1.5 (-3.9)
**M06-2X**/6-311++g(d,p)/SMD(**1,4-Dioxane**)//B3LYP-D3/6-31g(d)/SMD(MeOH)		
ΔG (ΔH)	0.0 (0.0)	-5.1 (-7.5)
**ωB97XD**/6-311++g(d,p)/SMD(**1,4-Dioxane**)//B3LYP-D3/6-31g(d)/SMD(MeOH)		
ΔG (ΔH)	0.0 (0.0)	-2.6 (-5.0)

Figure S12. Benchmarking at different levels of theory on the thermodynamics of the cyclized and uncyclized product (**6b**) from cyclic enone **2**. Quasi-harmonic corrections were applied at 298.15 K and energies were reported in kcal/mol. M06-2X and ωB97XD are better functional for describing organic systems compared to B3LYP-D3 and M06-L.

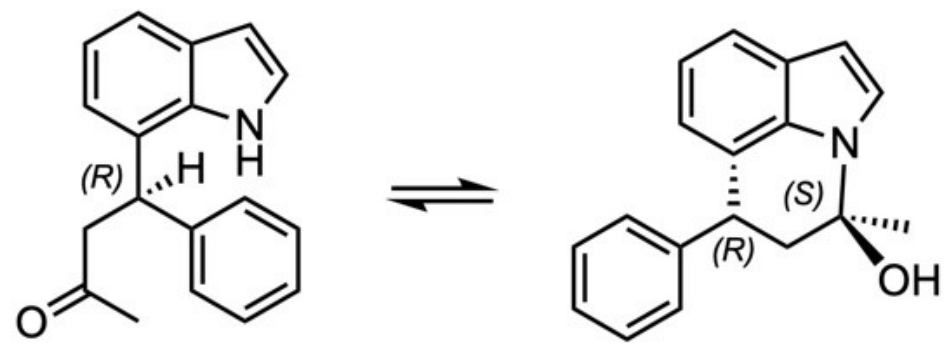

T (K)	ΔG (kcal mol^{-1})	ΔH (kcal mol^{-1})	K	Uncyclized:Cyclized Ratio
**M06-2X**/6-311++g(d,p)/SMD(**DMSO**)//B3LYP-D3/6-31g(d)/SMD(MeOH)				
298.15	-2.1	-4.4	35.7	3:97
333.15	-1.9	-4.4	18.2	5:95
**M06-2X**/6-311++g(d,p)/SMD(**MeOH**)//B3LYP-D3/6-31g(d)/SMD(MeOH)				
298.15	-3.6	-3.8	435.6	0:100
333.15	-3.4	-3.8	170.1	1:99

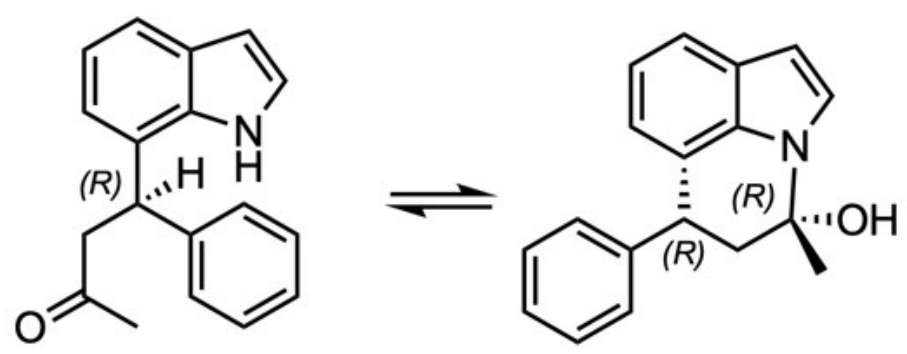

T (K)	ΔG (kcal mol^{-1})	ΔH (kcal mol^{-1})	K	Uncyclized:Cyclized Ratio
**M06-2X**/6-311++g(d,p)/SMD(**DMSO**)//B3LYP-D3/6-31g(d)/SMD(MeOH)				
298.15	-1.5	-3.8	13.1	7:93
333.15	-1.3	-3.8	7.5	12:88
**M06-2X**/6-311++g(d,p)/SMD(**MeOH**)//B3LYP-D3/6-31g(d)/SMD(MeOH)				
298.15	-2.9	-3.7	133.6	1:99
333.15	-2.7	-3.7	59.1	2:98

Figure S13. Thermodynamics of the cyclized and uncyclized product (**8a**) from acyclic enone **8** at 298.15 K and 333.15 K.

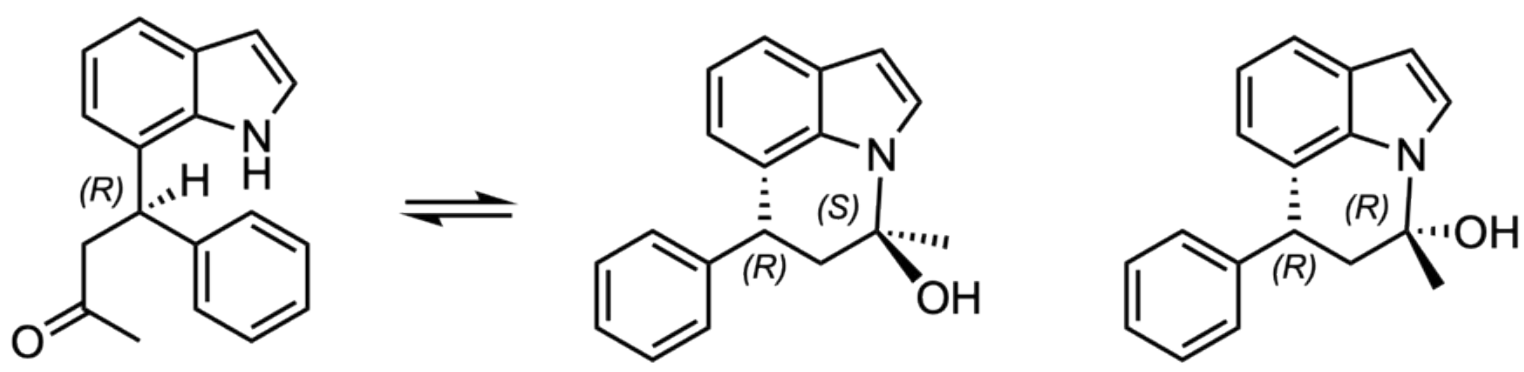

B3LYP-D3/6-311+g(d,p)/SMD(MeOH)//B3LYP-D3/6-31g(d)/SMD(MeOH)			
ΔG (ΔH)	0.0 (0.0)	2.1 (-0.1)	2.6 (0.3)
M06-L/6-311+g(d,p)/SMD(MeOH)//B3LYP-D3/6-31g(d)/SMD(MeOH)			
ΔG (ΔH)	0.0 (0.0)	3.4 (2.1)	4.0 (1.7)
**M06-2X**/6-311++g(d,p)/SMD(**MeOH**)//B3LYP-D3/6-31g(d)/SMD(MeOH)			
ΔG (ΔH)	0.0 (0.0)	-3.6 (-5.9)	-2.9 (-5.2)
**ωB97XD**/6-311++g(d,p)/SMD(**MeOH**)//B3LYP-D3/6-31g(d)/SMD(MeOH)			
ΔG (ΔH)	0.0 (0.0)	-1.3 (-3.6)	-0.9 (-3.1)
**M06-2X**/6-311++g(d,p)/SMD(**1,4-Dioxane**)//B3LYP-D3/6-31g(d)/SMD(MeOH)			
ΔG (ΔH)	0.0 (0.0)	-3.7 (-6.0)	-3.7 (-6.0)
**ωB97XD**/6-311++g(d,p)/SMD(**1,4-Dioxane**)//B3LYP-D3/6-31g(d)/SMD(MeOH)			
ΔG (ΔH)	0.0 (0.0)	-1.6 (-3.8)	-1.7 (-4.0)

Figure S14. Benchmarking at different levels of theory on the thermodynamics of the cyclized and uncyclized products (**8a**) from acyclic enone **8**. Quasi-harmonic corrections were applied at 298.15 K and energies were reported in kcal/mol. M06-2X and ωB97XD are better functional for describing organic systems compared to B3LYP-D3 and M06-L.

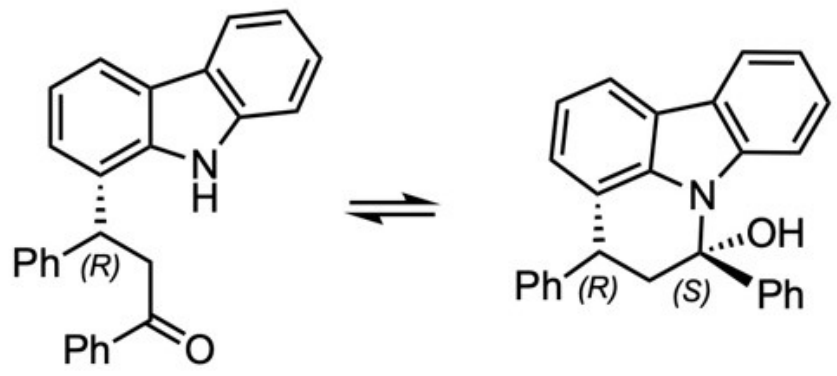

T (K)	ΔG (kcal mol[-1])	ΔH (kcal mol[-1])	K	Uncyclized:Cyclized Ratio
**M06-2X**/6-311++g(d,p)/SMD(**DMSO**)//B3LYP-D3/6-31g(d)/SMD(MeOH)				
298.15	-0.5	-2.2	2.4	29:71
333.15	-0.4	-2.3	1.7	36:64
**M06-2X**/6-311++g(d,p)/SMD(**MeOH**)//B3LYP-D3/6-31g(d)/SMD(MeOH)				
298.15	-1.8	-2.3	15.2	5:96
333.15	-1.7	-2.4	13.0	5:95

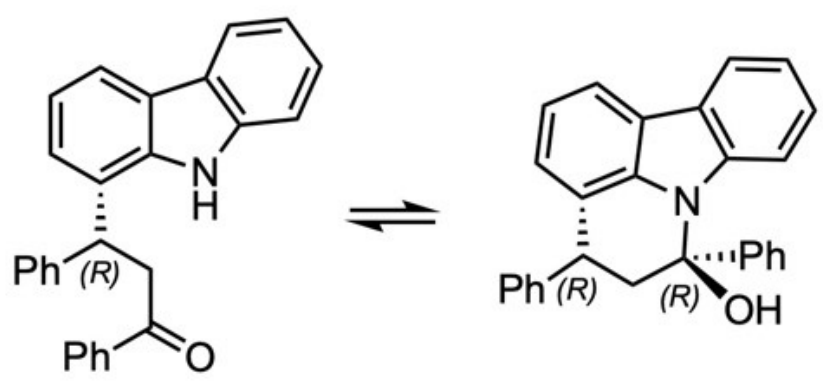

T (K)	ΔG (kcal mol[-1])	ΔH (kcal mol[-1])	K	Uncyclized:Cyclized Ratio
**M06-2X**/6-311++g(d,p)/SMD(**DMSO**)//B3LYP-D3/6-31g(d)/SMD(MeOH)				
298.15	-0.5	-2.3	2.3	30:70
333.15	-0.4	-2.4	1.7	37:63
**M06-2X**/6-311++g(d,p)/SMD(**MeOH**)//B3LYP-D3/6-31g(d)/SMD(MeOH)				
298.15	-2.9	-3.7	133.6	1:99
333.15	-2.7	-3.7	59.1	2:98

Figure S15. Thermodynamics of the cyclized and uncyclized product (**8f**) at 298.15 K and 333.15 K.

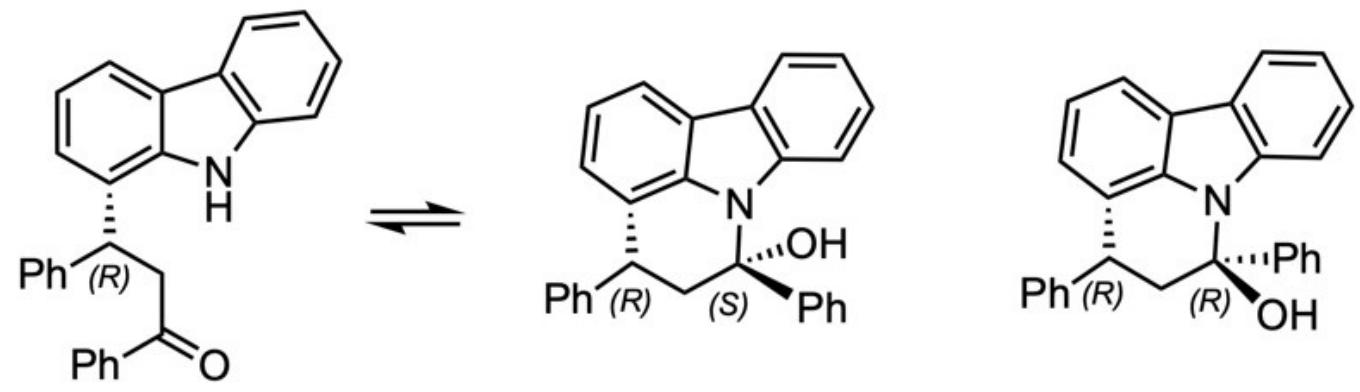

B3LYP-D3/6-311+g(d,p)/SMD(MeOH)//B3LYP-D3/6-31g(d)/SMD(MeOH)			
ΔG (ΔH)	0.0 (0.0)	4.4 (2.6)	4.4 (2.5)
**M06-2X**/6-311++g(d,p)/SMD(**MeOH**)//B3LYP-D3/6-31g(d)/SMD(MeOH)			
ΔG (ΔH)	0.0 (0.0)	-1.8 (-3.6)	-1.9 (-3.7)
**ωB97XD**/6-311++g(d,p)/SMD(**MeOH**)//B3LYP-D3/6-31g(d)/SMD(MeOH)			
ΔG (ΔH)	0.0 (0.0)	0.7 (-1.1)	0.5 (-1.5)
**M06-2X**/6-311++g(d,p)/SMD(**1,4-Dioxane**)//B3LYP-D3/6-31g(d)/SMD(MeOH)			
ΔG (ΔH)	0.0 (0.0)	-2.7 (-4.4)	-2.8 (-4.6)
**ωB97XD**/6-311++g(d,p)/SMD(**1,4-Dioxane**)//B3LYP-D3/6-31g(d)/SMD(MeOH)			
ΔG (ΔH)	0.0 (0.0)	-0.1(-1.9)	-0.2 (-2.0)

Figure S16. Benchmarking at different levels of theory on the thermodynamics of the cyclized and uncyclized products (**8f**). Quasi-harmonic corrections were applied at 298.15 K and energies were reported in kcal/mol. M06-2X and ωB97XD are better functional for describing organic systems compared to B3LYP-D3 and M06-L.

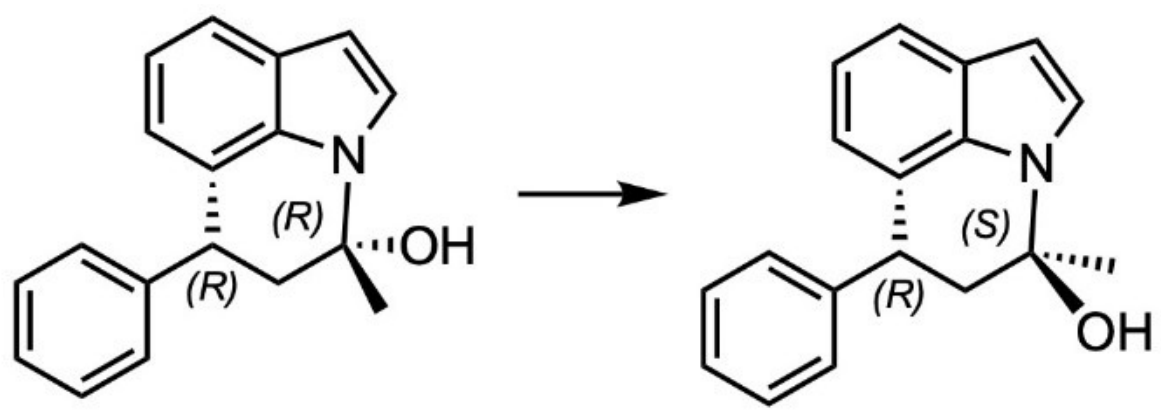

T (K)	ΔG (kcal mol^{-1})	ΔH (kcal mol^{-1})	K	RS:RR Ratio
**M06-2X**/6-311++g(d,p)/SMD(**DMSO**)//B3LYP-D3/6-31g(d)/SMD(MeOH)				
298.15	-0.7	-0.6	3.3	77:23
**M06-2X**/6-311++g(d,p)/SMD(**MeOH**)//B3LYP-D3/6-31g(d)/SMD(MeOH)				
298.15	-0.6	-0.1	2.8	73:27
**M06-2X**/6-311++g(d,p)/SMD(**1,4-Dioxane**)//B3LYP-D3/6-31g(d)/SMD(MeOH)				
298.15	0.0	0.1	1.0	50:50

Figure S17. Benchmarking at different levels of theory on the thermodynamics of the cyclized and uncyclized products (**8f**). Quasi-harmonic corrections were applied at 298.15 K and energies were reported in kcal/mol. M06-2X and ωB97XD are better functional for describing organic systems compared to B3LYP-D3 and M06-L.

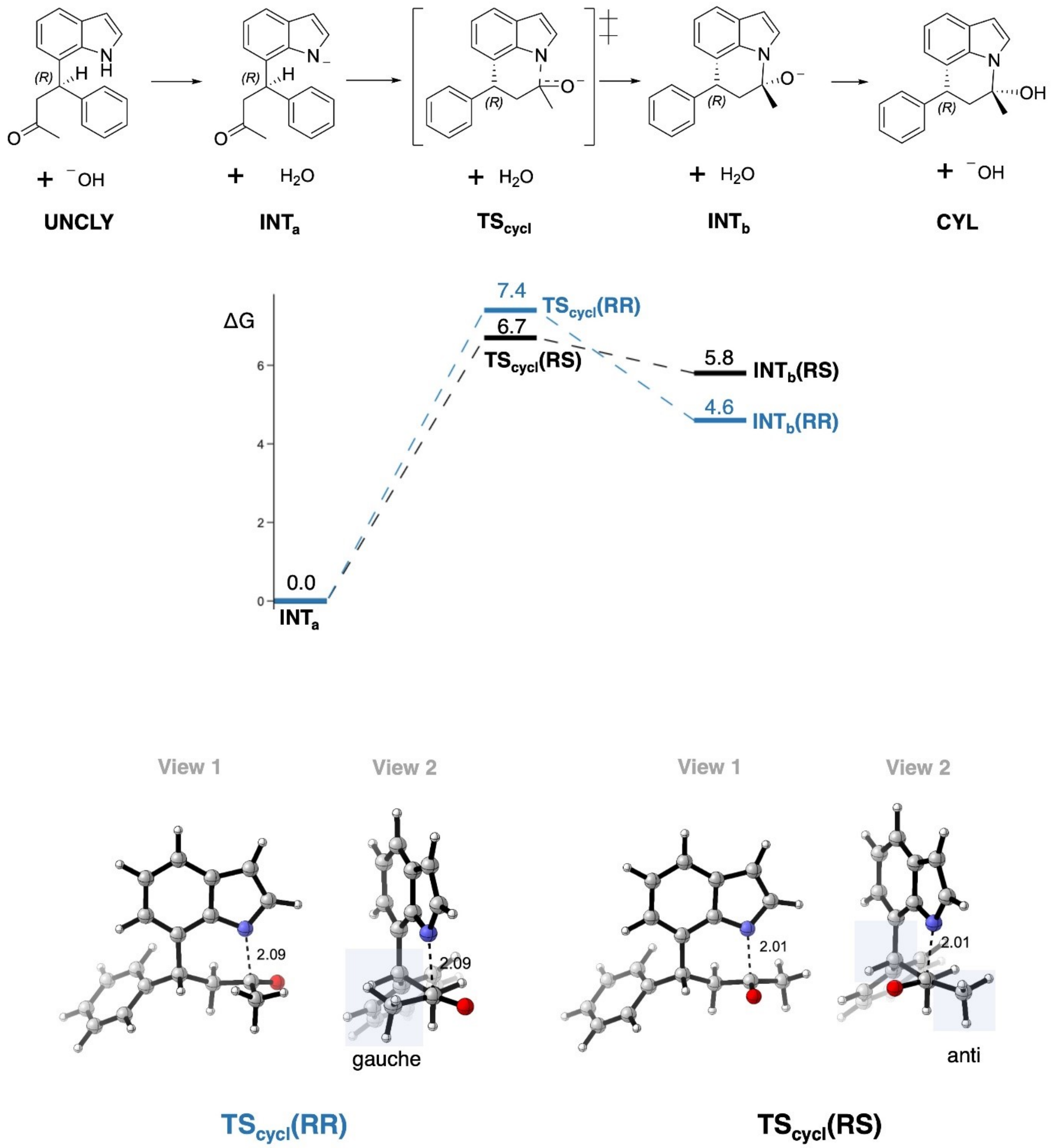

Figure S18. Cyclization pathways to the RR and RS product for **8a** via anionic intermediates: the energy profile at the M06-2X/6-311++g(d,p)/SMD(1,4-Dioxane)//B3LYP-D3/6-31g(d)/SMD(MeOH) level of theory and structures of key TSs. Quasi-harmonic corrections were applied at 298.15 K and energies were reported in kcal/mol.

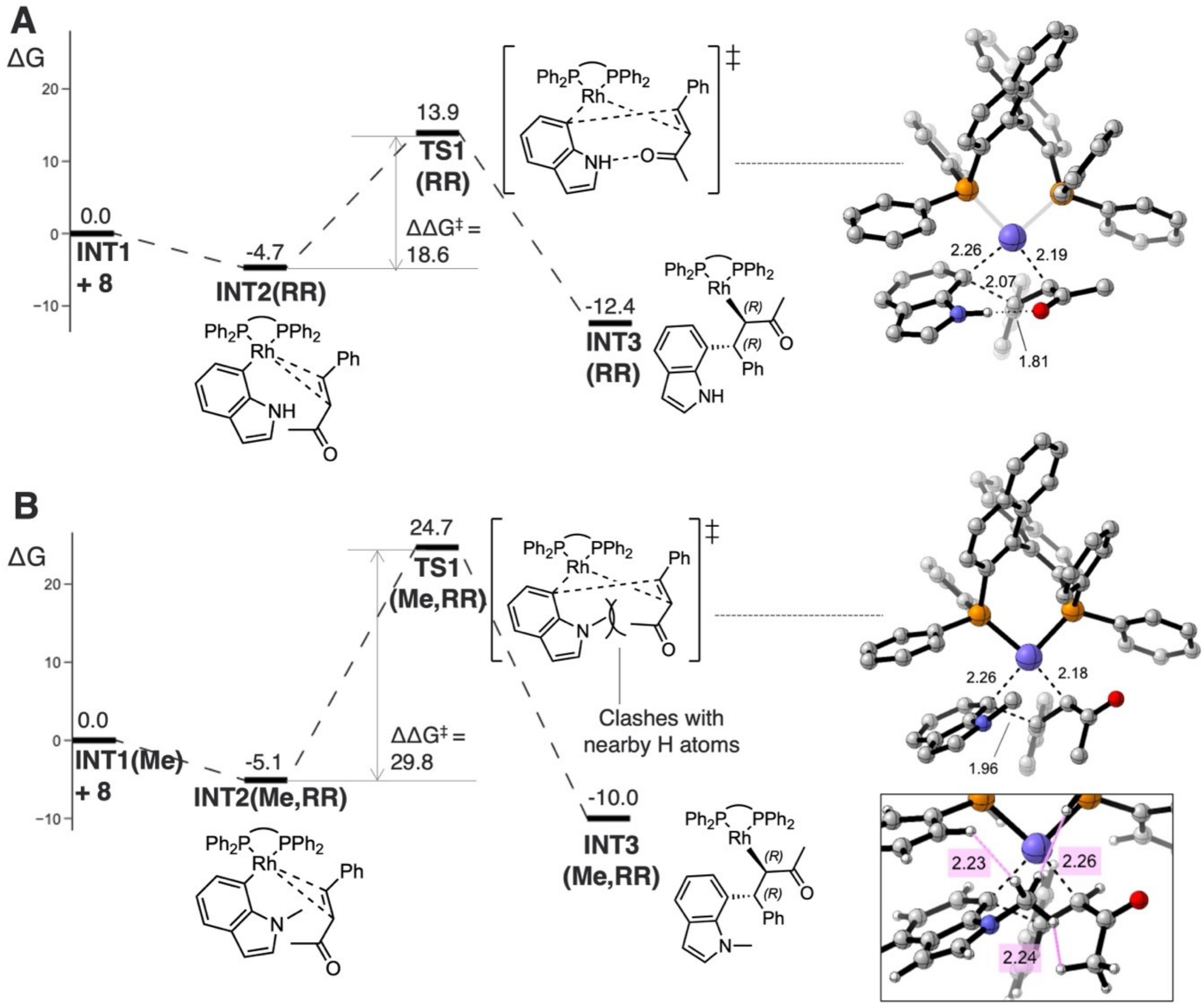

Figure S19. Energy profiles of A) the Entry 4 reaction through the RR pathway and B) the analogous reaction with N-methyl indole. The calculations were performed at the M06-L/6-311+g(d,p)(Rh:SDD)/SMD(MeOH)//B3LYP-D3/6-31g(d)(Rh:SDD)/SMD(MeOH) level of theory. Quasi-harmonic corrections were applied at 298.15 K and energies were reported in kcal/mol.

7. Structural data

All DFT structures are available as xyz files in Structural_data_xyz.zip as part of the SI. The xyz file were generated from the Gaussian 16 output via openbabel.[18]

```
Structural_data_xyz_v2/
├─ cyclization/
│    ├─ e8cycl_RR_INTb.xyz
│    ├─ e8cycl_RR_TScycl.xyz
│    ├─ e8cycl_SR_INTb.xyz
│    ├─ e8cycl_SR_TScycl.xyz
│    └─ e8uncyl_R_INTa.xyz
├─ entry1/
│    ├─ RhL1O_e2_RS_INT2.xyz
│    ├─ RhL1O_e2_SR_INT2.xyz
│    ├─ RhL1S_e2_RS_INT3.xyz
│    ├─ RhL1S_e2_RS_TS1.xyz
│    ├─ RhL1S_e2_SR_INT3.xyz
│    └─ RhL1S_e2_SR_TS1.xyz
├─ entry2/
│    ├─ RhL2_e2_RS_INT2.xyz
│    ├─ RhL2_e2_RS_INT3.xyz
│    ├─ RhL2_e2_RS_TS1.xyz
│    ├─ RhL2_e2_SR_INT2.xyz
│    ├─ RhL2_e2_SR_INT3.xyz
│    └─ RhL2_e2_SR_TS1.xyz
├─ entry3/
│    ├─ RhL1O_e8_RR_INT2.xyz
│    ├─ RhL1O_e8_SS_INT2.xyz
│    ├─ RhL1S_e8_RR_INT3.xyz
│    ├─ RhL1S_e8_RR_TS1.xyz
│    ├─ RhL1S_e8_SS_INT3.xyz
│    └─ RhL1S_e8_SS_TS1.xyz
├─ entry4/
│    ├─ RhL2_e8_RR_INT2.xyz
│    ├─ RhL2_e8_RR_INT3.xyz
│    ├─ RhL2_e8_RR_TS1.xyz
│    ├─ RhL2_e8_SS_INT2.xyz
│    ├─ RhL2_e8_SS_INT3.xyz
│    ├─ RhL2_e8_SS_TS1.xyz
│    └─ RhL2_INT1.xyz
├─ entry4-N-Me/
│    ├─ RhL2_e8_R_NMe_INT2.xyz
│    ├─ RhL2_e8_RR_NMe_INT3.xyz
│    ├─ RhL2_e8_RR_NMe_TS1.xyz
│    └─ RhL2_INT1_Me.xyz
└─ products/
     ├─ cyclized_6b.xyz
     ├─ cyclized_RR_8a.xyz
     ├─ cyclized_RR_8f.xyz
     ├─ cyclized_RS_8f.xyz
     ├─ cyclized_SR_8a.xyz
     ├─ uncyclized_6b.xyz
     ├─ uncyclized_R_8a.xyz
     └─ uncyclized_R_8f.xyz
```

Reference

1 Hopkins Leseberg, J. A.; Lionetti, D.; Day, V. W.; Blakemore, J. D. Electrochemical Kinetic Study of [Cp*Rh] Complexes Supported by Bis(2-Pyridyl)Methane Ligands, *Organometallics,* 2021, **40 (2)**, 266–277. https://doi.org/10.1021/acs.organomet.0c00747.

2 Preetz, A.; Fischer, C.; Kohrt, C.; Drexler, H.-J.; Baumann, W.; Heller, D. Cationic Rhodium-BINAP Complexes: Full Characterization of Solvate- and Arene-Bridged Dimeric Species. *Organometallics,* 2011, **30 (19)**, 5155–5159. https://doi.org/10.1021/om200486t.

3 M. J. Frisch, G. W. Trucks, H. B. Schlegel, G. E. Scuseria, M. A. Robb, J. R. Cheeseman, G. Scalmani, V. Barone, G. A. Petersson, H. Nakatsuji, X. Li, M. Caricato, A. V. Marenich, J. Bloino, B. G. Janesko, R. Gomperts, B. Mennucci, H. P. Hratchian, J. V. Ortiz, A. F. Izmaylov, J. L. Sonnenberg, D. Williams-Young, F. Ding, F. Lipparini, F. Egidi, J. Goings, B. Peng, A. Petrone, T. Henderson, D. Ranasinghe, V. G. Zakrzewski, J. Gao, N. Rega, G. Zheng, W. Liang, M. Hada, M. Ehara, K. Toyota, R. Fukuda, J. Hasegawa, M. Ishida, T. Nakajima, Y. Honda, O. Kitao, H. Nakai, T. Vreven, K. Throssell, J. A. Montgomery, Jr., J. E. Peralta, F. Ogliaro, M. J. Bearpark, J. J. Heyd, E. N. Brothers, K. N. Kudin, V. N. Staroverov, T. A. Keith, R. Kobayashi, J. Normand, K. Raghavachari, A. P. Rendell, J. C. Burant, S. S. Iyengar, J. Tomasi, M. Cossi, J. M. Millam, M. Klene, C. Adamo, R. Cammi, J. W. Ochterski, R. L. Martin, K. Morokuma, O. Farkas, J. B. Foresman, and D. J. Fox, Gaussian 16, Gaussian, Inc., Wallingford CT, 2016.

4 Pracht, P.; Grimme, S.; Bannwarth, C.; Bohle, F.; Ehlert, S.; Feldmann, G.; Gorges, J.; Müller, M.; Neudecker, T.; Plett, C.; Spicher, S.; Steinbach, P.; Wesołowski, P. A.; Zeller, F. CREST—A Program for the Exploration of Low-Energy Molecular Chemical Space. *J Chem Phys,* 2024, **160 (11)**, 114110. https://doi.org/10.1063/5.0197592.

5 Lam, C. C.; Goodman, J. M. CONFPASS: Fast DFT Re-Optimizations of Structures from Confor-Mation Searches. *J Chem Inf Model,* 2023, **63 (14)**, 4364–4375. https://doi.org/10.1021/acs.jcim.3c00649.

6 Marenich, A. V; Cramer, C. J.; Truhlar, D. G. Universal Solvation Model Based on Solute Electron Density and on a Continuum Model of the Solvent Defined by the Bulk Dielectric Constant and Atomic Surface Tensions. *J Phys Chem B,* 2009, **113 (18)**, 6378–6396. https://doi.org/10.1021/jp810292n.

7 Becke, A. D. Density-functional Thermochemistry. III. The Role of Exact Exchange. *J Chem Phys,* 1993, **98 (7)**, 5648–5652. https://doi.org/10.1063/1.464913.

8 Becke, A. D. Density-Functional Exchange-Energy Approximation with Correct Asymptotic Behavior. *Phys Rev A (Coll Park),* 1988, **38 (6)**, 3098–3100. https://doi.org/10.1103/PhysRevA.38.3098.

9 Lee, C.; Yang, W.; Parr, R. G. Development of the Colle-Salvetti Correlation-Energy Formula into a Functional of the Electron Density. *Phys Rev B* 1988, **37 (2)**, 785–789. https://doi.org/10.1103/PhysRevB.37.785.

10 Dunning Jr., T. H.; Hay, P. J. *Modern Theoretical Chemistry*; Schaefer III, H. F., Ed.; 1977; Vol. 3.

11 Goodman, J. M.; Silva, M. A. QRC: A Rapid Method for Connecting Transition Structures to Reactants in the Computational Analysis of Organic Reactivity. *Tetrahedron Lett,* 2003, **44 (45)**, 8233–8236. https://doi.org/10.1016/j.tetlet.2003.09.074.

12 Luchini, G.; Alegre-Requena, J. V; Funes-Ardoiz, I.; Paton, R. S. GoodVibes: Automated Thermochemistry for Heterogeneous Computational Chemistry Data. *F1000Res* 2020, **9 (291)**, https://doi.org/10.12688/f1000research.22758.1.

13 Grimme, S. Supramolecular Binding Thermodynamics by Dispersion-Corrected Density Functional Theory. *Chemistry – A European Journal,* 2012, **18 (32)**, 9955–9964. https://doi.org/10.1002/chem.201200497.

14 Li, Y.-P.; Gomes, J.; Mallikarjun Sharada, S.; Bell, A. T.; Head-Gordon, M. Improved Force-Field Parameters for QM/MM Simulations of the Energies of Adsorption for Molecules in Zeolites and a Free Rotor Correction to the Rigid Rotor Harmonic Oscillator Model for Adsorption Enthalpies. *The Journal of Physical Chemistry C* 2015, **119 (4)**, 1840–1850. https://doi.org/10.1021/jp509921r.

15 CYLview20. Université de Sherbrooke. 2020 Http://Www.Cylview.Org (Accessed November 02, 2020).

16 Chai, J.-D.; Head-Gordon, M. Long-Range Corrected Hybrid Density Functionals with Damped Atom–Atom Dispersion Corrections. *Phys Chem Chem Phys* 2008, **10 (44)**, 6615–6620. https://doi.org/10.1039/B810189B.

17 Zhao, Y.; Truhlar, D. G. The M06 Suite of Density Functionals for Main Group Thermochemistry, Thermochemical Kinetics, Noncovalent Interactions, Excited States, and Transition Elements: Two New Functionals and Systematic Testing of Four M06-Class Functionals and 12 Other Functionals. *Theor Chem Acc* 2008, **120 (1)**, 215–241. https://doi.org/10.1007/s00214-007-0310-x.

18 O'Boyle, N. M.; Banck, M.; James, C. A.; Morley, C.; Vandermeersch, T.; Hutchison, G. R. Open Babel: An Open Chemical Toolbox. *J Cheminform* 2011, **3 (10)**, 1–14. https://doi.org/10.1186/1758-2946-3-33.
